# FoxP3-miR-150-5p/3p suppresses ovarian tumorigenesis via an IGF1R/IRS1 pathway feedback loop

**DOI:** 10.1038/s41419-021-03554-6

**Published:** 2021-03-15

**Authors:** Qinkai Zhang, Xunzhu Zhou, Maoping Wan, Xixi Zeng, Jiarong Luo, Yesha Xu, Liying Ji, Jian-An Zhang, Pei Fan, Jianing Zhong, Jianmin Wu

**Affiliations:** 1 Institute of Genomic Medicine, Wenzhou Medical University, Wenzhou, Zhejiang P.R. China; 2 Department of Obstetrics and Gynecology, The Second Affiliated Hospital and Yuying Children’s Hospital of Wenzhou Medical University, Wenzhou, Zhejiang P.R. China; 3 Department of Orthopedics, The Second Affiliated Hospital and Yuying Children’s Hospital of Wenzhou Medical University, Wenzhou, Zhejiang P.R. China; 4 Key Laboratory of Prevention and Treatment of Cardiovascular and Cerebrovascular Diseases, Ministry of Education, Gannan Medical University, Ganzhou, Jiangxi P.R. China

**Keywords:** Cancer, Translational research

## Abstract

Ovarian cancer (OC) causes more deaths than any other gynecological cancer. Many cellular pathways have been elucidated to be associated with OC development and progression. Specifically, the insulin-like growth factor 1 receptor/insulin receptor substrate 1 (IGF1R/IRS1) pathway participates in OC development. Moreover, accumulating evidence has shown that microRNA deregulation contributes to tumor initiation and progression. Here, our study aimed to investigate the molecular functions and regulatory mechanisms of miR-150, specifically, in OC. We found that the expression of miR-150-5p/3p and their precursor, mir-150, was downregulated in OC tissues; lower mir-150 levels were associated with poor OC patient outcomes. Ectopic mir-150 expression inhibited OC cell growth and metastasis in vitro and in vivo. Furthermore, both IRS1 and IGF1R were confirmed as direct targets of miR-150-5p/3p, and the miR-150-IGF1R/IRS1 axis exerted antitumor effects via the PI3K/AKT/mTOR pathway. Forkhead box protein 3 (FoxP3) positively regulated the expression of miR-150-5p/3p by binding to the mir-150 promoter. In turn, the PI3K/AKT/mTOR pathway downregulated FoxP3 and miR-150-5p/3p. Taken together, these findings indicate that a complex FoxP3-miR-150-IGF1R/IRS1-PI3K/AKT/mTOR feedback loop regulates OC pathogenesis, providing a novel mechanism for miR-150 as a tumor suppressor miRNA in OC.

## Introduction

Ovarian cancer (OC) is reported to be the most lethal gynecological cancer^1,2^. Worldwide, approximately 295,400 cases of OC and 184,800 OC-related deaths were reported in 2018, presenting a case-to-fatality ratio nearly twice that of breast cancer^3^. Due to asymptomatic early stages and limited proper screening for precancerous lesions, many OC cases remain undetected until the advanced stages (III/IV)^4^. Currently, a combination of platinum-based and taxane-based chemotherapy is the standard for systemic OC treatment^5^. Despite many initial responses to chemotherapy, cases almost invariably relapse^6^. To date, OC still lacks efficient therapeutic targets, and a better understanding of the mechanisms driving OC is needed.

Abnormal regulation of cell-signal-transduction pathways play a key role in cancer. Among them, the insulin-like growth factor 1 receptor/insulin receptor substrate 1 (IGF1R/IRS1) signaling pathway contributes to the transformation and growth of malignant cells, and enhances the migration and invasiveness of tumor cells in several types of cancers, including OC^7–10^. Among the dysregulated downstream signaling of the IGF1R/IRS1 pathway, the PI3K/AKT/mTOR cascade has been identified as frequently altered in OC, mediating key mechanisms underlying its growth and progression^11,12^. Thus, this pathway is considered as one of the most important signaling pathways for therapeutic intervention in OC^12,13^.

The Forkhead/winged helix family of transcription factors are key molecular players during tumorigenesis^14^. One member of which, Forkhead box protein 3 (FoxP3) was first described as a major molecular factor involved in regulatory T-cell (Treg) development and function^15,16^, and was later detected in various other cells and tissues, suggesting that the biological effects of FoxP3 are not restricted to Tregs^17–20^. Recently, FoxP3 has been identified as a tumor suppressor or oncogene in various cancer types^20–23^. In breast cancer, it has been shown to repress the expression of HER2 and SKP2^24,25^; conversely, in non-small cell lung cancer, it can act as a co-activator to facilitate the Wnt/β-catenin signaling pathway to induce tumor growth and metastasis^26^. Notably, one study found that FoxP3 expression was lower in OC than in normal ovarian tissue, and its upregulation inhibited OC progression^27^. Nevertheless, the precise molecular mechanism underlying the regulation of FoxP3 in OC remains unclear.

microRNAs (miRNAs) are small (~22 nt) noncoding RNAs that influence a wide range of biological processes by repressing target gene expression at posttranscriptional levels^28,29^. A growing body of evidence has demonstrated that aberrant miRNA expression is implicated in the pathogenesis of cancer, which suggests that miRNAs may be effective therapeutic targets^30,31^. miR-150 has been identified as an aberrantly expressed miRNA in various cancers with recent evidence suggesting that it functions as an oncogene in breast cancer, lung cancer, and OC^32–34^, as well as a tumor suppressor in liver, colorectal, breast, and non-small-cell lung cancers^35–38^. Considering the complex and contradictory nature of the molecular basis and function of miR-150, further investigation is needed to confirm its context-specific role. The purpose of the present study was to investigate the molecular function of miR-150 and its regulatory mechanisms in OC. We found that a complex FoxP3-miR-150-IGF1R/IRS1-PI3K/AKT/mTOR feedback loop was associated with OC pathogenesis, which suppressed OC cell growth and metastasis. Thus, this study highlights a novel mechanism for miR-150 as a tumor suppressor in OC.

## Methods

### Cell culture and drug treatment

The cell lines SKOV3, ES2, and HEK293T were obtained from ATCC (Manassas, VA, USA). The A2780 cell line was acquired from ECACC (Salisbury, UK). A2780 and HEK293T cells were cultured in Dulbecco’s modified Eagle’s medium (DMEM, Biological Industries (BI), Israel), ES2 cells were cultured in McCoy’s 5A medium (BI), and SKOV3 cells were cultured in Roswell Park Memorial Institute (RPMI) 1640 medium (BI). All media were supplemented with 10% fetal bovine serum (Gibco, Grand Island, NY, USA) and cells were cultured at 37 °C in a 5% CO_2_ incubator. All cell lines were authenticated by genetic profiling using polymorphic short tandem repeat loci. The cells were treated with LY294002 (Beyotime, Shanghai, China) or rapamycin (Beyotime) at the indicated concentrations for 24 h, before subsequent analysis.

### Microarray data analysis

Microarray datasets were obtained from Gene Expression Omnibus (GEO): GSE71477 (eight groups including 19 paired tissue samples representing OC and endometrium), GSE106817 (including 29 serum samples of benign ovarian disease and 320 serum samples of OC), GSE61485 (including five tissue samples of normal fallopian tube fimbria and five of high-grade serous OC). miRNAs downregulated by <0.8-fold or upregulated by >1.25-fold (*P* < 0.025) in the OC samples were considered to be dysregulated.

### Establishment of stable OC cell lines

To generate stable mir-150-overexpressing cells, A2780, SKOV3, and ES2 cells were transduced with a lentivirus-expressing mir-150 or a negative control (Genechem, Shanghai, China), and selected using 0.5 μg/mL puromycin. After 1 week, puromycin-resistant cell pools with green fluorescent protein signals were identified using laser scanning confocal microscopy, collected, and then verified by real-time RT-PCR. To generate stable FoxP3-overexpressing, IRS1-overexpressing, mir-150 and IRS1 co-expressing, or mir-150 and IGF1R/IRS1 co-expressing cells, the lentiviral particles were first produced in HEK293T cells transfected with p-FoxP3 (EX-T8364-Lv105, GeneCopoeia, USA), p-IRS1 (EX-G0328-Lv105, GeneCopoeia), p-IGF1R (EX-L0202-Lv152-IGF1R, GeneCopoeia), or control plasmid (EX-NEG-Lv105, EX-L0202-Lv152, GeneCopoeia) along with psPAX2 (Addgene, Watertown, MA, USA) and pMD2.G (Addgene) plasmids. OC cells were then infected with lentiviral particles and selected using 0.5 μg/mL puromycin or 200 μg/mL hygromycin. After 1 week, puromycin or hygromycin-resistant cell pools were collected and verified by western blotting.

### miRNA, siRNA, and transfection

miRNAs (miR-150-5p mimics, miR-150-3p mimics, and negative control) were obtained from RiboBio (Guangzhou, China) and referred to as miR-150-5p, miR-150-3p and miR-NC, respectively. siRNAs were obtained from GenePharma (Shanghai, China). The sequences of siRNAs are listed in Supplementary Table S1. miRNAs and siRNAs were transfected at a final concentration of 50 nM and 30 nM respectively, using Lipofectamine 6000 Reagent (Beyotime). The cells were then collected 48 h after transfection for subsequent analysis.

### Real-time RT-PCR

Total RNA was extracted using TRIzol (Thermo Fisher Scientific, Waltham, MA, USA). To quantify mRNA levels, cDNA was synthesized using Hiscript II Q RT SuperMix for qPCR (+gDNA wiper) (Vazyme Biotech, Nanjing, China), and PCR was conducted using ChamQ Universal SYBR qPCR Master Mix (Vazyme). GAPDH mRNA levels served as a reference control. The sequences of the qPCR primers are listed in Supplementary Table S1. miRNA expression was evaluated as described previously^39^, using the bulge-loop miR-150-5p/3p qPCR primer set (RiboBio), with human small nuclear RNA U6 as an internal control.

### Western blot analysis

Western blot analysis was performed as described previously^40^. The following primary antibodies were used: anti-cyclin D1 (60186-lg, Proteintech, Chicago, IL, USA), anti-p27 (ab193379, Abcam, Cambridge, UK), anti-p21 (#2947, Cell Signaling Technology, Danvers, MA, USA), anti-phosphorylated Rb (Ser795) (#9301, Cell Signaling Technology), anti-IRS1 (#2382, Cell Signaling Technology), anti-phosphorylated IRS1 (Ser307) (D151214, BBI, Shanghai, China), anti-IGF1R (20254-1-AP, Proteintech), anti-phosphorylated AKT (Ser473) (66444-1-lg, Proteintech), anti-AKT (10176-2-AP, Proteintech), anti-phosphorylated mTOR (Ser2448) (D155324, BBI), anti-mTOR (20657-1-AP, Proteintech), anti-FoxP3 (D260367, BBI), anti-Dicer (ab14601, Abcam), anti-Drosha (55001-1-AP, Proteintech), and anti-GAPDH (#2118, Cell Signaling Technology).

### Immunohistochemistry

Immunohistochemistry analysis was performed as described previously^40^, using a super-sensitive horseradish peroxidase immunohistochemistry kit (Sangon Biotech, Shanghai, China); the anti-Ki67 (sc-23900, Santa Cruz), anti-IGF1R (D163034, BBI), and anti-IRS1 (D120888, BBI) antibodies were used.

### Dual-luciferase reporter assay

DNA fragments containing the miR-150-5p/3p binding sites in the CDS or 3ʹ-UTR region of five candidate genes (IRS1, PSPH, LRIG2, ARMC9, and IGF1R) were amplified by PCR primers, and cloned into the pGL3-control vector (Promega, Madison, WI, USA) at the XbaI site using an In-Fusion HD Cloning Plus kit (TaKaRa, Dalian, China). To construct the mir-150 promoter reporter plasmids (P1, P2, P3, and P4), four genomic fragments in the 2-kb region upstream of the mir-150 gene were inserted into the XhoI sites of the pGL6-TA vector (Beyotime). For reporter assays, firefly luciferase reporter vectors and *Renilla* luciferase control vector (pRL-CMV) were co-transfected into HEK293T cells with miR-150-5p/3p mimics or p-FoxP3. Luciferase activity was measured 24 h after transfection using the Dual-Glo Luciferase Assay System (Promega). Primer sequences used for PCR amplification of plasmid construction are listed in Supplementary Table S1.

### Isobaric tags for relative and absolute quantitation (iTRAQ) proteomic analysis

To identify the differentially expressed proteins between Lv-mir-150 and Lv-mir-NC-A2780 cells, iTRAQ combined with nanoscale liquid chromatography coupled with tandem mass spectrometry (nano LC-MS/MS) analysis was performed as described previously^41^. Briefly, cells were harvested and re-suspended in cell SDT lysis buffer (4% SDS, 100 mM Tris-HCl, 1 mM DTT, pH 7.6), before filter-aided sample preparation digestion was performed. A 100 μg peptide mixture of each sample was labeled using iTRAQ reagent (115 and 116 for Lv-mir-150-A2780, 113 and 114 for Lv-mir-NC-A2780; Applied Biosystems), which was fractionated by SCX chromatography using the AKTA Purifier system (GE Healthcare). After nano LC-MS/MS analysis of each fraction, protein identification and iTRAQ quantitation were performed using Mascot 2.5 and Proteome Discoverer 2.1 (Thermo Fisher Scientific, USA).

### Animal studies

All animal studies were reviewed and approved by the Institutional Ethics Committee of Wenzhou Medical University and performed as described previously^39^. Briefly, 6-week-old female BALB/c nude mice were purchased from Shanghai Laboratory Animal Center (Shanghai, China) and housed under pathogen-free conditions. For the xenograft studies, a total of 2.5 × 10^6^ Lv-mir-150-ES2 or control cells in 100 µL phosphate-buffered saline (PBS) were subcutaneously (s.c.) injected into the bilateral rear flank of the mice. Tumors were measured using a Vernier caliper, and tumor volume was calculated using the following equation: *V* = *L* × *W*^2^ × 0.5236 (*L* = long axis, *W* = short axis). After 22 days, the mice were sacrificed, and the tumors were harvested and weighed. For in vivo metastasis assays, 3 × 10^6^ Lv-mir-150-A2780 or Lv-mir-150-ES2 cells, and the corresponding control cells, in 100 µL PBS were injected intraperitoneally (i.p.) into the nude mice. After 45 days (for A2780) or 24 days (for ES2), the mice were photographed, and then euthanized. The nodules throughout the peritoneal cavity were counted and the ascites weight was measured. The intestines were subjected to fluorescent image detection using the IVIS Lumina III In Vivo Imaging System (PerkinElmer, MA, USA).

### The Cancer Genome Atlas (TCGA) data set analysis

TCGA gene expression data were obtained from TCGA Data Portal (March 2015 release; http://cancergenome.nih.gov/). mRNA expression was determined by next generation sequencing using the HiSeq 2000 platform. Reads per million (RPM) was used to quantify mir-150 expression levels from the miRNA-Seq datasets of 483 OC tissues. mRNA expression was calculated as reads per kilobase per million mapped reads (RPKM) values in 265 OC tissues. The normalized values of mir-150 and other genes’ expression were converted to log_10_-transformed values for subsequent analysis.

### Colony formation and 5-ethynyl-2′-deoxyuridine (EdU) proliferation assays

For the colony formation assay, treated cells were seeded in six-well plates at a density of 1000 cells per well and cultured for 8–10 days. The colonies were then fixed with cold methanol and stained with 0.1% crystal violet; colonies comprising more than 50 cells were counted. For the EdU incorporation assay, cell proliferation was determined as described previously^39^, using the Cell-Light™ EdU Apollo®643 In Vitro Imaging kit (RiboBio).

### Cell cycle and apoptosis analysis

Treated cells were harvested at 80% confluence and washed twice with ice-cold PBS. Cell cycle and apoptosis analysis were performed as described previously^39^.

### In vitro migration and invasion, and wound healing assays

The migration and invasion assays were conducted as we have described previously^42^; 4.5 × 10^4^ cells were used for migration (SKOV3, ES2 for 6 h and A2780 for 12 h) and invasion (SKOV3, ES2 for 12 h). Wound healing assay was performed as described previously^39^, and the scratch healing ability was recorded by acquiring images 0, 24, 48, and 72 h after scratching.

### Statistical analysis

Data are presented as the mean ± s.d. Unless noted otherwise, each experiment was carried out in triplicate. Statistical significance was determined by two-tailed Student’s *t*-test or Mann–Whitney *U*-test. Differences between groups were determined using two-way analysis of variance (ANOVA). The correlation between the expression of different genes in the same sample was determined using Pearson’s correlation analysis. For all statistical tests, *P* values < 0.05 were considered to be statistically significant.

## Results

### miR-150-5p and miR-150-3p are downregulated in OC

**Fig. 1:**
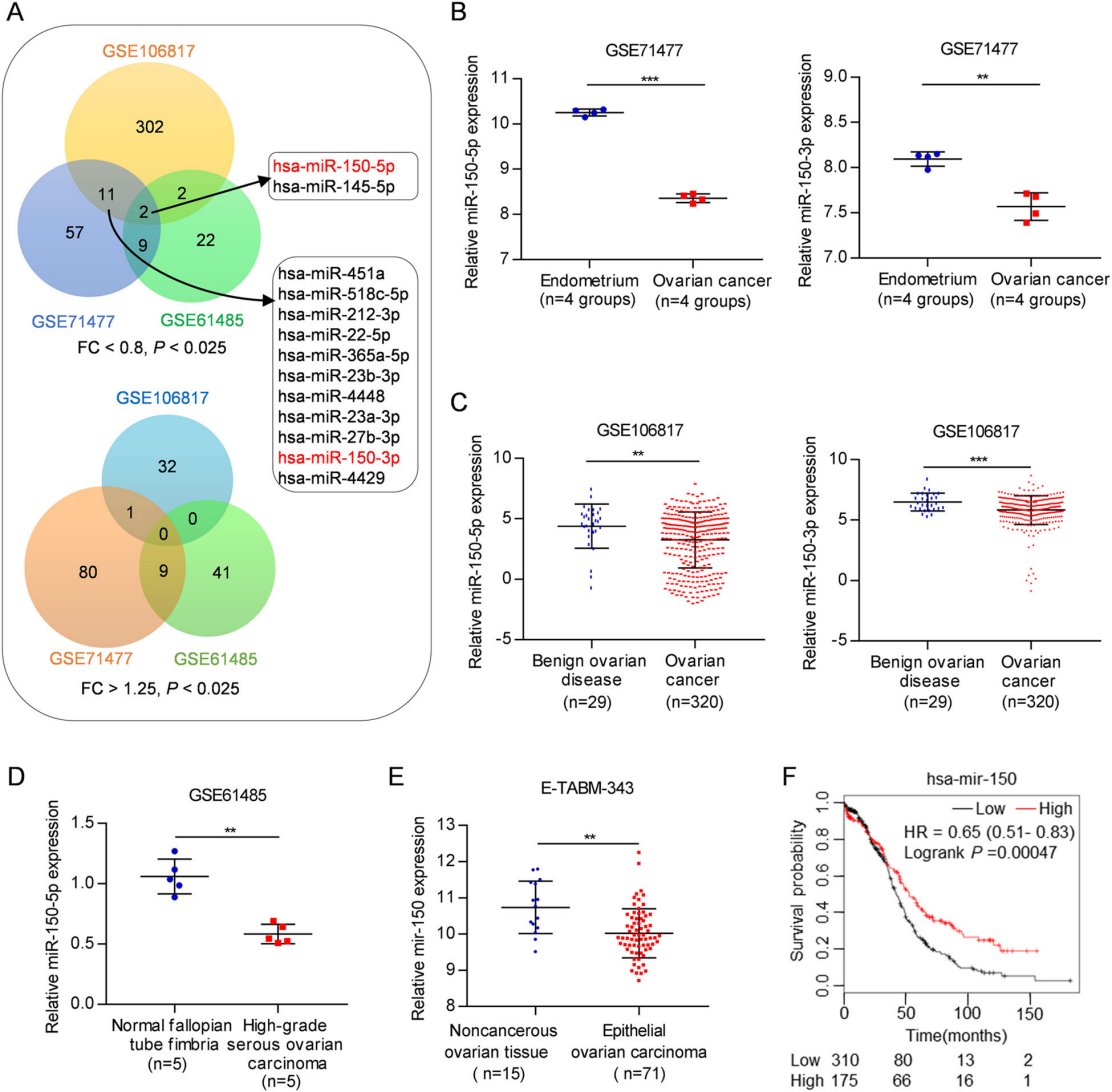
miR-150-5p/3p are downregulated in OC. **A** Dysregulated miRNAs in OC compared with the corresponding control in GSE71477, GSE106817, and GSE61485 datasets (FC <0.8 or >1.25, *P* < 0.025). **B**–**D** Expression of miR-150-5p and miR-150-3p was significantly downregulated in OC compared with those in the endometrium (GSE71477) (**B**), benign ovarian disease (GSE106817) (**C**), or normal fallopian tube fimbria (GSE61485) (**D**). **E** The levels of miR-150 precursor (mir-150) were also reduced in OC compared with noncancerous ovarian tissues (E-TABM-343). **F** Kaplan–Meier survival curves for OC patients according to mir-150 expression levels in tumor tissues; significance was calculated using log-rank test. The data are presented as the mean ± s.d. **P < 0.01, ***P < 0.001 by Wilcoxon signed-rank test or Mann–Whitney *U*-test.

To identify key miRNAs required for OC development, we comprehensively analyzed three GEO datasets obtained from: 1) paired OC and endometriosis tissues (GSE71477), 2) patient serum of OC and benign ovarian disease (GSE106817)^43^, and 3) high-grade serous OC and normal fallopian tube fimbria tissues (GSE61485)^44^. We found that a total of 34 (24 downregulated and 10 upregulated) miRNAs were significantly commonly dysregulated in at least two datasets (fold-change (FC) <0.8 or >1.25, *P* < 0.025) (Fig. 1A and Supplementary Table S2). Apart from miR-145-5p, only miR-150-5p, one of the top dysregulated miRNAs, was downregulated in OC compared with the corresponding control in these three datasets. Accordingly, miR-150-3p was reduced in the GSE71477 and GSE106817 datasets, despite no available expression value in GSE61485 (Fig. 1A–D and Supplementary Table S2). Similarly, the levels of miR-150 precursor (mir-150) were also reduced in OC compared with noncancerous ovarian tissues (E-TABM-343) (Fig. 1E). Furthermore, the association of mir-150 expression and patient survival in pan-cancer was evaluated using an online Kaplan–Meier plotter tool^45^. Kaplan–Meier survival curves and log-rank tests showed that lower expression levels of mir-150 were significantly correlated with poor patient outcomes in 9 out of 21 types of cancers, including OC (Fig. 1F and Supplementary Fig. S1). These data demonstrate that miR-150 may play a tumor-suppressive role in the development and progression of OC.

### miR-150 inhibits growth of OC cells and promotes apoptosis in vitro and in vivo

**Fig. 2:**
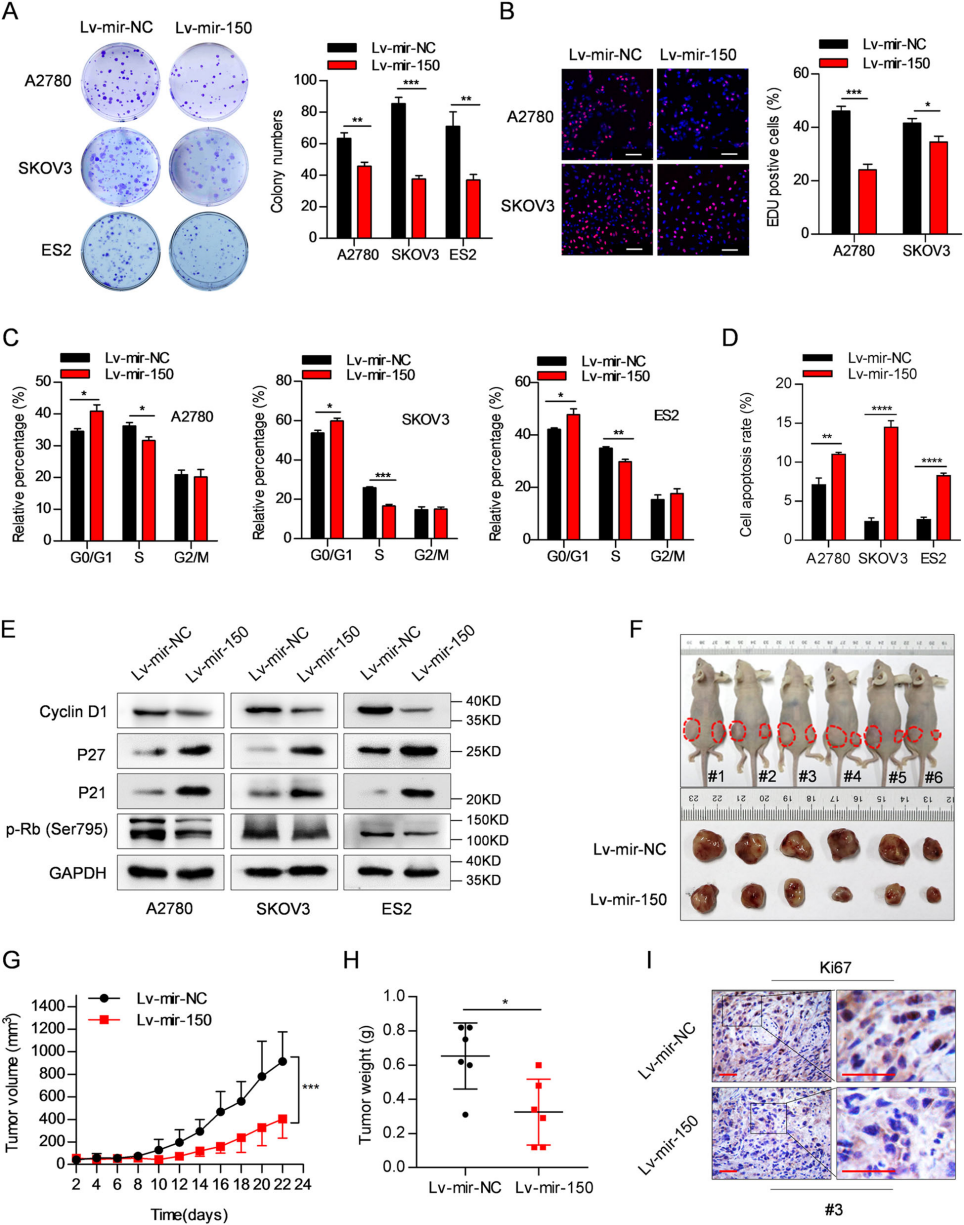
miR-150 inhibits growth of OC cells and promotes apoptosis in vitro and in vivo. A2780, SKOV3, and ES2 cells were transduced with mir-150-overexpressing lentivirus (Lv-mir-150) or control lentivirus (Lv-mir-NC). **A, B** Cell proliferation was determined by colony formation assay (**A**) and EdU incorporation assay (**B**). Scale bar: 100 µm. **C** Cell cycle was determined by flow cytometry. **D** Cell apoptotic rate was determined by flow cytometry of cells with Annexin V-PE/7AA-D double staining. **E** Levels of Cyclin D1, p27, p21, and p-Rb (Ser795) were detected in Lv-mir-150 cells by western blotting. **F** ES2 cells stably transfected with Lv-mir-150 (right) or Lv-mir-NC (left) were injected subcutaneously into female BALB/c nude mice (*n* = 6), and images of the tumors at autopsy from nude mice were presented (bottom). **G** Tumor volumes were measured at the indicated time points. **H** Average weight of xenografted tumors was measured. **I** Representative photographs of immunohistochemical (IHC) staining of Ki67 in xenografted tumors from Lv-mir-150-ES2 cells or control cells. Magnification: ×400. Scale bar: 50 µm. The data are presented as the mean ± s.d. *P < 0.05, **P < 0.01, ***P < 0.001, ****P < 0.0001 by Student’s *t*-test or two-way ANOVA.

To further investigate the biological role of miR-150 in OC, stable OC cell lines (A2780, SKOV3, and ES2) were generated by infecting cells with either mir-150-overexpressing lentivirus (Lv-mir-150) or control lentivirus (Lv-mir-NC). Large increases in both miR-150-5p and miR-150-3p levels were observed in Lv-mir-150-OC cells compared with those of the corresponding Lv-mir-NC group (Supplementary Fig. S2). Overexpression of miR-150 inhibited proliferation of OC cells in the colony formation assay (Fig. 2A). In line with this, EdU incorporation assays showed that DNA replication in Lv-mir-150-OC cells was significantly reduced (Fig. 2B). Cell cycle analysis revealed that miR-150 overexpression significantly arrested OC cells at the G1-S transition, as indicated by a marked accumulation of cells in the G1 peak and a reduction of cells in S phase (Fig. 2C and Supplementary Fig. S3a). In addition, overexpression of miR-150 markedly promoted apoptosis of OC cells (Fig. 2D and Supplementary Fig. S3b). We also observed that miR-150 overexpression significantly regulated the expression of cell cycle-related proteins including decreased CyclinD1 and p-Rb (Ser795), and increased p27 and p21 (Fig. 2E). To determine whether miR-150 affects tumor formation in vivo, Lv-mir-150-ES2 cells were subcutaneously injected into BALB/c nude mice that were euthanized 22 days after inoculation. As expected, the average tumor volumes and weights were significantly reduced in Lv-mir-150-ES2 groups compared with those in Lv-mir-NC groups (Fig. 2F–H). Furthermore, Ki-67 (a cell proliferation marker) staining revealed that tumors from Lv-mir-150-ES2 groups had fewer proliferative cells than those in the control group (Fig. 2I). Collectively, these results indicate that miR-150 can exert a significant inhibitory effect on OC growth in vitro and in vivo.

### miR-150 inhibits migratory and invasive abilities of OC cells in vitro and in vivo

**Fig. 3:**
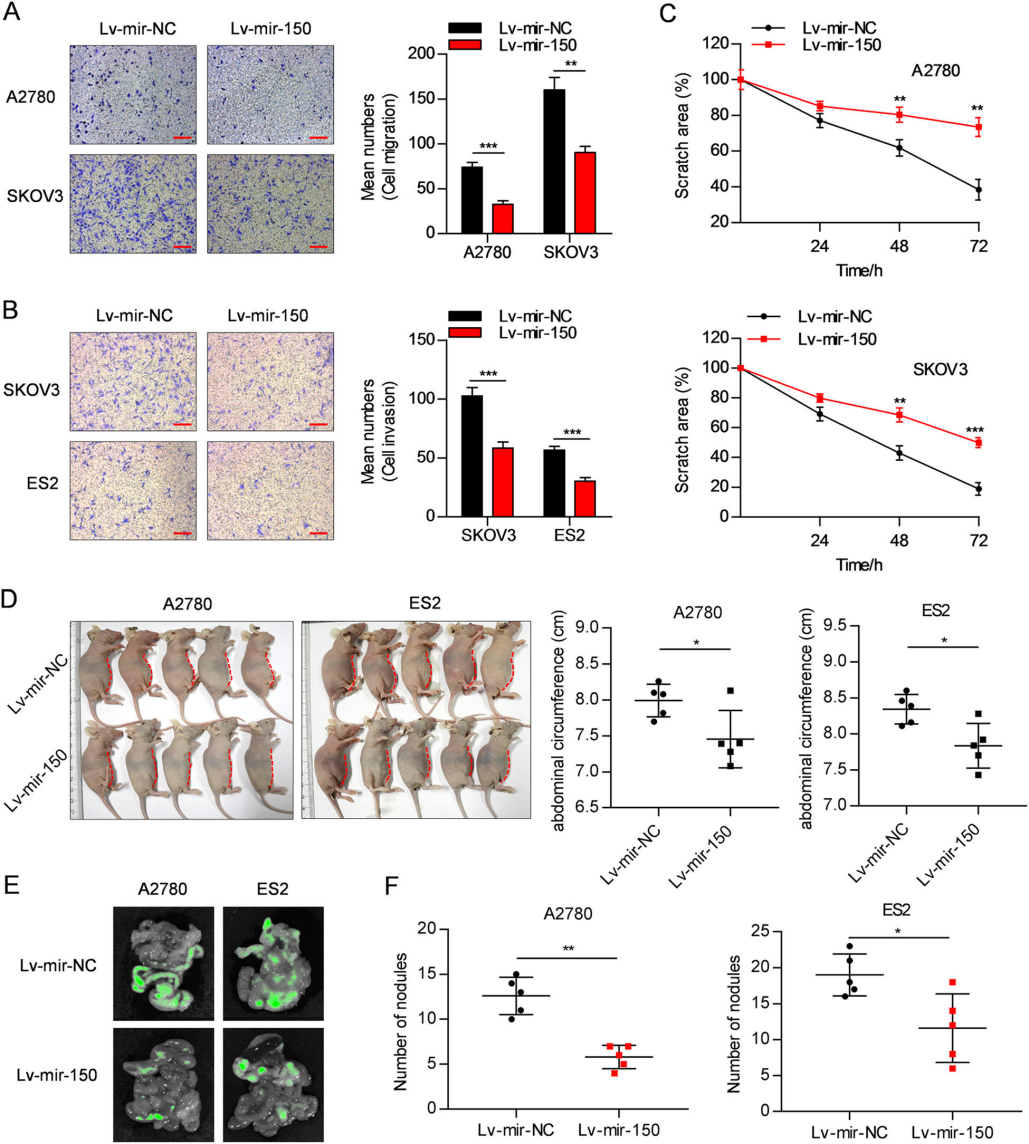
miR-150 inhibits migratory and invasive ability of OC cells in vitro and in vivo. **A**, **B** Stably overexpressing miR-150 reduced migration (**A**) and invasion (**B**) of OC cells in vitro. Scale bar: 200 µm. **C** Wound healing assay indicated that miR-150 overexpression inhibited healing ability of A2780 (top) and SKOV3 (bottom) cells. **D–F** Lv-mir-150 OC cells and corresponding Lv-mir-NC cells were intraperitoneally injected into female BALB/c nude mice that were euthanized after 45 days (for A2780 cells) or 24 days (for ES2 cells) (*n* = 5). **D** Images showing smaller ascites volumes (left) and abdominal circumference (right) in mice injected with Lv-mir-150 cells than those in control cells. **E** Fluorescence imaging revealed that mice injected with Lv-mir-150 cells had weaker green fluorescence signals on the mesentery. **F** Number of nodules was significantly lower in mice injected with Lv-mir-150 cells when compared with that of the control group. The data are presented as the mean ± s.d. *P < 0.05, **P < 0.01, ***P < 0.001 by Student’s *t*-test.

To examine the effect of miR-150 on OC cell migration and invasion, Lv-mir-150-OC cells and the corresponding control cells were cultured in Transwell chambers pre-coated with or without Matrigel. As shown in Fig. 3A, B, Lv-mir-150 OC cells showed significantly decreased migratory and invasive abilities compared with that of Lv-mir-NC cells. Consistent with this, in vitro wound healing assays showed that overexpression of miR-150 inhibited cell migration and impeded closure of a scratched area of cells (Fig. 3C and Supplementary Fig. S3c). To investigate the effect of miR-150 on OC metastasis in vivo, Lv-mir-150-A2780, Lv-mir-150-ES2, and their corresponding control cells were intraperitoneally injected into nude mice that were sacrificed after 45 and 24 days, respectively. Notably, the mice that had been injected with Lv-mir-150 cells showed smaller ascites volumes along with abdominal circumference (Fig. 3D), although no significant difference in ascites weight was observed between the two groups. Fluorescence imaging revealed that the mice inoculated with Lv-mir-150 cells had less green fluorescence signals on their mesentery than did mice injected with Lv-mir-NC cells (Fig. 3E). Furthermore, a few small nodules throughout the peritoneal cavity were also detected in the Lv-mir-150 group (Fig. 3F). Although the inhibitory effects of miR-150 observed in the in vivo metastasis assay may be partially due to decreased cell proliferation, these results indicate that miR-150 also inhibits the migratory and invasive abilities of OC cells.

### IRS1 and IGF1R are direct targets of miR-150-5p/3p

**Fig. 4:**
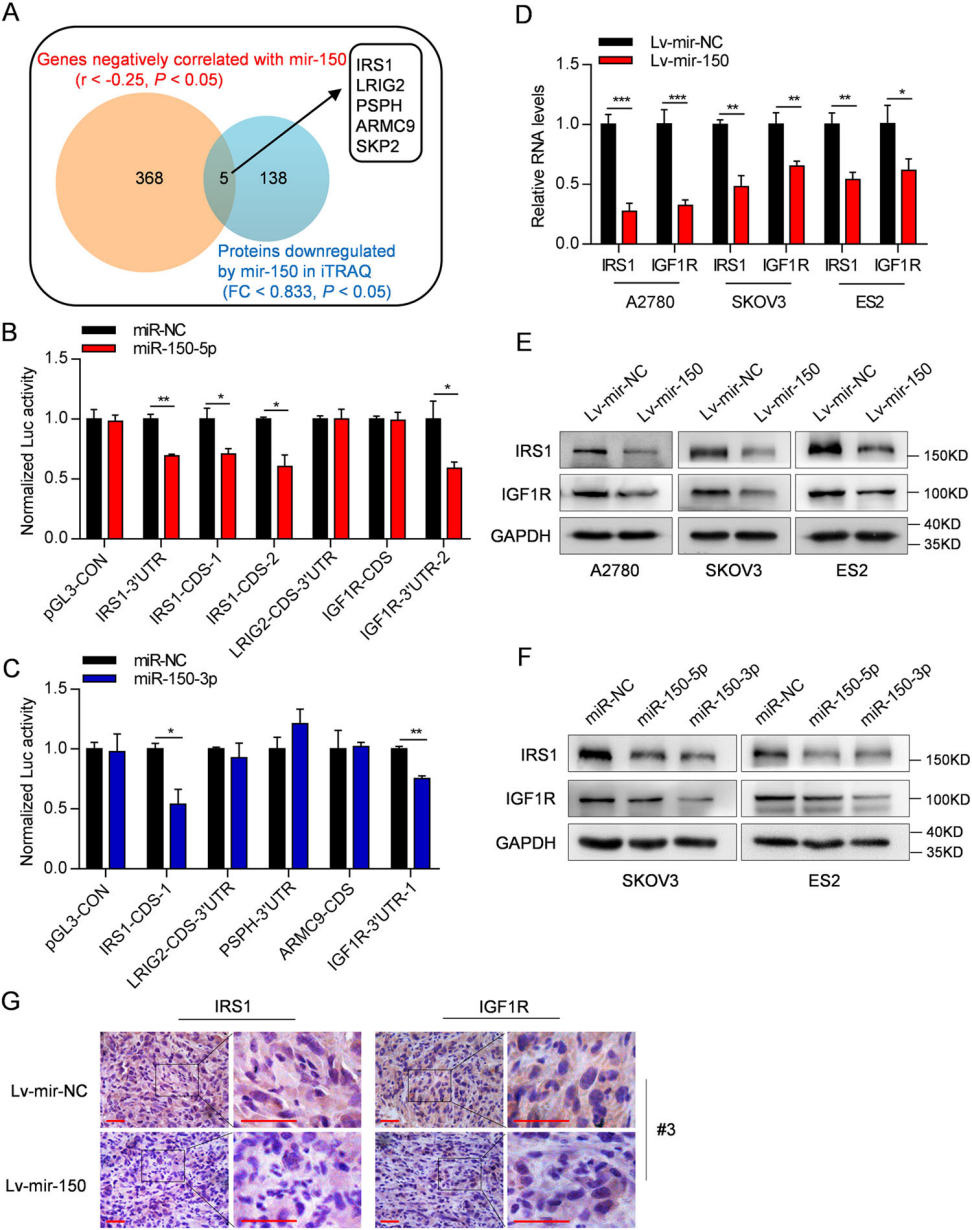
IRS1 and IGF1R are directly targets of miR-150-5p/3p. **A** Venn diagram depicting the number of the proteins significantly downregulated (blue) (FC < 0.833, *P* < 0.05) in Lv-mir-150-A2780 cells shown in iTRAQ analysis and the number of protein-coding genes negatively correlated with mir-150 (orange) (*r* < −0.25, *P* < 0.05) in 265 OC tissues from TCGA. **B**, **C** Luciferase activities were measured in HEK293T cells transfected with reporter plasmids containing the predicted binding sites, together with miR-150-5p (**B**), miR-150-3p (**C**) or control mimic. **D**, **E** Relative mRNA (**D**) and protein (**E**) levels of IRS1 and IGF1R in Lv-mir-150-OC cells were measured by real-time RT-PCR and western blot analyses. **F** The protein levels of IRS1 and IGF1R in SKOV3 and ES2 cells transfected with miR-150-5p or miR-150-3p mimic. **G** IHC staining showed that the expression of IRS1 and IGF1R was reduced in xenografted tumors from Lv-mir-150-ES2 cells. Magnification: ×400. Scale bar: 50 µm. The data are presented as the mean ± s.d. *P < 0.05, **P < 0.01, ***P < 0.001 by Student’s *t*-test.

To probe into the molecular mechanism by which miR-150 exerts tumor inhibitory effects on OC, protein extracted from the Lv-mir-150 and Lv-mir-NC-A2780 cells were firstly analyzed using an iTRAQ approach (Supplementary Fig. S4a). In total, 5970 proteins were identified from 41,274 distinct peptides (FDR < 0.01; Supplementary Table S3). Among them, 167 downregulated and 77 upregulated proteins (143 and 64 with gene symbol, respectively; FC <0.833 or >1.2, *P* < 0.05) were identified in Lv-mir-150-A2780 cells (Supplementary Fig. S4b and Supplementary Table S3). Second, we investigated the relationship between mir-150 and all protein-coding genes in 265 OC tissues from TCGA and found that the levels of mir-150 were negatively correlated with 373 protein-coding genes (*r* < −0.25, *P* < 0.05) (Supplementary Table S4). Interestingly, five overlapping genes were identified, which included IRS1, LRIG2, PSPH, ARMC9, and SKP2 (Fig. 4A and Supplementary Fig. S5). Using miRWalk bioinformatics algorithms, we found that, except for SKP2, the remaining genes had predicted miR-150-5p or miR-150-3p binding sites in their CDS or 3ʹ-UTR region (Supplementary Tables S5 and S6). Notably, we also found a significant negative correlation between mir-150 and IGF1R. IGF1R is an upstream gene of IRS1, harboring predicted miR-150-5p/3p binding sites in its CDS or 3ʹ-UTR region, although its protein levels were not shown in our iTRAQ data (Supplementary Fig. S5 and Tables S5 and S6).

To validate specific regulation through these predicted binding sites, we constructed nine reporter vectors (IRS1-CDS-1, IRS1-CDS-2, IRS1-3ʹUTR, PSPH-3ʹUTR, LRIG2-CDS-3ʹUTR, ARMC9-CDS, IGF1R-CDS, IGF1R-3ʹUTR-1, and IGF1R-3ʹUTR-2) consisting of the luciferase coding sequence followed by the region containing the predicted binding sites, respectively, as shown in Supplementary Fig. S6. Co-transfection experiments showed that miR-150-5p reduced the luciferase activities of IRS1-3ʹUTR, IRS1-CDS-1, IRS1-CDS-2, and IGF1R-3ʹUTR-2 (Fig. 4B), and miR-150-3p reduced the luciferase activities of IRS1-CDS-1 and IGF1R-3ʹUTR-1 in HEK293T cells (Fig. 4C), indicating that IRS1 and IGF1R are potential targets of miR-150-5p/3p. Furthermore, endogenous mRNA and protein levels of IRS1 and IGF1R were decreased in Lv-mir-150-OC cells compared with those in the corresponding control cells (Fig. 4D, E). Similarly, transient transfection with miR-150-5p or miR-150-3p mimic significantly downregulated the expression of IRS1 and IGF1R in OC cells (Fig. 4F). Consistent with this, the protein levels of IRS1 and IGF1R were also decreased in Lv-mir-150 tumor samples from nude mice (Fig. 2F), as shown by immunohistochemistry analysis (Fig. 4G). More interestingly, the highest association scores between miR-150-5p/3p and IRS1 or IGF1R were found in 481 ovarian serous cystadenocarcinoma tissues through pan-cancer analysis by CancerMiner (Supplementary Fig. S7). Collectively, these results indicate that IRS1 and IGF1R are novel targets of miR-150-5p/3p in OC cells.

### The miR-150-IGF1R/IRS1 axis exerts antitumor effects via the PI3K/AKT/mTOR pathway

**Fig. 5:**
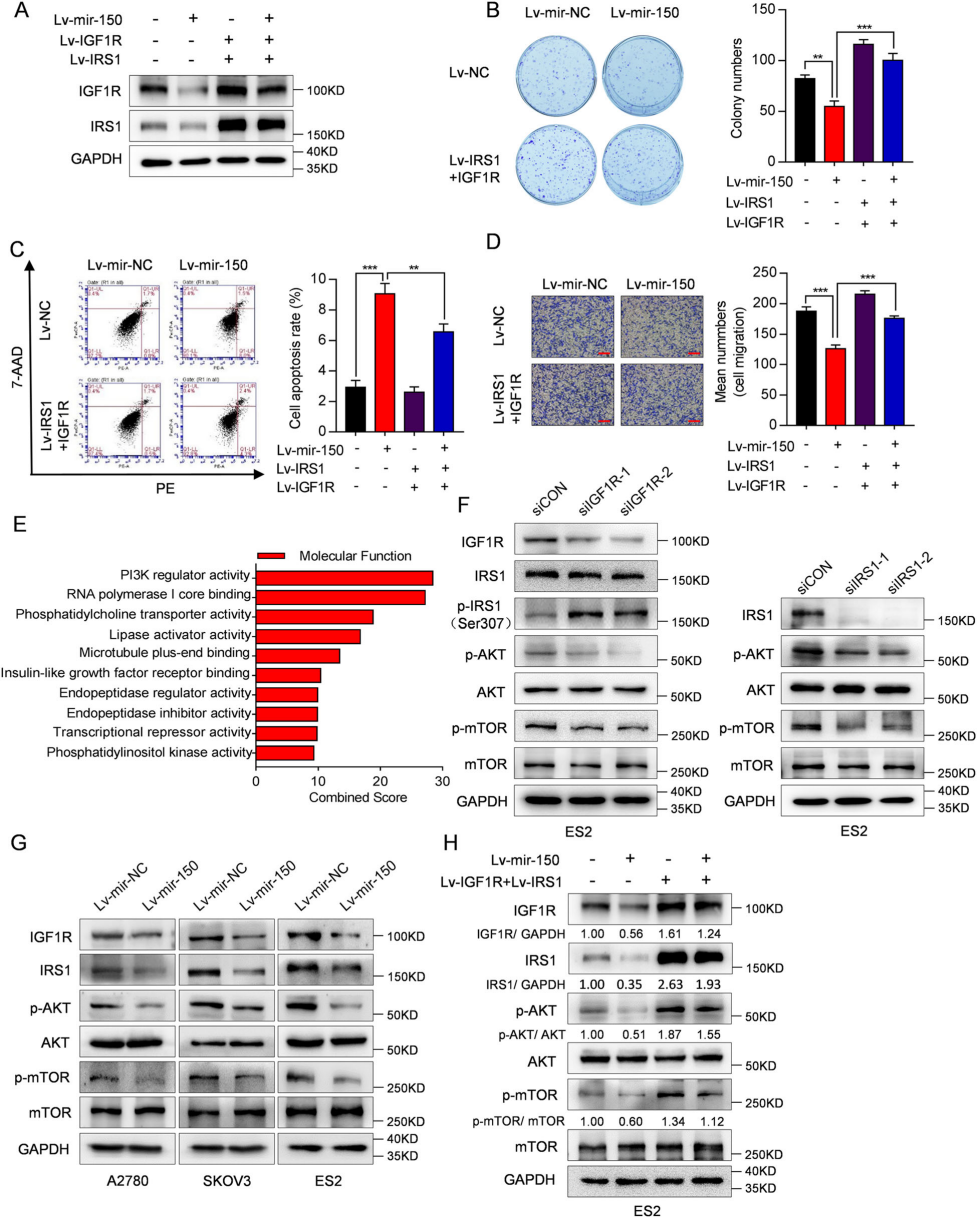
miR-150-IGF1R/IRS1 regulates PI3K/AKT/mTOR signaling in OC cells. **A**–**D** ES2 cells stably co-expressing miR-150, IGF1R and IRS1 were established. **A** The protein levels of IGF1R and IRS1 determined by western blotting. Forced IGF1R and IRS1 expression restored the effects of miR-150 on cell proliferation (**B**), cell apoptosis (**C**), and cell migration (**D**) in ES2 cells. Scale bar: 200 µm. **E** Functional annotation clustering of proteins dysregulated by miR-150 in A2780 cells was shown. The ten most enriched groups according to GO molecular function analysis are ranked based on combined score. **F** The protein levels of IGF1R, IRS1, phosphorylated IRS1 (Ser307), phosphorylated AKT (Ser473), total AKT, phosphorylated mTOR (Ser2448), and total mTOR in ES2 cells transfected with siIGF1R or siIRS1 were determined by western blotting. **G** Effects of overexpression of miR-150 on protein levels of IGF1R, IRS1, phosphorylated AKT (Ser473), total AKT, phosphorylated mTOR (Ser2448), and total mTOR in OC cells. **H** Forced IGF1R and IRS1 expression partially restored the levels of phosphorylated AKT (Ser473) and phosphorylated mTOR (Ser2448) reduced by miR-150. The data are presented as the mean ± s.d. **P < 0.01, ***P < 0.001, by Student’s *t*-test.

To investigate the role of IRS1 and IGF1R in OC development, siRNAs targeting IRS1 and IGF1R were utilized to suppress their expression, and knockdown efficiency was determined by both real-time RT-PCR and western blot analyses (Supplementary Fig. S8a, b). As expected, knockdown of IRS1 or IGF1R blocked cell proliferation, as assessed by colony formation assay, promoted cell apoptosis, and inhibited cell migration and invasion in OC cells (Supplementary Fig. S8c–g). These data indicate that suppression of IRS1 and IGF1R have similar effects as miR-150 on OC tumorigenesis. To determine whether miR-150-5p/3p-exerted antitumor effects are mediated by IGF1R and IRS1, we considered that IRS1 is downstream of IGF1R and firstly established ES2 cells that stably co-express miR-150 and IRS1 (Supplementary Fig. S9a). As expected, forced IRS1 expression partially restored the effects of miR-150 (Supplementary Fig. S9b–d). Furthermore, joint forced IGF1R and IRS1 expression completely restored miR-150-inhibited cell proliferation and migration, and more significantly decreased miR-150-induced cell apoptosis (Fig. 5A–D).

To generate comprehensive insights into the molecular mechanisms underlying the miR-150-IGF1R/IRS1 axis, we analyzed the dysregulated proteins in Lv-mir-150-A2780 cells from iTRAQ data. Gene ontology (GO) analysis^46^ revealed that a molecular function related to phosphatidylinositol 3-kinase (PI3K) regulator activity was within the top enriched proteins dysregulated by miR-150 overexpression (Fig. 5e). Notably, the activation of the PI3K/AKT/mTOR pathway occurs in many types of cancer, and is one of the key downstream transduction signaling pathways of the IGF1R/IRS1 interaction^7,47^. Therefore, we first determined whether IGF1R/IRS1 is also involved in PI3K/AKT/mTOR signaling in OC. Indeed, we found that knockdown of IGF1R increased the level of phosphorylated IRS1 (Ser307) (p-IRS1(Ser307)), and decreased the levels of phosphorylated AKT (Ser473) (p-AKT) and phosphorylated mTOR (Ser2448) (p-mTOR). Similar effects were observed following IRS1 knockdown (Fig. 5F). In line with this, overexpression of miR-150 inhibited the expression of IGF1R and IRS1 and reduced the levels of p-AKT and p-mTOR (Fig. 5G), which could be restored by ectopic IRS1 expression (Supplementary Fig. S9e) or joint ectopic IGF1R and IRS1 expression (Fig. 5H). Collectively, these results show that miR-150-5p/3p suppresses the PI3K/AKT/mTOR signaling pathway by targeting IGF1R and IRS1 in OC cells.

### The miR-150-IGF1R/IRS1 axis as a downstream target of FoxP3

**Fig. 6:**
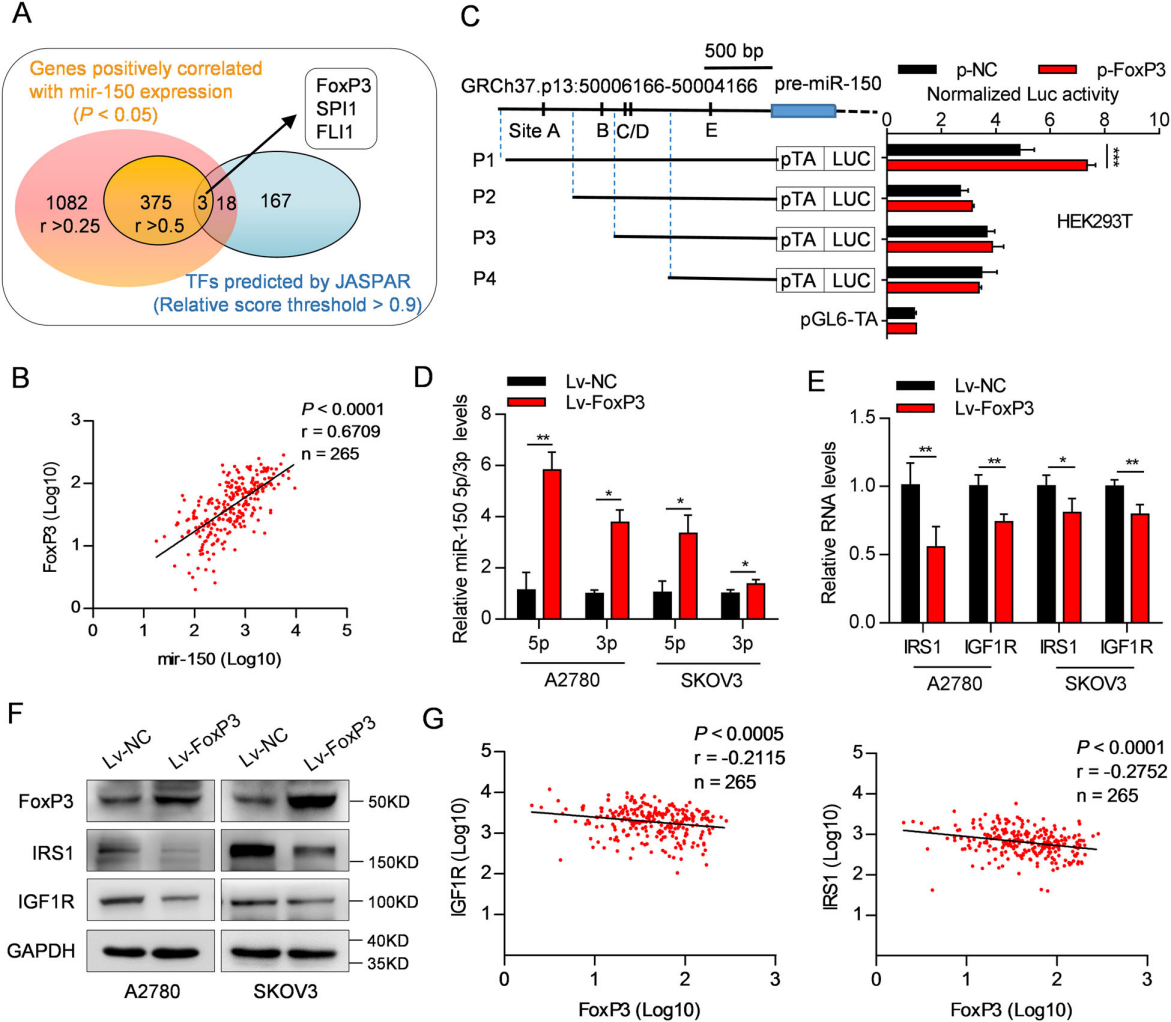
miR-150-IGF1R/IRS1 axis is a downstream target of FoxP3. **A** Venn diagram depicting the number of predicted transcription factors (blue) in 2-kb region upstream of miR-150 using the JASPAR database (relative score threshold >0.9) and the number of protein-coding genes positively correlated with miR-150 (orange) (*r* > 0.25 or >0.5, *P* < 0.05) in 265 OC tissues from TCGA. **B** Correlation between FoxP3 and mir-150 expression in 265 OC tissues from TCGA. **C** Diagram of the 2-kb region upstream of mir-150 promoter. The predicted FoxP3-binding sites (named A, B, C, D and E), and the DNA fragments incorporated into luciferase reporter constructs (named P1–P4) were indicated. Luciferase reporter assays with the corresponding reporter constructs in HEK293T cells were shown. **D** The relative levels of miR-150-5p and miR-150-3p in Lv-FoxP3-OC cells were determined by real-time RT-PCR. **E**, **F** Relative mRNA (**E**) and protein (**F**) levels of IRS1 and IGF1R in Lv-FoxP3-OC cells were measured by real-time RT-PCR and western blotting. **G** Correlation between FoxP3 and IRS1 or IGF1R expression in 265 OC tissues from TCGA. The data are presented as the mean ± s.d. *P < 0.05, **P < 0.01, ***P < 0.001 by Student’s *t*-test.

To elucidate the mechanism underlying the downregulation of miR-150-5p/3p in OC cells, we analyzed the 2-kb region upstream of mir-150 for the presence of various transcription factor binding motifs using the JASPAR database^48^. Subsequently, we predicted a total of 188 transcription factors, among which FoxP3 was one of the genes most significantly correlated with mir-150 (*r* = 0.67, *P* < 0.0001; Fig. 6A, B and Supplementary Table S4). By JASPAR database analysis, five FoxP3-binding sites (named A, B, C, D and E; relative score threshold >0.9) were identified inside the putative mir-150 promoter region; thus, DNA fragments including different binding sites were used to construct luciferase reporter plasmids, named P1–P4 (Fig. 6C). Co-transfection experiments revealed only the induction of P1 luciferase activity upon FoxP3 overexpression (Fig. 6C), suggesting that binding site A was the functional FoxP3 binding site. Furthermore, ectopic FoxP3 expression significantly increased the levels of miR-150-5p/3p and their primary transcript (pri-miR-150) without affecting the Drosha and Dicer expression (Fig. 6D and Supplementary Fig. S10a–c). Accordingly, the mRNA and protein levels of IRS1 and IGF1R were decreased in Lv-FoxP3-OC cells compared with those in the corresponding control cells (Fig. 6E, F). Notably, there was a significant negative correlation between FoxP3 and IRS1/IGF1R (Fig. 6G). Together, these data suggest that FoxP3 positively regulates mir-150 expression and in turn, inhibits the expression of IRS1 or IGF1R.

### The PI3K/AKT/mTOR pathway regulates FoxP3 and miR-150-5p/3p expression

**Fig. 7:**
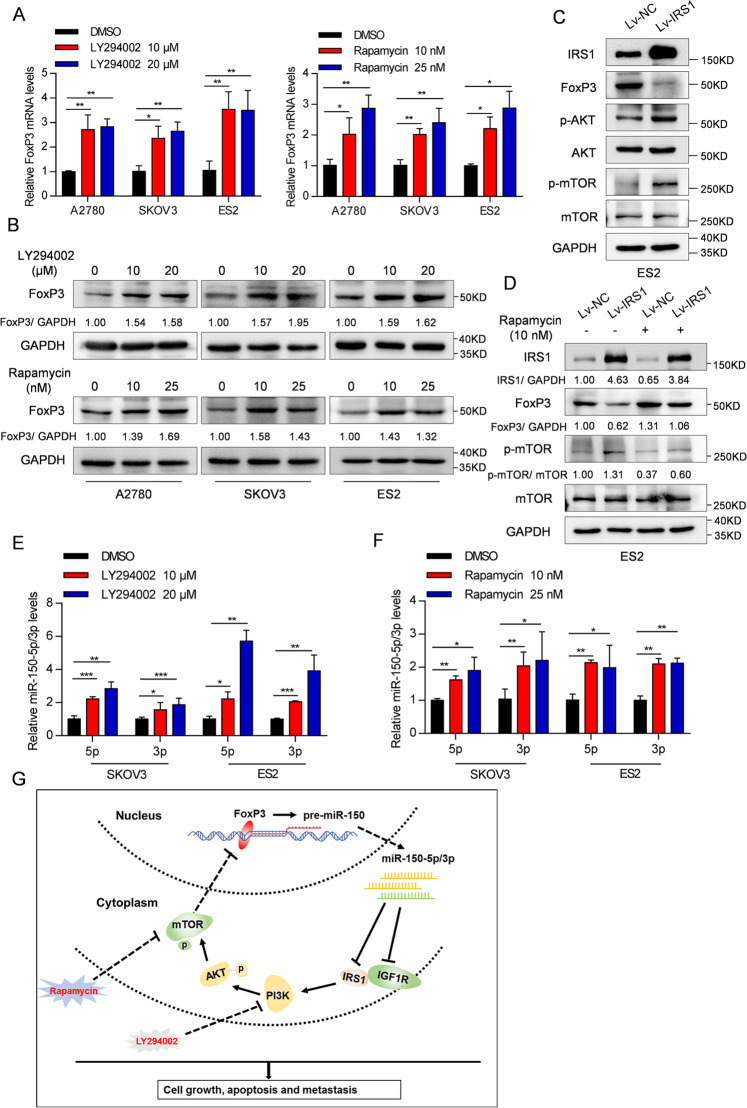
The PI3K/AKT/mTOR pathway regulates the expression of FoxP3 and miR-150-5p/3p. **A**, **B** OC cells were treated with PI3K inhibitor (LY294002) or mTOR inhibitor (rapamycin) for 24 h. The mRNA (**A**) and protein (**B**) levels of FoxP3 were measured by real-time RT-PCR and western blot analyses, respectively. **C** The protein levels of IRS1, phosphorylated AKT (Ser473), total AKT, phosphorylated mTOR (Ser2448), total mTOR, and FoxP3 in Lv-IRS1-ES2 cells were determined by western blotting. **D** The protein expression levels of IRS1, FoxP3, phosphorylated mTOR (Ser2448), and total mTOR were measured in Lv-IRS1-ES2 cells treated with 10 nM rapamycin for 24 h. **E**, **F** The relative levels of miR-150-5p and miR-150-3p in SKOV3 and ES2 cells treated with LY294002 (**E**) or rapamycin (**F**). The data are presented as the mean ± s.d. *P < 0.05, **P < 0.01, ***P < 0.001 by Student’s *t*-test. **G** Schematic diagram of a feedback loop formed from the FoxP3-miR-150-IGF1R/IRS1 axis and PI3K/AKT/mTOR signaling pathway, and its function in OC tumorigenesis.

Previous studies have shown that the PI3K/AKT/mTOR signaling network blocks FoxP3 expression in T cells^49,50^. Additionally, we found that miR-150 is a downstream target of FoxP3. This observation led us to hypothesize that the activation of PI3K/AKT/mTOR inhibits the expression of FoxP3, which then downregulates miR-150 in OC cells. As expected, we found that the mRNA and protein levels of FoxP3 were significantly enhanced in OC cells when treated with either the PI3K inhibitor (LY294002) or mTOR inhibitor (rapamycin) (Fig. 7A, B). Conversely, ectopic IRS1 expression increased the levels of p-AKT and p-mTOR and reduced the expression of FoxP3 in ES2 cells (Fig. 7C), which was then reversed by the mTOR inhibitor (Fig. 7D). These results indicate that the PI3K/AKT/mTOR pathway inhibits FoxP3 in OC cells.

Lastly, consistent with the FoxP3 induction, the expression of miR-150-5p and miR-150-3p was also increased in OC cells treated with the PI3K or mTOR inhibitors (Fig. 7E, F), suggesting that a feedback loop was created from the FoxP3-miR-150-IGF1R/IRS1 and PI3K/AKT/mTOR signaling pathways.

## Discussion

In this study, we found that the expression of miR-150-5p/3p and their precursor, mir-150, was downregulated in OC tissues compared with those in normal endometrium or ovarian tissues. Furthermore, we demonstrated that ectopic mir-150 expression significantly decreased cell proliferation, migration, and invasion, but promoted cell apoptosis in vitro. In addition, mir-150 inhibited tumor growth and metastasis in vivo. These findings provide strong evidence to support a tumor-suppressive role of mir-150 in OC cells, potentially attributable to the miR-150-5p and miR-150-3p overexpression.

miR-150-5p has been shown to control B cell differentiation^51^, regulate the development of NK and iNKT cells^52^, and enhance targeted endothelial cell migration^53^. Subsequent studies have reported both tumor-promoting and tumor-suppressive effects of miR-150-5p and miR-150-3p in several types of cancer. Specifically, miR-150-5p promotes cell proliferation and migration by targeting the P2X7 receptor in breast cancer cells^34^, by targeting FOXO4^33^ and SRC kinase signaling inhibitor 1^54^ in lung cancer cells, and by the c-Myb/Slug signaling cascade in OC cells^32^. In contrast, miR-150-5p inhibits CD133-positive liver cancer stem cells^35^, functions as a tumor suppressor in colorectal cancer cells^36^, and suppresses triple-negative breast cancer metastasis^37^. In addition, miR-150-3p suppresses the growth of glioma cells by targeting SP1^55^, and induces apoptosis in pancreatic cancer cells^56^. In the present study, we demonstrated that the overexpression of both miR-150-5p and miR-150-3p inhibits OC growth and metastasis in vitro and in vivo, which combined with previous reports^57,58^ strongly supports the tumor-suppressive effects of miR-150-5p/3p in OC. Although there are conflicting reports regarding miR-150 function in the same cancer type, the varied roles of miR-150 across different cancer types may result from the different miR-150-targeting genes and their diverse effects.

To our knowledge, OC does not exhibit distinct early symptoms, and a lack of effective early detection biomarkers is one cause of the high case mortality^4^. That plasma miR-150-5p and miR-150-3p levels were lower in OC patients than in control subjects, together with the finding that lower levels of mir-150 were associated with poor OC patient outcomes, strongly support that miR-150-5p/3p is dysregulated during OC progression and development. Whether miR-150-5p/3p can be used as candidate biomarkers of OC requires further validation in a larger sample cohort. However, it is not clear which factors trigger dysregulation in OC. Here, our study showed that miR-150-5p/3p is a downstream target of FoxP3, which has a dual role as both transcriptional activator and repressor. In Treg cells, it has been proposed that FoxP3 is able to bind to more than 2800 transcription factor binding sites (TFBS) of over 700 genes^59^. Moreover, thousands of other genes are indirectly regulated by FoxP3, suggesting that others factors, such as FoxP3 interacting partners or its targeting-miRNAs, may be involved in the regulation mechanisms^14,60,61^. In OC, we first reported that the expression of miR-150-5p/3p was obviously induced by FoxP3. Combined with the inhibitory effects of mir-150 on OC tumorigenesis, we surmise that miR-150-5p/3p may be a key factor in FoxP3-mediated tumor suppression in OC^27^. However, the increased frequency of tumor-infiltrating Tregs has been shown to be associated with poor survival in OC^62^. These contradictory reports support the notion that different mechanisms underlie the involvement of FoxP3 in OC, which are not yet fully understood.

The IGF1R/IRS1 signaling pathway plays an important role in the development, progression, and chemotherapeutic response of OC^7^. Ample preclinical evidence demonstrates the therapeutic relevance of IGF1R-targeted strategies in OC^63–65^. However, recent clinical trial results have proved disappointing, with IGF1R-targeted approaches activating the insulin receptor to promote tumor growth^66,67^. A previous study showed the negative regulation between miR-150-3p and IGF1R in pancreatic cancer cells^56^. Interestingly, in this study, we identified IRS1 and IGF1R as direct targets of both miR-150-5p and miR-150-3p, and silencing IRS1 or IGF1R mimicked the effects of mir-150 on OC cell growth, apoptosis, and metastasis. Overexpression of miR-150 markedly suppressed both IGFIR and IRS1 protein expressions in OC cells. These data imply that targeting IGF1R antagonists and IGF1R downstream factor-based inhibitors (e.g., miR-150) will have more effective therapeutic potential and may be a better choice.

Following the IGF1R/IRS1 pathway, PI3K/AKT/mTOR signaling is crucial to the malignant transformation of human tumors and their subsequent growth, proliferation, and metastasis^12,13^. Preclinical investigations have suggested that the PI3K/AKT/mTOR pathway is frequently activated in OC^12,13^. In our study, miR-150 directly targeted IGF1R and IRS1 before inhibiting PI3K/AKT/mTOR signaling, which was evidenced by the reduction of p-AKT and p-mTOR levels. This suppressed OC cell growth and metastasis while promoting apoptosis, which could be restored by ectopic IGF1R and IRS1 expression. We also identified FoxP3 as a downstream target of PI3K/AKT/mTOR signaling, which negatively regulated the expression of FoxP3 and miR-150-5p/3p in OC cells. Although the detailed mechanisms behind PI3K/AKT/mTOR inhibiting FoxP3 in OC remain unclear, previous studies have provided several possible explanations. For instance, FoxO factors (e.g., FoxO1 and FoxO3a), drivers of FoxP3 expression, can be inactivated by PI3K/AKT/mTOR signaling in Tregs^49^. Dysregulated PI3K/AKT/mTOR signaling may also contribute to the change in DNA methylation and chromatin structure in FoxP3 locus^50,68,69^, which is intimately linked to its expression.

As summarized in Fig. 7g, miR-150-5p/3p is able to suppress OC cell growth and metastasis by directly targeting both IRS1 and IGF1R and subsequently mediating downstream PI3K/AKT/mTOR signaling. The PI3K/AKT/mTOR pathway in turn downregulates miR-150-5p/3p via FoxP3. Thus, a complex FoxP3-miR-150-IGF1R/IRS1-PI3K/AKT/mTOR feedback loop is formed in OC. This model is a work-in-progress that will likely be modified in future studies; nevertheless, the present study underscores the significance of the FoxP3-miR-150-IGF1R/IRS1-PI3K/AKT/mTOR loop in mediating OC pathogenesis.

## Supplementary information

Supplementary Figure Legends

Supplementary Figure S1

Supplementary Figure S2

Supplementary Figure S3

Supplementary Figure S4

Supplementary Figure S5

Supplementary Figure S6

Supplementary Figure S7

Supplementary Figure S8

Supplementary Figure S9

Supplementary Figure S10

Supplementary Table S1-6
