# Supplementary material for: FoxP3-miR-150-5p/3p suppresses ovarian tumorigenesis via an IGF1R/IRS1 pathway feedback loop
Source: Cell Death Dis. 2021 Mar 15;12(3):275. doi: 10.1038/s41419-021-03554-6 (PMC7961150; doi:10.1038/s41419-021-03554-6)
Supplement: Supplementary file 1 — Supplementary Figure Legends [file 41419_2021_3554_MOESM1_ESM.docx]

**Supplementary Figure legends**

**Supplementary Figure S1.** Kaplan-Meier survival curves for patients with 20 types of cancer according to mir-150 expression levels in tumor tissues; significance was calculated using the log-rank test.

**Supplementary Figure S2.** Transduction efficiency was determined in A2780, SKOV3, and ES2 cells transduced with mir-150-overexpressing lentivirus (Lv-mir-150) or control lentivirus (Lv-mir-NC). **a** Representative GFP-fluorescence micrographs were shown. Scale bar: 100 μm. **b** The levels of miR-150-5p/3p were determined by real-time RT-PCR. The data are presented as the mean ± s.d. *****P* < 0.0001 by Student’s *t*-test.

**Supplementary Figure S3.** miR-150 inhibits tumor properties in OC cells. **a** Representative graphs of raw data for the cell cycle determined by flow cytometry (related to Fig. 2c). **b** Representative graphs of raw data for cell apoptosis determined by flow cytometry (related to Fig. 2d). **c** Representative graphs of raw data for wound healing assay (related to Fig. 3c). Scale bar: 200 µm.

**Supplementary Figure S4.** iTRAQ-based proteomic profiling of Lv-mir-150-A2780 cells. **a** Schematic representation of the experimental design for iTRAQ labeling showing two biological replicates for each cell line. **b** Heatmap showing the hierarchical clustering of 244 dysregulated proteins (167 downregulated and 77 upregulated) in Lv-mir-150-A2780 cells.

**Supplementary Figure S5.** Correlation between mir-150 and the expression of six genes (IRS1, LRIG2, PSPH, ARMC9, SKP2, and IGF1R) in 265 OC tissues from TCGA.

**Supplementary Figure S6.** Diagram of the putative miR-150-5p and miR-150-3p binding sites in the CDS or 3ʹ-UTR region of predicted targets by miRWalk. DNA fragments incorporated into pGL3-control luciferase reporter constructs were indicated and the named corresponding plasmids were shown.

**Supplementary Figure S7.** The association between miR-150-5p/3p and IRS1/IGF1R was evaluated through pan-cancer analysis by CancerMiner.

**Supplementary Figure S8.** Knockdown of IRS1 or IGF1R exerts antitumor effects in OC cells. SKOV3 and ES2 cells were transfected with siIRS1, siIGF1R or siCON. **a**, **b** The mRNA (a) and protein (b) levels of IRS1 and IGF1R in OC cells transfected with siIRS1 or siIGF1R were measured by real-time RT-PCR and western blot analyses. **c** Cell proliferation was determined by colony formation assay. **d** Cell apoptotic rate was increased in IRS1 or IGF1R-knockdown cells by flow cytometry of cells with Annexin V-PE/7AA-D double staining. **e, f** Silencing IRS1 or IGF1R markedly reduced the ability of migration (e) and invasion (f) in OC cells. Scale bar: 200 µm. **g** Wound healing assay indicated that silencing IRS1 or IGF1R inhibited healing ability of ES2 cells. The data are presented as the mean ± s.d. ***P* < 0.01, *** *P* <0.001 by Student’s *t*-test or two-way ANOVA.

**Supplementary Figure S9.** Forced IRS1 expression partially restored the effects of miR-150. **a–d** ES2 cells stably co-expressing miR-150 and IRS1 were established. **a** The protein levels of IRS1 determined by western blotting. Forced IRS1 expression partially restored the effects of miR-150 on cell proliferation (b), cell apoptosis (c) and cell migration (d) in ES2 cells. Scale bar: 200 µm. **e** Forced IRS1 expression partially restored the levels of phosphorylated AKT (Ser473) and phosphorylated mTOR (Ser2448) reduced by miR-150. The data are presented as the mean ± s.d. **P* < 0.05, ***P* < 0.01, ****P* < 0.001, *****P* < 0.0001 by Student’s *t*-test.

**Supplementary Figure S10.** Ectopic FoxP3 expression increased the primary transcript levels of miR-150 in OC cells. **a** Relative primary transcript levels of miR-150 (pri-miR-150) in Lv-FoxP3-OC cells were measured by real-time RT-PCR. **b** The protein levels of FoxP3, Drosha and Dicer in Lv-FoxP3-OC cells were determined by western blotting. **c** Correlation between Drosha, Dicer, and FoxP3 expression in 265 OC tissues from TCGA. The data are presented as the mean ± s.d. ***P* < 0.01 by Student’s *t*-test.

**Supplementary Table S1.** Oligonucleotides used for real-time RT-PCR, siRNA, and plasmid construction.

**Supplementary Table S2.** The expression of 34 dysregulated miRNAs in GSE71477, GSE106817, and GSE61485 datasets.

**Supplementary Table S3.** The identified proteins in Lv-mir-150-A2780 and Lv-mir-NC-A2780 cells by iTRAQ.

**Supplementary Table S4.** Correlation between mir-150 and 1851 protein-coding genes in 265 OC tissues from TCGA.

**Supplementary Table S5.** The putative miR-150-5p and miR-150-3p binding sites in the CDS or 3ʹ-UTR region of five predicted targets by miRWalk.

**Supplementary Table S6.** Comprehensive analysis of miR-150-5p/3p and five predicted targets.
