# Supplementary figures and images for: FoxP3-miR-150-5p/3p suppresses ovarian tumorigenesis via an IGF1R/IRS1 pathway feedback loop
Source: Cell Death Dis. 2021 Mar 15;12(3):275. doi: 10.1038/s41419-021-03554-6 (PMC7961150; doi:10.1038/s41419-021-03554-6)

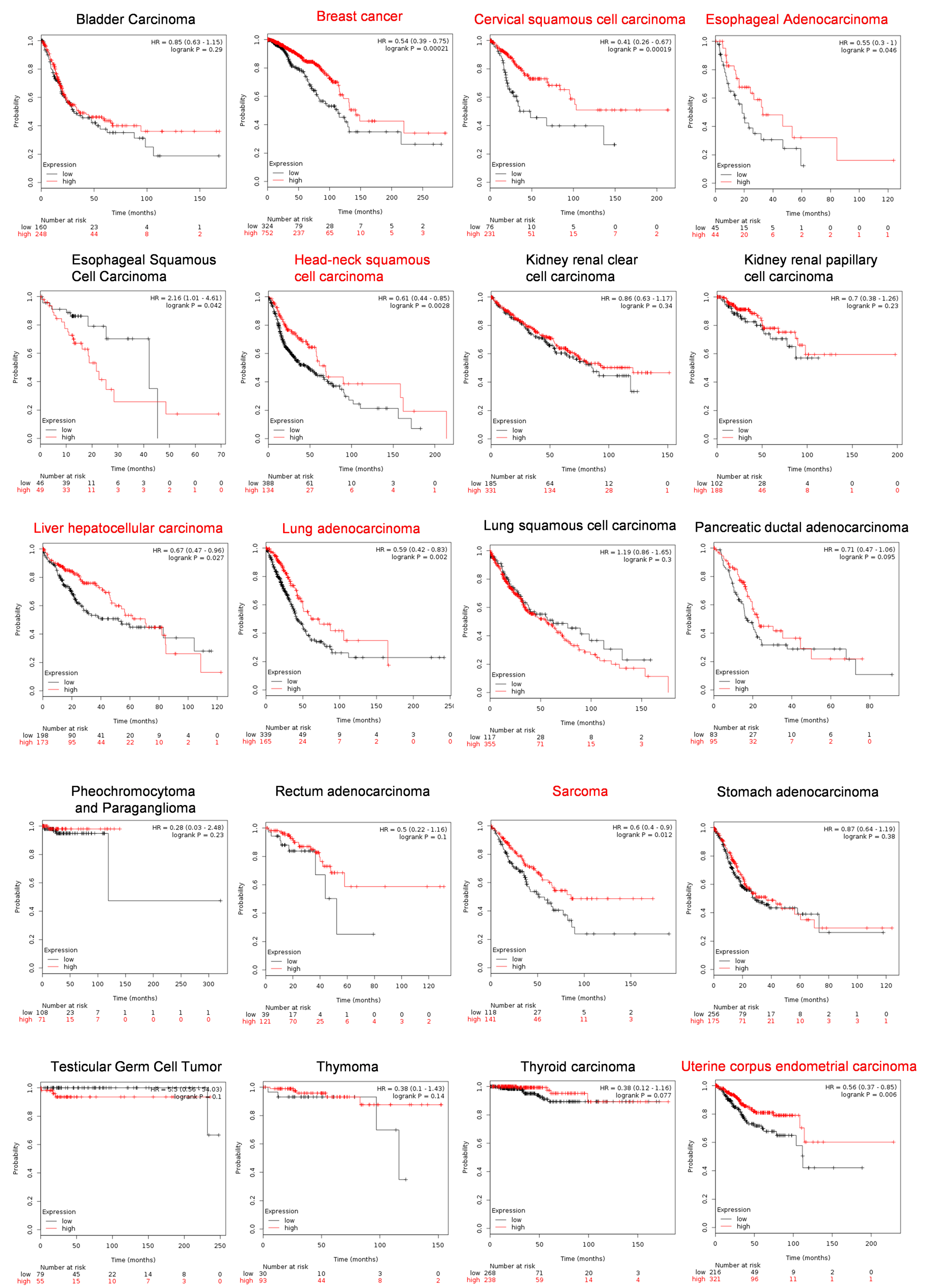

Supplement: Supplementary file 2 — Supplementary Figure S1 [file 41419_2021_3554_MOESM2_ESM.tif]

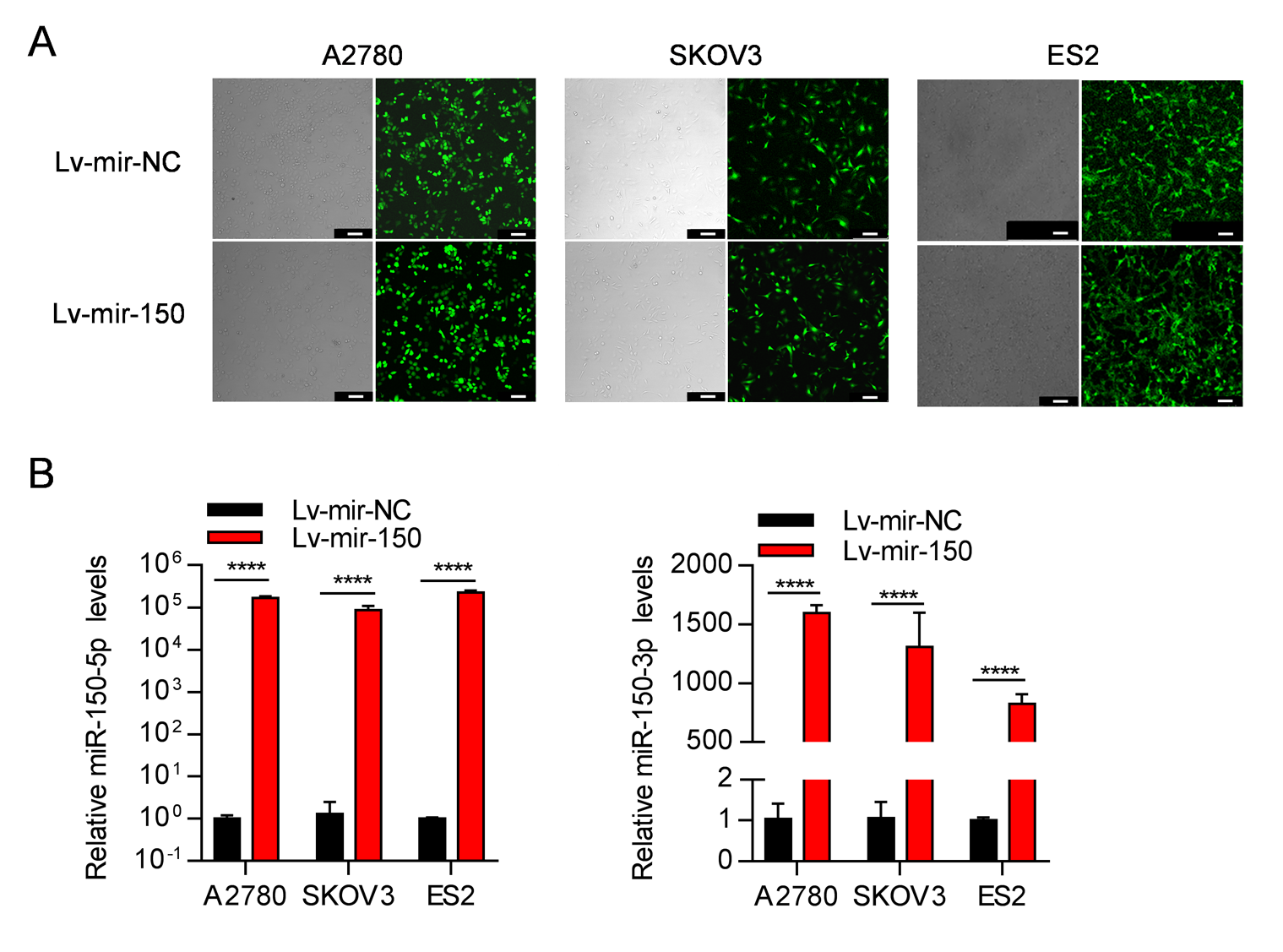

Supplement: Supplementary file 3 — Supplementary Figure S2 [file 41419_2021_3554_MOESM3_ESM.tif]

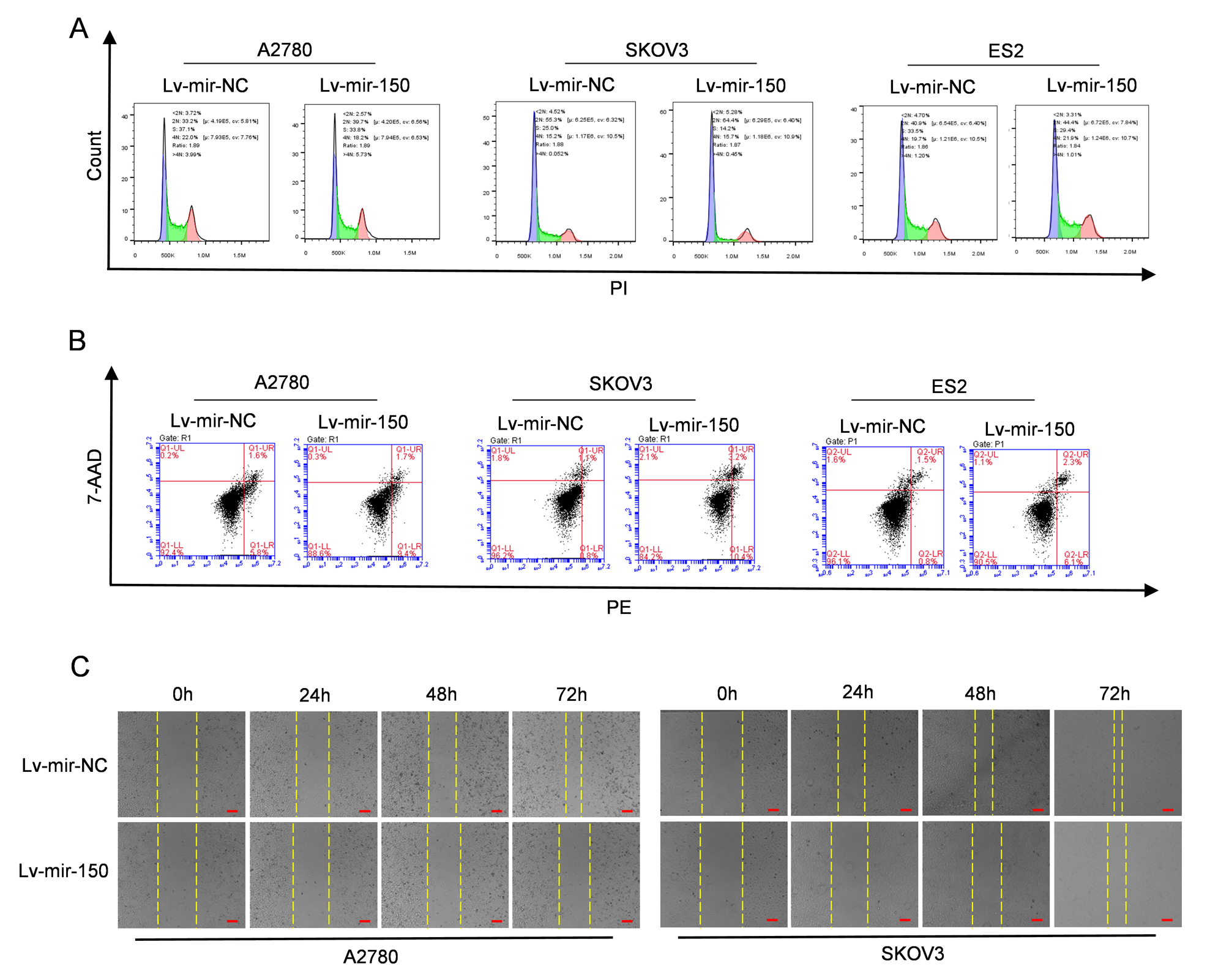

Supplement: Supplementary file 4 — Supplementary Figure S3 [file 41419_2021_3554_MOESM4_ESM.tif]

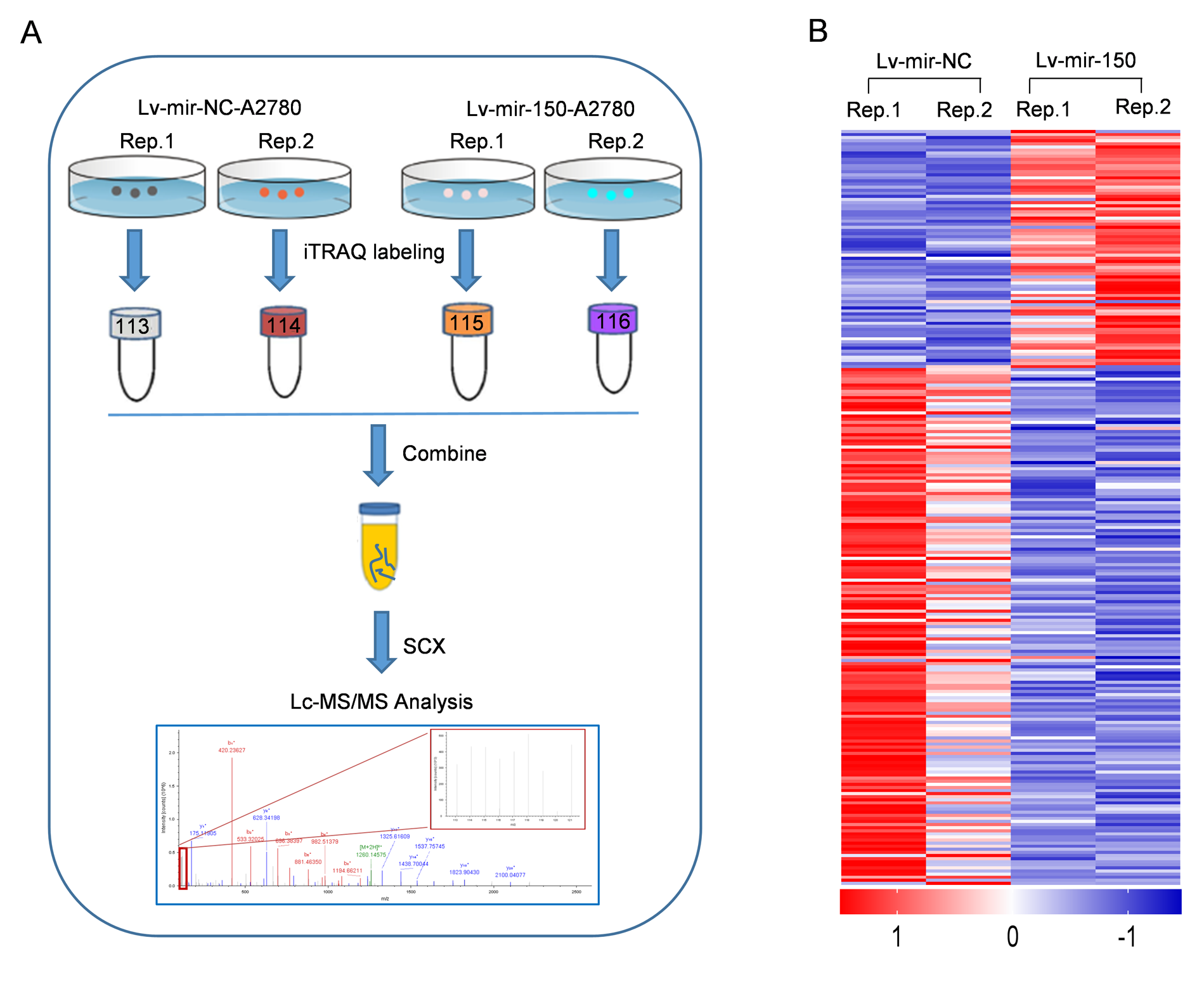

Supplement: Supplementary file 5 — Supplementary Figure S4 [file 41419_2021_3554_MOESM5_ESM.tif]

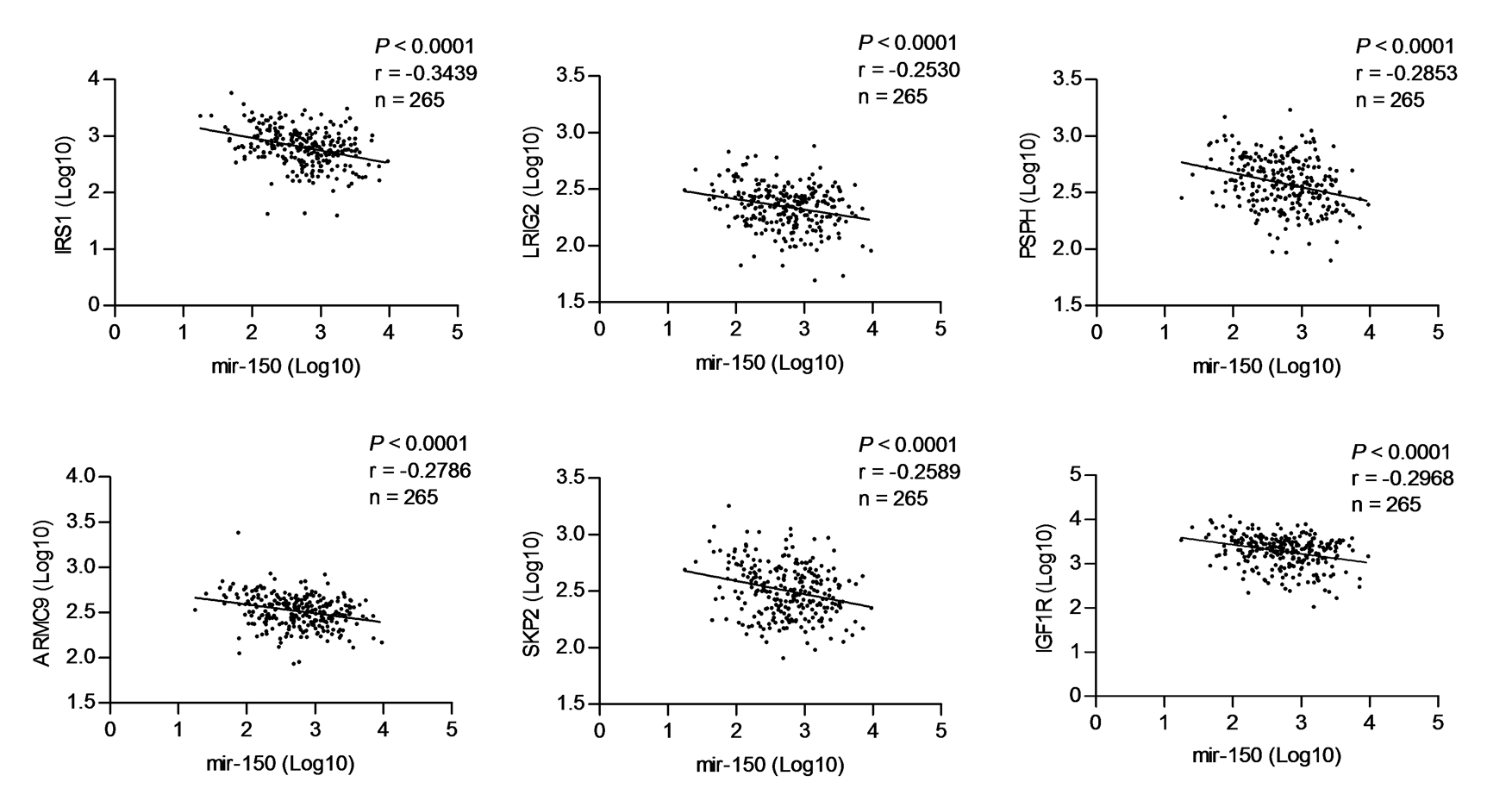

Supplement: Supplementary file 6 — Supplementary Figure S5 [file 41419_2021_3554_MOESM6_ESM.tif]

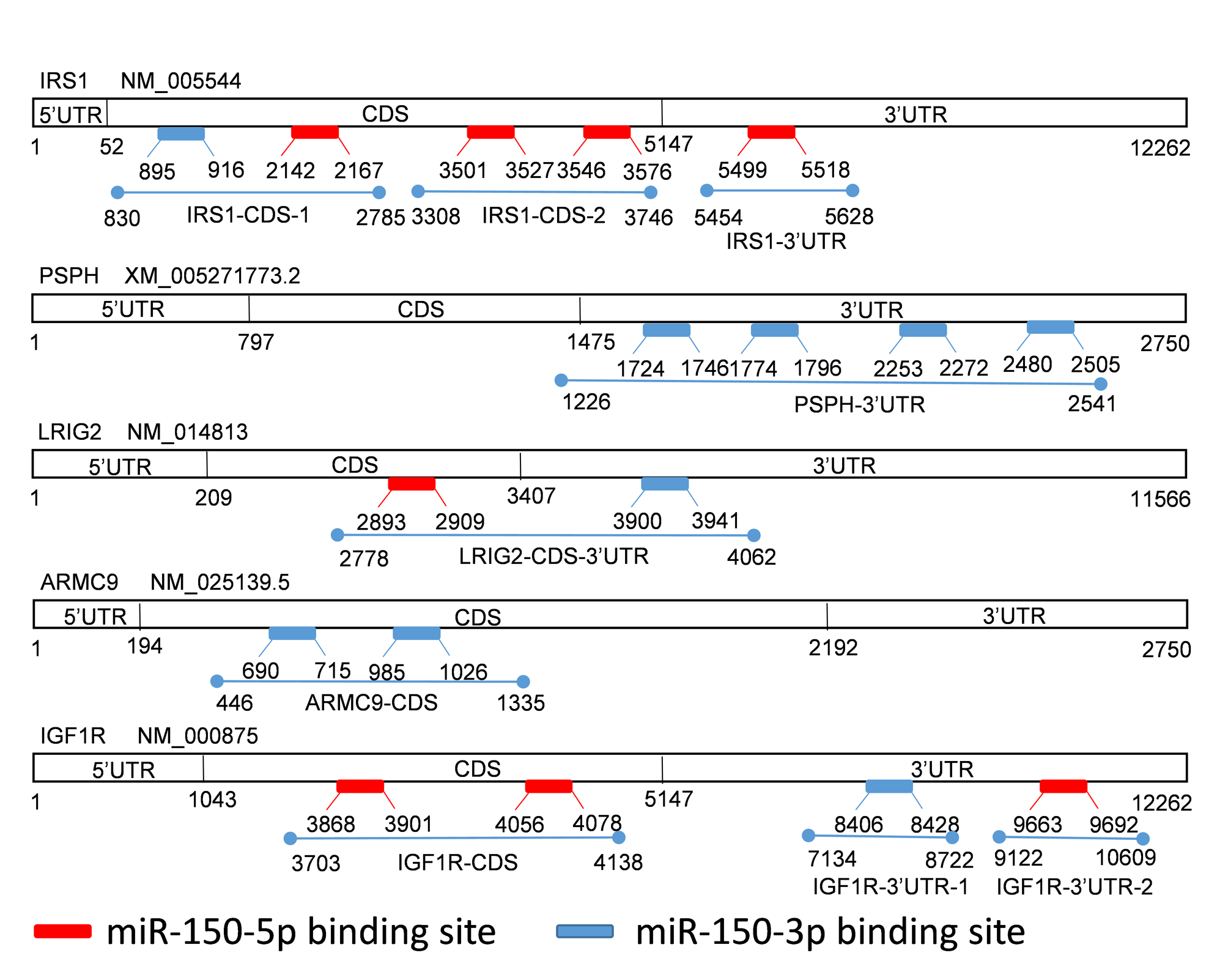

Supplement: Supplementary file 7 — Supplementary Figure S6 [file 41419_2021_3554_MOESM7_ESM.tif]

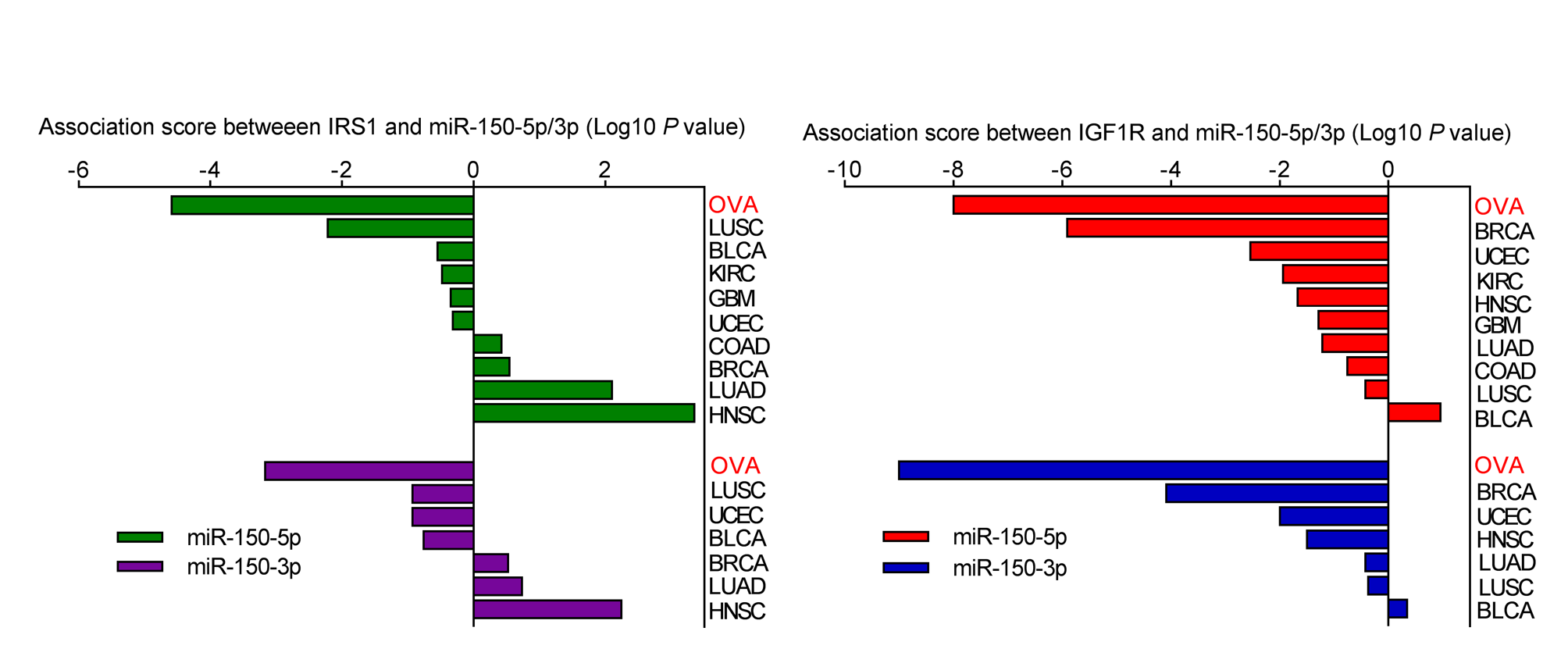

Supplement: Supplementary file 8 — Supplementary Figure S7 [file 41419_2021_3554_MOESM8_ESM.tif]

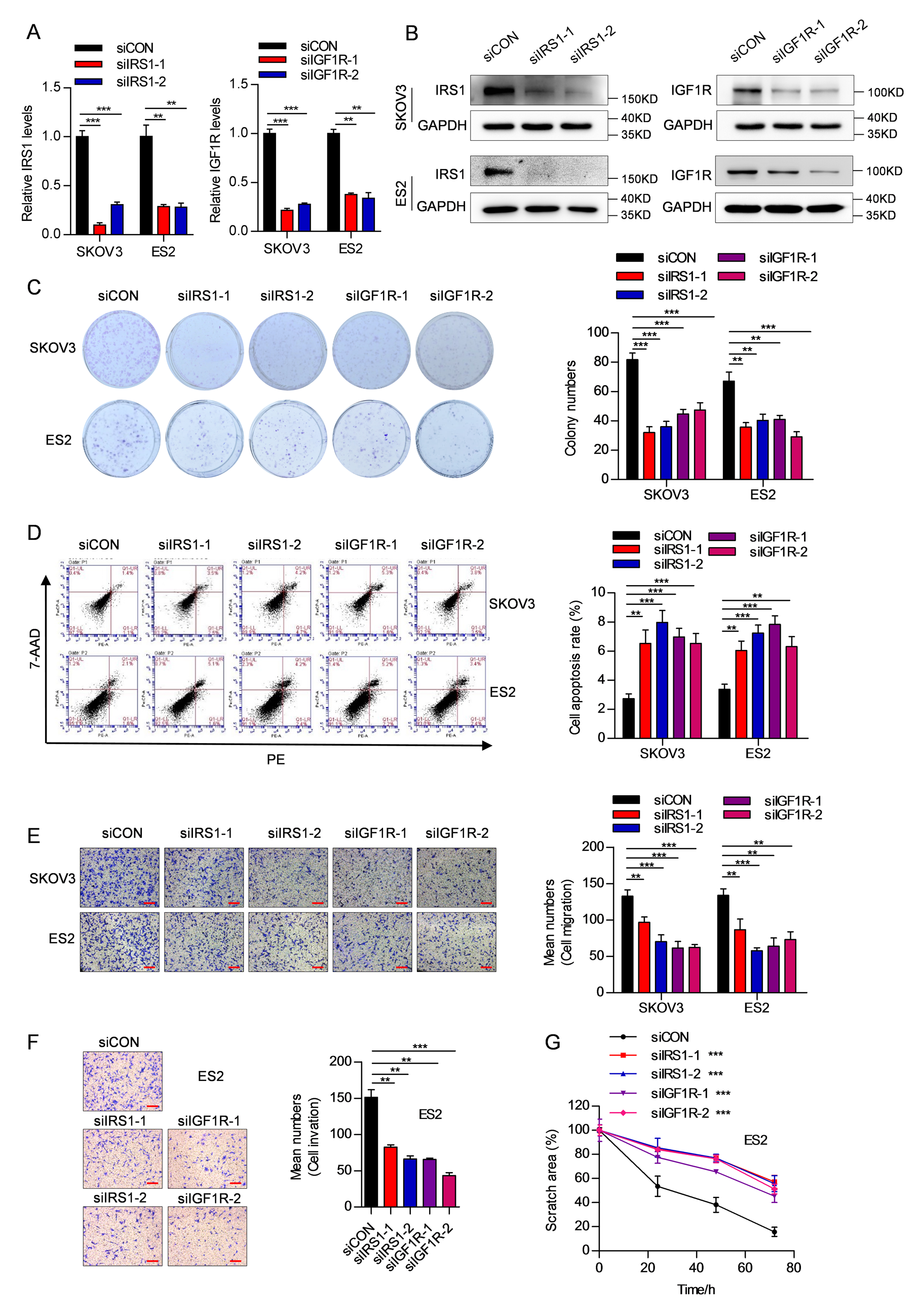

Supplement: Supplementary file 9 — Supplementary Figure S8 [file 41419_2021_3554_MOESM9_ESM.tif]

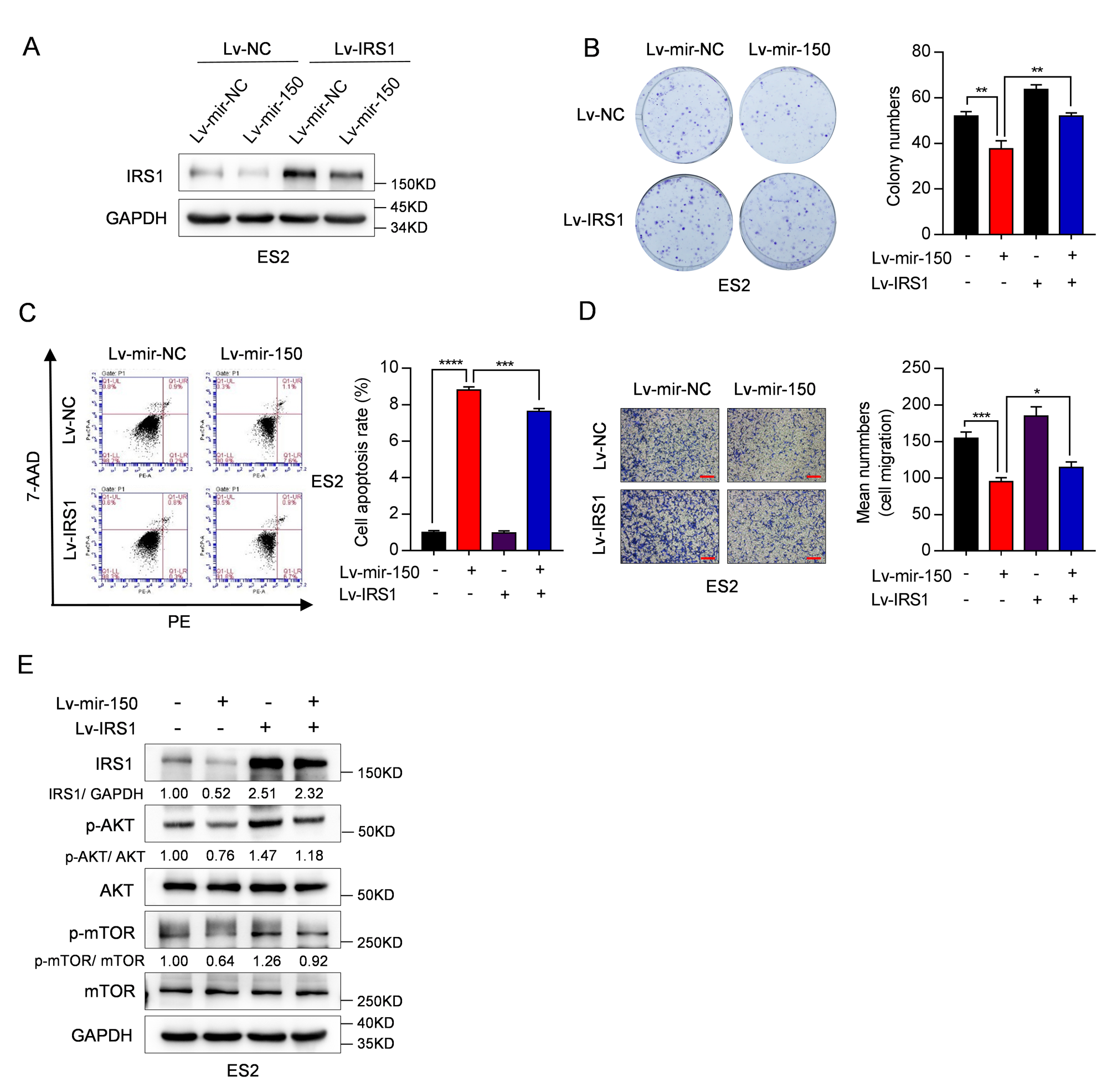

Supplement: Supplementary file 10 — Supplementary Figure S9 [file 41419_2021_3554_MOESM10_ESM.tif]

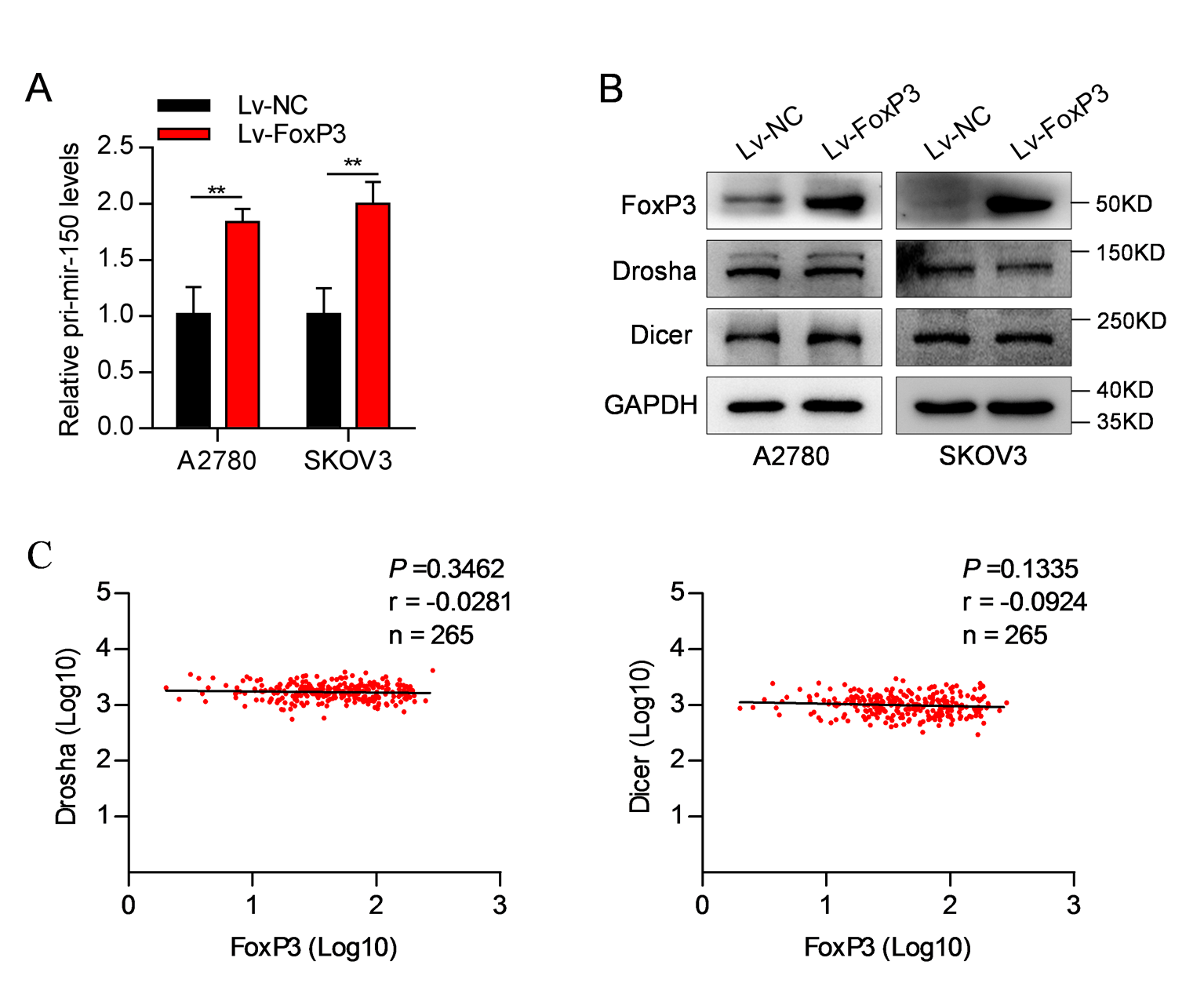

Supplement: Supplementary file 11 — Supplementary Figure S10 [file 41419_2021_3554_MOESM11_ESM.tif]
