# Supplementary material for: FoxP3-miR-150-5p/3p suppresses ovarian tumorigenesis via an IGF1R/IRS1 pathway feedback loop
Source: Cell Death Dis. 2021 Mar 15;12(3):275. doi: 10.1038/s41419-021-03554-6 (PMC7961150; doi:10.1038/s41419-021-03554-6)
Supplement: Supplementary file 12 — Supplementary Table S1-6 [file 41419_2021_3554_MOESM12_ESM.pdf]

Table S1. Oligonucleotides used for real-time RT-PCR, siRNA and plasmid construction.

Name	Forward	Reverse
**Oligonucleotides used for real-time RT-PCR**		
IGF1R	5' CCTGCACAACTCCATCTTCGTG 3'	5' CGGTGATGTTGTAGGTGTCTGC 3'
IRS1	5' AGTCTGTCGTCCAGTAGCACCA 3'	5' ACTGGAGCCATACTCATCCGAG 3'
FoxP3	5' GGCACAATGTCTCCTCCAGAGA 3'	5' CAGATGAAGCCTTGGTCAGTGC 3'
pri-miR-150	5' GAGACGCATAAAAGCCGCAG 3'	5' CTGTCTCCCAACCCTTGTACC 3'
GAPDH	5' ACCCACTCCTCCACCTTTG 3'	5' CACCACCCTGTTGCTGTAG 3'
**Oligonucleotides used for siRNA**		
siIRS1-1	5' CCACGGCGAUCUAGUGCUUTT 3'	5' AAGCACUAGAUCGCCGUGGTT 3'
siIRS1-2	5' GUCAGUCUGUCGUCCAGUATT 3'	5' UACUGGACGACAGACUGACTT 3'
siIGF1R-1	5' UGACUGUGAAAUCUUCGGCTT 3'	5' GCCGAAGAUUUCACAGUCATT 3'
siIGF1R-2	5' CAACAGUGGUCAUCAUGGAACUGAUTT 3'	5' AUCAGUUCCAUGAUGACCACUGUUGTT 3'
siCON	5' UUCUCCGAACGUGUCACGUTT 3'	5' ACGUGACACGUUCGGAGAATT 3'
**Oligonucleotides used for plasmid construction**		
pGL3-IRS1-CDS-1	5' GCCGTGTAATTCTAGAATGAGTGATGAGTTCCGCCC 3'	5' CCGCCCCGACTCTAGAGCCAGACAAGTAGCCAGACT 3'
PGL3-IRS1-CDS-2	5' GCCGTGTAATTCTAGAAAGTGATCCGTGCAGACCC 3'	5' CCGCCCCGACTCTAGAGCTGGCATAGGCGCTTAAAT 3'
PGL3-IRS1-3'UTR	5' GCCGTGTAATTCTAGCTCTGAACGCCTAAGCTCCC 3'	5' CCGCCCCGACTCTAGAAACAGTAACACGGGCCTCAG 3'
pGL3-PSPH-3'UTR	5' GCCGTGTAATTCTAGATGCAGGTTTTGATGAGACGCA 3'	5' CCGCCCCGACTCTAGATCCTCAAGTGATCCTCTTAACTTGT 3'
pGL3-LRIG2-CDS-3'UTR	5' GCCGTGTAATTCTAGATCTTCCCAAGGAACGCTGTC 3'	5' CCGCCCCGACTCTAGATGCAGCCAACACATATGGTCA 3'
pGL3-ARMC9-CDS	5' GCCGTGTAATTCTAGATTCCATCCGAGATGGGGACT 3'	5' CCGCCCCGACTCTAGAGCAGCAACTGAAGCACACTC 3'
pGL3-IGF1R-CDS	5' GCCGTGTAATTCTAGAAGTATGGAGGGGCCAAGCTA 3'	5' CCGCCCCGACTCTAGAATGGCCACTCTGGTTTCAGG 3'
pGL3-IGF1R-3'UTR-1	5' GCCGTGTAATTCTAGACTTCGCACTGGCGTTGAGT 3'	5' CCGCCCCGACTCTAGACCCAGCGAAATCATCAGTGC 3'
pGL3-IGF1R-3'UTR-2	5' GCCGTGTAATTCTAGACTTGGCTTGTGTGATGGTGC 3'	5' CCGCCCCGACTCTAGAGTCATGCCCCAGAACTGACA 3'
pGL6-TA-P1	5' TACCGCTAGCCTCGAGTGCCAAAGCCTCAGATGTGT 3'	5' GCGTGAGCTCCTCGAGGCCCAGTCCCTGCCTTATAC 3'
pGL6-TA-P2	5' TACCGCTAGCCTCGAGCCTTCGGAGTAGCTGGGATTAC 3'	5' GCGTGAGCTCCTCGAGGCCCAGTCCCTGCCTTATAC 3'
pGL6-TA-P3	5' TACCGCTAGCCTCGAGGACTTGTTCCTGAACGATGACAC 3'	5' GCGTGAGCTCCTCGAGGCCCAGTCCCTGCCTTATAC 3'
pGL6-TA-P4	5' TACCGCTAGCCTCGAGCAGAGCAAGAATCTGCCTCAAA 3'	5' GCGTGAGCTCCTCGAGGCCCAGTCCCTGCCTTATAC 3'

Table S2. The expression of 34 dysregulated miRNAs in GSE71477, GSE106817 and GSE61485 datasets.

miRNA ID	GSE71477		GSE106817		GSE61485	
	Fold change (Tumor/Normal)	*P* value	Fold change (Tumor/Normal)	*P* value	Fold change (Tumor/Normal)	*P* value
**Down-regulated miRNAs**						
**hsa-miR-150-5p**	**0.269510**	**0.000001**	**0.457403**	**0.011000**	**0.517355**	**0.007937**
hsa-miR-145-5p	0.485381	0.000006	0.379416	0.001660	0.054554	0.007937
hsa-miR-451a	0.313277	0.000008	0.297450	0.000032	*0.659664*	*0.031746*
hsa-miR-518c-5p	0.348963	0.008471	0.517160	0.016600	*N/A*	*N/A*
hsa-miR-212-3p	0.406041	0.008986	0.362235	0.000741	*1.673435*	*0.095238*
hsa-miR-22-5p	0.454997	0.016304	0.443435	0.007190	*1.745276*	*0.222222*
hsa-miR-365a-5p	0.521766	0.012777	0.713034	0.002860	*N/A*	*N/A*
hsa-miR-23b-3p	0.545373	0.000020	0.440342	0.023400	*0.723956*	*0.055556*
hsa-miR-4448	0.551213	0.024722	0.521951	0.002670	*N/A*	*N/A*
hsa-miR-23a-3p	0.619312	0.000252	0.356969	0.005020	*1.649597*	*0.547619*
hsa-miR-27b-3p	0.695898	0.000647	0.367247	0.001610	*0.482936*	*0.309524*
**hsa-miR-150-3p**	**0.698449**	**0.003790**	**0.629150**	**0.003570**	*N/A*	*N/A*
hsa-miR-4429	0.791528	0.004251	0.521528	0.000004	*1.051932*	*0.690476*
hsa-miR-26a-5p	*0.879905*	*0.028370*	0.328753	0.000399	0.491674	0.007937
hsa-miR-214-3p	*N/A*	*N/A*	0.394840	0.001030	0.268880	0.007937
hsa-miR-133a	0.066024	0.000008	*1.174578*	*0.547000*	0.408387	0.007937
hsa-miR-133b	0.080552	0.000071	*0.773379*	*0.367000*	0.528826	0.007937
hsa-miR-145-3p	0.189794	0.000179	0.944855	*0.809000*	0.385596	0.007937
hsa-miR-4328	0.343004	0.000306	*0.602556*	*0.080200*	0.067231	0.007937
hsa-miR-143-3p	0.350913	0.000006	*0.761872*	*0.425000*	0.266658	0.007937
hsa-miR-486-5p	0.365760	0.001550	*0.759892*	*0.081900*	0.715155	0.015873
hsa-miR-199b-5p	0.393948	0.013815	*1.324926*	*0.383000*	0.670113	0.007937
hsa-miR-424-3p	0.455598	0.003574	0.870471	0.651000	0.639740	0.007937
hsa-miR-140-3p	0.525605	0.000081	0.712241	*0.296000*	0.250924	0.007937
**Up-regulated miRNAs**						
hsa-miR-449b-3p	2.197373	0.014833	1.987477	0.013400	*N/A*	*N/A*
hsa-miR-200c-3p	3.582516	0.000000	*1.700200*	*0.096300*	5.110348	0.015873
hsa-miR-135b-5p	2.687258	0.004332	*0.882441*	*0.717000*	79.478992	0.007937
hsa-miR-877-5p	1.929255	0.010371	*0.607028*	*0.103000*	4.213645	0.007937
hsa-miR-3182	1.880541	0.002013	*0.727933*	*0.248000*	1.959537	0.007937
hsa-miR-17-5p	1.866003	0.000113	*1.104263*	*0.765000*	4.462319	0.007937
hsa-miR-3676-5p	1.606840	0.001458	*N/A*	*N/A*	3.046639	0.015873
hsa-miR-20a-5p	1.573481	0.000327	*1.447138*	*0.264000*	4.613715	0.015873
hsa-miR-205-5p	1.448228	0.001529	*0.622108*	*0.118000*	4.764859	0.007937
hsa-miR-92b-3p	1.258464	0.009361	*0.733885*	*0.221000*	2.956566	0.007937

Table S3. The identified proteins in Lv-mir-150-A2780 and Lv-mir-NC-A2780 cells by iTRAQ.

Accession	Gene Symbol	Description	Unique Peptides	Abundances C1 (Lv-mir-NC-A2780)	Abundances C2 (Lv-mir-NC-A2780)	Abundances M1 (Lv-mir-150-A2780)	Abundances M2 (Lv-mir-150-A2780)	Average C12	Average M12	M12/C12	*P* value
A8KA30		Oxysterol-binding protein OS=Homo sapiens	1	79.5	80.9	239.4	66.5	80.2	152.95	1.907107232	1.83503E-32
A0A087X186	ZNF658	Zinc finger protein 658 (Fragment) OS=Homo	1	57.2	80.4	111	139.4	68.8	125.2	1.819767442	2.87817E-28
A8K1Z4		cDNA FLJ76704, highly similar to Homo sap	1	105.8	64.4	154.2	132.8	85.1	143.5	1.686251469	4.30107E-22
J3QRZ1	NDEL1	Nuclear distribution protein nudE-like 1 (Frag	1	93.3	78.9	157.9	115.5	86.1	136.7	1.587688734	8.90468E-18
B4E1C2	KNG1	Kininogen 1, isoform CRA_b OS=Homo sapi	2	75.2	71.7	111.3	112.8	73.45	112.05	1.52552757	3.44841E-15
Q330K2	NDUFAF6	NADH dehydrogenase (ubiquinone) complex	1	85.4	86.7	104.6	154.6	86.05	129.6	1.506101104	2.09826E-14
P0C0L4	C4A	Complement C4-A OS=Homo sapiens GN=C	9	76.5	71.6	104.5	117.6	74.05	111.05	1.49966239	3.79332E-14
Q6ZRA8		cDNA FLJ46514 fis, clone THYMU3032798,	1	65.3	84.4	103	118.4	74.85	110.7	1.478957916	2.49024E-13
U6BN61	HLA-B	MHC class I antigen (Fragment) OS=Homo sa	2	75.4	89	115.4	124.1	82.2	119.75	1.456812652	1.79172E-12
P02747	C1QC	Complement C1q subcomponent subunit C OS	1	75.2	73.9	109.1	107.8	74.55	108.45	1.45472837	2.15273E-12
V9HWD8	HEL-S-163pA	Epididymis secretory sperm binding protein L	7	73.1	74.5	104.3	108.7	73.8	106.5	1.443089431	5.95791E-12
D9ZGG2	VTN	Vitronectin OS=Homo sapiens GN=VTN PE=	3	91.6	83.1	130.9	121	87.35	125.95	1.441900401	6.60639E-12
A0A024R944	SERPINC1	Serpin peptidase inhibitor, clade C (Antithrom	3	83.1	76.5	117.7	110.5	79.8	114.1	1.429824561	1.87275E-11
P02790	HPX	Hemopexin OS=Homo sapiens GN=HPX PE=	14	76.1	74.7	104.8	108.8	75.4	106.8	1.416445623	5.84692E-11
P25311	AZGP1	Zinc-alpha-2-glycoprotein OS=Homo sapiens	2	81.9	69.5	106.2	108.1	75.7	107.15	1.415455746	6.35649E-11
Q96I36	COX14	Cytochrome c oxidase assembly protein COX	1	87.6	86	103.9	140.8	86.8	122.35	1.409562212	1.04332E-10
P10909	CLU	Clusterin OS=Homo sapiens GN=CLU PE=1	2	66.1	69.9	96.1	93.2	68	94.65	1.391911765	4.50814E-10
B3KS79		cDNA FLJ35730 fis, clone TESTI2003131, h	8	80.9	79.9	109.9	111.9	80.4	110.9	1.379353234	1.25265E-09
Q494V2	CFAP100	Cilia- and flagella-associated protein 100 OS=	1	91.6	79.6	127.7	107.9	85.6	117.8	1.376168224	1.61903E-09
A8K4B4		cDNA FLJ78441, highly similar to Homo sap	4	85.8	87.7	121.8	112.8	86.75	117.3	1.352161383	1.0809E-08
Q9UMS6	SYNPO2	Synaptopodin-2 OS=Homo sapiens GN=SYN	5	93.2	86.5	121.3	121.5	89.85	121.4	1.35114079	1.17012E-08
Q562Z4	ACT	Actin-like protein (Fragment) OS=Homo sapi	1	72	90.7	98.2	121.6	81.35	109.9	1.350952674	1.18733E-08
K0A7K7	TRAP1	TNF receptor-associated protein 1 (Fragment)	1	97.8	83.1	85.7	158.1	90.45	121.9	1.347705915	1.52672E-08
B7Z8Q2		cDNA FLJ55606, highly similar to Alpha-2-H	3	85.5	75.8	113.7	103	80.65	108.35	1.343459392	2.11732E-08
Q9Y5W9	SNX11	Sorting nexin-11 OS=Homo sapiens GN=SNX	1	91.7	89.2	104.2	135.8	90.45	120	1.326699834	7.54102E-08
A0A126GW78	OR5AC2	Olfactory receptor OS=Homo sapiens GN=OI	1	97	89.2	130.2	116.5	93.1	123.35	1.324919441	8.61358E-08
D6RF35	GC	Vitamin D-binding protein OS=Homo sapiens	3	83	82.6	106.5	112.5	82.8	109.5	1.322463768	1.03412E-07
D9YZV5	TPM1	Tropomyosin 1 (Alpha) isoform 4 OS=Homo	1	91.4	92.3	114.4	127.5	91.85	120.95	1.316820904	1.5696E-07
A0A0B4J2C1	MRPL51	39S ribosomal protein L51, mitochondrial OS	1	81.1	83	120.6	93.4	82.05	107	1.304082876	3.96811E-07
C0JYY2	APOB	Apolipoprotein B (Including Ag(X) antigen) (	14	95.7	86.4	122.3	114	91.05	118.15	1.29763866	6.29474E-07
V9HWA5	HEL-S-14	Calponin OS=Homo sapiens GN=HEL-S-14 I	3	93.2	93.7	118.4	123.4	93.45	120.9	1.293739968	8.30029E-07
Q96CC6	RHBDF1	Inactive rhomboid protein 1 OS=Homo sapien	1	85.1	83.4	77.7	140.1	84.25	108.9	1.292581602	9.00773E-07
A0A024QZN7	C10orf70	Chromosome 10 open reading frame 70, isofo	4	87.6	90	113.4	116.1	88.8	114.75	1.29222973	9.23398E-07
A0A140VKG2		Testis tissue sperm-binding protein Li 79P OS	3	88.9	94.6	115.5	121.5	91.75	118.5	1.291553134	9.68478E-07
P02749	APOH	Beta-2-glycoprotein 1 OS=Homo sapiens GN	3	79.9	78.9	96.4	108.6	79.4	102.5	1.29093199	1.01175E-06
B7ZKJ8	ITIH4	ITIH4 protein OS=Homo sapiens GN=ITIH4	3	79.1	86.7	102.9	111	82.9	106.95	1.290108565	1.07201E-06
Q9UIB8	CD84	SLAM family member 5 OS=Homo sapiens G	1	78.8	98.2	102.9	124.9	88.5	113.9	1.28700565	1.33212E-06
B2R8I2		cDNA, FLJ93914, highly similar to Homo sap	2	77.4	88.2	104.3	108.3	82.8	106.3	1.283816425	1.66317E-06
E9PGN7	SERPING1	Plasma protease C1 inhibitor OS=Homo sapie	4	82.3	87.2	109.4	107.9	84.75	108.65	1.2820059	1.88538E-06
F8W914	RTN4	Reticulon OS=Homo sapiens GN=RTN4 PE=	1	92.7	87.1	112	117.8	89.9	114.9	1.278086763	2.46966E-06
Q9B2U7	COX1	Cytochrome c oxidase subunit 1 OS=Homo sa	1	96.2	69.7	111.8	100.1	82.95	105.95	1.277275467	2.61093E-06
Q9Y4D1	DAAM1	Disheveled-associated activator of morphogen	1	78.7	102.4	106.2	124.7	90.55	115.45	1.274986195	3.05319E-06
B2RCY4		Serine--pyruvate aminotransferase OS=Homo	1	88.9	93.6	101.3	131	91.25	116.15	1.272876712	3.5245E-06
D3DRR6	ITIH2	Inter-alpha (Globulin) inhibitor H2, isoform C	3	81	81.8	103.4	103.6	81.4	103.5	1.271498771	3.86973E-06
A0A024R2Z6	GNL3	Guanine nucleotide binding protein-like 3 (Nu	1	92.3	87.4	120	108.3	89.85	114.15	1.270450751	4.15407E-06
Q9Y5U9	IER3IP1	Immediate early response 3-interacting protein	1	87	87	112.6	108.4	87	110.5	1.270114943	4.24938E-06
P00734	F2	Prothrombin OS=Homo sapiens GN=F2 PE=1	6	90.2	87.1	110.8	114.2	88.65	112.5	1.269035533	4.57032E-06
Q96DE5	ANAPC16	Anaphase-promoting complex subunit 16 OS=	1	88.7	94.8	91.5	140.2	91.75	115.85	1.2626703	6.99847E-06
H0YE68	WHSC1L1	Histone-lysine N-methyltransferase NSD3 (Fr	1	98.4	85.7	112.5	119.9	92.05	116.2	1.262357414	7.14552E-06
Q5EBL8	PDZD11	PDZ domain-containing protein 11 OS=Homo	1	87.2	85.9	96.6	121.5	86.55	109.05	1.259965338	8.37339E-06
B7Z1F8		cDNA FLJ53025, highly similar to Compleme	1	90.9	82.2	87.1	130.4	86.55	108.75	1.256499133	1.05212E-05
Q01415	GALK2	N-acetylgalactosamine kinase OS=Homo sapi	1	89.1	88	85.8	136	88.55	110.9	1.252399774	1.37527E-05
O94964	SOGA1	Protein SOGA1 OS=Homo sapiens GN=SOG	1	97.9	90.1	106	128.9	94	117.45	1.249468085	1.66317E-05
Q9H2U2	PPA2	Inorganic pyrophosphatase 2, mitochondrial C	4	95.6	91.2	117.8	115.4	93.4	116.6	1.248394004	1.78257E-05
K7ELP0	TPM4	Tropomyosin alpha-4 chain (Fragment) OS=H	1	94.7	87.8	99.2	128.6	91.25	113.9	1.248219178	1.80278E-05
Q9NQR1	KMT5A	N-lysine methyltransferase KMT5A OS=Hom	1	80.7	121.9	170.3	80.9	101.3	125.6	1.23988154	3.06965E-05
Q9UHD2	TBK1	Serine/threonine-protein kinase TBK1 OS=Ho	3	88.3	91.8	89.5	133.7	90.05	111.6	1.239311494	3.18222E-05
A4D1P6	WDR91	WD repeat-containing protein 91 OS=Homo s	1	73.6	86.9	97.2	101.7	80.25	99.45	1.239252336	3.19413E-05
B2RCP7		cDNA, FLJ96197, highly similar to Homo sap	2	106.8	95.1	131.7	118.2	100.95	124.95	1.237741456	3.51319E-05
Q08431	MFGE8	Lactadherin OS=Homo sapiens GN=MFGE8	13	87.1	94	116	108	90.55	112	1.236885699	3.70732E-05
A0A1B2FJA7	APOL1	Apolipoprotein L1 (Fragment) OS=Homo sap	1	92.3	94.1	98.5	131.3	93.2	114.9	1.232832618	4.77564E-05
P40189	IL6ST	Interleukin-6 receptor subunit beta OS=Homo	1	85.7	93.2	98.9	120.3	89.45	109.6	1.225265511	7.61171E-05
P02750	LRG1	Leucine-rich alpha-2-glycoprotein OS=Homo	1	97.9	83.1	106.2	115.2	90.5	110.7	1.22320442	8.62916E-05
V9HVY1	HEL-S-78p	Epididymis secretory sperm binding protein L	3	96.2	98.5	103.8	134.2	97.35	119	1.222393426	9.06421E-05
Q05682	CALD1	Caldesmon OS=Homo sapiens GN=CALD1 F	10	96.8	96.6	118.1	118.1	96.7	118.1	1.221302999	9.68237E-05
P02792	FTL	Ferritin light chain OS=Homo sapiens GN=FT	7	90.5	88.8	108.5	110.3	89.65	109.4	1.220301171	0.000102858
A0A0J9YX62	DNAJB6	DnaJ homolog subfamily B member 6 OS=Ho	1	93.8	93.8	108	120	93.8	114	1.215351812	0.00013834
A0A024R1S2	CDC6	CDC6 cell division cycle 6 homolog (S. cerev	1	100.6	80.4	98.9	120.8	90.5	109.85	1.213812155	0.000151583
Q9BWH2	FUNDC2	FUN14 domain-containing protein 2 OS=Hom	3	93.2	87.1	104.1	114.7	90.15	109.4	1.213533001	0.000154109
A0A0A0MTJ9	NCEH1	Neutral cholesterol ester hydrolase 1 OS=Hom	10	94.9	93.8	114.5	114.4	94.35	114.45	1.213036566	0.000158704
Q9H8M2	BRD9	Bromodomain-containing protein 9 OS=Homo	1	86.5	95.8	112.7	108.2	91.15	110.45	1.211738892	0.000171338
B4E1Z4		Uncharacterized protein OS=Homo sapiens PI	3	86.3	82.8	94.1	110.7	84.55	102.4	1.211117682	0.000177722
C8C504	HBB	Beta-globin OS=Homo sapiens GN=HBB PE=	4	93.5	94.2	107	120.1	93.85	113.55	1.20990943	0.000190791
A0A0B5HR54		Serine/threonine-protein kinase receptor OS=I	1	98.8	86	93.2	129.9	92.4	111.55	1.207251082	0.00022285
Q9BT67	NDFIP1	NEDD4 family-interacting protein 1 OS=Hom	2	98.6	85.1	109.4	112.1	91.85	110.75	1.205770278	0.000242871
A0A024R0Z5	TEGT	Testis enhanced gene transcript (BAX inhibito	2	92.3	93.1	107.5	115.3	92.7	111.4	1.201725998	0.00030665
Q96A08	HIST1H2BA	Histone H2B type 1-A OS=Homo sapiens GN	1	80.5	124	170.6	74.9	102.25	122.75	1.200488998	0.000329143
Q8NE91	TM4SF1	TM4SF1 protein OS=Homo sapiens GN=TM4	1	113.3	99.7	89.9	87.5	106.5	88.7	0.83286385	0.00730009
Q9Y426	C2CD2	C2 domain-containing protein 2 OS=Homo sa	1	120.8	110	104.2	88	115.4	96.1	0.832755633	0.007254
Q53TN4	CYBRD1	Cytochrome b reductase 1 OS=Homo sapiens	1	109.7	106.6	93.3	86.6	108.15	89.95	0.83171521	0.00682386
Q86X53	ERICH1	Glutamate-rich protein 1 OS=Homo sapiens C	1	109	108.2	82.8	97.8	108.6	90.3	0.831491713	0.00673442
O75382	TRIM3	Tripartite motif-containing protein 3 OS=Hom	2	109.4	109.6	94.4	87.6	109.5	91	0.831050228	0.0065608
Q5VW26	NFIB	Nuclear factor 1 OS=Homo sapiens GN=NFIB	1	119.5	92.9	95	81.5	106.2	88.25	0.830979284	0.00653328
Q9HBG6	IFT122	Intraflagellar transport protein 122 homolog C	3	111.3	103.4	89	89.4	107.35	89.2	0.830926875	0.00651301
Q9Y2H0	DLGAP4	Disks large-associated protein 4 OS=Homo sa	1	101.3	105.7	88.1	83.9	103.5	86	0.830917874	0.00650954
Q14103	HNRNPD	Heterogeneous nuclear ribonucleoprotein D0 (	2	108.6	111.1	93	89.5	109.85	91.25	0.830678198	0.00641758
Q9NPQ8	RIC8A	Synembryn-A OS=Homo sapiens GN=RIC8A	3	122.6	99.9	95.3	89.5	111.25	92.4	0.830561798	0.00637333
B8PRF2	CD160	CD160 antigen OS=Homo sapiens GN=CD16	1	110.7	109.4	93.5	89.2	110.05	91.35	0.830077238	0.00619199
Q59GJ0		Eukaryotic translation initiation factor 4 gamr	1	123.9	103.2	94.3	94	113.55	94.15	0.829150154	0.00585767
Q99504	EYA3	Eyes absent homolog 3 OS=Homo sapiens GN	1	129.5	100.6	96.3	94.3	115.05	95.3	0.828335506	0.00557715
Q6P1R4	DUS1L	tRNA-dihydrouridine(16/17) synthase [NAD(	1	117.3	104.4	90	93.5	110.85	91.75	0.827695083	0.005365
O75223	GGCT	Gamma-glutamylcyclotransferase OS=Homo	3	100.6	116.5	88.9	90.7	108.55	89.8	0.82726854	0.00522769
Q5JVS0	HABP4	Intracellular hyaluronan-binding protein 4 OS	1	136.4	90.1	88.7	98.6	113.25	93.65	0.826931567	0.00512144
A0A024R035	C9	Complement component 9, isoform CRA_a O	2	118.3	105.7	96.7	88.5	112	92.6	0.826785714	0.00507604
Q5TZC3	PACSIN1	Protein kinase C and casein kinase substrate in	1	89	88.2	80.6	65.8	88.6	73.2	0.826185102	0.00489279
A6NDB9	PALM3	Paralemmin-3 OS=Homo sapiens GN=PALM	2	116.5	97.9	83.4	93.7	107.2	88.55	0.826026119	0.00484528
Q9BUT9	MCRIP2	MAPK regulated corepressor interacting prote	1	118.4	106.2	75.4	110.1	112.3	92.75	0.825912734	0.00481164
Q92599	SEPT8	Septin-8 OS=Homo sapiens GN=SEPT8 PE=	1	110.1	110.4	91.4	90.5	110.25	90.95	0.824943311	0.0045324
A8K6U2		cDNA FLJ78675, highly similar to Homo sap	1	120	98.5	90.6	89.6	109.25	90.1	0.824713959	0.00446847
O75317	USP12	Ubiquitin carboxyl-terminal hydrolase 12 OS=	1	114.1	111.9	96.2	90.1	113	93.15	0.824336283	0.00436495
B4DLT5		cDNA FLJ56739, highly similar to Alpha-gal	1	113.5	97.5	87.4	86.5	105.5	86.95	0.824170616	0.00432021
P54289	CACNA2D1	Voltage-dependent calcium channel subunit a	4	125.4	114	101.2	96.1	119.7	98.65	0.824143693	0.00431299
A8K8Y8		cDNA FLJ77796, highly similar to Homo sap	2	102.5	117.9	89.2	92.4	110.2	90.8	0.823956443	0.00426298
Q14442	PIGH	Phosphatidylinositol N-acetylglucosaminyltra	1	124.3	99.6	88.9	95.5	111.95	92.2	0.823581956	0.00416452
P42574	CASP3	Caspase-3 OS=Homo sapiens GN=CASP3 PE	2	114.1	110.6	94.6	90.3	112.35	92.45	0.822874944	0.00398414
Q9NV88	INTS9	Integrator complex subunit 9 OS=Homo sapie	1	107.4	101.3	88.2	83.2	104.35	85.7	0.821274557	0.00360123
Q9H8M5	CNNM2	Metal transporter CNNM2 OS=Homo sapiens	2	108.5	101.5	90.2	82.2	105	86.2	0.820952381	0.00352822
Q9H9A6	LRRC40	Leucine-rich repeat-containing protein 40 OS=	1	109.3	106.8	75.6	101.8	108.05	88.7	0.820916242	0.00352012
Q6JQN1	ACAD10	Acyl-CoA dehydrogenase family member 10	1	108.2	81.8	75.3	80.5	95	77.9	0.82	0.00332008
Q15323	KRT31	Keratin, type I cuticular Ha1 OS=Homo sapie	1	118.9	106.7	99.2	85.5	112.8	92.35	0.818705674	0.00305477
Q9UBC3	DNMT3B	DNA (cytosine-5)-methyltransferase 3B OS=I	1	130.8	113.4	101.3	98.6	122.1	99.95	0.818591319	0.00303226
Q9NTM9	CUTC	Copper homeostasis protein cutC homolog OS	1	111.9	111.3	93.8	88.8	111.6	91.3	0.818100358	0.00293732
A0A024R1V5	MLLT6	Myeloid/lymphoid or mixed-lineage leukemia	1	118	103.5	88.3	92.9	110.75	90.6	0.818058691	0.00292939
Q4LE65	PIK3C2B	PIK3C2B variant protein (Fragment) OS=Hor	1	101.1	106.6	81.4	88.5	103.85	84.95	0.81800674	0.00291953
Q96HA7	TONSL	Tonsoku-like protein OS=Homo sapiens GN=	1	117.4	97.7	78.6	97.2	107.55	87.9	0.817294282	0.0027872
Q9BUW7	C9orf16	UPF0184 protein C9orf16 OS=Homo sapiens	1	117.9	99.7	79.6	98.2	108.8	88.9	0.817095588	0.00275126
B4DE53		cDNA FLJ61213, highly similar to Tubulin-sp	1	109	97.3	81.1	87.4	103.15	84.25	0.816771692	0.00269357

B2RC53		cDNA, FLJ95856, highly similar to Homo sap	1	108.7	114.4	86.8	95.1	111.55	90.95	0.815329449	0.00244961
A0A024R9Y2	KIAA1267	KIAA1267, isoform CRA_a OS=Homo sapien	1	121.1	111.6	93.1	96.5	116.35	94.8	0.814782982	0.00236248
A0A024RC31	KIAA1012	KIAA1012, isoform CRA_b OS=Homo sapie	3	115.8	106.2	95.3	85.4	111	90.35	0.813963964	0.0022371
A0A024RBE6	NAP1L1	Nucleosome assembly protein 1-like 1, isofor	1	127.8	98.9	86.4	98.1	113.35	92.25	0.813850904	0.00222026
P51687	SUOX	Sulfite oxidase, mitochondrial OS=Homo sap	3	124.3	102.2	90.1	94	113.25	92.05	0.812803532	0.00206966
O75379	VAMP4	Vesicle-associated membrane protein 4 OS=H	1	128.7	109	93.7	99.5	118.85	96.6	0.81278923	0.00206767
H0YMV8	RPS27L	40S ribosomal protein S27 OS=Homo sapiens	1	113	96.5	90.6	79.5	104.75	85.05	0.811933174	0.00195158
Q16595	FXN	Frataxin, mitochondrial OS=Homo sapiens Gl	4	130.7	95.2	92.3	91.1	112.95	91.7	0.811863656	0.00194242
A8K0E7		cDNA FLJ77464, highly similar to Homo sap	2	106.9	107.4	83.5	90.4	107.15	86.95	0.811479235	0.00189243
P13984	GTF2F2	General transcription factor IIF subunit 2 OS=	1	112.9	116.5	98.1	88	114.7	93.05	0.811246731	0.00186276
A0A024QZW1	RANBP9	RAN binding protein 9, isoform CRA_a OS=l	3	131.6	102	93.2	96.1	116.8	94.65	0.810359589	0.00175337
Q96EM0	L3HYPDH	Trans-3-hydroxy-L-proline dehydratase OS=H	1	135.4	96.8	86.7	101.4	116.1	94.05	0.810077519	0.00171983
A0A024R499	IRS1	Insulin receptor substrate 1, isoform CRA_a C	1	114.5	101.7	91.6	83.5	108.1	87.55	0.809898242	0.0016988
E9PNQ8	THY1	Thy-1 membrane glycoprotein (Fragment) OS	1	105.1	97.3	81	82.9	101.2	81.95	0.809782609	0.00168537
Q8N5L8	RPP25L	Ribonuclease P protein subunit p25-like prote	1	120	107.1	101.8	82.1	113.55	91.95	0.809775429	0.00168454
B4E2P4		cDNA FLJ58474, highly similar to Xaa-Pro a	1	112.1	105.1	88.8	86.9	108.6	87.85	0.80893186	0.00158944
P61254	RPL26	60S ribosomal protein L26 OS=Homo sapiens	3	108.2	98.1	85.3	81.5	103.15	83.4	0.808531265	0.00154599
Q14114	LRP8	Low-density lipoprotein receptor-related prote	1	124.1	100.3	91.9	89.5	112.2	90.7	0.808377897	0.00152965
Q13088		ZEB (Fragment) OS=Homo sapiens PE=2 SV	1	130.2	101.4	80	106.9	115.8	93.45	0.806994819	0.00138914
V9HWA9	HEL-S-62p	Epididymis secretory sperm binding protein L	1	111.5	105.8	87.4	87.9	108.65	87.65	0.806718822	0.00136254
A0A087X1I8	ARMC9	LisH domain-containing protein ARMC9 OS=	1	120.6	96.9	86.9	88.5	108.75	87.7	0.806436782	0.00133584
Q9BW61	DDA1	DET1- and DDB1-associated protein 1 OS=H	2	103.2	127.2	95.9	89.9	115.2	92.9	0.806423611	0.0013346
A0A024RAQ3	DHFR	Dihydrofolate reductase, isoform CRA_a OS=	4	124.7	102.1	94.1	88.6	113.4	91.35	0.805555556	0.00125542
Q8IVP5	FUNDC1	FUN14 domain-containing protein 1 OS=Hon	1	132.7	96.5	89.9	94	114.6	91.95	0.802356021	0.000998961
Q99447	PCYT2	Ethanolamine-phosphate cytidylyltransferase	1	114.1	106.9	88.9	88.2	110.5	88.55	0.801357466	0.000929275
A8K9U2		cDNA FLJ77474, highly similar to Homo sap	1	118.4	107.8	88.5	92.7	113.1	90.6	0.801061008	0.000909456
A0A024R563	PPP1R14B	Protein phosphatase 1, regulatory (Inhibitor) s	2	111	95.5	79	86.4	103.25	82.7	0.800968523	0.000903351
Q495G5	MMAA	MMAA protein OS=Homo sapiens GN=MMA	1	118.7	101.9	90.7	85.9	110.3	88.3	0.800543971	0.000875804
A0A024RDL9	PSPH	Phosphoserine phosphatase, isoform CRA_b C	1	106.9	135.4	106.9	86.4	121.15	96.65	0.797771358	0.000713915
P11226	MBL2	Mannose-binding protein C OS=Homo sapien	1	138.7	91.1	92.5	90.6	114.9	91.55	0.796779809	0.000662998
Q99426	TBCB	Tubulin-folding cofactor B OS=Homo sapiens	6	111	110.9	89.8	87	110.95	88.4	0.796755295	0.000661783
Q96BH1	RNF25	E3 ubiquitin-protein ligase RNF25 OS=Homo	2	118	114.9	93.4	92.1	116.45	92.75	0.796479176	0.000648227
A0A0S2Z5S7	MIF4GD	MIF4G domain containing isoform 2 (Fragme	1	109.2	111.8	93.1	82.8	110.5	87.95	0.795927602	0.000621908
Q6IBU4	SDF2	SDF2 protein OS=Homo sapiens GN=SDF2 F	3	133.1	102.1	92.4	94.7	117.6	93.55	0.795493197	0.000601871
B2R7S7		cDNA, FLJ93585, highly similar to Homo sap	6	104.6	106.5	81.4	86.5	105.55	83.95	0.79535765	0.000595741
P27986	PIK3R1	Phosphatidylinositol 3-kinase regulatory subu	1	119.8	100	95	79.8	109.9	87.4	0.795268426	0.000591737
B4E1C1		cDNA FLJ55943, highly similar to Death don	4	129.2	100.9	92.5	90.4	115.05	91.45	0.794871795	0.000574235
Q9Y508	RNF114	E3 ubiquitin-protein ligase RNF114 OS=Hom	1	128.4	102.1	88.9	94.3	115.25	91.6	0.794793926	0.000570853
F2Z2X0	NME4	Nucleoside diphosphate kinase OS=Homo sap	2	108.1	121.1	90.1	92	114.6	91.05	0.794502618	0.000558369
B4E0C4		Poly [ADP-ribose] polymerase OS=Homo sap	2	138.3	100.3	101.2	88.2	119.3	94.7	0.79379715	0.000529162
A0A024RBD0	NUDT4	Nudix (Nucleoside diphosphate linked moiety	2	123.7	103.7	87.6	92.9	113.7	90.25	0.793755497	0.000527482
Q13426	XRCC4	DNA repair protein XRCC4 OS=Homo sapier	1	107.1	129.1	100.1	87.3	118.1	93.7	0.793395428	0.000513162
A0A0S2Z4U9	USH1C	Usher syndrome 1C (Autosomal recessive, sev	1	119.6	108.8	95.4	85.5	114.2	90.45	0.792031524	0.00046209
E9PGC8	MAP1A	Microtubule-associated protein 1A OS=Homo	1	139.5	90.1	94.1	87.7	114.8	90.9	0.791811847	0.000454314
Q53GZ2		Polymerase (RNA) III (DNA directed) polype	2	125.3	105.1	95.6	86.8	115.2	91.2	0.791666667	0.000449242
Q4LE70	APC	APC variant protein (Fragment) OS=Homo sa	2	124.3	102.2	101.6	77.3	113.25	89.45	0.789845475	0.000389866
O15482	TEX28	Testis-specific protein TEX28 OS=Homo sap	1	134.3	94.5	84.9	95.6	114.4	90.25	0.788898601	0.000361928
Q3B7T1	EDRF1	Erythroid differentiation-related factor 1 OS=	1	107.7	114.6	90.6	84.7	111.15	87.65	0.788573999	0.000352783
Q86SK9	SCD5	Stearoyl-CoA desaturase 5 OS=Homo sapiens	1	127.4	102	89.4	91.4	114.7	90.4	0.788142982	0.000340967
Q03001	DST	Dystonin OS=Homo sapiens GN=DST PE=1	2	139.9	94.2	95.8	88.6	117.05	92.2	0.787697565	0.00032914
P62875	POLR2L	DNA-directed RNA polymerases I, II, and III	2	143.7	90.4	94.1	90.1	117.05	92.1	0.786843229	0.000307505
B3KMW3		cDNA FLJ12779 fis, clone NT2RP2001748, r	1	119.5	108.1	87.6	91.2	113.8	89.4	0.785588752	0.000278099
A4ZVS7	PIAS1	Protein inhibitor of activated STAT1 OS=Hor	2	129.8	93	86.4	88.5	111.4	87.45	0.785008977	0.000265404
Q6ZRN6		cDNA FLJ46220 fis, clone TESTI4013774 O	1	141	127	154	55.6	134	104.8	0.782089552	0.000209215
P36542	ATP5C1	ATP synthase subunit gamma, mitochondrial	1	91.5	152.3	101.9	88.7	121.9	95.3	0.781788351	0.000204092
Q9P260	KIAA1468	LisH domain and HEAT repeat-containing pro	1	114.3	107.9	89.9	83.8	111.1	86.85	0.781728173	0.000203082
Q9H7D0	DOCK5	Dedicator of cytokinesis protein 5 OS=Homo	1	134.3	98.5	79.2	102.6	116.4	90.9	0.780927835	0.000190087
Q9Y2J4	AMOTL2	Angiomotin-like protein 2 OS=Homo sapiens	1	130.8	97	91.9	85.8	113.9	88.85	0.780070237	0.000177018
M0R2R1	ZNF766	Zinc finger protein 766 (Fragment) OS=Homo	1	123.6	115	104	81.9	119.3	92.95	0.779128248	0.000163623
Q969M7	UBE2F	NEDD8-conjugating enzyme UBE2F OS=Ho	1	116.3	102.7	92.4	78.2	109.5	85.3	0.778995434	0.000161812
Q8TEQ8	PIGO	GPI ethanolamine phosphate transferase 3 OS	2	131.8	116.1	106.4	86.4	123.95	96.4	0.777732957	0.000145499
Q96D05	C10orf35	Uncharacterized protein C10orf35 OS=Homo	2	122.3	105.8	82.5	94.2	114.05	88.35	0.774660237	0.000111946
A0A090N8Y9	LOC392652	Similar to RP9 protein OS=Homo sapiens GN	1	122.1	114.8	94.9	88.5	118.45	91.7	0.774166315	0.000107276
O60671	RAD1	Cell cycle checkpoint protein RAD1 OS=Hon	1	109.2	103	77.2	86.5	106.1	81.85	0.771442036	8.46117E-05
Q5W0V3	FAM160B1	Protein FAM160B1 OS=Homo sapiens GN=F	1	110.4	93.9	79.4	77.7	102.15	78.55	0.768967205	6.79656E-05
P0DJ93	SMIM13	Small integral membrane protein 13 OS=Hom	1	125	98.3	76.5	95.2	111.65	85.85	0.768920734	6.76843E-05
Q9P0B6	CCDC167	Coiled-coil domain-containing protein 167 OS	1	135	97.6	87.2	90.4	116.3	88.8	0.763542562	0.000041559
Q9ULF5	SLC39A10	Zinc transporter ZIP10 OS=Homo sapiens GN	3	139.6	105.4	100.3	86.6	122.5	93.45	0.762857143	3.90097E-05
A0A0A0MRP1	FPGT	Fucose-1-phosphate guanylyltransferase OS=l	1	113.8	105.7	81.6	85.7	109.75	83.65	0.762186788	3.66583E-05
Q5JTD0	TJAP1	Tight junction-associated protein 1 OS=Homo	1	124.9	115.6	93.2	88.9	120.25	91.05	0.757172557	2.28424E-05
Q562E7	WDR81	WD repeat-containing protein 81 OS=Homo s	1	121.3	114.1	88.1	89.8	117.7	88.95	0.755734919	1.98929E-05
Q96B77	TMEM186	Transmembrane protein 186 OS=Homo sapien	2	132.1	83.8	80.1	83	107.95	81.55	0.755442334	1.93382E-05
Q9NVQ4	FAIM	Fas apoptotic inhibitory molecule 1 OS=Hom	1	108.9	114.2	86	82.3	111.55	84.15	0.754370238	1.74272E-05
H0Y7T8	AMPH	Amphiphysin (Fragment) OS=Homo sapiens (	2	135.7	107.1	99	83.7	121.4	91.35	0.75247117	1.44703E-05
P55789	GFER	FAD-linked sulfhydryl oxidase ALR OS=Hor	4	144	90.6	81	94.4	117.3	87.7	0.747655584	8.94583E-06
Q9UHW5	GPN3	GPN-loop GTPase 3 OS=Homo sapiens GN=	2	130.6	107.1	86.2	90.4	118.85	88.3	0.742953302	5.5199E-06
A0A024RBV2	AACS	Acetoacetyl-CoA synthetase, isoform CRA_b	1	130.1	100.2	80.6	90.5	115.15	85.55	0.742943986	5.51457E-06
A1L471	ABCB1	ATP-binding cassette, sub-family B (MDR/T/	1	128.1	104.1	84.3	88.2	116.1	86.25	0.742894057	5.48597E-06
Q9BW85	CCDC94	Coiled-coil domain-containing protein 94 OS	1	132	109.1	88.5	90	120.55	89.25	0.740356698	4.20405E-06
P10243	MYBL1	Myb-related protein A OS=Homo sapiens GN	1	149.7	79.8	97.6	71.7	114.75	84.65	0.737690632	3.16509E-06
Q9NPF4	OSGEP	Probable tRNA N6-adenosine threonylcarbam	1	124.6	112.8	88.8	86.3	118.7	87.55	0.737573715	3.12563E-06
H0YB34	RIDA	Protein RIDA (Fragment) OS=Homo sapiens	3	154.7	89.7	89.5	90.7	122.2	90.1	0.737315876	3.04022E-06
A0A0A0MQS	CLASRP	CLK4-associating serine/arginine-rich protein	1	120.7	107.4	87.2	80.5	114.05	83.85	0.735203858	2.41926E-06
L0R819	ASNSD1	Alternative protein ASNSD1 OS=Homo sapie	1	151.4	90.4	95.2	82.4	120.9	88.8	0.734491315	2.23838E-06
A0A1B0GVL	FAM193A	Protein FAM193A OS=Homo sapiens GN=F/	1	118.1	110.3	78.4	89.3	114.2	83.85	0.734238179	2.17729E-06
A8MYK1	MRPL23	39S ribosomal protein L23, mitochondrial OS	1	150.7	88.3	85.7	89.3	119.5	87.5	0.732217573	1.74313E-06
A0A0A0MTL	SKP2	S-phase kinase-associated protein 2 OS=Hom	2	146.9	92.5	87.9	87.3	119.7	87.6	0.731829574	1.66977E-06
B7Z765		cDNA FLJ56258, highly similar to Squamous	1	140.6	95.6	76.3	95.8	118.1	86.05	0.728619814	1.16571E-06
Q3KRB4	MRPS33	MRPS33 protein (Fragment) OS=Homo sapie	2	146.6	98.5	92.6	85.9	122.55	89.25	0.728274174	1.12103E-06
Q9NW68	BSDC1	BSD domain-containing protein 1 OS=Homo	1	130.4	96.4	85	80.1	113.4	82.55	0.727954145	1.08111E-06
P55197	MLLT10	Protein AF-10 OS=Homo sapiens GN=MLLT	1	126.2	86.7	66.7	88.1	106.45	77.4	0.727101926	9.81291E-07
P0C7U0	ELFN1	Protein ELFN1 OS=Homo sapiens GN=ELFN	2	133.9	102.4	85.6	86.2	118.15	85.9	0.727041896	9.74601E-07
Q53ET9		Ariadne homolog 2 variant (Fragment) OS=H	1	120.9	124.1	85.6	92.4	122.5	89	0.726530612	9.19348E-07
Q8IZY2	ABCA7	ATP-binding cassette sub-family A member 7	1	118.9	113.9	84.8	84.3	116.4	84.55	0.72637457	9.03088E-07
A8K0K1		cDNA FLJ76996 OS=Homo sapiens PE=2 SV	1	139.7	106	91.3	87.1	122.85	89.2	0.726088726	8.74006E-07
Q01850	CDR2	Cerebellar degeneration-related protein 2 OS=	2	129	121.8	95.8	85.8	125.4	90.8	0.724082935	6.93624E-07
B2RA50		cDNA, FLJ94698, highly similar to Homo sap	1	160.4	93.4	86.6	96.9	126.9	91.75	0.723010244	6.12307E-07
Q9NPG8	ZDHHC4	Probable palmitoyltransferase ZDHHC4 OS=	1	110.8	137.5	97.2	82.3	124.15	89.75	0.722915828	6.05602E-07
A0A059QXN6	COX1	Cytochrome c oxidase subunit 1 OS=Homo sa	1	133.2	113.4	100.3	77.9	123.3	89.1	0.722627737	5.85574E-07
Q14142	TRIM14	Tripartite motif-containing protein 14 OS=Ho	1	142	97.6	86.8	86	119.8	86.4	0.721202003	4.95387E-07
A1A4S6	ARHGAP10	Rho GTPase-activating protein 10 OS=Homo	1	128.4	125.4	99	83.2	126.9	91.1	0.717888101	3.3409E-07
F4MHH6	UTY	Ubiquitously transcribed tetratricopeptide rep	1	159.1	95.9	85.3	97.7	127.5	91.5	0.717647059	3.24561E-07
P46531	NOTCH1	Neurogenic locus notch homolog protein 1 OS	2	157.5	87	76.6	98.7	122.25	87.65	0.716973415	2.99285E-07
Q9NYV6	RRN3	RNA polymerase I-specific transcription initia	1	168	78.7	86.8	90	123.35	88.4	0.716659911	2.88172E-07
Q9BUA3	C11orf84	Uncharacterized protein C11orf84 OS=Homo	2	125.7	117.1	88.8	85.1	121.4	86.95	0.716227348	2.73485E-07
A0A024R5P6	C1orf121	Chromosome 1 open reading frame 121, isofo	1	132.3	105.6	91.7	78.4	118.95	85.05	0.715006305	2.35781E-07
Q9HCL0	PCDH18	Protocadherin-18 OS=Homo sapiens GN=PCI	1	155.8	82.4	100	69.5	119.1	84.75	0.711586902	1.54798E-07
P08842	STS	Steryl-sulfatase OS=Homo sapiens GN=STS	1	102.6	104.1	79.8	67	103.35	73.4	0.710208031	1.30346E-07
Q86UU0	BCL9L	B-cell CLL/lymphoma 9-like protein OS=Hor	1	134.5	103.7	85.5	83.2	119.1	84.35	0.70822838	1.01602E-07
A0A024R7B1	ZNF317	Zinc finger protein 317, isoform CRA_b OS=	1	122.9	116.8	84.2	84.4	119.85	84.3	0.703379224	5.45605E-08
Q32NC0	C18orf21	UPF0711 protein C18orf21 OS=Homo sapien	1	131.3	86.3	69.4	82.7	108.8	76.05	0.698988971	3.06335E-08
P16383	GCFC2	GC-rich sequence DNA-binding factor 2 OS=	1	135.2	101.4	81.8	81.1	118.3	81.45	0.688503804	7.29375E-09
A4D150	LOC402468	Similar to Dual specificity protein kinase CLI	1	135.1	112.2	86.6	82.8	123.65	84.7	0.684997978	4.43251E-09
O15530	PDPK1	3-phosphoinositide-dependent protein kinase	2	158.5	87.9	83.7	82.8	123.2	83.25	0.675730519	1.1354E-09
Q53FI8		DNA-directed RNA polymerase III 39 kDa po	2	138.9	108.4	78.5	87.9	123.65	83.2	0.672866963	7.35307E-10
Q5TB80	CEP162	Centrosomal protein of 162 kDa OS=Homo sa	1	50.1	20.9	24.5	23.1	35.5	23.8	0.670422535	5.04844E-10
E7ETY2	TCOF1	Treacle protein OS=Homo sapiens GN=TCOI	1	129.1	104.8	80.8	75.6	116.95	78.2	0.668661821	3.83903E-10
E9PN57	SSSCA1	Sjoegren syndrome/scleroderma autoantigen 1	1	100.4	152.1	76.6	91.6	126.25	84.1	0.666138614	2.58147E-10
Q9NRR4	DROSHA	Ribonuclease 3 OS=Homo sapiens GN=DRO	1	139.4	114.7	72.4	94.6	127.05	83.5	0.657221566	6.08673E-11
B2R9G9		cDNA, FLJ94385, highly similar to Homo sap	1	162.3	88.2	80.9	78.7	125.25	79.8	0.637125749	1.82125E-12
Q86TJ2	TADA2B	Transcriptional adapter 2-beta OS=Homo sap	1	166.4	90.7	83.2	78.5	128.55	80.85	0.628938156	3.92127E-13
A8K0P2		cDNA FLJ76209, highly similar to Homo sap	1	152.5	109.6	90.4	69.6	131.05	80	0.610454025	9.61541E-15

P13647	KRT5	Keratin, type II cytoskeletal 5 OS=Homo sapi	1	196.3	90.7	71.9	101.3	143.5	86.6	0.603484321	2.16826E-15
P84101	SERF2	Small EDRK-rich factor 2 OS=Homo sapiens	2	171.1	103.8	75.9	79.1	137.45	77.5	0.563841397	1.59099E-19
O94898	LRIG2	Leucine-rich repeats and immunoglobulin-like	1	134.6	162.1	73	68.5	148.35	70.75	0.476912706	5.82556E-32
O94985	CLSTN1	Calsyntenin-1 OS=Homo sapiens GN=CLSTN	1	259.8	72.8	62.2	72.9	166.3	67.55	0.406193626	6.79219E-47
Q96DE1		Putative uncharacterized protein (Fragment) C	1	74.8	274.7	67.3	61.4	174.75	64.35	0.368240343	1.78027E-57
B2RAH2		Sodium/hydrogen exchanger OS=Homo sapie	2	90.2	94.3	99.6	121.8	92.25	110.7	1.2	0.000338461
A2TEM8	HLA-B	MHC class I antigen (Fragment) OS=Homo sa	2	94.6	96.6	118.5	110.2	95.6	114.35	1.196129707	0.000421575
Q9BXA5	SUCNR1	Succinate receptor 1 OS=Homo sapiens GN=S	1	93	96.5	113.4	112.9	94.75	113.15	1.194195251	0.000470046
Q07283	TCHH	Trichohyalin OS=Homo sapiens GN=TCHH P	2	83.4	86.6	103	100	85	101.5	1.194117647	0.000472097
Q99536	VAT1	Synaptic vesicle membrane protein VAT-1 ho	10	93.5	92	110.7	110.7	92.75	110.7	1.193530997	0.000487875
Q9NS18	GLRX2	Glutaredoxin-2, mitochondrial OS=Homo sap	1	92.3	99.5	111.8	117.1	95.9	114.45	1.193430657	0.000490625
B2R7F8		Plasminogen OS=Homo sapiens PE=2 SV=1	1	85.7	90	103.3	106.2	87.85	104.75	1.192373364	0.00052049
A0A024R5I7	PITPNM1	Phosphatidylinositol transfer protein, membra	2	87.3	96.2	110.8	107.1	91.75	108.95	1.18746594	0.000683094
F4YU36	HLA-B	MHC class I antigen (Fragment) OS=Homo sa	3	93.1	94	113.9	108	93.55	110.95	1.185996793	0.000740436
A0A0R4J2E6	ELAVL4	ELAV-like protein OS=Homo sapiens GN=EL	1	58.6	133.4	96.4	131.3	96	113.85	1.1859375	0.000742842
Q5T6U8	HMGA1	High mobility group AT-hook 1 OS=Homo sa	1	89.4	100.3	117	107.8	94.85	112.4	1.185028993	0.00078066
Q86Y79	PTRH1	Probable peptidyl-tRNA hydrolase OS=Homo	2	86.8	98.8	105.3	114.1	92.8	109.7	1.182112069	0.000914738
Q16739	UGCG	Ceramide glucosyltransferase OS=Homo sapi	1	92.6	96.2	121.8	101.3	94.4	111.55	1.181673729	0.00093667
Q6ZSJ8	C1orf122	Uncharacterized protein C1orf122 OS=Homo	1	90.7	95.8	100.3	119.2	93.25	109.75	1.1769437	0.00120706
Q9H009	NACA2	Nascent polypeptide-associated complex subu	1	89.9	83.6	87.9	116.2	86.75	102.05	1.176368876	0.00124452
A0A024RAC4	ID3	Inhibitor of DNA binding 3, dominant negativ	1	91.7	87.7	92.2	118.6	89.7	105.4	1.175027871	0.00133619
P08254	MMP3	Stromelysin-1 OS=Homo sapiens GN=MMP3	2	96.1	92.3	112.6	108.7	94.2	110.65	1.17462845	0.00136469
Q9NRX5	SERINC1	Serine incorporator 1 OS=Homo sapiens GN=	1	100.8	86.5	109	110.7	93.65	109.85	1.172984517	0.00148815
Q14126	DSG2	Desmoglein-2 OS=Homo sapiens GN=DSG2	12	98.4	94.2	95.4	130.4	96.3	112.9	1.172377985	0.00153628
Q06033	ITIH3	Inter-alpha-trypsin inhibitor heavy chain H3 C	3	90.5	92.4	102.9	111.4	91.45	107.15	1.171678513	0.0015936
A0A024R9D8	SPAG1	Sperm associated antigen 1, isoform CRA_b C	1	79.9	104.8	100.8	115.6	92.35	108.2	1.17162967	0.00159768
Q5SZJ8	BEND6	BEN domain-containing protein 6 OS=Homo	1	97.2	99.1	109.3	120.5	98.15	114.9	1.170657157	0.0016809
V9HW25	HEL-S-273	Epididymis secretory protein Li 273 OS=Hom	1	102.7	92.4	114.4	113.7	97.55	114.05	1.169144029	0.00181851
P02760	AMBP	Protein AMBP OS=Homo sapiens GN=AMB	2	99.7	81.9	103.6	108.7	90.8	106.15	1.169052863	0.00182713
A8K2G7		cDNA FLJ76071, highly similar to Homo sap	5	92.6	92.9	107.9	108.9	92.75	108.4	1.168733154	0.00185766
A0A024R466	ITM2C	Integral membrane protein 2C, isoform CRA_	1	92	94.6	108.5	109.4	93.3	108.95	1.167738478	0.00195572
K7ENP4	CEP192	Centrosomal protein of 192 kDa (Fragment) C	1	100.1	81.9	90.1	122.2	91	106.15	1.166483516	0.00208633
B3KW93		Sodium/potassium-transporting ATPase subu	2	91.4	99.1	111.8	110.4	95.25	111.1	1.166404199	0.00209485
U3KQB3	CSNK1G1	Casein kinase I isoform gamma-1 OS=Homo	1	94.8	102.2	118.3	111.4	98.5	114.85	1.165989848	0.00213991
B2R687		cDNA, FLJ92839, highly similar to Homo sap	1	78.9	88.1	100.3	94.4	83.5	97.35	1.165868263	0.0021533
O00622	CYR61	Protein CYR61 OS=Homo sapiens GN=CYR	5	100.7	99.3	116	116.7	100	116.35	1.1635	0.00243028
P35527	KRT9	Keratin, type I cytoskeletal 9 OS=Homo sapie	7	91.1	97.3	111.5	107.4	94.2	109.45	1.161889597	0.00263722
P02649	APOE	Apolipoprotein E OS=Homo sapiens GN=AP	4	92.4	90.6	105.4	107.2	91.5	106.3	1.161748634	0.00265608
G3V556	C14orf2	6.8 kDa mitochondrial proteolipid OS=Homo	1	83.3	103.7	104.1	113.1	93.5	108.6	1.161497326	0.00269004
A0A1B0GTY7	TMEM191C	Transmembrane protein 191C (Fragment) OS	1	89.5	93.5	104.5	107.9	91.5	106.2	1.160655738	0.00280674
A0A087WZV9	DTD1	D-tyrosyl-tRNA(Tyr) deacylase (Fragment) C	1	79.2	84.9	95.3	95	82.05	95.15	1.159658745	0.00295106
A0A024RB87	RAP1B	RAP1B, member of RAS oncogene family, is	5	95.8	98.3	114	110.7	97.05	112.35	1.157650696	0.00326294
O15162	PLSCR1	Phospholipid scramblase 1 OS=Homo sapiens	1	99	92.3	107.7	113.7	95.65	110.7	1.157344485	0.0033131
Q9NRU3	CNNM1	Metal transporter CNNM1 OS=Homo sapiens	2	94.3	90.8	101.2	112.7	92.55	106.95	1.155591572	0.00361429
P32456	GBP2	Guanylate-binding protein 2 OS=Homo sapie	5	95.8	94.2	108.7	110.8	95	109.75	1.155263158	0.00367348
A8K9L8		cDNA FLJ76964, highly similar to Homo sap	1	102.1	94.9	122.1	105	98.5	113.55	1.152791878	0.00414845
Q5T7S2	TGFBR1	Receptor protein serine/threonine kinase OS=	1	82.5	84.2	96.2	95.7	83.35	95.95	1.151169766	0.00449043
Q9NXV2	KCTD5	BTB/POZ domain-containing protein KCTD5	2	89.7	93	99.1	111.1	91.35	105.1	1.150519978	0.0046346
Q9BYX7	POTEKP	Putative beta-actin-like protein 3 OS=Homo s	3	91.9	109.1	146.4	84.8	100.5	115.6	1.150248756	0.00469602
O14524	NEMP1	Nuclear envelope integral membrane protein 1	3	92.9	93.2	107	106.9	93.05	106.95	1.149382053	0.00489741
Q6N075	MFSD5	Molybdate-anion transporter OS=Homo sapie	1	84.8	99.6	101.4	110.4	92.2	105.9	1.148590022	0.00508836
A0A024R371	ARL6IP5	PRA1 family protein OS=Homo sapiens GN=	6	96.2	95	111.9	107.7	95.6	109.8	1.148535565	0.00510172
P07686	HEXB	Beta-hexosaminidase subunit beta OS=Homo	11	93.9	96.5	109.8	108.8	95.2	109.3	1.148109244	0.0052076
B2RD00		cDNA, FLJ96396, highly similar to Homo sap	1	86.9	99	100.4	113	92.95	106.7	1.147928994	0.00525296
U3KQB5	TGIF2	Homeobox protein TGIF2 (Fragment) OS=Ho	1	104	95.1	117.2	111.3	99.55	114.25	1.14766449	0.00532016
Q8NDC0	MAPK1IP1L	MAPK-interacting and spindle-stabilizing pro	1	87.5	104.7	108	112.5	96.1	110.25	1.147242456	0.00542909
Q9BSJ8	ESYT1	Extended synaptotagmin-1 OS=Homo sapiens	1	92.5	97.2	99.4	118.2	94.85	108.8	1.147074328	0.00547304
O00560	SDCBP	Syntenin-1 OS=Homo sapiens GN=SDCBP P	8	97.9	96.1	112.4	110.1	97	111.25	1.146907216	0.00551706
P36021	SLC16A2	Monocarboxylate transporter 8 OS=Homo sap	1	96.1	85.7	93.6	114.9	90.9	104.25	1.146864686	0.00552832
B4DY32		cDNA FLJ51387, highly similar to Asparagin	1	92.3	93.9	110.5	103	93.1	106.75	1.146616541	0.00559438
A8K4H5		Chloride channel protein OS=Homo sapiens P	2	98.7	93.2	114.6	105.4	95.95	110	1.146430433	0.00564441
Q13137	CALCOCO2	Calcium-binding and coiled-coil domain-cont	4	87.1	81.7	95.8	97.5	84.4	96.65	1.14514218	0.00600225
L0R5C4	POTEM	Alternative protein POTEM OS=Homo sapien	1	95.3	97.4	106.6	113.9	96.35	110.25	1.144265698	0.00625751
Q8NCM2	KCNH5	Potassium voltage-gated channel subfamily H	1	84.1	81.7	92.2	97.5	82.9	94.85	1.144149578	0.00629206
A0A140VJX1		Testicular tissue protein Li 198 OS=Homo sap	18	94.5	94.4	108	108.1	94.45	108.05	1.14399153	0.00633934
Q12980	NPRL3	Nitrogen permease regulator 3-like protein OS	1	96.6	91.5	104.7	110.1	94.05	107.4	1.141945774	0.00698198
P25940	COL5A3	Collagen alpha-3(V) chain OS=Homo sapiens	1	92.8	101.7	113.9	108.2	97.25	111.05	1.141902314	0.00699623
Q9NUE0	ZDHHC18	Palmitoyltransferase ZDHHC18 OS=Homo sa	1	92.6	95.3	107.4	107.1	93.95	107.25	1.141564662	0.00710809
Q13319	CDK5R2	Cyclin-dependent kinase 5 activator 2 OS=Ho	1	95.6	100.6	114.2	109.7	98.1	111.95	1.141182467	0.00723666
Q6NZ59	ATP5J	ATP synthase-coupling factor 6, mitochondria	1	91.7	89	106.1	100.1	90.35	103.1	1.141117875	0.00725856
A0A024R3E3	APOA1	Apolipoprotein A-I, isoform CRA_a OS=Hom	2	101.1	90.7	114.5	104.2	95.9	109.35	1.140250261	0.00755924
Q9H813	TMEM206	Transmembrane protein 206 OS=Homo sapien	3	94.4	99.1	105.9	114.7	96.75	110.3	1.14005168	0.0076296
Q5T0D9	TPRG1L	Tumor protein p63-regulated gene 1-like prote	1	86.1	87.2	100.9	96.6	86.65	98.75	1.139642239	0.00777665
B4DHZ6	TF	Transferrin, isoform CRA_c OS=Homo sapien	1	92.3	94.5	111.1	101.7	93.4	106.4	1.139186296	0.00794345
A0A024RDZ1	RP11-484I6.3	Uncharacterized protein OS=Homo sapiens G	1	103.9	92.6	101	122.7	98.25	111.85	1.138422392	0.00823023
L8E847	SNAP29	Alternative protein SNAP29 OS=Homo sapie	1	86.6	102.9	108.7	106.9	94.75	107.8	1.137730871	0.00849798
Q59FN8		Adaptor-related protein complex 2, alpha 2 su	1	100.3	90.8	120	97.4	95.55	108.7	1.13762428	0.00853994
A0A0A0MTL(	FAM65A	Protein FAM65A OS=Homo sapiens GN=FA	1	93	94.7	95.3	118.1	93.85	106.7	1.136920618	0.0088218
O95562	SFT2D2	Vesicle transport protein SFT2B OS=Homo sa	3	87.1	111.7	117.6	108.3	99.4	112.95	1.136317907	0.00906993
A8K5D4	MPZL1	Myelin protein zero-like 1, isoform CRA_b O	4	97.4	97.7	110.1	111.5	97.55	110.8	1.135827781	0.00927636
B2RA34		cDNA, FLJ94678, highly similar to Homo sap	1	88.9	102.7	109.4	108.2	95.8	108.8	1.135699374	0.00933115
A0A024R472	TMBIM1	Transmembrane BAX inhibitor motif containi	1	91.9	88.1	108.8	95.6	90	102.2	1.135555556	0.00939289
E9PFZ2	CP	Ceruloplasmin OS=Homo sapiens GN=CP PE	5	94.5	89.2	103.8	104.8	91.85	104.3	1.135547088	0.00939654
Q9UNN8	PROCR	Endothelial protein C receptor OS=Homo sap	1	99.5	90.2	105	110.4	94.85	107.7	1.135477069	0.00942671
A8K3K1		cDNA FLJ78096, highly similar to Homo sap	5	98.4	97.3	108.5	113.7	97.85	111.1	1.135411344	0.00945512
Q9UK58	CCNL1	Cyclin-L1 OS=Homo sapiens GN=CCNL1 PE	4	95.8	94.5	105.8	110.2	95.15	108	1.135049921	0.00961282
Q9NRZ7	AGPAT3	1-acyl-sn-glycerol-3-phosphate acyltransferas	1	87.3	91.2	102	100.6	89.25	101.3	1.135014006	0.0096286
Q5BJF6	ODF2	Outer dense fiber protein 2 OS=Homo sapiens	1	74.5	66.9	70.7	89.7	70.7	80.2	1.13437058	0.00991573
P41250	GARS	Glycine--tRNA ligase OS=Homo sapiens GN	1	90.8	99	101.7	113.6	94.9	107.65	1.134351949	0.00992415
Q8TAG9	EXOC6	Exocyst complex component 6 OS=Homo sap	1	90	105.6	100.9	120.9	97.8	110.9	1.13394683	0.0101091
Q9UIW2	PLXNA1	Plexin-A1 OS=Homo sapiens GN=PLXNA1 P	1	91.5	97	102.1	111.6	94.25	106.85	1.133687003	0.0102293
P16104	H2AFX	Histone H2AX OS=Homo sapiens GN=H2AF	1	85	100.6	106.8	103.5	92.8	105.15	1.133081897	0.0105143
P08243	ASNS	Asparagine synthetase [glutamine-hydrolyzing	1	98.9	94.8	117	102.4	96.85	109.7	1.132679401	0.0107079
Q04912	MST1R	Macrophage-stimulating protein receptor OS=	1	100.1	78.4	96.4	105.6	89.25	101	1.131652661	0.0112164
P08697	SERPINF2	Alpha-2-antiplasmin OS=Homo sapiens GN=	1	97.2	95.3	114.8	103	96.25	108.9	1.131428571	0.0113303
V9HWB4	HEL-S-89n	Epididymis secretory sperm binding protein L	38	95.8	96	108.3	108.7	95.9	108.5	1.131386861	0.0113516
Q8IXS2	CCDC65	Coiled-coil domain-containing protein 65 OS=	1	58.4	63.5	57	80.9	60.95	68.95	1.131255127	0.0114191
Q6NS36	FTH1	Ferritin (Fragment) OS=Homo sapiens GN=F	9	101.5	93.8	111.8	109.1	97.65	110.45	1.131080389	0.0115093
O15455	TLR3	Toll-like receptor 3 OS=Homo sapiens GN=T	1	92	102.5	109	110.9	97.25	109.95	1.13059126	0.0117651
Q5T7N0	RPL5	60S ribosomal protein L5 (Fragment) OS=Ho	1	79.6	106.2	105.6	104.4	92.9	105	1.130247578	0.0119479
Q5VST6	ABHD17B	Protein ABHD17B OS=Homo sapiens GN=A	2	98	94.2	104.5	112.7	96.1	108.6	1.130072841	0.0120419
A0A158RFU6	RAB7A	RAB7, member RAS oncogene family, isofor	18	96	95.3	108.7	107.4	95.65	108.05	1.12963931	0.0122778
P12814	ACTN1	Alpha-actinin-1 OS=Homo sapiens GN=ACT	33	99.6	98.1	111.7	111.6	98.85	111.65	1.129489125	0.0123605
B2R5B3		Histone H2A OS=Homo sapiens PE=2 SV=1	3	79.5	102.3	96	109.3	90.9	102.65	1.129262926	0.012486
P0CG38	POTEI	POTE ankyrin domain family member I OS=H	1	101.3	110.4	152	87	105.85	119.5	1.12895607	0.0126581
Q6IPH7	RPL14	RPL14 protein OS=Homo sapiens GN=RPL1	1	87.4	104.3	107.6	108.7	95.85	108.15	1.128325509	0.0130186
Q9Y3D5	MRPS18C	28S ribosomal protein S18c, mitochondrial OS	1	93.8	98.7	102.7	114.5	96.25	108.6	1.128311688	0.0130266
A0A0C4DG49	PVR	Poliovirus receptor OS=Homo sapiens GN=P	3	96	99.1	109.3	110.7	97.55	110	1.127626858	0.0134288
P49748	ACADVL	Very long-chain specific acyl-CoA dehydroge	32	95.8	93.9	107.7	106.2	94.85	106.95	1.127569847	0.0134628
B2RE36		cDNA, FLJ96903 OS=Homo sapiens PE=2 SV	2	97.1	95.1	96.2	120.5	96.1	108.35	1.127471384	0.0135217
F8VW96	CSRP2	Cysteine and glycine-rich protein 2 OS=Homo	4	94.7	100.3	109.7	110	97.5	109.85	1.126666667	0.0140116
A0A024R8V5	hCG_1642326	HCG1642326, isoform CRA_b OS=Homo sap	1	94.7	95.9	102.2	112.5	95.3	107.35	1.126442812	0.0141507
A0A024RAF7	ECE1	Endothelin converting enzyme 1, isoform CR	3	94.6	100	108	111.2	97.3	109.6	1.126413155	0.0141692
A0A024RB05	CD63	Tetraspanin OS=Homo sapiens GN=CD63 PE	3	100.5	93.2	114.9	103.2	96.85	109.05	1.125967992	0.0144499
Q16822	PCK2	Phosphoenolpyruvate carboxykinase [GTP], m	22	96.2	93	104.4	108.3	94.6	106.35	1.124207188	0.01561
A0A059RVX4	COX1	Cytochrome c oxidase subunit 1 OS=Homo sa	1	101.9	86.5	106.2	105.6	94.2	105.9	1.124203822	0.0156122
A8K8D2		cDNA FLJ77371, highly similar to Homo sap	1	88.2	87.4	104.7	92.7	87.8	98.7	1.124145786	0.0156519
J3QTA2	BAG1	BAG family molecular chaperone regulator 1	3	90.8	100.1	112.7	101.8	95.45	107.25	1.123624935	0.0160116
P31751	AKT2	RAC-beta serine/threonine-protein kinase OS	1	84.9	105.7	108.7	105.4	95.3	107.05	1.123294858	0.0162434
Q9ULH1	ASAP1	Arf-GAP with SH3 domain, ANK repeat and	1	100.4	99.3	124.4	99.8	99.85	112.1	1.122684026	0.0166803
A0A024QYW	PLP2	Proteolipid protein 2 (Colonic epithelium-enri	2	89.8	102.9	104.2	112.1	96.35	108.15	1.122470161	0.0168358
Q8NC42	RNF149	E3 ubiquitin-protein ligase RNF149 OS=Hom	2	89.2	98.3	98.8	111.6	93.75	105.2	1.122133333	0.0170832

P18085	ARF4	ADP-ribosylation factor 4 OS=Homo sapiens	5	97.7	95.1	108.5	107.7	96.4	108.1	1.121369295	0.0176568
A0A024RC97	PTDSS2	Phosphatidylserine synthase 2, isoform CRA_	2	95.2	94.4	107.7	104.9	94.8	106.3	1.121308017	0.0177035
P11279	LAMP1	Lysosome-associated membrane glycoprotein	4	95.9	96.6	110.6	105.2	96.25	107.9	1.121038961	0.0179101
Q70CQ2	USP34	Ubiquitin carboxyl-terminal hydrolase 34 OS=	2	89.9	100.4	101.3	112	95.15	106.65	1.120861797	0.0180472
Q3MHU6	NDUFB1	NDUFB1 protein (Fragment) OS=Homo sapie	1	96.7	91.3	102.6	108	94	105.3	1.120212766	0.0185578
Q96BM9	ARL8A	ADP-ribosylation factor-like protein 8A OS=H	3	95.1	97.1	110.5	104.8	96.1	107.65	1.120187305	0.0185781
Q9HC85	MB2	Metastasis related protein (Fragment) OS=Ho	3	103	93.6	114.3	105.9	98.3	110.1	1.120040692	0.0186953
Q08722	CD47	Leukocyte surface antigen CD47 OS=Homo s	3	96.6	97.8	107.4	110.3	97.2	108.85	1.119855967	0.0188439
A0A024R1M8	APOL2	Apolipoprotein L, 2, isoform CRA_a OS=Hon	9	95.9	96.9	108.6	107.3	96.4	107.95	1.119813278	0.0188784
P10606	COX5B	Cytochrome c oxidase subunit 5B, mitochond	7	92.7	100.3	113	103.1	96.5	108.05	1.119689119	0.0189792
A0A024R292	FVT1	Follicular lymphoma variant translocation 1, i	1	98	92.5	100.5	112.8	95.25	106.65	1.119685039	0.0189824
B2R7U4		cDNA, FLJ93605, highly similar to Homo sap	13	93.9	92.5	104.6	104.1	93.2	104.35	1.119635193	0.019023
Q86V37	TAOK2	TAOK2 protein (Fragment) OS=Homo sapien	1	92.9	89.6	99.2	105.1	91.25	102.15	1.119452055	0.0191727
B5BUG9	BMPR1A	Receptor protein serine/threonine kinase (Frag	4	104.4	93.2	113.8	107.4	98.8	110.6	1.119433198	0.0191882
P17301	ITGA2	Integrin alpha-2 OS=Homo sapiens GN=ITGA	1	100.4	99.8	113	111.1	100.1	112.05	1.119380619	0.0192314
A0A1B0GTQ2	RAB34	Ras-related protein Rab-34, isoform NARR O	1	95.1	95.2	104.2	108.5	95.15	106.35	1.117708881	0.020651
A5A3E0	POTEF	POTE ankyrin domain family member F OS=	1	109.8	98.4	124.5	108.2	104.1	116.35	1.117675312	0.0206804
D3DR65	SPFH1	SPFH domain family, member 1, isoform CRA	6	97.4	98.3	108	110.7	97.85	109.35	1.117526827	0.0208112
Q68CP4	HGSNAT	Heparan-alpha-glucosaminide N-acetyltransfe	1	92	95.7	99.4	110.3	93.85	104.85	1.117208311	0.0210939
Q9Y2B2	PIGL	N-acetylglucosaminyl-phosphatidylinositol de	1	88.8	89.1	99.2	99.5	88.95	99.35	1.116919618	0.0213532
Q9C040	TRIM2	Tripartite motif-containing protein 2 OS=Hom	1	90.7	95.3	99.7	108	93	103.85	1.116666667	0.0215827
Q8WUA4	GTF3C2	General transcription factor 3C polypeptide 2	1	91.2	101.9	110.3	105.2	96.55	107.75	1.116002071	0.0221962
P28065	PSMB9	Proteasome subunit beta type-9 OS=Homo sa	2	92.3	94.9	106.2	102.6	93.6	104.4	1.115384615	0.0227802
P49006	MARCKSL1	MARCKS-related protein OS=Homo sapiens	4	98.5	98.3	114.4	105.1	98.4	109.75	1.115345528	0.0228176
Q9Y241	HIGD1A	HIG1 domain family member 1A, mitochondr	3	86	105.2	101	112.2	95.6	106.6	1.115062762	0.0230899
A0A024R5H0	BANF1	Barrier to autointegration factor 1, isoform CR	3	89	98	102.4	106	93.5	104.2	1.114438503	0.0237013
Q6QNY1	BLOC1S2	Biogenesis of lysosome-related organelles cor	1	101.6	93.3	92	125.2	97.45	108.6	1.11441765	0.023722
P61927	RPL37	60S ribosomal protein L37 OS=Homo sapiens	1	75.6	112.3	100.2	109	93.95	104.6	1.113358169	0.0247937
P24844	MYL9	Myosin regulatory light polypeptide 9 OS=Ho	4	95.8	98.3	108.6	107.5	97.05	108.05	1.113343637	0.0248087
A8K132		cDNA FLJ75476, highly similar to Homo sap	9	96.9	96.8	108.5	107.1	96.85	107.8	1.113061435	0.0251014
Q13813	SPTAN1	Spectrin alpha chain, non-erythrocytic 1 OS=I	1	97.6	100	109.8	110.1	98.8	109.95	1.112854251	0.0253183
A6NCE7	MAP1LC3B2	Microtubule-associated proteins 1A/1B light c	3	99.4	87.1	106.7	100.8	93.25	103.75	1.112600536	0.0255861
A0A024RA85	CDC2L5	Cell division cycle 2-like 5 (Cholinesterase-re	1	92.5	86.9	102.6	96.9	89.7	99.75	1.112040134	0.0261866
Q6UWY0	ARSK	Arylsulfatase K OS=Homo sapiens GN=ARS	1	96	103.1	110.8	110.6	99.55	110.7	1.112004018	0.0262257
P06213	INSR	Insulin receptor OS=Homo sapiens GN=INSF	1	90.3	100.3	111.7	100.2	95.3	105.95	1.111752361	0.0264998
Q9Y5B8	NME7	Nucleoside diphosphate kinase 7 OS=Homo s	1	97.1	92.7	98.5	112.5	94.9	105.5	1.111696523	0.026561
O43818	RRP9	U3 small nucleolar RNA-interacting protein 2	11	94	94.3	106.2	103.1	94.15	104.65	1.111524164	0.0267506
Q59G70		Mannosyl (Alpha-1,3-)-glycoprotein beta-1,2-	2	90.5	99.7	102.6	108.8	95.1	105.7	1.111461619	0.0268196
A0A126GWB4	OR2L3	Olfactory receptor OS=Homo sapiens GN=OI	1	61.1	122.1	100.3	103.3	91.6	101.8	1.111353712	0.0269391
D3DUL8	KIAA1609	KIAA1609 protein, isoform CRA_a OS=Hom	1	96.3	96	104.7	109	96.15	106.85	1.111284451	0.0270161
A0A140VKA2	CLDN20	Claudin OS=Homo sapiens GN=CLDN20 PE	1	95.4	85.9	96.4	105	90.65	100.7	1.110865968	0.0274855
H3BP20	HEXA	Beta-hexosaminidase OS=Homo sapiens GN=	1	97.6	94.1	110.3	102.6	95.85	106.45	1.110589463	0.0277995
Q07817	BCL2L1	Bcl-2-like protein 1 OS=Homo sapiens GN=E	2	96.3	98.5	110.5	105.8	97.4	108.15	1.11036961	0.0280516
Q9BUV8	C20orf24	Uncharacterized protein C20orf24 OS=Homo	1	95.7	99.2	100	116.4	97.45	108.2	1.110312981	0.0281168
A0A059QB80	ATP6	ATP synthase subunit a OS=Homo sapiens GI	2	93.2	93.1	105.3	101.5	93.15	103.4	1.110037574	0.0284359
P53794	SLC5A3	Sodium/myo-inositol cotransporter OS=Homo	4	99.2	96.4	109.8	107.3	97.8	108.55	1.1099182	0.0285751
A0A0S2Z4Z6	SRRM1	Serine/arginine repetitive matrix 1 isoform 2 (	8	90.9	101.2	106.8	106.4	96.05	106.6	1.109838626	0.0286684
Q9NX12		cDNA FLJ20496 fis, clone KAT08729 OS=H	3	100.5	92.3	103.3	110.6	96.4	106.95	1.109439834	0.0291395
O14686	KMT2D	Histone-lysine N-methyltransferase 2D OS=H	1	88.4	94.8	105.9	97.3	91.6	101.6	1.109170306	0.0294618
P54652	HSPA2	Heat shock-related 70 kDa protein 2 OS=Hom	2	83.8	95.3	93.1	105.5	89.55	99.3	1.108877722	0.0298151
A0A059QFD5	COX2	Cytochrome c oxidase subunit 2 OS=Homo sa	1	101.4	86.9	104.4	104.4	94.15	104.4	1.108868826	0.029826
Q9NU23	LYRM2	LYR motif-containing protein 2 OS=Homo sa	1	97.8	91.9	107.3	102.8	94.85	105.05	1.107538218	0.0314813
P48060	GLIPR1	Glioma pathogenesis-related protein 1 OS=Ho	1	91.4	104	108.1	108.3	97.7	108.2	1.107471853	0.031566
Q8NC44	FAM134A	Protein FAM134A OS=Homo sapiens GN=FA	1	97.7	92.3	102.1	108.3	95	105.2	1.107368421	0.0316982
Q9P2C4	TMEM181	Transmembrane protein 181 OS=Homo sapier	1	97.7	96.5	101.6	113.4	97.1	107.5	1.107106076	0.0320361
K7PPU4	TP53	Cellular tumor antigen p53 OS=Homo sapiens	2	106.2	95.4	112.3	110.7	100.8	111.5	1.106150794	0.033293
Q5VW32	BROX	BRO1 domain-containing protein BROX OS=	2	95.8	93.6	100.1	109.4	94.7	104.75	1.106124604	0.033328
A0A087X0M0	R3HCC1L	Coiled-coil domain-containing protein R3HC	1	92.2	97.4	103.9	105.8	94.8	104.85	1.106012658	0.0334782
P57088	TMEM33	Transmembrane protein 33 OS=Homo sapiens	8	93.5	97	101.1	109.5	95.25	105.3	1.105511811	0.0341577
Q9Y487	ATP6V0A2	V-type proton ATPase 116 kDa subunit a isof	4	97.1	95.7	104.3	108.8	96.4	106.55	1.105290456	0.0344618
A0A1B0GW05	DPY19L1	Probable C-mannosyltransferase DPY19L1 O	3	94.5	93.6	102.9	105	94.05	103.95	1.105263158	0.0344995
A0A0A6YYL4	CORO7-PAM1	Coronin OS=Homo sapiens GN=CORO7-PA	6	90.7	103.3	97.5	116.9	97	107.2	1.105154639	0.0346495
Q92688	ANP32B	Acidic leucine-rich nuclear phosphoprotein 32	3	89.6	103.5	105.8	107.6	96.55	106.7	1.105126877	0.0346881
D6RB85	CANX	Calnexin (Fragment) OS=Homo sapiens GN=	1	98.1	97.4	115.8	100.2	97.75	108	1.104859335	0.0350609
A8K602		Mitogen-activated protein kinase kinase kinas	1	98	97.7	104.4	111.8	97.85	108.1	1.104752172	0.0352112
G3XAI2	LAMB1	Laminin subunit beta-1 OS=Homo sapiens GI	21	96.4	95.8	105.6	106.7	96.1	106.15	1.104578564	0.0354559
Q15043	SLC39A14	Zinc transporter ZIP14 OS=Homo sapiens GN	1	92.5	105.5	111.5	107.1	99	109.3	1.104040404	0.0362242
Q96ET8	TVP23C	Golgi apparatus membrane protein TVP23 ho	2	92.1	92.9	99.4	104.7	92.5	102.05	1.103243243	0.0373887
A8K6Q8		cDNA FLJ75881, highly similar to Homo sap	1	101.1	99.4	114.8	106.4	100.25	110.6	1.103241895	0.0373907
P15954	COX7C	Cytochrome c oxidase subunit 7C, mitochond	2	98.3	93.5	109.4	102.2	95.9	105.8	1.103232534	0.0374046
Q12974	PTP4A2	Protein tyrosine phosphatase type IVA 2 OS=	3	100.5	95.2	109.9	106	97.85	107.95	1.103219213	0.0374242
P01023	A2M	Alpha-2-macroglobulin OS=Homo sapiens GI	1	94.4	94.8	103.1	105.6	94.6	104.35	1.103065539	0.0376528
P07305	H1F0	Histone H1.0 OS=Homo sapiens GN=H1F0 P	2	99.9	94.9	110.2	104.6	97.4	107.4	1.102669405	0.0382473
Q7Z5H4	VN1R5	Vomeronasal type-1 receptor 5 OS=Homo sap	1	104.2	106.4	122.9	109.3	105.3	116.1	1.102564103	0.0384066
O43759	SYNGR1	Synaptogyrin-1 OS=Homo sapiens GN=SYN(	1	93.7	93.7	100.4	106.1	93.7	103.25	1.101921025	0.0393925
O15431	SLC31A1	High affinity copper uptake protein 1 OS=Hon	2	89	100.9	108.7	100.5	94.95	104.6	1.101632438	0.0398422
E5KBQ3	TRAF2	TRAF2 OS=Homo sapiens GN=TRAF2 PE=2	3	88.9	95.4	94.4	108.6	92.15	101.5	1.101465003	0.0401052
P60604	UBE2G2	Ubiquitin-conjugating enzyme E2 G2 OS=Ho	3	96.4	97.9	108	106	97.15	107	1.101389604	0.040224
Q9BS26	ERP44	Endoplasmic reticulum resident protein 44 OS	16	96.6	97	107.6	105.6	96.8	106.6	1.101239669	0.0404613
Q17R23	STON2	Stonin 2 OS=Homo sapiens GN=STON2 PE=	1	95.5	91.6	101.3	104.7	93.55	103	1.1010155	0.0408185
P50579	METAP2	Methionine aminopeptidase 2 OS=Homo sapi	12	95.7	92.8	100.9	106.6	94.25	103.75	1.100795756	0.0411712
Q59GX9		Ribosomal protein L5 variant (Fragment) OS=	1	98	94.5	106.7	105.2	96.25	105.95	1.100779221	0.0411979
Q8NEZ4	KMT2C	Histone-lysine N-methyltransferase 2C OS=H	4	99.7	97.9	111	106.5	98.8	108.75	1.100708502	0.041312
A0A024RB50	B4GALNT1	Beta-1,4 N-acetylgalactosaminyltransferase O	1	97.9	94.4	90.1	121.5	96.15	105.8	1.100364015	0.0418719
C6KMR8	FOXC1	Forkhead box C1 OS=Homo sapiens GN=FO	1	85	106.4	96.3	114.3	95.7	105.3	1.10031348	0.0419547
Q99808	SLC29A1	Equilibrative nucleoside transporter 1 OS=Ho	4	94	99.7	107	106.1	96.85	106.55	1.100154879	0.042215
G5D8C8	COX2	Cytochrome c oxidase subunit 2 OS=Homo sa	1	94.1	98.7	101.5	110.6	96.4	106.05	1.100103734	0.0422993
Q9H3U5	MFSD1	Major facilitator superfamily domain-containi	1	107.2	82.9	104.1	105	95.05	104.55	1.099947396	0.0425578
Q9UI26	IPO11	Importin-11 OS=Homo sapiens GN=IPO11 P	1	92.6	110.8	125.1	98.6	101.7	111.85	1.099803343	0.0427973
A0A0J9YXF2	PON2	Paraoxonase 2, isoform CRA_a OS=Homo sa	7	93.9	97.7	109	101.7	95.8	105.35	1.099686848	0.0429918
B4E2S7		cDNA FLJ58780, highly similar to Homo sap	7	99.4	95.3	107.9	106.2	97.35	107.05	1.099640473	0.0430694
Q9BQ69	MACROD1	O-acetyl-ADP-ribose deacetylase MACROD1	1	100	89.8	105.3	103.4	94.9	104.35	1.099578504	0.0431734
Q9NZ23	YA61	Drug-sensitive protein 1 OS=Homo sapiens G	9	102.7	99.5	114.4	107.9	101.1	111.15	1.099406528	0.0434631
B4DK98		cDNA FLJ53535 OS=Homo sapiens PE=2 SV	1	94.6	99.3	103.8	109.3	96.95	106.55	1.099020113	0.0441198
Q53GG0		Epithelial protein lost in neoplasm beta varian	8	101.6	96.7	110	107.9	99.15	108.95	1.098840141	0.0444287
B2R533		cDNA, FLJ92320, highly similar to Homo sap	1	93.2	95	104.7	102.1	94.1	103.4	1.098831031	0.0444443
Q9NPJ3	ACOT13	Acyl-coenzyme A thioesterase 13 OS=Homo	3	95.8	97.5	109.9	102.5	96.65	106.2	1.09881014	0.0444802
Q08623	PUDP	Pseudouridine-5'-phosphatase OS=Homo sapi	1	87.4	93	102.7	95.5	90.2	99.1	1.098669623	0.044723
A0A0A0MR3	MYEF2	Myelin expression factor 2 OS=Homo sapiens	5	92.6	99.2	105.3	105.4	95.9	105.35	1.098540146	0.0449476
A8K2Q6		Peptidyl-prolyl cis-trans isomerase OS=Homo	4	96.2	98.3	104	109.6	97.25	106.8	1.098200514	0.0455415
Q9H0Z9	RBM38	RNA-binding protein 38 OS=Homo sapiens C	1	93.9	100.1	106.2	106.8	97	106.5	1.097938144	0.0460049
A0A087WVP4	SMTN	Smoothelin OS=Homo sapiens GN=SMTN P	2	102.2	90.9	101.6	110.4	96.55	106	1.097876748	0.046114
A8K6F4		cDNA FLJ77991, highly similar to Homo sap	1	93.7	95.5	107.3	100.4	94.6	103.85	1.097780127	0.0462861
Q59G88		Coronin (Fragment) OS=Homo sapiens PE=3	1	94.2	94.2	102	104.8	94.2	103.4	1.097664544	0.0464925
A0A024R2S0	TMEM16K	Anoctamin OS=Homo sapiens GN=TMEM16	2	93.7	93.8	100.4	105.4	93.75	102.9	1.0976	0.0466083
B4DN39		cDNA FLJ53065, highly similar to T-complex	1	78	96.2	93	98.2	87.1	95.6	1.097588978	0.046628
Q8N3C0	ASCC3	Activating signal cointegrator 1 complex subu	2	96.5	94.1	99.4	109.8	95.3	104.6	1.097586569	0.0466325
Q8TC07	TBC1D15	TBC1 domain family member 15 OS=Homo s	3	100.8	94.3	101.3	112.8	97.55	107.05	1.097385956	0.0469936
Q9H8G2	CAAP1	Caspase activity and apoptosis inhibitor 1 OS	2	95.8	94.2	98.9	109.6	95	104.25	1.097368421	0.0470253
O60825	PFKFB2	6-phosphofructo-2-kinase/fructose-2,6-bispho	2	97.4	95.7	116	95.9	96.55	105.95	1.097358881	0.0470428
Q59GF1		Anion exchange protein (Fragment) OS=Hom	18	97.6	96.9	108	105.4	97.25	106.7	1.097172237	0.0473813
F4MH42	UTY	Ubiquitously transcribed tetratricopeptide rep	2	91.7	93.8	90	113.5	92.75	101.75	1.09703504	0.0476315
O94916	NFAT5	Nuclear factor of activated T-cells 5 OS=Hom	2	90.4	92.3	96.3	104	91.35	100.15	1.096332786	0.0489305
P06881	CALCA	Calcitonin gene-related peptide 1 OS=Homo s	1	90.4	105.2	99.5	114.9	97.8	107.2	1.096114519	0.0493404
D3DQ48	TMEM59	Transmembrane protein 59, isoform CRA_e C	2	97.4	99.5	109.5	106.3	98.45	107.9	1.095987811	0.0495797
Q96AD6	GRK6	Protein-serine/threonine kinase OS=Homo sap	2	100.6	93.3	88.7	123.8	96.95	106.25	1.095925735	0.0496974
Q96GX5	MASTL	Serine/threonine-protein kinase greatwall OS=	1	113.8	99.3	94.2	91.3	106.55	92.75	0.870483341	0.049637
F5H1S1	PIGB	Mannosyltransferase OS=Homo sapiens GN=	1	107.2	107.2	91.4	95.2	107.2	93.3	0.870335821	0.0493171
O95822	MLYCD	Malonyl-CoA decarboxylase, mitochondrial C	2	115.6	97.5	90.1	95.3	106.55	92.7	0.870014078	0.0486251
B3KY15		cDNA FLJ46595 fis, clone THYMU3045704	1	115.2	102.5	101.9	87.5	108.85	94.7	0.870004593	0.0486049
Q9UIC8	LCMT1	Leucine carboxyl methyltransferase 1 OS=Ho	4	115.6	100.2	90.3	97.4	107.9	93.85	0.86978684	0.0481413
Q9Y547	HSPB11	Intraflagellar transport protein 25 homolog OS	2	110.8	104.2	98.8	88.2	107.5	93.5	0.869767442	0.0481001

H0Y6V6	LMBR1	Limb region 1 protein homolog (Fragment) O	3	118.7	97	90.9	96.7	107.85	93.8	0.869726472	0.0480134
Q14353	GAMT	Guanidinoacetate N-methyltransferase OS=H	3	105.3	102.6	90.6	90.2	103.95	90.4	0.86964887	0.0478493
H7BY36	EWSR1	RNA-binding protein EWS (Fragment) OS=H	1	115.9	98.6	89	97.5	107.25	93.25	0.869463869	0.0474602
Q96D15	RCN3	Reticulocalbin-3 OS=Homo sapiens GN=RCN	1	114.2	107.2	94.3	98.1	110.7	96.2	0.869015357	0.0465274
A8K9A5		cDNA FLJ78114, highly similar to Homo sap	7	110.3	104.7	93.4	93.4	107.5	93.4	0.868837209	0.0461612
Q8N108	MIER1	Mesoderm induction early response protein 1	1	115.9	91.4	87.1	93	103.65	90.05	0.868789194	0.0460628
A0A024R5D2	MEN1	Multiple endocrine neoplasia I, isoform CRA_	2	118.7	108.2	104.2	92.9	113.45	98.55	0.86866461	0.0458085
A2RU67	FAM234B	Protein FAM234B OS=Homo sapiens GN=FA	1	117.3	91.7	87.5	94	104.5	90.75	0.868421053	0.0453146
P32519	ELF1	ETS-related transcription factor Elf-1 OS=Ho	1	118	99.8	89.6	99.5	108.9	94.55	0.868227732	0.0449257
P42684	ABL2	Abelson tyrosine-protein kinase 2 OS=Homo	1	114.6	100.9	91.2	95.9	107.75	93.55	0.868213457	0.0448971
A0A024R4S5	ZNF444	Zinc finger protein 444, isoform CRA_b OS=	1	136.1	78.5	83.6	102.7	107.3	93.15	0.868126747	0.0447236
Q7Z5L9	IRF2BP2	Interferon regulatory factor 2-binding protein	2	116.4	103.4	99.8	91	109.9	95.4	0.868061874	0.0445943
Q9BQ24	ZFYVE21	Zinc finger FYVE domain-containing protein	1	116	99	92.2	94.4	107.5	93.3	0.867906977	0.0442865
P48454	PPP3CC	Serine/threonine-protein phosphatase 2B catal	2	105.8	105.1	91.2	91.8	105.45	91.5	0.867709815	0.0438972
L0R6M0	PCDHGB3	Alternative protein PCDHGB3 OS=Homo sap	1	113.8	110.6	104.3	90.3	112.2	97.3	0.867201426	0.0429065
Q96S15	WDR24	WD repeat-containing protein 24 OS=Homo s	1	88.5	84.5	66.1	83.9	86.5	75	0.867052023	0.0426188
Q8TCD5	NT5C	5'(3')-deoxyribonucleotidase, cytosolic type O	3	110.8	102	95.1	89.4	106.4	92.25	0.867011278	0.0425406
Q9Y2L1	DIS3	Exosome complex exonuclease RRP44 OS=H	15	109.8	111	96.1	95.3	110.4	95.7	0.866847826	0.0422282
Q96RV3	PCNX1	Pecanex-like protein 1 OS=Homo sapiens GN	1	115.5	106.5	91.2	101.2	111	96.2	0.866666667	0.0418842
Q16186	ADRM1	Proteasomal ubiquitin receptor ADRM1 OS=l	5	124.7	97.1	96.6	95.6	110.9	96.1	0.866546438	0.0416572
Q96BJ3	AIDA	Axin interactor, dorsalization-associated prote	2	115.1	96.1	89.9	93.1	105.6	91.5	0.866477273	0.0415269
Q4KMP7	TBC1D10B	TBC1 domain family member 10B OS=Homo	3	112.2	107.8	95.7	94.9	110	95.3	0.866363636	0.0413139
A0A0C4DFR8	UXT	Protein UXT OS=Homo sapiens GN=UXT PE	1	108.5	99.2	86	93.9	103.85	89.95	0.866153105	0.0409214
Q8NC96	NECAP1	Adaptin ear-binding coat-associated protein 1	4	117.1	95.7	87.6	96.7	106.4	92.15	0.866071429	0.0407699
Q9Y3S1	WNK2	Serine/threonine-protein kinase WNK2 OS=H	1	112.7	100	96.5	87.7	106.35	92.1	0.866008463	0.0406535
Q6ZMW8		cDNA FLJ16624 fis, clone TESTI4015442, m	1	101.2	107.8	106.4	74.5	104.5	90.45	0.865550239	0.0398143
Q5SRH9	TTC39A	Tetratricopeptide repeat protein 39A OS=Hon	1	118.9	97.3	90.7	96.4	108.1	93.55	0.865402405	0.0395467
Q9NS87	KIF15	Kinesin-like protein KIF15 OS=Homo sapien	2	108.7	105.3	97.7	87.4	107	92.55	0.864953271	0.0387426
A8K575		cDNA FLJ76190, highly similar to Homo sap	2	116.6	105.3	91.5	100.4	110.95	95.95	0.864803966	0.0384783
Q8IYD1	GSPT2	Eukaryotic peptide chain release factor GTP-b	1	101.1	88.8	76.1	88.1	94.95	82.1	0.864665613	0.0382348
A0A024R046	HMGN4	High mobility group nucleosomal binding dor	1	108.6	106.8	95.2	91	107.7	93.1	0.864438254	0.0378372
Q8N9N8	EIF1AD	Probable RNA-binding protein EIF1AD OS=l	4	117.7	102.1	100.5	89.5	109.9	95	0.864422202	0.0378093
Q9UBB6	NCDN	Neurochondrin OS=Homo sapiens GN=NCDI	3	116.2	106.3	96.3	96	111.25	96.15	0.864269663	0.0375447
Q8N6L1	KRTCAP2	Keratinocyte-associated protein 2 OS=Homo	1	120.2	96.4	83.6	103.6	108.3	93.6	0.864265928	0.0375382
P63146	UBE2B	Ubiquitin-conjugating enzyme E2 B OS=Hon	1	117.9	97.5	94.2	91.8	107.7	93	0.863509749	0.0362491
A8K7S5	KATNA1	Katanin p60 ATPase-containing subunit A1 C	1	119.3	100.4	89.1	100.6	109.85	94.85	0.863450159	0.0361491
P17858	PFKL	ATP-dependent 6-phosphofructokinase, liver	7	120.1	93	90.6	93.2	106.55	91.9	0.862505866	0.0345944
G3V4K3	VIPAS39	Spermatogenesis-defective protein 39 homolo	1	110.7	99.9	89.5	92.1	105.3	90.8	0.862298196	0.0342601
Q59GG2		Caspase 9 isoform alpha preproprotein variant	1	109	98.6	83.1	95.9	103.8	89.5	0.862235067	0.034159
Q53EN2		MURR1 variant (Fragment) OS=Homo sapier	1	122.5	93.7	84.9	101.5	108.1	93.2	0.862164662	0.0340465
Q9H0N5	PCBD2	Pterin-4-alpha-carbinolamine dehydratase 2 C	1	104.1	98.1	85.3	89	101.1	87.15	0.862017804	0.0338129
B3KQL8		cDNA FLJ90713 fis, clone PLACE1008315, l	1	124.6	97.8	100.8	90.9	111.2	95.85	0.861960432	0.033722
Q9Y5Z9	UBIAD1	UbiA prenyltransferase domain-containing pro	2	111.9	107.4	95.8	93.2	109.65	94.5	0.861833105	0.033521
A0A024R8E4	C9orf32	Chromosome 9 open reading frame 32, isofor	1	111.7	103.1	88	97.1	107.4	92.55	0.861731844	0.0333618
Q7Z589	EMSY	BRCA2-interacting transcriptional repressor E	1	126.2	93.4	87.4	101.8	109.8	94.6	0.861566485	0.0331033
B7Z2P6		cDNA FLJ55296, highly similar to Homo sap	2	107.5	110.5	95.5	92.3	109	93.9	0.86146789	0.03295
F5GXR3	PTMS	Parathymosin OS=Homo sapiens GN=PTMS	1	112.5	100.9	91	92.8	106.7	91.9	0.861293346	0.03268
Q8WXE1	ATRIP	ATR-interacting protein OS=Homo sapiens G	1	117.7	101.3	92.9	95.6	109.5	94.25	0.860730594	0.0318218
Q15800	MSMO1	Methylsterol monooxygenase 1 OS=Homo sa	2	113.9	99.3	97.4	86.1	106.6	91.75	0.860694184	0.0317669
B4DL86		6-phosphogluconate dehydrogenase, decarbox	3	106.4	104.1	90.8	90.3	105.25	90.55	0.860332542	0.0312262
H7C1D1		Uncharacterized protein (Fragment) OS=Hom	1	111.1	98.2	88.4	91.6	104.65	90	0.860009556	0.0307498
O75794	CDC123	Cell division cycle protein 123 homolog OS=l	4	112.9	101.1	90.5	93.5	107	92	0.859813084	0.030463
Q9BX59	TAPBPL	Tapasin-related protein OS=Homo sapiens GN	1	127.9	93.8	106.1	84.5	110.85	95.3	0.859720343	0.0303283
Q9UPP1	PHF8	Histone lysine demethylase PHF8 OS=Homo	1	111	109	85.7	103.4	110	94.55	0.859545455	0.0300759
Q86TN4	TRPT1	tRNA 2'-phosphotransferase 1 OS=Homo sap	1	108.9	116.7	95.4	98.5	112.8	96.95	0.859485816	0.0299901
Q9NRR8	CDC42SE1	CDC42 small effector protein 1 OS=Homo sa	1	114.6	95.1	99.8	80.4	104.85	90.1	0.859322842	0.029757
Q8WUU5	GATAD1	GATA zinc finger domain-containing protein	1	114.9	102.7	96	90.9	108.8	93.45	0.858915441	0.0291808
A8K099		cDNA FLJ77959, highly similar to Homo sap	1	117.7	99.1	92.6	93.6	108.4	93.1	0.858856089	0.0290976
Q96B26	EXOSC8	Exosome complex component RRP43 OS=Ho	10	122.8	96.4	93.7	94.5	109.6	94.1	0.858576642	0.0287087
A8MT69	STRA13	Centromere protein X OS=Homo sapiens GN	1	112.4	94	85.5	91.7	103.2	88.6	0.858527132	0.0286402
P63313	TMSB10	Thymosin beta-10 OS=Homo sapiens GN=TM	1	105.6	99.8	86.4	89.9	102.7	88.15	0.858325219	0.0283626
P53384	NUBP1	Cytosolic Fe-S cluster assembly factor NUBP	2	111.9	105.1	90.8	95.4	108.5	93.1	0.858064516	0.0280073
D3DSQ0	PCM1	Pericentriolar material 1, isoform CRA_a OS=	6	115.5	104.7	95.2	93.7	110.1	94.45	0.857856494	0.0277265
P53602	MVD	Diphosphomevalonate decarboxylase OS=Ho	3	108.3	102.6	90.7	90.2	105.45	90.45	0.857752489	0.027587
A0A024R3S1	C1orf198	Chromosome 1 open reading frame 198, isofo	1	121.7	97.4	90.7	97.2	109.55	93.95	0.85759927	0.0273826
H7C5R1	CP	Ceruloplasmin (Fragment) OS=Homo sapiens	1	120.5	90.6	89.2	91.7	105.55	90.45	0.856939839	0.0265172
Q53GG5	PDLIM3	PDZ and LIM domain protein 3 OS=Homo sa	1	98.2	103.7	83.2	89.8	100.95	86.5	0.856859832	0.0264137
P98198	ATP8B2	Phospholipid-transporting ATPase ID OS=Ho	1	123.3	105.1	96.6	99.1	114.2	97.85	0.856830123	0.0263754
A0A024RAP2	HMGCR	3-hydroxy-3-methylglutaryl coenzyme A redu	1	112.3	97.5	96.3	83.4	104.9	89.85	0.856530029	0.025991
A0A183YE00	TUB4q	Tubulin beta chain OS=Homo sapiens GN=TU	2	105.1	102.6	88.1	89.8	103.85	88.95	0.856523832	0.0259831
Q00613	HSF1	Heat shock factor protein 1 OS=Homo sapien	3	97.6	96	84.8	81	96.8	82.9	0.856404959	0.0258322
Q9BZV1	UBXN6	UBX domain-containing protein 6 OS=Homo	1	106.8	116.7	93.4	98	111.75	95.7	0.856375839	0.0257953
Q9BV57	ADI1	1,2-dihydroxy-3-keto-5-methylthiopentene di	4	104.4	105.7	92.2	87.7	105.05	89.95	0.856258924	0.0256478
A0A0S2Z4U0	RIN1	Ras and Rab interactor 1 (Fragment) OS=Hon	1	110.2	95.7	100.5	75.8	102.95	88.15	0.856240894	0.0256251
Q8N2S1	LTBP4	Latent-transforming growth factor beta-bindir	1	111.1	104.5	93.9	90.7	107.8	92.3	0.856215213	0.0255929
A8K8Z4		cDNA FLJ78071, highly similar to Human M	1	123.4	98.3	96.3	93.5	110.85	94.9	0.856111863	0.0254633
Q53FV1	ORMDL2	ORM1-like protein 2 OS=Homo sapiens GN=	1	120.6	102.9	100.2	91.1	111.75	95.65	0.855928412	0.0252346
Q53HF3		Galactosidase, alpha variant (Fragment) OS=I	4	117.6	100.8	93.7	93.2	109.2	93.45	0.855769231	0.0250375
Q96FJ2	DYNLL2	Dynein light chain 2, cytoplasmic OS=Homo	1	111.4	97.2	87.9	90.5	104.3	89.2	0.855225312	0.024374
Q53G59	KLHL12	Kelch-like protein 12 OS=Homo sapiens GN=	1	113.9	105.7	88.8	99	109.8	93.9	0.855191257	0.024333
P11441	UBL4A	Ubiquitin-like protein 4A OS=Homo sapiens (	2	117.1	105.9	93.8	96.9	111.5	95.35	0.855156951	0.0242917
Q9Y597	KCTD3	BTB/POZ domain-containing protein KCTD3	2	115.1	102.3	90.8	95.1	108.7	92.95	0.855105796	0.0242303
A0A087WXF8	ZCCHC17	Nucleolar protein of 40 kDa OS=Homo sapier	1	104.1	100.6	90.7	84.3	102.35	87.5	0.854909624	0.0239958
Q9H1V8	SLC6A17	Sodium-dependent neutral amino acid transpo	1	109.9	101.6	91.3	89.5	105.75	90.4	0.854846336	0.0239206
B1AKJ6	OSBPL9	Oxysterol-binding protein OS=Homo sapiens	3	126	97	94.1	96.5	111.5	95.3	0.85470852	0.0237574
Q9Y5Q9	GTF3C3	General transcription factor 3C polypeptide 3	14	125.2	95	93.6	94.6	110.1	94.1	0.854677566	0.0237209
Q5SVQ8	ZBTB41	Zinc finger and BTB domain-containing prote	1	112.3	97.9	98.2	81.3	105.1	89.75	0.85394862	0.0228747
O60232	SSSCA1	Sjoegren syndrome/scleroderma autoantigen 1	3	106	117.2	97.6	92.9	111.6	95.25	0.853494624	0.0223607
D2X2H5	nlgn1	Neuroligin 1 OS=Homo sapiens GN=nlgn1 PE	1	103.7	104.3	87.8	89.5	104	88.65	0.852403846	0.0211654
Q86V59	PNMAL1	PNMA-like protein 1 OS=Homo sapiens GN=	3	124	98	90.6	98.4	111	94.5	0.851351351	0.0200641
Q8NDQ0	DKFZp434I21	Putative uncharacterized protein DKFZp434I2	1	95.5	95.7	80.8	81.9	95.6	81.35	0.850941423	0.0196484
Q8WWI1	LMO7	LIM domain only protein 7 OS=Homo sapien	1	105.6	107	85.7	95.2	106.3	90.45	0.850893697	0.0196005
O60583	CCNT2	Cyclin-T2 OS=Homo sapiens GN=CCNT2 PE	1	108	111.9	94.1	93	109.95	93.55	0.850841291	0.019548
Q8NDX6	ZNF740	Zinc finger protein 740 OS=Homo sapiens GN	2	119.5	92.8	83.4	97.2	106.15	90.3	0.850682996	0.0193902
B2RBC1		cDNA, FLJ95432, highly similar to Homo sap	1	108	94.8	90.1	82.4	101.4	86.25	0.850591716	0.0192997
Q9NP77	SSU72	RNA polymerase II subunit A C-terminal dom	2	108	100.8	89.9	87.7	104.4	88.8	0.850574713	0.0192829
M0R2B7	POLD1	DNA polymerase OS=Homo sapiens GN=PO	6	115.8	103.7	93.3	93.3	109.75	93.3	0.850113895	0.0188316
P31323	PRKAR2B	cAMP-dependent protein kinase type II-beta r	2	113.1	101.7	89.2	93.4	107.4	91.3	0.85009311	0.0188116
F8W0P7	ATP5B	ATP synthase subunit beta, mitochondrial (Fra	2	115.7	103.1	91.2	94.8	109.4	93	0.850091408	0.0188099
A6P4V4		Tyrosine-protein kinase receptor OS=Homo sa	1	112.1	104.8	92.3	91.9	108.45	92.1	0.849239281	0.0180001
A4D1Z4	KIAA0415	KIAA0415 gene product OS=Homo sapiens G	1	107.4	103.3	90.2	88.7	105.35	89.45	0.849074514	0.017847
Q4JM47	AGR2	AGR2 OS=Homo sapiens GN=AGR2 PE=4 S	4	109.4	109.3	93.9	91.7	109.35	92.8	0.84865112	0.0174586
Q8IV63	VRK3	Inactive serine/threonine-protein kinase VRK3	1	104.5	122.1	95	97.3	113.3	96.15	0.848631951	0.0174412
B4DUH8		cDNA FLJ60163, highly similar to Carbonic a	1	105.4	114.1	94	92.1	109.75	93.05	0.847835991	0.0167313
Q9H6S0	YTHDC2	Probable ATP-dependent RNA helicase YTH	1	103.9	107.2	88.5	90.3	105.55	89.4	0.846991947	0.0160056
A8K3Y8		cDNA FLJ76141 OS=Homo sapiens PE=2 SV	3	107.9	100.4	88.5	87.9	104.15	88.2	0.846855497	0.0158909
Q7Z7A4	PXK	PX domain-containing protein kinase-like pro	1	116.3	101.2	87.8	96.3	108.75	92.05	0.846436782	0.0155432
Q14119	VEZF1	Vascular endothelial zinc finger 1 OS=Homo	1	110.1	112.2	91.9	96.1	111.15	94	0.845704004	0.0149503
Q8IZP0	ABI1	Abl interactor 1 OS=Homo sapiens GN=ABI1	2	120.6	104.2	93.9	96.2	112.4	95.05	0.845640569	0.0148999
A1L188	NDUFAF8	NADH dehydrogenase [ubiquinone] 1 alpha s	1	120.2	103.3	93	95.9	111.75	94.45	0.845190157	0.0145463
B3KNT3	SRA1	Steroid receptor RNA activator 1, isoform CR	2	113.5	101.8	94.4	87.5	107.65	90.95	0.844867627	0.0142975
B4DTW9		cDNA FLJ57043, highly similar to Protein EM	1	104.8	96.9	64.9	105.5	100.85	85.2	0.844819038	0.0142603
Q8NBF2	NHLRC2	NHL repeat-containing protein 2 OS=Homo s	7	111.6	100.8	91	88.4	106.2	89.7	0.844632768	0.0141187
B8ZWD9	DBI	Diazepam binding inhibitor, splice form 1D(2	3	104.1	104.3	88.4	87.6	104.2	88	0.84452975	0.0140408
Q9NVT9	ARMC1	Armadillo repeat-containing protein 1 OS=Ho	3	116.9	106.6	89.7	99	111.75	94.35	0.844295302	0.0138651
A0A0C4ZLW	CDK5RAP2	CDK5RAP2 protein OS=Homo sapiens GN=C	1	109.1	110.1	96.6	88.4	109.6	92.5	0.843978102	0.0136303
K7ELC2	RPS15	40S ribosomal protein S15 OS=Homo sapiens	5	114.2	108.7	95.3	92.8	111.45	94.05	0.843876178	0.0135556
Q5BLP8	C4orf48	Neuropeptide-like protein C4orf48 OS=Homo	1	126.8	96.7	91	97.6	111.75	94.3	0.843847875	0.0135349
P52569	SLC7A2	Cationic amino acid transporter 2 OS=Homo s	2	132.9	100.6	97.3	99.7	116.75	98.5	0.843683084	0.013415
Q9BX10	GTPBP2	GTP-binding protein 2 OS=Homo sapiens GN	2	118.7	103.5	91.2	96.2	111.1	93.7	0.843384338	0.0131999
Q9Y3C7	MED31	Mediator of RNA polymerase II transcription	2	117.7	103.1	93.9	92.3	110.4	93.1	0.843297101	0.0131377
V9HW44	HEL-S-303	Epididymis secretory protein Li 303 OS=Hom	3	111.4	110.2	92.6	94.2	110.8	93.4	0.842960289	0.0128999

A0A024R9A9	UBE2T	Ubiquitin-conjugating enzyme E2T (Putative)	5	101.6	105.6	87.1	87.4	103.6	87.25	0.842181467	0.012364
G5EKE5	PPFIBP1-ALK	Tyrosine-protein kinase receptor OS=Homo s	1	125.6	101.2	95.1	95.8	113.4	95.45	0.841710758	0.0120495
Q96HR9	REEP6	Receptor expression-enhancing protein 6 OS=	1	113.1	100.2	88.8	90.6	106.65	89.7	0.841068917	0.0116319
Q8WUY1	THEM6	Protein THEM6 OS=Homo sapiens GN=THE	4	114.3	105.7	96	89	110	92.5	0.840909091	0.0115299
P49459	UBE2A	Ubiquitin-conjugating enzyme E2 A OS=Hom	2	125.7	96.4	91.2	95.5	111.05	93.35	0.840612337	0.0113425
B4DL14		ATP synthase subunit gamma OS=Homo sapi	1	136.7	107.2	107.9	97.1	121.95	102.5	0.840508405	0.0112775
F2Z2X4	XPO4	Exportin-4 OS=Homo sapiens GN=XPO4 PE	1	125.7	95	84.6	100.9	110.35	92.75	0.840507476	0.0112769
V9HW77	HEL-211	Epididymis luminal protein 211 OS=Homo sa	11	105	106.7	88.7	89.1	105.85	88.9	0.839867737	0.0108839
Q9P0J7	KCMF1	E3 ubiquitin-protein ligase KCMF1 OS=Hom	1	100.7	132.4	99.9	95.7	116.55	97.8	0.839124839	0.0104426
P16298	PPP3CB	Serine/threonine-protein phosphatase 2B catal	2	115.9	102.6	88.4	94.9	109.25	91.65	0.838901602	0.010313
O00418	EEF2K	Eukaryotic elongation factor 2 kinase OS=Ho	1	110.8	105.9	93	88.7	108.35	90.85	0.838486387	0.0100757
Q9UKD1	GMEB2	Glucocorticoid modulatory element-binding p	1	109.1	108.9	94.9	87.8	109	91.35	0.838073394	0.0098444
Q08AD1	CAMSAP2	Calmodulin-regulated spectrin-associated prot	2	94	120.5	90.8	88.9	107.25	89.85	0.837762238	0.00967322
Q08289	CACNB2	Voltage-dependent L-type calcium channel su	1	121.8	94.7	90.9	90.4	108.25	90.65	0.837413395	0.00948436
P48634	PRRC2A	Protein PRRC2A OS=Homo sapiens GN=PRI	4	119.9	94.1	91.6	87.5	107	89.55	0.836915888	0.00922063
P00352	ALDH1A1	Retinal dehydrogenase 1 OS=Homo sapiens C	23	103.3	109.4	87.6	90.4	106.35	89	0.836859426	0.00919108
Q8N806	UBR7	Putative E3 ubiquitin-protein ligase UBR7 OS	1	97.4	125.1	96.2	90	111.25	93.1	0.836853933	0.00918821
L7RXH5	MAPK3	Mitogen-activated protein kinase OS=Homo s	2	112.2	102.9	89.8	90.2	107.55	90	0.836820084	0.00917056
A0A024R9R5	ZFP106	Zinc finger protein 106 homolog (Mouse), iso	2	118.9	102.9	97.3	88.3	110.9	92.8	0.836789901	0.00915485
Q8NEN9	PDZD8	PDZ domain-containing protein 8 OS=Homo	2	113.3	114.5	98.3	92.2	113.9	95.25	0.836259877	0.00888262
Q8IZT6	ASPM	Abnormal spindle-like microcephaly-associate	1	110	103.5	86	92.5	106.75	89.25	0.836065574	0.0087846
A0A024R1M5	ZNF278	Zinc finger protein 278, isoform CRA_c OS=	2	104.3	121.3	95.2	93.4	112.8	94.3	0.835992908	0.00874819
O00178	GTPBP1	GTP-binding protein 1 OS=Homo sapiens GN	5	122.3	100.7	90.6	95.8	111.5	93.2	0.835874439	0.00868909
Q66LE6	PPP2R2D	Serine/threonine-protein phosphatase 2A 55 k	1	130.7	96.5	101.4	88.5	113.6	94.95	0.835827465	0.00866576
Q96AE4	FUBP1	Far upstream element-binding protein 1 OS=H	2	108.2	108.9	93.7	87.7	108.55	90.7	0.83555965	0.00853379
Q8IWV8	UBR2	E3 ubiquitin-protein ligase UBR2 OS=Homo	1	119.7	100.4	95.5	88.4	110.05	91.95	0.835529305	0.00851896
Q59HH0		Retinoblastoma-associated protein variant (Fr	1	118.1	97	96.4	83.3	107.55	89.85	0.835425384	0.00846828
P62877	RBX1	E3 ubiquitin-protein ligase RBX1 OS=Homo	1	106.3	113.3	96.6	86.8	109.8	91.7	0.835154827	0.00833763
B7Z287		cDNA FLJ55197, highly similar to Protein FA	1	119.4	102	98	86.9	110.7	92.45	0.835140018	0.00833051
Q75QN2	INTS8	Integrator complex subunit 8 OS=Homo sapie	5	120.3	99.2	91	92.3	109.75	91.65	0.835079727	0.00830168
Q4VC00	MAN2B2	Alpha-mannosidase OS=Homo sapiens GN=M	1	113.7	96	78	97	104.85	87.5	0.834525513	0.00804053
P51790	CLCN3	H(+)/Cl(-) exchange transporter 3 OS=Homo	4	95.4	94.8	101.6	106.8	95.1	104.2	1.095688749	0.0501486
P05165	PCCA	Propionyl-CoA carboxylase alpha chain, mito	2	87.7	100	99.3	106.3	93.85	102.8	1.095364944	0.0507707
P09669	COX6C	Cytochrome c oxidase subunit 6C OS=Homo	5	93.8	100.5	108.4	104.4	97.15	106.4	1.095213587	0.0510639
Q59EN5		Prosaposin variant (Fragment) OS=Homo sap	21	97.6	96	107.5	104.5	96.8	106	1.095041322	0.0513993
Q86U90	YRDC	YrdC domain-containing protein, mitochondri	4	95.6	89.6	98.8	104	92.6	101.4	1.095032397	0.0514165
P08134	RHOC	Rho-related GTP-binding protein RhoC OS=H	2	98	100	107.4	109.4	99	108.4	1.094949495	0.0515788
Q8IV76	PASD1	Circadian clock protein PASD1 OS=Homo sa	1	98.2	96.3	102.1	110.8	97.25	106.45	1.094601542	0.0522641
A0A0S2Z5P2	GINS3	GINS complex subunit 3 isoform 2 (Fragment	2	91.6	95	104.2	100	93.3	102.1	1.0943194	0.0528255
A1L4Q0	DGKE	Diacylglycerol kinase OS=Homo sapiens GN:	1	100.5	91.2	104.1	105.6	95.85	104.85	1.093896714	0.0536761
H3BMD8	ARPP19	cAMP-regulated phosphoprotein 19 OS=Hom	1	85.2	104.8	96.9	110.8	95	103.85	1.093157895	0.0551914
Q5T9A4	ATAD3B	ATPase family AAA domain-containing prote	7	88.6	86.2	92.2	98.8	87.4	95.5	1.092677346	0.0561965
Q9Y221	NIP7	60S ribosome subunit biogenesis protein NIP7	9	93.8	97.2	105	103.7	95.5	104.35	1.092670157	0.0562116
Q8IYK4	COLGALT2	Procollagen galactosyltransferase 2 OS=Hom	3	95.5	101.1	107.4	107.4	98.3	107.4	1.092573754	0.0564153
Q8N697	SLC15A4	Solute carrier family 15 member 4 OS=Homo	2	88.6	86	88	102.7	87.3	95.35	1.092210767	0.0571875
Q8WWX9	SELM	Selenoprotein M OS=Homo sapiens GN=SEL	2	94.3	98.9	103.9	107.1	96.6	105.5	1.092132505	0.0573552
A0A087WVA	TEX2	Testis-expressed sequence 2 protein OS=Hom	1	97.9	93.4	105.9	103	95.65	104.45	1.092002091	0.0576356
P22415	USF1	Upstream stimulatory factor 1 OS=Homo sapi	2	92.5	91.2	99.6	101	91.85	100.3	1.091997823	0.0576445
Q9Y2Z4	YARS2	Tyrosine--tRNA ligase, mitochondrial OS=Ho	10	96.2	98.1	106.4	105.7	97.15	106.05	1.091610911	0.0584834
B7Z6K5		cDNA FLJ54805, highly similar to Ionized ca	2	98.2	97.9	104.9	109.1	98.05	107	1.091279959	0.059209
Q9NUJ7	PLCXD1	PI-PLC X domain-containing protein 1 OS=H	1	92.4	104.6	103.9	111	98.5	107.45	1.090862944	0.0601342
P11717	IGF2R	Cation-independent mannose-6-phosphate rec	59	97.9	96.9	107.4	105	97.4	106.2	1.090349076	0.0612915
Q9H6K4	OPA3	Optic atrophy 3 protein OS=Homo sapiens GI	1	95.2	93.7	108.5	97.4	94.45	102.95	1.089994706	0.0621006
B4DRQ6		Eyes absent homolog OS=Homo sapiens PE=	1	90.6	100.6	97.8	110.6	95.6	104.2	1.089958159	0.0621844
Q9Y305	ACOT9	Acyl-coenzyme A thioesterase 9, mitochondri	15	98.4	96.8	104.2	108.5	97.6	106.35	1.089651639	0.0628926
Q8N4H5	TOMM5	Mitochondrial import receptor subunit TOM5	2	92.5	102.8	105.3	107.5	97.65	106.4	1.089605735	0.0629991
Q969Y2	GTPBP3	tRNA modification GTPase GTPBP3, mitoch	4	92.1	101.3	103.6	107.1	96.7	105.35	1.089451913	0.0633574
Q0ZGT2	NEXN	Nexilin OS=Homo sapiens GN=NEXN PE=1	4	98.5	100.6	106.1	110.8	99.55	108.45	1.08940231	0.0634733
B2R8N1		cDNA, FLJ93976, highly similar to Homo sap	5	92.3	95.6	99.2	105.4	93.95	102.3	1.088877062	0.0647119
P30044	PRDX5	Peroxiredoxin-5, mitochondrial OS=Homo sa	9	94.5	95.9	103.1	104.2	95.2	103.65	1.088760504	0.0649897
Q99595	TIMM17A	Mitochondrial import inner membrane translo	3	97.7	94.5	102.9	106.3	96.1	104.6	1.088449532	0.0657355
H6VRG1	KRT1	Keratin 1 OS=Homo sapiens GN=KRT1 PE=:	17	94.8	103.2	109.9	105.6	99	107.75	1.088383838	0.0658939
P60520	GABARAPL2	Gamma-aminobutyric acid receptor-associate	2	94.9	94.4	102.9	103.1	94.65	103	1.08821975	0.066291
Q9Y6S7	unknown	Uncharacterized protein OS=Homo sapiens G	1	91.2	104.8	99.7	113.5	98	106.6	1.087755102	0.0674269
Q15293	RCN1	Reticulocalbin-1 OS=Homo sapiens GN=RCN	14	99.2	96.9	106.8	106.5	98.05	106.65	1.087710352	0.067537
P35611	ADD1	Alpha-adducin OS=Homo sapiens GN=ADD1	6	96	95.7	96.2	112.3	95.85	104.25	1.087636933	0.0677183
K7EKE6	LONP1	Lon protease homolog, mitochondrial OS=Ho	32	95.8	98.3	105.9	105.2	97.05	105.55	1.08758372	0.0678501
A0A024R608	RPLP1	Ribosomal protein, large, P1, isoform CRA_a	1	97.8	99.2	102.3	111.9	98.5	107.1	1.087309645	0.0685313
P62745	RHOB	Rho-related GTP-binding protein RhoB OS=H	2	95.6	99.3	110.1	101.8	97.45	105.95	1.087224218	0.0687447
A8K2T7		Receptor protein-tyrosine kinase OS=Homo s	4	102.6	92.5	102.7	109.4	97.55	106.05	1.087134803	0.0689688
J3KNA0	OXA1L	Mitochondrial inner membrane protein OXA1	8	96.2	96.9	102.7	107.2	96.55	104.95	1.087001554	0.0693039
E7EX18	MPV17	Protein Mpv17 OS=Homo sapiens GN=MPV	1	90.3	102	108.2	100.7	96.15	104.45	1.086323453	0.0710306
A0A0A0MRL	CASP7	Caspase-7 OS=Homo sapiens GN=CASP7 PE	1	89.9	102.6	99.9	109.2	96.25	104.55	1.086233766	0.0712619
Q53F64		Heterogeneous nuclear ribonucleoprotein AB	17	94.8	101.6	105.4	107.9	98.2	106.65	1.08604888	0.0717403
Q6KCM7	SLC25A25	Calcium-binding mitochondrial carrier protein	3	93.8	99.3	99.6	110.1	96.55	104.85	1.085965821	0.0719559
B7Z6S9		Glucosylceramidase OS=Homo sapiens PE=2	8	96.6	99.1	106.1	106.4	97.85	106.25	1.085845682	0.0722691
P54709	ATP1B3	Sodium/potassium-transporting ATPase subu	9	100.3	96.8	110.2	103.8	98.55	107	1.085743278	0.0725368
P20645	M6PR	Cation-dependent mannose-6-phosphate recep	6	101.3	98.3	110.2	106.4	99.8	108.3	1.085170341	0.0740506
P27144	AK4	Adenylate kinase 4, mitochondrial OS=Homo	5	97.3	95.8	104.5	105	96.55	104.75	1.084930088	0.0746934
Q9NX58	LYAR	Cell growth-regulating nucleolar protein OS=	14	96.1	96.1	103.8	104.7	96.1	104.25	1.084807492	0.075023
P62070	RRAS2	Ras-related protein R-Ras2 OS=Homo sapien	4	101.5	98.1	110.8	105.7	99.8	108.25	1.084669339	0.0753961
O95470	SGPL1	Sphingosine-1-phosphate lyase 1 OS=Homo s	9	97.7	96.4	104.2	106.3	97.05	105.25	1.08449253	0.0758757
Q9UK23	NAGPA	N-acetylglucosamine-1-phosphodiester alpha-	1	102.7	95.9	100	115.3	99.3	107.65	1.08408862	0.0769815
P33981	TTK	Dual specificity protein kinase TTK OS=Hom	1	96.5	100	106.9	106.1	98.25	106.5	1.083969466	0.07731
A0A024R0W3	SLC38A2	Solute carrier family 38, member 2, isoform C	4	102	93.4	109.3	102.5	97.7	105.9	1.083930399	0.0774181
J3KPF3	SLC3A2	4F2 cell-surface antigen heavy chain OS=Hor	16	99.3	99.7	107.2	108.5	99.5	107.85	1.083919598	0.0774478
Q8IUW5	RELL1	RELT-like protein 1 OS=Homo sapiens GN=I	2	92.1	103.5	102.1	109.9	97.8	106	1.083844581	0.0776559
B3KPM6		cDNA FLJ31963 fis, clone NT2RP7007610, l	1	99.7	90	101	104.6	94.85	102.8	1.083816552	0.0777337
A0A0K2BMD	HBA2	Mutant hemoglobin alpha 2 globin chain OS=	4	99.2	97.7	110.7	102.7	98.45	106.7	1.083798883	0.0777827
Q9HD26	GOPC	Golgi-associated PDZ and coiled-coil motif-c	2	99.4	100.4	94.6	121.9	99.9	108.25	1.083583584	0.0783826
Q9Y277	VDAC3	Voltage-dependent anion-selective channel pr	13	94.2	98.6	103.7	105.2	96.4	104.45	1.083506224	0.0785992
A0A024R892	LRRC8A	Leucine rich repeat containing 8 family, mem	7	100	98.8	108	107.4	99.4	107.7	1.083501006	0.0786139
A0A059RS35	CYTB	Cytochrome b OS=Homo sapiens GN=CYTB	1	82	90.5	91.5	95.4	86.25	93.45	1.083478261	0.0786777
A8MTT3	CEBPZOS	Protein CEBPZOS OS=Homo sapiens GN=C	1	95.7	94.8	109.6	96.8	95.25	103.2	1.083464567	0.0787158
Q6P587	FAHD1	Acylpyruvase FAHD1, mitochondrial OS=Ho	4	97.3	98.6	107	105.2	97.95	106.1	1.083205717	0.0794451
A0A024RDV7	KPNA3	Importin subunit alpha OS=Homo sapiens GN	3	94	98.3	102.5	105.8	96.15	104.15	1.083203328	0.0794519
Q9BSR8	YIPF4	Protein YIPF4 OS=Homo sapiens GN=YIPF4	1	91.4	91.7	100.5	97.8	91.55	99.15	1.083014746	0.0799866
E9PSG9	POLR2M	DNA-directed RNA polymerase II subunit GF	1	97.5	89.5	97.3	105.2	93.5	101.25	1.082887701	0.0803487
A0A0S4T3S1	HLA-C	MHC class I antigen OS=Homo sapiens GN=	1	111.7	90.2	117.6	101	100.95	109.3	1.082714215	0.0808451
Q9UKZ7		Colon carcinoma related protein OS=Homo sa	1	95.5	112.5	114.8	110.4	104	112.6	1.082692308	0.080908
Q9H1C7	CYSTM1	Cysteine-rich and transmembrane domain-con	1	103.3	91	107.2	103.1	97.15	105.15	1.082346886	0.0819047
Q04837	SSBP1	Single-stranded DNA-binding protein, mitoch	9	92.4	98.5	102.2	104.4	95.45	103.3	1.082242012	0.0822094
Q53SZ7	PRR30	Proline-rich protein 30 OS=Homo sapiens GN	1	97.1	102.4	107.1	108.8	99.75	107.95	1.082205514	0.0823156
Q14147	DHX34	Probable ATP-dependent RNA helicase DHX	1	104.3	77.2	94.3	102.1	90.75	98.2	1.082093664	0.0826419
Q16832	DDR2	Discoidin domain-containing receptor 2 OS=I	7	98	100.7	108.7	106.3	99.35	107.5	1.082033216	0.082819
A8K171		cDNA FLJ77253, highly similar to Homo sap	1	105.5	85.9	99.3	107.8	95.7	103.55	1.082027168	0.0828365
P01893	HLA-H	Putative HLA class I histocompatibility antige	1	94.3	100.8	104.8	106.3	97.55	105.55	1.082009226	0.0828892
Q8N1G0	ZNF687	Zinc finger protein 687 OS=Homo sapiens GI	3	98.8	93.3	94	113.8	96.05	103.9	1.081728267	0.0837159
Q9H9S5	FKRP	Fukutin-related protein OS=Homo sapiens GN	1	92.9	99.7	99.2	109.1	96.3	104.15	1.081516096	0.0843444
Q9BZM5	ULBP2	NKG2D ligand 2 OS=Homo sapiens GN=UL	1	104.3	88.4	109.4	99	96.35	104.2	1.081473793	0.0844703
A8K0M6		cDNA FLJ76697 OS=Homo sapiens PE=2 SV	5	98.7	95.6	102.9	107.2	97.15	105.05	1.08131755	0.0849365
D3DV26	S100A10	S100 calcium binding protein A10 (Annexin I	3	98.7	105.6	106.5	114.4	102.15	110.45	1.081253059	0.0851295
A0A024RBH2	CKAP4	Cytoskeleton-associated protein 4, isoform CI	33	100	96	105.7	106.2	98	105.95	1.081122449	0.0855217
Q5HYM0	ZC3H12B	Probable ribonuclease ZC3H12B OS=Homo s	1	91.9	107.9	107.4	108.6	99.9	108	1.081081081	0.0856462
Q8NHU3	SGMS2	Phosphatidylcholine:ceramide cholinephosph	2	95.9	97.8	101.6	107.8	96.85	104.7	1.081053175	0.0857302
Q5JYT7	KIAA1755	Uncharacterized protein KIAA1755 OS=Hom	1	97.6	98.6	109.6	102.5	98.1	106.05	1.081039755	0.0857705
O43464	HTRA2	Serine protease HTRA2, mitochondrial OS=H	5	99.1	93.6	104.8	103.5	96.35	104.15	1.080954852	0.0860267
B3KT93		Polyadenylate-binding protein OS=Homo sap	11	94.9	100.8	104.3	107.2	97.85	105.75	1.08073582	0.086691
Q9P2K3	RCOR3	REST corepressor 3 OS=Homo sapiens GN=I	1	98.6	89.8	90.1	113.5	94.2	101.8	1.080679406	0.0868626
Q969E2	SCAMP4	Secretory carrier-associated membrane protei	1	99.3	95.3	108.8	101.4	97.3	105.1	1.08016444	0.0884435
Q8N283	ANKRD35	Ankyrin repeat domain-containing protein 35	1	100.1	98.3	108.6	105.7	99.2	107.15	1.080141129	0.0885154
B3KSU4		cDNA FLJ36986 fis, clone BRACE2006598,	1	92.2	95.5	91.3	111.4	93.85	101.35	1.079914758	0.0892183

B4DEE8		cDNA FLJ56323, highly similar to Methylate	1	85.3	97.5	95.4	102	91.4	98.7	1.079868709	0.0893622
Q9UI09	NDUFA12	NADH dehydrogenase [ubiquinone] 1 alpha s	8	94.1	95.1	102.3	102	94.6	102.15	1.079809725	0.0895464
Q15572	TAF1C	TATA box-binding protein-associated factor I	1	105.9	82.1	107.7	95.3	94	101.5	1.079787234	0.0896164
P26368	U2AF2	Splicing factor U2AF 65 kDa subunit OS=Ho	16	95	99.3	105.5	104.3	97.15	104.9	1.079773546	0.0896593
Q9H3N1	TMX1	Thioredoxin-related transmembrane protein 1	9	97.3	98.5	107.2	104.2	97.9	105.7	1.079673136	0.0899738
Q86WC4	OSTM1	Osteopetrosis-associated transmembrane prote	2	97.4	96.1	98.9	110	96.75	104.45	1.079586563	0.090246
G3V3D1	NPC2	Epididymal secretory protein E1 (Fragment) C	7	98	96	105	104.4	97	104.7	1.079381443	0.0908928
P05161	ISG15	Ubiquitin-like protein ISG15 OS=Homo sapie	4	96	98.1	106.2	103.3	97.05	104.75	1.079340546	0.0910222
Q9NZV5	SEPN1	Selenoprotein N OS=Homo sapiens GN=SEP	1	98.2	86.7	101.5	98	92.45	99.75	1.078961601	0.0922293
A0A0A8K8C(	URLC2	Up-regulated in lung cancer 2 OS=Homo sapi	3	91.2	88.8	92.6	101.6	90	97.1	1.078888889	0.0924624
Q8IXM2	BAP18	Chromatin complexes subunit BAP18 OS=Ho	2	95.6	97.1	103.7	104.2	96.35	103.95	1.078879087	0.0924938
P23470	PTPRG	Receptor-type tyrosine-protein phosphatase ga	1	104.1	90.4	97.6	112.2	97.25	104.9	1.078663239	0.0931889
B3KWW3		cDNA FLJ44017 fis, clone TESTI4025920, w	1	101.9	111.9	118.5	112.1	106.9	115.3	1.07857811	0.0934642
M0QZM1	HNRNPM	Heterogeneous nuclear ribonucleoprotein M (	1	99.9	97.5	104.1	108.8	98.7	106.45	1.07852077	0.09365
A8K9T3		cDNA FLJ76467, highly similar to Homo sap	23	99.7	99.2	108.4	106.1	99.45	107.25	1.078431373	0.0939404
Q96J01	THOC3	THO complex subunit 3 OS=Homo sapiens G	9	94.1	98.5	99.7	108	96.3	103.85	1.078400831	0.0940396
B2R4H6		cDNA, FLJ92093, highly similar to Homo sap	2	91.9	104.1	101.9	109.4	98	105.65	1.078061224	0.0951504
Q9Y2Q5	LAMTOR2	Ragulator complex protein LAMTOR2 OS=H	6	95.5	96.2	105	101.6	95.85	103.3	1.077725613	0.0962585
K7EQD6	NSF	Vesicle-fusing ATPase (Fragment) OS=Homo	3	98	99.2	104.4	108.1	98.6	106.25	1.077586207	0.0967221
A4UCT1		Glyceraldehyde 3-phosphate dehydrogenase (	1	98.7	87.2	89.2	111.1	92.95	100.15	1.077461001	0.0971399
Q8IWB1	ITPRIP	Inositol 1,4,5-trisphosphate receptor-interactin	2	102.6	96.4	110.5	103.9	99.5	107.2	1.077386935	0.0973876
B2RCL4		cDNA, FLJ96143, highly similar to Homo sap	2	96.3	93.7	95.1	109.6	95	102.35	1.077368421	0.0974499
P19827	ITIH1	Inter-alpha-trypsin inhibitor heavy chain H1 C	1	105	87.6	100.5	107	96.3	103.75	1.077362409	0.0974699
Q96HE7	ERO1A	ERO1-like protein alpha OS=Homo sapiens G	15	98.1	97.2	105.9	104.5	97.65	105.2	1.077316948	0.0976222
Q9H1E3	NUCKS1	Nuclear ubiquitous casein and cyclin-depende	3	95.3	92.3	89.4	112.7	93.8	101.05	1.077292111	0.0977059
A8K644	SFRS4	Splicing factor, arginine/serine-rich 4, isoforn	6	95.4	96.4	104.8	101.8	95.9	103.3	1.077163712	0.098138
Q15758	SLC1A5	Neutral amino acid transporter B(0) OS=Hom	9	106.6	92.2	109.7	104.4	99.4	107.05	1.076961771	0.0988208
H0YEH1	PICALM	Phosphatidylinositol-binding clathrin assembl	1	104.4	85.6	105.1	99.5	95	102.3	1.076842105	0.0992273
B3KSM6		cDNA FLJ36606 fis, clone TRACH2015654,	1	107.5	86.9	103.4	105.9	97.2	104.65	1.076646091	0.0998959
O43752	STX6	Syntaxin-6 OS=Homo sapiens GN=STX6 PE=	6	101	97.5	104.2	109.5	99.25	106.85	1.076574307	0.100142
P68431	HIST1H3A	Histone H3.1 OS=Homo sapiens GN=HIST1I	3	79.3	81.5	81.5	91.6	80.4	86.55	1.076492537	0.100422
A0A024R755	CALU	Calumenin, isoform CRA_a OS=Homo sapien	1	102	94.1	105.2	105.9	98.05	105.55	1.076491586	0.100426
A0A024R6A5	PPM1A	Protein phosphatase 1A (Formerly 2C), magn	3	97.8	91.8	95.6	108.5	94.8	102.05	1.076476793	0.100476
Q96IZ0	PAWR	PRKC apoptosis WT1 regulator protein OS=H	3	98.9	94.5	105.2	102.9	96.7	104.05	1.076008273	0.102098
O43707	ACTN4	Alpha-actinin-4 OS=Homo sapiens GN=ACT	40	100.3	98.4	107.2	106.6	99.35	106.9	1.075993961	0.102147
B7ZLW8	MTR	5-methyltetrahydrofolate-homocysteine methy	1	92	98.9	93.9	111.5	95.45	102.7	1.075955998	0.10228
B7Z992		cDNA FLJ53698, highly similar to Gelsolin C	9	91	92.4	98.1	99.2	91.7	98.65	1.075790622	0.102858
Q02252	ALDH6A1	Methylmalonate-semialdehyde dehydrogenase	3	92.6	96.5	100	103.4	94.55	101.7	1.075621364	0.103453
Q9P2T0	THEG	Testicular haploid expressed gene protein OS=	1	91.9	104.3	99.9	111.1	98.1	105.5	1.075433231	0.104117
P04181	OAT	Ornithine aminotransferase, mitochondrial OS	17	95.6	97.1	103.8	103.4	96.35	103.6	1.075246497	0.10478
P21796	VDAC1	Voltage-dependent anion-selective channel pr	20	94.2	97.6	102.5	103.7	95.9	103.1	1.075078206	0.10538
Q86WA6	BPHL	Valacyclovir hydrolase OS=Homo sapiens GN	1	101.4	96.1	96.1	116.2	98.75	106.15	1.074936709	0.105887
O95210	STBD1	Starch-binding domain-containing protein 1 C	6	100.2	94.8	105.8	103.8	97.5	104.8	1.074871795	0.10612
A0A024R8P8	RPL38	Ribosomal protein L38, isoform CRA_a OS=I	4	92.7	104.3	105.3	106.4	98.5	105.85	1.074619289	0.107031
Q13707	ACTA2	ACTA2 protein (Fragment) OS=Homo sapien	5	98.4	99	104.9	107.2	98.7	106.05	1.074468085	0.107579
P19338	NCL	Nucleolin OS=Homo sapiens GN=NCL PE=1	39	93.8	97.3	100.8	104.5	95.55	102.65	1.074306646	0.108168
X6RAL5	SAP18	Histone deacetylase complex subunit SAP18 (	9	96.8	99.1	106.2	104.2	97.95	105.2	1.074017356	0.109229
P30040	ERP29	Endoplasmic reticulum resident protein 29 OS	14	97.1	96.2	104.5	103.1	96.65	103.8	1.073978272	0.109373
Q13641	TPBG	Trophoblast glycoprotein OS=Homo sapiens (	2	103.2	99.6	116.3	101.5	101.4	108.9	1.073964497	0.109424
P56177	DLX1	Homeobox protein DLX-1 OS=Homo sapiens	1	89.1	100.3	100.8	102.6	94.7	101.7	1.073917635	0.109596
Q6FIE9	TOLLIP	TOLLIP protein OS=Homo sapiens GN=TOL	4	101.6	92.3	104.1	104.1	96.95	104.1	1.073749355	0.110219
B3KVJ8		Chloride channel protein OS=Homo sapiens F	5	97	96.9	103.5	104.7	96.95	104.1	1.073749355	0.110219
A6XMH4		Beta-2-microglobulin OS=Homo sapiens PE=	1	104.3	96.4	113.7	101.8	100.35	107.75	1.073741903	0.110247
O95292	VAPB	Vesicle-associated membrane protein-associat	10	98.5	98.4	107.1	104.3	98.45	105.7	1.073641442	0.11062
B4DN86		cDNA FLJ56047, highly similar to A kinase a	4	93.7	99.2	101.7	105.4	96.45	103.55	1.073613271	0.110725
L0R6G9	FUNDC2	Alternative protein FUNDC2 OS=Homo sapie	1	97.4	114.5	114.7	112.7	105.95	113.7	1.073147711	0.112469
B7ZLW2	HELZ	HELZ protein OS=Homo sapiens GN=HELZ	3	93.4	95	98.3	103.8	94.2	101.05	1.072717622	0.1141
Q9BUL9	RPP25	Ribonuclease P protein subunit p25 OS=Hom	5	89.9	94.9	98	100.2	92.4	99.1	1.072510823	0.114892
A0A0K0L4Y0	PNPLA2	Patatin-like phospholipase domain-containing	2	112.9	91.3	106.8	112.2	102.1	109.5	1.072477963	0.115018
A0A024R415	PNKD	Paroxysmal nonkinesiogenic dyskinesia, isofo	1	95.6	93.5	99	103.8	94.55	101.4	1.07244844	0.115131
H0UI06	COX7A2	Cytochrome c oxidase subunit 7A2, mitochon	2	98.8	94.3	104.6	102.4	96.55	103.5	1.071983428	0.116929
O75964	ATP5L	ATP synthase subunit g, mitochondrial OS=H	6	94.8	101.1	101.6	108.4	97.95	105	1.071975498	0.116961
Q5D1D5		Guanylate binding protein 1 OS=Homo sapien	10	100.8	97.9	106.5	106.5	99.35	106.5	1.071967791	0.11699
Q9Y6I3	EPN1	Epsin-1 OS=Homo sapiens GN=EPN1 PE=1	5	92.6	99.2	96.7	108.9	95.9	102.8	1.071949948	0.11706
Q53Y06	ATP6V1E1	ATPase, H+ transporting, lysosomal 31kDa, V	11	95.7	97.5	104	103.1	96.6	103.55	1.07194617	0.117075
P20674	COX5A	Cytochrome c oxidase subunit 5A, mitochond	8	92.2	100.4	100.6	105.8	96.3	103.2	1.07165109	0.118229
B2R5I8		cDNA, FLJ92490, highly similar to Homo sap	9	99.1	96.4	106	103.5	97.75	104.75	1.071611253	0.118385
O75145	PPFIA3	Liprin-alpha-3 OS=Homo sapiens GN=PPFIA	1	100.4	96.5	101.2	109.8	98.45	105.5	1.071609954	0.11839
Q96DP5	MTFMT	Methionyl-tRNA formyltransferase, mitochon	1	97.2	105.5	99.6	117.6	101.35	108.6	1.071534287	0.118688
A0A140LJL2	CBARP	Voltage-dependent calcium channel beta subu	2	100	90.3	103.5	100.4	95.15	101.95	1.071466106	0.118957
A0A0S2Z3H3	SLC25A4	Solute carrier family 25 member 4 isoform 1 (	4	103.9	99	113.9	103.5	101.45	108.7	1.071463775	0.118966
B9EK47	HEATR5B	HEAT repeat containing 5B OS=Homo sapien	1	93	100.3	98.8	108.3	96.65	103.55	1.071391619	0.119252
Q6NXT6	TAPT1	Transmembrane anterior posterior transformat	2	103	95.4	99.8	112.7	99.2	106.25	1.071068548	0.120535
Q96DE0	NUDT16	U8 snoRNA-decapping enzyme OS=Homo sa	3	91.1	96.5	98.8	102.1	93.8	100.45	1.070895522	0.121226
Q9NWM8	FKBP14	Peptidyl-prolyl cis-trans isomerase FKBP14 C	3	94.5	106	103	111.7	100.25	107.35	1.070822943	0.121518
Q8IY81	FTSJ3	pre-rRNA processing protein FTSJ3 OS=Hon	23	94.5	97.7	104.6	101.2	96.1	102.9	1.070759625	0.121772
A0A024QZS4	PPIF	Peptidyl-prolyl cis-trans isomerase OS=Homo	8	100.6	96.2	106.2	104.5	98.4	105.35	1.070630081	0.122294
Q71U36	TUBA1A	Tubulin alpha-1A chain OS=Homo sapiens G	1	87.6	99.3	90.2	109.9	93.45	100.05	1.070626003	0.122311
Q15942	ZYX	Zyxin OS=Homo sapiens GN=ZYX PE=1 SV	11	92.9	102.5	101.8	107.4	97.7	104.6	1.07062436	0.122317
A0A024RD41	RAB23	RAB23, member RAS oncogene family, isofo	6	98	97.9	105.1	104.6	97.95	104.85	1.070444104	0.123047
O43324	EEF1E1	Eukaryotic translation elongation factor 1 epsi	4	106.2	94.2	108.1	106.4	100.2	107.25	1.070359281	0.123391
C9JJN9	VPS8	Vacuolar protein sorting-associated protein 8	1	83.8	109.5	94.8	112.1	96.65	103.45	1.070356958	0.123401
Q9NVH0	EXD2	Exonuclease 3'-5' domain-containing protein 2	6	100.1	93.3	103	104	96.7	103.5	1.070320579	0.123548
Q9UHB7	AFF4	AF4/FMR2 family member 4 OS=Homo sapi	5	97.8	97.1	107.1	101.5	97.45	104.3	1.070292458	0.123663
B4DVA0		cDNA FLJ56039, highly similar to 5-aminole	2	93.3	94.7	97.7	103.5	94	100.6	1.070212766	0.123988
H0Y4K0	UCHL5	Ubiquitin carboxyl-terminal hydrolase isozym	1	103.6	91.7	100.1	108.9	97.65	104.5	1.07014849	0.124251
A0A024RA89	STARD3NL	STARD3 N-terminal like, isoform CRA_a OS	1	100.2	99	99.9	113.2	99.6	106.55	1.069779116	0.125768
Q13277	STX3	Syntaxin-3 OS=Homo sapiens GN=STX3 PE=	3	99.6	95.5	105.7	103	97.55	104.35	1.069707842	0.126063
J9JIE6	TMCO1	Calcium load-activated calcium channel OS=I	4	96.7	99	105.6	103.7	97.85	104.65	1.069494124	0.126949
Q99943	AGPAT1	1-acyl-sn-glycerol-3-phosphate acyltransferas	2	99.5	94.9	92.3	115.6	97.2	103.95	1.069444444	0.127156
P55010	EIF5	Eukaryotic translation initiation factor 5 OS=I	11	97.7	98.3	103.2	106.4	98	104.8	1.069387755	0.127392
Q9UP95	SLC12A4	Solute carrier family 12 member 4 OS=Homo	4	100.5	94.1	96	112.1	97.3	104.05	1.069373073	0.127453
Q8IVW6	ARID3B	AT-rich interactive domain-containing protein	2	97.2	96	96.2	110.4	96.6	103.3	1.069358178	0.127516
A0A024R3D4	DKFZp547C19	Uncharacterized protein OS=Homo sapiens G	1	97	93.5	103.9	99.8	95.25	101.85	1.069291339	0.127795
P21964	COMT	Catechol O-methyltransferase OS=Homo sapi	9	100.1	96.7	106	104.4	98.4	105.2	1.069105691	0.128573
A8K5T0		cDNA FLJ75416, highly similar to Homo sap	1	96.3	98.1	104.5	103.3	97.2	103.9	1.068930041	0.129314
B4DVA7		Beta-hexosaminidase OS=Homo sapiens PE=	1	96	98.6	101.4	106.6	97.3	104	1.068859198	0.129613
Q5VZK9	CARMIL1	F-actin-uncapping protein LRRC16A OS=Ho	1	88.6	94.5	84.5	111.2	91.55	97.85	1.068814855	0.129801
Q53HU8		Vimentin variant (Fragment) OS=Homo sapie	2	113.4	94.5	103	119.2	103.95	111.1	1.068783069	0.129935
P46926	GNPDA1	Glucosamine-6-phosphate isomerase 1 OS=H	5	97.8	98.6	105.1	104.8	98.2	104.95	1.068737271	0.13013
Q68D38	DKFZp686O15	Putative uncharacterized protein DKFZp686C	4	97.4	96.2	104	102.9	96.8	103.45	1.068698347	0.130295
Q16795	NDUFA9	NADH dehydrogenase [ubiquinone] 1 alpha s	13	96.9	99.7	106.7	103.4	98.3	105.05	1.068667345	0.130427
B2R6N9		cDNA, FLJ93042, highly similar to Homo sap	4	98.2	98.8	105	105.5	98.5	105.25	1.068527919	0.13102
P05141	SLC25A5	ADP/ATP translocase 2 OS=Homo sapiens G	6	92.8	95.5	99.3	101.9	94.15	100.6	1.0685077	0.131107
Q9ULG6	CCPG1	Cell cycle progression protein 1 OS=Homo sa	2	105.1	93.6	106.8	105.5	99.35	106.15	1.068444892	0.131375
Q7Z2E3	APTX	Aprataxin OS=Homo sapiens GN=APTX PE=	3	96.1	92.7	98.8	102.9	94.4	100.85	1.068326271	0.131883
A0A0C4DFU2	SOD2	Superoxide dismutase OS=Homo sapiens GN	9	96.7	99.7	105.7	104.1	98.2	104.9	1.068228106	0.132305
A8K683		cDNA FLJ75708, highly similar to Homo sap	2	100.8	97.7	109.3	102.7	99.25	106	1.068010076	0.133245
Q9ULX9	MAFF	Transcription factor MafF OS=Homo sapiens	1	96.5	99.3	109.6	99.5	97.9	104.55	1.067926456	0.133608
A0A024R001	TMEM14C	Transmembrane protein 14C, isoform CRA_a	1	97.3	99.1	105	104.7	98.2	104.85	1.067718941	0.134509
Q9HD33	MRPL47	39S ribosomal protein L47, mitochondrial OS	8	97.4	99.2	98.9	111	98.3	104.95	1.067650051	0.13481
B4DT57		cDNA FLJ61383, highly similar to Serine/thre	1	97.6	87.2	98.6	98.7	92.4	98.65	1.067640693	0.134851
A0A024QZN9	VDAC2	Voltage-dependent anion channel 2, isoform C	14	95.4	95.5	100.4	103.4	95.45	101.9	1.067574646	0.135139
P19256	CD58	Lymphocyte function-associated antigen 3 OS	1	98.6	95.3	102.5	104.5	96.95	103.5	1.067560598	0.135201
A0A024QYY1	EPN2	Epsin 2, isoform CRA_a OS=Homo sapiens C	2	95.9	96.2	103.6	101.4	96.05	102.5	1.067152525	0.136996
P48047	ATP5O	ATP synthase subunit O, mitochondrial OS=H	4	93.3	93	100.5	98.3	93.15	99.4	1.067096082	0.137246
A0A140VK92		Testis secretory sperm-binding protein Li 219	1	95	99.2	100.4	106.8	97.1	103.6	1.066941298	0.137933
A6XMV9	PRSS2	Protease serine 2 preproprotein OS=Homo sap	2	96.4	97.9	101.6	105.7	97.15	103.65	1.066906845	0.138086
O15260	SURF4	Surfeit locus protein 4 OS=Homo sapiens GN	5	95.2	100.6	106	102.9	97.9	104.45	1.066905005	0.138094
O14647	CHD2	Chromodomain-helicase-DNA-binding protei	5	101	95	94	115.1	98	104.55	1.066836735	0.138399
A0A024QZW:	NOL7	Nucleolar protein 7, 27kDa, isoform CRA_a C	6	95.5	97.8	103	103.2	96.65	103.1	1.066735644	0.13885
H0YG38	PRPF40A	Pre-mRNA-processing factor 40 homolog A (	1	93.6	97	103.9	99.4	95.3	101.65	1.066631689	0.139315

Q92879	CELF1	CUGBP Elav-like family member 1 OS=Hom	7	92	103.2	99.6	108.6	97.6	104.1	1.066598361	0.139464
A0A140VJZ4		Ubiquitin carboxyl-terminal hydrolase OS=H	2	99.3	94.5	100	106.7	96.9	103.35	1.066563467	0.139621
A0A024RAE1	C1orf33	Chromosome 1 open reading frame 33, isofor	11	95.7	98.2	104.5	102.3	96.95	103.4	1.066529139	0.139775
B4DWN4		cDNA FLJ52286 OS=Homo sapiens PE=2 SV	1	101.7	94	97.3	111.4	97.85	104.35	1.066428206	0.14023
O75340	PDCD6	Programmed cell death protein 6 OS=Homo s	7	93.7	96.4	100.8	101.9	95.05	101.35	1.066280905	0.140894
B5BU36	TNFRSF10B	Tumor necrosis factor receptor superfamily m	2	102.5	93.9	103.1	106.3	98.2	104.7	1.066191446	0.1413
P35659	DEK	Protein DEK OS=Homo sapiens GN=DEK PI	12	96.6	99	104	104.5	97.8	104.25	1.06595092	0.142394
P84157	MXRA7	Matrix-remodeling-associated protein 7 OS=H	2	99.6	98	107.5	103.1	98.8	105.3	1.065789474	0.143131
A0A024RB01	ITGA5	Integrin, alpha 5 (Fibronectin receptor, alpha	11	101.8	97.6	107.4	105.1	99.7	106.25	1.065697091	0.143555
Q6FGS1	TPD52L2	TPD52L2 protein OS=Homo sapiens GN=TP	11	96.7	99.3	103.4	105.4	98	104.4	1.065306122	0.145359
Q96AG4	LRRC59	Leucine-rich repeat-containing protein 59 OS	15	98.3	99.3	105.7	104.8	98.8	105.25	1.065283401	0.145464
B4DXW9		cDNA FLJ57228, highly similar to Proteasom	1	93.4	104.3	102.4	108.2	98.85	105.3	1.065250379	0.145617
A0A024R3A2	DCUN1D5	DCN1-like protein OS=Homo sapiens GN=D	3	96.9	98.2	105.2	102.6	97.55	103.9	1.065094823	0.146341
A0A024R5Z7	ANXA2	Annexin OS=Homo sapiens GN=ANXA2 PE	26	97.2	99.5	104.5	105	98.35	104.75	1.065073716	0.14644
P06727	APOA4	Apolipoprotein A-IV OS=Homo sapiens GN=	5	109.6	84.2	105.1	101.3	96.9	103.2	1.06501548	0.146712
P30048	PRDX3	Thioredoxin-dependent peroxide reductase, m	12	96.1	96.5	101.5	103.6	96.3	102.55	1.06490135	0.147245
Q16577	PTI-1	Elongation factor 1-alpha 1 OS=Homo sapien	2	91.9	94.7	94.6	104.1	93.3	99.35	1.064844587	0.147511
Q13217	DNAJC3	DnaJ homolog subfamily C member 3 OS=H	14	100.6	96.8	105.3	104.9	98.7	105.1	1.064842958	0.147519
Q96BD0	SLCO4A1	Solute carrier organic anion transporter family	2	98.6	95.8	104	103	97.2	103.5	1.064814815	0.147651
O75935	DCTN3	Dynactin subunit 3 OS=Homo sapiens GN=D	2	97.8	97.7	102.7	105.4	97.75	104.05	1.064450128	0.149371
P0CG39	POTEJ	POTE ankyrin domain family member J OS=	2	90.4	94.6	95.4	101.5	92.5	98.45	1.064324324	0.149968
Q9ULX6	AKAP8L	A-kinase anchor protein 8-like OS=Homo sap	8	100.7	98.3	104	107.8	99.5	105.9	1.064321608	0.149981
Q00765	REEP5	Receptor expression-enhancing protein 5 OS=	6	101.4	98	107.7	104.5	99.7	106.1	1.064192578	0.150596
P38646	HSPA9	Stress-70 protein, mitochondrial OS=Homo sa	41	96	95.8	101.6	102.5	95.9	102.05	1.064129301	0.150897
Q96BZ9	TBC1D20	TBC1 domain family member 20 OS=Homo s	1	98.5	93.5	94.7	109.6	96	102.15	1.0640625	0.151217
P17900	GM2A	Ganglioside GM2 activator OS=Homo sapien	1	92.6	90.5	99.5	95.3	91.55	97.4	1.063899508	0.151998
Q6P4Q7	CNNM4	Metal transporter CNNM4 OS=Homo sapiens	5	99.8	94.4	105.1	101.5	97.1	103.3	1.063851699	0.152228
Q9H8H0	NOL11	Nucleolar protein 11 OS=Homo sapiens GN=	19	96.9	95.8	99.9	105.1	96.35	102.5	1.063829787	0.152334
B4DJL3		cDNA FLJ51219, highly similar to Homo sap	1	92.2	99	106.2	97.2	95.6	101.7	1.063807531	0.152441
A8K9X0		Protein YIPF OS=Homo sapiens PE=2 SV=1	2	98.5	99.1	111.2	99	98.8	105.1	1.063765182	0.152645
P52272	HNRNPM	Heterogeneous nuclear ribonucleoprotein M C	25	95	98	102.3	103	96.5	102.65	1.06373057	0.152811
Q93050	ATP6V0A1	V-type proton ATPase 116 kDa subunit a isof	13	99.2	100.1	107.4	104.6	99.65	106	1.063723031	0.152848
O95104	SCAF4	Splicing factor, arginine/serine-rich 15 OS=H	7	97.3	97.6	102.9	104.4	97.45	103.65	1.06362237	0.153334
O76021	RSL1D1	Ribosomal L1 domain-containing protein 1 O	26	98.1	98.7	104.5	104.8	98.4	104.65	1.06351626	0.153848
P11387	TOP1	DNA topoisomerase 1 OS=Homo sapiens GN	29	97	98.7	104.7	103.4	97.85	104.05	1.063362289	0.154597
Q96DA6	DNAJC19	Mitochondrial import inner membrane translo	4	96.8	99	101.2	107	97.9	104.1	1.063329928	0.154754
Q5H8A4	PIGG	GPI ethanolamine phosphate transferase 2 OS	2	95.4	100.8	107.7	100.9	98.1	104.3	1.063200815	0.155384
Q9UKR5	C14orf1	Probable ergosterol biosynthetic protein 28 O	1	95	99.8	104.6	102.5	97.4	103.55	1.063141684	0.155673
O15173	PGRMC2	Membrane-associated progesterone receptor c	9	96.9	98.1	104.6	102.7	97.5	103.65	1.063076923	0.155991
Q15904	ATP6AP1	V-type proton ATPase subunit S1 OS=Homo	4	98.7	101.3	105.3	107.3	100	106.3	1.063	0.156369
P60602	ROMO1	Reactive oxygen species modulator 1 OS=Ho	2	96.3	102.3	102	109.1	99.3	105.55	1.062940584	0.15666
O14681	EI24	Etoposide-induced protein 2.4 homolog OS=H	1	100.1	97	104.4	105.1	98.55	104.75	1.062912227	0.1568
P30536	TSPO	Translocator protein OS=Homo sapiens GN=	2	96.5	95.9	103.8	100.7	96.2	102.25	1.062889813	0.15691
Q9GZT3	SLIRP	SRA stem-loop-interacting RNA-binding prot	8	96.3	97.8	103.4	102.9	97.05	103.15	1.062854199	0.157086
Q86Y82	STX12	Syntaxin-12 OS=Homo sapiens GN=STX12 I	6	98.6	98.8	104.8	105	98.7	104.9	1.062816616	0.157271
Q6ZT21	TMPPE	Transmembrane protein with metallophospho	1	87.4	96.5	100.5	94.9	91.95	97.7	1.062533986	0.158671
Q10589	BST2	Bone marrow stromal antigen 2 OS=Homo sa	5	99.2	95	101	105.3	97.1	103.15	1.0623069	0.159803
A0A0A0MRJ7	F5	Coagulation factor V OS=Homo sapiens GN=	1	96	120.8	132.9	97.4	108.4	115.15	1.062269373	0.159991
Q96QR8	PURB	Transcriptional activator protein Pur-beta OS	1	113.1	86.2	102.3	109.4	99.65	105.85	1.062217762	0.160249
P30101	PDIA3	Protein disulfide-isomerase A3 OS=Homo sap	32	98.6	99.5	105.3	105.1	99.05	105.2	1.062089854	0.160891
O15440	ABCC5	Multidrug resistance-associated protein 5 OS=	2	94.9	100.1	98.5	108.6	97.5	103.55	1.062051282	0.161084
Q96EB6	SIRT1	NAD-dependent protein deacetylase sirtuin-1	1	92	96.7	98.9	101.5	94.35	100.2	1.06200318	0.161326
A0A024R6D4	ERH	Enhancer of rudimentary homolog OS=Homo	6	91.2	99.2	98.1	104.1	95.2	101.1	1.06197479	0.161469
Q96CM8	ACSF2	Acyl-CoA synthetase family member 2, mitoc	1	95.7	94.9	101.6	100.8	95.3	101.2	1.061909759	0.161797
B2RCJ6		cDNA, FLJ96114, highly similar to Homo sap	10	97.5	97	99.4	107.1	97.25	103.25	1.061696658	0.162875
P23142	FBLN1	Fibulin-1 OS=Homo sapiens GN=FBLN1 PE	2	96.2	96.7	110.4	94.4	96.45	102.4	1.061689995	0.162909
P46459	NSF	Vesicle-fusing ATPase OS=Homo sapiens GN	25	98.6	98.1	103.6	105.2	98.35	104.4	1.061514997	0.163799
Q9NZM1	MYOF	Myoferlin OS=Homo sapiens GN=MYOF PE	67	99.8	97.4	106	103.3	98.6	104.65	1.061359026	0.164595
Q92804	TAF15	TATA-binding protein-associated factor 2N C	5	93.7	103.6	101.9	107.5	98.65	104.7	1.061327927	0.164754
B7WPA5	PLEKHB2	Pleckstrin homology domain-containing famil	2	107.9	89.4	102.3	107.1	98.65	104.7	1.061327927	0.164754
Q15853	USF2	Upstream stimulatory factor 2 OS=Homo sapi	1	96.8	97.3	104	102	97.05	103	1.061308604	0.164853
Q9BQQ3	GORASP1	Golgi reassembly-stacking protein 1 OS=Hon	1	96	91.8	96.9	102.4	93.9	99.65	1.061235357	0.165229
B2R950		cDNA, FLJ94213, highly similar to Homo sap	1	106.1	96.9	106	109.4	101.5	107.7	1.061083744	0.166008
H0YA47	RUFY1	RUN and FYVE domain-containing protein 1	1	101.3	98.7	101.3	110.9	100	106.1	1.061	0.16644
Q9Y3D6	FIS1	Mitochondrial fission 1 protein OS=Homo sap	4	101.4	97.4	105.3	105.6	99.4	105.45	1.060865191	0.167136
Q8NHU6	TDRD7	Tudor domain-containing protein 7 OS=Hom	1	100.8	94.9	120	87.6	97.85	103.8	1.060807358	0.167436
Q9Y2T2	AP3M1	AP-3 complex subunit mu-1 OS=Homo sapie	8	97.8	95.3	96.7	108.1	96.55	102.4	1.060590368	0.168564
Q9H583	HEATR1	HEAT repeat-containing protein 1 OS=Homo	2	93.4	90.2	95.2	99.5	91.8	97.35	1.060457516	0.169257
P99999	CYCS	Cytochrome c OS=Homo sapiens GN=CYCS	8	97.4	100	103.9	105.4	98.7	104.65	1.060283688	0.170168
O00400	SLC33A1	Acetyl-coenzyme A transporter 1 OS=Homo s	5	101.2	93.2	105.2	100.9	97.2	103.05	1.060185185	0.170685
Q8WVK2	SNRNP27	U4/U6.U5 small nuclear ribonucleoprotein 27	2	93.1	101.7	105.1	101.4	97.4	103.25	1.060061602	0.171337
A0A087WWS	THOC1	THO complex subunit 1 OS=Homo sapiens G	19	96.5	97.1	102.2	103	96.8	102.6	1.059917355	0.172099
A0A0D9SG79	UBAP1	Ubiquitin-associated protein 1 (Fragment) OS	2	95.4	85	95.5	95.7	90.2	95.6	1.059866962	0.172367
P23284	PPIB	Peptidyl-prolyl cis-trans isomerase B OS=Ho	13	94.1	98	101.2	102.4	96.05	101.8	1.059864654	0.172379
Q99666	RGPD5	RANBP2-like and GRIP domain-containing p	3	101.3	94.4	103.5	103.9	97.85	103.7	1.059785386	0.172799
A8K8U7		cDNA FLJ78640, highly similar to Homo sap	1	93.9	95.5	93.1	107.6	94.7	100.35	1.059662091	0.173455
Q86SQ9	DHDDS	Dehydrodolichyl diphosphate synthase compl	2	97.1	102.6	107.2	104.4	99.85	105.8	1.059589384	0.173843
Q9H7Z6	KAT8	Histone acetyltransferase KAT8 OS=Homo sa	1	92.5	92.3	99.6	96.2	92.4	97.9	1.05952381	0.174193
Q9C0B7	TANGO6	Transport and Golgi organization protein 6 ho	1	99.1	98.1	96.8	112.1	98.6	104.45	1.059330629	0.175229
Q8N183	NDUFAF2	Mimitin, mitochondrial OS=Homo sapiens GI	9	99.2	96.4	105	102.2	97.8	103.6	1.059304703	0.175368
B2RAH5		Protein phosphatase 1 regulatory subunit OS=	18	100.7	98.7	105.3	105.9	99.7	105.6	1.059177533	0.176052
A0A024QZN6	RAI17	Retinoic acid induced 17, isoform CRA_a OS	1	95.4	107.4	105.5	109.3	101.4	107.4	1.059171598	0.176085
Q13190	STX5	Syntaxin-5 OS=Homo sapiens GN=STX5 PE	3	96.4	98.2	99.6	106.5	97.3	103.05	1.059095581	0.176494
P29317	EPHA2	Ephrin type-A receptor 2 OS=Homo sapiens C	22	102.3	97.6	107.4	104.3	99.95	105.85	1.059029515	0.176852
B2RWN5	HEATR1	HEAT repeat containing 1 OS=Homo sapiens	2	92.7	99.7	102.2	101.5	96.2	101.85	1.058731809	0.178468
P05556	ITGB1	Integrin beta-1 OS=Homo sapiens GN=ITGB	25	98.9	98.8	104.7	104.6	98.85	104.65	1.05867476	0.178778
B4DGM9	TOR1A	Torsin family 1, member A (Torsin A), isofor	7	98.9	98.9	103.1	106.3	98.9	104.7	1.058645096	0.17894
Q8N257	HIST3H2BB	Histone H2B type 3-B OS=Homo sapiens GN	3	90.5	100.6	101.1	101.2	95.55	101.15	1.058608059	0.179143
P52788	SMS	Spermine synthase OS=Homo sapiens GN=SI	5	99.3	98.8	103.9	105.8	99.05	104.85	1.058556285	0.179426
O94826	TOMM70	Mitochondrial import receptor subunit TOM7	28	99.9	96.5	104.9	103	98.2	103.95	1.058553971	0.179439
Q9HBL7	PLGRKT	Plasminogen receptor (KT) OS=Homo sapien	2	100.6	97.8	107.2	102.8	99.2	105	1.058467742	0.179911
Q09666	AHNAK	Neuroblast differentiation-associated protein	223	99.5	99.1	106.2	104	99.3	105.1	1.058408862	0.180234
Q9NZY4		HSPC051 OS=Homo sapiens PE=1 SV=1	2	95.6	99.6	107.6	99	97.6	103.3	1.058401639	0.180274
B7Z6L7		cDNA FLJ61737, moderately similar to Adre	1	104	89.5	110.2	94.6	96.75	102.4	1.058397933	0.180294
A0A0S2Z433	NDUFS4	NADH dehydrogenase (Ubiquinone) Fe-S pro	2	98.8	100	111.9	98.5	99.4	105.2	1.058350101	0.180557
Q9GZR7	DDX24	ATP-dependent RNA helicase DDX24 OS=H	15	96.7	96	102.5	101.4	96.35	101.95	1.058121432	0.181817
Q59GR1		Niemann-Pick disease, type C1 variant (Fragn	6	101.2	96.9	106.1	103.5	99.05	104.8	1.058051489	0.182204
O75942	PRNP	Major prion protein OS=Homo sapiens GN=P	1	103.3	95	107.6	102.2	99.15	104.9	1.05799294	0.182528
B2R6D4		Phosphomannomutase OS=Homo sapiens PE	3	96.1	90.5	90.5	106.9	93.3	98.7	1.057877814	0.183167
Q8IY95	TMEM192	Transmembrane protein 192 OS=Homo sapie	4	98.8	100	109.4	100.9	99.4	105.15	1.057847082	0.183338
A0A024R7J0	PRKACA	Protein kinase, cAMP-dependent, catalytic, al	9	98.4	99.2	103.2	105.8	98.8	104.5	1.057692308	0.184201
O00566	MPHOSPH10	U3 small nucleolar ribonucleoprotein protein	13	100.8	100.4	112.4	100.4	100.6	106.4	1.057654076	0.184414
Q59FL1		Serine/threonine-protein kinase receptor (Frag	1	98.3	89.1	95.9	102.3	93.7	99.1	1.057630736	0.184544
I3L0E3	hCG_1984214	HCG1984214, isoform CRA_a OS=Homo sap	6	98.3	98.3	104.8	103.1	98.3	103.95	1.057477111	0.185405
Q7Z4F1	LRP10	Low-density lipoprotein receptor-related prote	2	102	99.9	96.5	117	100.95	106.75	1.057454185	0.185533
O75947	ATP5H	ATP synthase subunit d, mitochondrial OS=H	10	94.7	97.1	101.5	101.3	95.9	101.4	1.057351408	0.186112
P62879	GNB2	Guanine nucleotide-binding protein G(I)/G(S)	6	95.6	92.9	98.5	100.8	94.25	99.65	1.05729443	0.186432
A0A024R5U8	C15orf15	Chromosome 15 open reading frame 15, isofo	3	98.3	99	102.2	106.4	98.65	104.3	1.057273188	0.186552
O60220	TIMM8A	Mitochondrial import inner membrane translo	5	98.2	97.4	103.1	103.7	97.8	103.4	1.057259714	0.186628
P21333	FLNA	Filamin-A OS=Homo sapiens GN=FLNA PE	111	100.7	100.2	107.3	105.1	100.45	106.2	1.057242409	0.186725
Q5SNT2	TMEM201	Transmembrane protein 201 OS=Homo sapie	3	96.8	97.2	102.7	102.4	97	102.55	1.057216495	0.186871
Q5U091	NRAS	Neuroblastoma RAS viral (V-ras) oncogene h	2	97.5	96.5	100.3	104.8	97	102.55	1.057216495	0.186871
Q59GQ0		Actin related protein 2/3 complex subunit 1A	4	99.1	98.5	102.7	106.2	98.8	104.45	1.057186235	0.187042
P02786	TFRC	Transferrin receptor protein 1 OS=Homo sapi	1	101.9	96.1	102.6	106.7	99	104.65	1.057070707	0.187696
V9HWE1	HEL113	Epididymis luminal protein 113 OS=Homo sa	2	89.9	90.9	94.1	97	90.4	95.55	1.056969027	0.188272
Q9Y3Q8	TSC22D4	TSC22 domain family protein 4 OS=Homo sa	1	95.5	103.1	96.8	113.1	99.3	104.95	1.056898288	0.188675
A0A024R1U4	RAB5C	RAB5C, member RAS oncogene family, isofo	6	101.6	97	104.3	105.6	99.3	104.95	1.056898288	0.188675
P37802	TAGLN2	Transgelin-2 OS=Homo sapiens GN=TAGLN	12	96.3	100.7	105.7	102.5	98.5	104.1	1.056852792	0.188933
Q9BUR5	APOO	MICOS complex subunit MIC26 OS=Homo s	4	99.8	97.6	100.5	108.1	98.7	104.3	1.056737589	0.18959
Q9BSQ5	CCM2	Cerebral cavernous malformations 2 protein C	1	94	91.1	91.9	103.7	92.55	97.8	1.056726094	0.189656
Q96B49	TOMM6	Mitochondrial import receptor subunit TOM6	2	97.9	92.5	95.2	106	95.2	100.6	1.056722689	0.189675
U3KQS4	PXK	PX domain-containing protein kinase-like pro	1	73.2	118	104.4	97.6	95.6	101	1.056485356	0.191034

O43615	TIMM44	Mitochondrial import inner membrane translo	21	97.1	97.8	104.3	101.6	97.45	102.95	1.0564392	0.191299
Q92618	ZNF516	Zinc finger protein 516 OS=Homo sapiens GN	1	102.4	78.5	86.8	104.3	90.45	95.55	1.056384743	0.191612
Q8TB61	SLC35B2	Adenosine 3'-phospho 5'-phosphosulfate trans	4	92.4	106.6	109.2	101	99.5	105.1	1.056281407	0.192207
Q9Y2X3	NOP58	Nucleolar protein 58 OS=Homo sapiens GN=	17	96.1	96.5	101.8	101.6	96.3	101.7	1.056074766	0.193403
B4DI54		cDNA FLJ56386, highly similar to Heat shocl	2	90.1	101	92.1	109.7	95.55	100.9	1.055991627	0.193885
Q9GZP1	NRSN2	Neurensin-2 OS=Homo sapiens GN=NRSN2	3	103	92	100	105.9	97.5	102.95	1.055897436	0.194432
Q86WA8	LONP2	Lon protease homolog 2, peroxisomal OS=Ho	2	95.5	97.8	90.8	113.3	96.65	102.05	1.055871702	0.194582
O75937	DNAJC8	DnaJ homolog subfamily C member 8 OS=Ho	7	97.8	97.3	103.9	102.1	97.55	103	1.055868785	0.194599
Q01081	U2AF1	Splicing factor U2AF 35 kDa subunit OS=Ho	10	96.3	102.5	107.5	102.4	99.4	104.95	1.05583501	0.194796
P81605	DCD	Dermcidin OS=Homo sapiens GN=DCD PE=	2	94.8	99.6	97.5	107.7	97.2	102.6	1.055555556	0.196429
Q8TED1	GPX8	Probable glutathione peroxidase 8 OS=Homo	1	101.6	95.1	103.1	104.5	98.35	103.8	1.055414337	0.197259
A0A024R883	ATP6V1G1	ATPase, H+ transporting, lysosomal 13kDa, V	4	99.3	95.7	102.8	103	97.5	102.9	1.055384615	0.197434
Q9HCG8	CWC22	Pre-mRNA-splicing factor CWC22 homolog (	5	98.5	95.2	98.2	106.2	96.85	102.2	1.055240062	0.198287
A8K509		cDNA FLJ75931, highly similar to Homo sap	1	102.6	100.2	111.5	102.5	101.4	107	1.055226824	0.198365
B2R7B1		cDNA, FLJ93361, highly similar to Homo sap	4	98.7	97.4	106.6	100.3	98.05	103.45	1.055073942	0.19927
X6RLX0	ERC1	ELKS/Rab6-interacting/CAST family membe	2	104.4	91.7	100.8	106.1	98.05	103.45	1.055073942	0.19927
Q9Y394	DHRS7	Dehydrogenase/reductase SDR family membe	13	97.5	96.8	102.9	102.1	97.15	102.5	1.05506948	0.199296
Q9NZI8	IGF2BP1	Insulin-like growth factor 2 mRNA-binding p	20	99.6	102.3	106.2	106.8	100.95	106.5	1.054977712	0.199841
Q9NVG8	TBC1D13	TBC1 domain family member 13 OS=Homo s	2	105.5	89.3	111.4	94.1	97.4	102.75	1.054928131	0.200136
Q53T59	HS1BP3	HCLS1-binding protein 3 OS=Homo sapiens	2	96.8	91	94	104.1	93.9	99.05	1.05484558	0.200627
B4DL31		cDNA FLJ52681, highly similar to HLA class	1	84.4	90	86.8	97.1	87.2	91.95	1.054472477	0.202861
Q9UPT9	USP22	Ubiquitin carboxyl-terminal hydrolase 22 OS	1	104.4	89	94.3	109.6	96.7	101.95	1.054291624	0.20395
Q01167	FOXK2	Forkhead box protein K2 OS=Homo sapiens (	1	97.3	98.6	92.2	114.3	97.95	103.25	1.054109239	0.205053
P23458	JAK1	Tyrosine-protein kinase JAK1 OS=Homo sap	1	100.6	102.9	106.8	107.7	101.75	107.25	1.054054054	0.205388
Q9NRM2	ZNF277	Zinc finger protein 277 OS=Homo sapiens GN	1	91.4	132.5	157.3	78.7	111.95	118	1.054041983	0.205461
P11388	TOP2A	DNA topoisomerase 2-alpha OS=Homo sapie	40	97.5	95.5	102.7	100.7	96.5	101.7	1.05388601	0.206409
Q9H7B2	RPF2	Ribosome production factor 2 homolog OS=H	11	93.4	103.7	105.7	102	98.55	103.85	1.053779807	0.207057
V9HWH9	HEL-S-43	Protein S100 OS=Homo sapiens GN=HEL-S-	5	95.8	97.9	102.7	101.4	96.85	102.05	1.053691275	0.207598
Q969F1	GTF3C6	General transcription factor 3C polypeptide 6	1	99.8	99.5	105	105	99.65	105	1.053687908	0.207618
A8K5R6		Golgi SNAP receptor complex member 1 OS=	5	97.9	95.9	101.1	103.1	96.9	102.1	1.053663571	0.207767
Q6FHX6	FEN1	Flap endonuclease 1 OS=Homo sapiens GN=	13	97.5	98.3	103.4	102.9	97.9	103.15	1.053626149	0.207996
Q9P2N6	KANSL3	KAT8 regulatory NSL complex subunit 3 OS	1	81.1	84.9	84.7	90.2	83	87.45	1.053614458	0.208068
B4DP80	APOA1BP	NAD(P)H-hydrate epimerase OS=Homo sapi	10	93.8	104	114.5	93.9	98.9	104.2	1.053589484	0.208221
A0A096LNX8	SCARF2	Scavenger receptor class F member 2 OS=Ho	1	96.3	96	105	97.6	96.15	101.3	1.053562142	0.208389
Q8IXU6	SLC35F2	Solute carrier family 35 member F2 OS=Hom	3	96.1	96.2	100.3	102.3	96.15	101.3	1.053562142	0.208389
Q8N2H4	SYS1	Protein SYS1 homolog OS=Homo sapiens GN	1	98.6	101.4	101.2	109.5	100	105.35	1.0535	0.20877
B3KY94	CDIPT	CDP-diacylglycerol--inositol 3-phosphatidylt	3	99.2	93.4	104.2	98.7	96.3	101.45	1.053478712	0.208901
B3V096	CSTF2	BetaCstF-64 variant 2 OS=Homo sapiens GN	7	95.7	97	101.2	101.8	96.35	101.5	1.05345096	0.209072
Q59ED5		Tetraspanin (Fragment) OS=Homo sapiens PE	2	100.2	98.2	110.7	98.3	99.2	104.5	1.053427419	0.209216
P19525	EIF2AK2	Interferon-induced, double-stranded RNA-act	12	96.8	99.9	103.8	103.4	98.35	103.6	1.053380783	0.209504
A6NMQ1	POLA1	DNA polymerase OS=Homo sapiens GN=PO	2	89.6	90.7	93.6	96.3	90.15	94.95	1.053244592	0.210344
Q9Y2R0	COA3	Cytochrome c oxidase assembly factor 3 hom	4	98.8	96.7	103.8	102.1	97.75	102.95	1.053196931	0.210639
A8K905		cDNA FLJ77615, highly similar to Homo sap	13	96.3	99.5	105.2	101	97.9	103.1	1.053115424	0.211143
B3KY32		cDNA FLJ46731 fis, clone TRACH3019621,	3	95.3	98.8	102.8	101.6	97.05	102.2	1.05306543	0.211453
Q9Y3P9	RABGAP1	Rab GTPase-activating protein 1 OS=Homo s	3	98.2	92.5	100.8	100	95.35	100.4	1.052962769	0.21209
Q14165	MLEC	Malectin OS=Homo sapiens GN=MLEC PE=	9	99.4	97.5	104.5	102.8	98.45	103.65	1.05281869	0.212988
Q8N5K1	CISD2	CDGSH iron-sulfur domain-containing protei	8	98.1	96.3	102.2	102.4	97.2	102.3	1.052469136	0.215176
B1AHD1	SNU13	NHP2-like protein 1 OS=Homo sapiens GN=	6	95.8	98.6	102.7	101.9	97.2	102.3	1.052469136	0.215176
O75381	PEX14	Peroxisomal membrane protein PEX14 OS=H	3	96.5	96	101.1	101.5	96.25	101.3	1.052467532	0.215186
A0A0G2JNW:	SLC12A7	Solute carrier family 12 member 7 OS=Homo	7	103.7	94.8	103.6	105.3	99.25	104.45	1.052392947	0.215655
J3KPS0	DNAJB12	DnaJ (Hsp40) homolog, subfamily B, member	4	101.7	95.1	106.5	100.6	98.4	103.55	1.052337398	0.216005
Q9NQS7	INCENP	Inner centromere protein OS=Homo sapiens (	8	101.6	97.3	96.8	112.5	99.45	104.65	1.052287582	0.216319
P38606	ATP6V1A	V-type proton ATPase catalytic subunit A OS	20	98	99	104.2	103.1	98.5	103.65	1.052284264	0.216341
O60293	ZFC3H1	Zinc finger C3H1 domain-containing protein (	4	93.5	99.7	96.7	106.6	96.6	101.65	1.052277433	0.216383
Q8WW12	PCNP	PEST proteolytic signal-containing nuclear pr	2	93.5	90.4	97.3	96.2	91.95	96.75	1.052202284	0.216859
O00148	DDX39A	ATP-dependent RNA helicase DDX39A OS=	11	97.5	99.9	103.6	104.1	98.7	103.85	1.052178318	0.21701
A0A087WVP	FAT1	Protocadherin Fat 1 OS=Homo sapiens GN=F	1	99.2	92.5	104.4	97.3	95.85	100.85	1.052164841	0.217095
A8K594		cDNA FLJ77256, highly similar to Homo sap	1	109.9	91.5	104.9	107	100.7	105.95	1.052135055	0.217283
Q4VC31	CCDC58	Coiled-coil domain-containing protein 58 OS	8	99.2	98.6	105.4	102.7	98.9	104.05	1.052072801	0.217678
H7C1N3	BET1	BET1 homolog (Fragment) OS=Homo sapien	1	103.4	96.9	99.1	111.6	100.15	105.35	1.051922117	0.218635
A0A024RCJ0	DDR1	Discoidin domain receptor family, member 1,	3	102.8	92.1	104.7	100.3	97.45	102.5	1.051821447	0.219276
E5KNY5	LRPPRC	Leucine-rich PPR-motif containing OS=Hom	84	98.6	98.4	104	103.2	98.5	103.6	1.05177665	0.219562
B4DJ79		cDNA FLJ55847 OS=Homo sapiens PE=2 SV	1	90.9	110.4	110.7	101	100.65	105.85	1.051664183	0.22028
O75882	ATRN	Attractin OS=Homo sapiens GN=ATRN PE=	1	92	103.7	100.3	105.5	97.85	102.9	1.051609607	0.220629
P84085	ARF5	ADP-ribosylation factor 5 OS=Homo sapiens	5	98.6	97.3	103.1	102.9	97.95	103	1.051556917	0.220967
A0A024R5Y1	SPG21	Spastic paraplegia 21 (Autosomal recessive, M	2	106.8	93	105.6	104.5	99.9	105.05	1.051551552	0.221001
Q92466	DDB2	DNA damage-binding protein 2 OS=Homo sa	1	92.2	82.7	83.5	100.4	87.45	91.95	1.051457976	0.221602
D3DQU2	TPP1	Tripeptidyl peptidase I, isoform CRA_a OS=I	3	93.9	100.6	102.7	101.8	97.25	102.25	1.051413882	0.221886
A0A024R601	CLN6	Ceroid-lipofuscinosis neuronal 6 late infantile	1	104.4	96	94.6	116.1	100.2	105.35	1.051397206	0.221993
B4DNR0		cDNA FLJ60315 OS=Homo sapiens PE=2 SV	1	95.4	97.3	97	105.6	96.35	101.3	1.051375195	0.222135
Q9UHQ9	CYB5R1	NADH-cytochrome b5 reductase 1 OS=Homo	8	100.6	98	106.5	102.3	99.3	104.4	1.051359517	0.222235
B2RAK1		cDNA, FLJ94965, highly similar to Homo sap	7	98.3	100.4	104.3	104.6	99.35	104.45	1.051333669	0.222402
D3DP46	SPCS3	Signal peptidase complex subunit 3 homolog (	5	99.3	95.7	103.7	101.3	97.5	102.5	1.051282051	0.222735
H0Y4E8	SIK3	Serine/threonine-protein kinase SIK3 (Fragme	2	90	107.5	103.5	104.1	98.75	103.8	1.051139241	0.223657
Q96ES7	SGF29	SAGA-associated factor 29 OS=Homo sapien	2	98.4	101.4	106.1	103.9	99.9	105	1.051051051	0.224229
Q9Y4L1	HYOU1	Hypoxia up-regulated protein 1 OS=Homo saj	43	99.2	98.7	103.1	104.9	98.95	104	1.051035877	0.224327
Q5TEU4	NDUFAF5	Arginine-hydroxylase NDUFAF5, mitochond	1	99.3	98.8	99.7	108.5	99.05	104.1	1.050984351	0.224661
Q9Y639	NPTN	Neuroplastin OS=Homo sapiens GN=NPTN I	5	100	98.1	105.5	102.7	99.05	104.1	1.050984351	0.224661
J3KPT4	TRABD	TraB domain-containing protein OS=Homo sa	7	98.3	97.9	103.6	102.6	98.1	103.1	1.0509684	0.224765
Q59H06		Transporter 2, ATP-binding cassette, sub-fam	4	96.8	95.8	101.9	100.5	96.3	101.2	1.050882658	0.225322
O43772	SLC25A20	Mitochondrial carnitine/acylcarnitine carrier p	3	96.7	96	100.9	101.6	96.35	101.25	1.050856253	0.225494
Q7LGC8	CHST3	Carbohydrate sulfotransferase 3 OS=Homo sa	1	95.1	97.9	100.4	102.4	96.5	101.4	1.050777202	0.226009
Q14728	MFSD10	Major facilitator superfamily domain-containi	2	101	94.4	107	98.3	97.7	102.65	1.050665302	0.22674
Q8NI08	NCOA7	Nuclear receptor coactivator 7 OS=Homo sap	1	100.5	98.9	107	102.5	99.7	104.75	1.050651956	0.226827
B7ZM99	MTHFD1L	MTHFD1L protein OS=Homo sapiens GN=M	27	99.4	98.6	103.9	104.1	99	104	1.050505051	0.227789
Q8TB52	FBXO30	F-box only protein 30 OS=Homo sapiens GN	1	91.5	96.9	103.5	94.4	94.2	98.95	1.050424628	0.228317
B2R6F3	SFRS3	Splicing factor arginine/serine-rich 3 OS=Hor	9	93.1	99.4	97.8	104.4	96.25	101.1	1.05038961	0.228547
B4DS79		cDNA FLJ56828, highly similar to Adapter-re	1	101.2	105.5	114.9	102.2	103.35	108.55	1.050314465	0.229042
P34949	MPI	Mannose-6-phosphate isomerase OS=Homo s	3	95.5	97.3	95.9	106.6	96.4	101.25	1.050311203	0.229063
Q13740	ALCAM	CD166 antigen OS=Homo sapiens GN=ALCA	6	102.9	99.9	108.3	104.7	101.4	106.5	1.050295858	0.229164
Q8WXD5	GEMIN6	Gem-associated protein 6 OS=Homo sapiens (	2	91.3	97.8	95.2	103.4	94.55	99.3	1.050237969	0.229545
Q4G176	ACSF3	Acyl-CoA synthetase family member 3, mitoc	3	98.3	95.6	103.9	99.7	96.95	101.8	1.050025786	0.230948
Q9P013	CWC15	Spliceosome-associated protein CWC15 hom	4	98.4	102	105.7	104.7	100.2	105.2	1.0499002	0.231782
Q96AQ6	PBXIP1	Pre-B-cell leukemia transcription factor-intera	2	103.6	93	102.5	103.9	98.3	103.2	1.049847406	0.232133
A0A1B0GTW	TJP2	Tight junction protein ZO-2 OS=Homo sapier	11	95.6	101.8	102.3	104.9	98.7	103.6	1.04964539	0.233479
A5PLN4	SF4	Splicing factor 4 OS=Homo sapiens GN=SF4	7	97.4	100.3	100.6	106.9	98.85	103.75	1.049570056	0.233983
Q99828	CIB1	Calcium and integrin-binding protein 1 OS=H	1	103.8	90	103.4	100	96.9	101.7	1.049535604	0.234214
Q6FII1	LOC51064	Glutathione S-transferase kappa 1 OS=Homo	9	95.1	96.7	101.9	99.4	95.9	100.65	1.049530761	0.234246
A0A024R7S3	CLTB	Clathrin, light polypeptide (Lcb), isoform CR.	8	102.4	99.5	108	103.9	100.95	105.95	1.04952947	0.234255
P51991	HNRNPA3	Heterogeneous nuclear ribonucleoprotein A3 (	3	91.9	100	100.7	100.7	95.95	100.7	1.04950495	0.234419
Q15417	CNN3	Calponin-3 OS=Homo sapiens GN=CNN3 PE	8	96.4	99.6	101.8	103.9	98	102.85	1.049489796	0.234521
Q6IN85	PPP4R3A	Serine/threonine-protein phosphatase 4 regula	5	100	98.6	109.7	98.7	99.3	104.2	1.049345418	0.23549
L7N2F9		Uncharacterized protein (Fragment) OS=Hom	1	97.1	95.5	97.8	104.3	96.3	101.05	1.049325026	0.235627
P53801	PTTG1IP	Pituitary tumor-transforming gene 1 protein-i	1	98.6	104.4	107.6	105.4	101.5	106.5	1.049261084	0.236057
O95881	TXNDC12	Thioredoxin domain-containing protein 12 OS	6	98.6	100.4	103.5	105.3	99.5	104.4	1.049246231	0.236158
L0R6Q1	SLC35A4	Alternative protein SLC35A4 OS=Homo sapi	4	98.9	98.1	102.9	103.8	98.5	103.35	1.049238579	0.236209
B0UZZ8	WDR46	Chromosome 6 open reading frame 11 OS=H	14	95.5	95.9	98.8	102	95.7	100.4	1.049111808	0.237064
B4DW33		cDNA FLJ54187, highly similar to Homo sap	2	101.4	94.2	103.1	102.1	97.8	102.6	1.049079755	0.237281
B2RNB2	ADAM17	ADAM metallopeptidase domain 17 OS=Hon	3	100	99.8	103.8	105.8	99.9	104.8	1.049049049	0.237489
F2YHL7	APOBEC3F	Apolipoprotein B mRNA editing enzyme cyti	1	105.1	92.7	95.1	112.4	98.9	103.75	1.049039434	0.237553
P27449	ATP6V0C	V-type proton ATPase 16 kDa proteolipid sub	1	96	99.8	104.9	100.5	97.9	102.7	1.049029622	0.23762
F5GYQ1	ATP6V0D1	V-type proton ATPase subunit d 1 OS=Homo	13	101.1	96.9	104.7	103	99	103.85	1.048989899	0.237889
Q9P0L0	VAPA	Vesicle-associated membrane protein-associat	12	97.5	101.4	105.5	103.1	99.45	104.3	1.048768225	0.239394
B4E1U9		cDNA FLJ54776, highly similar to Cell divisi	7	97.1	97.7	103.4	100.9	97.4	102.15	1.048767967	0.239396
J3KMZ9	LDLR	Low-density lipoprotein receptor (Fragment) (	4	104.7	100.4	105.5	109.6	102.55	107.55	1.048756704	0.239473
Q6IAD8	SLC39A1	SLC39A1 protein OS=Homo sapiens GN=SL	2	107.2	89.7	101.7	104.8	98.45	103.25	1.048755714	0.239479
Q8NF37	LPCAT1	Lysophosphatidylcholine acyltransferase 1 OS	8	98.9	96	103.7	100.7	97.45	102.2	1.048742945	0.239567
Q9Y2W1	THRAP3	Thyroid hormone receptor-associated protein	17	96.7	98.4	102.9	101.7	97.55	102.3	1.048692978	0.239907
A0A024R8V6	USP36	Ubiquitin specific peptidase 36, isoform CRA	4	97.7	89.2	95.6	100.4	93.45	98	1.048689139	0.239933
A0A024RAR8	ARTS-1	Type 1 tumor necrosis factor receptor sheddin	24	98.1	99.4	104.4	102.7	98.75	103.55	1.048607595	0.240489
V9HW00	HEL-S-77p	Epididymis secretory sperm binding protein L	7	94.8	99.1	100.9	102.4	96.95	101.65	1.048478597	0.241371
P36871	PGM1	Phosphoglucomutase-1 OS=Homo sapiens GN	5	97.5	106.9	120.2	94.1	102.2	107.15	1.048434442	0.241673

A0A024RBE8	SLC25A3	Solute carrier family 25 (Mitochondrial carrie	17	93.5	99.5	101	101.3	96.5	101.15	1.048186528	0.243377
B7Z213		cDNA FLJ50130, highly similar to Heterogen	1	95.6	95.7	103.5	97	95.65	100.25	1.048092002	0.244029
P11047	LAMC1	Laminin subunit gamma-1 OS=Homo sapiens	28	101.9	95.7	104.5	102.6	98.8	103.55	1.048076923	0.244134
B2RBM7		cDNA, FLJ95595, highly similar to Homo sap	19	99	100.7	103.7	105.6	99.85	104.65	1.048072108	0.244166
V9HWE0	HEL-S-7	Annexin OS=Homo sapiens GN=HEL-S-7 PE	17	97.4	98.2	102.9	102.1	97.8	102.5	1.04805726	0.244269
O95167	NDUFA3	NADH dehydrogenase [ubiquinone] 1 alpha s	2	98.7	94.9	101.3	101.6	96.8	101.45	1.04803719	0.244408
Q8NE01	CNNM3	Metal transporter CNNM3 OS=Homo sapiens	3	100.6	101.4	101.6	110.1	101	105.85	1.048019802	0.244528
Q6LES2	ANXA4	Annexin (Fragment) OS=Homo sapiens GN=.	7	93.7	94.3	95.5	101.5	94	98.5	1.04787234	0.245549
A8K9A4		cDNA FLJ75154, highly similar to Homo sap	1	101.8	96.7	101.1	106.9	99.25	104	1.047858942	0.245642
P09661	SNRPA1	U2 small nuclear ribonucleoprotein A' OS=Ho	11	96.9	99.7	101.2	104.8	98.3	103	1.047812818	0.245962
A0A024R957	TOR1AIP2	Torsin A interacting protein 2, isoform CRA_	5	100.7	96.1	103.8	102.4	98.4	103.1	1.047764228	0.2463
Q5T3I0	GPATCH4	G patch domain-containing protein 4 OS=Hor	11	95.7	97.1	102.8	99.2	96.4	101	1.047717842	0.246622
B3KN76		cDNA FLJ13834 fis, clone THYRO1000684,	1	89	100.1	100.2	97.9	94.55	99.05	1.047593866	0.247486
P13073	COX4I1	Cytochrome c oxidase subunit 4 isoform 1, m	8	97.4	95.1	100.2	101.4	96.25	100.8	1.047272727	0.249733
Q9Y3A2	UTP11	Probable U3 small nucleolar RNA-associated	9	98.5	98.4	103.9	102.3	98.45	103.1	1.047232098	0.250017
V9HW88	HEL-S-99n	Calreticulin, isoform CRA_b OS=Homo sapie	23	94.6	96.5	98.2	101.9	95.55	100.05	1.047095761	0.250977
Q03188	CENPC	Centromere protein C OS=Homo sapiens GN=	1	106.4	95.5	90.8	120.6	100.95	105.7	1.047052997	0.251279
H0Y886	NDUFB5	NADH dehydrogenase [ubiquinone] 1 beta su	3	94.5	97.4	101.2	99.7	95.95	100.45	1.046899427	0.252363
Q9NV96	TMEM30A	Cell cycle control protein 50A OS=Homo sap	4	98.3	100.1	105.6	102.1	99.2	103.85	1.046875	0.252536
B2R9K5		cDNA, FLJ94435 OS=Homo sapiens PE=2 S	3	99.3	93.1	100.4	101	96.2	100.7	1.046777547	0.253226
Q6IAW5	CALU	CALU protein OS=Homo sapiens GN=CALU	4	104	99.2	106.8	105.9	101.6	106.35	1.046751969	0.253408
P36507	MAP2K2	Dual specificity mitogen-activated protein kin	3	94.5	93.9	97	100.2	94.2	98.6	1.04670913	0.253712
P40763	STAT3	Signal transducer and activator of transcriptio	2	100.7	96.4	98.1	108.2	98.55	103.15	1.046676814	0.253942
Q9Y3B9	RRP15	RRP15-like protein OS=Homo sapiens GN=R	5	95.1	102	101.1	105.2	98.55	103.15	1.046676814	0.253942
F8VX04	SLC38A1	Sodium-coupled neutral amino acid transporte	4	102.1	93	105.5	98.7	97.55	102.1	1.046642747	0.254184
P61011	SRP54	Signal recognition particle 54 kDa protein OS	17	101.9	100	104.8	106.5	100.95	105.65	1.046557702	0.25479
A0A024RDJ3	NOLA1	Nucleolar protein family A, member 1 (H/AC	3	97.6	98.3	102.5	102.5	97.95	102.5	1.046452272	0.255541
P37108	SRP14	Signal recognition particle 14 kDa protein OS	9	96.7	101.4	100.8	106.5	99.05	103.65	1.046441191	0.25562
P54920	NAPA	Alpha-soluble NSF attachment protein OS=H	12	99.6	98.6	103.7	103.7	99.1	103.7	1.04641776	0.255788
O94919	ENDOD1	Endonuclease domain-containing 1 protein OS	8	99.4	95.1	99.8	103.7	97.25	101.75	1.046272494	0.256827
Q9Y512	SAMM50	Sorting and assembly machinery component 5	11	98.9	98.2	99.6	106.6	98.55	103.1	1.046169457	0.257567
Q9P035	HACD3	Very-long-chain (3R)-3-hydroxyacyl-CoA de	9	99.5	96	102.1	102.4	97.75	102.25	1.046035806	0.258528
A0A024R2Q9	OXSM	3-oxoacyl-[acyl-carrier-protein] synthase, mit	2	103.8	98.5	106.8	104.8	101.15	105.8	1.04597133	0.258992
A8K2G0		cDNA FLJ76605, highly similar to Homo sap	5	98.8	97.2	101	104	98	102.5	1.045918367	0.259374
P37235	HPCAL1	Hippocalcin-like protein 1 OS=Homo sapiens	4	99.3	94.9	101.9	101.2	97.1	101.55	1.045829042	0.260019
Q10713	PMPCA	Mitochondrial-processing peptidase subunit al	19	96.5	100.5	103.3	102.7	98.5	103	1.045685279	0.26106
Q9C0D9	EPT1	Ethanolaminephosphotransferase 1 OS=Homo	1	101.1	93.9	103.7	100.2	97.5	101.95	1.045641026	0.261381
O14828	SCAMP3	Secretory carrier-associated membrane protei	9	99.3	98.2	99.3	107.2	98.75	103.25	1.04556962	0.261899
P55084	HADHB	Trifunctional enzyme subunit beta, mitochonc	27	99.6	98	104.1	102.5	98.8	103.3	1.045546559	0.262068
Q13144	EIF2B5	Translation initiation factor eIF-2B subunit ep	5	100.8	95	97.4	107.3	97.9	102.35	1.045454545	0.262737
B8ZZD4	TAX1BP1	Tax1-binding protein 1 OS=Homo sapiens GN	3	103	97.3	105.9	103.5	100.15	104.7	1.045431852	0.262903
Q8N353	TMEM106B	TMEM106B protein (Fragment) OS=Homo sa	4	100.3	95.6	103	101.8	97.95	102.4	1.045431343	0.262906
O43920	NDUFS5	NADH dehydrogenase [ubiquinone] iron-sulfi	4	100	98.6	105.1	102.5	99.3	103.8	1.045317221	0.263739
Q16630	CPSF6	Cleavage and polyadenylation specificity fact	11	97.1	97.2	101.6	101.5	97.15	101.55	1.045290787	0.263932
P54198	HIRA	Protein HIRA OS=Homo sapiens GN=HIRA	3	98.9	97.7	104.6	100.9	98.3	102.75	1.045269583	0.264087
A0A024QZW3	NUP153	Nucleoporin 153kDa, isoform CRA_a OS=Ho	31	98.5	98.2	103.8	101.8	98.35	102.8	1.045246568	0.264255
A7XZE4	TPM2b	Beta tropomyosin isoform OS=Homo sapiens	2	92.4	97.9	93.3	105.6	95.15	99.45	1.045191802	0.264657
E5KTA5	UNG	Uracil-DNA glycosylase OS=Homo sapiens C	1	93.6	92.9	101.1	93.8	93.25	97.45	1.045040214	0.265768
A0A024RCY7	C6orf64	Chromosome 6 open reading frame 64, isofor	1	103.3	92.1	96.5	107.7	97.7	102.1	1.045035824	0.2658
Q9HAV7	GRPEL1	GrpE protein homolog 1, mitochondrial OS=H	14	99.4	98.8	104.7	102.4	99.1	103.55	1.044904137	0.266769
A0A024R1N4	XRCC6	X-ray repair complementing defective repair i	32	96.6	99.4	102.8	102	98	102.4	1.044897959	0.266815
O43776	NARS	Asparagine--tRNA ligase, cytoplasmic OS=H	11	95	98.8	100.3	102.2	96.9	101.25	1.044891641	0.266862
P11233	RALA	Ras-related protein Ral-A OS=Homo sapiens	3	102.5	98	109.2	100.3	100.25	104.75	1.044887781	0.26689
Q04206	RELA	Transcription factor p65 OS=Homo sapiens G	6	100.9	95.2	94.3	110.6	98.05	102.45	1.044875064	0.266984
Q9NQ50	MRPL40	39S ribosomal protein L40, mitochondrial OS	6	95.5	98.6	98	104.8	97.05	101.4	1.044822257	0.267373
Q86U42	PABPN1	Polyadenylate-binding protein 2 OS=Homo sa	7	100.8	97.8	106.5	101	99.3	103.75	1.044813696	0.267436
O15061	SYNM	Synemin OS=Homo sapiens GN=SYNM PE=	32	101.6	97.1	103	104.6	99.35	103.8	1.044791142	0.267603
Q59E89		DnaJ (Hsp40) homolog, subfamily B, member	2	92.9	101.5	103.7	99.4	97.2	101.55	1.044753086	0.267884
Q9C0D5	TANC1	Protein TANC1 OS=Homo sapiens GN=TAN	1	95.9	96.3	102.1	98.7	96.1	100.4	1.044745057	0.267943
H7C3C4	SLC4A7	Anion exchange protein (Fragment) OS=Hom	2	94.5	97.8	94.3	106.6	96.15	100.45	1.044721789	0.268114
P09622	DLD	Dihydrolipoyl dehydrogenase, mitochondrial (	20	98.6	98.2	100.2	105.4	98.4	102.8	1.044715447	0.268162
Q9UJX5	ANAPC4	Anaphase-promoting complex subunit 4 OS=I	1	123.7	95.6	115.2	113.9	109.65	114.55	1.044687642	0.268367
Q6IAN0	DHRS7B	Dehydrogenase/reductase SDR family membe	4	97.4	99.6	102.8	103	98.5	102.9	1.044670051	0.268497
O96011	PEX11B	Peroxisomal membrane protein 11B OS=Hom	7	95.6	94.8	99.7	99.2	95.2	99.45	1.044642857	0.268699
E9KL44		Epididymis tissue sperm binding protein Li 14	35	98.2	97.5	102.5	101.9	97.85	102.2	1.0444558	0.270087
O15498	YKT6	Synaptobrevin homolog YKT6 OS=Homo sap	7	100	95.8	100.6	103.9	97.9	102.25	1.044433095	0.270255
Q9P032	NDUFAF4	NADH dehydrogenase [ubiquinone] 1 alpha s	5	96.8	94.7	98.3	101.7	95.75	100	1.044386423	0.270603
Q9BRR6	ADPGK	ADP-dependent glucokinase OS=Homo sapie	7	98.8	100.3	102.9	105	99.55	103.95	1.044198895	0.272001
B3KM95		Phosphatidate cytidylyltransferase OS=Homo	1	96.8	95.6	99.2	101.7	96.2	100.45	1.044178794	0.27215
O75934	BCAS2	Pre-mRNA-splicing factor SPF27 OS=Homo	12	99.3	97.9	100.7	105.2	98.6	102.95	1.044117647	0.272608
Q14690	PDCD11	Protein RRP5 homolog OS=Homo sapiens GN	54	96.5	98.7	101.9	101.9	97.6	101.9	1.044057377	0.273059
Q9BX67	JAM3	Junctional adhesion molecule C OS=Homo sa	3	102.1	97.7	101.9	106.7	99.9	104.3	1.044044044	0.273159
P82673	MRPS35	28S ribosomal protein S35, mitochondrial OS	5	98.1	95.1	100.4	101.3	96.6	100.85	1.043995859	0.27352
P60866	RPS20	40S ribosomal protein S20 OS=Homo sapiens	5	98.1	102	104.2	104.7	100.05	104.45	1.043978011	0.273654
Q14687	GSE1	Genetic suppressor element 1 OS=Homo sapi	2	90.8	105.1	102.1	102.4	97.95	102.25	1.043899949	0.274241
P23381	WARS	Tryptophan--tRNA ligase, cytoplasmic OS=H	11	97.6	101.2	104.1	103.4	99.4	103.75	1.043762575	0.275274
A0A024R6W0	GOT2	Aspartate aminotransferase OS=Homo sapien	23	96.6	97.9	101.9	101.1	97.25	101.5	1.043701799	0.275732
A0A087WUF6	FGF2	Fibroblast growth factor OS=Homo sapiens G	2	99.4	102.3	103.7	106.8	100.85	105.25	1.043629152	0.27628
B7ZLJ0	DIAPH2	DIAPH2 protein OS=Homo sapiens GN=DIA	1	102.1	106.7	110.1	107.8	104.4	108.95	1.043582375	0.276634
O00483	NDUFA4	Cytochrome c oxidase subunit NDUFA4 OS=	5	102.5	90.4	104.9	96.4	96.45	100.65	1.043545879	0.27691
O75390	CS	Citrate synthase, mitochondrial OS=Homo sap	11	97.1	98.5	101.9	102.2	97.8	102.05	1.043456033	0.27759
P0DJ07	PET100	Protein PET100 homolog, mitochondrial OS=	1	96.8	104	105.3	104.2	100.4	104.75	1.043326693	0.278572
Q6I9V5	SLC25A6	SLC25A6 protein OS=Homo sapiens GN=SL	5	95.5	93.8	96.2	101.3	94.65	98.75	1.043317485	0.278642
Q13443	ADAM9	Disintegrin and metalloproteinase domain-cor	7	102.9	95.8	105.1	102.2	99.35	103.65	1.043281329	0.278917
Q5JTV8	TOR1AIP1	Torsin-1A-interacting protein 1 OS=Homo sa	16	99.1	97.8	103.4	102	98.45	102.7	1.043169121	0.279771
P49458	SRP9	Signal recognition particle 9 kDa protein OS=	7	97.1	100.3	103.6	102.3	98.7	102.95	1.043059777	0.280605
Q96D31	ORAI1	Calcium release-activated calcium channel pro	1	104.3	88.5	100.8	100.3	96.4	100.55	1.043049793	0.280682
Q9NRR3	CDC42SE2	CDC42 small effector protein 2 OS=Homo sa	1	98.8	98.7	105.5	100.5	98.75	103	1.043037975	0.280772
A0A0A0MRM	NOLC1	Nucleolar and coiled-body phosphoprotein 1 (	20	97.2	100.5	104.3	101.9	98.85	103.1	1.042994436	0.281105
Q16626	MEA1	Male-enhanced antigen 1 OS=Homo sapiens (	2	87.8	101	93.9	103	94.4	98.45	1.042902542	0.281808
A0A1C7CYX	ELMSAN1	ELM2 and SANT domain-containing protein	3	96.4	99.5	102.4	101.9	97.95	102.15	1.04287902	0.281989
O94905	ERLIN2	Erlin-2 OS=Homo sapiens GN=ERLIN2 PE=	10	100.5	97.8	104	102.8	99.15	103.4	1.042864347	0.282101
P21283	ATP6V1C1	V-type proton ATPase subunit C 1 OS=Homo	8	98	95.7	102.8	99.2	96.85	101	1.042849768	0.282213
Q9BYG3	NIFK	MKI67 FHA domain-interacting nucleolar pho	17	96.8	97.1	101.5	100.7	96.95	101.1	1.04280557	0.282552
Q8N5N7	MRPL50	39S ribosomal protein L50, mitochondrial OS	5	99	94.9	100.7	101.5	96.95	101.1	1.04280557	0.282552
P27824	CANX	Calnexin OS=Homo sapiens GN=CANX PE=	13	100.6	98.2	104.6	102.7	99.4	103.65	1.042756539	0.282928
A4D2P0	RAC1	Ras-related C3 botulinum toxin substrate 1 (R	5	99.2	102.3	104.7	105.4	100.75	105.05	1.042679901	0.283518
B4DF88		cDNA FLJ60464, highly similar to Homo sap	1	95.7	96.5	103.3	97.1	96.1	100.2	1.042663892	0.283641
Q9NW13	RBM28	RNA-binding protein 28 OS=Homo sapiens C	24	97.3	97.4	102	101	97.35	101.5	1.042629687	0.283904
A8K0D5		cDNA FLJ75952, highly similar to Homo sap	1	99.9	92.5	94.2	106.4	96.2	100.3	1.042619543	0.283982
B3KRJ9		cDNA FLJ34439 fis, clone HLUNG2001146,	9	97.4	102.1	104.5	103.5	99.75	104	1.042606516	0.284083
P46776	RPL27A	60S ribosomal protein L27a OS=Homo sapier	6	94.9	104.8	107.6	100.6	99.85	104.1	1.042563846	0.284412
Q6IQ49	SDE2	Protein SDE2 homolog OS=Homo sapiens GI	1	96.2	101.2	95.5	110.3	98.7	102.9	1.042553191	0.284494
Q9H936	SLC25A22	Mitochondrial glutamate carrier 1 OS=Homo	9	98.6	96.5	104.2	99.2	97.55	101.7	1.042542286	0.284578
Q96Q15	SMG1	Serine/threonine-protein kinase SMG1 OS=H	4	96.9	96	98.7	102.4	96.45	100.55	1.042509072	0.284835
A0A024R0H2	MRPS12	Mitochondrial ribosomal protein S12, isoform	2	92.6	95.7	96.7	99.6	94.15	98.15	1.042485396	0.285018
P55081	MFAP1	Microfibrillar-associated protein 1 OS=Homo	5	97.9	98.6	101.9	102.9	98.25	102.4	1.042239186	0.286924
Q6P1X5	TAF2	Transcription initiation factor TFIID subunit 2	9	102	97	99.5	107.9	99.5	103.7	1.042211055	0.287142
Q14TF0	GCLC	Glutamate-cysteine ligase OS=Homo sapiens	1	92.8	96.9	88.5	109.2	94.85	98.85	1.04217185	0.287447
Q96A57	TMEM230	Transmembrane protein 230 OS=Homo sapien	3	100.5	96.6	99.5	105.9	98.55	102.7	1.042110604	0.287923
B4DX69		cDNA FLJ55158, highly similar to Thioredox	4	99	93.5	101.9	98.7	96.25	100.3	1.042077922	0.288177
P42126	ECI1	Enoyl-CoA delta isomerase 1, mitochondrial (	7	97.3	97.7	101.4	101.8	97.5	101.6	1.042051282	0.288384
P61604	HSPE1	10 kDa heat shock protein, mitochondrial OS=	10	95	92.9	96.8	99	93.95	97.9	1.04204364	0.288444
A0A024QZ31	LOC130074	Uncharacterized protein OS=Homo sapiens G	1	97	107.7	100.6	112.7	102.35	106.65	1.042012702	0.288685
Q86W92	PPFIBP1	Liprin-beta-1 OS=Homo sapiens GN=PPFIBI	7	101.3	96.6	99.3	106.9	98.95	103.1	1.041940374	0.28925
Q6P1A2	LPCAT3	Lysophospholipid acyltransferase 5 OS=Hom	3	97.2	100.8	110.9	95.4	99	103.15	1.041919192	0.289414
Q8IYV2	DDX20	DEAD (Asp-Glu-Ala-Asp) box polypeptide 2	3	100.2	100.6	101.7	107.5	100.4	104.6	1.041832669	0.290091
B2RDG9		cDNA, FLJ96603, highly similar to Homo sap	3	96.6	99.5	100.2	104.1	98.05	102.15	1.0418154	0.290226
Q86UE8	TLK2	Serine/threonine-protein kinase tousled-like 2	2	97.9	96.2	91.2	111	97.05	101.1	1.041731066	0.290886
B2R5N4		cDNA, FLJ92544, highly similar to Homo sap	6	100.1	98.9	104.6	102.7	99.5	103.65	1.041708543	0.291062
E9PR17	CD59	CD59 glycoprotein OS=Homo sapiens GN=C	4	99.6	99.5	104.5	102.9	99.55	103.7	1.041687594	0.291227
I3L448	ATPAF1	ATP synthase mitochondrial F1 complex asse	5	93.6	98.5	98.3	101.8	96.05	100.05	1.041644977	0.291562

Q9H7Z7	PTGES2	Prostaglandin E synthase 2 OS=Homo sapiens	15	94.8	95	98.4	99.3	94.9	98.85	1.041622761	0.291736
P61960	UFM1	Ubiquitin-fold modifier 1 OS=Homo sapiens	4	97.5	98	93.6	110	97.75	101.8	1.041432225	0.293235
I1VE18	SEC22B	SEC22 vesicle trafficking protein B (Fragmen	2	99	99.5	103.3	103.4	99.25	103.35	1.041309824	0.294201
Q86Y39	NDUFA11	NADH dehydrogenase [ubiquinone] 1 alpha s	3	99.4	96.9	101.2	103.2	98.15	102.2	1.041263372	0.294568
A0A140VKC4	MECP2	Methyl CpG binding protein 2 (Rett syndrome	6	98.8	100.1	105.8	101.3	99.45	103.55	1.041226747	0.294857
Q8WWV3	RTN4IP1	Reticulon-4-interacting protein 1, mitochondr	3	98.6	97.9	104.5	100.1	98.25	102.3	1.041221374	0.2949
A0A024R169	TEX10	Testis expressed sequence 10, isoform CRA_a	10	95.6	98.9	98.7	103.8	97.25	101.25	1.041131105	0.295615
P49189	ALDH9A1	4-trimethylaminobutyraldehyde dehydrogenas	14	96.1	98.5	100.1	102.5	97.3	101.3	1.041109969	0.295783
P82909	MRPS36	28S ribosomal protein S36, mitochondrial OS	6	97.4	99.9	100.7	104.7	98.65	102.7	1.041054232	0.296224
Q1HDL3	HBEBP2BPA	HBeAg-binding protein 2 binding protein A O	2	92.5	97.8	99.5	98.6	95.15	99.05	1.040987914	0.296751
Q8IYS2	KIAA2013	Uncharacterized protein KIAA2013 OS=Hom	3	99.9	100.3	102.8	105.6	100.1	104.2	1.040959041	0.296981
Q59EA2		Coronin (Fragment) OS=Homo sapiens PE=2	18	100.4	104.7	108.5	105	102.55	106.75	1.040955631	0.297008
O43390	HNRNPR	Heterogeneous nuclear ribonucleoprotein R O	22	96.8	96.1	101.4	99.4	96.45	100.4	1.040953862	0.297022
P62851	RPS25	40S ribosomal protein S25 OS=Homo sapiens	5	96.6	103.9	103.9	104.8	100.25	104.35	1.040897756	0.297469
P24588	AKAP5	A-kinase anchor protein 5 OS=Homo sapiens	1	100.5	97.8	99.2	107.2	99.15	103.2	1.040847201	0.297871
B4E1F7		cDNA FLJ56305 OS=Homo sapiens PE=2 SV	1	104.4	94.1	98.9	107.7	99.25	103.3	1.040806045	0.298199
A0A024R880	CDK9	Cyclin-dependent kinase 9 (CDC2-related kin	6	101.9	99.2	106.9	102.4	100.55	104.65	1.040775733	0.298441
Q04721	NOTCH2	Neurogenic locus notch homolog protein 2 OS	7	99	102.4	96.7	112.9	100.7	104.8	1.040714995	0.298926
Q07812	BAX	Apoptosis regulator BAX OS=Homo sapiens	3	97.6	98.9	101.4	103.1	98.25	102.25	1.040712468	0.298946
Q9NXK8	FBXL12	F-box/LRR-repeat protein 12 OS=Homo sapie	1	106.5	97.5	104.8	107.5	102	106.15	1.040686275	0.299156
A6NJ78	METTL15	Probable methyltransferase-like protein 15 OS	1	110.9	88.3	108.1	99.2	99.6	103.65	1.040662651	0.299344
Q8N5G0	SMIM20	Small integral membrane protein 20 OS=Hom	2	100.8	96	99.7	105.1	98.4	102.4	1.040650407	0.299443
Q7Z434	MAVS	Mitochondrial antiviral-signaling protein OS=	7	101.4	97.9	105.9	101.5	99.65	103.7	1.040642248	0.299507
O14925	TIMM23	Mitochondrial import inner membrane translo	5	96.3	100.6	104.1	100.8	98.45	102.45	1.040629761	0.299608
Q8NG31	CASC5	Protein CASC5 OS=Homo sapiens GN=CASC	2	100.5	94	99.3	103.1	97.25	101.2	1.040616967	0.29971
A0A024R3R1	ARID4B	AT rich interactive domain 4B (RBP1-like), is	6	99.9	94.9	100.4	102.3	97.4	101.35	1.040554415	0.300211
Q59EC0		Adenosine deaminase, RNA-specific isoform	46	97.7	97.8	103	100.4	97.75	101.7	1.040409207	0.301376
Q4KMQ1	TPRN	Taperin OS=Homo sapiens GN=TPRN PE=1	2	101.6	94	84.2	119.3	97.8	101.75	1.040388548	0.301542
Q9BTT4	MED10	Mediator of RNA polymerase II transcription	2	98.3	92.8	95.2	103.6	95.55	99.4	1.04029304	0.30231
Q7Z7K0	CMC1	COX assembly mitochondrial protein homolo	3	102.2	96.5	103.4	103.3	99.35	103.35	1.040261701	0.302562
A0A024R4K3	MDH2	Malate dehydrogenase OS=Homo sapiens GN	20	94.7	96.8	99.1	100.1	95.75	99.6	1.040208877	0.302988
A0A024QYY4	BCL9	B-cell CLL/lymphoma 9, isoform CRA_a OS	1	103.1	96	104.4	102.7	99.55	103.55	1.040180814	0.303215
G8JLA2	MYL6	Myosin light polypeptide 6 OS=Homo sapiens	1	102	93.3	93	110.1	97.65	101.55	1.039938556	0.305174
B3KTA5		cDNA FLJ37943 fis, clone CTONG2008621,	1	99.1	94.1	102.5	98.4	96.6	100.45	1.039855072	0.305851
O14976	GAK	Cyclin-G-associated kinase OS=Homo sapien	8	100.3	98.7	99.5	107.4	99.5	103.45	1.039698492	0.307124
Q96FH0	BORCS8	BLOC-1-related complex subunit 8 OS=Homo	2	100.1	97.3	102.9	102.3	98.7	102.6	1.039513678	0.308631
Q0VAN5	TBCEL	Tubulin folding cofactor E-like OS=Homo sap	1	99.7	98	95.5	110	98.85	102.75	1.039453718	0.309121
Q71RG4	TMUB2	Transmembrane and ubiquitin-like domain-co	2	104.2	93.8	103.6	102.2	99	102.9	1.039393939	0.309611
A0A024R6H1	SPTLC2	Serine palmitoyltransferase, long chain base s	11	105.1	95.5	104.8	103.7	100.3	104.25	1.039381854	0.30971
Q53H88		Dynactin 2 variant (Fragment) OS=Homo sap	14	98.1	97.5	101.6	101.7	97.8	101.65	1.039366053	0.30984
A0A024R333	TMEM113	Transmembrane protein 113, isoform CRA_a	8	97.4	98.2	101	102.3	97.8	101.65	1.039366053	0.30984
A0A087WVQ	CLTC	Clathrin heavy chain OS=Homo sapiens GN=	82	98.4	99.9	102.2	103.9	99.15	103.05	1.039334342	0.310099
B4DQJ5		cDNA FLJ59211, highly similar to Glucosida	21	100.9	98.2	103.6	103.3	99.55	103.45	1.039176293	0.311396
A8K9K8		rRNA adenine N(6)-methyltransferase OS=H	11	99.3	97.3	104.5	99.8	98.3	102.15	1.039165819	0.311482
Q53RX3	RDH14	Putative uncharacterized protein RDH14 OS=	10	98.8	97.9	105.2	99.2	98.35	102.2	1.039145907	0.311646
Q9H6F5	CCDC86	Coiled-coil domain-containing protein 86 OS=	9	100.4	99.1	108.4	98.9	99.75	103.65	1.039097744	0.312042
A4D1V4	MRPL32	Mitochondrial ribosomal protein L32 OS=Hon	3	98.6	101.5	102.9	105	100.05	103.95	1.03898051	0.313009
Q9HBK9	AS3MT	Arsenite methyltransferase OS=Homo sapiens	3	90.6	99.6	101.1	96.5	95.1	98.8	1.038906414	0.31362
D9HTE9	SLC25A1	Plasma membrane citrate carrier OS=Homo sa	8	95	100.5	100.7	102.4	97.75	101.55	1.03887468	0.313883
Q96CB9	NSUN4	5-methylcytosine rRNA methyltransferase NS	2	99.2	91.6	100.9	97.3	95.4	99.1	1.038784067	0.314633
Q15366	PCBP2	Poly(rC)-binding protein 2 OS=Homo sapiens	7	94.8	103.8	101.4	104.9	99.3	103.15	1.0387714	0.314737
S4R3H4	ACIN1	Apoptotic chromatin condensation inducer in	29	99.7	97.3	101.8	102.8	98.5	102.3	1.03857868	0.316337
A8K799		cDNA FLJ77471, highly similar to Homo sap	1	110.2	84.3	94.6	107.4	97.25	101	1.038560411	0.316488
O15091	KIAA0391	Mitochondrial ribonuclease P protein 3 OS=H	12	98.3	98.8	102.1	102.6	98.55	102.35	1.038559107	0.316499
Q9H0X4	FAM234A	Protein FAM234A OS=Homo sapiens GN=FA	1	101.3	101	106.7	103.4	101.15	105.05	1.038556599	0.31652
O75367	H2AFY	Core histone macro-H2A.1 OS=Homo sapiens	15	96	98.8	101	101.3	97.4	101.15	1.038501027	0.316982
Q6PI98	INO80C	INO80 complex subunit C OS=Homo sapiens	2	100.7	96.7	100.4	104.6	98.7	102.5	1.038500507	0.316986
Q8IUB6	CD95	CD95 antigen (Fragment) OS=Homo sapiens	7	100.7	99.3	105	102.7	100	103.85	1.0385	0.316991
Q9Y4C8	RBM19	Probable RNA-binding protein 19 OS=Homo	11	98.1	96.9	100.8	101.7	97.5	101.25	1.038461538	0.317311
O60832	DKC1	H/ACA ribonucleoprotein complex subunit 4	16	99.6	98.3	101.5	104	98.95	102.75	1.038403234	0.317796
H3BVG0	NUP93	Nuclear pore complex protein Nup93 OS=Hon	30	96.6	99	101.7	101.4	97.8	101.55	1.038343558	0.318294
I3L0A0	TMEM189-UB	HCG2044781 OS=Homo sapiens GN=TMEM	3	97.3	101.1	96	110	99.2	103	1.038306452	0.318604
B4DUQ1		cDNA FLJ54552, highly similar to Heterogen	2	97.8	100.8	102.6	103.6	99.3	103.1	1.038267875	0.318927
D3DU92	RNPS1	RNA binding protein S1 OS=Homo sapiens C	7	98	98.1	101.8	101.8	98.05	101.8	1.038245793	0.319111
A0A024R5X2	hCG_2001986	HCG2001986, isoform CRA_a OS=Homo sap	19	100.9	97.9	105.4	101	99.4	103.2	1.038229376	0.319249
A0A0S2Z5H0	MRPS28	Mitochondrial ribosomal protein S28 isoform	4	99.7	96.5	103.2	100.5	98.1	101.85	1.0382263	0.319275
P16157	ANK1	Ankyrin-1 OS=Homo sapiens GN=ANK1 PE	1	90.4	90.4	89.6	98.1	90.4	93.85	1.038163717	0.319798
Q8NHH9	ATL2	Atlastin-2 OS=Homo sapiens GN=ATL2 PE=	14	100.2	96.4	102	102.1	98.3	102.05	1.038148525	0.319925
P51572	BCAP31	B-cell receptor-associated protein 31 OS=Hor	11	97.9	98.8	102.7	101.5	98.35	102.1	1.038129131	0.320088
B2R5J1		cDNA, FLJ92494, highly similar to Homo sap	1	91.8	102.3	98.9	102.6	97.05	100.75	1.038124678	0.320125
Q9NQW6	ANLN	Anillin OS=Homo sapiens GN=ANLN PE=1	9	106.1	98.5	109.7	102.7	102.3	106.2	1.038123167	0.320138
B2R4R9	RPS28	HCG26477 OS=Homo sapiens GN=RPS28 PI	2	94.9	94.1	97.6	98.6	94.5	98.1	1.038095238	0.320372
A0A140VJC8		Testicular tissue protein Li 2 OS=Homo sapie	14	100.1	97	102.6	102	98.55	102.3	1.03805175	0.320737
Q12800	TFCP2	Alpha-globin transcription factor CP2 OS=Ho	1	109.2	88	102.9	101.8	98.6	102.35	1.038032454	0.320899
A0A024RAJ9	ARSB	Arylsulfatase B, isoform CRA_a OS=Homo s	2	96.3	96	99.4	100.2	96.15	99.8	1.037961518	0.321494
Q13151	HNRNPA0	Heterogeneous nuclear ribonucleoprotein A0	13	98.4	99.2	103.4	101.7	98.8	102.55	1.037955466	0.321545
D3DUK9	C16orf60	Chromosome 16 open reading frame 60, isofo	1	101.7	95.9	98.9	106.2	98.8	102.55	1.037955466	0.321545
Q6ZWT7	MBOAT2	Lysophospholipid acyltransferase 2 OS=Hom	1	100.2	92.3	102	97.8	96.25	99.9	1.037922078	0.321826
Q53HQ0		Flotillin 1 variant (Fragment) OS=Homo sapie	14	98.6	99.6	102.8	102.9	99.1	102.85	1.037840565	0.322511
J3KN67	TPM3	Tropomyosin alpha-3 chain OS=Homo sapien	3	101	100	107.4	101.2	100.5	104.3	1.037810945	0.322762
H7BZJ7	FANCD2	Fanconi anemia group D2 protein (Fragment)	1	107.2	96.6	109.3	102.2	101.9	105.75	1.037782139	0.323004
B2RDQ3		cDNA, FLJ96718, highly similar to Homo sap	5	98.2	97.7	101.5	101.8	97.95	101.65	1.037774375	0.32307
A8K964		cDNA FLJ75071, highly similar to Homo sap	17	98.5	97.5	102.5	100.9	98	101.7	1.037755102	0.323232
Q969X6	UTP4	U3 small nucleolar RNA-associated protein 4	18	98.1	100.7	105.5	100.8	99.4	103.15	1.037726358	0.323474
O94855	SEC24D	Protein transport protein Sec24D OS=Homo s	4	97.8	101.3	97.8	108.8	99.55	103.3	1.037669513	0.323954
A8K946		mRNA cap guanine-N7 methyltransferase OS	7	96.7	97.1	101.1	100	96.9	100.55	1.037667699	0.32397
A0A024RAH8	DDX18	DEAD (Asp-Glu-Ala-Asp) box polypeptide 1	17	98.3	98.4	101.4	102.7	98.35	102.05	1.037620742	0.324367
Q8TC36	SUN5	SUN domain-containing protein 5 OS=Homo	1	91.6	113.9	96.9	116.3	102.75	106.6	1.037469586	0.325647
Q9NXE4	SMPD4	Sphingomyelin phosphodiesterase 4 OS=Hom	15	98.2	96.9	101.9	100.5	97.55	101.2	1.037416709	0.326095
O75694	NUP155	Nuclear pore complex protein Nup155 OS=H	36	98.1	97.3	101.8	100.9	97.7	101.35	1.037359263	0.326583
O15111	CHUK	Inhibitor of nuclear factor kappa-B kinase sub	1	93	102.6	99.1	103.8	97.8	101.45	1.037321063	0.326908
B3KQJ0		cDNA FLJ90530 fis, clone NT2RP4002187, l	7	102.6	95.8	105.3	100.5	99.2	102.9	1.037298387	0.3271
Q9BT22	ALG1	Chitobiosyldiphosphodolichol beta-mannosyl	4	99.7	98.8	103.5	102.4	99.25	102.95	1.037279597	0.32726
P13010	XRCC5	X-ray repair cross-complementing protein 5 C	39	98.2	100.6	103.2	103	99.4	103.1	1.03722334	0.327739
Q00577	PURA	Transcriptional activator protein Pur-alpha OS	6	100.4	101.1	100.8	108.2	100.75	104.5	1.037220844	0.327761
Q16594	TAF9	Transcription initiation factor TFIID subunit 9	4	96.3	105.2	117.7	91.3	100.75	104.5	1.037220844	0.327761
Q9UMN6	KMT2B	Histone-lysine N-methyltransferase 2B OS=H	1	91.5	86	94.8	89.3	88.75	92.05	1.037183099	0.328083
E9PSH0	CELF1	CUGBP Elav-like family member 1 (Fragmer	1	99.6	96.9	108.7	95.1	98.25	101.9	1.037150127	0.328363
Q15363	TMED2	Transmembrane emp24 domain-containing pr	5	97	105.1	113.7	95.9	101.05	104.8	1.037110341	0.328703
Q13427	PPIG	Peptidyl-prolyl cis-trans isomerase G OS=Hor	7	97.5	96.7	101.8	99.6	97.1	100.7	1.03707518	0.329003
Q15397	PUM3	Pumilio homolog 3 OS=Homo sapiens GN=P	23	97.3	97.3	101.7	100.1	97.3	100.9	1.036998972	0.329654
Q13310	PABPC4	Polyadenylate-binding protein 4 OS=Homo sa	17	99.6	100.5	104.3	103.2	100.05	103.75	1.036981509	0.329804
A8K5S3		cDNA FLJ78449 OS=Homo sapiens PE=2 SV	5	97.7	99.7	103	101.7	98.7	102.35	1.03698075	0.32981
Q13501	SQSTM1	Sequestosome-1 OS=Homo sapiens GN=SQS	10	101.8	98.4	104.9	102.7	100.1	103.8	1.036963037	0.329962
Q96HN2	AHCYL2	Adenosylhomocysteinase 3 OS=Homo sapien	2	101.6	96.7	102.7	102.9	99.15	102.8	1.03681291	0.331248
Q9NUQ7	UFSP2	Ufm1-specific protease 2 OS=Homo sapiens	10	98.4	97.5	101.8	101.3	97.95	101.55	1.036753446	0.331758
Q53GD1		Guanine nucleotide-binding protein subunit g	4	97.6	101.1	101.3	104.7	99.35	103	1.036738802	0.331884
O14949	UQCRQ	Cytochrome b-c1 complex subunit 8 OS=Hon	4	93.4	100	100.6	99.9	96.7	100.25	1.036711479	0.332119
P51159	RAB27A	Ras-related protein Rab-27A OS=Homo sapie	3	105.6	96	106.8	102.2	100.8	104.5	1.036706349	0.332163
B4DK09		Tetraspanin OS=Homo sapiens PE=2 SV=1	1	116.1	82.8	97.9	108.3	99.45	103.1	1.03670186	0.332201
Q99961	SH3GL1	Endophilin-A2 OS=Homo sapiens GN=SH3C	4	94.5	99.1	98.3	102.4	96.8	100.35	1.036673554	0.332445
Q15326	ZMYND11	Zinc finger MYND domain-containing protei	4	90	92.8	91.8	97.7	91.4	94.75	1.036652079	0.332629
A0A024R8D2	SLC27A4	Solute carrier family 27 (Fatty acid transporte	13	97.3	99.7	102.7	101.5	98.5	102.1	1.036548223	0.333523
O95169	NDUFB8	NADH dehydrogenase [ubiquinone] 1 beta su	3	102.9	96.9	102.5	104.6	99.9	103.55	1.036536537	0.333624
P62701	RPS4X	40S ribosomal protein S4, X isoform OS=Hor	19	97.7	102.4	103.3	104.1	100.05	103.7	1.036481759	0.334097
A0A024R275	RFK	Riboflavin kinase, isoform CRA_a OS=Homo	1	95.9	98.8	101.3	100.5	97.35	100.9	1.036466359	0.33423
Q9NRJ7	PCDHB16	Protocadherin beta-16 OS=Homo sapiens GN	1	107.2	79.5	82.5	111	93.35	96.75	1.036422067	0.334612
P51151	RAB9A	Ras-related protein Rab-9A OS=Homo sapien	8	101.6	93.7	102.3	100.1	97.65	101.2	1.036354327	0.335198
Q13439	GOLGA4	Golgin subfamily A member 4 OS=Homo sap	24	99.3	99.2	100.8	104.9	99.25	102.85	1.03627204	0.33591
Q30201	HFE	Hereditary hemochromatosis protein OS=Hon	1	98.6	102.9	105.9	102.9	100.75	104.4	1.036228288	0.336289
A0A0G2JPF8	HNRNPCL4	Heterogeneous nuclear ribonucleoprotein C-li	1	99.7	96.3	107.5	95.6	98	101.55	1.03622449	0.336322
A0A0B4J2C3	TPT1	Translationally-controlled tumor protein OS=	4	94.8	95.7	99.5	97.9	95.25	98.7	1.036220472	0.336357

Q8NI37	PPTC7	Protein phosphatase PTC7 homolog OS=Hom	2	99.4	102.2	103	105.9	100.8	104.45	1.036210317	0.336445
Q8N983	MRPL43	39S ribosomal protein L43, mitochondrial OS	6	98.4	100.5	102.6	103.5	99.45	103.05	1.036199095	0.336542
Q16762	TST	Thiosulfate sulfurtransferase OS=Homo sapie	2	98.9	97.3	100.7	102.6	98.1	101.65	1.036187564	0.336642
O60725	ICMT	Protein-S-isoprenylcysteine O-methyltransfera	1	93.1	103.2	96.3	107.1	98.15	101.7	1.036169129	0.336801
Q9Y6R0	NUMBL	Numb-like protein OS=Homo sapiens GN=NU	1	96.5	97.3	96.8	104	96.9	100.4	1.036119711	0.337231
A0A024RA24	FLJ20323	Uncharacterized protein OS=Homo sapiens G	4	99.4	94.5	101.7	99.2	96.95	100.45	1.036101083	0.337392
B4DKQ5		cDNA FLJ54710, highly similar to Target of	5	95.4	95.8	97.9	100.2	95.6	99.05	1.036087866	0.337507
Q9BVC5	C2orf49	Ashwin OS=Homo sapiens GN=C2orf49 PE=	1	92.3	98.9	100.8	97.3	95.6	99.05	1.036087866	0.337507
B4DR52		Histone H2B OS=Homo sapiens PE=2 SV=1	3	87.7	98	92.9	99.5	92.85	96.2	1.036079698	0.337579
Q8IZV5	RDH10	Retinol dehydrogenase 10 OS=Homo sapiens	13	97	97.5	100.4	101.1	97.25	100.75	1.035989717	0.33836
A0A024RAM:	GLRX	Glutaredoxin (Thioltransferase), isoform CRA	2	95.5	91.1	95.9	97.4	93.3	96.65	1.035905681	0.339093
A0A024R0F6	TRIM33	Tripartite motif-containing 33, isoform CRA_	5	98.2	102.8	104.4	103.8	100.5	104.1	1.035820896	0.339833
A0A0J9YVP6	PUF60	Poly(U)-binding-splicing factor PUF60 (Frag	21	99.6	99.1	103.8	102	99.35	102.9	1.03573226	0.340607
P60468	SEC61B	Protein transport protein Sec61 subunit beta C	3	104.3	94.8	110.5	95.7	99.55	103.1	1.035660472	0.341235
Q9UKX7	NUP50	Nuclear pore complex protein Nup50 OS=Ho	17	99.4	96.9	103.3	100	98.15	101.65	1.035659705	0.341242
P49756	RBM25	RNA-binding protein 25 OS=Homo sapiens C	20	98.4	97.9	102.4	100.9	98.15	101.65	1.035659705	0.341242
A0A024R7I4	FAM32A	Family with sequence similarity 32, member A	1	101.4	100.6	96.9	112.3	101	104.6	1.035643564	0.341383
Q9NXE8	CWC25	Pre-mRNA-splicing factor CWC25 homolog	3	98.9	103.2	106.4	102.9	101.05	104.65	1.035625928	0.341537
Q9BUB7	TMEM70	Transmembrane protein 70, mitochondrial OS	4	97.8	99.2	101.4	102.6	98.5	102	1.035532995	0.342352
A0A140VKF3		Testis tissue sperm-binding protein Li 70n OS	4	98.9	87.6	95.1	98	93.25	96.55	1.03538874	0.343619
A0A024R2K1	RAB5A	RAB5A, member RAS oncogene family, isofo	5	102.4	95.7	103.7	101.4	99.05	102.55	1.035335689	0.344086
A8MUM1	TSSC1	Protein TSSC1 OS=Homo sapiens GN=TSSC	2	94	81.7	89	92.9	87.85	90.95	1.035287422	0.344511
H7C146	MAST4	Microtubule-associated serine/threonine-prote	1	88.8	101.1	95.4	101.2	94.95	98.3	1.035281727	0.344561
Q8TAA9	VANGL1	Vang-like protein 1 OS=Homo sapiens GN=V	6	103.9	97.8	104.1	104.7	100.85	104.4	1.035200793	0.345275
B5BU61	HDAC1	Histone deacetylase OS=Homo sapiens GN=I	9	97.7	101.2	101.5	104.4	99.45	102.95	1.035193565	0.345339
A0A024R806	MGC10911	Uncharacterized protein OS=Homo sapiens G	2	104.9	96.9	102.4	106.5	100.9	104.45	1.03518335	0.345429
P57060	RWDD2B	RWD domain-containing protein 2B OS=Hon	1	98.8	94.5	96.1	104	96.65	100.05	1.035178479	0.345472
B3KW34		Protein YIPF OS=Homo sapiens PE=2 SV=1	2	103.5	98.7	105.1	104.2	101.1	104.65	1.035113749	0.346044
Q92545	TMEM131	Transmembrane protein 131 OS=Homo sapie	1	100.1	105.1	107.5	104.9	102.6	106.2	1.035087719	0.346274
A0A024R0Q4	PLD3	Phospholipase D family, member 3, isoform C	11	99.1	97.7	102	101.7	98.4	101.85	1.035060976	0.34651
Q53FH6		Mitogen-activated protein kinase kinase 1 inte	3	105.6	94.6	108.1	99.1	100.1	103.6	1.034965035	0.347359
Q86YT6	MIB1	E3 ubiquitin-protein ligase MIB1 OS=Homo s	3	97.4	94.7	96.5	102.3	96.05	99.4	1.034877668	0.348134
Q5U0I6	RAB1A	H.sapiens ras-related Hrab1A protein OS=Ho	7	99	98.9	102.8	102	98.95	102.4	1.034866094	0.348237
A0A024RBS2	RPLP0	60S acidic ribosomal protein P0 OS=Homo sa	15	95.5	102.4	100.2	104.6	98.95	102.4	1.034866094	0.348237
Q9NX24	NHP2	H/ACA ribonucleoprotein complex subunit 2	4	96	102	98.2	106.7	99	102.45	1.034848485	0.348393
P50613	CDK7	Cyclin-dependent kinase 7 OS=Homo sapiens	4	97.1	98.2	101.4	100.7	97.65	101.05	1.034818228	0.348662
C9JCC6	DRAP1	Dr1-associated corepressor OS=Homo sapien:	3	98	100.4	103.7	101.6	99.2	102.65	1.034778226	0.349017
Q8IXK0	PHC2	Polyhomeotic-like protein 2 OS=Homo sapier	5	102.7	96.8	100.1	106.3	99.75	103.2	1.034586466	0.350725
X6R8A1	CTSA	Carboxypeptidase OS=Homo sapiens GN=CT	7	98.8	98	103.5	100.1	98.4	101.8	1.034552846	0.351025
A0A024R9I5	SLC12A6	Solute carrier family 12 (Potassium/chloride t	2	98.8	104.1	120.4	89.5	101.45	104.95	1.034499754	0.351498
O60271	SPAG9	C-Jun-amino-terminal kinase-interacting prote	8	98.7	101.5	103.3	103.8	100.1	103.55	1.034465534	0.351804
Q9Y5Y5	PEX16	Peroxisomal membrane protein PEX16 OS=H	3	98.7	101.6	104.6	102.6	100.15	103.6	1.034448328	0.351957
A8K3X2	TARBP2	RISC-loading complex subunit TARBP2 OS=	1	96.9	88.9	88.9	103.3	92.9	96.1	1.03444564	0.351982
O95218	ZRANB2	Zinc finger Ran-binding domain-containing p	7	99.9	101	105.1	102.7	100.45	103.9	1.034345445	0.352879
A8K5U9		cDNA FLJ75056, highly similar to Homo sap	10	101.1	97.1	101	104	99.1	102.5	1.034308779	0.353206
P22087	FBL	rRNA 2'-O-methyltransferase fibrillarin OS=I	17	98.1	97.4	101.3	100.9	97.75	101.1	1.0342711	0.353544
B2RAQ8		cDNA, FLJ95058, highly similar to Homo sap	25	99.9	98.7	102.6	102.8	99.3	102.7	1.034239678	0.353826
B3KNI2		cDNA FLJ14650 fis, clone NT2RP2002185, l	5	97.1	101.8	104.2	101.5	99.45	102.85	1.034188034	0.35429
Q6UXI7	VIT	Vitrin OS=Homo sapiens GN=VIT PE=2 SV=	1	114.9	95.8	109.6	108.3	105.35	108.95	1.034171808	0.354435
B2R7I9		cDNA, FLJ93464, highly similar to Homo sap	6	103.5	98.7	107.3	101.8	101.1	104.55	1.034124629	0.354859
Q9BUE6	ISCA1	Iron-sulfur cluster assembly 1 homolog, mito	3	100.3	90.7	99	98.5	95.5	98.75	1.034031414	0.355698
Q86SX6	GLRX5	Glutaredoxin-related protein 5, mitochondrial	3	96.9	97.1	98.6	102	97	100.3	1.034020619	0.355794
A0A024R6W2	NUDT21	Nudix (Nucleoside diphosphate linked moiety	10	97.6	102.4	104.6	102.2	100	103.4	1.034	0.35598
Q8N6M3	FITM2	Fat storage-inducing transmembrane protein 2	1	99.5	97.7	102.6	101.3	98.6	101.95	1.033975659	0.3562
P26373	RPL13	60S ribosomal protein L13 OS=Homo sapiens	9	94.9	105.4	105.2	101.9	100.15	103.55	1.033949076	0.35644
A8KA05	EIF2C3	Protein argonaute-3 OS=Homo sapiens GN=E	1	96.8	94.7	88.5	109.5	95.75	99	1.033942559	0.356498
Q53S33	BOLA3	BolA-like protein 3 OS=Homo sapiens GN=E	5	94.6	95.1	97.2	98.9	94.85	98.05	1.03373748	0.358351
O43684	BUB3	Mitotic checkpoint protein BUB3 OS=Homo	14	98	101.1	98	107.8	99.55	102.9	1.033651431	0.359129
Q96EK4	THAP11	THAP domain-containing protein 11 OS=Hon	1	105.2	96.9	105.5	103.4	101.05	104.45	1.03364671	0.359172
A0A024R7K6	FKSG24	Uncharacterized protein OS=Homo sapiens G	1	99.7	96.6	93.6	109.3	98.15	101.45	1.033622007	0.359396
P42766	RPL35	60S ribosomal protein L35 OS=Homo sapiens	7	96.6	106	103.6	105.8	101.3	104.7	1.033563672	0.359925
D2CFK9	GTPBP4	Nucleolar GTP-binding protein 1 OS=Homo s	28	97.6	99.2	103.6	99.8	98.4	101.7	1.033536585	0.360171
Q92643	PIGK	GPI-anchor transamidase OS=Homo sapiens	10	99.4	100.5	100.7	105.9	99.95	103.3	1.033516758	0.360351
B2RBX8		cDNA, FLJ95758, highly similar to Homo sap	1	111.4	91.5	101.8	107.9	101.45	104.85	1.033514046	0.360376
Q562R1	ACTBL2	Beta-actin-like protein 2 OS=Homo sapiens G	6	94.3	102.7	96.9	106.7	98.5	101.8	1.033502538	0.360479
Q13428	TCOF1	Treacle protein OS=Homo sapiens GN=TCOF	1	99.8	106.3	114.6	98.4	103.05	106.5	1.033478894	0.360695
P18074	ERCC2	TFIIH basal transcription factor complex helic	9	100	97.9	104	100.5	98.95	102.25	1.033350177	0.361865
Q8NI36	WDR36	WD repeat-containing protein 36 OS=Homo s	21	99.3	99.2	104.3	100.8	99.25	102.55	1.03324937	0.362784
Q7Z4H7	HAUS6	HAUS augmin-like complex subunit 6 OS=H	1	95.3	97.4	101.4	97.7	96.35	99.55	1.033212247	0.363122
A8K1Z3		cDNA FLJ75002, highly similar to Homo sap	1	99.5	93.7	96.4	103.2	96.6	99.8	1.033126294	0.363908
Q12906	ILF3	Interleukin enhancer-binding factor 3 OS=Ho	31	97.5	98.9	100.9	102	98.2	101.45	1.033095723	0.364187
E7EQR8	YIPF3	Protein YIPF3 OS=Homo sapiens GN=YIPF3	2	94.8	98.7	97.9	102	96.75	99.95	1.033074935	0.364377
P32246	CCR1	C-C chemokine receptor type 1 OS=Homo sap	1	103.3	102.4	94.3	118.2	102.85	106.25	1.033057851	0.364534
Q16576	RBBP7	Histone-binding protein RBBP7 OS=Homo sa	1	104	102.1	107	105.9	103.05	106.45	1.032993692	0.365121
Q53HA8		Mucolipin 1 variant (Fragment) OS=Homo sa	1	107.2	89.9	115.6	88	98.55	101.8	1.032978184	0.365263
Q10471	GALNT2	Polypeptide N-acetylgalactosaminyltransferas	13	97.4	96.7	100.5	100	97.05	100.25	1.032972694	0.365314
P56524	HDAC4	Histone deacetylase 4 OS=Homo sapiens GN	1	100.6	99.7	110.7	96.2	100.15	103.45	1.032950574	0.365517
Q9NQE9	HINT3	Histidine triad nucleotide-binding protein 3 O	5	103.2	100.3	105.9	104.3	101.75	105.1	1.032923833	0.365762
P13804	ETFA	Electron transfer flavoprotein subunit alpha, n	12	96.8	98	100.7	100.5	97.4	100.6	1.032854209	0.366401
Q8IXI2	RHOT1	Mitochondrial Rho GTPase 1 OS=Homo sapi	5	99	96.4	98.7	103.1	97.7	100.9	1.032753327	0.367327
Q53H29		Nucleoporin 54kDa variant (Fragment) OS=H	15	99.5	96.2	96.5	105.6	97.85	101.05	1.032703117	0.367789
Q59F66		DEAD box polypeptide 17 isoform p82 variar	29	99.4	99.5	103.5	101.9	99.45	102.7	1.032679739	0.368004
V9HW63	HEL-S-97n	Epididymis secretory sperm binding protein L	10	100.1	102.2	106	102.9	101.15	104.45	1.032624815	0.36851
P17096	HMGA1	High mobility group protein HMG-I/HMG-Y	1	93.3	100.1	100.6	99.1	96.7	99.85	1.032574974	0.36897
B4DH07		cDNA FLJ53321, highly similar to Homo sap	29	99	97.5	102.1	100.8	98.25	101.45	1.032569975	0.369016
P10589	NR2F1	COUP transcription factor 1 OS=Homo sapie	1	98.7	98	104.6	98.5	98.35	101.55	1.032536858	0.369322
Q9P0J0	NDUFA13	NADH dehydrogenase [ubiquinone] 1 alpha s	6	95.4	101.4	102	101.2	98.4	101.6	1.032520325	0.369475
Q01804	OTUD4	OTU domain-containing protein 4 OS=Homo	4	101.4	98.5	93.8	112.6	99.95	103.2	1.032516258	0.369512
Q5TDG3	WDR3	WD repeat domain 3, isoform CRA_b OS=Ho	30	99.4	97.5	102	101.3	98.45	101.65	1.032503809	0.369627
Q9UMS0	NFU1	NFU1 iron-sulfur cluster scaffold homolog, m	6	99.4	97.5	100.7	102.6	98.45	101.65	1.032503809	0.369627
Q00688	FKBP3	Peptidyl-prolyl cis-trans isomerase FKBP3 O	8	96	104.2	101	105.7	100.1	103.35	1.032467532	0.369963
Q53GL6	RALY	RNA binding protein (Autoantigenic, hnRNP-	16	98.3	98.9	101.4	102.2	98.6	101.8	1.032454361	0.370084
Q86W42	THOC6	THO complex subunit 6 homolog OS=Homo	12	98.9	98.8	102.4	101.7	98.85	102.05	1.032372281	0.370843
A0A0S2Z3Y1	LGALS3BP	Lectin galactoside-binding soluble 3 binding	7	103.2	97.8	100.2	107.3	100.5	103.75	1.032338308	0.371158
Q9UGU5	HMGXB4	HMG domain-containing protein 4 OS=Homo	2	100.1	101	106.1	101.5	100.55	103.8	1.032322228	0.371306
E7ESC7	MAEA	Macrophage erythroblast attacher OS=Homo	3	96.7	98.6	95.2	106.4	97.65	100.8	1.032258065	0.371901
Q9Y5M8	SRPRB	Signal recognition particle receptor subunit be	10	100.1	95.3	101.1	100.6	97.7	100.85	1.032241556	0.372054
F4ZW62		NF45 OS=Homo sapiens PE=1 SV=1	18	98.7	96.8	101.4	100.4	97.75	100.9	1.032225064	0.372208
Q8NBJ4	GOLM1	Golgi membrane protein 1 OS=Homo sapiens	3	97.6	98.8	103.3	99.4	98.2	101.35	1.032077393	0.373578
B4DUT8	CNN2	Calponin OS=Homo sapiens GN=CNN2 PE=	10	98.2	101.4	104.7	101.3	99.8	103	1.032064128	0.373703
P10809	HSPD1	60 kDa heat shock protein, mitochondrial OS	30	96.4	94.2	96.5	100.2	95.3	98.35	1.032004197	0.374259
A0A024R394	CHORDC1	Cysteine and histidine-rich domain (CHORD)	12	95.8	101.2	100.5	102.8	98.5	101.65	1.031979695	0.374488
P83731	RPL24	60S ribosomal protein L24 OS=Homo sapiens	9	96.6	103.7	103.4	103.3	100.15	103.35	1.031952072	0.374746
A0A024R3T3	TMEM63A	Transmembrane protein 63A, isoform CRA_a	2	96.1	95.9	96.7	101.4	96	99.05	1.031770833	0.376437
Q92889	ERCC4	DNA repair endonuclease XPF OS=Homo sap	2	102.1	96.3	96.9	107.8	99.2	102.35	1.031754032	0.376594
Q8N465	D2HGDH	D-2-hydroxyglutarate dehydrogenase, mitoch	1	89	97	91.7	100.2	93	95.95	1.03172043	0.376908
B2R4D5		Actin-related protein 2/3 complex subunit 3 C	4	90.8	105.1	96.3	105.8	97.95	101.05	1.0316488	0.377579
P52756	RBM5	RNA-binding protein 5 OS=Homo sapiens GN	9	98.6	101.2	105.9	100.2	99.9	103.05	1.031531532	0.378677
A7E261	OPLAH	5-oxoprolinase (ATP-hydrolysing) OS=Homo	2	99.6	94.5	93.8	106.4	97.05	100.1	1.031427099	0.379658
P85037	FOXK1	Forkhead box protein K1 OS=Homo sapiens C	5	96.5	100.9	102	101.6	98.7	101.8	1.031408308	0.379835
Q9H7F0	ATP13A3	Probable cation-transporting ATPase 13A3 O	5	99.9	97.6	101.5	102.2	98.75	101.85	1.031392405	0.379984
Q9NUQ6	SPATS2L	SPATS2-like protein OS=Homo sapiens GN=	2	101.2	93.2	96.3	104.2	97.2	100.25	1.031378601	0.380114
Q8TDZ2	MICAL1	Protein-methionine sulfoxide oxidase MICAL	1	96.1	88.9	90.2	100.6	92.5	95.4	1.031351351	0.380371
A0A024R012	SIRT5	NAD-dependent protein deacylase sirtuin-5, n	1	111.6	86.3	107.9	96.2	98.95	102.05	1.031328954	0.380582
Q9HD42	CHMP1A	Charged multivesicular body protein 1a OS=I	4	98.5	96.6	101	100.2	97.55	100.6	1.031266017	0.381175
B4DIS6		cDNA FLJ56443, highly similar to Putative A	2	99.9	98.7	104	100.8	99.3	102.4	1.03121853	0.381622
Q05513	PRKCZ	Protein kinase C zeta type OS=Homo sapiens	1	94.6	95.1	100	95.6	94.85	97.8	1.03110174	0.382725
P62424	RPL7A	60S ribosomal protein L7a OS=Homo sapiens	18	96.7	103	103.5	102.4	99.85	102.95	1.03104657	0.383246
A0A024RDG6	SCARB2	Scavenger receptor class B, member 2, isofor	6	103.1	96.7	102.5	103.5	99.9	103	1.031031031	0.383393
Q5F1R6	DNAJC21	DnaJ homolog subfamily C member 21 OS=F	2	94.8	101.8	99.6	103.1	98.3	101.35	1.031027467	0.383427
I3L4C2	BAIAP2	Brain-specific angiogenesis inhibitor 1-associ	3	99.2	97.4	99.1	103.6	98.3	101.35	1.031027467	0.383427
Q9NWB6	ARGLU1	Arginine and glutamate-rich protein 1 OS=Ho	3	98	98.9	102.4	100.6	98.45	101.5	1.030980193	0.383874

P17480	UBTF	Nucleolar transcription factor 1 OS=Homo sap	25	98.7	98.9	101.6	102.1	98.8	101.85	1.030870445	0.384914
A0A0U5PXQ	HLA-C	MHC class I antigen OS=Homo sapiens GN=	3	94.7	106.2	100.7	106.4	100.45	103.55	1.030861125	0.385002
P36957	DLST	Dihydrolipoyllysine-residue succinyltransfera	13	99.6	98.2	101.3	102.6	98.9	101.95	1.030839232	0.38521
H0Y368	DPM1	Dolichol-phosphate mannosyltransferase subu	9	99	99	102.3	101.8	99	102.05	1.030808081	0.385506
Q8TC12	RDH11	Retinol dehydrogenase 11 OS=Homo sapiens	10	98.5	99.8	104.7	99.7	99.15	102.2	1.030761473	0.385948
E9PHG5	CYP20A1	Cytochrome P450 20A1 OS=Homo sapiens G	6	99.9	98.5	102.1	102.4	99.2	102.25	1.030745968	0.386096
P02751	FN1	Fibronectin OS=Homo sapiens GN=FN1 PE=	13	99.2	115.5	114.1	107.2	107.35	110.65	1.030740568	0.386147
A0A096WXL	ATP8	ATP synthase protein 8 OS=Homo sapiens G	3	99.1	96.2	105.2	96.1	97.65	100.65	1.030721966	0.386323
V9HWD3	HEL117	Epididymis luminal protein 117 OS=Homo sa	8	100.6	98	103.5	101.2	99.3	102.35	1.030715005	0.38639
A8K7A0		DNA primase large subunit OS=Homo sapien	2	96.6	99	93.6	108	97.8	100.8	1.030674847	0.386772
Q96EV2	RBM33	RNA-binding protein 33 OS=Homo sapiens C	4	96	103	104.5	100.6	99.5	102.55	1.030653266	0.386978
F8W930	IGF2BP2	Insulin-like growth factor 2 mRNA-binding p	11	98.8	103.6	105.2	103.4	101.2	104.3	1.030632411	0.387176
P83876	TXNL4A	Thioredoxin-like protein 4A OS=Homo sapier	6	100.4	95.5	101.9	100	97.95	100.95	1.030627871	0.387219
A8K3Q3		cDNA FLJ78230 OS=Homo sapiens PE=2 SV	3	100.8	98.4	108.6	96.7	99.6	102.65	1.03062249	0.38727
Q96A26	FAM162A	Protein FAM162A OS=Homo sapiens GN=FA	6	96.7	99.5	101.9	100.3	98.1	101.1	1.03058104	0.387665
Q6L8Q7	PDE12	2',5'-phosphodiesterase 12 OS=Homo sapiens	14	100.5	96.3	102.9	99.9	98.4	101.4	1.030487805	0.388554
Q9H2D1	SLC25A32	Mitochondrial folate transporter/carrier OS=H	3	105	98.4	103.7	105.9	101.7	104.8	1.030481809	0.388611
Q9GZR2	REXO4	RNA exonuclease 4 OS=Homo sapiens GN=R	7	99.8	97.4	103.3	99.9	98.6	101.6	1.030425963	0.389145
Q562M3	ACT	Actin-like protein (Fragment) OS=Homo sapi	1	96.4	97.8	99.1	101	97.1	100.05	1.03038105	0.389573
E7EQL5	DYNC1I2	Cytoplasmic dynein 1 intermediate chain 2 (F	1	89.9	104.6	96.8	103.6	97.25	100.2	1.03033419	0.390021
Q96MF7	NSMCE2	E3 SUMO-protein ligase NSE2 OS=Homo sa	3	99.4	102.2	103	104.7	100.8	103.85	1.030257937	0.390751
Q6IPI1	RPL29	Ribosomal protein L29 OS=Homo sapiens GN	2	92.3	106.4	103.6	101.1	99.35	102.35	1.030196276	0.391342
Q8WVX3	C4orf3	Uncharacterized protein C4orf3 OS=Homo sa	1	99.9	95.9	101.5	100.2	97.9	100.85	1.030132789	0.391951
A8K7D9		Importin subunit alpha OS=Homo sapiens PE	17	98.2	98	100.9	101.2	98.1	101.05	1.030071356	0.392541
Q8TCS8	PNPT1	Polyribonucleotide nucleotidyltransferase 1, n	23	101.6	98	101.8	103.8	99.8	102.8	1.03006012	0.392648
Q9NRG0	CHRAC1	Chromatin accessibility complex protein 1 OS	2	95.3	104.5	100.7	105.1	99.9	102.9	1.03003003	0.392938
A8K878	MANF	Mesencephalic astrocyte-derived neurotrophic	11	97.7	98.8	99.2	103.2	98.25	101.2	1.030025445	0.392982
Q06481	APLP2	Amyloid-like protein 2 OS=Homo sapiens GN	2	103.4	96.5	100.5	105.4	99.95	102.95	1.030015008	0.393082
B3KM57		cDNA FLJ10347 fis, clone NT2RM2001035,	4	102.2	97.7	103.5	102.4	99.95	102.95	1.030015008	0.393082
Q8NBW4	SLC38A9	Sodium-coupled neutral amino acid transporte	1	101.6	91.7	89.8	109.3	96.65	99.55	1.030005173	0.393176
Q86SQ4	ADGRG6	Adhesion G-protein coupled receptor G6 OS=	5	105.4	98.2	104	105.7	101.8	104.85	1.029960707	0.393604
Q9Y3D9	MRPS23	28S ribosomal protein S23, mitochondrial OS	6	98.2	95.5	100.7	98.8	96.85	99.75	1.029943211	0.393773
Q53GT1	KLHL22	Kelch-like protein 22 OS=Homo sapiens GN=	1	101.9	98.6	99.7	106.8	100.25	103.25	1.029925187	0.393946
Q9Y6N1	COX11	Cytochrome c oxidase assembly protein COX	1	100.2	100.4	99.9	106.7	100.3	103.3	1.029910269	0.394089
A0A087WT44	HMOX2	Heme oxygenase 2 OS=Homo sapiens GN=H	13	100.1	97.2	102.4	100.8	98.65	101.6	1.0299037	0.394154
Q5U0D2	TAGLN	Transgelin OS=Homo sapiens GN=TAGLN F	15	98.5	102.2	104.2	102.5	100.35	103.35	1.029895366	0.394233
B3KNS8		cDNA FLJ30322 fis, clone BRACE2006703,	9	96.3	98.2	100.1	100.2	97.25	100.15	1.029820051	0.394959
Q59EL4		PRPF4 protein variant (Fragment) OS=Homo	21	98.1	100	100.2	103.8	99.05	102	1.029782938	0.395318
P67812	SEC11A	Signal peptidase complex catalytic subunit SE	6	102	99.6	106	101.6	100.8	103.8	1.029761905	0.39552
A0A024R136	RACGAP1	Rac GTPase activating protein 1, isoform CR	15	100.6	97.9	100.7	103.7	99.25	102.2	1.029722922	0.395897
Q9UK22	FBXO2	F-box only protein 2 OS=Homo sapiens GN=	8	93.9	98.3	100.1	97.8	96.1	98.95	1.029656608	0.396537
A8K588		cDNA FLJ76823, highly similar to Homo sap	7	100.7	98.3	100.7	104.2	99.5	102.45	1.029648241	0.396619
Q9GZU7	CTDSP1	Carboxy-terminal domain RNA polymerase II	1	96	96.4	99.3	98.8	96.2	99.05	1.02962578	0.396836
E5KS22		Mitochondrial DNA polymerase subunit gamı	1	99	100.2	105.7	99.4	99.6	102.55	1.029618474	0.396906
Q53GQ2		COP9 constitutive photomorphogenic homolo	1	101.7	97.7	97.3	108	99.7	102.65	1.029588766	0.397193
J3QLS3	MRPS7	28S ribosomal protein S7, mitochondrial OS=	10	97.5	98.6	100.9	101	98.05	100.95	1.029576747	0.39731
P13995	MTHFD2	Bifunctional methylenetetrahydrofolate dehyd	9	101.4	102.5	105.8	104.1	101.95	104.95	1.029426189	0.398769
P06730	EIF4E	Eukaryotic translation initiation factor 4E OS	6	97.3	103.9	103	104.1	100.6	103.55	1.029324056	0.399761
Q9H501	ESF1	ESF1 homolog OS=Homo sapiens GN=ESF1	16	99.7	98.1	102.5	101.1	98.9	101.8	1.029322548	0.399776
A0A0S2Z491	NPM1	Nucleophosmin isoform 2 (Fragment) OS=Ho	7	96.2	95.3	97.3	99.8	95.75	98.55	1.02924282	0.40055
Q8TCT9	HM13	Minor histocompatibility antigen H13 OS=Ho	6	103.4	91.8	104	96.9	97.6	100.45	1.02920082	0.40096
E7EW20	MYO6	Unconventional myosin-VI OS=Homo sapien	12	101.5	95.4	102.5	100.1	98.45	101.3	1.028948705	0.40342
A0A024RD36	RPL7L1	Ribosomal protein L7-like 1, isoform CRA_b	8	101.2	99.3	105.5	100.8	100.25	103.15	1.028927681	0.403625
Q9BQP7	MGME1	Mitochondrial genome maintenance exonucle	7	99.5	97.9	101.6	101.5	98.7	101.55	1.02887538	0.404137
Q13084	MRPL28	39S ribosomal protein L28, mitochondrial OS	7	96.4	101.2	102.5	100.8	98.8	101.65	1.028846154	0.404423
P14927	UQCRB	Cytochrome b-c1 complex subunit 7 OS=Hon	1	103.7	97.9	105.9	101.5	100.8	103.7	1.028769841	0.40517
Q13445	TMED1	Transmembrane emp24 domain-containing pr	3	97.4	97.4	101.1	99.3	97.4	100.2	1.028747433	0.40539
Q6ICQ8	ARHG	ARHG protein (Fragment) OS=Homo sapiens	8	99.9	99.8	103.3	102.1	99.85	102.7	1.028542814	0.4074
P14866	HNRNPL	Heterogeneous nuclear ribonucleoprotein L O	25	96.9	100	101.7	100.8	98.45	101.25	1.028440833	0.408403
Q9UGU0	TCF20	Transcription factor 20 OS=Homo sapiens GN	4	98.5	94.9	92.3	106.6	96.7	99.45	1.028438469	0.408427
Q96K17	BTF3L4	Transcription factor BTF3 homolog 4 OS=Ho	2	94.1	103.2	100.6	102.3	98.65	101.45	1.028383173	0.408972
A0A0G2JIW1	HSPA1B	Heat shock 70 kDa protein 1B OS=Homo sap	12	96.5	101	101.9	101.2	98.75	101.55	1.02835443	0.409255
A0A024R9J7	DCC1	Defective in sister chromatid cohesion homolo	2	97.3	100.4	103.5	99.8	98.85	101.65	1.028325746	0.409538
Q6ZNF7		cDNA FLJ16146 fis, clone BRAMY2040159,	1	103	95	102	101.6	99	101.8	1.028282828	0.409961
B2RDF5		cDNA, FLJ96587, highly similar to Homo sap	8	100.1	98.6	102.3	102	99.35	102.15	1.028183191	0.410946
A0A024RDT6	C13orf1	Chromosome 13 open reading frame 1, isofor	3	95.3	103.5	102.9	101.5	99.4	102.2	1.028169014	0.411087
Q5TEC6	HIST2H3PS2	Histone H3 OS=Homo sapiens GN=HIST2H3	2	88.3	90.5	89.7	94.1	89.4	91.9	1.027964206	0.413116
A0A0S2Z4V6	WFS1	Wolfram syndrome 1 isoform 1 (Fragment) O	8	99.7	97.1	101.6	100.7	98.4	101.15	1.027947154	0.413285
Q8TED0	UTP15	U3 small nucleolar RNA-associated protein 1:	16	98.3	98.6	103.2	99.2	98.45	101.2	1.027932961	0.413426
A0A024QYR3		Transmembrane 9 superfamily member OS=H	8	99.9	97	101.2	101.2	98.45	101.2	1.027932961	0.413426
Q9UII2	ATPIF1	ATPase inhibitor, mitochondrial OS=Homo sa	5	97.5	99.4	101.6	100.8	98.45	101.2	1.027932961	0.413426
Q96JJ7	TMX3	Protein disulfide-isomerase TMX3 OS=Homo	6	102.2	101.9	100.6	109.2	102.05	104.9	1.027927487	0.41348
Q9NR48	ASH1L	Histone-lysine N-methyltransferase ASH1L C	1	103.5	97.2	102.1	104.2	100.35	103.15	1.027902342	0.41373
Q9BVJ6	UTP14A	U3 small nucleolar RNA-associated protein 1·	24	97.8	99.4	101.4	101.3	98.6	101.35	1.027890467	0.413849
Q9NX47	MARCH5	E3 ubiquitin-protein ligase MARCH5 OS=Ho	5	101.6	96	99.8	103.3	98.8	101.55	1.027834008	0.414409
P16333	NCK1	Cytoplasmic protein NCK1 OS=Homo sapien	4	98.9	102.4	106.9	100	100.65	103.45	1.027819175	0.414557
Q7Z2Z2	EFL1	Elongation factor-like GTPase 1 OS=Homo sa	3	105.1	96.4	102.5	104.6	100.75	103.55	1.027791563	0.414831
O75152	ZC3H11A	Zinc finger CCCH domain-containing protein	17	99.8	98.2	102.5	101	99	101.75	1.027777778	0.414969
A0A024R0C0	CEPT1	Choline/ethanolamine phosphotransferase 1, i	2	95.9	98.7	99.8	100.2	97.3	100	1.027749229	0.415254
P62280	RPS11	40S ribosomal protein S11 OS=Homo sapiens	12	95.5	103	101.9	102.1	99.25	102	1.027707809	0.415666
P35914	HMGCL	Hydroxymethylglutaryl-CoA lyase, mitochon	5	99.4	95.6	99.9	100.5	97.5	100.2	1.027692308	0.41582
Q8TCT7	SPPL2B	Signal peptide peptidase-like 2B OS=Homo s	1	104	94.8	102.8	101.5	99.4	102.15	1.027665996	0.416083
O75396	SEC22B	Vesicle-trafficking protein SEC22b OS=Hom	9	99.4	99.5	101.3	103.1	99.45	102.2	1.027652086	0.41622
Q8IWJ2	GCC2	GRIP and coiled-coil domain-containing prote	1	106.4	99.9	104.9	107.1	103.15	106	1.027629666	0.416445
A0A024R6L5	TDP1	Tyrosyl-DNA phosphodiesterase 1, isoform C	1	100.9	98.6	98.9	106.1	99.75	102.5	1.027568922	0.417051
Q6FGH9	DNCL1	DNCL1 protein OS=Homo sapiens GN=DNC	2	96.1	96.6	98.1	99.9	96.35	99	1.027503892	0.417701
Q9NV31	IMP3	U3 small nucleolar ribonucleoprotein protein	7	100.4	96.7	101.5	101	98.55	101.25	1.02739726	0.418767
A0A0S2Z404	RCC1	Regulator of chromosome condensation 1 isof	15	98.7	98.4	101.8	100.7	98.55	101.25	1.02739726	0.418767
O15504	NUPL2	Nucleoporin-like protein 2 OS=Homo sapiens	5	99.1	102.2	104.6	102.2	100.65	103.4	1.027322404	0.419516
Q00839	HNRNPU	Heterogeneous nuclear ribonucleoprotein U C	32	96.5	98	99.6	100.2	97.25	99.9	1.027249357	0.420249
Q9H6Y7	RNF167	E3 ubiquitin-protein ligase RNF167 OS=Hom	2	100.1	98.4	102.1	101.8	99.25	101.95	1.02720403	0.420704
Q8N1S5	SLC39A11	Zinc transporter ZIP11 OS=Homo sapiens GN	1	103	99.6	103.3	104.8	101.3	104.05	1.027147088	0.421276
A0A024RBN1	PRKAB1	Protein kinase, AMP-activated, beta 1 non-cat	1	100.3	95.3	99.5	101.4	97.8	100.45	1.027096115	0.421787
B4DRE5		cDNA FLJ55391 OS=Homo sapiens PE=2 SV	4	97.2	103.2	100.5	105.3	100.2	102.9	1.026946108	0.423298
Q9BQG0	MYBBP1A	Myb-binding protein 1A OS=Homo sapiens C	40	97.9	99.1	102.2	100.1	98.5	101.15	1.026903553	0.423727
A0A0K0K1J1	HEL-S-2	Cystatin OS=Homo sapiens GN=HEL-S-2 PE	3	101.2	95.9	99.2	103.2	98.55	101.2	1.026889904	0.423864
A0A024QZK8	HNRPH3	Heterogeneous nuclear ribonucleoprotein H3 (	12	97.7	99.5	100.6	101.9	98.6	101.25	1.026876268	0.424003
Q8IUR0	TRAPPC5	Trafficking protein particle complex subunit 5	2	98.8	98.4	100.8	101.7	98.6	101.25	1.026876268	0.424003
Q9UKG1	APPL1	DCC-interacting protein 13-alpha OS=Homo	4	95.4	102.2	99	103.9	98.8	101.45	1.026821862	0.424551
A8K8P8		Alpha-(1,6)-fucosyltransferase OS=Homo sap	4	92.9	106	102.1	102.1	99.45	102.1	1.026646556	0.426323
Q06830	PRDX1	Peroxiredoxin-1 OS=Homo sapiens GN=PRD	11	92.8	99.5	97.8	99.6	96.15	98.7	1.026521061	0.427594
E9KL30		Proteasome subunit beta type OS=Homo sapie	7	97	99.3	101.5	100	98.15	100.75	1.026490066	0.427909
A0A024R9K7	C15orf24	Chromosome 15 open reading frame 24, isofo	6	101.2	98.9	104	101.4	100.05	102.7	1.026486757	0.427943
O60264	SMARCA5	SWI/SNF-related matrix-associated actin-dep	28	98.5	97.9	100.3	101.3	98.2	100.8	1.026476578	0.428045
Q59ED7		Putative uncharacterized protein (Fragment) C	25	98.3	98.4	98.3	103.6	98.35	100.95	1.026436197	0.428456
O15439	ABCC4	Multidrug resistance-associated protein 4 OS=	2	98.3	102.3	104.2	101.7	100.3	102.95	1.026420738	0.428613
Q9H3Z4	DNAJC5	DnaJ homolog subfamily C member 5 OS=Ho	3	98.2	88.4	96.5	95	93.3	95.75	1.026259378	0.430253
O00629	KPNA4	Importin subunit alpha-3 OS=Homo sapiens C	7	101.8	93.6	100.3	100.2	97.7	100.25	1.026100307	0.431875
Q9Y320	TMX2	Thioredoxin-related transmembrane protein 2	8	98	97.6	99.2	101.5	97.8	100.35	1.02607362	0.432148
P22626	HNRNPA2B1	Heterogeneous nuclear ribonucleoproteins A2	21	95.1	96.7	97.6	99.2	95.9	98.4	1.026068822	0.432196
Q2L6J2	ZNRD1	DNA-directed RNA polymerase subunit OS=I	1	100.5	91.3	96.4	100.4	95.9	98.4	1.026068822	0.432196
Q96Q89	KIF20B	Kinesin-like protein KIF20B OS=Homo sapie	6	98.1	97.6	98.7	102.1	97.85	100.4	1.026060296	0.432283
Q2L6I0	PPP1R10	FB19 protein OS=Homo sapiens GN=PPP1R1	6	98	101.6	101.3	103.5	99.8	102.4	1.026052104	0.432367
O15460	P4HA2	Prolyl 4-hydroxylase subunit alpha-2 OS=Hor	14	101	98.8	104.6	100.4	99.9	102.5	1.026026026	0.432633
A0A0J9YWM	ALDH7A1	Alpha-aminoadipic semialdehyde dehydrogen	1	90.7	105.3	99	102.1	98	100.55	1.026020408	0.432691
A0A0S2Z5U6	PYCR2	Pyrroline-5-carboxylate reductase (Fragment)	11	99	101	103.3	101.9	100	102.6	1.026	0.432899
O95415	BRI3	Brain protein I3 OS=Homo sapiens GN=BRI3	1	101.5	110.3	108.3	109	105.9	108.65	1.025967894	0.433228
A1L3Z9	QRICH1	Glutamine-rich 1 OS=Homo sapiens GN=QR	2	97.8	106.5	105	104.6	102.15	104.8	1.025942242	0.43349
Q6IFL7		Olfactory receptor OR17-2 OS=Homo sapiens	1	107.4	97.2	110.3	99.6	102.3	104.95	1.025904203	0.433879
Q9H3P7	ACBD3	Golgi resident protein GCP60 OS=Homo sapi	8	100.4	100.6	103.4	102.8	100.5	103.1	1.025870647	0.434222
A0A096LPI6		Uncharacterized protein (Fragment) OS=Hom	8	98.4	98.9	101.9	100.5	98.65	101.2	1.025848961	0.434444
H0Y8C3	MTCH1	Mitochondrial carrier homolog 1 (Fragment) C	7	100.6	101	105.4	101.4	100.8	103.4	1.025793651	0.435011

O95067	CCNB2	G2/mitotic-specific cyclin-B2 OS=Homo sapi	1	110.1	91.6	95.3	111.6	100.85	103.45	1.025780863	0.435141
Q6IAX2	RPL21	RPL21 protein OS=Homo sapiens GN=RPL2	8	98	103.7	102.6	104.3	100.85	103.45	1.025780863	0.435141
Q9HCD5	NCOA5	Nuclear receptor coactivator 5 OS=Homo sap	10	96.1	97.9	98.4	100.6	97	99.5	1.025773196	0.435221
M0R2A0	EMC10	ER membrane protein complex subunit 10 OS	4	97.2	97.2	99	100.4	97.2	99.7	1.025720165	0.435765
Q9UJU1	VIL2	Cytovillin 2 (Fragment) OS=Homo sapiens G	1	93.7	108.8	106.3	101.4	101.25	103.85	1.025679012	0.436187
Q14019	COTL1	Coactosin-like protein OS=Homo sapiens GN	6	99.9	102.8	103.3	104.6	101.35	103.95	1.025653675	0.436447
Q9BYN8	MRPS26	28S ribosomal protein S26, mitochondrial OS	9	98.3	96.8	100.7	99.4	97.55	100.05	1.025627883	0.436712
M0QXF9	BCAT2	Branched-chain-amino-acid aminotransferase	8	102.8	97	98.6	106.3	99.9	102.45	1.025525526	0.437764
A0A087WZN	IDH3B	Isocitrate dehydrogenase [NAD] subunit, mitc	15	100	100.3	104.4	101	100.15	102.7	1.025461807	0.438421
A0A024R8A7	hCG_31253	HCG31253, isoform CRA_a OS=Homo sapie	19	98.7	97.9	100.4	101.2	98.3	100.8	1.02543235	0.438724
B4DPG9		cDNA FLJ59630, highly similar to Growth ho	3	98.5	98.6	99.8	102.3	98.55	101.05	1.025367834	0.439389
A0A087WUT	EIF5B	Eukaryotic translation initiation factor 5B OS	25	100.1	101.1	104.2	102.1	100.6	103.15	1.025347913	0.439594
O14965	AURKA	Aurora kinase A OS=Homo sapiens GN=AUI	5	100	101.5	102.5	104.1	100.75	103.3	1.025310174	0.439984
P08754	GNAI3	Guanine nucleotide-binding protein G(k) subu	5	101.1	100.6	109.1	97.7	100.85	103.4	1.025285077	0.440243
Q5BKT4	ALG10	Dol-P-Glc:Glc(2)Man(9)GlcNAc(2)-PP-Dol a	2	104.7	89.1	100.5	98.2	96.9	99.35	1.025283798	0.440256
P61019	RAB2A	Ras-related protein Rab-2A OS=Homo sapien	8	101.9	99.9	102.6	104.3	100.9	103.45	1.025272547	0.440373
Q9BRU9	UTP23	rRNA-processing protein UTP23 homolog OS	2	92.6	97.6	98.4	96.6	95.1	97.5	1.025236593	0.440744
Q5ZPR3	CD276	CD276 antigen OS=Homo sapiens GN=CD27	6	102.9	99.3	102.5	104.8	101.1	103.65	1.025222552	0.440889
A0A024R1S8	LASP1	LIM and SH3 protein 1, isoform CRA_b OS=	8	97.2	101.4	101.9	101.7	99.3	101.8	1.025176234	0.441367
P17706	PTPN2	Tyrosine-protein phosphatase non-receptor ty	7	98.6	96.3	99.7	100.1	97.45	99.9	1.025141098	0.441731
Q8WWC4	C2orf47	m-AAA protease-interacting protein 1, mitocl	6	100.5	98.6	102.2	101.9	99.55	102.05	1.025113009	0.442022
A8K865		cDNA FLJ75789, highly similar to Homo sap	3	102	97.2	99.9	104.3	99.6	102.1	1.025100402	0.442153
A0A024R1V6	PCGF2	Polycomb group ring finger 2, isoform CRA_	1	106	89.3	87.7	112.5	97.65	100.1	1.025089606	0.442264
O15121	DEGS1	Sphingolipid delta(4)-desaturase DES1 OS=H	3	100	103.3	99.5	108.9	101.65	104.2	1.02508608	0.442301
Q05D60	DEUP1	Deuterosome assembly protein 1 OS=Homo s	1	105.2	106.1	102.2	114.4	105.65	108.3	1.025082821	0.442334
Q6FH20	NTS	NTS protein OS=Homo sapiens GN=NTS PE	2	98.1	105.5	102.1	106.6	101.8	104.35	1.025049116	0.442684
V9HWI3	HEL-S-130P	Cathepsin D (Lysosomal aspartyl peptidase), i	14	100	99.7	102.6	102.1	99.85	102.35	1.025037556	0.442804
O60645	EXOC3	Exocyst complex component 3 OS=Homo sap	2	101.5	98.5	101.9	103.1	100	102.5	1.025	0.443193
H7C2Q8	EBNA1BP2	EBNA1 binding protein 2, isoform CRA_d O	14	94.8	98.2	98.6	99.2	96.5	98.9	1.024870466	0.444536
A0A024R7I8	IL27RA	Interleukin 27 receptor, alpha, isoform CRA_	1	112.5	93.1	101.3	109.4	102.8	105.35	1.024805447	0.445213
Q9Y289	SLC5A6	Sodium-dependent multivitamin transporter C	2	99.6	98.2	103.9	98.8	98.9	101.35	1.024772497	0.445555
P11177	PDHB	Pyruvate dehydrogenase E1 component subur	14	99.4	98.4	101.9	100.8	98.9	101.35	1.024772497	0.445555
Q9NZW5	MPP6	MAGUK p55 subfamily member 6 OS=Homo	9	99.1	99.2	98.2	105	99.15	101.6	1.024710035	0.446205
P53582	METAP1	Methionine aminopeptidase 1 OS=Homo sapi	13	100.9	101.6	105.8	101.7	101.25	103.75	1.024691358	0.4464
Q96QE5	TEFM	Transcription elongation factor, mitochondria	6	99.2	99.4	103.3	100.2	99.3	101.75	1.024672709	0.446594
B2R6X5		cDNA, FLJ93166, highly similar to Homo sap	1	104.1	94.7	95.9	107.8	99.4	101.85	1.024647887	0.446853
Q9NR12	PDLIM7	PDZ and LIM domain protein 7 OS=Homo sa	14	104.5	102.6	107.2	105	103.55	106.1	1.024625785	0.447083
P52907	CAPZA1	F-actin-capping protein subunit alpha-1 OS=I	9	100.5	98.8	102.8	101.4	99.65	102.1	1.024586051	0.447497
Q8N4L2	TMEM55A	Type 2 phosphatidylinositol 4,5-bisphosphate	3	96.9	98.4	94.2	105.9	97.65	100.05	1.024577573	0.447585
Q99442	SEC62	Translocation protein SEC62 OS=Homo sapie	6	100.8	99	101.7	103	99.9	102.35	1.024524525	0.448139
B0QYD3	APOBEC3B	DNA dC->dU-editing enzyme APOBEC-3B (	12	100.6	95.9	101.4	99.9	98.25	100.65	1.024427481	0.449152
Q8NI27	THOC2	THO complex subunit 2 OS=Homo sapiens G	21	100.9	95.9	100.1	101.5	98.4	100.8	1.024390244	0.449543
Q9NQ92	COPRS	Coordinator of PRMT5 and differentiation sti	3	107.3	98.5	108.3	102.5	102.9	105.4	1.024295432	0.450534
Q8IZ83	ALDH16A1	Aldehyde dehydrogenase family 16 member A	3	100.7	101.1	105	101.7	100.9	103.35	1.024281467	0.450681
B7Z2B5		cDNA FLJ54713, highly similar to Gephyrin	3	98.1	99.8	100.9	101.8	98.95	101.35	1.024254674	0.450962
O60488	ACSL4	Long-chain-fatty-acid--CoA ligase 4 OS=Hor	19	103.4	102.8	105.3	105.9	103.1	105.6	1.024248303	0.451028
G5E998	CREM	cAMP-responsive element modulator OS=Ho	1	103.3	98.9	96.2	110.9	101.1	103.55	1.024233432	0.451184
Q9NPF0	CD320	CD320 antigen OS=Homo sapiens GN=CD32	4	101.3	101.1	103.3	104	101.2	103.65	1.024209486	0.451435
Q15042	RAB3GAP1	Rab3 GTPase-activating protein catalytic subu	6	101.8	97	101.8	101.8	99.4	101.8	1.024144869	0.452113
Q8IWE4	DCUN1D3	DCN1-like protein 3 OS=Homo sapiens GN=	1	114.3	97.2	114.3	102.3	105.75	108.3	1.024113475	0.452443
Q6SPF0	SAMD1	Atherin OS=Homo sapiens GN=SAMD1 PE=	3	92.9	98	95.8	99.7	95.45	97.75	1.024096386	0.452622
A8K018		cDNA FLJ75944 OS=Homo sapiens PE=2 SV	1	96.8	102.4	99.9	104.1	99.6	102	1.024096386	0.452622
O95298	NDUFC2	NADH dehydrogenase [ubiquinone] 1 subunit	3	98.6	92.4	97.3	98.3	95.5	97.8	1.02408377	0.452755
Q96G21	IMP4	U3 small nucleolar ribonucleoprotein protein	8	98.4	101.1	104.3	100	99.75	102.15	1.02406015	0.453003
Q543A1	TMEM9B	Putative NFkB activating protein OS=Homo s	1	109.9	94.1	103.9	105	102	104.45	1.024019608	0.453429
Q99816	TSG101	Tumor susceptibility gene 101 protein OS=Ho	5	99.3	96.4	98.6	101.8	97.85	100.2	1.024016352	0.453463
Q9UHG3	PCYOX1	Prenylcysteine oxidase 1 OS=Homo sapiens (	13	98.3	97.6	102.2	98.4	97.95	100.3	1.023991833	0.453721
X5D945	HRAS	Harvey rat sarcoma viral oncoprotein isoform	2	97.3	98.9	103.3	97.6	98.1	100.45	1.023955148	0.454107
Q96IK0	TMEM101	Transmembrane protein 101 OS=Homo sapier	1	95.3	105.1	99.4	105.8	100.2	102.6	1.023952096	0.454139
P62318	SNRPD3	Small nuclear ribonucleoprotein Sm D3 OS=I	4	93.5	94.9	94.5	98.4	94.2	96.45	1.02388535	0.454842
P52815	MRPL12	39S ribosomal protein L12, mitochondrial OS	6	97	99.8	101.3	100.2	98.4	100.75	1.023882114	0.454875
Q05639	EEF1A2	Elongation factor 1-alpha 2 OS=Homo sapien	9	93	103.8	101.2	100.3	98.4	100.75	1.023882114	0.454875
Q9H0A0	NAT10	RNA cytidine acetyltransferase OS=Homo sa	20	99	97.9	101.6	100	98.45	100.8	1.023869985	0.455004
Q9BV79	MECR	Trans-2-enoyl-CoA reductase, mitochondrial (	3	97.3	95.5	97	100.4	96.4	98.7	1.023858921	0.45512
P42285	SKIV2L2	Superkiller viralicidic activity 2-like 2 OS=Ho	34	99.5	97.9	102.3	99.8	98.7	101.05	1.023809524	0.455641
Q9ULW0	TPX2	Targeting protein for Xklp2 OS=Homo sapier	16	98.1	99.4	100	102.2	98.75	101.1	1.023797468	0.455768
P67936	TPM4	Tropomyosin alpha-4 chain OS=Homo sapien	6	99.9	102	102.2	104.5	100.95	103.35	1.023774146	0.456014
Q96KM6	ZNF512B	Zinc finger protein 512B OS=Homo sapiens (	2	97.9	96.8	97.2	102.1	97.35	99.65	1.023626091	0.457578
P82933	MRPS9	28S ribosomal protein S9, mitochondrial OS=	17	100.2	98.8	100.9	102.8	99.5	101.85	1.02361809	0.457662
B4E2B5		MICOS complex subunit MIC60 OS=Homo s	2	104.5	94.9	99.9	104.2	99.7	102.05	1.023570712	0.458164
Q6Y288	B3GLCT	Beta-1,3-glucosyltransferase OS=Homo sapie	1	96.5	103.2	99.9	104.5	99.85	102.2	1.023535303	0.458539
A4D0W0	LSM8	LSM8 homolog, U6 small nuclear RNA assoc	4	94	97.6	99.6	96.5	95.8	98.05	1.02348643	0.459056
B2R9Y2		cDNA, FLJ94609 OS=Homo sapiens PE=2 S	4	96.4	99.6	97.7	102.9	98	100.3	1.023469388	0.459237
Q9UMX5	NENF	Neudesin OS=Homo sapiens GN=NENF PE=	8	96	104.6	102.9	102.4	100.3	102.65	1.023429711	0.459656
Q16740	CLPP	ATP-dependent Clp protease proteolytic subu	10	96.5	95.8	98.6	98.2	96.15	98.4	1.023400936	0.459962
Q7Z4X2		Neuronal protein OS=Homo sapiens PE=2 SV	4	98.6	98.1	99.3	102	98.35	100.65	1.023385867	0.460121
E7EPK1	SEPT7	Septin-7 OS=Homo sapiens GN=SEPT7 PE=	17	104.3	97.3	104.6	101.7	100.8	103.15	1.023313492	0.460889
P13645	KRT10	Keratin, type I cytoskeletal 10 OS=Homo sap	15	100.7	105.2	103.7	107	102.95	105.35	1.023312288	0.460902
B4DEF8		cDNA FLJ61100, highly similar to 39S ribos	11	100.9	98.1	102.9	100.7	99.5	101.8	1.023115578	0.462993
Q9HDC9	APMAP	Adipocyte plasma membrane-associated prote	16	100.9	98.2	101.8	101.9	99.55	101.85	1.023103968	0.463118
A0A024R2W	DAG1	Dystroglycan 1 (Dystrophin-associated glycop	9	104.5	99	105.4	102.8	101.75	104.1	1.023095823	0.463204
Q6IN84	MRM1	rRNA methyltransferase 1, mitochondrial OS	1	101.2	98	101.7	102.1	99.6	101.9	1.023092369	0.463241
Q9H9A5	CNOT10	CCR4-NOT transcription complex subunit 10	1	109.2	90.3	99.4	104.7	99.75	102.05	1.023057644	0.46361
P25685	DNAJB1	DnaJ homolog subfamily B member 1 OS=Ho	11	95.1	100.6	98.6	101.6	97.85	100.1	1.022994379	0.464285
A0A140TA86	C19orf70	MICOS complex subunit MIC13 OS=Homo s	2	98.7	93.2	99.3	97	95.95	98.15	1.022928609	0.464987
P30043	BLVRB	Flavin reductase (NADPH) OS=Homo sapien	7	97.2	99.2	103.5	97.4	98.2	100.45	1.022912424	0.46516
P08236	GUSB	Beta-glucuronidase OS=Homo sapiens GN=C	5	95.6	100.8	101.3	99.6	98.2	100.45	1.022912424	0.46516
Q12846	STX4	Syntaxin-4 OS=Homo sapiens GN=STX4 PE	6	103.6	97.2	103.6	101.8	100.4	102.7	1.022908367	0.465203
Q8IYB8	SUPV3L1	ATP-dependent RNA helicase SUPV3L1, mit	12	101.8	95.5	102.1	99.7	98.65	100.9	1.022807907	0.466276
B4DLH1		cDNA FLJ55982, highly similar to Homo sap	2	100.3	92.9	98.5	99.1	96.6	98.8	1.022774327	0.466635
B2R761		cDNA, FLJ93299, highly similar to Homo sap	18	96.6	101.1	101	101.2	98.85	101.1	1.02276176	0.466771
B2RMV2	CYTSA	CYTSA protein OS=Homo sapiens GN=CYT	8	98.6	99.2	98.9	103.4	98.9	101.15	1.022750253	0.466893
B0QYN7	UBE2I	SUMO-conjugating enzyme OS=Homo sapier	8	97.2	100.9	100.5	102.1	99.05	101.3	1.0227158	0.467262
P50213	IDH3A	Isocitrate dehydrogenase [NAD] subunit alpha	16	100.1	98.5	102.2	100.9	99.3	101.55	1.02265861	0.467875
H7BZJ3	PDIA3	Protein disulfide-isomerase A3 (Fragment) O	1	98.1	91.8	93.8	100.4	94.95	97.1	1.022643497	0.468037
Q9NQC3	RTN4	Reticulon-4 OS=Homo sapiens GN=RTN4 PI	3	102	96.9	98.3	105.1	99.45	101.7	1.022624434	0.46824
Q9NUD5	ZCCHC3	Zinc finger CCHC domain-containing protein	4	102.1	101.4	104.4	103.7	101.75	104.05	1.022604423	0.468455
A0A024R8W	DDX48	DEAD (Asp-Glu-Ala-Asp) box polypeptide 4	22	97.9	101.2	102.8	100.8	99.55	101.8	1.022601708	0.468484
J3KTL2	SRSF1	Serine/arginine-rich-splicing factor 1 OS=Hor	16	97.4	97.4	99.1	100.1	97.4	99.6	1.022587269	0.468639
H3BLT4	BIRC5	Baculoviral IAP repeat-containing protein 5 C	3	99.9	99.5	93.9	110	99.7	101.95	1.022567703	0.468848
B2R4I8		cDNA, FLJ92106, highly similar to Homo sap	2	103.7	95.8	95.5	108.5	99.75	102	1.022556391	0.46897
P62330	ARF6	ADP-ribosylation factor 6 OS=Homo sapiens	5	102.5	101.9	106.3	102.7	102.2	104.5	1.022504892	0.469523
P30837	ALDH1B1	Aldehyde dehydrogenase X, mitochondrial O	15	96.2	99.6	101.7	98.5	97.9	100.1	1.02247191	0.469877
H0Y405	ST8SIA6	Alpha-2,8-sialyltransferase 8F (Fragment) OS	1	106.6	84.8	91.3	104.4	95.7	97.85	1.02246604	0.46994
A0A0C4DG17	RPSA	40S ribosomal protein SA OS=Homo sapiens	14	98.5	101.9	101.5	103.4	100.2	102.45	1.02245509	0.470058
P20248	CCNA2	Cyclin-A2 OS=Homo sapiens GN=CCNA2 P	1	96.7	94.8	87.2	108.6	95.75	97.9	1.022454308	0.470067
Q32P28	P3H1	Prolyl 3-hydroxylase 1 OS=Homo sapiens GN	21	100.1	100.6	103.2	102	100.35	102.6	1.022421525	0.470419
Q8NBS9	TXNDC5	Thioredoxin domain-containing protein 5 OS=	18	99.3	101.6	103.1	102.3	100.45	102.7	1.022399204	0.470659
Q9NR30	DDX21	Nucleolar RNA helicase 2 OS=Homo sapiens	41	98	98.5	101.7	99.2	98.25	100.45	1.022391858	0.470737
P31930	UQCRC1	Cytochrome b-c1 complex subunit 1, mitocho	18	98.3	98.3	100.1	100.9	98.3	100.5	1.022380468	0.47086
A8K4W2		cDNA FLJ78635, highly similar to Homo sap	13	98.5	98.4	103.1	98.2	98.45	100.65	1.022346369	0.471227
Q96BK5	PINX1	PIN2/TERF1-interacting telomerase inhibitor	4	96.1	100.9	98.1	103.3	98.5	100.7	1.022335025	0.471349
A0A173GMX	ACTC1	Actin alpha cardiac muscle 1 (Fragment) OS=	1	101.5	95.5	96.4	105	98.5	100.7	1.022335025	0.471349
Q8N3Z3	GTPBP8	GTP-binding protein 8 OS=Homo sapiens GN	4	99.7	97.7	99.5	102.3	98.7	100.9	1.022289767	0.471836
A0A024R7B7	CDC37	CDC37 cell division cycle 37 homolog (S. cer	10	98.9	98.9	101.5	100.7	98.9	101.1	1.022244692	0.472322
B4DQ06		cDNA FLJ51932 OS=Homo sapiens PE=2 SV	1	97.1	101.2	94.7	108	99.15	101.35	1.022188603	0.472927
Q9Y2S7	POLDIP2	Polymerase delta-interacting protein 2 OS=Ho	12	97	101.3	100	102.7	99.15	101.35	1.022188603	0.472927
A8K3X7		cDNA FLJ77081, highly similar to Homo sap	1	93.5	100.5	92.3	106	97	99.15	1.022164948	0.473181
Q15057	ACAP2	Arf-GAP with coiled-coil, ANK repeat and PI	3	93.9	100.6	97.1	101.7	97.25	99.4	1.022107969	0.473796
Q8N5M4	TTC9C	Tetratricopeptide repeat protein 9C OS=Homo	1	97	97.9	92.1	107.1	97.45	99.6	1.022062596	0.474286
Q9BX40	LSM14B	Protein LSM14 homolog B OS=Homo sapien	4	100.8	99.6	103.3	101.5	100.2	102.4	1.021956088	0.475437
P13797	PLS3	Plastin-3 OS=Homo sapiens GN=PLS3 PE=1	18	102.3	98.1	103.9	100.9	100.2	102.4	1.021956088	0.475437

A6NMH8	CD81	Tetraspanin OS=Homo sapiens GN=CD81 PE	3	105.9	99.2	108.1	101.5	102.55	104.8	1.021940517	0.475606
P52294	KPNA1	Importin subunit alpha-5 OS=Homo sapiens C	4	96.4	100.1	95.6	105.2	98.25	100.4	1.021882952	0.476228
Q9HBH0	RHOF	Rho-related GTP-binding protein RhoF OS=H	2	104.3	97	101	104.7	100.65	102.85	1.021857923	0.476499
P61163	ACTR1A	Alpha-centractin OS=Homo sapiens GN=ACT	5	102.8	99	103.5	102.7	100.9	103.1	1.021803766	0.477087
J3KN01	AFDN	Afadin OS=Homo sapiens GN=AFDN PE=1	8	101.6	100.2	100.1	106.1	100.9	103.1	1.021803766	0.477087
O94887	FARP2	FERM, RhoGEF and pleckstrin domain-conta	2	100.4	96.9	104.4	97.2	98.65	100.8	1.021794222	0.47719
Q9BWJ5	SF3B5	Splicing factor 3B subunit 5 OS=Homo sapien	4	99.4	102.5	106.2	100.1	100.95	103.15	1.021792967	0.477203
A0A024R9D7	DECR1	2,4-dienoyl CoA reductase 1, mitochondrial, i	10	98.8	98.6	100.3	101.4	98.7	100.85	1.021783181	0.477309
P15880	RPS2	40S ribosomal protein S2 OS=Homo sapiens	14	99.1	102.9	105.5	100.9	101	103.2	1.021782178	0.477321
B4E106		cDNA FLJ53399, highly similar to Monocarb	5	102.8	99.4	104.3	102.3	101.1	103.3	1.021760633	0.477555
E5KSL5	DGUOK	Deoxyguanosine kinase isoform 1 OS=Homo	3	98.3	99.5	101.3	100.8	98.9	101.05	1.02173913	0.477787
P05023	ATP1A1	Sodium/potassium-transporting ATPase subu	29	103.1	99.6	104.9	102.2	101.35	103.55	1.021706956	0.478136
B2R7T8		cDNA, FLJ93598, highly similar to Homo sap	16	99.7	98.4	102.1	100.3	99.05	101.2	1.021706209	0.478144
P49792	RANBP2	E3 SUMO-protein ligase RanBP2 OS=Homo	66	99.6	99	102.3	100.6	99.3	101.45	1.021651561	0.478738
Q12972	PPP1R8	Nuclear inhibitor of protein phosphatase 1 OS	8	99.1	99.7	99.3	103.8	99.4	101.55	1.021629779	0.478974
Q8IWA0	WDR75	WD repeat-containing protein 75 OS=Homo s	20	99.6	99.6	102.7	100.8	99.6	101.75	1.021586345	0.479447
P61020	RAB5B	Ras-related protein Rab-5B OS=Homo sapien	6	99.8	99.9	102.5	101.5	99.85	102	1.021532298	0.480034
P63218	GNG5	Guanine nucleotide-binding protein G(I)/G(S)	2	100	99.8	104.7	99.4	99.9	102.05	1.021521522	0.480151
Q8NCN5	PDPR	Pyruvate dehydrogenase phosphatase regulato	12	98.1	98.9	102	99.2	98.5	100.6	1.021319797	0.482349
A0A024RC47	ZNF24	Zinc finger protein 24 (KOX 17), isoform CR	1	93.3	94.4	92.9	98.8	93.85	95.85	1.021310602	0.48245
Q8IX12	CCAR1	Cell division cycle and apoptosis regulator pr	18	99.7	97.4	100.7	100.6	98.55	100.65	1.02130898	0.482466
Q8TF72	SHROOM3	Protein Shroom3 OS=Homo sapiens GN=SHI	9	100.7	101.1	102	104.1	100.9	103.05	1.021308226	0.482476
P29590	PML	Protein PML OS=Homo sapiens GN=PML PE	8	99.2	98	100.8	100.6	98.6	100.7	1.021298174	0.482585
P62072	TIMM10	Mitochondrial import inner membrane translo	7	102.4	95.3	100	101.9	98.85	100.95	1.02124431	0.483173
Q02880	TOP2B	DNA topoisomerase 2-beta OS=Homo sapien	6	101.4	96.8	100.4	102	99.1	101.2	1.021190716	0.483758
J3KNN5	DDX41	Probable ATP-dependent RNA helicase DDX	17	96.8	96.9	96.1	101.7	96.85	98.9	1.021166753	0.48402
A0A024RA75	HIBADH	3-hydroxyisobutyrate dehydrogenase OS=Ho	8	100.9	98.2	98.6	104.7	99.55	101.65	1.021094927	0.484806
P54753	EPHB3	Ephrin type-B receptor 3 OS=Homo sapiens C	1	99.8	95.4	91.5	107.8	97.6	99.65	1.021004098	0.4858
Q13541	EIF4EBP1	Eukaryotic translation initiation factor 4E-bin	1	95.9	94.8	93.3	101.4	95.35	97.35	1.020975354	0.486115
A0A024R0Z3	DDX23	DEAD (Asp-Glu-Ala-Asp) box polypeptide 2	27	96.9	99	100.1	99.9	97.95	100	1.020929045	0.486622
Q8NBQ5	HSD17B11	Estradiol 17-beta-dehydrogenase 11 OS=Hon	13	98.1	102.7	105.6	99.4	100.4	102.5	1.020916335	0.486762
Q6NS38	ALKBH2	DNA oxidative demethylase ALKBH2 OS=H	1	95.9	95.4	91.4	103.9	95.65	97.65	1.020909566	0.486836
E9KL35		Epididymis tissue sperm binding protein Li 3a	22	98.9	102.1	101.5	103.7	100.5	102.6	1.020895522	0.486991
B3KNP2		cDNA FLJ30079 fis, clone BGGI12000544, h	1	97	99.3	104.5	95.9	98.15	100.2	1.020886398	0.48709
A0A024R9D9	ENY2	Transcription and mRNA export factor ENY2	1	106.9	94.8	102.1	103.8	100.85	102.95	1.020823004	0.487786
Q8TAV0	FAM76A	Protein FAM76A OS=Homo sapiens GN=FA	1	95.7	111.7	105.7	106	103.7	105.85	1.020732883	0.488776
O43491	EPB41L2	Band 4.1-like protein 2 OS=Homo sapiens GI	25	102.8	99.9	105.1	101.8	101.35	103.45	1.020720276	0.488915
Q969V3	NCLN	Nicalin OS=Homo sapiens GN=NCLN PE=1	13	99.7	98.2	101.3	100.7	98.95	101	1.020717534	0.488945
Q4LE51	PIK3CA	PIK3CA variant protein (Fragment) OS=Hom	1	105.2	97.8	107.7	99.5	101.5	103.6	1.020689655	0.489252
Q5T5C7	SARS	Serine--tRNA ligase, cytoplasmic OS=Homo	9	100.7	98.2	101.5	101.5	99.45	101.5	1.020613374	0.49009
Q53F62		ADP-ribosylation factor GTPase activating pr	7	96.9	102.1	100.1	103	99.5	101.55	1.020603015	0.490205
Q96RQ3	MCCC1	Methylcrotonoyl-CoA carboxylase subunit al	17	98.4	101	102.2	101.3	99.7	101.75	1.020561685	0.49066
B4DI08		cDNA FLJ60091, highly similar to Hypoxia-i	1	95.3	94.7	92.4	101.5	95	96.95	1.020526316	0.49105
Q49B96	COX19	Cytochrome c oxidase assembly protein COX	1	101.5	93.9	99.4	100	97.7	99.7	1.020470829	0.491662
P16989	YBX3	Y-box-binding protein 3 OS=Homo sapiens C	4	97.4	98.3	95.9	103.8	97.85	99.85	1.020439448	0.492008
A8K7T4		cDNA FLJ75774, highly similar to Homo sap	15	100.7	100.1	104.1	100.8	100.4	102.45	1.020418327	0.492242
Q3ZCS4	PABPC3	Polyadenylate-binding protein OS=Homo sap	3	99.4	101.7	103.1	102.1	100.55	102.6	1.020387867	0.492578
Q8WUD1	RAB2B	Ras-related protein Rab-2B OS=Homo sapien	1	100.6	100.6	101.1	104.2	100.6	102.65	1.020377734	0.492689
A0A024R3X9	PREI3	Preimplantation protein 3, isoform CRA_a OS	7	98.6	97.7	97.5	102.8	98.15	100.15	1.020376974	0.492699
Q9BTU6	PI4K2A	Phosphatidylinositol 4-kinase type 2-alpha OS	9	99.4	97.3	98.4	102.3	98.35	100.35	1.020335536	0.493156
A0A0S2Z5H3	CLINT1	Clathrin interactor 1 isoform 2 (Fragment) OS	10	101.9	99.9	105.7	100.2	100.9	102.95	1.020317146	0.493359
O75410	TACC1	Transforming acidic coiled-coil-containing pr	2	112.3	89.5	95.7	110.2	100.9	102.95	1.020317146	0.493359
B9EGQ5	WIZ	Protein Wiz OS=Homo sapiens GN=WIZ PE=	1	89.7	103.1	109.8	86.9	96.4	98.35	1.020228216	0.494342
Q12769	NUP160	Nuclear pore complex protein Nup160 OS=H	24	100	97.8	102.7	99.1	98.9	100.9	1.020222447	0.494407
A0A024R6U0	RSPRY1	Ring finger and SPRY domain containing 1, i	4	107.4	95.5	100.6	106.4	101.45	103.5	1.020206999	0.494577
Q14684	RRP1B	Ribosomal RNA processing protein 1 homolo	27	99.2	98.8	102.2	99.8	99	101	1.02020202	0.494632
Q96AQ8	MCUR1	Mitochondrial calcium uniporter regulator 1 C	2	105.1	93.1	100.4	101.8	99.1	101.1	1.020181635	0.494858
B4DYA5		cDNA FLJ59637, highly similar to Mitochon	1	97.6	100.9	97.6	104.9	99.25	101.25	1.020151134	0.495196
A0A024R491	C2orf33	Chromosome 2 open reading frame 33, isofor	8	97.5	101.5	99.6	103.4	99.5	101.5	1.020100503	0.495757
Q15691	MAPRE1	Microtubule-associated protein RP/EB family	15	96.3	102.7	102	101	99.5	101.5	1.020100503	0.495757
P62861	FAU	40S ribosomal protein S30 OS=Homo sapiens	1	93	111.4	105.8	102.7	102.2	104.25	1.020058708	0.49622
B2R856		Acyl-coenzyme A oxidase OS=Homo sapiens	6	101.3	93.3	102.2	96.3	97.3	99.25	1.02004111	0.496416
B4DPY0		Glutathione peroxidase OS=Homo sapiens PE	1	95.7	104.5	97.6	106.6	100.1	102.1	1.01998002	0.497093
P78527	PRKDC	DNA-dependent protein kinase catalytic subu	155	99	101.2	103.7	100.5	100.1	102.1	1.01998002	0.497093
Q9NQZ2	UTP3	Something about silencing protein 10 OS=Ho	7	100.6	99.7	103.3	101	100.15	102.15	1.019970045	0.497204
B2R6S9		cDNA, FLJ93097, highly similar to Homo sap	20	99.2	101.5	102.8	101.9	100.35	102.35	1.019930244	0.497646
Q9UGL1	KDM5B	Lysine-specific demethylase 5B OS=Homo sa	1	96.3	99.4	99.5	100.1	97.85	99.8	1.019928462	0.497666
A0A0S2Z4G8	TPM3	Tropomyosin 3 isoform 2 OS=Homo sapiens	1	100.6	105.5	101.9	108.3	103.05	105.1	1.019893256	0.498057
Q16134	ETFDH	Electron transfer flavoprotein-ubiquinone oxi	6	103.6	97.5	102.1	103	100.55	102.55	1.019890602	0.498087
Q8N1G4	LRRC47	Leucine-rich repeat-containing protein 47 OS	11	100.1	101.2	103.4	101.9	100.65	102.65	1.01987084	0.498306
Q96BP3	PPWD1	Peptidylprolyl isomerase domain and WD rep	8	97.9	98.4	98.9	101.3	98.15	100.1	1.01986755	0.498343
Q53SS8	PCBP1	Epididymis secretory protein Li 85 OS=Hom	11	96.6	99.7	97.9	102.3	98.15	100.1	1.01986755	0.498343
O15400	STX7	Syntaxin-7 OS=Homo sapiens GN=STX7 PE	8	100	97	98.8	102.1	98.5	100.45	1.019796954	0.499128
B3KP90		cDNA FLJ31447 fis, clone NT2NE2000913,	2	99.5	102.7	103	103.2	101.1	103.1	1.019782394	0.49929
Q8TCG2	PI4K2B	Phosphatidylinositol 4-kinase type 2-beta OS	2	104.1	94.4	98	104.4	99.25	101.2	1.019647355	0.500794
O60493	SNX3	Sorting nexin-3 OS=Homo sapiens GN=SNX	6	99.8	99	101.5	101.2	99.4	101.35	1.019617706	0.501125
O75323	GBAS	Protein NipSnap homolog 2 OS=Homo sapier	5	98.7	100.3	103.9	99	99.5	101.45	1.01959799	0.501345
Q8N2F6	ARMC10	Armadillo repeat-containing protein 10 OS=H	4	102.7	96.5	105.5	97.6	99.6	101.55	1.019578313	0.501564
Q9P0M6	H2AFY2	Core histone macro-H2A.2 OS=Homo sapiens	17	99.5	100	102.8	100.6	99.75	101.7	1.019548872	0.501893
Q06609	RAD51	DNA repair protein RAD51 homolog 1 OS=H	1	95.7	98.8	100.6	97.7	97.25	99.15	1.019537275	0.502022
A8K122	KIAA1946	KIAA1946, isoform CRA_b OS=Homo sapie	4	94.1	105.9	103.2	100.7	100	101.95	1.0195	0.502439
Q6ZNA5	FRRS1	Ferric-chelate reductase 1 OS=Homo sapiens	2	103.2	96.9	104	100	100.05	102	1.019490255	0.502548
Q9BTL3	FAM103A1	RNMT-activating mini protein OS=Homo sap	1	94.8	95.6	88.6	105.5	95.2	97.05	1.019432773	0.50319
O95140	MFN2	Mitofusin-2 OS=Homo sapiens GN=MFN2 P	17	98.1	97.7	99.4	100.2	97.9	99.8	1.019407559	0.503473
A0A024RAM(	TNPO1	Transportin 1, isoform CRA_a OS=Homo sap	16	100.5	101.2	101.8	103.8	100.85	102.8	1.019335647	0.504277
O00186	STXBP3	Syntaxin-binding protein 3 OS=Homo sapien	17	100.8	100.9	103.5	102.1	100.85	102.8	1.019335647	0.504277
Q15906	VPS72	Vacuolar protein sorting-associated protein 72	4	99.2	97.5	97.7	102.8	98.35	100.25	1.01931876	0.504465
Q03164	KMT2A	Histone-lysine N-methyltransferase 2A OS=H	6	101.4	95.7	96.8	104.1	98.55	100.45	1.019279554	0.504904
Q76LA1	CSTB	CSTB protein OS=Homo sapiens GN=CSTB	4	90.6	96.2	94.1	96.3	93.4	95.2	1.019271949	0.50499
A0A024R3D8	DLAT	Acetyltransferase component of pyruvate deh	22	99.1	98.3	102.1	99.1	98.7	100.6	1.019250253	0.505233
Q9P287	BCCIP	BRCA2 and CDKN1A-interacting protein OS	9	98.1	99.8	100.3	101.4	98.95	100.85	1.019201617	0.505778
P78310	CXADR	Coxsackievirus and adenovirus receptor OS=I	2	104.1	99.5	105.8	101.7	101.8	103.75	1.019155206	0.506298
A0A024R6D8	SFRS5	Splicing factor, arginine/serine-rich 5, isoforn	8	98.5	99.9	101.3	100.9	99.2	101.1	1.019153226	0.506321
Q59EK3		Adaptor-related protein complex 1, mu 1 subu	12	96.7	96.6	97.7	99.3	96.65	98.5	1.019141231	0.506456
Q15427	SF3B4	Splicing factor 3B subunit 4 OS=Homo sapier	3	99.4	99.3	101.6	100.9	99.35	101.25	1.019124308	0.506646
Q9Y2R4	DDX52	Probable ATP-dependent RNA helicase DDX	10	99.7	99.1	96.1	106.5	99.4	101.3	1.019114688	0.506753
Q5SRE5	NUP188	Nucleoporin NUP188 homolog OS=Homo sap	19	99.7	99.1	102.3	100.3	99.4	101.3	1.019114688	0.506753
Q6NT76	HMBOX1	Homeobox-containing protein 1 OS=Homo sa	1	92	96.4	91.5	100.5	94.2	96	1.01910828	0.506825
A0A024R2V1	hCG_20123	HCG20123, isoform CRA_b OS=Homo sapie	1	94.8	93.8	96.6	95.6	94.3	96.1	1.019088017	0.507053
B4E1N4		cDNA FLJ61695, highly similar to Glioma tu	8	100.8	98.3	101.7	101.2	99.55	101.45	1.019085886	0.507077
M0R226	MRPL34	39S ribosomal protein L34, mitochondrial OS	2	99.2	100	101.3	101.7	99.6	101.5	1.019076305	0.507184
O75940	SMNDC1	Survival of motor neuron-related-splicing fact	4	99	100.3	103.8	99.3	99.65	101.55	1.019066734	0.507292
Q96CS3	FAF2	FAS-associated factor 2 OS=Homo sapiens G	10	101.4	98.7	103.4	100.5	100.05	101.95	1.018990505	0.508148
Q02241	KIF23	Kinesin-like protein KIF23 OS=Homo sapien	23	102.7	97.6	102.7	101.4	100.15	102.05	1.018971543	0.508361
Q15036	SNX17	Sorting nexin-17 OS=Homo sapiens GN=SN	6	101	99.8	105.4	99.2	100.4	102.3	1.018924303	0.508892
Q96L91	EP400	E1A-binding protein p400 OS=Homo sapiens	11	100.4	95.3	100.1	99.3	97.85	99.7	1.01890649	0.509094
O95684	FGFR1OP	FGFR1 oncogene partner OS=Homo sapiens	5	99.2	96.6	102	97.5	97.9	99.75	1.018896834	0.509202
Q6ZMF6		Glycerol-3-phosphate acyltransferase 1, mito	1	91.4	109.7	100.1	104.8	100.55	102.45	1.018896072	0.50921
A0A024R814	RPL7	Ribosomal protein L7, isoform CRA_a OS=H	18	98.2	103.1	103.1	102	100.65	102.55	1.018877298	0.509422
J3KQN4	RPL36A	60S ribosomal protein L36a OS=Homo sapier	2	94.7	107.9	102.9	103.5	101.3	103.2	1.01875617	0.510787
A0A024RCY(	C6orf89	Chromosome 6 open reading frame 89, isofor	3	96.6	101	97.8	103.5	98.8	100.65	1.018724696	0.511142
P52948	NUP98	Nuclear pore complex protein Nup98-Nup96	30	100.2	98	102.4	99.5	99.1	100.95	1.018668012	0.511781
Q9H920	RNF121	RING finger protein 121 OS=Homo sapiens C	2	95.2	97.8	99.4	97.2	96.5	98.3	1.01865285	0.511953
P61244	MAX	Protein max OS=Homo sapiens GN=MAX PE	2	98.3	100.2	104.4	97.8	99.25	101.1	1.018639798	0.5121
A0A024R0R4	SAE1	SUMO-1 activating enzyme subunit 1, isoforn	10	97.7	101.1	102.5	100	99.4	101.25	1.01861167	0.512418
Q96ST2	IWS1	Protein IWS1 homolog OS=Homo sapiens GI	5	95.6	99.1	99.4	98.9	97.35	99.15	1.018489985	0.513793
Q8NE86	MCU	Calcium uniporter protein, mitochondrial OS=	5	99.3	95.9	98.4	100.4	97.6	99.4	1.018442623	0.514329
P46940	IQGAP1	Ras GTPase-activating-like protein IQGAP1	60	101.1	99.6	102.8	101.6	100.35	102.2	1.018435476	0.51441
Q08257	CRYZ	Quinone oxidoreductase OS=Homo sapiens G	12	95.3	100.1	99.1	99.9	97.7	99.5	1.018423746	0.514542
P62136	PPP1CA	Serine/threonine-protein phosphatase PP1-alp	4	98.6	102.6	101.8	103.1	100.6	102.45	1.018389662	0.514928
Q13895	BYSL	Bystin OS=Homo sapiens GN=BYSL PE=1 S	12	98.4	98	98.5	101.5	98.2	100	1.018329939	0.515605
Q8N7G1		Purine nucleoside phosphorylase OS=Homo s	13	95.6	102.3	100.3	101.2	98.95	100.75	1.018191006	0.517182

Q9H3S7	PTPN23	Tyrosine-protein phosphatase non-receptor ty	6	96.8	101.3	100	101.7	99.05	100.85	1.01817264	0.517391
P34897	SHMT2	Serine hydroxymethyltransferase, mitochondr	6	99.9	98.3	100.4	101.4	99.1	100.9	1.018163471	0.517495
Q7Z7H5	TMED4	Transmembrane emp24 domain-containing pr	6	99.1	101.4	102.8	101.3	100.25	102.05	1.017955112	0.519865
P49916	LIG3	DNA ligase 3 OS=Homo sapiens GN=LIG3 F	5	105.6	94.9	106	98.1	100.25	102.05	1.017955112	0.519865
Q9NUG6	PDRG1	p53 and DNA damage-regulated protein 1 OS	1	100.1	100.6	101.3	103	100.35	102.15	1.01793722	0.520068
Q9H444	CHMP4B	Charged multivesicular body protein 4b OS=I	6	100.8	100.2	101.2	103.4	100.5	102.3	1.017910448	0.520373
Q9BSH4	TACO1	Translational activator of cytochrome c oxida:	5	97	98.5	100.1	98.9	97.75	99.5	1.017902813	0.52046
P46087	NOP2	Probable 28S rRNA (cytosine(4447)-C(5))-m	29	98.6	97.1	99.8	99.4	97.85	99.6	1.017884517	0.520669
B4DJL6	VWA9	cDNA FLJ55475 OS=Homo sapiens GN=VW	2	92.9	108.6	110.3	94.8	100.75	102.55	1.017866005	0.520879
Q4W4Y1	DRIP4	Dopamine receptor interacting protein 4 OS=I	31	98.1	98.8	100.7	99.7	98.45	100.2	1.017775521	0.521911
Q9Y570	PPME1	Protein phosphatase methylesterase 1 OS=Ho	8	96.6	100.4	100.1	100.4	98.5	100.25	1.017766497	0.522015
Q59E85		Caveolin (Fragment) OS=Homo sapiens PE=2	1	111.2	92.1	102.2	104.7	101.65	103.45	1.017707821	0.522685
Q9UBU9	NXF1	Nuclear RNA export factor 1 OS=Homo sapie	12	101.3	96.4	102.3	98.9	98.85	100.6	1.017703591	0.522734
A0A024R9M9	CHP	Calcium binding protein P22, isoform CRA_a	8	99.5	98.5	99.3	102.2	99	100.75	1.017676768	0.52304
P23246	SFPQ	Splicing factor, proline- and glutamine-rich O	26	98.8	99.2	100.7	100.8	99	100.75	1.017676768	0.52304
Q9H8H2	DDX31	Probable ATP-dependent RNA helicase DDX	15	101.2	97.2	100.5	101.4	99.2	100.95	1.017641129	0.523446
A0A140VKA6		Testis secretory sperm-binding protein Li 233	10	97.4	101.2	100.3	101.8	99.3	101.05	1.017623364	0.523651
A0A024R4N0	hCG_1640809	HCG1640809, isoform CRA_b OS=Homo sap	3	109.7	94.8	105	103.1	102.25	104.05	1.017603912	0.523873
Q4KMQ2	ANO6	Anoctamin-6 OS=Homo sapiens GN=ANO6	4	100.3	99.5	101.4	101.9	99.9	101.65	1.017517518	0.52486
Q12962	TAF10	Transcription initiation factor TFIID subunit 1	3	95.7	98.5	101.2	96.4	97.1	98.8	1.017507724	0.524974
A0A087X020	SBDS	Ribosome maturation protein SBDS OS=Hom	10	99.4	100.6	102.6	100.9	100	101.75	1.0175	0.525061
P0C0S5	H2AFZ	Histone H2A.Z OS=Homo sapiens GN=H2AI	2	89.8	93.7	90.5	96.2	91.75	93.35	1.017438692	0.525764
Q9NVM9	ASUN	Protein asunder homolog OS=Homo sapiens (	7	96.5	98.5	99.6	98.8	97.5	99.2	1.017435897	0.525796
I6L975	HSDL1	Hydroxysteroid dehydrogenase like 1 OS=Ho	6	100	101.1	104.3	100.3	100.55	102.3	1.017404276	0.526158
P25398	RPS12	40S ribosomal protein S12 OS=Homo sapiens	9	99.9	101.3	101.8	102.9	100.6	102.35	1.017395626	0.526258
P52298	NCBP2	Nuclear cap-binding protein subunit 2 OS=Ho	3	99.3	102.1	103.6	101.3	100.7	102.45	1.017378352	0.526456
O95625	ZBTB11	Zinc finger and BTB domain-containing prote	4	102.9	98.5	102.3	102.6	100.7	102.45	1.017378352	0.526456
Q9Y6D0	SELK	Selenoprotein K OS=Homo sapiens GN=SEL	1	103.3	92.4	98.7	100.4	97.85	99.55	1.017373531	0.52651
Q09161	NCBP1	Nuclear cap-binding protein subunit 1 OS=Ho	15	102.8	98.7	101.7	103.3	100.75	102.5	1.017369727	0.526554
F5H018	RAN	GTP-binding nuclear protein Ran (Fragment)	1	87.2	108.7	99.8	99.5	97.95	99.65	1.017355794	0.526714
P36873	PPP1CC	Serine/threonine-protein phosphatase PP1-gar	2	95.8	106	105.3	100	100.9	102.65	1.017343905	0.526851
P61204	ARF3	ADP-ribosylation factor 3 OS=Homo sapiens	5	98.6	97.7	100.9	98.8	98.15	99.85	1.017320428	0.52712
A0A024R7M0	TMED9	Transmembrane emp24 protein transport dom	7	96.4	94.2	95.4	98.5	95.3	96.95	1.017313746	0.527197
Q08211	DHX9	ATP-dependent RNA helicase A OS=Homo s	56	99.2	98.8	101.7	99.7	99	100.7	1.017171717	0.528828
Q6NX51	EXOC4	Exocyst complex component 4 OS=Homo sap	3	99.3	98.7	98.3	103.1	99	100.7	1.017171717	0.528828
P56282	POLE2	DNA polymerase epsilon subunit 2 OS=Homo	2	98.5	99.8	99	102.7	99.15	100.85	1.017145739	0.529127
P62304	SNRPE	Small nuclear ribonucleoprotein E OS=Homo	4	97.5	101.8	99.4	103.3	99.65	101.35	1.017059709	0.530117
Q8IXM3	MRPL41	39S ribosomal protein L41, mitochondrial OS	6	100.3	100.1	102.8	101	100.2	101.9	1.016966068	0.531195
H0Y9V7	ATP2C1	Calcium-transporting ATPase type 2C membe	1	94.9	100.6	89.9	108.9	97.75	99.4	1.016879795	0.53219
A2A3R6	RPS6	40S ribosomal protein S6 OS=Homo sapiens (	11	98	104.3	103.8	101.9	101.15	102.85	1.01680 6723	0.533034
Q00537	CDK17	Cyclin-dependent kinase 17 OS=Homo sapier	1	101.6	94.8	98.1	101.6	98.2	99.85	1.016802444	0.533083
Q93034	CUL5	Cullin-5 OS=Homo sapiens GN=CUL5 PE=1	7	97.2	99.2	97.3	102.4	98.2	99.85	1.016802444	0.533083
A0A024RD03	MRPS10	Mitochondrial ribosomal protein S10, isoform	5	96.5	94.1	95.3	98.5	95.3	96.9	1.016789087	0.533238
P46013	MKI67	Proliferation marker protein Ki-67 OS=Homo	96	99.1	97.6	100.6	99.4	98.35	100	1.016776817	0.533379
Q6IAZ5	CRSP9	CRSP9 protein OS=Homo sapiens GN=CRSF	1	100.4	90.6	96.3	97.9	95.5	97.1	1.016753927	0.533644
Q9UJZ1	STOML2	Stomatin-like protein 2, mitochondrial OS=H	16	100.2	97.4	99.8	101.1	98.8	100.45	1.016700405	0.534263
Q15819	UBE2V2	Ubiquitin-conjugating enzyme E2 variant 2 O	2	101.1	96.8	102	99.2	98.95	100.6	1.016675088	0.534555
O43823	AKAP8	A-kinase anchor protein 8 OS=Homo sapiens	11	98.6	99.4	100.2	101.1	99	100.65	1.016666667	0.534653
Q9H0U6	MRPL18	39S ribosomal protein L18, mitochondrial OS	4	101.4	96.8	102.3	99.2	99.1	100.75	1.016649849	0.534847
X6R5Z6	COA6	Cytochrome c oxidase assembly factor 6 hom	3	100.2	98.2	100.7	101	99.2	100.85	1.016633065	0.535042
A0A0A0MRK	MTX1	Metaxin 1, isoform CRA_b OS=Homo sapien	7	100.4	98.2	102.4	99.5	99.3	100.95	1.016616314	0.535235
A0A024R7T3	HNRPF	Heterogeneous nuclear ribonucleoprotein F, is	14	100.8	98.1	101.6	100.6	99.45	101.1	1.016591252	0.535524
Q6UW68	TMEM205	Transmembrane protein 205 OS=Homo sapier	3	98	95.2	97.9	98.5	96.6	98.2	1.016563147	0.53585
P23396	RPS3	40S ribosomal protein S3 OS=Homo sapiens (	20	98.1	101.3	101.7	101	99.7	101.35	1.016549649	0.536006
A0A024R566	SF1	Splicing factor 1, isoform CRA_a OS=Homo	13	98.1	101.4	101.6	101.2	99.75	101.4	1.016541353	0.536103
Q69YH5	CDCA2	Cell division cycle-associated protein 2 OS=H	8	101.3	98.3	101.4	101.5	99.8	101.45	1.016533066	0.5362
Q8WU90	ZC3H15	Zinc finger CCCH domain-containing protein	13	99.9	100.5	101.8	101.9	100.2	101.85	1.016467066	0.536964
O60333	KIF1B	Kinesin-like protein KIF1B OS=Homo sapien	2	111.3	90.3	88.9	116	100.8	102.45	1.016369048	0.5381
B0YJ89	RIOK3	RIO kinase 3 (Yeast), isoform CRA_a OS=Ho	1	102.8	98.8	90	114.9	100.8	102.45	1.016369048	0.5381
A0A0A2R2W3	PRKAR2A	Protein kinase cAMP-dependent regulatory ty	14	103	98.8	103.5	101.6	100.9	102.55	1.016352825	0.53829
Q9H5Q4	TFB2M	Dimethyladenosine transferase 2, mitochondri	9	98.3	97.7	100.5	98.7	98	99.6	1.016326531	0.538594
P17844	DDX5	Probable ATP-dependent RNA helicase DDX	30	98.6	99	101.7	99.1	98.8	100.4	1.016194332	0.540131
O15055	PER2	Period circadian protein homolog 2 OS=Hom	1	29.6	26	31.4	25.1	27.8	28.25	1.01618705	0.540216
P09543	CNP	2',3'-cyclic-nucleotide 3'-phosphodiesterase O	16	100.5	97.6	101.4	99.9	99.05	100.65	1.016153458	0.540607
O14548	COX7A2L	Cytochrome c oxidase subunit 7A-related prot	2	100.9	98.1	102.1	100.1	99.5	101.1	1.016080402	0.541457
A0A0B4J1V8	PPAN-P2RY11	HCG2039996 OS=Homo sapiens GN=PPAN-	14	100.5	98.6	104.7	97.6	99.55	101.15	1.016072325	0.541552
Q17RE6	TXNRD2	TXNRD2 protein OS=Homo sapiens GN=TX	4	102.9	96.3	99.8	102.6	99.6	101.2	1.016064257	0.541645
J3KNL6	SEC16A	Protein transport protein Sec16A OS=Homo s	3	100.3	99.3	101.6	101.2	99.8	101.4	1.016032064	0.54202
Q9UPN4	CEP131	Centrosomal protein of 131 kDa OS=Homo sa	1	93.2	107.5	104.3	99.6	100.35	101.95	1.015944195	0.543044
P51398	DAP3	28S ribosomal protein S29, mitochondrial OS	18	98.2	96.3	98.8	98.8	97.25	98.8	1.015938303	0.543114
H7BXF5	SAP130	Histone deacetylase complex subunit SAP130	2	96	98.8	96.8	101.1	97.4	98.95	1.015913758	0.5434
Q641Q2	FAM21A	WASH complex subunit FAM21A OS=Homo	3	98	96.9	96.2	101.8	97.45	99	1.015905593	0.543495
Q14344	GNA13	Guanine nucleotide-binding protein subunit al	9	101.9	99.4	101.2	103.3	100.65	102.25	1.015896672	0.543599
P14625	HSP90B1	Endoplasmin OS=Homo sapiens GN=HSP90I	42	101.1	100.5	102.1	102.7	100.8	102.4	1.015873016	0.543875
Q9BW27	NUP85	Nuclear pore complex protein Nup85 OS=Ho	13	100.5	94.9	99.5	99	97.7	99.25	1.015864893	0.543971
P30049	ATP5D	ATP synthase subunit delta, mitochondrial OS	2	100.5	95.3	98.2	100.7	97.9	99.45	1.015832482	0.54435
B4DW52		cDNA FLJ55253, highly similar to Actin, cyto	3	100.3	95.6	99.2	99.8	97.95	99.5	1.0158244	0.544443
Q8WV92	MITD1	MIT domain-containing protein 1 OS=Homo	2	96.8	99.7	103.2	96.4	98.25	99.8	1.015776081	0.545009
A0A0S2Z5C3	PAK1IP1	PAK1 interacting protein 1 isoform 1 (Fragme	12	100.5	96.5	101.1	99	98.5	100.05	1.015736041	0.545477
Q92783	STAM	Signal transducing adapter molecule 1 OS=H	6	98.9	98.3	98.8	101.5	98.6	100.15	1.015720081	0.545662
Q6DD88	ATL3	Atlastin-3 OS=Homo sapiens GN=ATL3 PE=	16	98.8	98.7	99.5	101.1	98.75	100.3	1.015696203	0.545943
Q6IQ22	RAB12	Ras-related protein Rab-12 OS=Homo sapiens	1	95.4	102.2	104.3	96.4	98.8	100.35	1.015688259	0.546035
Q8NCH0	CHST14	Carbohydrate sulfotransferase 14 OS=Homo s	2	99	98.7	106.2	94.6	98.85	100.4	1.015680324	0.546128
B1AK66	SNIP1	Smad nuclear interacting protein 1 OS=Homo	3	96.1	95.5	95.9	98.7	95.8	97.3	1.01565762	0.546393
Q8WUM0	NUP133	Nuclear pore complex protein Nup133 OS=H	29	100.9	97.6	100.8	100.8	99.25	100.8	1.015617128	0.546868
Q13425	SNTB2	Beta-2-syntrophin OS=Homo sapiens GN=SN	11	100.9	97.9	100.9	101	99.4	100.95	1.015593561	0.547144
P04843	RPN1	Dolichyl-diphosphooligosaccharide--protein g	30	100.6	98.4	101.5	100.6	99.5	101.05	1.015577889	0.547327
O43175	PHGDH	D-3-phosphoglycerate dehydrogenase OS=Hc	17	100.2	98.8	101	101.1	99.5	101.05	1.015577889	0.547327
Q86U44	METTL3	N6-adenosine-methyltransferase 70 kDa subu	4	100.8	98.4	95.2	107.1	99.6	101.15	1.015562249	0.54751
Q5T200	ZC3H13	Zinc finger CCCH domain-containing protein	5	98.9	94.3	97	99.2	96.6	98.1	1.01552795	0.547912
A0A0A0MTH	BTAF1	TATA-binding protein-associated factor 172 (	5	96	97.2	94.5	101.7	96.6	98.1	1.01552795	0.547912
Q5JPC1	DKFZp667O16	Putative uncharacterized protein DKFZp667C	3	101.8	98.2	103.8	99.3	100	101.55	1.0155	0.548241
Q8WWM7	ATXN2L	Ataxin-2-like protein OS=Homo sapiens GN=	17	100	100.7	101.5	102.3	100.35	101.9	1.015445939	0.548874
Q69YL0	NCBP2-AS2	Uncharacterized protein NCBP2-AS2 OS=Ho	2	98.8	102	101	102.9	100.4	101.95	1.015438247	0.548965
A0A024RDE8	PDLIM5	PDZ and LIM domain 5, isoform CRA_c OS=	10	100.6	101.1	104.3	100.5	100.85	102.4	1.01536936	0.549773
P51946	CCNH	Cyclin-H OS=Homo sapiens GN=CCNH PE=	3	104.8	97.6	104.6	100.9	101.2	102.75	1.015316206	0.550397
A0A0U1RRM	ENAH	Protein enabled homolog OS=Homo sapiens (	10	98.5	104	102.9	102.7	101.25	102.8	1.015308642	0.550487
J3QK89	CHERP	Calcium homeostasis endoplasmic reticulum p	19	98.2	99.3	99.6	100.9	98.75	100.25	1.015189873	0.551883
Q8NFC6	BOD1L1	Biorientation of chromosomes in cell division	2	101.6	96.4	95.7	105.3	99	100.5	1.015151515	0.552335
P22695	UQCRC2	Cytochrome b-c1 complex subunit 2, mitocho	20	99.2	98.9	101.1	100	99.05	100.55	1.015143867	0.552425
B1Q2B0	URCC5	URCC5 OS=Homo sapiens GN=URCC5 PE=	9	98	100.6	104	97.6	99.3	100.8	1.01510574	0.552874
A0A024RAC6	TCEB3	Transcription elongation factor B (SIII), polyp	3	105.8	93	100.4	101.4	99.4	100.9	1.015090543	0.553052
Q9Y295	DRG1	Developmentally-regulated GTP-binding prot	18	99	99.9	100.8	101.1	99.45	100.95	1.015082956	0.553142
Q9UEI6		Polio virus related protein 2, alpha isoform (F	7	99.8	99.4	100.9	101.3	99.6	101.1	1.015060241	0.553411
Q8TCJ2	STT3B	Dolichyl-diphosphooligosaccharide--protein g	10	99.4	99.8	103	99.2	99.6	101.1	1.015060241	0.553411
A0A024QZI5	NCOA4	Nuclear receptor coactivator 4, isoform CRA_	1	108.2	98.1	111.1	98.3	103.15	104.7	1.01502666	0.553805
Q7L0Y3	TRMT10C	Mitochondrial ribonuclease P protein 1 OS=H	17	100.9	98.8	100.9	101.8	99.85	101.35	1.015022534	0.553855
V9HW04	HEL-S-80p	Serine/threonine-protein phosphatase OS=Ho	7	99.3	101.1	101.2	102.2	100.2	101.7	1.01497006	0.554473
B4E1H4		cDNA FLJ54226, highly similar to Transmem	2	97.1	103.8	97.8	106.1	100.45	101.95	1.014932802	0.554913
P62249	RPS16	40S ribosomal protein S16 OS=Homo sapiens	13	97.3	104.2	101.8	102.7	100.75	102.25	1.014888337	0.555438
A8KA74		cDNA FLJ76065 OS=Homo sapiens PE=2 SV	20	98.7	96.9	100.4	98.1	97.8	99.25	1.014826176	0.556172
Q9P0K7	RAI14	Ankycorbin OS=Homo sapiens GN=RAI14 P	7	101.7	100.7	101.4	104	101.2	102.7	1.014822134	0.55622
Q8WTS6	SETD7	Histone-lysine N-methyltransferase SETD7 O	2	101.9	94.3	99.1	100	98.1	99.55	1.014780836	0.556708
Q9C0J8	WDR33	pre-mRNA 3' end processing protein WDR33	10	98.6	97.8	99.7	99.6	98.2	99.65	1.014765784	0.556887
O43819	SCO2	Protein SCO2 homolog, mitochondrial OS=H	5	98.7	97.7	100.6	98.7	98.2	99.65	1.014765784	0.556887
Q8NEJ9	NGDN	Neuroguidin OS=Homo sapiens GN=NGDN	7	98.2	98.2	96.5	102.8	98.2	99.65	1.014765784	0.556887
Q9H4L4	SENP3	Sentrin-specific protease 3 OS=Homo sapiens	6	102.5	93.9	94.8	104.5	98.2	99.65	1.014765784	0.556887
Q15286	RAB35	Ras-related protein Rab-35 OS=Homo sapiens	6	103.6	99.7	102.7	103.6	101.65	103.15	1.014756517	0.556995
O60508	CDC40	Pre-mRNA-processing factor 17 OS=Homo sa	13	98.7	98.1	97.2	102.5	98.4	99.85	1.014735772	0.55724
Q96E29	MTERF3	Transcription termination factor 3, mitochond	10	99.1	97.8	98.8	101	98.45	99.9	1.014728288	0.557329
Q8NHP6	MOSPD2	Motile sperm domain-containing protein 2 OS	3	99.3	91.6	94.7	99	95.45	96.85	1.014667365	0.55805
Q96N66	MBOAT7	Lysophospholipid acyltransferase 7 OS=Hom	7	101.9	95.9	101.6	99.1	98.9	100.35	1.014661274	0.558122

J3KQ48	PTRH2	Peptidyl-tRNA hydrolase 2, mitochondrial OS	6	101.4	96.9	103.5	97.7	99.15	100.6	1.014624307	0.55856
P35610	SOAT1	Sterol O-acyltransferase 1 OS=Homo sapiens	7	101.2	97.1	101.5	99.7	99.15	100.6	1.014624307	0.55856
Q9NR45	NANS	Sialic acid synthase OS=Homo sapiens GN=N	6	97.7	100.7	98.8	102.5	99.2	100.65	1.014616935	0.558647
H0Y5K5	ERGIC3	Endoplasmic reticulum-Golgi intermediate co	6	100.9	97.6	101.4	100	99.25	100.7	1.014609572	0.558735
Q5RKV6	EXOSC6	Exosome complex component MTR3 OS=Ho	11	97.4	101.1	100.6	100.8	99.25	100.7	1.014609572	0.558735
Q8N684	CPSF7	Cleavage and polyadenylation specificity fact	13	99.4	99.3	100.2	101.4	99.35	100.8	1.014594867	0.558909
Q86TW2	ADCK1	Uncharacterized aarF domain-containing prot	1	100.3	105.3	101	107.6	102.8	104.3	1.01459144	0.558949
Q99623	PHB2	Prohibitin-2 OS=Homo sapiens GN=PHB2 PE	22	98.1	100.8	102.3	99.5	99.45	100.9	1.014580191	0.559082
D3DND1	CDV3	CDV3 homolog (Mouse), isoform CRA_a OS	6	98.1	101.1	98.9	103.2	99.6	101.05	1.014558233	0.559344
Q13889	GTF2H3	General transcription factor IIH subunit 3 OS	2	102	97.5	103.7	98.7	99.75	101.2	1.014536341	0.559602
Q99549	MPHOSPH8	M-phase phosphoprotein 8 OS=Homo sapiens	7	99.4	100.1	105.1	97.3	99.75	101.2	1.014536341	0.559602
Q9BVX2	TMEM106C	Transmembrane protein 106C OS=Homo sapi	1	102.1	90.9	95.3	100.5	96.5	97.9	1.014507772	0.559941
P61165	TMEM258	Transmembrane protein 258 OS=Homo sapie	1	97	102.9	103.7	99.1	99.95	101.4	1.014507254	0.559947
Q5VUA4	ZNF318	Zinc finger protein 318 OS=Homo sapiens GN	4	103.8	96.9	105.5	98.1	100.35	101.8	1.014449427	0.560633
A0A023T787	RBM8	RNA-binding protein 8A OS=Homo sapiens G	6	95.2	98.7	96.6	100.1	96.95	98.35	1.014440433	0.560741
Q7L014	DDX46	Probable ATP-dependent RNA helicase DDX	41	100.5	100.4	103	100.8	100.45	101.9	1.014435042	0.560804
Q86UP2	KTN1	Kinectin OS=Homo sapiens GN=KTN1 PE=1	69	100	101	102	101.9	100.5	101.95	1.014427861	0.560889
A0A140VJE8	AP2B1	AP complex subunit beta OS=Homo sapiens G	20	99.8	101.5	103.4	100.8	100.65	102.1	1.014406359	0.561145
Q6PJ53		Uncharacterized protein (Fragment) OS=Hom	1	105.5	82.1	89.5	100.8	93.8	95.15	1.014392324	0.561311
E7ESZ7	NDUFA10	NADH dehydrogenase [ubiquinone] 1 alpha s	6	104.3	97.2	104.8	99.6	100.75	102.2	1.01439206	0.561315
E9PI68	SPCS2	Signal peptidase complex subunit 2 OS=Hom	9	102.6	99	104.9	99.6	100.8	102.25	1.014384921	0.5614
A0A0B4J1S4	SEP15	15 kDa selenoprotein OS=Homo sapiens GN=	2	102	99.7	103.1	101.5	100.85	102.3	1.014377789	0.561484
P26885	FKBP2	Peptidyl-prolyl cis-trans isomerase FKBP2 OS	8	102.4	99.7	105.7	99.3	101.05	102.5	1.014349332	0.561822
U3KQ64	FKBP8	Peptidyl-prolyl cis-trans isomerase FKBP8 (F	1	98.1	97.7	89.3	109.3	97.9	99.3	1.014300306	0.562404
Q6UXH1	CRELD2	Cysteine-rich with EGF-like domain protein 2	4	103.8	99.4	106.4	99.7	101.6	103.05	1.014271654	0.562746
P15291	B4GALT1	Beta-1,4-galactosyltransferase 1 OS=Homo sa	1	102.4	100.9	102.4	103.8	101.65	103.1	1.014264634	0.562829
A0A024R438	ATG9A	ATG9 autophagy related 9 homolog A (S. cer	8	97.8	98.6	98.5	100.7	98.2	99.6	1.014256619	0.562924
Q9UNR6	ERG1	Squalene epoxidase OS=Homo sapiens GN=E	3	106.2	97.8	99.9	107	102	103.45	1.014215686	0.563411
Q5C9Z4	NOM1	Nucleolar MIF4G domain-containing protein	14	98.5	98.6	100.4	99.5	98.55	99.95	1.014205987	0.563527
A0A024R4U3	TTLL12	Tubulin tyrosine ligase-like family, member 1	13	99.8	97.4	94.9	105.1	98.6	100	1.014198783	0.563612
Q9ULI0	ATAD2B	ATPase family AAA domain-containing prote	1	98.5	98.7	96.5	103.5	98.6	100	1.014198783	0.563612
Q9NVF7	FBXO28	F-box only protein 28 OS=Homo sapiens GN	1	98.7	98.6	101.5	98.6	98.65	100.05	1.014191586	0.563697
Q9UNF0	PACSIN2	Protein kinase C and casein kinase substrate i	4	101	96.5	99.3	101	98.75	100.15	1.014177215	0.563869
Q8TA92		Similar to AFG3 ATPase family gene 3-like 2	19	100	97.6	101.6	98.8	98.8	100.2	1.01417004	0.563954
Q9NVR2	INTS10	Integrator complex subunit 10 OS=Homo sap	8	99.6	98.1	99.9	100.6	98.85	100.25	1.014162873	0.564039
Q9H6R4	NOL6	Nucleolar protein 6 OS=Homo sapiens GN=N	16	97.1	100.7	102.9	97.7	98.9	100.3	1.014155713	0.564125
Q9UMS4	PRPF19	Pre-mRNA-processing factor 19 OS=Homo sa	19	100.5	98.8	100.6	101.5	99.65	101.05	1.014049172	0.565394
B2RBL3		Thymidine phosphorylase OS=Homo sapiens	1	102.7	90	101.5	93.9	96.35	97.7	1.014011417	0.565845
A0A024R321	FLNB	Filamin B, beta (Actin binding protein 278), i	41	101.1	99.1	100.9	102.1	100.1	101.5	1.013986014	0.566148
E5KLJ5	OPA1	Dynamin-like 120 kDa protein, mitochondrial	35	101.1	99.2	102.4	100.7	100.15	101.55	1.013979031	0.56623
R4GMX3	COMMD3-BM	Protein COMMD3-BMI1 OS=Homo sapiens	3	94.8	105.6	101.8	101.4	100.2	101.6	1.013972056	0.566314
Q92542	NCSTN	Nicastrin OS=Homo sapiens GN=NCSTN PE	8	102.4	98	100.9	102.3	100.2	101.6	1.013972056	0.566314
Q12986	NFX1	Transcriptional repressor NF-X1 OS=Homo s	1	96.5	96.8	97	99	96.65	98	1.013967926	0.566364
P22681	CBL	E3 ubiquitin-protein ligase CBL OS=Homo sa	3	97.2	103.6	100.9	102.7	100.4	101.8	1.013944223	0.566645
Q9NY93	DDX56	Probable ATP-dependent RNA helicase DDX	16	96.6	97.2	99.1	97.4	96.9	98.25	1.013931889	0.566793
O95817	BAG3	BAG family molecular chaperone regulator 3	5	102.1	100	103.1	101.8	101.05	102.45	1.013854527	0.567717
A0A087WY31	YTHDF3	YTH domain-containing family protein 3 OS	5	93.1	95.1	91.7	99.1	94.1	95.4	1.01381509	0.568189
Q549M8	C14orf166	CLE7 OS=Homo sapiens GN=C14orf166 PE	11	102.6	100.8	103.8	102.4	101.7	103.1	1.013765978	0.568776
Q9GZQ3	COMMD5	COMM domain-containing protein 5 OS=Ho	1	112.5	98.9	107.2	107.1	105.7	107.15	1.01371807	0.569348
O60566	BUB1B	Mitotic checkpoint serine/threonine-protein k	4	100.9	96	98.5	101.1	98.45	99.8	1.013712544	0.569415
A0A0S2Z5D8	WDR41	WD repeat domain 41 isoform 2 (Fragment)	1	102.3	95	100.4	99.6	98.65	100	1.013684744	0.569747
Q6NUQ4	TMEM214	Transmembrane protein 214 OS=Homo sapie	10	98.3	99.4	100.1	100.3	98.85	100.2	1.013657056	0.570078
Q8WW59	SPRYD4	SPRY domain-containing protein 4 OS=Hom	7	99.9	97.9	100.3	100.2	98.9	100.25	1.013650152	0.570161
P46060	RANGAP1	Ran GTPase-activating protein 1 OS=Homo s	27	100	98.3	102.1	98.9	99.15	100.5	1.013615734	0.570574
Q9H2G2	SLK	STE20-like serine/threonine-protein kinase O	7	102.9	95.5	100.8	100.3	99.2	100.55	1.013608871	0.570656
Q9Y3C1	NOP16	Nucleolar protein 16 OS=Homo sapiens GN=	7	99.1	99.4	100.7	100.5	99.25	100.6	1.013602015	0.570738
P55809	OXCT1	Succinyl-CoA:3-ketoacid coenzyme A transfe	20	100.3	98.9	100.8	101.1	99.6	100.95	1.013554217	0.571311
J3QL71	SCRN2	Secernin-2 OS=Homo sapiens GN=SCRN2 P	1	109.9	89.6	91.8	110.4	99.75	101.1	1.013533835	0.571555
Q8IZ52	CHPF	Chondroitin sulfate synthase 2 OS=Homo sap	1	120.2	80	103.2	99.7	100.1	101.45	1.013486513	0.572122
Q96A33	CCDC47	Coiled-coil domain-containing protein 47 OS	18	102.2	98.3	102.1	101.1	100.25	101.6	1.013466334	0.572364
Q9HC07	TMEM165	Transmembrane protein 165 OS=Homo sapie	5	104.1	97.5	101.9	102.4	100.8	102.15	1.01339285	0.573247
Q8TCC3	MRPL30	39S ribosomal protein L30, mitochondrial OS	3	99.9	101.7	103.6	100.7	100.8	102.15	1.01339285	0.573247
Q9Y6X4	FAM169A	Soluble lamin-associated protein of 75 kDa O	4	99.6	95	99.2	98	97.3	98.6	1.01336074	0.573632
K7ELG9	LSM12	Protein LSM12 homolog OS=Homo sapiens G	4	102.3	100	102.6	102.4	101.15	102.5	1.013346515	0.573802
Q53R41	FASTKD1	FAST kinase domain-containing protein 1, mi	2	101.8	102.2	110.2	96.5	102	103.35	1.013235294	0.575139
Q9H173	SIL1	Nucleotide exchange factor SIL1 OS=Homo s	4	104.2	100	101	105.9	102.1	103.45	1.013222331	0.575296
A0A024R718	PBEF1	Pre-B-cell colony enhancing factor 1, isoform	11	99	98.3	99.8	100.1	98.65	99.95	1.013177902	0.575831
E7EPT4	NDUFV2	NADH dehydrogenase [ubiquinone] flavoprot	9	101.3	96.3	100	100.2	98.8	100.1	1.013157895	0.576072
O14975	SLC27A2	Very long-chain acyl-CoA synthetase OS=Ho	9	99	99	99.5	101.1	99	100.3	1.013131313	0.576392
A0A024QZ30	SDHA	Succinate dehydrogenase [ubiquinone] flavop	21	100.9	97.6	99.3	101.8	99.25	100.55	1.013098237	0.576789
Q6UN15	FIP1L1	Pre-mRNA 3'-end-processing factor FIP1 OS	9	101.3	97.4	100.1	101.2	99.35	100.65	1.013085053	0.576949
Q9HD20	ATP13A1	Manganese-transporting ATPase 13A1 OS=H	27	99.9	99	101	100.5	99.45	100.75	1.013071895	0.577107
A0A0U1RRM	PTBP1	Polypyrimidine tract-binding protein 1 OS=H	19	98.1	101.2	101	100.9	99.65	100.95	1.01304566	0.577423
Q96EL3	MRPL53	39S ribosomal protein L53, mitochondrial OS	4	99.2	100.5	100.9	101.4	99.85	101.15	1.013019529	0.577738
Q6U8A4		Ubiquitin-specific protease 7 isoform (Fragme	23	100	99.8	100.5	101.9	99.9	101.2	1.013013013	0.577817
A0A087X256	KIAA1033	WASH complex subunit SWIP OS=Homo sap	4	101.4	99.4	103.2	100.2	100.4	101.7	1.012948207	0.578599
Q7L523	RRAGA	Ras-related GTP-binding protein A OS=Hom	5	99.6	101.9	102.5	101.6	100.75	102.05	1.012903226	0.579141
Q96TC7	RMDN3	Regulator of microtubule dynamics protein 3	8	101	100.9	104.1	100.4	100.95	102.25	1.012877662	0.579449
E9PRZ1	SAAL1	Protein SAAL1 OS=Homo sapiens GN=SAA	1	91.5	111.1	113.1	92.1	101.3	102.6	1.012833169	0.579988
A0A0C4DG91	NME6	Nucleoside diphosphate kinase OS=Homo sap	2	101	102.1	99.1	106.6	101.55	102.85	1.012801576	0.580369
V9HW26	HEL-S-123m	ATP synthase subunit alpha OS=Homo sapie	34	98.4	97	98.7	99.2	97.7	98.95	1.012794268	0.580457
B1AKZ4	PEA15	Phosphoprotein enriched in astrocytes 15, iso	2	97.8	106.5	104.1	102.8	102.15	103.45	1.012726383	0.581277
Q13148	TARDBP	TAR DNA-binding protein 43 OS=Homo sap	9	99.3	98.6	100.6	99.8	98.95	100.2	1.012632643	0.582412
Q15459	SF3A1	Splicing factor 3A subunit 1 OS=Homo sapie	27	99.2	99.1	100.6	100.2	99.15	100.4	1.012607161	0.58272
Q53F19	NCBP3	Nuclear cap-binding protein subunit 3 OS=H	7	99.9	98.5	101.6	99.3	99.2	100.45	1.012600806	0.582798
Q05048	CSTF1	Cleavage stimulation factor subunit 1 OS=Ho	8	98	100.7	100.4	100.8	99.35	100.6	1.012581782	0.583027
A8K883		cDNA FLJ77590, highly similar to Homo sap	3	95.8	103.3	93.5	108.1	99.55	100.8	1.012556504	0.583333
Q92552	MRPS27	28S ribosomal protein S27, mitochondrial OS	14	99.3	99.9	102.5	99.2	99.6	100.85	1.012550201	0.58341
Q14240	EIF4A2	Eukaryotic initiation factor 4A-II OS=Homo s	4	98.2	101	100.3	101.4	99.6	100.85	1.012550201	0.58341
Q13435	SF3B2	Splicing factor 3B subunit 2 OS=Homo sapie	36	99.1	101	102.2	100.4	100.05	101.3	1.012493753	0.584095
Q969N2	PIGT	GPI transamidase component PIG-T OS=Hon	8	100.4	99.9	103.4	99.4	100.15	101.4	1.012481278	0.584245
O14893	GEMIN2	Gem-associated protein 2 OS=Homo sapiens	1	101.1	99.2	99.7	103.1	100.15	101.4	1.012481278	0.584245
Q9Y3L5	RAP2C	Ras-related protein Rap-2c OS=Homo sapien	4	102.7	97.7	102.3	100.6	100.2	101.45	1.01247505	0.584322
Q9UP83	COG5	Conserved oligomeric Golgi complex subunit	2	87.9	104.5	92.9	101.9	96.2	97.4	1.012474012	0.584333
Q14197	MRPL58	Peptidyl-tRNA hydrolase ICT1, mitochondria	8	95.3	97.6	96.4	98.9	96.45	97.65	1.01244168	0.584727
Q9UNK0	STX8	Syntaxin-8 OS=Homo sapiens GN=STX8 PE	7	100.5	100.6	100	103.6	100.55	101.8	1.012431626	0.584848
A8K3Q9		cDNA FLJ76611, highly similar to Homo sap	1	97.2	96	98	97.6	96.6	97.8	1.01242236	0.584961
P05997	COL5A2	Collagen alpha-2(V) chain OS=Homo sapiens	9	101	101.4	102.6	102.3	101.2	102.45	1.012351779	0.585817
A0A059R2S3	COX1	Cytochrome c oxidase subunit 1 OS=Homo sa	1	96.5	97.9	103.9	92.9	97.2	98.4	1.012345679	0.585891
Q9Y3I0	RTCB	tRNA-splicing ligase RtcB homolog OS=Hom	22	100.3	102.4	103.4	101.8	101.35	102.6	1.012333498	0.586039
Q9HBI2		Putative uncharacterized protein (Fragment)	2	99.4	104	98	107.9	101.7	102.95	1.012291052	0.586554
B2RDE8		cDNA, FLJ96580, highly similar to Homo sap	9	98.1	97.9	99.7	98.7	98	99.2	1.012244898	0.587115
B2RBH6		cDNA, FLJ95513, highly similar to Homo sap	4	107.2	97.8	103.5	104	102.5	103.75	1.012195122	0.587721
Q5VT52	RPRD2	Regulation of nuclear pre-mRNA domain-con	20	99	98.2	98.4	101.2	98.6	99.8	1.012170385	0.588021
A0A0B4J2E5	LOC10272415	Protein LOC102724159 OS=Homo sapiens G	1	89.8	107.8	97.5	102.5	98.8	100	1.012145749	0.588321
Q13564	NAE1	NEDD8-activating enzyme E1 regulatory sub	7	98.5	99.1	98	102	98.8	100	1.012145749	0.588321
Q9Y3A4	RRP7A	Ribosomal RNA-processing protein 7 homolo	9	100.4	97.6	100.9	99.5	99	100.2	1.012121212	0.58862
B3KUB5		cDNA FLJ39527 fis, clone PUAEN2003116,	8	100.9	99	100.8	101.5	99.95	101.15	1.012006003	0.590023
A0A024R1D6	hCG_2011153	HCG2011153, isoform CRA_b OS=Homo sap	13	101.5	98.8	100.5	102.2	100.15	101.35	1.011982027	0.590315
P62487	POLR2G	DNA-directed RNA polymerase II subunit RF	5	99	101.3	100.5	102.2	100.15	101.35	1.011982027	0.590315
P51970	NDUFA8	NADH dehydrogenase [ubiquinone] 1 alpha s	7	98.9	101.7	99.8	103.2	100.3	101.5	1.011964108	0.590534
B4DPP6		cDNA FLJ54371, highly similar to Serum alb	5	101.6	90.7	96.9	97.7	96.15	97.3	1.011960478	0.590578
P82663	MRPS25	28S ribosomal protein S25, mitochondrial OS	6	96.4	105.2	99.7	104.3	100.8	102	1.011904762	0.591258
Q92665	MRPS31	28S ribosomal protein S31, mitochondrial OS	9	102.4	100.2	102.9	102.1	101.3	102.5	1.011846002	0.591976
P24311	COX7B	Cytochrome c oxidase subunit 7B, mitochond	1	90.4	87.8	91.3	89	89.1	90.15	1.011784512	0.592725
A0A024R687	PLEKHC1	Pleckstrin homology domain containing, fami	14	102.6	101.2	104.3	101.9	101.9	103.1	1.011776251	0.592827
F5GXJ1	NDUFS7	NADH dehydrogenase [ubiquinone] iron-sulf	2	100	95.4	98.2	99.5	97.7	98.85	1.011770727	0.592894
V9HWK4	HEL162	Epididymis luminal protein 162 OS=Homo sa	11	96.6	99.1	98.7	99.3	97.85	99	1.011752683	0.593114
A0A075B746	MRPS21	28S ribosomal protein S21, mitochondrial OS	2	100.1	96	102.2	96.2	98.05	99.2	1.01172871	0.593407
Q9NR31	SAR1A	GTP-binding protein SAR1a OS=Homo sapie	5	97.1	99.6	99.9	99.1	98.35	99.5	1.011692933	0.593846
P18583	SON	Protein SON OS=Homo sapiens GN=SON PE	11	99.4	97.4	99.4	99.7	98.4	99.55	1.011686992	0.593917
Q86TX8		Full-length cDNA clone CS0DC015YK16 of	1	97.9	99.1	91.7	107.6	98.5	99.65	1.011675127	0.594063

Q9H7D7	WDR26	WD repeat-containing protein 26 OS=Homo s	8	99.8	98.5	97.9	102.7	99.15	100.3	1.011598588	0.594999
Q9GZN1	ACTR6	Actin-related protein 6 OS=Homo sapiens GN	4	102	96.3	96.8	103.8	99.15	100.3	1.011598588	0.594999
Q12996	CSTF3	Cleavage stimulation factor subunit 3 OS=Ho	16	98.8	99.5	100.6	100	99.15	100.3	1.011598588	0.594999
Q9P015	MRPL15	39S ribosomal protein L15, mitochondrial OS	12	100	98.9	98.8	102.4	99.45	100.6	1.0115636	0.595427
Q6PGP7	TTC37	Tetratricopeptide repeat protein 37 OS=Homo	4	99.8	99.8	100.4	101.5	99.8	100.95	1.011523046	0.595924
Q9BVI4	NOC4L	Nucleolar complex protein 4 homolog OS=Ho	13	101.7	97.9	101.3	100.6	99.8	100.95	1.011523046	0.595924
Q15061	WDR43	WD repeat-containing protein 43 OS=Homo s	15	98.9	100.7	102.9	99	99.8	100.95	1.011523046	0.595924
P62699	YPEL5	Protein yippee-like 5 OS=Homo sapiens GN=	1	106.2	93.7	106.3	95.9	99.95	101.1	1.011505753	0.596136
Q7Z3K3	POGZ	Pogo transposable element with ZNF domain	17	101	99.1	97.5	104.9	100.05	101.2	1.011494253	0.596276
O75822	EIF3J	Eukaryotic translation initiation factor 3 subu	12	99.1	102.2	99.7	103.9	100.65	101.8	1.011425733	0.597116
A0A024R3H6	PHLDB1	Pleckstrin homology-like domain, family B, n	3	96.8	105.1	96.9	107.3	100.95	102.1	1.011391778	0.597533
P53634	CTSC	Dipeptidyl peptidase 1 OS=Homo sapiens GN	9	101.3	101	103.4	101.2	101.15	102.3	1.011369254	0.597809
Q9Y6C9	MTCH2	Mitochondrial carrier homolog 2 OS=Homo s	9	101.7	100.8	105.3	99.5	101.25	102.4	1.011358025	0.597947
G3V1L9	TJP1	Tight junction protein 1 (Zona occludens 1), i	23	103.9	98.7	101.2	103.7	101.3	102.45	1.011352419	0.598016
Q12888	TP53BP1	Tumor suppressor p53-binding protein 1 OS=	28	97.9	96.8	98.6	98.3	97.35	98.45	1.011299435	0.598665
B5BU77	PRKCH	Protein kinase C eta type OS=Homo sapiens (	1	103.7	100.1	98.8	107.3	101.9	103.05	1.011285574	0.598836
Q9BUM1	G6PC3	Glucose-6-phosphatase 3 OS=Homo sapiens (	1	107.2	88.5	90.3	107.6	97.85	98.95	1.011241696	0.599375
P19784	CSNK2A2	Casein kinase II subunit alpha' OS=Homo sap	13	98.1	98.8	99.5	99.6	98.45	99.55	1.011173184	0.600217
V9HW31	HEL-S-271	ATP synthase subunit beta OS=Homo sapiens	18	98.5	98.6	98.7	100.6	98.55	99.65	1.011161847	0.600357
G9FP35	GNAQ	Guanine nucleotide binding protein OS=Hom	4	101.3	97.1	99	101.6	99.2	100.3	1.01108871	0.601255
Q9P2N7	KLHL13	Kelch-like protein 13 OS=Homo sapiens GN=	1	108	91.1	94.8	106.5	99.55	100.65	1.011049724	0.601735
Q5ZEY3	GAPD	Glyceraldehyde-3-phosphate dehydrogenase (	1	95.5	103.9	99.5	102.1	99.7	100.8	1.011033099	0.601941
A8K7Z3		cDNA FLJ77229, highly similar to Homo sap	1	100.9	98.9	101.8	100.2	99.9	101	1.011011011	0.602212
P49750	YLPM1	YLP motif-containing protein 1 OS=Homo sa	10	99.9	100.2	103.9	98.4	100.05	101.15	1.010994503	0.602415
A0A024R2T6	DHX30	DEAH (Asp-Glu-Ala-His) box polypeptide 30	39	100.4	99.8	102.5	99.9	100.1	101.2	1.010989011	0.602482
A0A024RAD5	DDOST	Dolichyl-diphosphooligosaccharide--protein g	14	101.3	99.4	101.7	101.2	100.35	101.45	1.010961634	0.60282
P61026	RAB10	Ras-related protein Rab-10 OS=Homo sapiens	11	103.4	97.9	103.9	99.6	100.65	101.75	1.010928962	0.603222
Q969Z3	MARC2	Mitochondrial amidoxime reducing componen	2	100.5	100.9	103.5	100.1	100.7	101.8	1.010923535	0.60329
P09874	PARP1	Poly [ADP-ribose] polymerase 1 OS=Homo s	46	100.6	102.1	103.2	101.7	101.35	102.45	1.010853478	0.604153
Q15075	EEA1	Early endosome antigen 1 OS=Homo sapiens	57	103.1	100.9	102.7	103.5	102	103.1	1.010784314	0.605007
Q9H1A3	METTL9	Methyltransferase-like protein 9 OS=Homo sa	1	106.4	97.7	105.1	101.2	102.05	103.15	1.01077903	0.605072
Q07021	C1QBP	Complement component 1 Q subcomponent-b	9	101.3	94.7	98.3	99.8	98	99.05	1.010714286	0.605871
P21281	ATP6V1B2	V-type proton ATPase subunit B, brain isofor	18	99.2	98	99.3	100	98.6	99.65	1.010649087	0.606677
Q14320	FAM50A	Protein FAM50A OS=Homo sapiens GN=FA	8	97.5	100.3	100.9	99	98.9	99.95	1.010616785	0.607076
Q99496	RNF2	E3 ubiquitin-protein ligase RING2 OS=Homo	7	101.8	96.5	98.2	102.2	99.15	100.2	1.010590015	0.607407
O75530	EED	Polycomb protein EED OS=Homo sapiens GN	11	99.6	100	103.9	97.8	99.8	100.85	1.010521042	0.60826
O14657	TOR1B	Torsin-1B OS=Homo sapiens GN=TOR1B PE	5	102	98.5	102.6	100	100.25	101.3	1.010473815	0.608844
A0A087X1Z3	PSME2	Proteasome activator complex subunit 2 OS=I	9	95.5	96.4	94.4	99.5	95.95	96.95	1.010422095	0.609485
A0A024R943	TOR3A	Torsin family 3, member A, isoform CRA_b (	8	100.6	101.3	102.7	101.3	100.95	102	1.010401189	0.609745
Q53GR7		Solute carrier family 25, member 13 (Citrin) v	15	102.6	100.1	102.9	101.9	101.35	102.4	1.010360138	0.610253
Q14498	RBM39	RNA-binding protein 39 OS=Homo sapiens C	17	95.5	98	97.7	97.8	96.75	97.75	1.010335917	0.610553
P47813	EIF1AX	Eukaryotic translation initiation factor 1A, X-	6	97.5	105.8	102.8	102.6	101.65	102.7	1.010329562	0.610631
X5D8T4	ALDH5A1	Aldehyde dehydrogenase 5 family member A	10	100.2	95.6	100.2	97.6	97.9	98.9	1.010214505	0.61206
Q96HV5	TMEM41A	Transmembrane protein 41A OS=Homo sapie	1	122.1	93.4	112.1	105.6	107.75	108.85	1.010208817	0.612129
P05204	HMGN2	Non-histone chromosomal protein HMG-17 C	1	97.8	99	97.2	101.6	98.4	99.4	1.010162602	0.612703
Q6GMV3	PTRHD1	Putative peptidyl-tRNA hydrolase PTRHD1 C	5	97.4	99.8	100	99.2	98.6	99.6	1.010141988	0.61296
Q8NDX5	PHC3	Polyhomeotic-like protein 3 OS=Homo sapien	5	99.5	97.7	101.8	97.4	98.6	99.6	1.010141988	0.61296
Q6P6C2	ALKBH5	RNA demethylase ALKBH5 OS=Homo sapie	7	99.2	98.4	99.4	100.2	98.8	99.8	1.010121457	0.613214
Q96RN5	MED15	Mediator of RNA polymerase II transcription	4	97.5	91	95.1	95.3	94.25	95.2	1.010079576	0.613734
O75533	SF3B1	Splicing factor 3B subunit 1 OS=Homo sapien	49	100	98.5	101.3	99.2	99.25	100.25	1.010075567	0.613785
A0A024R094	PAIP1	Poly(A) binding protein interacting protein 1,	6	96.6	102.2	99.1	101.7	99.4	100.4	1.010060362	0.613974
Q53GS9	USP39	U4/U6.U5 tri-snRNP-associated protein 2 OS	14	100	98.9	101.3	99.6	99.45	100.45	1.010055304	0.614037
Q96EL2	MRPS24	28S ribosomal protein S24, mitochondrial OS	4	100.4	99.4	102.9	98.9	99.9	100.9	1.01001001	0.6146
C9JEH3	AAMP	Angio-associated migratory cell protein OS=I	5	97.5	102.5	97.5	104.5	100	101	1.01	0.614724
O15144	ARPC2	Actin-related protein 2/3 complex subunit 2 C	15	99.3	101.2	102.2	100.3	100.25	101.25	1.009975062	0.615034
P05114	HMGN1	Non-histone chromosomal protein HMG-14 C	6	96	104.7	99.9	102.8	100.35	101.35	1.009965122	0.615157
B4E0L0		cDNA FLJ54030, highly similar to Polymeras	16	100.9	100.1	102.4	100.6	100.5	101.5	1.009950249	0.615343
Q9NVH1	DNAJC11	DnaJ homolog subfamily C member 11 OS=H	11	101.7	99.9	103.7	99.9	100.8	101.8	1.009920635	0.615712
A0A0A6YYL(	RPL17-C18orf	Protein RPL17-C18orf32 OS=Homo sapiens (	10	98.3	103.6	102.5	101.4	100.95	101.95	1.009905894	0.615895
P35613	BSG	Basigin OS=Homo sapiens GN=BSG PE=1 S	11	102.5	102.2	104.4	102.3	102.35	103.35	1.009770396	0.617583
E7EWW0	C16orf62	UPF0505 protein C16orf62 OS=Homo sapien	1	104.8	100.1	103.2	103.7	102.45	103.45	1.009760859	0.617702
Q9BYE7	PCGF6	Polycomb group RING finger protein 6 OS=H	1	101.3	104.4	101.4	106.3	102.85	103.85	1.009722897	0.618175
B2RDW0		cDNA, FLJ96792, highly similar to Homo sap	7	97.4	98.3	96	101.6	97.85	98.8	1.009708738	0.618352
B2RDX7		cDNA, FLJ96814, highly similar to Homo sap	1	105.2	101.6	102.3	106.5	103.4	104.4	1.00967118	0.618821
A0A024R837	HDHD3	Haloacid dehalogenase-like hydrolase domain	6	97.4	99.7	100.3	98.7	98.55	99.5	1.009639777	0.619213
G5E9I4	BRMS1	Breast cancer metastasis suppressor 1, isoforn	1	108	89.2	92.7	106.4	98.6	99.55	1.009634888	0.619274
A0A087X0H9	RBM26	RNA-binding protein 26 OS=Homo sapiens C	6	98.9	99.1	99.9	100	99	99.95	1.00959596	0.61976
A8K6J9		cDNA FLJ76879, highly similar to Homo sap	17	98.1	100.2	99.6	100.6	99.15	100.1	1.009581442	0.619941
O75489	NDUFS3	NADH dehydrogenase [ubiquinone] iron-sulf	10	102.1	96.3	100.3	100	99.2	100.15	1.009576613	0.620002
Q6IBN1	HNRPK	HNRPK protein OS=Homo sapiens GN=HNF	1	102.6	96.8	97.6	103.7	99.7	100.65	1.009528586	0.620601
A0A024R648	TIMM9	Translocase of inner mitochondrial membrane	4	102.6	96.9	100.7	100.7	99.75	100.7	1.00952381	0.620661
E9KL48	GLUD1	Epididymis tissue sperm binding protein Li 18	22	98.9	100.7	101.9	99.6	99.8	100.75	1.009519038	0.620722
Q9GZR5	ELOVL4	Elongation of very long chain fatty acids prot	2	95	104.7	98.9	102.7	99.85	100.8	1.009514271	0.620781
Q13405	MRPL49	39S ribosomal protein L49, mitochondrial OS	6	99.5	100.6	102.3	99.7	100.05	101	1.009495252	0.621018
P11274	BCR	Breakpoint cluster region protein OS=Homo s	2	97.5	102.6	100.6	101.4	100.05	101	1.009495252	0.621018
A0A024R653	FBXO34	F-box protein 34, isoform CRA_a OS=Homo	1	98.4	102.1	97.8	104.6	100.25	101.2	1.009476309	0.621255
A0A024R3W7	EEF1B2	Eukaryotic translation elongation factor 1 bet	7	98.6	102.6	99.9	103.2	100.6	101.55	1.00944334	0.621668
O75886	STAM2	Signal transducing adapter molecule 2 OS=H	3	103.7	97.5	101.9	101.2	100.6	101.55	1.00944334	0.621668
Q8N2L6		cDNA FLJ90138 fis, clone HEMBB1000905,	11	104.3	97.2	100.8	102.6	100.75	101.7	1.00942928	0.621842
O60716	CTNND1	Catenin delta-1 OS=Homo sapiens GN=CTNI	15	103.2	98.7	101.6	102.2	100.95	101.9	1.009410599	0.622077
A0A024RBV4	MID1	Midline 1 (Opitz/BBB syndrome), isoform CI	2	102.3	100	100.5	103.7	101.15	102.1	1.009391992	0.622309
Q8WVX7		Ribosomal protein S19 (Fragment) OS=Homo	14	97	105.5	102.7	101.7	101.25	102.2	1.009382716	0.622426
B5MBX0	CDCA5	Sororin OS=Homo sapiens GN=CDCA5 PE=	2	103.9	100	95.6	110.2	101.95	102.9	1.009318293	0.623231
B2RDJ6	CIAO1	Probable cytosolic iron-sulfur protein assemb	4	96.9	96.4	98.5	96.6	96.65	97.55	1.00931195	0.623312
P38159	RBMX	RNA-binding motif protein, X chromosome C	6	95.4	98.8	97.5	98.5	97.1	98	1.009268795	0.623853
B3KMR5		cDNA FLJ12434 fis, clone NT2RM1000037,	31	98.9	97	100.6	97.1	97.95	98.85	1.009188361	0.62486
Q9HC10	OTOF	Otoferlin OS=Homo sapiens GN=OTOF PE=	1	92.3	103.7	92	105.8	98	98.9	1.009183673	0.624919
Q71UF1	ACO2	Aconitate hydratase, mitochondrial OS=Hom	2	87.2	109.1	95.8	102.3	98.15	99.05	1.009169638	0.625096
Q9H0S4	DDX47	Probable ATP-dependent RNA helicase DDX	13	97.5	99.8	100.8	98.3	98.65	99.55	1.009123163	0.625677
Q9NRC1	ST7	Suppressor of tumorigenicity 7 protein OS=H	1	95.2	102.4	95.9	103.5	98.8	99.7	1.009109312	0.625852
B2R791		cDNA, FLJ93335, highly similar to Homo sap	24	99.4	99.2	100.6	99.8	99.3	100.2	1.009063444	0.626427
P35249	RFC4	Replication factor C subunit 4 OS=Homo sapi	7	100.3	98.5	98.3	102.3	99.4	100.3	1.009054326	0.626542
A0A0U1RRK	MICU1	Calcium uptake protein 1, mitochondrial OS=	4	103.1	96.4	100.3	101	99.75	100.65	1.009022556	0.62694
Q8NCK5		cDNA FLJ90195 fis, clone MAMMA100131(	14	102.5	97.2	100.3	101.2	99.85	100.75	1.00901352	0.627054
P12931	SRC	Proto-oncogene tyrosine-protein kinase Src O	4	101.7	98.7	99.7	102.5	100.2	101.1	1.008982036	0.627449
A0A140VK17	EHBP1	EH domain binding protein 1, isoform CRA_a	1	92.4	108.1	104	98.3	100.25	101.15	1.008977556	0.627506
B3KMC9		cDNA FLJ10711 fis, clone NT2RP3000917, l	27	101.1	100.3	101.2	102	100.7	101.6	1.008937438	0.62801
Q6FHF7	RABGGTA	RABGGTA protein (Fragment) OS=Homo sa	5	101.1	101.6	99.6	104.9	101.35	102.25	1.008880118	0.62873
P46781	RPS9	40S ribosomal protein S9 OS=Homo sapiens (	12	98.3	104.4	103.7	100.8	101.35	102.25	1.008880118	0.62873
O75165	DNAJC13	DnaJ homolog subfamily C member 13 OS=H	28	102.6	100.6	102.1	102.9	101.6	102.5	1.008858268	0.629005
Q9NY27	PPP4R2	Serine/threonine-protein phosphatase 4 regula	4	103.5	100.9	103.2	103	102.2	103.1	1.008806262	0.62966
O75182	SIN3B	Paired amphipathic helix protein Sin3b OS=H	4	95.5	100.2	95.7	101.7	97.85	98.7	1.008686765	0.631164
Q6P1N1	PPM2C	PPM2C protein (Fragment) OS=Homo sapien	2	101.3	95	93	105	98.15	99	1.008660214	0.631499
B2R8G6		cDNA, FLJ93891, Homo sapiens FK506 bind	5	95.4	101.1	96	102.2	98.25	99.1	1.008651399	0.63161
P05412	JUN	Transcription factor AP-1 OS=Homo sapiens	1	97.3	88	95.3	91.6	92.65	93.45	1.008634647	0.63182
Q15182	SNRPB	Small nuclear ribonucleoprotein-associated pr	8	96.5	100.7	99.6	99.3	98.6	99.45	1.00862069	0.631996
Q53GZ6		Heat shock 70kDa protein 8 isoform 1 variant	18	98	100.1	99.8	100	99.05	99.9	1.008581524	0.632491
O75648	TRMU	Mitochondrial tRNA-specific 2-thiouridylase	2	100.2	98	97.2	102.7	99.1	99.95	1.008577195	0.632545
A0A024R1R8	hCG_2014768	HCG2014768, isoform CRA_a OS=Homo sap	1	94.4	104.1	98	102.2	99.25	100.1	1.008564232	0.632709
Q8IXQ6	PARP9	Poly [ADP-ribose] polymerase 9 OS=Homo s	1	103.2	96.2	107	94.1	99.7	100.55	1.008525577	0.633196
P82650	MRPS22	28S ribosomal protein S22, mitochondrial OS	15	100	99.7	100.5	100.9	99.85	100.7	1.008512769	0.633358
P25786	PSMA1	Proteasome subunit alpha type-1 OS=Homo s	12	98.1	102	101.5	100.3	100.05	100.9	1.008495752	0.633572
B4DRS6		Sideroflexin OS=Homo sapiens PE=2 SV=1	2	103	97.4	102.3	99.8	100.2	101.05	1.008483034	0.633733
Q6RFH5	WDR74	WD repeat-containing protein 74 OS=Homo s	11	99.4	101.2	99.7	102.6	100.3	101.15	1.008474576	0.63384
Q07666	KHDRBS1	KH domain-containing, RNA-binding, signal	8	101.2	99.5	102.1	100.3	100.35	101.2	1.008470354	0.633894
Q7L8L6	FASTKD5	FAST kinase domain-containing protein 5, mi	14	101.3	99.7	104.3	98.4	100.5	101.35	1.008457711	0.634054
Q86WK9	PAQR7	Membrane progestin receptor alpha OS=Hom	1	89.4	111.7	93.4	109.4	100.55	101.4	1.008453506	0.634106
Q15149	PLEC	Plectin OS=Homo sapiens GN=PLEC PE=1 S	163	100.7	100.8	101.9	101.3	100.75	101.6	1.008436725	0.634319
P56537	EIF6	Eukaryotic translation initiation factor 6 OS=I	8	99.4	102.2	101.8	101.5	100.8	101.65	1.00843254	0.634371
E9PLN8		Uncharacterized protein (Fragment) OS=Hom	1	96.3	93.7	99	92.6	95	95.8	1.008421053	0.634516
A0A0D9SF53	DDX3X	ATP-dependent RNA helicase DDX3X OS=H	37	100.3	101.9	102.5	101.4	101.1	101.95	1.008407517	0.634687
Q15650	TRIP4	Activating signal cointegrator 1 OS=Homo sa	3	104.3	89.5	96.7	98.7	96.9	97.7	1.008255934	0.636603

A0A024R5J5	RAB6A	H.sapiens ras-related Hrab6 protein OS=Hom	1	98.1	95.9	100.1	95.5	97	97.8	1.008247423	0.636711
A0A024R3R5	LBR	Lamin B receptor, isoform CRA_a OS=Homo	1	92.8	101.5	89	106.9	97.15	97.95	1.008234689	0.636871
O60499	STX10	Syntaxin-10 OS=Homo sapiens GN=STX10 I	4	98.8	96.2	94.6	102	97.5	98.3	1.008205128	0.637245
Q96LZ7	RMDN2	Regulator of microtubule dynamics protein 2	7	98.8	96.2	97.1	99.5	97.5	98.3	1.008205128	0.637245
Q8WUW1	BRK1	Protein BRICK1 OS=Homo sapiens GN=BRI	1	95.8	99.3	97.3	99.4	97.55	98.35	1.008200923	0.637299
A0A024R0C3	NNT	Nicotinamide nucleotide transhydrogenase, is	35	98.5	97.1	99.1	98.1	97.8	98.6	1.008179959	0.637565
P56589	PEX3	Peroxisomal biogenesis factor 3 OS=Homo sa	5	101	95.3	99.5	98.4	98.15	98.95	1.00815079	0.637933
P68104	EEF1A1	Elongation factor 1-alpha 1 OS=Homo sapien	5	95.3	101.5	97.4	101	98.4	99.2	1.008130081	0.638196
A0A024R1U2	PHF5A	PHD finger protein 5A, isoform CRA_a OS=I	8	101	96.9	101.7	97.8	98.95	99.75	1.008084891	0.638768
Q8NB49	ATP11C	Phospholipid-transporting ATPase IG OS=Ho	7	100.5	98.2	99.2	101.1	99.35	100.15	1.00805234	0.639181
O43678	NDUFA2	NADH dehydrogenase [ubiquinone] 1 alpha s	4	98.3	100.9	100	100.8	99.6	100.4	1.008032129	0.639437
E5KND7	GFM1	Elongation factor G, mitochondrial OS=Homo	24	101.1	99.1	102.9	98.9	100.1	100.9	1.007992008	0.639945
A0A024R6S1	DNAJA2	DnaJ (Hsp40) homolog, subfamily A, membe	13	100.7	99.9	102.3	99.9	100.3	101.1	1.007976072	0.640148
Q86X10	RALGAPB	Ral GTPase-activating protein subunit beta O	1	93.7	106.9	94.5	107.7	100.3	101.1	1.007976072	0.640148
O95202	LETM1	LETM1 and EF-hand domain-containing prot	24	101.8	98.9	101.3	101	100.35	101.15	1.007972098	0.640198
Q9Y5T4	DNAJC15	DnaJ homolog subfamily C member 15 OS=H	1	95.6	93.1	95.6	94.6	94.35	95.1	1.007949126	0.64049
A0A0U1RRB6	EXOC6B	Exocyst complex component 6B OS=Homo sa	1	104.4	96.9	97.8	105.1	100.65	101.45	1.007948336	0.6405
Q86YQ8	CPNE8	Copine-8 OS=Homo sapiens GN=CPNE8 PE	10	101.3	100.3	101.1	102.1	100.8	101.6	1.007936508	0.64065
Q14157	UBAP2L	Ubiquitin-associated protein 2-like OS=Homo	18	100.9	101	101.6	101.9	100.95	101.75	1.007924715	0.6408
Q9UHB9	SRP68	Signal recognition particle subunit SRP68 OS	27	100.9	101.7	103.4	100.8	101.3	102.1	1.007897335	0.641146
O00487	PSMD14	26S proteasome non-ATPase regulatory subu	8	101.6	101.2	101.3	103.1	101.4	102.2	1.007889546	0.641246
A1L3A7	NUFIP2	Nuclear fragile X mental retardation protein i	13	105.3	98.3	101.3	103.9	101.8	102.6	1.007858546	0.641639
B3KTM8	MORF4L1	Mortality factor 4-like protein 1 OS=Homo sa	6	96.2	94.9	95.1	97.5	95.55	96.3	1.007849294	0.641756
Q9Y6W5	WASF2	Wiskott-Aldrich syndrome protein family mer	2	103.6	100.7	102.7	103.2	102.15	102.95	1.00783162	0.641981
Q9NZ63	C9orf78	Uncharacterized protein C9orf78 OS=Homo s	3	98.1	98.4	96.8	101.2	98.25	99	1.007633588	0.644498
O60318	MCM3AP	Germinal-center associated nuclear protein O	6	99.1	97.5	99.8	98.3	98.3	99.05	1.007629705	0.644547
A0A024R0G0	CLCC1	Chloride channel CLIC-like 1, isoform CRA_	8	100	97.6	101.8	97.3	98.8	99.55	1.007591093	0.645038
Q9Y2G3	ATP11B	Probable phospholipid-transporting ATPase II	1	100.7	97.4	107.6	92	99.05	99.8	1.007571933	0.645283
Q8NCC3	PLA2G15	Group XV phospholipase A2 OS=Homo sapie	4	100.4	98	96.3	103.6	99.2	99.95	1.007560484	0.645428
Q8TBB5	KLHDC4	Kelch domain-containing protein 4 OS=Homo	2	108.1	104.1	104.7	109.1	106.1	106.9	1.007540057	0.645688
B3KM36		cDNA FLJ10153 fis, clone HEMBA1003417,	10	99.8	99.4	100.7	100	99.6	100.35	1.00753012	0.645815
A5Y5A3		PC1/MRPS28 fusion protein OS=Homo sapie	7	100.7	98.6	101	99.8	99.65	100.4	1.007526342	0.645862
A0A0A0MSG	FHL2	Four and a half LIM domains protein 2 OS=H	4	99.3	100	102	98.8	99.65	100.4	1.007526342	0.645862
A0A024R8S5	P4HB	Protein disulfide-isomerase OS=Homo sapien	37	99.8	99.5	100.6	100.2	99.65	100.4	1.007526342	0.645862
P23434	GCSH	Glycine cleavage system H protein, mitochon	2	99.8	99.8	99	102.1	99.8	100.55	1.00751503	0.646007
Q9HB90	RRAGC	Ras-related GTP-binding protein C OS=Homo	7	99	101.3	100.3	101.5	100.15	100.9	1.007488767	0.646342
Q96DM3	C18orf8	Uncharacterized protein C18orf8 OS=Homo s	3	100.8	99.6	103.4	98.5	100.2	100.95	1.00748503	0.646389
P12270	TPR	Nucleoprotein TPR OS=Homo sapiens GN=T	90	101.5	98.9	100.8	101.1	100.2	100.95	1.00748503	0.646389
A0JNW5	UHRF1BP1L	UHRF1-binding protein 1-like OS=Homo sap	3	98.4	102.1	97.8	104.2	100.25	101	1.007481297	0.646436
Q06210	GFPT1	Glutamine--fructose-6-phosphate aminotransf	21	100.9	99.8	101.8	100.4	100.35	101.1	1.007473842	0.646532
A0A140T9T7	TAP1	Antigen peptide transporter 1 OS=Homo sapie	3	102.3	98.7	96.6	105.9	100.5	101.25	1.007462687	0.646673
Q9H0U9	TSPYL1	Testis-specific Y-encoded-like protein 1 OS=	11	101.4	100.1	101.4	101.6	100.75	101.5	1.007444169	0.64691
B3KXW5		cDNA FLJ46199 fis, clone TESTI4007965, h	13	99.8	102.3	103.5	100.1	101.05	101.8	1.007422068	0.647191
B3KME2		cDNA FLJ10772 fis, clone NT2RP4000243, l	13	100.9	101.3	100.5	103.2	101.1	101.85	1.007418398	0.647239
A0A087X0W7	ACOT2	Acyl-coenzyme A thioesterase 2, mitochondri	12	104.4	99.1	103	102	101.75	102.5	1.007371007	0.647844
E5KNV8		DNA polymerase subunit gamma-1 OS=Hom	4	103.3	100.3	96.7	108.4	101.8	102.55	1.007367387	0.647889
Q9NP97	DYNLRB1	Dynein light chain roadblock-type 1 OS=Hon	5	94.3	96.5	89.6	102.6	95.4	96.1	1.007337526	0.648269
A0A024R962	hCG_40889	HCG40889, isoform CRA_b OS=Homo sapie	1	102.1	103.4	88.4	118.6	102.75	103.5	1.00729927	0.648758
Q9BXK5	BCL2L13	Bcl-2-like protein 13 OS=Homo sapiens GN=	9	99	94.8	97.1	98.1	96.9	97.6	1.007223942	0.649719
Q8N8R5	C2orf69	UPF0565 protein C2orf69 OS=Homo sapiens	1	97.6	98.2	96.6	100.6	97.9	98.6	1.007150153	0.650662
Q9BY49	PECR	Peroxisomal trans-2-enoyl-CoA reductase OS	3	97.9	98.1	97.4	100	98	98.7	1.007142857	0.650755
A8MXV4	NUDT19	Nucleoside diphosphate-linked moiety X moti	8	101.9	94.8	99.5	98.6	98.35	99.05	1.007117438	0.651081
P57740	NUP107	Nuclear pore complex protein Nup107 OS=H	23	101.8	96.3	101.1	98.4	99.05	99.75	1.007067138	0.651724
B2R7D2		cDNA, FLJ93389, highly similar to Homo sap	6	100	98.2	97.8	101.8	99.1	99.8	1.007063572	0.651768
Q9NWS8	RMND1	Required for meiotic nuclear division protein	3	104.8	93.9	100.8	99.3	99.35	100.05	1.007045798	0.651997
A0A024R473	MRPL44	Mitochondrial ribosomal protein L44, isoforn	12	100.8	98	101.1	99.1	99.4	100.1	1.007042254	0.652041
Q9Y679	AUP1	Ancient ubiquitous protein 1 OS=Homo sapie	9	101.5	97.9	101.3	99.5	99.7	100.4	1.007021063	0.652313
A0A024R9L1	AQR	Aquarius homolog (Mouse), isoform CRA_b	21	98.7	100.7	100.8	100	99.7	100.4	1.007021063	0.652313
Q86UE4	MTDH	Protein LYRIC OS=Homo sapiens GN=MTD	22	99.8	99.7	100.5	100.4	99.75	100.45	1.007017544	0.652358
Q70UQ0	IKBIP	Inhibitor of nuclear factor kappa-B kinase-int	8	100.9	98.7	98.5	102.5	99.8	100.5	1.007014028	0.652403
Q9UHV9	PFDN2	Prefoldin subunit 2 OS=Homo sapiens GN=P	6	98.5	101.5	99.2	102.2	100	100.7	1.007	0.652583
O15042	U2SURP	U2 snRNP-associated SURP motif-containing	23	100.5	99.8	101.8	99.9	100.15	100.85	1.006989516	0.652717
B2RBI2		cDNA, FLJ95525, highly similar to Homo sap	10	99.7	101.5	103.6	99	100.6	101.3	1.00695825	0.653117
B4DPV7		cDNA FLJ54534, highly similar to Homo sap	13	102.9	98.4	100.7	102	100.65	101.35	1.006954794	0.653161
O00217	NDUFS8	NADH dehydrogenase [ubiquinone] iron-sulf	3	101.4	100.4	100.2	103	100.9	101.6	1.006937562	0.653382
Q99986	VRK1	Serine/threonine-protein kinase VRK1 OS=H	13	100.5	102.2	102.9	101.2	101.35	102.05	1.006906759	0.653776
P62277	RPS13	40S ribosomal protein S13 OS=Homo sapiens	12	98.8	104.2	102	102.4	101.5	102.2	1.006896552	0.653907
P55036	PSMD4	26S proteasome non-ATPase regulatory subu	13	101.4	101.7	99.3	105.2	101.55	102.25	1.006893156	0.65395
B3KY86		cDNA FLJ16043 fis, clone BRCAN2028355,	3	107.7	99.4	103.7	104.8	103.55	104.25	1.006760019	0.655657
Q9UG56	PISD	Phosphatidylserine decarboxylase proenzyme	3	104	103.2	103.6	105	103.6	104.3	1.006756757	0.655698
Q9UK45	LSM7	U6 snRNA-associated Sm-like protein LSm7	3	98.1	95.6	97.7	97.3	96.85	97.5	1.006711409	0.65628
P34741	SDC2	Syndecan-2 OS=Homo sapiens GN=SDC2 PI	1	103.7	105.1	106.8	103.4	104.4	105.1	1.006704981	0.656363
P58317	ZNF121	Zinc finger protein 121 OS=Homo sapiens GN	2	98.5	97.7	103.1	94.4	98.1	98.75	1.006625892	0.657377
Q5SSJ5	HP1BP3	Heterochromatin protein 1-binding protein 3	17	98.1	98.1	100.3	97.2	98.1	98.75	1.006625892	0.657377
Q7Z4Y4	AK3	GTP:AMP phosphotransferase AK3, mitocho	9	98.6	98.5	100.8	97.6	98.55	99.2	1.006595637	0.657766
D3DWY7	VBP1	von Hippel-Lindau binding protein 1, isoform	6	98.1	99.8	98.5	100.7	98.95	99.6	1.006568974	0.658108
B3KQA0		cDNA FLJ90015 fis, clone HEMBA1000634,	3	100.4	97.8	100.9	98.6	99.1	99.75	1.006559031	0.658236
Q13283	G3BP1	Ras GTPase-activating protein-binding protei	14	98.1	100.2	99	100.6	99.15	99.8	1.006555724	0.658279
Q9Y5L4	TIMM13	Mitochondrial import inner membrane translo	6	98.8	99.6	101.1	98.6	99.2	99.85	1.006552419	0.65832
Q9NP72	RAB18	Ras-related protein Rab-18 OS=Homo sapien	12	100	98.6	99.7	100.2	99.3	99.95	1.006545821	0.658406
A0A0S2Z4B3	RBMS1	RNA binding motif single stranded interacting	2	102.8	96.1	99.5	100.7	99.45	100.1	1.006535948	0.658532
Q9UJ41	RABGEF1	Rab5 GDP/GTP exchange factor OS=Homo s	3	103.8	95.3	96.1	104.3	99.55	100.2	1.006529382	0.658618
Q8NFG4	FLCN	Folliculin OS=Homo sapiens GN=FLCN PE=	2	91.8	108.7	96.6	105.2	100.25	100.9	1.006483791	0.659203
Q5T160	RARS2	Probable arginine--tRNA ligase, mitochondria	7	102.3	98.8	100	102.4	100.55	101.2	1.006464446	0.659451
Q15717	ELAVL1	ELAV-like protein 1 OS=Homo sapiens GN=	13	100.5	101.2	102.9	100.1	100.85	101.5	1.006445216	0.659699
Q96CN7	ISOC1	Isochorismatase domain-containing protein 1	4	101.8	100	104.7	98.4	100.9	101.55	1.006442022	0.659739
P46783	RPS10	40S ribosomal protein S10 OS=Homo sapiens	10	99.6	102.5	102.2	101.2	101.05	101.7	1.006432459	0.659863
A0A087WUM	SYNJ2BP-COX	Protein SYNJ2BP-COX16 (Fragment) OS=H	8	102.7	99.7	104.6	99.1	101.2	101.85	1.006422925	0.659986
A8JZZ5		cDNA FLJ78721, highly similar to Homo sap	1	98.1	91.3	89.6	101	94.7	95.3	1.006335797	0.661106
Q7Z5G4	GOLGA7	Golgin subfamily A member 7 OS=Homo sap	2	116.5	94.3	106.1	106	105.4	106.05	1.006166983	0.663282
F5H5D3	TUBA1C	Tubulin alpha chain OS=Homo sapiens GN=T	2	95.4	100	95.5	101.1	97.7	98.3	1.006141249	0.663612
Q15050	RRS1	Ribosome biogenesis regulatory protein homc	11	98.7	97.2	97.1	100	97.95	98.55	1.006125574	0.663815
Q9NRV9	HEBP1	Heme-binding protein 1 OS=Homo sapiens G	9	97.4	99.6	100.2	98	98.5	99.1	1.006091371	0.664256
Q0P5W4	RCE1	RCE1 protein (Fragment) OS=Homo sapiens	1	100	97.7	97.5	101.4	98.85	99.45	1.006069803	0.664535
Q96SZ6	CDK5RAP1	CDK5 regulatory subunit-associated protein 1	10	98.1	99.8	99.3	99.8	98.95	99.55	1.006063669	0.664613
Q9Y5J1	UTP18	U3 small nucleolar RNA-associated protein 1	13	101.3	96.7	101.5	97.7	99	99.6	1.006060606	0.664653
C9JRZ6	CHCHD3	MICOS complex subunit OS=Homo sapiens (	14	100.9	97.3	100.3	99.1	99.1	99.7	1.00605449	0.664732
Q9ULJ7	ANKRD50	Ankyrin repeat domain-containing protein 50	3	99.9	99	102.2	97.9	99.45	100.05	1.006033183	0.665007
B2RBR9		cDNA, FLJ95650, highly similar to Homo sap	26	100.2	98.9	100.1	100.2	99.55	100.15	1.006027122	0.665085
Q16775	HAGH	Hydroxyacylglutathione hydrolase, mitochon	7	100	99.3	98.1	102.4	99.65	100.25	1.006021074	0.665164
P11234	RALB	Ras-related protein Ral-B OS=Homo sapiens	3	103.5	96	102.4	98.3	99.75	100.35	1.006015038	0.665241
Q86T03	TMEM55B	Type 1 phosphatidylinositol 4,5-bisphosphate	3	105.4	94.2	100.4	100.4	99.8	100.4	1.006012024	0.665279
J3KPZ4	C1D	Nuclear nucleic acid-binding protein C1D OS	2	98.8	101.2	99.7	101.5	100	100.6	1.006	0.665435
P15529	CD46	Membrane cofactor protein OS=Homo sapien	6	100.8	99.4	101.8	99.6	100.1	100.7	1.005994006	0.665513
P49406	MRPL19	39S ribosomal protein L19, mitochondrial OS	11	101	99.2	101.4	100	100.1	100.7	1.005994006	0.665513
B2R983		cDNA, FLJ94267, highly similar to Homo sap	12	100.3	100.3	101.6	100.2	100.3	100.9	1.005982054	0.665667
P61962	DCAF7	DDB1- and CUL4-associated factor 7 OS=Ho	3	101.1	100.3	101.9	100.7	100.7	101.3	1.005958292	0.665973
P54578	USP14	Ubiquitin carboxyl-terminal hydrolase 14 OS	19	99.8	101.6	102.1	100.5	100.7	101.3	1.005958292	0.665973
O43290	SART1	U4/U6.U5 tri-snRNP-associated protein 1 OS	26	101.8	99.7	101.4	101.3	100.75	101.35	1.005955335	0.666012
Q96FZ2	HMCES	Embryonic stem cell-specific 5-hydroxymethy	1	106.1	96.5	98.9	104.9	101.3	101.9	1.005923001	0.666429
A0A024QZ89	COG4	Component of oligomeric golgi complex 4, is	2	109.1	94.8	101.1	104	101.95	102.55	1.005885238	0.666917
Q6ICL7	SLC35E4	Solute carrier family 35 member E4 OS=Hom	1	100.7	104	102.6	103.3	102.35	102.95	1.005862237	0.667215
Q6P2D0	ZFP1	Zinc finger protein 1 homolog OS=Homo sap	1	102.8	90.3	90	104.2	96.55	97.1	1.00569653	0.669358
Q5JPE7	NOMO2	Nodal modulator 2 OS=Homo sapiens GN=N	31	100.8	97.3	100.1	99.1	99.05	99.6	1.005552751	0.67122
A4D0U5	TES	Testis derived transcript (3 LIM domains) OS	11	100.6	97.6	101.2	98.1	99.1	99.65	1.00554995	0.671257
P23258	TUBG1	Tubulin gamma-1 chain OS=Homo sapiens G	6	101.1	97.4	97.5	102.1	99.25	99.8	1.005541562	0.671365
Q6ZVM7	TOM1L2	TOM1-like protein 2 OS=Homo sapiens GN=	11	99.8	98.8	98.8	100.9	99.3	99.85	1.005538771	0.671401
Q13206	DDX10	Probable ATP-dependent RNA helicase DDX	20	100.1	98.7	99.1	100.8	99.4	99.95	1.005533199	0.671473
A0A0S2Z569	DAZAP1	DAZ associated protein 1 isoform 1 (Fragmen	9	98.3	100.7	99.2	100.9	99.5	100.05	1.005527638	0.671546
Q00653	NFKB2	Nuclear factor NF-kappa-B p100 subunit OS=	12	102.7	96.7	102.7	97.8	99.7	100.25	1.00551655	0.67169
Q9BYD1	MRPL13	39S ribosomal protein L13, mitochondrial OS	10	100.7	99.2	100.5	100.5	99.95	100.5	1.005502751	0.671869
P18084	ITGB5	Integrin beta-5 OS=Homo sapiens GN=ITGB	4	100.4	99.6	99	102.1	100	100.55	1.0055	0.671904

A8K4J5		cDNA FLJ76040, highly similar to Homo sap	2	102.3	97.7	90.7	110.4	100	100.55	1.0055	0.671904
A0A024R7I3	RAB8A	RAB8A, member RAS oncogene family, isofo	4	98.5	102.6	102.2	100	100.55	101.1	1.005469915	0.672294
Q8WVQ1	CANT1	Soluble calcium-activated nucleotidase 1 OS=	3	100.1	101.7	100.6	102.3	100.9	101.45	1.005450942	0.67254
O14734	ACOT8	Acyl-coenzyme A thioesterase 8 OS=Homo sa	3	102.8	99.9	97.9	105.9	101.35	101.9	1.005426739	0.672854
Q9NRL2	BAZ1A	Bromodomain adjacent to zinc finger domain	23	103.9	99.1	101.9	102.2	101.5	102.05	1.005418719	0.672957
A0A024R683	ATP6V1D	ATPase, H+ transporting, lysosomal 34kDa, V	5	101.1	104.2	101.5	104.9	102.65	103.2	1.005358013	0.673746
Q9HC06		Cd002 protein OS=Homo sapiens PE=2 SV=1	4	94.6	98.2	97.1	96.7	96.4	96.9	1.005186722	0.675971
Q9NP84	TNFRSF12A	Tumor necrosis factor receptor superfamily m	2	94.2	102.5	97.6	100.1	98.35	98.85	1.005083884	0.677308
W8QEH3	LMNA	Lamin A/C OS=Homo sapiens GN=LMNA P	1	95.3	101.8	96.8	101.3	98.55	99.05	1.005073567	0.677443
Q6KC79	NIPBL	Nipped-B-like protein OS=Homo sapiens GN	9	101.6	95.7	96.6	101.7	98.65	99.15	1.005068424	0.67751
Q6FH36	PPIH	Peptidyl-prolyl cis-trans isomerase (Fragment	9	98.3	99.3	101.4	97.2	98.8	99.3	1.005060729	0.67761
A0A0S2Z4Z9	NONO	Non-POU domain containing octamer-binding	10	98.2	100.2	100.9	98.5	99.2	99.7	1.005040323	0.677876
O60925	PFDN1	Prefoldin subunit 1 OS=Homo sapiens GN=P	4	96.5	102.4	105	94.9	99.45	99.95	1.005027652	0.67804
Q9Y5Q8	GTF3C5	General transcription factor 3C polypeptide 5	9	99.7	99.6	99.8	100.5	99.65	100.15	1.005017561	0.678172
Q9H9Q4	NHEJ1	Non-homologous end-joining factor 1 OS=Ho	1	98.6	100.8	92.1	108.3	99.7	100.2	1.005015045	0.678205
Q03252	LMNB2	Lamin-B2 OS=Homo sapiens GN=LMNB2 P	36	100.9	99.1	101.7	99.3	100	100.5	1.005	0.6784
Q53FE8		cDNA FLJ36526 fis, clone TRACH2003347,	1	96.9	83.3	92.8	88.3	90.1	90.55	1.004994451	0.678473
Q68E01	INTS3	Integrator complex subunit 3 OS=Homo sapie	6	101.9	98.4	100.8	100.5	100.15	100.65	1.004992511	0.678498
A0A024R5H8	RAB6A	RAB6A, member RAS oncogene family, isofo	1	104	96.8	98	103.8	100.4	100.9	1.00498008	0.678659
Q92905	COPS5	COP9 signalosome complex subunit 5 OS=Ho	10	98.5	102.8	101	101.3	100.65	101.15	1.00496771	0.678821
A0A087WUB	CTNNBL1	Beta-catenin-like protein 1 OS=Homo sapiens	13	98.5	102.8	102.6	99.7	100.65	101.15	1.00496771	0.678821
P62269	RPS18	40S ribosomal protein S18 OS=Homo sapiens	11	97.7	104.1	102	100.8	100.9	101.4	1.004955401	0.678981
Q9H553	ALG2	Alpha-1,3/1,6-mannosyltransferase ALG2 OS	5	101.9	100	99.7	103.2	100.95	101.45	1.004952947	0.679013
Q59HG1		Chromosome-associated kinesin KIF4A varia	19	101.6	100.7	101.5	101.8	101.15	101.65	1.004943154	0.679141
O75976	CPD	Carboxypeptidase D OS=Homo sapiens GN=	20	102.5	100	101.8	101.7	101.25	101.75	1.004938272	0.679204
A0A024RBN6	COX6A1	Cytochrome c oxidase subunit 6A, mitochond	1	97.9	107.4	95.7	110.6	102.65	103.15	1.004870921	0.680082
Q32MZ4	LRRFIP1	Leucine-rich repeat flightless-interacting prot	2	97.2	96.2	97	97.3	96.7	97.15	1.004653568	0.682917
P49761	CLK3	Dual specificity protein kinase CLK3 OS=Ho	5	96.5	97.6	93.1	101.9	97.05	97.5	1.004636785	0.683137
Q4LE27	ABCA3	ABCA3 variant protein (Fragment) OS=Hom	4	102.5	94.4	98.7	99.1	98.45	98.9	1.004570848	0.683998
P82912	MRPS11	28S ribosomal protein S11, mitochondrial OS	5	99	98.1	98	100	98.55	99	1.00456621	0.684058
Q9UIG0	BAZ1B	Tyrosine-protein kinase BAZ1B OS=Homo sa	42	98.3	98.9	100.1	98	98.6	99.05	1.004563895	0.684088
J3KSJ5	C17orf62	Uncharacterized protein C17orf62 OS=Homo	2	103.9	94.9	98	101.7	99.4	99.85	1.004527163	0.684568
O75820	ZNF189	Zinc finger protein 189 OS=Homo sapiens GN	1	101.3	97.5	99.8	99.9	99.4	99.85	1.004527163	0.684568
P08621	SNRNP70	U1 small nuclear ribonucleoprotein 70 kDa O	22	99.2	99.6	100.1	99.6	99.4	99.85	1.004527163	0.684568
A0A024RE04	RP11-98F14.6	Uncharacterized protein OS=Homo sapiens G	5	100.2	99.8	99.3	101.6	100	100.45	1.0045	0.684923
B2R7C2		cDNA, FLJ93375, highly similar to Homo sap	6	99.8	100.5	102.2	99	100.15	100.6	1.00449326	0.685012
P09132	SRP19	Signal recognition particle 19 kDa protein OS	4	101	99.4	101.9	99.4	100.2	100.65	1.004491018	0.685042
B2R841		Serine/threonine-protein kinase PLK OS=Hom	6	104.4	96.9	101.3	100.9	100.65	101.1	1.004470939	0.685304
Q9UPN7	PPP6R1	Serine/threonine-protein phosphatase 6 regula	3	100.8	102.4	92.1	112	101.6	102.05	1.004429134	0.685851
A0A087X2I1	PSMC6	26S protease regulatory subunit 10B OS=Hom	17	102.7	100.9	103.1	101.4	101.8	102.25	1.004420432	0.685965
A0A0A0MRR	SAMD8	Sphingomyelin synthase-related protein 1 OS	2	107.2	96.5	100.2	104.4	101.85	102.3	1.004418262	0.685993
P26196	DDX6	Probable ATP-dependent RNA helicase DDX	14	101.5	103.6	103	103	102.55	103	1.004388103	0.686387
Q5IRN4	MEF2D/DAZA	Myocyte enhancer factor 2D/deleted in azoos	1	103.3	102.2	100.6	105.8	102.75	103.2	1.004379562	0.6865
P62081	RPS7	40S ribosomal protein S7 OS=Homo sapiens	10	102.5	103.2	104.7	101.9	102.85	103.3	1.004375304	0.686554
A8K124		cDNA FLJ76213, highly similar to Homo sap	1	104.9	88.7	86.2	108.2	96.8	97.2	1.004132231	0.689739
Q9H5Z1	DHX35	Probable ATP-dependent RNA helicase DHX	2	97.4	98.6	93.8	103	98	98.4	1.004081633	0.690403
Q9H1K1	ISCU	Iron-sulfur cluster assembly enzyme ISCU, m	6	97	100	97.7	100.1	98.5	98.9	1.004060914	0.690675
P62942	FKBP1A	Peptidyl-prolyl cis-trans isomerase FKBP1A	3	97	101.2	97.1	101.9	99.1	99.5	1.004036327	0.690997
Q6NXE6	ARMC6	Armadillo repeat-containing protein 6 OS=Ho	5	97.8	100.4	95.3	103.7	99.1	99.5	1.004036327	0.690997
H7BYT1	CSNK1D	Casein kinase I isoform delta OS=Homo sapie	1	97.6	101	97.6	101.8	99.3	99.7	1.004028197	0.691104
A0A0R4J2E8	MATR3	Matrin-3 OS=Homo sapiens GN=MATR3 PE	33	99.3	100.4	101.4	99.1	99.85	100.25	1.004006009	0.691395
A8K5C2		cDNA FLJ75055 OS=Homo sapiens PE=2 SV	1	95	104.9	94.5	106.2	99.95	100.35	1.004002001	0.691448
O60506	SYNCRIP	Heterogeneous nuclear ribonucleoprotein Q O	19	100.9	99.1	100.9	99.9	100	100.4	1.004	0.691475
P08559	PDHA1	Pyruvate dehydrogenase E1 component subun	22	99.9	100.2	101.3	99.6	100.05	100.45	1.003998001	0.6915
Q6ZYL4	GTF2H5	General transcription factor IIH subunit 5 OS	1	94.2	106	97	104	100.1	100.5	1.003996004	0.691526
P17019	ZNF708	Zinc finger protein 708 OS=Homo sapiens GN	1	101.4	98.9	101.4	99.7	100.15	100.55	1.003994009	0.691553
Q9NSE4	IARS2	Isoleucine--tRNA ligase, mitochondrial OS=H	28	101.8	99	101.5	100.1	100.4	100.8	1.003984064	0.691683
Q6IB11	PGRMC1	PGRMC1 protein OS=Homo sapiens GN=PG	6	96	105.6	104.5	97.9	100.8	101.2	1.003968254	0.691891
Q13185	CBX3	Chromobox protein homolog 3 OS=Homo sap	9	101.2	101.5	103	100.5	101.35	101.75	1.003946719	0.692174
Q9BUN8	DERL1	Derlin-1 OS=Homo sapiens GN=DERL1 PE=	4	103	99.8	101	102.6	101.4	101.8	1.003944773	0.692199
Q5VYS8	ZCCHC6	Terminal uridylyltransferase 7 OS=Homo sap	2	103.2	99.6	97.9	105.7	101.4	101.8	1.003944773	0.692199
O15371	EIF3D	Eukaryotic translation initiation factor 3 subu	17	101.3	101.8	102.7	101.2	101.55	101.95	1.003938946	0.692276
Q59GV6		Zinedin variant (Fragment) OS=Homo sapien	2	102.5	100.8	102.2	101.9	101.65	102.05	1.003935071	0.692326
F5H2A4	HMGA2	High mobility group protein HMGI-C OS=Ho	4	97.2	110.8	105.5	103.3	104	104.4	1.003846154	0.693495
Q8IUE6	HIST2H2AB	Histone H2A type 2-B OS=Homo sapiens GN	1	94.8	101.1	96.2	100.4	97.95	98.3	1.003573252	0.697085
O95831	AIFM1	Apoptosis-inducing factor 1, mitochondrial O	19	99.1	97.3	98.7	98.4	98.2	98.55	1.003564155	0.697206
B3KM34		cDNA FLJ10132 fis, clone HEMBA1003046,	18	98.5	98	99	98.2	98.25	98.6	1.003562341	0.697229
B4DUT2		cDNA FLJ53206 OS=Homo sapiens PE=2 SV	12	100.8	95.8	98.6	98.7	98.3	98.65	1.003560529	0.697253
Q9H788	SH2D4A	SH2 domain-containing protein 4A OS=Hom	2	97.1	100.4	97.8	100.4	98.75	99.1	1.003544304	0.697467
A0A0S2Z410	HSD17B10	Hydroxysteroid dehydrogenase 10 isoform 1 (	14	99.9	97.9	98.6	99.9	98.9	99.25	1.003538928	0.697537
Q86U86	PBRM1	Protein polybromo-1 OS=Homo sapiens GN=	23	98.2	99.7	97.8	100.8	98.95	99.3	1.00353714	0.697561
Q13610	PWP1	Periodic tryptophan protein 1 homolog OS=H	8	100	98.6	98.1	101.2	99.3	99.65	1.003524673	0.697726
Q53HE6		HSPC163 protein variant (Fragment) OS=Hom	1	98.8	100.3	97.7	102.1	99.55	99.9	1.003515821	0.697842
P56385	ATP5I	ATP synthase subunit e, mitochondrial OS=H	5	99.1	101.3	102.4	98.7	100.2	100.55	1.003493014	0.698142
Q53GE7		Tetratricopeptide repeat domain 1 variant (Fra	4	99.2	101.3	98	103.2	100.25	100.6	1.003491272	0.698166
Q9NW64	RBM22	Pre-mRNA-splicing factor RBM22 OS=Hom	9	102.1	98.8	102.6	99	100.45	100.8	1.003484321	0.698257
O94776	MTA2	Metastasis-associated protein MTA2 OS=Hor	17	100.3	100.7	103.2	98.5	100.5	100.85	1.003482587	0.69828
A8K900		Mitochondrial GTPase 1 OS=Homo sapiens F	2	99.6	101.5	98.1	103.7	100.55	100.9	1.003480855	0.698302
Q8N7H5	PAF1	RNA polymerase II-associated factor 1 homol	16	101.5	100.1	101.5	100.8	100.8	101.15	1.003472222	0.698417
A0A024R8G6	SSNA1	Sjogren's syndrome nuclear autoantigen 1, iso	1	103.2	98.5	98.7	103.7	100.85	101.2	1.003470501	0.698439
Q05D26	BLK	Tyrosine-protein kinase (Fragment) OS=Hom	1	96.2	106	99.7	103.2	101.1	101.45	1.003461919	0.698552
Q96CP7	TLCD1	Calfacilitin OS=Homo sapiens GN=TLCD1 P	1	102.8	99.8	105.1	98.2	101.3	101.65	1.003455084	0.698643
Q14254	FLOT2	Flotillin-2 OS=Homo sapiens GN=FLOT2 PE	12	102.8	100.1	100.5	103.1	101.45	101.8	1.003449975	0.698711
E9PGM1	EIF4G1	Eukaryotic translation initiation factor 4 gamr	40	103	103.2	104.4	102.5	103.1	103.45	1.003394762	0.699439
Q15119	PDK2	[Pyruvate dehydrogenase (acetyl-transferring)	1	99	89.9	92	97.5	94.45	94.75	1.003176284	0.702322
A0A024QZD9	ZNF668	Zinc finger protein 668, isoform CRA_a OS=	1	91.9	101.4	102.9	91	96.65	96.95	1.003103983	0.703279
A0A0S2Z497	PEX19	Peroxisomal biogenesis factor 19 isoform 1 (F	4	98.5	95.5	97.4	97.2	97	97.3	1.003092784	0.703427
A0A024R719	SLC7A6	Solute carrier family 7 (Cationic amino acid t	1	118.7	79.2	109.3	89.2	98.95	99.25	1.003031834	0.704232
Q9H116	GZF1	GDNF-inducible zinc finger protein 1 OS=Ho	4	92.2	107.2	96	104	99.7	100	1.003009027	0.704534
O75116	ROCK2	Rho-associated protein kinase 2 OS=Homo sa	1	94.2	105.3	85	115.1	99.75	100.05	1.003007519	0.704554
Q6P2Q9	PRPF8	Pre-mRNA-processing-splicing factor 8 OS=I	81	99.6	99.9	101.6	98.5	99.75	100.05	1.003007519	0.704554
D6RJ07	ZNF346	Zinc finger protein 346 OS=Homo sapiens GN	1	102.7	96.9	94.7	105.5	99.8	100.1	1.003006012	0.704575
A8K9K6		cDNA FLJ76962, highly similar to Homo sap	26	100.1	99.9	101.3	99.3	100	100.3	1.003	0.704653
Q86YQ0	HZGJ	HZGJ OS=Homo sapiens GN=HZGJ PE=2 S	3	101.4	98.8	99	101.8	100.1	100.4	1.002997003	0.704693
H3BN98		Uncharacterized protein (Fragment) OS=Hom	2	99	101.2	100	100.8	100.1	100.4	1.002997003	0.704693
Q96DF8	DGCR14	Protein DGCR14 OS=Homo sapiens GN=DG	3	106.9	93.9	94.9	106.5	100.4	100.7	1.002988048	0.704811
P78406	RAE1	mRNA export factor OS=Homo sapiens GN=	13	103	99.7	101.7	101.6	101.35	101.65	1.002960039	0.705182
A0A024R188	C9orf125	Chromosome 9 open reading frame 125, isofo	2	105.4	98.7	98.8	105.9	102.05	102.35	1.002939735	0.705452
Q9HBU6	ETNK1	Ethanolamine kinase 1 OS=Homo sapiens GN	1	91.1	130.7	144	78.4	110.9	111.2	1.00270514	0.70856
A0A087WUW	ARL15	ADP-ribosylation factor-like protein 15 OS=H	2	98.2	94.1	93.4	99.4	96.15	96.4	1.002600104	0.709953
Q9BQ95	ECSIT	Evolutionarily conserved signaling intermedia	10	98	94.5	93.5	99.5	96.25	96.5	1.002597403	0.709988
Q13242	SRSF9	Serine/arginine-rich splicing factor 9 OS=Hor	10	98.5	97.2	97.9	98.3	97.85	98.1	1.002554931	0.710553
P22033	MUT	Methylmalonyl-CoA mutase, mitochondrial O	15	100.6	97.4	97.2	101.3	99	99.25	1.002525253	0.710947
Q99590	SCAF11	Protein SCAF11 OS=Homo sapiens GN=SCA	25	99.8	98.3	98.7	99.9	99.05	99.3	1.002523978	0.710963
A8K359		cDNA FLJ76102, highly similar to Homo sap	19	100.6	98	97.8	101.3	99.3	99.55	1.002517623	0.711049
Q9NVC6	MED17	Mediator of RNA polymerase II transcription	8	104	95.3	101.5	98.3	99.65	99.9	1.002508781	0.711166
Q8NEF9	SRFBP1	Serum response factor-binding protein 1 OS=	5	99.8	99.7	102.4	97.6	99.75	100	1.002506266	0.711199
A0A140VJK1		Testicular tissue protein Li 75 OS=Homo sapi	13	98.3	101.2	99.3	100.7	99.75	100	1.002506266	0.711199
P49593	PPM1F	Protein phosphatase 1F OS=Homo sapiens GN	2	100.5	100	98.1	102.9	100.25	100.5	1.002493766	0.711365
Q15643	TRIP11	Thyroid receptor-interacting protein 11 OS=H	16	100.2	100.3	97.7	103.3	100.25	100.5	1.002493766	0.711365
Q9NZE8	MRPL35	39S ribosomal protein L35, mitochondrial OS	3	102	98.7	101.8	99.4	100.35	100.6	1.002491281	0.711399
E5KSX8		Mitochondrial transcription factor A OS=Hom	7	99.1	102	100.4	101.2	100.55	100.8	1.002486325	0.711463
Q969S3	ZNF622	Zinc finger protein 622 OS=Homo sapiens GN	5	102.8	98.8	100.4	101.7	100.8	101.05	1.002480159	0.711546
Q9C0E8	LNP	Protein lunapark OS=Homo sapiens GN=LNF	5	103.2	98.4	102.4	99.7	100.8	101.05	1.002480159	0.711546
Q9NUQ3	TXLNG	Gamma-taxilin OS=Homo sapiens GN=TXLN	7	100.6	101.4	100.8	101.7	101	101.25	1.002475248	0.711611
Q14694	USP10	Ubiquitin carboxyl-terminal hydrolase 10 OS=	15	102.6	100.1	100.6	102.6	101.35	101.6	1.0024667	0.711725
A7BI36	RRBP1	p180/ribosome receptor OS=Homo sapiens G	46	102.4	100.3	101.9	101.3	101.35	101.6	1.0024667	0.711725
P61247	RPS3A	40S ribosomal protein S3a OS=Homo sapiens	22	100.4	102.9	101.6	102.2	101.65	101.9	1.00245942	0.711821
Q6ZRP7	QSOX2	Sulfhydryl oxidase 2 OS=Homo sapiens GN=	12	101.3	102.6	102	102.4	101.95	102.2	1.002452182	0.711918
O00488	ZNF593	Zinc finger protein 593 OS=Homo sapiens GN	2	94.4	92.3	97	90.1	93.35	93.55	1.002142475	0.716038
Q9NPE3	NOP10	H/ACA ribonucleoprotein complex subunit 3	3	88.6	105.9	92.1	102.8	97.25	97.45	1.002056555	0.717181
Q86WB0	ZC3HC1	Nuclear-interacting partner of ALK OS=Hom	11	101.1	96.4	95.9	102	98.75	98.95	1.002025316	0.717598

Q9Y3B4	SF3B6	Splicing factor 3B subunit 6 OS=Homo sapier	5	101.1	98.5	101.1	98.9	99.8	100	1.002004008	0.717882
Q6NTF9	RHBDD2	Rhomboid domain-containing protein 2 OS=H	1	98.5	101.6	101	99.5	100.05	100.25	1.001999	0.717949
Q9BXP5	SRRT	Serrate RNA effector molecule homolog OS=	33	98.6	101.6	100.4	100.2	100.1	100.3	1.001998002	0.717963
P50402	EMD	Emerin OS=Homo sapiens GN=EMD PE=1 S	11	100	100.3	100.6	100.1	100.15	100.35	1.001997004	0.717976
A8K2U2		cDNA FLJ75392, highly similar to Homo sap	20	101.3	99.3	98.5	102.5	100.3	100.5	1.001994018	0.718016
Q96E39	RBMXL1	RNA binding motif protein, X-linked-like-1 C	5	96.7	104.1	105.1	96.1	100.4	100.6	1.001992032	0.718043
Q96JN0	LCOR	Ligand-dependent corepressor OS=Homo sap	2	100.3	101.2	100.4	101.5	100.75	100.95	1.001985112	0.718135
Q59GY2		Ribosomal protein L4 variant (Fragment) OS=	22	99	103.3	102.4	100.3	101.15	101.35	1.001977261	0.71824
Q53Z07	RPL9	NPC-A-16 OS=Homo sapiens GN=RPL9 PE=	11	101.8	100.9	101.9	101.2	101.35	101.55	1.00197336	0.718291
Q96QK1	VPS35	Vacuolar protein sorting-associated protein 35	19	102.4	100.3	101.5	101.6	101.35	101.55	1.00197336	0.718291
Q09028	RBBP4	Histone-binding protein RBBP4 OS=Homo sa	7	101.5	101.2	101.6	101.5	101.35	101.55	1.00197336	0.718291
B4DTK7		cDNA FLJ61387, highly similar to Homo sap	3	108.3	95.9	104	100.6	102.1	102.3	1.001958864	0.718485
P41091	EIF2S3	Eukaryotic translation initiation factor 2 subu	16	101.5	102.8	103.1	101.6	102.15	102.35	1.001957905	0.718497
Q5SW79	CEP170	Centrosomal protein of 170 kDa OS=Homo sa	7	101.5	103.3	101.7	103.5	102.4	102.6	1.001953125	0.718561
Q8WX93	PALLD	Palladin OS=Homo sapiens GN=PALLD PE=	2	108.1	97.4	107.6	98.3	102.75	102.95	1.001946472	0.71865
A0AV58	STRN3	Striatin, calmodulin binding protein 3 OS=Ho	6	100.4	105.5	103.8	102.5	102.95	103.15	1.001942691	0.718701
Q99856	ARID3A	AT-rich interactive domain-containing proteir	1	102	110.5	112.3	100.6	106.25	106.45	1.001882353	0.719506
A0A140VJF4		Biliverdin reductase A OS=Homo sapiens PE	5	98.5	95.8	94.4	100.2	97.15	97.3	1.001544004	0.724026
Q9BVC4	MLST8	Target of rapamycin complex subunit LST8 C	2	101.2	96	97.6	99.9	98.6	98.75	1.001521298	0.724329
O43808	SLC25A17	Peroxisomal membrane protein PMP34 OS=H	4	98.2	99.1	97.8	99.8	98.65	98.8	1.001520527	0.72434
P14635	CCNB1	G2/mitotic-specific cyclin-B1 OS=Homo sapi	4	101.6	97.4	100.5	98.5	99.5	99.65	1.001507538	0.724514
J3KPP4	LUC7L3	Cisplatin resistance-associated overexpressed	12	101.7	98.4	102.5	97.9	100.05	100.2	1.00149925	0.724624
P05387	RPLP2	60S acidic ribosomal protein P2 OS=Homo sa	7	100.3	100.1	99.3	101.4	100.2	100.35	1.001497006	0.724654
P17152	TMEM11	Transmembrane protein 11, mitochondrial OS	4	99.3	101.5	102.1	99	100.4	100.55	1.001494024	0.724694
A0A0S2Z2Z6	ANXA6	Annexin (Fragment) OS=Homo sapiens GN=.	33	99.6	101.5	101.6	99.8	100.55	100.7	1.001491795	0.724724
Q9P0I2	EMC3	ER membrane protein complex subunit 3 OS=	6	102.2	99.3	101.1	100.7	100.75	100.9	1.001488834	0.724764
A0A0D9SGF6	SPTAN1	Spectrin alpha chain, non-erythrocytic 1 OS=l	1	104	98.8	91.6	111.5	101.4	101.55	1.00147929	0.724892
O94973	AP2A2	AP-2 complex subunit alpha-2 OS=Homo sap	9	102.3	100.5	101.8	101.3	101.4	101.55	1.00147929	0.724892
H0YE88	TEAD1	Transcriptional enhancer factor TEF-1 OS=H	3	101.6	101.3	101	102.2	101.45	101.6	1.001478561	0.724901
A0A024RAI1	ACTR3	ARP3 actin-related protein 3 homolog (Yeast)	19	101.4	102.3	102	102	101.85	102	1.001472754	0.72498
P35240	NF2	Merlin OS=Homo sapiens GN=NF2 PE=1 SV	10	102.2	102	101.5	103	102.1	102.25	1.001469148	0.725027
Q14444	CAPRIN1	Caprin-1 OS=Homo sapiens GN=CAPRIN1 F	12	104.1	101.2	102.9	102.7	102.65	102.8	1.001461276	0.725133
A0A024R5C6	NAT11	N-acetyltransferase 11, isoform CRA_a OS=H	1	102.7	103.9	106.7	100.2	103.3	103.45	1.001452081	0.725256
Q5HYL0	DKFZp686E01	Putative uncharacterized protein DKFZp686E	1	109.3	100.6	107.8	102.4	104.95	105.1	1.001429252	0.725562
P04083	ANXA1	Annexin A1 OS=Homo sapiens GN=ANXA1	24	94.1	98.4	94.9	97.8	96.25	96.35	1.001038961	0.730795
A0A087WUW	USP17L15	Ubiquitin carboxyl-terminal hydrolase 17-like	1	97.2	96.6	94	100	96.9	97	1.001031992	0.730888
Q96F85	CNRIP1	CB1 cannabinoid receptor-interacting protein	1	108	91.5	96.3	103.4	99.75	99.85	1.001002506	0.731283
A8K5Y7		cDNA FLJ78655, highly similar to Homo sap	16	98	101.7	100.4	99.5	99.85	99.95	1.001001502	0.731297
M0R2Z9	SUGP2	SURP and G-patch domain-containing proteir	25	100.8	99.3	101.2	99.1	100.05	100.15	1.0009995	0.731325
P35579	MYH9	Myosin-9 OS=Homo sapiens GN=MYH9 PE=	86	99.3	100.9	100.8	99.6	100.1	100.2	1.000999001	0.731331
Q7Z6M4	MTERF4	Transcription termination factor 4, mitochond	1	103.2	97.2	105.2	95.4	100.2	100.3	1.000998004	0.731344
Q5BJF2	TMEM97	Transmembrane protein 97 OS=Homo sapiens	3	105.5	94.9	102.7	97.9	100.2	100.3	1.000998004	0.731344
Q96PK6	RBM14	RNA-binding protein 14 OS=Homo sapiens C	24	99.8	100.8	101.3	99.5	100.3	100.4	1.000997009	0.731357
Q59EU6		EF hand domain family, member D1 variant (	1	108.3	93	95.6	105.9	100.65	100.75	1.000993542	0.731405
Q13616	CUL1	Cullin-1 OS=Homo sapiens GN=CUL1 PE=1	14	100.6	100.8	98.6	103	100.7	100.8	1.000993049	0.731411
Q8NCS4	TMEM35B	Transmembrane protein 35B OS=Homo sapie	1	113	88.5	106.2	95.5	100.75	100.85	1.000992556	0.731418
Q96T60	PNKP	Bifunctional polynucleotide phosphatase/kina	3	100.9	101.5	100.1	102.5	101.2	101.3	1.000988142	0.731477
O75569	PRKRA	Interferon-inducible double-stranded RNA-de	5	103.4	99.4	100.7	102.3	101.4	101.5	1.000986193	0.731502
P61764	STXBP1	Syntaxin-binding protein 1 OS=Homo sapiens	9	104.2	98.9	99.5	103.8	101.55	101.65	1.000984737	0.731522
Q9Y383	LUC7L2	Putative RNA-binding protein Luc7-like 2 OS	11	101.7	101.5	100.2	103.2	101.6	101.7	1.000984252	0.731528
Q9P0H9	RER1	RER1 protein OS=Homo sapiens GN=RER1	3	100.8	102.6	104.9	98.7	101.7	101.8	1.000983284	0.731542
B2R6F5		Protein XRP2 OS=Homo sapiens PE=2 SV=1	2	106.3	99.6	104.2	101.9	102.95	103.05	1.000971345	0.731703
Q2TBE0	CWF19L2	CWF19-like protein 2 OS=Homo sapiens GN	1	102.7	113.3	111.9	104.3	108	108.1	1.000925926	0.732313
A0A140VK56		Transaldolase OS=Homo sapiens PE=2 SV=1	17	93.5	102.3	97.7	98.2	97.9	97.95	1.000510725	0.7379
Q9H0E9	BRD8	Bromodomain-containing protein 8 OS=Hom	2	99.5	97	99.8	96.8	98.25	98.3	1.000508906	0.737924
P49411	TUFM	Elongation factor Tu, mitochondrial OS=Hom	28	100.2	98.8	99.7	99.4	99.5	99.55	1.000502513	0.738011
A0A142I7X2		NUP98/PHF23 fusion 2 protein (Fragment) O	2	98.7	100.6	101.1	98.3	99.65	99.7	1.000501756	0.738021
P33897	ABCD1	ATP-binding cassette sub-family D member 1	8	98.8	100.8	98	101.7	99.8	99.85	1.000501002	0.73803
A0A024RDG1	VDP	Vesicle docking protein p115, isoform CRA_a	22	100.5	99.3	101.8	98.1	99.9	99.95	1.000500501	0.738037
Q53GF0		Cytidine 5'-monophosphate N-acetylneuramir	10	101.6	98.6	101.3	99	100.1	100.15	1.0004995	0.738051
B2RDY9		Adenylyl cyclase-associated protein OS=Hom	20	100.1	100.3	99.5	101	100.2	100.25	1.000499002	0.738057
A8K874		cDNA FLJ77588, highly similar to Homo sap	8	99.4	101.1	97.3	103.3	100.25	100.3	1.000498753	0.738061
O14617	AP3D1	AP-3 complex subunit delta-1 OS=Homo sapi	15	99.5	101.2	99.6	101.2	100.35	100.4	1.000498256	0.738067
B3KPX5		cDNA FLJ32418 fis, clone SKMUS2000863,	3	100.6	100.3	102.6	98.4	100.45	100.5	1.00049776	0.738074
Q96I51	RCC1L	RCC1-like G exchanging factor-like protein C	10	103.3	97.8	101.9	99.3	100.55	100.6	1.000497265	0.738082
Q13098	GPS1	COP9 signalosome complex subunit 1 OS=H	7	98.8	104	96.2	106.7	101.4	101.45	1.000493097	0.738138
A0A024R1X0	CASC3	Cancer susceptibility candidate 3, isoform CR	1	95.5	107.8	100.9	102.5	101.65	101.7	1.000491884	0.738154
Q9BQ61	C19orf43	Uncharacterized protein C19orf43 OS=Homo	6	99.1	104.3	100.9	102.6	101.7	101.75	1.000491642	0.738157
R4GMR5	PSMD8	26S proteasome non-ATPase regulatory subu	9	99.7	104.2	102.5	101.5	101.95	102	1.000490436	0.738173
A0A0G2JPQ2	NLRP2	NACHT, LRR and PYD domains-containing	7	93	100.3	94.8	98.5	96.65	96.65	1	0.744794
P19474	TRIM21	E3 ubiquitin-protein ligase TRIM21 OS=Hom	1	83.7	109.8	92.5	101	96.75	96.75	1	0.744794
P55212	CASP6	Caspase-6 OS=Homo sapiens GN=CASP6 PE	2	100.4	94.4	98.1	96.7	97.4	97.4	1	0.744794
Q9BRT2	UQCC2	Ubiquinol-cytochrome-c reductase complex a	2	99.1	96.2	97.6	97.7	97.65	97.65	1	0.744794
Q96GY3	LIN37	Protein lin-37 homolog OS=Homo sapiens GI	2	100.7	96.1	92.5	104.3	98.4	98.4	1	0.744794
E5KS95	TSFM	Elongation factor Ts, mitochondrial OS=Hom	9	99.9	98	100	97.9	98.95	98.95	1	0.744794
F1T0L5	DDX51	ATP-dependent RNA helicase DDX51 OS=H	11	97	101.2	98.2	100	99.1	99.1	1	0.744794
Q15181	PPA1	Inorganic pyrophosphatase OS=Homo sapiens	20	97.5	101.3	99.1	99.7	99.4	99.4	1	0.744794
J9JIC5	C17orf75	Protein Njmu-R1 OS=Homo sapiens GN=C1	6	103	96.5	95.8	103.7	99.75	99.75	1	0.744794
A0AVT1	UBA6	Ubiquitin-like modifier-activating enzyme 6 C	15	98.7	101.2	98.7	101.2	99.95	99.95	1	0.744794
A0A024R7M6	GATAD2A	GATA zinc finger domain containing 2A, isof	10	99.2	100.8	100.1	99.9	100	100	1	0.744794
Q96EH3	MALSU1	Mitochondrial assembly of ribosomal large su	2	101.4	100.1	100.7	100.8	100.75	100.75	1	0.744794
K7EJV9	RPL23A	60S ribosomal protein L23a (Fragment) OS=H	11	101.6	102.4	103.1	100.9	102	102	1	0.744794
F1JVV5	EWSR1/ATF1	EWSR1/ATF1 fusion protein type 1 OS=Hom	1	99	105.7	98.3	106.4	102.35	102.35	1	0.744794
Q8TCT8	SPPL2A	Signal peptide peptidase-like 2A OS=Homo s	3	108.2	97	103	102.2	102.6	102.6	1	0.744794
Q5VTL8	PRPF38B	Pre-mRNA-splicing factor 38B OS=Homo sap	6	103.1	102.2	103.1	102.2	102.65	102.65	1	0.744794
Q14651	PLS1	Plastin-1 OS=Homo sapiens GN=PLS1 PE=1	8	103.7	102.1	99.5	106.3	102.9	102.9	1	0.744794
A8K2T4		cDNA FLJ78207, highly similar to Human co	3	107	103.7	113.5	97.2	105.35	105.35	1	0.744794
Q8N5I2	ARRDC1	Arrestin domain-containing protein 1 OS=Hor	1	117.8	94.4	99.7	112.5	106.1	106.1	1	0.744794
B2RAI2		cDNA, FLJ94931, highly similar to Homo sap	3	94.9	100	100.3	94.6	97.45	97.45	1	0.744794
X5DR09	GTF2I	General transcription factor IIi isoform A (Fra	46	100.2	100.7	101.3	99.6	100.45	100.45	1	0.744794
Q9BRX9	WDR83	WD repeat domain-containing protein 83 OS=	2	96.4	105.2	103	98.6	100.8	100.8	1	0.744794
P18615	NELFE	Negative elongation factor E OS=Homo sapie	9	104.4	100.8	100	105.1	102.6	102.55	0.999512671	0.751396
Q8TEQ6	GEMIN5	Gem-associated protein 5 OS=Homo sapiens	20	101.9	101.3	102.6	100.5	101.6	101.55	0.999507874	0.751461
P78537	BLOC1S1	Biogenesis of lysosome-related organelles cor	2	98.6	103	100.6	100.9	100.8	100.75	0.999503968	0.751514
P34932	HSPA4	Heat shock 70 kDa protein 4 OS=Homo sapie	41	99.3	101.5	100	100.7	100.4	100.35	0.999501992	0.751541
O14744	PRMT5	Protein arginine N-methyltransferase 5 OS=H	17	96.6	103.8	100.6	99.7	100.2	100.15	0.999500998	0.751554
Q9NRZ5	AGPAT4	1-acyl-sn-glycerol-3-phosphate acyltransferas	1	103.7	96.7	95	105.3	100.2	100.15	0.999500998	0.751554
P21912	SDHB	Succinate dehydrogenase [ubiquinone] iron-su	10	101	99	101.2	98.7	100	99.95	0.9995	0.751568
Q02218	OGDH	2-oxoglutarate dehydrogenase, mitochondrial	34	101.8	98.1	100	99.8	99.95	99.9	0.99949975	0.751571
O75880	SCO1	Protein SCO1 homolog, mitochondrial OS=H	4	99.8	99.8	99.6	99.9	99.8	99.75	0.999498998	0.751581
O75400	PRPF40A	Pre-mRNA-processing factor 40 homolog A C	16	101.3	98.2	99.6	99.8	99.75	99.7	0.999498747	0.751585
O95861	BPNT1	3'(2'),5'-bisphosphate nucleotidase 1 OS=Hom	7	97.5	101.9	100.7	98.6	99.7	99.65	0.999498495	0.751588
Q66PJ3	ARL6IP4	ADP-ribosylation factor-like protein 6-interac	3	102.2	97.2	101.6	97.7	99.7	99.65	0.999498495	0.751588
B2R604		cDNA, FLJ92713, highly similar to Homo sap	1	100	99.3	93.5	105.7	99.65	99.6	0.999498244	0.751592
O15379	HDAC3	Histone deacetylase 3 OS=Homo sapiens GN	2	101.6	97.5	98.6	100.4	99.55	99.5	0.99949774	0.751599
O60287	URB1	Nucleolar pre-ribosomal-associated protein 1	19	101.4	97.3	99.1	99.5	99.35	99.3	0.999496729	0.751612
C9JME2	FARP1	FERM, RhoGEF and pleckstrin domain-conta	1	108	90.6	98.9	99.6	99.3	99.25	0.999496475	0.751616
P30084	ECHS1	Enoyl-CoA hydratase, mitochondrial OS=Hor	18	98.6	99.9	98.9	99.5	99.25	99.2	0.999496222	0.751619
Q5H9N4	DKFZp686L20	Putative uncharacterized protein DKFZp686L	11	98.7	99.7	99.2	99.1	99.2	99.15	0.999495968	0.751622
Q9UL26	RAB22A	Ras-related protein Rab-22A OS=Homo sapie	3	99.1	98.6	98	99.6	98.85	98.8	0.999494183	0.751647
P61970	NUTF2	Nuclear transport factor 2 OS=Homo sapiens	5	99.1	97.6	97.4	99.2	98.35	98.3	0.999491612	0.751681
Q5HYI8	RABL3	Rab-like protein 3 OS=Homo sapiens GN=RA	3	97	90.1	92.6	94.4	93.55	93.5	0.999465526	0.752036
A0A0S2Z556	PQBP1	Polyglutamine binding protein 1 isoform 2 (Fr	4	98.7	107.1	99.5	106.1	102.9	102.8	0.999028183	0.75798
Q9UPQ9	TNRC6B	Trinucleotide repeat-containing gene 6B prote	3	103.4	101.5	100.4	104.3	102.45	102.35	0.999023914	0.758038
Q9Y4A1		Talin-related protein (Fragment) OS=Homo sa	1	103.2	101.5	105.9	98.6	102.35	102.25	0.99902296	0.758051
Q96MH6	TMEM68	Transmembrane protein 68 OS=Homo sapiens	1	110.1	94.2	101.5	102.6	102.15	102.05	0.999021047	0.758077
P01137	TGFB1	Transforming growth factor beta-1 OS=Homo	5	101.2	103.1	103.8	100.3	102.15	102.05	0.999021047	0.758077
Q0VDF9	HSPA14	Heat shock 70 kDa protein 14 OS=Homo sapi	11	99.5	104	100.1	103.2	101.75	101.65	0.999017199	0.75813
P26641	EEF1G	Elongation factor 1-gamma OS=Homo sapien	22	99.5	103.6	101.4	101.5	101.55	101.45	0.999015263	0.758156
Q8N766	EMC1	ER membrane protein complex subunit 1 OS=	25	103.5	99.5	102.4	100.4	101.5	101.4	0.999014778	0.758162
Q6Y1H2	HACD2	Very-long-chain (3R)-3-hydroxyacyl-CoA de	1	100.4	102.3	108.5	94	101.35	101.25	0.99901332	0.758183
P23368	ME2	NAD-dependent malic enzyme, mitochondria	21	101.9	100	100.7	101	100.95	100.85	0.999009411	0.758235

Q92575	UBXN4	UBX domain-containing protein 4 OS=Homo	3	100.3	101.4	103.3	98.2	100.85	100.75	0.999008428	0.758249
O00411	POLRMT	DNA-directed RNA polymerase, mitochondri	3	102.2	99.2	98	103.2	100.7	100.6	0.999006951	0.75827
B5BUB5	SSB	Autoantigen La (Fragment) OS=Homo sapien	29	100.6	100.6	101.5	99.5	100.6	100.5	0.999005964	0.758283
Q9Y2W2	WBP11	WW domain-binding protein 11 OS=Homo sa	9	99.6	101.1	99.2	101.3	100.35	100.25	0.999003488	0.758317
Q02447	SP3	Transcription factor Sp3 OS=Homo sapiens G	2	100.4	100.3	104.3	96.2	100.35	100.25	0.999003488	0.758317
A0A0A0MT6	MUC15	Mucin-15 OS=Homo sapiens GN=MUC15 PE	2	99.4	101.3	100.8	99.7	100.35	100.25	0.999003488	0.758317
A8K7Q1		cDNA FLJ77770, highly similar to Homo sap	17	100.6	100	98	102.4	100.3	100.2	0.999002991	0.758323
Q9C0C4	SEMA4C	Semaphorin-4C OS=Homo sapiens GN=SEM	2	104.5	96.1	101.6	98.8	100.3	100.2	0.999002991	0.758323
O75691	UTP20	Small subunit processome component 20 hom	37	100.1	99.7	100.3	99.3	99.9	99.8	0.998998999	0.758377
P61981	YWHAG	14-3-3 protein gamma OS=Homo sapiens GN	8	101.9	97.4	101.3	97.8	99.65	99.55	0.998996488	0.758412
Q53F02		Cleavage and polyadenylation specific factor	15	98.5	100.4	97.3	101.4	99.45	99.35	0.99899447	0.758439
P09936	UCHL1	Ubiquitin carboxyl-terminal hydrolase isozym	8	97	101.8	100.4	98.2	99.4	99.3	0.998993964	0.758446
Q9Y6G9	DYNC1LI1	Cytoplasmic dynein 1 light intermediate chair	5	99.9	98.8	96.6	101.9	99.35	99.25	0.998993457	0.758453
Q86V81	ALYREF	THO complex subunit 4 OS=Homo sapiens G	10	99.9	97.7	96.8	100.6	98.8	98.7	0.998987854	0.758529
P07948	LYN	Tyrosine-protein kinase Lyn OS=Homo sapie	1	101.1	95.8	96.9	99.8	98.45	98.35	0.998984256	0.758578
Q9NVU7	SDAD1	Protein SDA1 homolog OS=Homo sapiens GI	5	99.3	97.6	93.1	103.6	98.45	98.35	0.998984256	0.758578
Q9NPE2	NGRN	Neugrin OS=Homo sapiens GN=NGRN PE=	2	96.3	100.3	98.1	98.3	98.3	98.2	0.998982706	0.758599
P38432	COIL	Coilin OS=Homo sapiens GN=COIL PE=1 S	5	95.7	97.6	98.3	94.8	96.65	96.55	0.998965339	0.758836
H9ZYJ2	TXN	Thioredoxin OS=Homo sapiens GN=TXN PE	7	92.4	100.9	95.8	97.3	96.65	96.55	0.998965339	0.758836
A0A024R914	CEP350	Centrosomal protein 350kDa, isoform CRA_a	1	105.1	99.2	100	104	102.15	102	0.998531571	0.764751
Q96PU4	UHRF2	E3 ubiquitin-protein ligase UHRF2 OS=Hom	1	104.8	98.5	104.5	98.5	101.65	101.5	0.998524348	0.764849
P20020	ATP2B1	Plasma membrane calcium-transporting ATPa	9	100.2	102.6	99.8	102.7	101.4	101.25	0.99852071	0.764899
O75063	FAM20B	Glycosaminoglycan xylosylkinase OS=Homo	4	101	99.1	96.7	103.1	100.05	99.9	0.99850075	0.765172
Q5J7U2	CDK105	TGF beta-inducible nuclear protein 1 OS=Ho	10	102.5	97.5	100.3	99.4	100	99.85	0.9985	0.765182
Q12872	SFSWAP	Splicing factor, suppressor of white-apricot h	9	100.8	98.9	99.3	100.1	99.85	99.7	0.998497747	0.765213
P15336	ATF2	Cyclic AMP-dependent transcription factor A	3	100.6	99	95	104.3	99.8	99.65	0.998496994	0.765223
Q96GC5	MRPL48	39S ribosomal protein L48, mitochondrial OS	6	97.5	101.9	99.1	100	99.7	99.55	0.998495486	0.765244
P14854	COX6B1	Cytochrome c oxidase subunit 6B1 OS=Hom	6	99.7	99.1	98.9	99.6	99.4	99.25	0.998490946	0.765306
Q6PCB5	RSBN1L	Round spermatid basic protein 1-like protein	6	99.4	99.2	98.2	100.1	99.3	99.15	0.998489426	0.765326
Q2M2I8	AAK1	AP2-associated protein kinase 1 OS=Homo sa	2	98.7	99.6	98.1	99.9	99.15	99	0.998487141	0.765358
Q5VWQ0	RSBN1	Round spermatid basic protein 1 OS=Homo s	3	92.4	87.2	90	89.3	89.8	89.65	0.998329621	0.76751
A0A024RDK4	INTS12	Integrator complex subunit 12, isoform CRA_	2	110.2	98	104.1	103.7	104.1	103.9	0.99807877	0.770943
B2ZZ89	SPTBN1	Epididymis luminal protein 102 OS=Homo sa	106	103.5	101.5	103.5	101.1	102.5	102.3	0.99804878	0.771353
Q9NPL8	TIMMDC1	Complex I assembly factor TIMMDC1, mitoc	5	100.9	102.5	102.7	100.3	101.7	101.5	0.998033432	0.771564
Q9Y5A9	YTHDF2	YTH domain-containing family protein 2 OS=	5	102	101.4	104.3	98.7	101.7	101.5	0.998033432	0.771564
O00425	IGF2BP3	Insulin-like growth factor 2 mRNA-binding p	16	103.2	99.8	103.2	99.4	101.5	101.3	0.998029557	0.771617
A0A0G2JMZ	OCLN	Occludin OS=Homo sapiens GN=OCLN PE=	8	102	100.5	102.4	99.7	101.25	101.05	0.998024691	0.771683
X6R4W8	ZNF207	BUB3-interacting and GLEBS motif-containi	5	99.7	102.6	104.6	97.3	101.15	100.95	0.998022739	0.77171
O00743	PPP6C	Serine/threonine-protein phosphatase 6 cataly	9	97.5	104.6	103.3	98.4	101.05	100.85	0.998020782	0.771737
E7EQ34	GOSR2	Golgi SNAP receptor complex member 2 OS=	5	104.5	97.4	98.3	103.2	100.95	100.75	0.998018821	0.771764
Q9NQ29	LUC7L	Putative RNA-binding protein Luc7-like 1 OS	6	99.7	101.6	100.6	100.3	100.65	100.45	0.998012916	0.771845
P62306	SNRPF	Small nuclear ribonucleoprotein F OS=Homo	2	101.5	99.5	99.8	100.8	100.5	100.3	0.99800995	0.771885
P28331	NDUFS1	NADH-ubiquinone oxidoreductase 75 kDa su	29	101	100	102.1	98.5	100.5	100.3	0.99800995	0.771885
Q9BTV4	TMEM43	Transmembrane protein 43 OS=Homo sapiens	4	104.3	96.2	102.7	97.4	100.25	100.05	0.998004988	0.771954
B7ZLQ5	SMARCA1	Probable global transcription activator SNF2L	9	103.6	96.3	100.8	98.7	99.95	99.75	0.997998999	0.772035
B4DHW9		cDNA FLJ56439, highly similar to Pantothen	6	96.9	102.6	100.3	98.8	99.75	99.55	0.997994987	0.772091
Q6PJJ2	RRP1	RRP1 protein (Fragment) OS=Homo sapiens	11	100.6	98.2	100.7	97.7	99.4	99.2	0.997987928	0.772187
O94876	TMCC1	Transmembrane and coiled-coil domains prot	1	87.9	110.8	90.6	107.7	99.35	99.15	0.997986915	0.772201
F6TB26	TDRKH	Tudor and KH domain-containing protein OS	6	100.1	98.3	98	100	99.2	99	0.997983871	0.772243
D3DUE7	N-PAC	Cytokine-like nuclear factor n-pac, isoform C	12	98.3	100	95.8	102.1	99.15	98.95	0.997982854	0.772257
A0A024R419	KIAA0971	KIAA0971, isoform CRA_b OS=Homo sapie	14	98.6	99.2	99.4	98	98.9	98.7	0.997977755	0.772327
O95989	NUDT3	Diphosphoinositol polyphosphate phosphohy	2	91.5	105.6	95.3	101.4	98.55	98.35	0.997970573	0.772425
O75151	PHF2	Lysine-specific demethylase PHF2 OS=Hom	2	95.4	100	95.9	99.1	97.7	97.5	0.997952917	0.772667
Q8N357	SLC35F6	Solute carrier family 35 member F6 OS=Hom	2	96.3	96.1	94.3	97.7	96.2	96	0.997920998	0.773105
A0A128DYX	HLA-C	MHC class I antigen (Fragment) OS=Homo s	2	109.5	101.2	107.7	102.5	105.35	105.1	0.997626958	0.777139
Q8N6N7	ACBD7	Acyl-CoA-binding domain-containing protein	1	97.9	103.6	98.4	102.6	100.75	100.5	0.99751861	0.778627
Q01780	EXOSC10	Exosome component 10 OS=Homo sapiens G	30	100	99.8	99.8	99.5	99.9	99.65	0.997497497	0.778917
A8K761	NDUFB10	NADH dehydrogenase (Ubiquinone) 1 beta su	8	98.5	100.6	98.4	100.2	99.55	99.3	0.997488699	0.779038
Q6IB54	ATP5J	ATP synthase-coupling factor 6, mitochondri	1	99.2	99.8	101.3	97.2	99.5	99.25	0.997487437	0.779056
O94913	PCF11	Pre-mRNA cleavage complex 2 protein Pcf11	6	99.1	99.9	100.7	97.8	99.5	99.25	0.997487437	0.779056
O95159	ZFPL1	Zinc finger protein-like 1 OS=Homo sapiens	3	103	95.8	99.1	99.2	99.4	99.15	0.997484909	0.77909
Q9Y3Z3	SAMHD1	Deoxynucleoside triphosphate triphosphohyd	13	98	100.8	99.2	99.1	99.4	99.15	0.997484909	0.77909
Q8NCA5	FAM98A	Protein FAM98A OS=Homo sapiens GN=FA	7	101.3	96.9	101.9	95.8	99.1	98.85	0.997477296	0.779195
B4DY08	HNRNPC	Heterogeneous nuclear ribonucleoproteins C1	2	96.6	99.2	95	100.3	97.9	97.65	0.997446374	0.77962
B2R4R0	HIST1H4H	Histone H4 OS=Homo sapiens GN=HIST1H4	9	90.6	97.5	92.3	95.3	94.05	93.8	0.997341839	0.781058
Q86YV9	HPS6	Hermansky-Pudlak syndrome 6 protein OS=H	3	91.6	96.3	92.6	94.8	93.95	93.7	0.99733901	0.781096
Q6ZRY4	RBPMS2	RNA-binding protein with multiple splicing 2	4	110.9	109.3	108.4	111.2	110.1	109.8	0.997275204	0.781974
Q6ZNG2	DBX2	Homeobox protein DBX2 OS=Homo sapiens	1	104.5	110.2	113.7	100.4	107.35	107.05	0.997205403	0.782935
Q7Z406	MYH14	Myosin-14 OS=Homo sapiens GN=MYH14 F	4	103	100.7	101.4	101.7	101.85	101.55	0.997054492	0.785013
P84098	RPL19	60S ribosomal protein L19 OS=Homo sapiens	9	99.6	103.8	102	100.8	101.7	101.4	0.997050147	0.785073
O95782	AP2A1	AP-2 complex subunit alpha-1 OS=Homo sap	27	101.6	100.4	101.9	99.5	101	100.7	0.997029703	0.785355
Q9Y6A4	CFAP20	Cilia- and flagella-associated protein 20 OS=I	3	101.7	100	99.3	101.8	100.85	100.55	0.997025285	0.785416
Q14331	FRG1	Protein FRG1 OS=Homo sapiens GN=FRG1	7	102.1	99.3	97.4	103.4	100.7	100.4	0.997020854	0.785477
P62316	SNRPD2	Small nuclear ribonucleoprotein Sm D2 OS=I	8	102.7	98.4	100.2	100.3	100.55	100.25	0.99701641	0.785538
Q6PJV4	THRAP3	THRAP3 protein (Fragment) OS=Homo sapie	1	104.2	96.8	93.1	107.3	100.5	100.2	0.997014925	0.785559
Q9GZL7	WDR12	Ribosome biogenesis protein WDR12 OS=Ho	12	101.5	98.9	99.5	100.3	100.2	99.9	0.997005988	0.785682
B2RA03		cDNA, FLJ94640, highly similar to Homo sap	9	102.4	97.9	101.3	98.4	100.15	99.85	0.997004493	0.785702
E5KRX5	SURF1	SURF1-like protein OS=Homo sapiens GN=S	2	105	95.1	94.2	105.3	100.05	99.75	0.997001499	0.785743
A0A024R5L	PRCP	Prolylcarboxypeptidase (Angiotensinase C), i	7	102.1	97.9	100	99.4	100	99.7	0.997	0.785765
Q6Y7W6	GIGYF2	PERQ amino acid-rich with GYF domain-con	8	100.4	99.4	98.5	100.7	99.9	99.6	0.996996997	0.785806
O43143	DHX15	Pre-mRNA-splicing factor ATP-dependent RI	37	100.4	99.2	99.9	99.1	99.8	99.5	0.996993988	0.785848
Q53G26	DNAJA3	DnaJ (Hsp40) homolog, subfamily A, membe	9	103.3	96	99.8	98.9	99.65	99.35	0.996989463	0.785909
B2RE46		cDNA, FLJ96923, highly similar to Homo sap	22	100	99.3	99.7	99	99.65	99.35	0.996989463	0.785909
Q59G24		Activated RNA polymerase II transcription co	5	99	99.9	99.8	98.5	99.45	99.15	0.996983409	0.785993
A0A0K0K1L	HEL-S-129m	Epididymis secretory sperm binding protein L	13	98	98.6	98.4	97.6	98.3	98	0.996948118	0.78648
Q9Y255	PRELID1	PRELI domain-containing protein 1, mitochor	1	100.7	93.9	99.5	94.5	97.3	97	0.996916752	0.786912
B4DRM3		cDNA FLJ54492, highly similar to Eukaryoti	11	100.2	107.1	103.2	103.4	103.65	103.3	0.996623251	0.790963
Q9Y2Z2	MTO1	Protein MTO1 homolog, mitochondrial OS=F	6	104	100.4	104.4	99.3	102.2	101.85	0.996575342	0.791625
Q14008	CKAP5	Cytoskeleton-associated protein 5 OS=Homo	48	102.2	101.3	101.9	100.9	101.75	101.4	0.996560197	0.791834
P53597	SUCLG1	Succinate--CoA ligase [ADP/GDP-forming] s	7	99.5	103	101.2	100.6	101.25	100.9	0.99654321	0.792069
O14802	POLR3A	DNA-directed RNA polymerase III subunit R	8	102.5	99.5	99.1	102.2	101	100.65	0.996534653	0.792188
P28838	LAP3	Cytosol aminopeptidase OS=Homo sapiens G	23	99.9	101.6	100.5	100.3	100.75	100.4	0.996526055	0.792306
P30419	NMT1	Glycylpeptide N-tetradecanoyltransferase 1 O	9	99.2	102.2	101.2	99.5	100.7	100.35	0.99652433	0.79233
A8K778		cDNA FLJ76902, highly similar to Homo sap	1	102.9	98.5	105.7	95	100.7	100.35	0.99652433	0.79233
O43815	STRN	Striatin OS=Homo sapiens GN=STRN PE=1	13	102.1	99.2	101.4	99.2	100.65	100.3	0.996522603	0.792354
Q13601	KRR1	KRR1 small subunit processome component I	7	98.6	102.6	101.2	99.3	100.6	100.25	0.996520875	0.792378
Q02978	SLC25A11	Mitochondrial 2-oxoglutarate/malate carrier p	13	101.8	99.3	100.7	99.7	100.55	100.2	0.996519145	0.792402
A0A087WT2	DCAF13	DDB1- and CUL4-associated factor 13 OS=H	16	101.6	99.5	101	99.4	100.55	100.2	0.996519145	0.792402
A2RRP1	NBAS	Neuroblastoma-amplified sequence OS=Hom	19	99.1	101.2	101.7	97.9	100.15	99.8	0.996505242	0.792594
P22830	FECH	Ferrochelatase, mitochondrial OS=Homo sapi	5	102.9	97.2	96.7	102.7	100.05	99.7	0.996501749	0.792642
A8K7F7		cDNA FLJ76913, highly similar to Homo sap	2	102.5	97.6	97.3	102.1	100.05	99.7	0.996501749	0.792642
O43681	ASNA1	ATPase ASNA1 OS=Homo sapiens GN=ASN	11	98.9	100.9	97.7	101.4	99.9	99.55	0.996496496	0.792715
Q8IY17	PNPLA6	Neuropathy target esterase OS=Homo sapiens	11	102.4	97.4	99.6	99.5	99.9	99.55	0.996496496	0.792715
Q9BQS8	FYCO1	FYVE and coiled-coil domain-containing prot	13	102.3	97.3	98	100.9	99.8	99.45	0.996492986	0.792763
P17010	ZFX	Zinc finger X-chromosomal protein OS=Hom	3	102	96.8	99.2	98.9	99.4	99.05	0.996478873	0.792959
Q96T37	RBM15	Putative RNA-binding protein 15 OS=Homo s	12	98.4	100.4	99.2	98.9	99.4	99.05	0.996478873	0.792959
Q9H8Y5	ANKZF1	Ankyrin repeat and zinc finger domain-contai	3	99.3	99.3	100.2	97.7	99.3	98.95	0.996475327	0.793007
A8K9B2		cDNA FLJ76725, highly similar to Homo sap	12	100.8	97.1	97.3	99.9	98.95	98.6	0.99646286	0.79318
P49711	CTCF	Transcriptional repressor CTCF OS=Homo sa	10	98.5	98.5	96.9	99.4	98.5	98.15	0.996446701	0.793403
P50336	PPOX	Protoporphyrinogen oxidase OS=Homo sapie	9	93.9	94.6	99.2	88.6	94.25	93.9	0.996286472	0.79562
Q9NPD3	EXOSC4	Exosome complex component RRP41 OS=H	7	92	92.6	89.5	94.4	92.3	91.95	0.996208017	0.796706
E9PFW3	AP2M1	AP-2 complex subunit mu OS=Homo sapiens	22	102.4	101.4	103	100	101.9	101.5	0.996074583	0.798555
Q9UKV5	AMFR	E3 ubiquitin-protein ligase AMFR OS=Homo	9	103.4	100.2	99.6	103.2	101.8	101.4	0.996070727	0.798608
Q9HCN4	GPN1	GPN-loop GTPase 1 OS=Homo sapiens GN=	2	102.5	100.5	96.7	105.5	101.5	101.1	0.996059113	0.798769
A0A087X1V	SHFM1	26S proteasome complex subunit DSS1 OS=I	1	100.8	101.6	98.2	103.4	101.2	100.8	0.996047431	0.798931
A0A0C4DGG	CHD4	Chromodomain-helicase-DNA-binding protei	37	101.8	100.1	102.2	98.9	100.95	100.55	0.996037642	0.799066
Q96DH6	MSI2	RNA-binding protein Musashi homolog 2 OS	1	99.9	101.8	103	97.9	100.85	100.45	0.996033713	0.799121
Q92733	PRCC	Proline-rich protein PRCC OS=Homo sapiens	6	101.7	99.3	98.4	101.8	100.5	100.1	0.9960199	0.799312
P61225	RAP2B	Ras-related protein Rap-2b OS=Homo sapiens	3	106.6	94.3	100.5	99.6	100.45	100.05	0.996017919	0.79934
Q4VC44	FLYWCH1	FLYWCH-type zinc finger-containing protein	1	104.9	96	102	98.1	100.45	100.05	0.996017919	0.79934
B5MBW9	CHCHD10	Coiled-coil-helix-coiled-coil-helix domain-co	1	97.4	103.3	96.2	103.7	100.35	99.95	0.996013951	0.799395
P54886	ALDH18A1	Delta-1-pyrroline-5-carboxylate synthase OS=	31	100.8	99.5	101.6	97.9	100.15	99.75	0.996005991	0.799505

E5RJR5	SKP1	S-phase kinase-associated protein 1 OS=Homo	6	100.3	99.7	98.7	100.5	100	99.6	0.996	0.799588
A8K7A2		cDNA FLJ78084, highly similar to Homo sap	5	101.8	98	99.1	99.9	99.9	99.5	0.995995996	0.799644
Q9NY61	AATF	Protein AATF OS=Homo sapiens GN=AATF	17	102.8	96.9	99.3	99.6	99.85	99.45	0.995993991	0.799672
Q12797	ASPH	Aspartyl/asparaginyl beta-hydroxylase OS=H	24	98.6	100.7	98.6	99.9	99.65	99.25	0.995985951	0.799783
P55011	SLC12A2	Solute carrier family 12 member 2 OS=Homo	14	101.7	97.5	99	99.4	99.6	99.2	0.995983936	0.799811
B2R673		Dihydrolipoamide acetyltransferase componen	10	100.8	97.9	100.2	97.7	99.35	98.95	0.99597383	0.799951
Q6IAQ2	SDHC	SDHC protein OS=Homo sapiens GN=SDHC	2	100.1	98	97.9	99.4	99.05	98.65	0.995961636	0.80012
A0A024R006	FAM8A1	Family with sequence similarity 8, member A	1	100.3	95.6	105.1	90	97.95	97.55	0.995916284	0.800749
B0LPF3	GRB2	Growth factor receptor-bound protein 2 OS=H	13	96.8	98.9	96.8	98.1	97.85	97.45	0.99591211	0.800806
Q15274	QPRT	Nicotinate-nucleotide pyrophosphorylase [car	4	96.7	97.3	95.2	98	97	96.6	0.995876289	0.801303
Q13686	ALKBH1	DNA demethylase ALKBH1 OS=Homo sapie	3	95.6	94	93	95.8	94.8	94.4	0.995780591	0.802632
Q14964	RAB39A	Ras-related protein Rab-39A OS=Homo sapie	1	105.2	99.4	100.3	103.4	102.3	101.85	0.995601173	0.805123
O43909	EXTL3	Exostosin-like 3 OS=Homo sapiens GN=EXT	2	104.1	100.1	94.3	109	102.1	101.65	0.995592556	0.805243
P55795	HNRNPH2	Heterogeneous nuclear ribonucleoprotein H2	9	102.9	100.1	100.1	102	101.5	101.05	0.995566502	0.805605
P61964	WDR5	WD repeat-containing protein 5 OS=Homo sa	7	102.1	100.7	99.8	102.1	101.4	100.95	0.99556213	0.805666
A8K3B6		Tyrosine-protein kinase OS=Homo sapiens PI	12	101	100.7	98.5	102.3	100.85	100.4	0.995537928	0.806002
P49207	RPL34	60S ribosomal protein L34 OS=Homo sapiens	6	98.6	103.1	101.1	99.7	100.85	100.4	0.995537928	0.806002
Q15019	SEPT2	Septin-2 OS=Homo sapiens GN=SEPT2 PE=	14	101.2	100	99.6	100.7	100.6	100.15	0.995526839	0.806156
A0A024R6Y3	hCG_2027080	Peptide deformylase OS=Homo sapiens GN=l	3	107.4	93.3	99.2	100.6	100.35	99.9	0.995515695	0.806311
Q8NBL1	POGLUT1	Protein O-glucosyltransferase 1 OS=Homo sa	6	102.7	97.5	99.4	99.9	100.1	99.65	0.995504496	0.806467
A0A024R1Q8	RPL23	Ribosomal protein L23, isoform CRA_b OS=	11	99.9	100.2	98.4	100.8	100.05	99.6	0.995502249	0.806498
Q9NYC9	DNAH9	Dynein heavy chain 9, axonemal OS=Homo s	1	101.3	98.5	97.4	101.5	99.9	99.45	0.995495495	0.806592
B2R4Q7		Mitochondrial pyruvate carrier OS=Homo sap	4	98.6	101	98.5	100.2	99.8	99.35	0.995490982	0.806655
Q13153	PAK1	Serine/threonine-protein kinase PAK 1 OS=H	1	102.8	96.6	101.2	97.3	99.7	99.25	0.995486459	0.806718
A8K6V6		N-acetylglucosamine-6-sulfatase OS=Homo s	11	101.7	97.6	101.1	97.3	99.65	99.2	0.995484195	0.806749
Q969T4	UBE2E3	Ubiquitin-conjugating enzyme E2 E3 OS=Ho	1	98.3	100.8	97.7	100.5	99.55	99.1	0.995479658	0.806812
B4E1J8		cDNA FLJ56285, highly similar to ADP-ribos	3	115	96.9	111.7	99.2	105.95	105.45	0.995280793	0.809578
H7C0C1		Uncharacterized protein (Fragment) OS=Hom	2	98.4	91	89.2	99.3	94.7	94.25	0.995248152	0.810033
Q99541	PLIN2	Perilipin-2 OS=Homo sapiens GN=PLIN2 PE	7	110.7	98	104.9	102.8	104.35	103.85	0.995208433	0.810585
Q9BZF1	OSBPL8	Oxysterol-binding protein-related protein 8 O	17	103.6	101.4	100.5	103.5	102.5	102	0.995121951	0.81179
A0A090N7Y2	ABCF2	ATP-binding cassette, sub-family F (GCN20)	24	103.5	101.4	102	101.9	102.45	101.95	0.995119571	0.811823
B7Z4C8	RPL31	60S ribosomal protein L31 OS=Homo sapiens	6	98.3	106.6	102.3	101.6	102.45	101.95	0.995119571	0.811823
A8K517	RPS23	Ribosomal protein S23, isoform CRA_a OS=l	8	98.6	105.1	100.5	102.2	101.85	101.35	0.99509082	0.812223
A0A024R9K4	FYTTD1	Forty-two-three domain containing 1, isoform	4	99.6	103.8	102.6	99.8	101.7	101.2	0.995083579	0.812324
Q9H9L3	ISG20L2	Interferon-stimulated 20 kDa exonuclease-like	5	103.6	99.6	102.4	99.8	101.6	101.1	0.99507874	0.812392
Q13619	CUL4A	Cullin-4A OS=Homo sapiens GN=CUL4A PI	4	98.4	104.6	101.6	100.4	101.5	101	0.995073892	0.812459
P28074	PSMB5	Proteasome subunit beta type-5 OS=Homo sa	13	99.6	102.8	100.3	101.1	101.2	100.7	0.995059289	0.812662
Q9BV40	VAMP8	Vesicle-associated membrane protein 8 OS=H	5	102.7	99.7	103.1	98.3	101.2	100.7	0.995059289	0.812662
Q53Y03	COX4NB	COX4 neighbor, isoform CRA_a OS=Homo s	7	103.2	98.8	100.4	100.6	101	100.5	0.995049505	0.812799
Q13838	DDX39B	Spliceosome RNA helicase DDX39B OS=Ho	10	101.6	100.4	101.1	99.9	101	100.5	0.995049505	0.812799
Q07960	ARHGAP1	Rho GTPase-activating protein 1 OS=Homo s	10	97.6	104.4	98.9	102.1	101	100.5	0.995049505	0.812799
P63272	SUPT4H1	Transcription elongation factor SPT4 OS=Ho	2	101.4	100.1	97.3	103.2	100.75	100.25	0.995037221	0.81297
Q9BRT6	LLPH	Protein LLP homolog OS=Homo sapiens GN=	3	98.2	103	101	99.2	100.6	100.1	0.995029821	0.813073
A8KAM9		Peptidyl-prolyl cis-trans isomerase E OS=Ho	6	97.2	103.9	98.6	101.5	100.55	100.05	0.99502735	0.813108
O96005	CLPTM1	Cleft lip and palate transmembrane protein 1	11	104.3	96.6	102.1	97.8	100.45	99.95	0.995022399	0.813176
A0A024RDZ2	CDC16	CDC16 cell division cycle 16 homolog (S. ce	4	99.5	101.4	96.7	103.2	100.45	99.95	0.995022399	0.813176
P46199	MTIF2	Translation initiation factor IF-2, mitochondri	8	100.1	99.9	97.6	101.4	100	99.5	0.995	0.813489
O15511	ARPC5	Actin-related protein 2/3 complex subunit 5 C	7	98.9	101.1	99.5	99.5	100	99.5	0.995	0.813489
Q7Z6E9	RBBP6	E3 ubiquitin-protein ligase RBBP6 OS=Hom	11	99.7	100.2	98.9	100	99.95	99.45	0.994997499	0.813524
P35251	RFC1	Replication factor C subunit 1 OS=Homo sapi	8	99.9	99.9	95.8	103	99.9	99.4	0.994994995	0.813559
Q96NC0	ZMAT2	Zinc finger matrin-type protein 2 OS=Homo s	3	96.6	103	102.1	96.5	99.8	99.3	0.99498998	0.813628
Q9NRW7	VPS45	Vacuolar protein sorting-associated protein 45	5	100.2	99.1	99.3	99	99.65	99.15	0.994982439	0.813734
B2R694		Terpene cyclase/mutase family member OS=I	10	100.6	97.1	98.1	98.6	98.85	98.35	0.994941831	0.8143
Q9HC52	CBX8	Chromobox protein homolog 8 OS=Homo sap	10	98.1	98.8	98.4	97.5	98.45	97.95	0.99492128	0.814586
A8K1A2		cDNA FLJ77265, highly similar to Homo sap	1	99.7	96.8	96.2	99.3	98.25	97.75	0.994910941	0.81473
A0A024QZT9	NQO2	NAD(P)H dehydrogenase, quinone 2, isoform	6	100.1	95.1	93.1	101.1	97.6	97.1	0.994877049	0.815203
P60763	RAC3	Ras-related C3 botulinum toxin substrate 3 O	2	99	94.4	94.1	98.3	96.7	96.2	0.994829369	0.815868
A0A1B0GVA	RYR3	Ryanodine receptor 3 (Fragment) OS=Homo s	1	108.7	103.3	107.8	103.1	106	105.45	0.994811321	0.81612
G3XAN8	TIMM8B	Mitochondrial import inner membrane translo	5	97	95	93.8	97.2	96	95.5	0.994791667	0.816394
Q969Q5	RAB24	Ras-related protein Rab-24 OS=Homo sapiens	4	105.7	99.4	105.2	98.8	102.55	102	0.994636763	0.818556
Q86SG6	NEK8	Serine/threonine-protein kinase Nek8 OS=Ho	1	109.3	95.7	104.6	99.3	102.5	101.95	0.994634146	0.818593
A7YA96	GGCX	Gamma-glutamyl carboxylase OS=Homo sapi	3	104.6	99.8	100.9	102.4	102.2	101.65	0.994618395	0.818813
P62906	RPL10A	60S ribosomal protein L10a OS=Homo sapier	12	99.5	104	101.9	100.5	101.75	101.2	0.994594595	0.819146
A0A087WYF	APOOL	MICOS complex subunit OS=Homo sapiens	4	104.7	98.6	100.4	101.8	101.65	101.1	0.994589277	0.81922
Q05519	SRSF11	Serine/arginine-rich splicing factor 11 OS=Ho	7	98.2	103.9	99.7	101.3	101.05	100.5	0.99455715	0.819669
A0A087WVA	SELT	Selenoprotein T OS=Homo sapiens GN=SELT	2	112.2	89.9	102.6	98.4	101.05	100.5	0.99455715	0.819669
Q12981	BNIP1	Vesicle transport protein SEC20 OS=Homo sa	6	103	98.5	100.7	99.7	100.75	100.2	0.994540943	0.819895
O75643	SNRNP200	U5 small nuclear ribonucleoprotein 200 kDa h	82	101.2	100	101.8	98.3	100.6	100.05	0.994532803	0.820008
B3KML1		cDNA FLJ11308 fis, clone PLACE1010074,	12	100	101.1	99.7	100.3	100.55	100	0.994530085	0.820047
L8B4J3	UbC	Ubiquitin C OS=Homo sapiens GN=UbC PE=	1	103.8	97	102.4	97.3	100.4	99.85	0.994521912	0.820161
O15143	ARPC1B	Actin-related protein 2/3 complex subunit 1B	13	97.3	101.4	97.1	100.5	99.35	98.8	0.994464016	0.820969
P51648	ALDH3A2	Fatty aldehyde dehydrogenase OS=Homo sap	15	99.3	98.6	99.2	97.6	98.95	98.4	0.994441637	0.821283
Q8TDD1	DDX54	ATP-dependent RNA helicase DDX54 OS=H	23	99.1	97.8	99.4	96.4	98.45	97.9	0.994413408	0.821677
Q9Y6H1	CHCHD2	Coiled-coil-helix-coiled-coil-helix domain-co	2	98.7	97.5	97.7	97.4	98.1	97.55	0.994393476	0.821956
O94842	TOX4	TOX high mobility group box family member	8	99.7	96.2	95.3	99.5	97.95	97.4	0.99438489	0.822076
Q15785	TOMM34	Mitochondrial import receptor subunit TOM3	8	100.2	95.5	95	99.6	97.85	97.3	0.994379152	0.822156
F1T0A5	PRPF31	PRP31 pre-mRNA processing factor 31 homo	11	98.6	97	97.7	96.8	97.8	97.25	0.994376278	0.822196
A0A024RDA	EXOC1	Exocyst complex component 1, isoform CRA_	4	96.1	98.6	96.5	97.1	97.35	96.8	0.994350282	0.82256
B2R4A2		Cytochrome b-c1 complex subunit 7 OS=Hon	1	92.8	97.1	93.1	95.7	94.95	94.4	0.994207478	0.824557
P17812	CTPS1	CTP synthase 1 OS=Homo sapiens GN=CTP	17	107.4	98.5	101.5	103.2	102.95	102.35	0.994171928	0.825055
Q9Y4B6	VPRBP	Protein VPRBP OS=Homo sapiens GN=VPR	8	100.2	105.7	101.5	103.2	102.95	102.35	0.994171928	0.825055
A6NHR9	SMCHD1	Structural maintenance of chromosomes flexil	46	102.3	102.6	103.1	100.6	102.45	101.85	0.994143485	0.825453
A0A024RAC	LUZP1	Leucine zipper protein 1, isoform CRA_a OS	4	105.5	99.3	104.9	98.7	102.4	101.8	0.994140625	0.825493
Q96J02	ITCH	E3 ubiquitin-protein ligase Itchy homolog OS	14	101.8	101.1	101.1	100.6	101.45	100.85	0.994085757	0.826262
Q9UKN8	GTF3C4	General transcription factor 3C polypeptide 4	13	102.4	100.5	99.8	101.9	101.45	100.85	0.994085757	0.826262
A0A024R1V4	RPL27	60S ribosomal protein L27 OS=Homo sapiens	7	99.6	103	101.5	99.9	101.3	100.7	0.994076999	0.826384
Q9GZZ1	NAA50	N-alpha-acetyltransferase 50 OS=Homo sapie	11	100.4	102	100.7	100.5	101.2	100.6	0.994071146	0.826466
Q9BTC0	DIDO1	Death-inducer obliterator 1 OS=Homo sapien	20	101.6	99.1	99.1	100.4	100.35	99.75	0.994020927	0.827169
A0A024R0P9	TOMM40	Translocase of outer mitochondrial membrane	11	101.1	99.2	98.5	100.6	100.15	99.55	0.994008987	0.827337
F5GX99	CLPB	Caseinolytic peptidase B protein homolog OS	1	105.2	95.1	97	102.1	100.15	99.55	0.994008987	0.827337
Q969P0	IGSF8	Immunoglobulin superfamily member 8 OS=I	2	103.2	96.9	99.2	99.7	100.05	99.45	0.994002999	0.827421
Q8TF01	PNISR	Arginine/serine-rich protein PNISR OS=Hom	1	103.5	96.3	99.8	98.8	99.9	99.3	0.993993994	0.827547
Q96GQ7	DDX27	Probable ATP-dependent RNA helicase DDX	29	101	98.7	100.4	98.1	99.85	99.25	0.993990986	0.827589
Q15022	SUZ12	Polycomb protein SUZ12 OS=Homo sapiens	9	97.2	102.2	97.5	100.7	99.7	99.1	0.993981946	0.827715
P40617	ARL4A	ADP-ribosylation factor-like protein 4A OS=l	1	100.7	98.6	92.2	105.9	99.65	99.05	0.993978926	0.827758
A0A140VJW5		Testicular tissue protein Li 192 OS=Homo sa	21	97.8	101.1	99.6	98.1	99.45	98.85	0.993966817	0.827928
Q6NUK7	LYN	Tyrosine-protein kinase (Fragment) OS=Hom	1	104.4	94.5	99	98.7	99.45	98.85	0.993966817	0.827928
A2RUM7	RPL5	Ribosomal protein L5 OS=Homo sapiens GN	2	98.2	100.4	97.4	100	99.3	98.7	0.993957704	0.828055
P53611	RABGGTB	Geranylgeranyl transferase type-2 subunit bet	2	101.8	96.3	95.5	101.4	99.05	98.45	0.993942453	0.828269
P39687	ANP32A	Acidic leucine-rich nuclear phosphoprotein 32	4	96.3	101.3	96.1	100.3	98.8	98.2	0.993927126	0.828484
Q86VE0	MYPOP	Myb-related transcription factor, partner of pr	1	111.8	85.4	90.2	105.8	98.6	98	0.993914807	0.828657
O43933	PEX1	Peroxisome biogenesis factor 1 OS=Homo sa	1	101	95.1	101.9	93	98.05	97.45	0.993880673	0.829135
Q15773	MLF2	Myeloid leukemia factor 2 OS=Homo sapiens	3	99.1	96.7	98	96.6	97.9	97.3	0.993871297	0.829267
A0A024R0F1	TTF2	Transcription termination factor, RNA polym	3	100.1	95.6	95.6	98.9	97.85	97.25	0.993868166	0.82931
Q7LBR1	CHMP1B	Charged multivesicular body protein 1b OS=I	1	100.5	94.8	94.4	99.7	97.65	97.05	0.993855607	0.829486
A0A024RAE5	MGC10993	Uncharacterized protein OS=Homo sapiens G	2	100.9	93.9	96.3	97.3	97.4	96.8	0.993839836	0.829707
O75071	EFCAB14	EF-hand calcium-binding domain-containing	1	89.6	88.8	84.5	92.8	89.2	88.65	0.993834081	0.829788
Q14BN4	SLMAP	Sarcolemmal membrane-associated protein O	7	99.2	93.6	91.4	100.2	96.4	95.8	0.993775934	0.830603
Q59GX2		Solute carrier family 2 (Facilitated glucose tra	7	102.9	100.9	100.1	102.4	101.9	101.25	0.993621197	0.832775
Q8N9N5	BANP	Protein BANP OS=Homo sapiens GN=BANP	1	102.5	100.8	99.6	102.4	101.65	101	0.993605509	0.832995
A8K885		Sorting nexin OS=Homo sapiens PE=2 SV=1	14	102.5	100.7	103.3	98.6	101.6	100.95	0.993602362	0.833039
C9JC84	FGG	Fibrinogen gamma chain OS=Homo sapiens	1	105.4	113.3	107.3	110	109.35	108.65	0.993598537	0.833093
B3KM47		cDNA FLJ10273 fis, clone HEMBB1001137,	12	101.5	101.2	101.7	99.7	101.35	100.7	0.993586581	0.83326
P40123	CAP2	Adenylyl cyclase-associated protein 2 OS=Ho	7	100.3	102.3	98.2	103.1	101.3	100.65	0.993583416	0.833304
Q6P4F7	ARHGAP11A	Rho GTPase-activating protein 11A OS=Hom	1	106.3	96	94.3	106.7	101.15	100.5	0.9935739	0.833438
O00541	PES1	Pescadillo homolog OS=Homo sapiens GN=F	23	99.7	102.2	102.6	98	100.95	100.3	0.993561169	0.833617
A0A0S2Z5J4	AP3B1	Adaptor-related protein complex 3 beta 1 sub	15	101.4	99.9	100.1	99.9	100.65	100	0.993541977	0.833887
Q969S9	GFM2	Ribosome-releasing factor 2, mitochondrial O	7	102.4	98.5	97	102.6	100.45	99.8	0.993529119	0.834067
B4DHQ3		Phosphoserine aminotransferase OS=Homo sa	16	99.3	101.5	100.4	99.1	100.4	99.75	0.993525896	0.834112
P46937	YAP1	Transcriptional coactivator YAP1 OS=Homo	4	98.5	101.7	98.4	100.5	100.1	99.45	0.993506494	0.834385
A0A024RAL3	ZFYVE16	Zinc finger, FYVE domain containing 16, iso	1	98.2	101.6	88.6	109.9	99.9	99.25	0.993493493	0.834567
Q9BU61	NDUFAF3	NADH dehydrogenase [ubiquinone] 1 alpha s	6	100.4	98.9	102	96	99.65	99	0.99347717	0.834796

Q8WYP5	AHCTF1	Protein ELYS OS=Homo sapiens GN=AHCT	32	100.6	98.3	98.1	99.5	99.45	98.8	0.993464052	0.834981
Q7Z7K6	CENPV	Centromere protein V OS=Homo sapiens GN	7	97	101.9	98.4	99.2	99.45	98.8	0.993464052	0.834981
O75319	DUSP11	RNA/RNP complex-1-interacting phosphatase	3	98.8	100.1	99.1	98.5	99.45	98.8	0.993464052	0.834981
Q6PUJ7	HEL-215	Epididymis luminal protein 215 OS=Homo sa	17	100	98.8	99.3	98.2	99.4	98.75	0.993460765	0.835027
A0A0S2Z4R4	HGS	Hepatocyte growth factor-regulated tyrosine k	9	100.6	98.1	98.6	98.8	99.35	98.7	0.993457474	0.835073
M0QWZ7	SARS2	Serine--tRNA ligase, mitochondrial OS=Hom	18	101.1	97.4	100.1	97.1	99.25	98.6	0.993450882	0.835166
O14737	PDCD5	Programmed cell death protein 5 OS=Homo s	7	98.1	100.1	97.2	99.7	99.1	98.45	0.993440969	0.835305
Q3MIR4	TMEM30B	Cell cycle control protein 50B OS=Homo sap	2	114.2	99.2	99.8	112.2	106.7	106	0.99343955	0.835325
P47985	UQCRFS1	Cytochrome b-c1 complex subunit Rieske, mi	12	100.2	97.8	99.6	97.1	99	98.35	0.993434343	0.835398
O14980	XPO1	Exportin-1 OS=Homo sapiens GN=XPO1 PE	27	99.2	98.6	99.1	97.4	98.9	98.25	0.993427705	0.835491
Q59G13		Syntaxin 16 isoform a variant (Fragment) OS	2	97.3	100.1	99.2	96.9	98.7	98.05	0.993414387	0.835678
A0A024R2N6	NKTR	Natural killer-tumor recognition sequence, iso	1	93.5	102.7	95.8	99.1	98.1	97.45	0.993374108	0.836244
Q9BUL8	PDCD10	Programmed cell death protein 10 OS=Homo	4	100.7	95.2	98	96.6	97.95	97.3	0.993363961	0.836387
Q9P0S3	ORMDL1	ORM1-like protein 1 OS=Homo sapiens GN=	1	103.5	104.1	103.2	103	103.8	103.1	0.993256262	0.8379
J3QRU1	YES1	Tyrosine-protein kinase OS=Homo sapiens G	11	102.6	103.9	103.8	101.3	103.25	102.55	0.993220339	0.838406
Q92968	PEX13	Peroxisomal membrane protein PEX13 OS=H	5	105.8	99.8	102.1	102.1	102.8	102.1	0.993190661	0.838823
Q6ZRV2	FAM83H	Protein FAM83H OS=Homo sapiens GN=FA	5	103.6	101.5	94.9	108.8	102.55	101.85	0.993174061	0.839056
P15056	BRAF	Serine/threonine-protein kinase B-raf OS=Ho	1	100.1	102	96.4	104.3	101.05	100.35	0.993072736	0.840482
B4DKM0		cDNA FLJ51883, highly similar to Mitochond	9	100.6	101.4	102.1	98.5	101	100.3	0.993069307	0.84053
Q9Y5T5	USP16	Ubiquitin carboxyl-terminal hydrolase 16 OS	5	103	98.9	98.3	102.2	100.95	100.25	0.993065874	0.840578
O14972	DSCR3	Down syndrome critical region protein 3 OS=	2	102.1	99.5	100.6	99.6	100.8	100.1	0.993055556	0.840723
Q92560	BAP1	Ubiquitin carboxyl-terminal hydrolase BAP1	1	84.9	116.6	99.5	100.6	100.75	100.05	0.993052109	0.840772
A0A024R8D4	MRPS2	Mitochondrial ribosomal protein S2, isoform	9	103.2	98.1	102.3	97.6	100.65	99.95	0.993045206	0.840869
A0A0G2JK23	BAG6	Large proline-rich protein BAG6 OS=Homo s	10	101.2	99.7	100.5	99	100.45	99.75	0.993031359	0.841064
A0A024RB85	PA2G4	Proliferation-associated 2G4, 38kDa, isoform	21	100.1	100.5	99.2	100	100.3	99.6	0.993020937	0.841211
Q96DP0		cDNA FLJ31479 fis, clone NT2NE2001634,	7	98.5	102.1	98.7	100.5	100.3	99.6	0.993020937	0.841211
Q68DK7	MSL1	Male-specific lethal 1 homolog OS=Homo sa	1	103.4	96.9	95.9	103	100.15	99.45	0.993010484	0.841358
A0A140VK53		Testicular secretory protein Li 53 OS=Homo s	34	101.3	98.9	100.3	98.5	100.1	99.4	0.993006993	0.841407
A0A0U1RQQ	SCYL2	SCY1-like protein 2 OS=Homo sapiens GN=	5	101.3	97.9	98.9	98.9	99.6	98.9	0.992971888	0.841901
A8K6G9		cDNA FLJ76871, highly similar to Homo sap	12	98	101.1	98.2	99.5	99.55	98.85	0.992968358	0.841951
Q9NUJ1	ABHD10	Mycophenolic acid acyl-glucuronide esterase,	9	100.1	98.9	97.4	100.2	99.5	98.8	0.992964824	0.842001
P82932	MRPS6	28S ribosomal protein S6, mitochondrial OS=	6	99.8	99.1	99.6	97.9	99.45	98.75	0.992961287	0.84205
Q9Y584	TIMM22	Mitochondrial import inner membrane translo	1	98.1	100.2	98.4	98.5	99.15	98.45	0.99293999	0.84235
Q6P087	RPUSD3	RNA pseudouridylate synthase domain-contai	3	101.9	96.2	97.5	99.2	99.05	98.35	0.992932862	0.842451
Q9NRN7	AASDHPPT	L-aminoadipate-semialdehyde dehydrogenase	3	95.4	102.6	95.6	101	99	98.3	0.992929293	0.842501
Q9H6T3	RPAP3	RNA polymerase II-associated protein 3 OS=	8	97.8	100.1	96.6	99.9	98.95	98.25	0.99292572	0.842551
Q8TAD4	SLC30A5	Zinc transporter 5 OS=Homo sapiens GN=SL	3	102.3	94.8	96.6	99.1	98.55	97.85	0.992897007	0.842955
B2RB38		cDNA, FLJ95288, highly similar to Homo sap	3	101.5	106.6	101.3	105.3	104.05	103.3	0.992791927	0.844435
O15014	ZNF609	Zinc finger protein 609 OS=Homo sapiens GN	1	101.7	106	104.8	101.4	103.85	103.1	0.992778045	0.844631
B4DUA7	MED29	Intersex-like (Drosophila) OS=Homo sapiens	2	105.7	100	100.2	104	102.85	102.1	0.992707827	0.845621
A0A024R6A0	ARG2	Arginase OS=Homo sapiens GN=ARG2 PE=	6	100.9	90.2	93.1	96.6	95.55	94.85	0.992673993	0.846098
Q9Y6N7	ROBO1	Roundabout homolog 1 OS=Homo sapiens G	5	103.5	100.7	97.1	105.6	102.1	101.35	0.992654261	0.846376
B4DF33		cDNA FLJ57960 OS=Homo sapiens PE=2 SV	2	103	101	98.8	103.7	102	101.25	0.992647059	0.846478
O95453	PARN	Poly(A)-specific ribonuclease PARN OS=Ho	14	104.2	99.6	101.9	100.4	101.9	101.15	0.992639843	0.846579
P30050	RPL12	60S ribosomal protein L12 OS=Homo sapien	9	101.8	101.6	102.5	99.4	101.7	100.95	0.992625369	0.846783
B3KSH1	EIF3F	Eukaryotic translation initiation factor 3 subu	10	102	101.3	102.5	99.3	101.65	100.9	0.992621741	0.846835
A0A024R355	THEX1	Three prime histone mRNA exonuclease 1, is	11	98.5	104.2	100.2	101	101.35	100.6	0.992599901	0.847142
P13667	PDIA4	Protein disulfide-isomerase A4 OS=Homo sa	40	101.7	100.9	101.4	99.7	101.3	100.55	0.992596249	0.847194
Q9BW19	KIFC1	Kinesin-like protein KIFC1 OS=Homo sapien	6	98.3	103.9	102.5	98.2	101.1	100.35	0.992581602	0.8474
P53618	COPB1	Coatomer subunit beta OS=Homo sapiens GN	31	101	101.1	99.7	100.9	101.05	100.3	0.992577932	0.847453
Q6P1L8	MRPL14	39S ribosomal protein L14, mitochondrial OS	4	105.7	96	96.4	103.8	100.85	100.1	0.992563213	0.84766
Q9NUQ2	AGPAT5	1-acyl-sn-glycerol-3-phosphate acyltransferas	6	98.6	102.7	102.5	97.3	100.65	99.9	0.992548435	0.847869
B3KM21	FAM36A	Family with sequence similarity 36, member	2	101.4	99.7	100.8	98.8	100.55	99.8	0.992541024	0.847973
P41223	BUD31	Protein BUD31 homolog OS=Homo sapiens	5	102.6	98.3	100.2	99.2	100.45	99.7	0.992533599	0.848077
Q9H9Y6	POLR1B	DNA-directed RNA polymerase I subunit RP	18	101.7	99.2	99.8	99.6	100.45	99.7	0.992533599	0.848077
Q5T4S7	UBR4	E3 ubiquitin-protein ligase UBR4 OS=Homo	31	99.6	101.1	98	101.2	100.35	99.6	0.992526158	0.848182
Q8TBQ9	TMEM167A	Protein kish-A OS=Homo sapiens GN=TMEM	1	101.5	98.3	100.3	98	99.9	99.15	0.992492492	0.848657
Q9BV38	WDR18	WD repeat-containing protein 18 OS=Homo s	12	101.3	98.5	101.5	96.8	99.9	99.15	0.992492492	0.848657
Q9UK41	VPS28	Vacuolar protein sorting-associated protein 2	5	102.1	96.7	101.2	96.1	99.4	98.65	0.992454728	0.849191
O00461	GOLIM4	Golgi integral membrane protein 4 OS=Homo	9	98.9	99.9	98.4	98.9	99.4	98.65	0.992454728	0.849191
G8XV63		Prostate cancer upregulated protein 1 OS=Ho	4	96.7	101.5	93.6	103.1	99.1	98.35	0.992431887	0.849513
P08237	PFKM	ATP-dependent 6-phosphofructokinase, musc	19	98.9	99	98	98.4	98.95	98.2	0.992420414	0.849674
Q8N567	ZCCHC9	Zinc finger CCHC domain-containing protein	4	96.1	99	90.7	102.9	97.55	96.8	0.992311635	0.85121
A0A0A0MQX	MBNL1	Muscleblind-like protein 1 OS=Homo sapiens	8	99.8	107.5	104.6	101.1	103.65	102.85	0.992281717	0.851632
Q59F99		Staufen isoform b variant (Fragment) OS=Ho	10	106.1	99.6	101.5	102.6	102.85	102.05	0.992221682	0.852481
B3KP18		cDNA FLJ30946 fis, clone FEBRA2007622,	5	104	100.8	101.9	101.3	102.4	101.6	0.9921875	0.852963
P40222	TXLNA	Alpha-taxilin OS=Homo sapiens GN=TXLN	15	102	102.8	102.2	101	102.4	101.6	0.9921875	0.852963
A0A024R0V4	VASP	Vasodilator-stimulated phosphoprotein isofor	9	99.8	104.2	99.6	102.8	102	101.2	0.992156863	0.853396
P20042	EIF2S2	Eukaryotic translation initiation factor 2 subu	15	103.1	100.8	101.6	100.7	101.95	101.15	0.992153016	0.853451
Q9Y3B8	REXO2	Oligoribonuclease, mitochondrial OS=Homo	3	103.7	100.2	101.2	101.1	101.95	101.15	0.992153016	0.853451
Q92900	UPF1	Regulator of nonsense transcripts 1 OS=Hom	29	100.2	102.5	100.3	100.8	101.35	100.55	0.992106561	0.854107
O43865	AHCYL1	Adenosylhomocysteinase 2 OS=Homo sapien	7	103.3	99.3	99.1	101.9	101.3	100.5	0.992102665	0.854162
Q5TH30	NDRG3	NDRG family member 3, isoform CRA_c OS	5	100.9	101.4	101.9	98.8	101.15	100.35	0.992090954	0.854328
O43715	TRIAP1	TP53-regulated inhibitor of apoptosis 1 OS=	1	101.4	112.7	104	108.4	107.05	106.2	0.992059785	0.854768
J3QQJ0	SAP30BP	SAP30-binding protein (Fragment) OS=Hom	5	104.9	96.6	102.5	97.4	100.75	99.95	0.992059553	0.854772
V9HW41	HEL-S-71	Epididymis secretory protein Li 71 OS=Hom	8	99.6	101.9	101.4	98.5	100.75	99.95	0.992059553	0.854772
B4DSS8		cDNA FLJ55936, highly similar to Polypyrim	3	105.1	95.8	98	101.3	100.45	99.65	0.992035839	0.855106
Q12849	GRSF1	G-rich sequence factor 1 OS=Homo sapiens	22	101.3	98.3	97.6	100.4	99.8	99	0.991983968	0.85584
O75844	ZMPSTE24	CAAX prenyl protease 1 homolog OS=Homo	8	100.1	99.5	99.7	98.3	99.8	99	0.991983968	0.85584
B2RA15		Inhibitor of growth protein OS=Homo sapiens	1	105.9	91.9	90.1	106.1	98.9	98.1	0.991911021	0.856872
Q96RU2	USP28	Ubiquitin carboxyl-terminal hydrolase 28 OS	9	99.1	98.1	97.4	98.2	98.6	97.8	0.99188641	0.857219
Q8TEW0	PARD3	Partitioning defective 3 homolog OS=Homo s	4	100.9	95.5	95.2	99.6	98.2	97.4	0.99185336	0.857687
P45954	ACADSB	Short/branched chain specific acyl-CoA dehy	9	97	99.2	97	97.6	98.1	97.3	0.991845056	0.857804
A7LNJ1	SLC20A1	Phosphate transporter OS=Homo sapiens GN	5	108.4	97.9	101.5	103.1	103.15	102.3	0.991759573	0.859014
Q07954	LRP1	Prolow-density lipoprotein receptor-related p	3	110.2	95.5	101	103	102.85	102	0.991735537	0.859355
Q96NB3	ZNF830	Zinc finger protein 830 OS=Homo sapiens G	3	101.1	92	91.1	100.4	96.55	95.75	0.991714138	0.859658
H3BM74	NUB1	NEDD8 ultimate buster 1 OS=Homo sapiens	1	109.2	93.6	93.2	107.9	101.4	100.55	0.991617357	0.861027
A0A0D9SGE8	PHF6	PHD finger protein 6 OS=Homo sapiens GN=	5	104.4	98.3	100.1	100.9	101.35	100.5	0.991613222	0.861086
A0A024R8T9	SYNGR2	Synaptogyrin OS=Homo sapiens GN=SYNG	4	103.1	99.3	100.3	100.4	101.2	100.35	0.991600791	0.861262
A0A024RD11	PPP2R5D	Protein phosphatase 2, regulatory subunit B (	5	100.1	102	99	101.4	101.05	100.2	0.991588323	0.861439
U4PSC4	MED4	Mediator complex subunit 4 (Fragment) OS=	6	107.9	94	98.9	101.3	100.95	100.1	0.99157999	0.861557
B3KWJ4		cDNA FLJ43156 fis, clone DFNES2011239,	2	98.2	102.9	104.5	94.9	100.55	99.7	0.991546494	0.862031
Q9BZZ5	API5	Apoptosis inhibitor 5 OS=Homo sapiens GN=	15	101.5	99.2	99.7	99.3	100.35	99.5	0.991529646	0.86227
Q9Y4C2	TCAF1	TRPM8 channel-associated factor 1 OS=Hom	3	102.6	97.9	98.6	100.2	100.25	99.4	0.991521197	0.86239
Q9UBV2	SEL1L	Protein sel-1 homolog 1 OS=Homo sapiens G	12	100.7	99.8	98.9	99.9	100.25	99.4	0.991521197	0.86239
Q9BSC4	NOL10	Nucleolar protein 10 OS=Homo sapiens GN=	14	100.3	99.4	99.9	98.1	99.85	99	0.991487231	0.862871
P56277	CMC4	Cx9C motif-containing protein 4 OS=Homo s	1	100.1	99.1	98.4	99.1	99.6	98.75	0.991465863	0.863174
O75380	NDUFS6	NADH dehydrogenase [ubiquinone] iron-sulf	5	96.5	102.7	97.9	99.6	99.6	98.75	0.991465863	0.863174
Q8NCG7	DAGLB	Sn1-specific diacylglycerol lipase beta OS=H	4	96.2	103	93.9	103.6	99.6	98.75	0.991465863	0.863174
Q8N5F7	NKAP	NF-kappa-B-activating protein OS=Homo sa	1	100.8	98.3	102.6	94.8	99.55	98.7	0.991461577	0.863234
O15031	PLXNB2	Plexin-B2 OS=Homo sapiens GN=PLXNB2	20	100.7	97.8	96.6	100.2	99.25	98.4	0.991435768	0.8636
B3KS98	EIF3H	Eukaryotic translation initiation factor 3 subu	14	97.8	97.8	97.7	96.2	97.8	96.95	0.991308793	0.8654
O15230	LAMA5	Laminin subunit alpha-5 OS=Homo sapiens	4	96.4	98	93.2	99.5	97.2	96.35	0.991255144	0.866161
Q4VXZ2	VPS52	Vacuolar protein sorting 52 (Yeast) OS=Hom	3	102.2	103.3	100.7	103	102.75	101.85	0.991240876	0.866364
O75581	LRP6	Low-density lipoprotein receptor-related prot	1	106.5	98.4	98.4	104.7	102.45	101.55	0.991215227	0.866727
Q53EL1		Protein KIAA0196 variant (Fragment) OS=H	10	104.5	99.6	97.5	104.8	102.05	101.15	0.991180794	0.867216
Q71VA3		Beta 1,4-galactosyltransferase homolog OS=	1	100.2	92	84.1	106.4	96.1	95.25	0.991155047	0.867581
A0A024R0E2	CSDE1	Cold shock domain containing E1, RNA-bind	33	101.3	101.7	101.2	100	101.5	100.6	0.991133005	0.867893
Q1HBJ4	MAPK1	Mitogen-activated protein kinase OS=Homo s	4	101.8	101	100.3	100.7	101.4	100.5	0.99112426	0.868017
Q969H8	MYDGF	Myeloid-derived growth factor OS=Homo sa	5	99.1	103.7	101.4	99.6	101.4	100.5	0.99112426	0.868017
B4DN31		cDNA FLJ55809 OS=Homo sapiens PE=2 SV	7	100.1	102.6	103.2	97.7	101.35	100.45	0.991119882	0.86808
P35270	SPR	Sepiapterin reductase OS=Homo sapiens GN=	7	99.6	103	99.8	101	101.3	100.4	0.991115499	0.868142
Q5VYK3	ECM29	Proteasome-associated protein ECM29 homol	12	102.4	100.2	98.4	102.4	101.3	100.4	0.991115499	0.868142
Q9UH65	SWAP70	Switch-associated protein 70 OS=Homo sapie	13	100.6	101.6	99.4	101	101.1	100.2	0.991097923	0.868391
Q32P41	TRMT5	tRNA (guanine(37)-N1)-methyltransferase O	2	98.2	103.7	91.8	108.3	100.95	100.05	0.991084695	0.868579
Q14204	DYNC1H1	Cytoplasmic dynein 1 heavy chain 1 OS=Hom	140	100.7	100.9	100	99.8	100.8	99.9	0.991071429	0.868768
B4E0X8		cDNA FLJ61021, highly similar to Far upstre	2	103.4	97.2	94.1	104.7	100.3	99.4	0.991026919	0.869399
A0A0A0MT6	FKBP15	FK506-binding protein 15 (Fragment) OS=H	4	103.7	96.7	100.6	98	100.2	99.3	0.991017964	0.869527
O15160	POLR1C	DNA-directed RNA polymerases I and III sub	9	100.9	99.2	99.2	99.1	100.05	99.15	0.991004498	0.869717
A0A024QZJ7	CCDC6	Coiled-coil domain containing 6, isoform CR	8	100.1	99.9	97.3	100.9	100	99.1	0.991	0.869781
P10588	NR2F6	Nuclear receptor subfamily 2 group F membe	3	98.6	101	96.4	101.4	99.8	98.9	0.990981964	0.870038
Q09328	MGAT5	Alpha-1,6-mannosylglycoprotein 6-beta-N-ac	1	105.5	94.1	92.4	105.4	99.8	98.9	0.990981964	0.870038

Q96JB5	CDK5RAP3	CDK5 regulatory subunit-associated protein 3	9	100.1	99.4	99.2	98.5	99.75	98.85	0.990977444	0.870101
B2RDZ9		cDNA, FLJ96850 OS=Homo sapiens PE=2 S	10	99.3	98.7	98.7	97.5	99	98.1	0.990909091	0.871072
Q5SY16	NOL9	Polynucleotide 5'-hydroxyl-kinase NOL9 OS=	10	99.2	98.3	99.2	96.5	98.75	97.85	0.990886076	0.871399
P62891	RPL39	60S ribosomal protein L39 OS=Homo sapiens	1	91.7	103.7	95.1	98.5	97.7	96.8	0.990788127	0.872791
Q9BQD7	FAM173A	Protein FAM173A OS=Homo sapiens GN=F	1	110.7	95	100.8	103	102.85	101.9	0.990763247	0.873144
Q9BVP2	GNL3	Guanine nucleotide-binding protein-like 3 OS	1	109.3	96.1	102.6	100.9	102.7	101.75	0.990749757	0.873336
Q9BRZ2	TRIM56	E3 ubiquitin-protein ligase TRIM56 OS=Hom	1	118.7	86.5	94.9	108.4	102.6	101.65	0.990740741	0.873464
Q15696	ZRSR2	U2 small nuclear ribonucleoprotein auxiliary	2	102.5	102.3	104.9	98	102.4	101.45	0.990722656	0.873721
Q9BUF5	TUBB6	Tubulin beta-6 chain OS=Homo sapiens GN=	9	100.7	103.5	101.4	100.9	102.1	101.15	0.990695397	0.874108
A4FTY4	TXNRD2	TXNRD2 protein OS=Homo sapiens GN=TX	1	91.9	101.4	98.8	92.7	96.65	95.75	0.99068805	0.874213
H0Y8C2	RPL22L1	60S ribosomal protein L22-like 1 (Fragment)	4	99.5	103.2	103.3	97.5	101.35	100.4	0.990626542	0.875088
Q9Y3B2	EXOSC1	Exosome complex component CSL4 OS=Hon	4	101.5	100.8	99	101.4	101.15	100.2	0.990608008	0.87535
P78357	CNTNAP1	Contactin-associated protein 1 OS=Homo sap	8	102.2	99.9	99.3	100.9	101.05	100.1	0.990598714	0.875483
Q13131	PRKAA1	5'-AMP-activated protein kinase catalytic sub	4	99	102.7	97.4	102.4	100.85	99.9	0.990580069	0.875748
Q96IZ7	RSRC1	Serine/Arginine-related protein 53 OS=Homo	3	102.2	99.3	102.3	97.3	100.75	99.8	0.99057072	0.875881
P09496	CLTA	Clathrin light chain A OS=Homo sapiens GN	9	101.4	100	101.1	98.4	100.7	99.75	0.990566038	0.875948
B3KTJ9		cDNA FLJ38393 fis, clone FEBRA2007212	27	101.4	99.7	99.4	99.8	100.55	99.6	0.990551964	0.876148
Q96RS6	NUDCD1	NudC domain-containing protein 1 OS=Homo	7	98.5	101.8	93.9	104.5	100.15	99.2	0.990514229	0.876685
A0A024R911	XPR1	Xenotropic and polytropic retrovirus receptor,	7	102.9	97.3	98.4	99.9	100.1	99.15	0.990509491	0.876752
E5KN59		Peptidyl-prolyl cis-trans isomerase D OS=Ho	13	98.1	101.8	98.7	99.3	99.95	99	0.990495248	0.876954
O95359	TACC2	Transforming acidic coiled-coil-containing pr	2	98.7	101.1	101.9	96	99.9	98.95	0.99049049	0.877022
A8K732		cDNA FLJ78771, highly similar to Homo sap	4	100.4	99.4	99.8	98.1	99.9	98.95	0.99049049	0.877022
I3VM53	KDM2A	F-box and leucine-rich repeat protein 11, isof	9	101	98.7	99.1	98.7	99.85	98.9	0.990485729	0.87709
Q6YP21	KYAT3	Kynurenine--oxoglutarate transaminase 3 OS=	11	99.5	99.5	100.4	96.7	99.5	98.55	0.990452261	0.877566
J3KQ32	OLA1	Obg-like ATPase 1 OS=Homo sapiens GN=C	11	98	100.8	98.6	98.3	99.4	98.45	0.990442656	0.877703
P78346	RPP30	Ribonuclease P protein subunit p30 OS=Hom	8	97.2	100.8	96.7	99.4	99	98.05	0.99040404	0.878252
F8W727	RPL32	60S ribosomal protein L32 OS=Homo sapiens	7	99.8	106	105.2	98.6	102.9	101.9	0.990281827	0.879992
B8ZZ87	MZT2B	Mitotic-spindle organizing protein 2B OS=Ho	1	106.8	98.6	89.2	114.2	102.7	101.7	0.990262902	0.880261
B3KMV8		cDNA FLJ12766 fis, clone NT2RP2001520,	13	104.8	100.1	101.7	101.2	102.45	101.45	0.990239141	0.880599
A0A024R2L1	WDR48	Testicular tissue protein Li 224 OS=Homo sa	3	102.1	101.7	99.6	102.2	101.9	100.9	0.990186457	0.88135
P60228	EIF3E	Eukaryotic translation initiation factor 3 subu	18	101.3	102.4	101.5	100.2	101.85	100.85	0.99018164	0.881418
A8K7F6		cDNA FLJ78244, highly similar to Homo sap	12	98.6	104.4	98.8	102.2	101.5	100.5	0.990147783	0.881901
H0Y2S1	GTPBP6	Putative GTP-binding protein 6 OS=Homo sa	7	99.7	103	99.7	101	101.35	100.35	0.990133202	0.882108
Q69YJ7	DKFZp667H19	Putative uncharacterized protein DKFZp667H	14	101.4	101.3	98.2	102.5	101.35	100.35	0.990133202	0.882108
B2RAF3		cDNA, FLJ94877, highly similar to Homo sa	3	105.4	97.1	103.7	96.8	101.25	100.25	0.990123457	0.882247
Q14247	CTTN	Src substrate cortactin OS=Homo sapiens GN	19	103.8	98.7	101.5	99	101.25	100.25	0.990123457	0.882247
Q99471	PFDN5	Prefoldin subunit 5 OS=Homo sapiens GN=P	8	100.4	102.1	98.7	101.8	101.25	100.25	0.990123457	0.882247
A0A024RA52	PSMA2	Proteasome subunit alpha type OS=Homo sap	12	99.6	102.8	99.4	101	101.2	100.2	0.990118577	0.882317
P62834	RAP1A	Ras-related protein Rap-1A OS=Homo sapien	4	99.6	102.3	101.5	98.4	100.95	99.95	0.990094106	0.882665
A8K566		cDNA FLJ78246, highly similar to Homo sap	15	101.7	99.9	100	99.6	100.8	99.8	0.990079365	0.882875
A0A024R8B6	NUP214	Nucleoporin 214kDa, isoform CRA_b OS=Ho	23	104.3	97.1	98.5	100.9	100.7	99.7	0.990069513	0.883015
Q13344		Fus-like protein (Fragment) OS=Homo sapien	8	96.3	104.3	98.4	100.2	100.3	99.3	0.99002991	0.883579
P08579	SNRPB2	U2 small nuclear ribonucleoprotein B" OS=H	6	99.4	100.4	97.3	100.5	99.9	98.9	0.98998999	0.884148
P35908	KRT2	Keratin, type II cytoskeletal 2 epidermal OS=	11	103.1	106.1	101.8	105.3	104.6	103.55	0.989961759	0.884551
O75792	RNASEH2A	Ribonuclease H2 subunit A OS=Homo sapien	1	100.9	98.2	92.1	105	99.55	98.55	0.989954797	0.884651
P43034	PAFAH1B1	Platelet-activating factor acetylhydrolase IB s	14	99.4	99.5	99.3	97.6	99.45	98.45	0.989944696	0.884794
A8KAL2		cDNA FLJ76981, highly similar to Homo sap	4	99.4	98.3	98.2	97.5	98.85	97.85	0.989883662	0.885664
M0QZ12	GRAMD1A	GRAM domain-containing protein 1A OS=Ho	5	97.8	99.3	97.8	97.3	98.55	97.55	0.989852867	0.886104
P68366	TUBA4A	Tubulin alpha-4A chain OS=Homo sapiens G	3	90.2	97	92	93.3	93.6	92.65	0.989850427	0.886138
Q8N9R8	SCAI	Protein SCAI OS=Homo sapiens GN=SCAI	1	98.9	97.4	91.8	102.5	98.15	97.15	0.989811513	0.886693
Q9P265	DIP2B	Disco-interacting protein 2 homolog B OS=H	3	104.3	99.7	104.8	97.1	102	100.95	0.989705882	0.8882
O15488	GYG2	Glycogenin-2 OS=Homo sapiens GN=GYG2	1	104.8	89.3	93.8	98.3	97.05	96.05	0.989696033	0.88834
Q59FT7		Mitogen-activated protein kinase kinase kinas	3	95.1	99	98.1	94	97.05	96.05	0.989696033	0.88834
Q14257	RCN2	Reticulocalbin-2 OS=Homo sapiens GN=RCN	12	104.6	98.6	100.4	100.7	101.6	100.55	0.989665354	0.888778
P49721	PSMB2	Proteasome subunit beta type-2 OS=Homo sa	9	101.6	101.6	101.6	99.5	101.6	100.55	0.989665354	0.888778
Q96GK7	FAHD2A	Fumarylacetoacetate hydrolase domain-contai	7	101.4	101.3	99.7	100.9	101.35	100.3	0.989639862	0.889141
Q5HYL6	DKFZp686E18	Putative uncharacterized protein DKFZp686E	9	103.5	98.5	99.1	100.8	101	99.95	0.98960396	0.889654
P51553	IDH3G	Isocitrate dehydrogenase [NAD] subunit gam	12	101.9	100	100.7	99.1	100.95	99.9	0.989598811	0.889727
A0A024R8C1	TTF1	Transcription termination factor, RNA polym	2	103.9	97.8	103.9	95.7	100.85	99.8	0.989588498	0.889875
Q9NVV4	MTPAP	Poly(A) RNA polymerase, mitochondrial OS=	11	102.3	99.3	100.5	99	100.8	99.75	0.989583333	0.889949
V9HW87	HEL-S-299	Abhydrolase domain containing 14B, isoform	5	102.6	98.5	98.9	100.1	100.55	99.5	0.989557434	0.890318
Q9H845	ACAD9	Acyl-CoA dehydrogenase family member 9,	20	101.4	99.2	99.8	98.7	100.3	99.25	0.989531406	0.89069
O00291	HIP1	Huntingtin-interacting protein 1 OS=Homo sa	8	102.3	98.1	98.4	99.9	100.2	99.15	0.989520958	0.890839
D6RFN0	COPS4	COP9 signalosome complex subunit 4 OS=H	20	98.8	100.6	97.5	99.8	99.7	98.65	0.989468405	0.891589
Q8WVC0	LEO1	RNA polymerase-associated protein LEO1 O	5	99.4	98.7	98.4	97.6	99.05	98	0.989399293	0.892576
Q9BXY0	MAK16	Protein MAK16 homolog OS=Homo sapiens	8	99.2	98.6	98	97.7	98.9	97.85	0.989383215	0.892805
A0A024RBA9	RAB21	RAB21, member RAS oncogene family, isofo	10	106.1	99.7	102.6	101	102.9	101.8	0.98931001	0.893851
Q8N5M9	JAGN1	Protein jagunal homolog 1 OS=Homo sapiens	3	103	101.4	101.5	100.7	102.2	101.1	0.989236791	0.894898
B2RD27		cDNA, FLJ96428, highly similar to Homo sa	8	100.3	103.6	100.4	101.3	101.95	100.85	0.989210397	0.895275
E7ESA6	PTK2	Focal adhesion kinase 1 OS=Homo sapiens G	3	104.2	99.5	95.8	105.7	101.85	100.75	0.989199804	0.895426
O75663	TIPRL	TIP41-like protein OS=Homo sapiens GN=TI	6	104	99.7	100.7	100.8	101.85	100.75	0.989199804	0.895426
V9GZ56	LSM4	U6 snRNA-associated Sm-like protein LSm4	5	99.6	103.7	100.8	100.3	101.65	100.55	0.989178554	0.89573
B3KQ21		cDNA FLJ32640 fis, clone SYNOV2001033,	14	104.1	98.4	100.7	99.6	101.25	100.15	0.989135802	0.896341
Q14015		PR371 c-K-ras oncogene (Fragment) OS=Ho	2	94.3	98.9	97.7	93.4	96.6	95.55	0.989130435	0.896417
Q92621	NUP205	Nuclear pore complex protein Nup205 OS=H	39	103	99.1	101.8	98.1	101.05	99.95	0.9891143	0.896648
A0A024R2H7	TRNT1	tRNA nucleotidyl transferase, CCA-adding, 1	4	100.3	101.7	99	100.8	101	99.9	0.989108911	0.896725
J3KN32	RCOR1	REST corepressor 1 OS=Homo sapiens GN=	8	102.4	99.6	102	97.8	101	99.9	0.989108911	0.896725
Q32Q14	NDUFA7	NDUFA7 protein (Fragment) OS=Homo sapi	3	101.8	99.7	96.7	102.6	100.75	99.65	0.989081886	0.897112
P29353	SHC1	SHC-transforming protein 1 OS=Homo sapie	10	99.9	101.1	98.6	100.2	100.5	99.4	0.989054726	0.8975
H0YAA8	MED28	Mediator of RNA polymerase II transcription	2	100	100.2	101.6	96.4	100.1	99	0.989010989	0.898125
A8K4G7		cDNA FLJ78528, highly similar to Homo sap	1	108	91.9	99.9	97.8	99.95	98.85	0.988994497	0.898361
A0A140VKF1		Kinesin-like protein OS=Homo sapiens PE=2	12	99	100.9	98.3	99.4	99.95	98.85	0.988994497	0.898361
D3DUP1	WNK1	WNK lysine deficient protein kinase 1, isofor	2	102.5	97.3	98	99.6	99.9	98.8	0.988988989	0.89844
Q9H8V3	ECT2	Protein ECT2 OS=Homo sapiens GN=ECT2	3	104.9	93.9	94.4	102.2	99.4	98.3	0.988933602	0.899232
O43660	PLRG1	Pleiotropic regulator 1 OS=Homo sapiens GN	17	100.4	97.2	98.3	97.1	98.8	97.7	0.988866397	0.900194
P20585	MSH3	DNA mismatch repair protein Msh3 OS=Hom	3	97.7	99.5	97.9	97.1	98.6	97.5	0.988843813	0.900517
A0A024RDJ4	NFKB1	Nuclear factor of kappa light polypeptide gen	5	96.3	99.9	90.8	103.2	98.1	97	0.988786952	0.90133
A0A024R4E5	HDLBP	High density lipoprotein binding protein (Vig	48	102	103	102.2	100.5	102.5	101.35	0.988780488	0.901423
Q9UL03	INTS6	Integrator complex subunit 6 OS=Homo sapie	5	104.9	100	101.8	100.8	102.45	101.3	0.988775012	0.901501
Q8NDT2	RBM15B	Putative RNA-binding protein 15B OS=Hom	9	98.7	97.1	93.5	100.1	97.9	96.8	0.988764045	0.901658
P08708	RPS17	40S ribosomal protein S17 OS=Homo sapiens	9	98.5	105.4	101.4	100.2	101.95	100.8	0.988719961	0.902289
Q6P996	PDXDC1	Pyridoxal-dependent decarboxylase domain-c	6	108.5	104.1	105.6	104.6	106.3	105.1	0.988711195	0.902415
Q8IY18	SMC5	Structural maintenance of chromosomes prote	8	100.8	101.5	99.3	100.7	101.15	100	0.988630746	0.903566
B2RBE5		cDNA, FLJ95468, highly similar to Homo sa	28	100.5	101.6	100.1	99.7	101.05	99.9	0.988619495	0.903727
O15541	RNF113A	RING finger protein 113A OS=Homo sapiens	2	96.3	105.4	102.6	96.8	100.85	99.7	0.988596926	0.904051
Q9H2W6	MRPL46	39S ribosomal protein L46, mitochondrial OS	9	101.3	100.4	102.1	97.3	100.85	99.7	0.988596926	0.904051
B4E3H1		cDNA FLJ53284, highly similar to Supervilli	2	100.8	100.3	94.4	104.4	100.55	99.4	0.988562904	0.904538
A0A090N8Y5	LSM5	LSM5 homolog, U6 small nuclear RNA asso	2	98.6	102.3	92.7	105.9	100.45	99.3	0.988551518	0.904701
D3DTH7	MYO1C	Myosin IC, isoform CRA_a OS=Homo sapien	1	101.5	99.3	102.8	95.7	100.4	99.25	0.988545817	0.904782
P48739	PITPNB	Phosphatidylinositol transfer protein beta isof	7	98.2	102.5	99.2	99.2	100.35	99.2	0.98854011	0.904864
Q8IY37	DHX37	Probable ATP-dependent RNA helicase DHX	19	101	99.7	96.6	101.8	100.35	99.2	0.98854011	0.904864
Q6WKZ4	RAB11FIP1	Rab11 family-interacting protein 1 OS=Hom	4	104.4	105	101.4	105.6	104.7	103.5	0.988538682	0.904885
Q9NX46	ADPRHL2	Poly(ADP-ribose) glycohydrolase ARH3 OS=	4	101.5	99.1	99	99.3	100.3	99.15	0.988534397	0.904946
P48735	IDH2	Isocitrate dehydrogenase [NADP], mitochond	21	98.4	101.9	98.7	99.3	100.15	99	0.988517224	0.905192
F8VYN9	ARL1	ADP-ribosylation factor-like protein 1 OS=H	5	102	98.2	99.7	98.2	100.1	98.95	0.988511489	0.905274
A8K2W4		cDNA FLJ76247, highly similar to Homo sap	3	102.6	97.5	97.2	100.6	100.05	98.9	0.988505747	0.905357
Q02040	AKAP17A	A-kinase anchor protein 17A OS=Homo sapie	7	101.8	97.8	95.3	102	99.8	98.65	0.988476954	0.905769
A9QQ22	WASH	Actin nucleation promoting factor (Fragment)	1	96.4	94.1	94.8	93.5	95.25	94.15	0.988451444	0.906135
Q59GY0		Apolipoprotein B mRNA editing enzyme, cat	6	100.1	99	99.8	97	99.55	98.4	0.988448016	0.906183
Q68D09	DKFZp686D19	Putative uncharacterized protein DKFZp686D	1	115.5	109	115.8	106.1	112.25	110.95	0.988418708	0.906603
P20700	LMNB1	Lamin-B1 OS=Homo sapiens GN=LMNB1 P	34	100.1	98.1	98.2	97.7	99.1	97.95	0.98839556	0.906935
G3V1V0	MYL6	Myosin light polypeptide 6 OS=Homo sapien	1	92.7	105.2	93.1	102.5	98.95	97.8	0.988377969	0.907187
P06733	ENO1	Alpha-enolase OS=Homo sapiens GN=ENO1	23	95.5	102.1	96.3	99	98.8	97.65	0.988360324	0.90744
Q14258	TRIM25	E3 ubiquitin/ISG15 ligase TRIM25 OS=Hom	13	104.4	101.5	100.3	103.2	102.95	101.75	0.988343856	0.907676
P27105	STOM	Erythrocyte band 7 integral membrane protei	8	109.4	95	100.7	101.3	102.2	101	0.988258317	0.908902
P33527	ABCC1	Multidrug resistance-associated protein 1 OS=	17	106.7	97.5	101.8	100	102.1	100.9	0.988246817	0.909067
Q13724	MOGS	Mannosyl-oligosaccharide glucosidase OS=H	22	100.2	103.8	101	100.6	102	100.8	0.988235294	0.909232
Q9UM13	ANAPC10	Anaphase-promoting complex subunit 10 OS=	3	96.9	98.4	98.5	94.5	97.65	96.5	0.988223246	0.909404
Q14669	TRIP12	E3 ubiquitin-protein ligase TRIP12 OS=Hom	29	102.3	100	101.4	98.5	101.15	99.95	0.988136431	0.91065
O43166	SIPA1L1	Signal-induced proliferation-associated 1-like	4	100.1	101.7	96.5	102.9	100.9	99.7	0.988107037	0.911071
Q15029	EFTUD2	116 kDa U5 small nuclear ribonucleoprotein	44	98	103.7	100.9	98.4	100.85	99.65	0.98810114	0.911156
B3KY51		cDNA FLJ46863 fis, clone UTERU3011558	14	102.6	99	98.3	100.9	100.8	99.6	0.988095238	0.91124

A8K492		cDNA FLJ76789, highly similar to Homo sap	23	101	100.6	100.7	98.5	100.8	99.6	0.988095238	0.91124
Q96DV4	MRPL38	39S ribosomal protein L38, mitochondrial OS	9	100.7	100.2	100.4	98.1	100.45	99.25	0.988053758	0.911835
B5BUB1	RUVBL1	RuvB-like helicase (Fragment) OS=Homo sap	23	99.6	100.9	99	99.1	100.25	99.05	0.988029925	0.912177
Q53GS7	GLE1	Nucleoporin GLE1 OS=Homo sapiens GN=G	5	101.6	103.6	102.9	99.8	102.6	101.35	0.987816764	0.915236
Q8NBJ5	COLGALT1	Procollagen galactosyltransferase 1 OS=Hom	15	105.6	99.5	104.2	98.4	102.55	101.3	0.987810824	0.915321
Q16864	ATP6V1F	V-type proton ATPase subunit F OS=Homo s	3	90.7	89.3	87.1	90.7	90	88.9	0.987777778	0.915795
A8JZZ8		cDNA FLJ77826, highly similar to Homo sap	11	97.5	98.5	95	98.6	98	96.8	0.987755102	0.916121
O75616	ERAL1	GTPase Era, mitochondrial OS=Homo sapien	10	106.1	97.9	100.3	101.2	102	100.75	0.987745098	0.916264
A0A024R8A2	GAPVD1	GTPase activating protein and VPS9 domains	7	105.7	98.2	100.5	100.9	101.95	100.7	0.987739088	0.916351
A4D1L5	UBE2H	Ubiquitin-conjugating enzyme E2H (UBC8 h	2	103.9	100	98.8	102.6	101.95	100.7	0.987739088	0.916351
X5D8S6	ADSL	Adenylosuccinate lyase (Fragment) OS=Hom	9	101.1	102.7	101.6	99.7	101.9	100.65	0.987733072	0.916437
A8K146		cDNA FLJ76216, highly similar to Homo sap	5	99.6	95.8	93.2	99.8	97.7	96.5	0.987717503	0.916661
Q6NUM9	RETSAT	All-trans-retinol 13,14-reductase OS=Homo s	2	104.1	99.3	99.6	101.3	101.7	100.45	0.987708948	0.916784
P43155	CRAT	Carnitine O-acetyltransferase OS=Homo sapi	4	98.7	95.7	99.9	92.1	97.2	96	0.987654321	0.917568
Q7L2H7	EIF3M	Eukaryotic translation initiation factor 3 subu	11	102.5	100	101.6	98.4	101.25	100	0.987654321	0.917568
O75054	IGSF3	Immunoglobulin superfamily member 3 OS=I	1	103.7	98.7	93.6	106.3	101.2	99.95	0.987648221	0.917655
Q9BQC3	DPH2	Diphthamide biosynthesis protein 2 OS=Hom	1	97.5	96.5	96.6	95	97	95.8	0.987628866	0.917933
P36954	POLR2I	DNA-directed RNA polymerase II subunit RI	5	101.7	100	100.4	98.8	100.85	99.6	0.987605354	0.918272
P05198	EIF2S1	Eukaryotic translation initiation factor 2 subu	18	101.7	99.8	98.8	100.2	100.75	99.5	0.987593052	0.918448
Q9UQ13	SHOC2	Leucine-rich repeat protein SHOC-2 OS=Hon	4	102.4	98.5	91.2	107.2	100.45	99.2	0.987555998	0.91898
Q9BV81	EMC6	ER membrane protein complex subunit 6 OS=	1	97.8	102.6	103	94.9	100.2	98.95	0.98752495	0.919427
A0A0C4DFS6	SPRY4	Protein sprouty homolog 4 OS=Homo sapiens	3	98.8	101.6	98.7	99.2	100.2	98.95	0.98752495	0.919427
A0A024R1T1	RPS19BP1	Ribosomal protein S19 binding protein 1, isof	5	103.1	97.3	101.6	96.3	100.2	98.95	0.98752495	0.919427
Q9BQ75	CMSS1	Protein CMSS1 OS=Homo sapiens GN=CMS	7	99.5	100	101.7	95.3	99.75	98.5	0.987468672	0.920235
Q5HYL4	DKFZp686E18	Putative uncharacterized protein DKFZp686E	11	100.5	98.9	98.5	98.4	99.7	98.45	0.987462387	0.920325
A0A024R745	NDUFA5	NADH dehydrogenase (Ubiquinone) 1 alpha s	7	101.4	97.6	97.7	98.8	99.5	98.25	0.987437186	0.920687
P14780	MMP9	Matrix metalloproteinase-9 OS=Homo sapien	1	119.1	87	92.3	111.2	103.05	101.75	0.987384765	0.921441
B7ZKM6	UACA	Uveal autoantigen with coiled-coil domains a	5	102.7	102.3	101.3	101.1	102.5	101.2	0.987317073	0.922413
F2Z3H6	BCL7B	B-cell CLL/lymphoma 7 protein family memb	2	104.5	100.1	104.8	97.2	102.3	101	0.987292278	0.92277
G5E9A6	USP11	Ubiquitin carboxyl-terminal hydrolase 11 OS=	5	101.4	95.3	91.2	103	98.35	97.1	0.98729029	0.922799
Q4QQP8	PTGFRN	PTGFRN protein (Fragment) OS=Homo sapie	7	105.5	99	104.4	97.5	102.25	100.95	0.987286064	0.922859
O00399	DCTN6	Dynactin subunit 6 OS=Homo sapiens GN=D	2	99.9	103.9	102	99.2	101.9	100.6	0.987242395	0.923487
A0A024R9Y6	GNL3L	Guanine nucleotide binding protein-like 3 (Nu	15	101.7	101.4	101.2	99.3	101.55	100.25	0.987198424	0.924119
O00116	AGPS	Alkyldihydroxyacetonephosphate synthase, p	23	102	100.7	100.7	99.4	101.35	100.05	0.987173162	0.924483
O75818	RPP40	Ribonuclease P protein subunit p40 OS=Hom	4	100.1	102.6	98.6	101.5	101.35	100.05	0.987173162	0.924483
P82675	MRPS5	28S ribosomal protein S5, mitochondrial OS=	13	99.6	102.9	101	98.9	101.25	99.95	0.987160494	0.924665
Q9BY50	SEC11C	Signal peptidase complex catalytic subunit SE	1	117	93.2	102.6	104.9	105.1	103.75	0.98715509	0.924743
O14936	CASK	Peripheral plasma membrane protein CASK C	6	103.2	99.1	99.3	100.4	101.15	99.85	0.9871478	0.924847
B3KUE6		cDNA FLJ39696 fis, clone SMINT2011033, l	1	95.4	99	94.6	97.3	97.2	95.95	0.987139918	0.92496
Q92615	LARP4B	La-related protein 4B OS=Homo sapiens GN=	13	100.4	101.7	100.1	99.4	101.05	99.75	0.987135082	0.925031
Q04446	GBE1	1,4-alpha-glucan-branching enzyme OS=Hom	7	103.6	98.2	95	104.2	100.9	99.6	0.987115956	0.925305
Q6IBN6	CBX1	CBX1 protein OS=Homo sapiens GN=CBX1	7	101.8	99.3	98.1	100.4	100.55	99.25	0.987071109	0.92595
A0A024R6K1	CCNK	Cyclin K, isoform CRA_c OS=Homo sapiens	5	101.4	98.9	99.4	98.3	100.15	98.85	0.987019471	0.926693
Q15642	TRIP10	Cdc42-interacting protein 4 OS=Homo sapien	5	99.9	100.2	94.1	103.4	100.05	98.75	0.987006497	0.92688
O95685	PPP1R3D	Protein phosphatase 1 regulatory subunit 3D (	1	91.7	108.2	95.5	101.8	99.95	98.65	0.986993497	0.927067
O00257	CBX4	E3 SUMO-protein ligase CBX4 OS=Homo sa	7	98	101.8	100.1	97.1	99.9	98.6	0.986986987	0.92716
O15047	SETD1A	Histone-lysine N-methyltransferase SETD1A	3	101.7	97.8	97.8	99.1	99.75	98.45	0.986967419	0.927442
Q9H488	POFUT1	GDP-fucose protein O-fucosyltransferase 1 O	10	102.4	104.2	103.3	100.6	103.3	101.95	0.986931268	0.927962
A0A140VK11	CLPB	ClpB caseinolytic peptidase B homolog (E. co	5	101.6	96.5	97.6	97.9	99.05	97.75	0.986875315	0.928768
B4E0Y9	STK26	Serine/threonine-protein kinase 26 OS=Homo	7	104.3	101.3	101.7	101.2	102.8	101.45	0.986867704	0.928877
A0A024R061	SYBL1	Synaptobrevin-like 1, isoform CRA_e OS=Ho	7	105.7	99.7	102.8	99.9	102.7	101.35	0.986854917	0.929061
P42696	RBM34	RNA-binding protein 34 OS=Homo sapiens G	12	102.7	94.9	98.4	96.6	98.8	97.5	0.986842105	0.929245
Q9Y3E0	GOLT1B	Vesicle transport protein GOT1B OS=Homo s	1	95.7	101.9	100.5	94.5	98.8	97.5	0.986842105	0.929245
Q13263	TRIM28	Transcription intermediary factor 1-beta OS=I	38	100.7	104.2	101.4	100.8	102.45	101.1	0.98682284	0.929523
Q13888	GTF2H2	General transcription factor IIH subunit 2 OS=	6	99	98.3	94.5	100.2	98.65	97.35	0.986822098	0.929534
Q9UBQ5	EIF3K	Eukaryotic translation initiation factor 3 subu	7	103.7	100.5	100.7	100.8	102.1	100.75	0.986777669	0.930173
B2RAU5		Sorting nexin OS=Homo sapiens PE=2 SV=1	8	103	100.9	97.1	104.1	101.95	100.6	0.986758215	0.930453
G3V5T9	CDK2	Cyclin-dependent kinase 2 OS=Homo sapiens	3	106	96.9	100.3	99.9	101.45	100.1	0.986692952	0.931392
A0A0D9SEI0	C6orf203	Uncharacterized protein C6orf203 OS=Homo	1	109.7	93.1	95.7	104.4	101.4	100.05	0.986686391	0.931487
Q8IUF8	MINA	Bifunctional lysine-specific demethylase and	6	101.4	101.4	100.8	99.3	101.4	100.05	0.986686391	0.931487
Q9UKK3	PARP4	Poly [ADP-ribose] polymerase 4 OS=Homo s	2	99.8	102.8	98.3	101.6	101.3	99.95	0.986673248	0.931676
B2R7G4		cDNA, FLJ93435, highly similar to Homo sap	8	105.5	96.4	99	100.2	100.95	99.6	0.986627043	0.932342
F5H7W8	C12orf43	Chromosome 12 open reading frame 43, isofo	3	96.5	90.4	90.3	94.1	93.45	92.2	0.986623863	0.932387
P55735	SEC13	Protein SEC13 homolog OS=Homo sapiens G	9	103.5	98.2	101.1	97.9	100.85	99.5	0.986613783	0.932532
Q9NPF5	DMAP1	DNA methyltransferase 1-associated protein 1	7	102.8	98.5	98.7	99.9	100.65	99.3	0.986587183	0.932916
A0A0A0MR6	RBM10	RNA binding motif protein 10, isoform CRA_	15	101	100.3	99.8	98.8	100.65	99.3	0.986587183	0.932916
A8K6D2		cDNA FLJ76620, highly similar to Homo sap	11	104.5	96.2	97.7	100.3	100.35	99	0.986547085	0.933493
Q96L92	SNX27	Sorting nexin-27 OS=Homo sapiens GN=SN	7	101.3	99.2	98.3	99.5	100.25	98.9	0.986533666	0.933687
O43617	TRAPPC3	Trafficking protein particle complex subunit 3	2	100.3	100	95.9	101.7	100.15	98.8	0.98652022	0.93388
A0A024QZ72	PRDM2	PR domain containing 2, with ZNF domain, is	2	90.8	108.3	88.7	107.7	99.55	98.2	0.986438975	0.93505
Q9NXV6	CDKN2AIP	CDKN2A-interacting protein OS=Homo sapie	8	99.3	107.1	106.3	97.3	103.2	101.8	0.986434109	0.935121
Q1KMD3	HNRNPUL2	Heterogeneous nuclear ribonucleoprotein U-li	30	100.1	98.7	99	97.1	99.4	98.05	0.986418511	0.935346
A0A140VJX5		Testicular tissue protein Li 202 OS=Homo sap	4	98.9	99.5	98.8	96.9	99.2	97.85	0.986391129	0.93574
Q8IZ81	ELMOD2	ELMO domain-containing protein 2 OS=Hom	5	101.9	96.5	96.9	98.8	99.2	97.85	0.986391129	0.93574
B7Z7G4		cDNA FLJ60939, highly similar to NAD-dep	2	96.1	87.6	89.1	92.1	91.85	90.6	0.986390855	0.935744
A8K3Z5		Nucleoporin NUP53 OS=Homo sapiens PE=2	7	101.8	96.5	97.2	98.4	99.15	97.8	0.986384266	0.935839
P31689	DNAJA1	DnaJ homolog subfamily A member 1 OS=Ho	17	104.9	99.2	101.4	99.9	102.05	100.65	0.986281235	0.937324
Q5SRQ6	CSNK2B	Casein kinase II subunit beta OS=Homo sapie	8	102.2	101.7	100.4	100.7	101.95	100.55	0.986267778	0.937517
Q15475	SIX1	Homeobox protein SIX1 OS=Homo sapiens G	5	100	103.5	98.4	102.3	101.75	100.35	0.986240786	0.937906
A0A0S2Z5M1	SEC63	SEC63-like (S. cerevisiae) (Fragment) OS=H	17	103.8	99.5	100.1	100.4	101.65	100.25	0.98622725	0.938101
Q59ER5		WD repeat-containing protein 1 isoform 1 var	27	101.5	101.6	100.3	100	101.55	100.15	0.986213688	0.938297
Q92734	TFG	Protein TFG OS=Homo sapiens GN=TFG PE	10	101.1	101	97.9	101.4	101.05	99.65	0.986145473	0.93928
Q02127	DHODH	Dihydroorotate dehydrogenase (quinone), mit	9	102.6	99.2	98.7	100.3	100.9	99.5	0.986124876	0.939578
B2R713		cDNA, FLJ93224 OS=Homo sapiens PE=2 S	15	101.5	99.8	99.2	99.3	100.65	99.25	0.986090412	0.940075
O75494	SRSF10	Serine/arginine-rich splicing factor 10 OS=H	9	101.3	99.7	98.4	99.8	100.5	99.1	0.986069652	0.940374
P42224	STAT1	Signal transducer and activator of transcriptio	17	100.7	99.8	98.2	99.5	100.25	98.85	0.986034913	0.940875
Q9HCE1	MOV10	Putative helicase MOV-10 OS=Homo sapiens	20	100.2	100.1	97.4	100.1	100.15	98.75	0.986020969	0.941076
H0UI66	ZNF217	Zinc finger protein 217, isoform CRA_b OS=	10	102.5	97.6	98.6	98.7	100.05	98.65	0.986006997	0.941278
Q13472	TOP3A	DNA topoisomerase 3-alpha OS=Homo sapie	5	98.3	101.8	99.5	97.8	100.05	98.65	0.986006997	0.941278
A0A024RC67	PRC1	Protein regulator of cytokinesis 1, isoform CR	8	100.7	99.3	96.6	100.6	100	98.6	0.986	0.941379
B4DMS5		cDNA FLJ57399, highly similar to HIV-1 Re	1	113.5	86.5	92.1	105.1	100	98.6	0.986	0.941379
P38117	ETFB	Electron transfer flavoprotein subunit beta OS	16	100.2	99.6	97.3	99.7	99.9	98.5	0.985985986	0.941581
Q9Y6M9	NDUFB9	NADH dehydrogenase [ubiquinone] 1 beta su	7	100.3	98.9	98.4	98	99.6	98.2	0.985943775	0.942189
Q6FGP0	MRAS	MRAS protein (Fragment) OS=Homo sapiens	3	97.4	94.6	93.3	96	96	94.65	0.9859375	0.94228
P12081	HARS	Histidine--tRNA ligase, cytoplasmic OS=Hon	13	97.4	101.6	98.1	98.1	99.5	98.1	0.985929648	0.942393
A8KAH1		cDNA FLJ75839, highly similar to Homo sap	1	104.2	101.8	101.3	101.8	103	101.55	0.98592233	0.942499
H0Y2N6	CSNK1D	Casein kinase I isoform delta (Fragment) OS=	2	106.1	99.8	95.8	107.2	102.95	101.5	0.985915493	0.942598
A0A024RDB8	HPSE	Heparanase, isoform CRA_a OS=Homo sapie	1	100.2	104.7	92.8	109.2	102.45	101	0.985846755	0.943589
Q53HV2		Chaperonin containing TCP1, subunit 7 (Eta)	30	101.2	103.6	100.1	101.8	102.4	100.95	0.985839844	0.943689
J3KQJ1	SUMF2	Sulfatase-modifying factor 2 OS=Homo sapie	8	101.9	102.6	99	102.6	102.25	100.8	0.985819071	0.943989
D7RF68		AGTRAP-BRAF fusion protein OS=Homo sa	1	99.4	97.2	94.5	99.3	98.3	96.9	0.985757884	0.944872
Q9NTJ5	SACM1L	Phosphatidylinositide phosphatase SAC1 OS=	17	103.5	99.8	100.8	99.6	101.65	100.2	0.985735366	0.945197
A5YKK6	CNOT1	CCR4-NOT transcription complex subunit 1 (	34	102.8	100.2	99.6	100.5	101.5	100.05	0.985714286	0.945501
Q99567	NUP88	Nuclear pore complex protein Nup88 OS=Ho	11	101.2	101.5	100.4	99.4	101.35	99.9	0.985693143	0.945806
A0A024RDT1	MTRF1	Mitochondrial translational release factor 1, is	1	101.2	101.3	97.5	102.1	101.25	99.8	0.985679012	0.94601
Q9Y2X0	MED16	Mediator of RNA polymerase II transcription	6	103.1	99.3	99.2	100.3	101.2	99.75	0.985671937	0.946112
O75306	NDUFS2	NADH dehydrogenase [ubiquinone] iron-sulf	16	102.3	99.7	101.2	97.9	101	99.55	0.985643564	0.946522
A0MNN4	SMU1	CDW3/SMU1 OS=Homo sapiens GN=SMU1	16	104.6	97.3	99.3	99.7	100.95	99.5	0.985636454	0.946624
X5DNK3	ERBB2	Receptor protein-tyrosine kinase (Fragment) (	6	100.6	101.2	99.2	99.7	100.9	99.45	0.985629336	0.946727
Q8ND56	LSM14A	Protein LSM14 homolog A OS=Homo sapien	4	111.2	97.4	102.7	102.9	104.3	102.8	0.985618408	0.946885
Q9Y3D3	MRPS16	28S ribosomal protein S16, mitochondrial OS	5	99.3	102.3	100.5	98.2	100.8	99.35	0.985615079	0.946933
A0A024RCA1	KIAA1542	CTD-binding SR-like protein rA9, isoform CI	2	96.1	98	97.5	93.8	97.05	95.65	0.985574446	0.94752
Q96EY7	PTCD3	Pentatricopeptide repeat domain-containing p	13	103.6	97.4	98.6	99.5	100.5	99.05	0.985572139	0.947552
B2RDH6		cDNA, FLJ96613, highly similar to Homo sap	5	100	100.9	99.8	98.2	100.45	99	0.985564958	0.947657
O95757	HSPA4L	Heat shock 70 kDa protein 4L OS=Homo sap	28	100.8	99.7	98.5	99.1	100.25	98.8	0.98553616	0.948072
Q53F37		SAR1a gene homolog 2 variant (Fragment) O	2	101.8	98.4	98.7	98.6	100.1	98.65	0.985514486	0.948385
Q86WR0	CCDC25	Coiled-coil domain-containing protein 25 OS=	1	106.7	100	99.6	104.1	103.35	101.85	0.985486212	0.948793
B2R7N6		Amino acid transporter OS=Homo sapiens PE	2	103.1	103.6	97.4	106.3	103.35	101.85	0.985486212	0.948793
K7ERV3	TK1	Thymidine kinase OS=Homo sapiens GN=TK	4	99.8	100	100.3	96.6	99.9	98.45	0.985485485	0.948804
O43665	RGS10	Regulator of G-protein signaling 10 OS=Hom	3	98.7	94.1	93.5	96.5	96.4	95	0.985477178	0.948924
P35080	PFN2	Profilin-2 OS=Homo sapiens GN=PFN2 PE=	1	92.7	106.6	96.1	100.3	99.65	98.2	0.985449072	0.94933
Q96J84	KIRREL	Kin of IRRE-like protein 1 OS=Homo sapiens	4	108.9	96.7	99.6	103	102.8	101.3	0.98540856	0.949915

Q5UIP0	RIF1	Telomere-associated protein RIF1 OS=Homo	35	99.9	98.6	99	96.6	99.25	97.8	0.985390428	0.950177
Q96T51	RUFY1	RUN and FYVE domain-containing protein 1	3	100.3	104.3	101.1	100.5	102.3	100.8	0.985337243	0.950945
P25788	PSMA3	Proteasome subunit alpha type-3 OS=Homo s	13	102.9	101.5	101.8	99.6	102.2	100.7	0.985322896	0.951152
A0A024RDF6	HNRPDL	Heterogeneous nuclear ribonucleoprotein D-li	14	98.1	99.2	98	96.4	98.65	97.2	0.985301571	0.95146
O75529	TAF5L	TAF5-like RNA polymerase II p300/CBP-ass	2	107.2	96.9	105.2	95.9	102.05	100.55	0.985301323	0.951464
Q15527	SURF2	Surfeit locus protein 2 OS=Homo sapiens GN	1	103.2	100.2	102.9	97.5	101.7	100.2	0.985250737	0.952195
P18031	PTPN1	Tyrosine-protein phosphatase non-receptor ty	19	102.5	100.9	102.5	97.9	101.7	100.2	0.985250737	0.952195
Q8IWZ3	ANKHD1	Ankyrin repeat and KH domain-containing pr	3	100	103.1	101.4	98.7	101.55	100.05	0.985228951	0.952509
O14646	CHD1	Chromodomain-helicase-DNA-binding protei	12	102	100.4	99.7	99.7	101.2	99.7	0.985177866	0.953247
Q13049	TRIM32	E3 ubiquitin-protein ligase TRIM32 OS=Hom	1	101.4	94.2	97.5	95.2	97.8	96.35	0.985173824	0.953306
Q7Z7E8	UBE2Q1	Ubiquitin-conjugating enzyme E2 Q1 OS=Ho	6	101.9	100.2	99	100.1	101.05	99.55	0.985155863	0.953565
Q9BZJ0	CRNKL1	Crooked neck-like protein 1 OS=Homo sapier	22	101.1	100.9	99.3	99.7	101	99.5	0.985148515	0.953672
O60701	UGDH	UDP-glucose 6-dehydrogenase OS=Homo sap	7	100.6	100.5	98.2	99.9	100.55	99.05	0.985082049	0.954632
P11172	UMPS	Uridine 5'-monophosphate synthase OS=Hom	18	101.1	99.9	99.3	98.7	100.5	99	0.985074627	0.954739
O14639	ABLIM1	Actin-binding LIM protein 1 OS=Homo sapie	1	95.4	98.3	95.5	95.3	96.85	95.4	0.985028394	0.955407
D4QEZ8	ACADS	Short-chain acyl-CoA dehydrogenase OS=Ho	9	100.5	99.3	100.4	96.4	99.9	98.4	0.984984985	0.956035
Q6P161	MRPL54	39S ribosomal protein L54, mitochondrial OS	2	98.2	101.4	98.4	98.2	99.8	98.3	0.98496994	0.956253
A0A024R5F9	B3GNT6	UDP-GlcNAc:betaGal beta-1,3-N-acetylgluco	2	114.3	91.7	94	108.9	103	101.45	0.984951456	0.95652
Q3ZAQ7	VMA21	Vacuolar ATPase assembly integral membran	1	102.5	96.3	98.4	97.4	99.4	97.9	0.984909457	0.957126
Q9UFC0	LRWD1	Leucine-rich repeat and WD repeat-containing	14	103.1	101.4	100.6	100.8	102.25	100.7	0.984841076	0.958116
P86790	CCZ1B	Vacuolar fusion protein CCZ1 homolog B OS	5	100.4	97.5	95.4	99.5	98.95	97.45	0.984840829	0.958119
Q32Q12	NME1-NME2	Nucleoside diphosphate kinase OS=Homo sap	7	95.8	101.8	99.1	95.5	98.8	97.3	0.984817814	0.958452
Q5SZL2	CEP85L	Centrosomal protein of 85 kDa-like OS=Hom	1	98.7	105	100.6	100	101.85	100.3	0.984781541	0.958976
O14776	TCERG1	Transcription elongation regulator 1 OS=Hom	19	103.6	100	100.8	99.7	101.8	100.25	0.984774067	0.959085
A0A0G2JJD3	ABHD16A	HLA-B associated transcript 5, isoform CRA_	7	97.7	105.5	97.3	102.8	101.6	100.05	0.984744094	0.959518
Q6ZRD3		cDNA FLJ44718 fis, clone BRACE3022051 (	2	105.2	104	102.6	103.4	104.6	103	0.984703633	0.960103
H0Y9X1	TMA16	Translation machinery-associated protein 16 (	5	101.5	101.1	101.4	98.1	101.3	99.75	0.984698914	0.960172
A0A161I202	LTF	Lactoferrin OS=Homo sapiens GN=LTF PE=	1	102.3	93.5	96.6	96.2	97.9	96.4	0.984678243	0.96047
O60568	PLOD3	Procollagen-lysine,2-oxoglutarate 5-dioxygen	14	102.2	99.7	97.9	100.9	100.95	99.4	0.984645864	0.960939
A0A024QYY3	PRPSAP2	Phosphoribosyl pyrophosphate synthetase-ass	4	99.2	96	95.1	97.1	97.6	96.1	0.984631148	0.961152
Q9UGM6	WARS2	Tryptophan--tRNA ligase, mitochondrial OS=	4	104.9	96.4	98.8	99.4	100.65	99.1	0.984600099	0.9616
Q969T7	NT5C3B	7-methylguanosine phosphate-specific 5'-nucl	2	104.4	96.6	88.8	109.1	100.5	98.95	0.984577114	0.961933
Q96I99	SUCLG2	Succinate--CoA ligase [GDP-forming] subuni	18	101.6	99.3	99.9	97.9	100.45	98.9	0.984569438	0.962044
Q4U2R6	MRPL51	39S ribosomal protein L51, mitochondrial OS	1	97.6	96.1	101.8	88.9	96.85	95.35	0.984512132	0.962873
A0A024R1A3	UBE1	Testicular secretory protein Li 63 OS=Homo s	42	96.9	103	98.2	98.6	99.95	98.4	0.984492246	0.963161
P07900	HSP90AA1	Heat shock protein HSP 90-alpha OS=Homo s	30	97.9	101.9	97.6	99.1	99.9	98.35	0.984484484	0.963273
S4R2Z7	MRPL42	39S ribosomal protein L42, mitochondrial OS	2	101	98.3	96.5	99.7	99.65	98.1	0.984445559	0.963836
H0Y8X4	DNPH1	2'-deoxynucleoside 5'-phosphate N-hydrolase	2	96.9	95.7	96.1	93.5	96.3	94.8	0.984423676	0.964153
A0A024QZF2	RRAS	Related RAS viral (R-ras) oncogene homolog	3	102.7	102.4	101.1	100.8	102.55	100.95	0.984397855	0.964527
Q6PCB8	EMB	Embigin OS=Homo sapiens GN=EMB PE=1	1	107.6	97.3	101.4	100.3	102.45	100.85	0.984382626	0.964747
H7C2I1	PRMT1	Protein arginine N-methyltransferase 1 OS=H	8	101.5	102.9	99.1	102.1	102.2	100.6	0.984344423	0.9653
G3V5Z7	PSMA6	Proteasome subunit alpha type OS=Homo sap	13	101.7	102.4	101.3	99.6	102.05	100.45	0.984321411	0.965633
A0A140VJK2		Glycerol-3-phosphate dehydrogenase OS=Ho	29	103.1	100.8	100.3	100.4	101.95	100.35	0.984306032	0.965856
Q9HBB9		HC56 OS=Homo sapiens PE=4 SV=1	7	104.1	99.6	101.3	99.2	101.85	100.25	0.984290623	0.966079
P60174	TPI1	Triosephosphate isomerase OS=Homo sapiens	18	95.2	101.9	95.2	98.8	98.55	97	0.984271943	0.966349
D3DVW9	PTPNS1	Protein tyrosine phosphatase, non-receptor typ	7	103.4	100	99.9	100.3	101.7	100.1	0.984267453	0.966414
A0A0B4J1S8	PI4KB	Phosphatidylinositol 4-kinase beta OS=Homo	4	101.3	101.5	97.1	102.5	101.4	99.8	0.984220907	0.967088
P62826	RAN	GTP-binding nuclear protein Ran OS=Homo s	2	96.8	99.3	95.6	97.4	98.05	96.5	0.984191739	0.96751
Q8N0S6	CENPL	Centromere protein L OS=Homo sapiens GN=	1	107.2	100.2	94.4	109.7	103.7	102.05	0.984088717	0.969002
Q9Y6A5	TACC3	Transforming acidic coiled-coil-containing pr	4	98.8	102.1	95.2	102.5	100.45	98.85	0.984071677	0.969248
A8K6U7		cDNA FLJ78252, highly similar to Homo sap	14	100.7	100.1	99.1	98.5	100.4	98.8	0.984063745	0.969363
Q96PC5	MIA2	Melanoma inhibitory activity protein 2 OS=H	14	101.2	99.4	98.8	98.6	100.3	98.7	0.984047856	0.969594
P50443	SLC26A2	Sulfate transporter OS=Homo sapiens GN=SI	1	87.1	119.7	111.8	91.7	103.4	101.75	0.984042553	0.96967
A0A0S2Z5L1	ABCF3	ATP-binding cassette sub-family F member 3	7	101	99.4	97.2	100	100.2	98.6	0.984031936	0.969824
Q03701	CEBPZ	CCAAT/enhancer-binding protein zeta OS=H	28	101.4	98.7	98.4	98.5	100.05	98.45	0.984007996	0.97017
Q6NXT1	ANKRD54	Ankyrin repeat domain-containing protein 54	1	101.3	80	84.6	93.8	90.65	89.2	0.984004413	0.970222
Q8TDN6	BRIX1	Ribosome biogenesis protein BRX1 homolog	10	99.3	100.5	99.8	96.8	99.9	98.3	0.983983984	0.970518
Q9UJX2	CDC23	Cell division cycle protein 23 homolog OS=H	8	100.3	99.4	98.8	97.7	99.85	98.25	0.983975964	0.970635
D3DSW3	PROSC	Proline synthetase co-transcribed homolog (B	6	101.3	103.2	100.7	100.5	102.25	100.6	0.983863081	0.97227
P35268	RPL22	60S ribosomal protein L22 OS=Homo sapiens	4	99.9	98.4	95.7	99.4	99.15	97.55	0.983862834	0.972273
A0AV96	RBM47	RNA-binding protein 47 OS=Homo sapiens G	5	99.6	98.1	100.3	94.2	98.85	97.25	0.983813859	0.972982
P46109	CRKL	Crk-like protein OS=Homo sapiens GN=CRK	6	101.7	102.1	98.6	101.9	101.9	100.25	0.983807655	0.973072
Q8WWQ0	PHIP	PH-interacting protein OS=Homo sapiens GN	15	97	100.6	92.3	102.1	98.8	97.2	0.983805668	0.973101
Q16531	DDB1	DNA damage-binding protein 1 OS=Homo sa	36	101.2	102.4	101.3	99	101.8	100.15	0.983791749	0.973302
Q02790	FKBP4	Peptidyl-prolyl cis-trans isomerase FKBP4 OS	24	100.6	102.6	99.4	100.5	101.6	99.95	0.983759843	0.973765
Q86U28	ISCA2	Iron-sulfur cluster assembly 2 homolog, mitoc	5	98.8	98.2	96.9	96.9	98.5	96.9	0.983756345	0.973815
Q8NFH9		MLL/SEPTIN6 fusion protein (Fragment) OS	4	101.6	101.4	97.7	102	101.5	99.85	0.983743842	0.973996
A8K7F3		cDNA FLJ75847, highly similar to Homo sap	1	98.2	98.5	97.4	96.1	98.35	96.75	0.983731571	0.974174
Q9NWK9	ZNHIT6	Box C/D snoRNA protein 1 OS=Homo sapier	3	97.5	98.5	94.6	98.2	98	96.4	0.983673469	0.975016
A0A140VKE1		Testis tissue sperm-binding protein Li 58p OS	7	101.2	100.7	99	99.6	100.95	99.3	0.983655275	0.97528
Q6ZW49	PAXIP1	PAX-interacting protein 1 OS=Homo sapiens	3	102.3	99.5	104.7	93.8	100.9	99.25	0.983647175	0.975397
Q8NBI6	XXYLT1	Xyloside xylosyltransferase 1 OS=Homo sapi	2	105.6	95.7	97.8	100.2	100.65	99	0.983606557	0.975985
A0A096LPC5	FAM21C	WASH complex subunit FAM21C OS=Homo	1	104.1	91	89.1	102.8	97.55	95.95	0.983598155	0.976107
Q53F76		Polymerase (RNA) III (DNA directed) polype	7	101.5	99.6	98.3	99.5	100.55	98.9	0.983590254	0.976222
P63104	YWHAZ	14-3-3 protein zeta/delta OS=Homo sapiens G	12	100.9	100.2	98.8	99	100.55	98.9	0.983590254	0.976222
O95999	BCL10	B-cell lymphoma/leukemia 10 OS=Homo sap	1	100.9	100.1	96.8	100.9	100.5	98.85	0.98358209	0.97634
P32321	DCTD	Deoxycytidylate deaminase OS=Homo sapien	4	97.6	103.3	98.3	99.3	100.45	98.8	0.983573917	0.976459
Q96IX5	USMG5	Up-regulated during skeletal muscle growth p	2	98.9	101.7	97.7	99.6	100.3	98.65	0.983549352	0.976815
Q9NRR5	UBQLN4	Ubiquilin-4 OS=Homo sapiens GN=UBQLN4	5	97.6	102.2	93.9	102.6	99.9	98.25	0.983483483	0.977769
B4DLM8		cDNA FLJ56105, highly similar to Nuclear va	17	100.4	99.3	96.9	99.5	99.85	98.2	0.983475213	0.977889
A8K329		cDNA FLJ76656, highly similar to Homo sap	19	101.5	98.2	98.1	98.3	99.85	98.2	0.983475213	0.977889
Q7L4I2	RSRC2	Arginine/serine-rich coiled-coil protein 2 OS=	3	97.8	101.3	98.6	97.2	99.55	97.9	0.983425414	0.978611
A0A024R8Q1	GAA	Glucosidase, alpha acid (Pompe disease, glyco	1	103.8	101.3	92.3	109.4	102.55	100.85	0.983422721	0.97865
Q9HBB3		60S ribosomal protein L6 OS=Homo sapiens	19	101.5	103.3	102.5	98.9	102.4	100.7	0.983398438	0.979002
Q86VX4	SMC3	Structural maintenance of chromosomes prote	44	103.3	101.2	99	102.1	102.25	100.55	0.983374083	0.979355
Q16715		Pyruvate kinase (Fragment) OS=Homo sapien	1	102.5	101.5	104	96.6	102	100.3	0.983333333	0.979946
V9HWJ9	HEL97	Epididymis luminal protein 97 OS=Homo sap	3	96.9	100.8	99.5	94.9	98.85	97.2	0.983308042	0.980313
Q9Y3U8	RPL36	60S ribosomal protein L36 OS=Homo sapiens	5	99.3	104.3	100.4	99.8	101.8	100.1	0.983300589	0.980421
B4DJ30		cDNA FLJ61290, highly similar to Neutral al	36	102.5	101	100.7	99.4	101.75	100.05	0.983292383	0.980539
A0A0S2Z6K1	HNRPLL	Heterogeneous nuclear ribonucleoprotein L-li	16	102.3	100.9	100.2	99.6	101.6	99.9	0.983267717	0.980897
Q9NS00	C1GALT1	Glycoprotein-N-acetylgalactosamine 3-beta-g	1	100.2	103	101.7	98.1	101.6	99.9	0.983267717	0.980897
Q5BKZ1	ZNF326	DBIRD complex subunit ZNF326 OS=Homo	11	102.3	100.3	100.2	99	101.3	99.6	0.983218164	0.981615
A8K8U1		cDNA FLJ77762, highly similar to Homo sap	26	101.5	101.1	100.7	98.5	101.3	99.6	0.983218164	0.981615
Q86XL3	ANKLE2	Ankyrin repeat and LEM domain-containing p	8	103.4	93.2	97.8	95.5	98.3	96.65	0.983214649	0.981666
Q53GI2		Testis expressed sequence 264 variant (Fragm	9	102.5	100	99.1	100	101.25	99.55	0.983209877	0.981736
A8K0Z3	WASH1	WAS protein family homolog 1 OS=Homo sa	1	101.1	106.3	96.8	107.1	103.7	101.95	0.983124397	0.982975
B7Z2R7	ACBD5	Acyl-CoA-binding domain-containing protein	8	101.8	98.7	99	98.1	100.25	98.55	0.983042394	0.984164
P82914	MRPS15	28S ribosomal protein S15, mitochondrial OS	8	99.5	94.5	96.5	94.2	97	95.35	0.982989691	0.984929
P13640	MT1G	Metallothionein-1G OS=Homo sapiens GN=M	1	95.8	98.1	88.3	102.3	96.95	95.3	0.982980918	0.985056
Q8IXT5	RBM12B	RNA-binding protein 12B OS=Homo sapiens	22	100.6	99.1	99.2	97.1	99.85	98.15	0.982974462	0.985149
Q9Y3C6	PPIL1	Peptidyl-prolyl cis-trans isomerase-like 1 OS=	5	102.8	102.6	103.8	98.1	102.7	100.95	0.982960078	0.985358
Q8WWI5	SLC44A1	Choline transporter-like protein 1 OS=Homo s	7	106.6	98.6	102.2	99.5	102.6	100.85	0.98294347	0.985599
Q9H582	ZNF644	Zinc finger protein 644 OS=Homo sapiens GN	3	99	100.1	97.9	97.8	99.55	97.85	0.982923154	0.985894
O00161	SNAP23	Synaptosomal-associated protein 23 OS=Hom	9	106	98.7	101	100.2	102.35	100.6	0.982901808	0.986203
Q7Z4G1	COMMD6	COMM domain-containing protein 6 OS=Hor	2	102.3	101.9	95.2	105.5	102.1	100.35	0.982859941	0.98681
A0A1B0GTB0	ATP6AP2	Renin receptor (Fragment) OS=Homo sapiens	8	100.9	103.3	100	100.7	102.1	100.35	0.982859941	0.98681
A0MNP2	WDR57	CDW11/WDR57 OS=Homo sapiens GN=WE	13	99.9	98.4	99.7	95.2	99.15	97.45	0.982854261	0.986893
Q8NCD3	HJURP	Holliday junction recognition protein OS=Hor	2	99.3	104.8	105.9	94.7	102.05	100.3	0.982851543	0.986932
Q92878	RAD50	DNA repair protein RAD50 OS=Homo sapier	37	102	101.3	100.3	99.5	101.65	99.9	0.982784063	0.987911
Q6NUK4	REEP3	Receptor expression-enhancing protein 3 OS=	3	102.4	100.8	97.3	102.4	101.6	99.85	0.982775591	0.988035
A8K7H4		Origin recognition complex subunit 4 OS=Ho	6	103.2	99.6	100.8	98.5	101.4	99.65	0.982741617	0.988528
P41208	CETN2	Centrin-2 OS=Homo sapiens GN=CETN2 PE	2	100.1	96.7	97	96.4	98.4	96.7	0.982723577	0.988789
A0A024R1M6	MGC3731	Uncharacterized protein OS=Homo sapiens G	3	94.9	96.1	93.9	93.8	95.5	93.85	0.982722513	0.988804
P28070	PSMB4	Proteasome subunit beta type-4 OS=Homo sa	8	100.6	101.2	98.9	99.4	100.9	99.15	0.982656095	0.989768
B4DDB6	HNRPA3	Heterogeneous nuclear ribonucleoprotein A3,	1	99.3	90.9	95.7	91.2	95.1	93.45	0.982649842	0.989859
A8K9T8		cDNA FLJ76106, highly similar to Homo sap	8	100.8	100	98	99.3	100.4	98.65	0.982569721	0.991022
A0A087WY85	UBE2D3	Ubiquitin-conjugating enzyme E2 D3 OS=Ho	3	99.2	107.3	97.9	105	103.25	101.45	0.982566586	0.991067
Q969X5	ERGIC1	Endoplasmic reticulum-Golgi intermediate co	4	97.7	102.9	99.6	97.5	100.3	98.55	0.982552343	0.991274
Q8TB36	GDAP1	Ganglioside-induced differentiation-associate	3	103.1	97.2	95.8	101	100.15	98.4	0.982526211	0.991653
Q4J6C6	PREPL	Prolyl endopeptidase-like OS=Homo sapiens	4	103.2	97.1	94.7	102.1	100.15	98.4	0.982526211	0.991653
Q32Q10	RSU1	RSU1 protein (Fragment) OS=Homo sapiens	8	99.3	100.4	98.7	97.5	99.85	98.1	0.982473711	0.992415
Q9P2R3	ANKFY1	Rabankyrin-5 OS=Homo sapiens GN=ANKF	9	102	103.2	100.9	100.7	102.6	100.8	0.98245614	0.99267

K7ENT6	TPM4	Tropomyosin alpha-4 chain (Fragment) OS=H	1	103.7	95.8	102.2	93.8	99.75	98	0.98245614	0.99267
Q6VMQ6	ATF7IP	Activating transcription factor 7-interacting pr	3	100.9	103.9	97.4	103.8	102.4	100.6	0.982421875	0.993167
Q01970	PLCB3	1-phosphatidylinositol 4,5-bisphosphate phosp	5	104.3	100.3	100.7	100.3	102.3	100.5	0.982404692	0.993416
Q9UGR2	ZC3H7B	Zinc finger CCCH domain-containing protein	7	101.3	97.5	96.8	98.5	99.4	97.65	0.982394366	0.993567
P62917	RPL8	60S ribosomal protein L8 OS=Homo sapiens	14	101.3	103.1	100.1	100.7	102.2	100.4	0.982387476	0.993666
B3KMR4		cDNA FLJ12419 fis, clone MAMMA100304	2	100.6	97.9	95.7	99.3	99.25	97.5	0.982367758	0.993953
Q9H479	FN3K	Fructosamine-3-kinase OS=Homo sapiens GN	5	94.9	97.9	92.3	97.1	96.4	94.7	0.982365145	0.993991
A0A024R658	ZFP36L1	Zinc finger protein 36, C3H type-like 1, isofo	1	121.3	82.2	101.4	98.5	101.75	99.95	0.982309582	0.994797
O75475	PSIP1	PC4 and SFRS1-interacting protein OS=Hom	16	105.1	98.2	99.6	100.1	101.65	99.85	0.982292179	0.99505
Q5U676	B3GAT3	B3GAT3 protein (Fragment) OS=Homo sapie	3	99.9	97.7	96.1	98	98.8	97.05	0.982287449	0.995118
A8K7J6		cDNA FLJ76418, highly similar to Homo sap	3	101.1	101.8	102.3	97	101.45	99.65	0.98225727	0.995557
A0A0S2Z3W7	ITPA	Nucleotide diphosphatase (Fragment) OS=Ho	4	101.3	95.9	92.8	100.9	98.6	96.85	0.982251521	0.99564
A8IK34	ANKRD40	Ankyrin repeat domain 40 OS=Homo sapiens	1	101.7	100.9	97	102	101.3	99.5	0.982230997	0.995938
A0A024RDI6	NUDT9	Nudix (Nucleoside diphosphate linked moiety	2	99.7	102.7	97.6	101.2	101.2	99.4	0.982213439	0.996193
Q9BTD8	RBM42	RNA-binding protein 42 OS=Homo sapiens G	6	100.6	95.6	94.9	97.8	98.1	96.35	0.98216106	0.996954
Q15386	UBE3C	Ubiquitin-protein ligase E3C OS=Homo sapie	9	105.1	102	104.4	99	103.55	101.7	0.982134235	0.997343
Q12788	TBL3	Transducin beta-like protein 3 OS=Homo sap	23	102.2	99.1	98.5	99.2	100.65	98.85	0.982116244	0.997604
Q6ZU65	UBN2	Ubinuclein-2 OS=Homo sapiens GN=UBN2	1	103	98.1	96.4	101.1	100.55	98.75	0.982098458	0.997862
Q9HB40	SCPEP1	Retinoid-inducible serine carboxypeptidase O	5	105.2	95.8	98.9	98.5	100.5	98.7	0.982089552	0.997991
E7EVH7		Uncharacterized protein OS=Homo sapiens Pl	11	100.2	99.9	97.6	98.9	100.05	98.25	0.982008996	0.999161
Q01813	PFKP	ATP-dependent 6-phosphofructokinase, platel	24	100	99.8	99.2	97	99.9	98.1	0.981981982	0.999553
B2RB94		cDNA, FLJ95380, highly similar to Homo sap	3	105.2	99.9	101.6	99.8	102.55	100.7	0.98196002	0.999873
Q92499	DDX1	ATP-dependent RNA helicase DDX1 OS=Ho	32	103.7	101.3	101.7	99.6	102.5	100.65	0.98195122	1
Q8N9E0	FAM133A	Protein FAM133A OS=Homo sapiens GN=F/	1	103.4	107	103.2	103.4	105.2	103.3	0.981939163	0.99984
A0A0C4DFN1	MFN1	Mitofusin-1 OS=Homo sapiens GN=MFN1 P	11	103.4	95.8	97	98.6	99.6	97.8	0.981927711	0.999688
Q5JSZ5	PRRC2B	Protein PRRC2B OS=Homo sapiens GN=PRI	1	106.1	98.5	100.2	100.7	102.3	100.45	0.981915934	0.999533
P46779	RPL28	60S ribosomal protein L28 OS=Homo sapiens	11	102.1	102.4	102.7	98.1	102.25	100.4	0.98190709	0.999415
B4DZF2		cDNA FLJ59571, highly similar to Eukaryoti	32	103.7	100.6	99.7	100.9	102.15	100.3	0.981889378	0.999181
O75394	MRPL33	39S ribosomal protein L33, mitochondrial OS	2	102.4	101.9	100.9	99.7	102.15	100.3	0.981889378	0.999181
Q2WGJ9	FER1L6	Fer-1-like protein 6 OS=Homo sapiens GN=F	1	102.1	96.6	101.5	93.6	99.35	97.55	0.981882235	0.999086
Q8TDY2	RB1CC1	RB1-inducible coiled-coil protein 1 OS=Hom	1	98	100.6	97	98	99.3	97.5	0.981873112	0.998966
Q9NYU2	UGGT1	UDP-glucose:glycoprotein glucosyltransfera	38	103.3	100.8	101.5	98.9	102.05	100.2	0.981871632	0.998946
V9GZZ7		Uncharacterized protein OS=Homo sapiens Pl	1	106.8	102.4	104.8	100.6	104.6	102.7	0.981835564	0.998468
P49959	MRE11A	Double-strand break repair protein MRE11A	20	102.2	101.4	100.5	99.4	101.8	99.95	0.981827112	0.998357
Q7Z2W9	MRPL21	39S ribosomal protein L21, mitochondrial OS	7	104.4	98.9	102.7	96.9	101.65	99.8	0.981800295	0.998002
Q14232	EIF2B1	Translation initiation factor eIF-2B subunit al	10	102.4	100.8	100.6	98.9	101.6	99.75	0.981791339	0.997883
Q9UPT5	EXOC7	Exocyst complex component 7 OS=Homo sap	9	101.4	101.8	99	100.5	101.6	99.75	0.981791339	0.997883
A0A0G2JPP5	SCRIB	Protein scribble homolog OS=Homo sapiens	19	103.5	99.2	97.6	101.4	101.35	99.5	0.981746423	0.997289
Q9ULC4	MCTS1	Malignant T-cell-amplified sequence 1 OS=H	5	100	102.7	100.1	98.9	101.35	99.5	0.981746423	0.997289
Q9HA77	CARS2	Probable cysteine--tRNA ligase, mitochondri	7	99.1	92.4	93.2	94.8	95.75	94	0.981723238	0.996982
A0A024R2F8	CHCHD4	Coiled-coil-helix-coiled-coil-helix domain co	1	98.5	98.4	94.8	98.5	98.45	96.65	0.981716607	0.996894
A0A1B0GTU	PXN	Paxillin OS=Homo sapiens GN=PXN PE=1 S	3	106.3	95.7	98.6	99.7	101	99.15	0.981683168	0.996451
A8K6A7		Alpha-mannosidase OS=Homo sapiens PE=2	13	102.8	99.1	99.9	98.3	100.95	99.1	0.981674096	0.99633
O95373	IPO7	Importin-7 OS=Homo sapiens GN=IPO7 PE=	25	101.9	100	99.5	98.7	100.95	99.1	0.981674096	0.99633
Q5T5P2	KIAA1217	Sickle tail protein homolog OS=Homo sapien	1	102.8	93.5	94.9	97.8	98.15	96.35	0.981660723	0.996154
Q9BYD6	MRPL1	39S ribosomal protein L1, mitochondrial OS=	9	101.1	100.5	99.8	98.1	100.8	98.95	0.981646825	0.99597
Q8NHZ8	CDC26	Anaphase-promoting complex subunit CDC26	2	88.2	85.4	81.3	89.1	86.8	85.2	0.98156682	0.99491
Q5VZL5	ZMYM4	Zinc finger MYM-type protein 4 OS=Homo s	15	101.1	99.6	100	97	100.35	98.5	0.981564524	0.99488
Q5STK2	PFDN6	Prefoldin subunit 6, isoform CRA_b OS=Hon	7	99.4	101.1	99	97.8	100.25	98.4	0.981546135	0.994637
O75146	HIP1R	Huntingtin-interacting protein 1-related protei	7	102.7	103.2	99.5	102.6	102.95	101.05	0.981544439	0.994614
P39023	RPL3	60S ribosomal protein L3 OS=Homo sapiens	4	101.1	104.7	102.2	99.8	102.9	101	0.981535471	0.994495
J3KPD3	RBM7	RNA binding motif protein 7, isoform CRA_c	5	102.3	103.4	103.1	98.8	102.85	100.95	0.981526495	0.994376
Q96IW7	SEC22A	Vesicle-trafficking protein SEC22a OS=Hom	3	103	102.6	102.6	99.2	102.8	100.9	0.98151751	0.994257
Q6PIY7	PAPD4	Poly(A) RNA polymerase GLD2 OS=Homo s	8	102.4	97.5	96.2	100	99.95	98.1	0.981490745	0.993902
Q8TDW0	LRRC8C	Volume-regulated anion channel subunit LRR	2	101.8	98	92.2	103.9	99.9	98.05	0.981481481	0.99378
Q99622	C12orf57	Protein C10 OS=Homo sapiens GN=C12orf5	3	96.7	102.6	94.7	100.9	99.65	97.8	0.981435023	0.993164
Q9H4L5	OSBPL3	Oxysterol-binding protein-related protein 3 O	8	98.5	100.2	95.6	99.4	99.35	97.5	0.981378963	0.992422
Q147X3	NAA30	N-alpha-acetyltransferase 30 OS=Homo sapie	4	100.3	98.3	96.1	98.8	99.3	97.45	0.981369587	0.992298
A0A024R9W8	SLC30A9	Solute carrier family 30 (Zinc transporter), me	1	102.2	96.4	92.4	102.5	99.3	97.45	0.981369587	0.992298
Q9NUL7	DDX28	Probable ATP-dependent RNA helicase DDX	7	102.6	95.9	93.4	101.4	99.25	97.4	0.981360202	0.992174
Q6FG99	RPLP1	RPLP1 protein OS=Homo sapiens GN=RPLP	3	104.3	99.5	99.7	100.3	101.9	100	0.981354269	0.992095
Q96JM3	CHAMP1	Chromosome alignment-maintaining phospho	12	97.1	95.8	94.3	95	96.45	94.65	0.981337481	0.991873
P55201	BRPF1	Peregrin OS=Homo sapiens GN=BRPF1 PE=	1	95.7	97.1	90.9	98.3	96.4	94.6	0.981327801	0.991744
Q15393	SF3B3	Splicing factor 3B subunit 3 OS=Homo sapier	42	104.4	97.9	101.4	97.1	101.15	99.25	0.981216016	0.990264
A0A024R3U9	RAB4A	RAB4A, member RAS oncogene family, isof	6	102.7	99.3	99.5	98.7	101	99.1	0.981188119	0.989894
B0YJ88	RDX	Radixin OS=Homo sapiens GN=RDX PE=2 S	18	100.7	101.3	99.4	98.8	101	99.1	0.981188119	0.989894
V9HW80	HEL-S-70	Epididymis luminal protein 220 OS=Homo sa	45	100.1	101.3	98.2	99.4	100.7	98.8	0.981132075	0.989152
A0A024RC48	GALNT1	Polypeptide N-acetylgalactosaminyltransferas	1	102.7	103.9	106.6	96.1	103.3	101.35	0.981122943	0.989031
A8MWD9	SNRPGP15	Putative small nuclear ribonucleoprotein G-lil	4	99.3	101.9	101.5	95.9	100.6	98.7	0.98111332	0.988903
Q53G19		Mitochondrial ribosomal protein L11 isoform	10	102.4	98.6	100.4	96.8	100.5	98.6	0.981094527	0.988654
B2R825		Alpha-1,4 glucan phosphorylase OS=Homo sa	17	101.2	99.8	98.9	98.3	100.5	98.6	0.981094527	0.988654
A0A024R2K4	LRRFIP2	Leucine rich repeat (In FLII) interacting prote	2	102.2	98.4	95.7	101.1	100.3	98.4	0.98105683	0.988155
Q9H2V7	SPNS1	Protein spinster homolog 1 OS=Homo sapiens	3	105.2	95.2	97.7	98.9	100.2	98.3	0.981037924	0.987904
B3KTS4		cDNA FLJ38665 fis, clone HLUNG2003378,	1	98.3	101.9	88.1	108.3	100.1	98.2	0.981018981	0.987653
A0A140VK69		Aspartate aminotransferase OS=Homo sapien	15	96.9	97.9	94.6	96.5	97.4	95.55	0.98100616	0.987483
O95619	YEATS4	YEATS domain-containing protein 4 OS=Hon	1	100.3	104.9	97	104.3	102.6	100.65	0.980994152	0.987325
Q9BRX8	FAM213A	Redox-regulatory protein FAM213A OS=Hon	6	104.2	100.7	102.7	98.3	102.45	100.5	0.980966325	0.986956
Q9Y4E8	USP15	Ubiquitin carboxyl-terminal hydrolase 15 OS	3	97.4	102.1	96.4	99.3	99.75	97.85	0.980952381	0.986771
P62847	RPS24	40S ribosomal protein S24 OS=Homo sapiens	7	99.9	104.5	101.9	98.6	102.2	100.25	0.980919765	0.986339
O14907	TAX1BP3	Tax1-binding protein 3 OS=Homo sapiens GN	3	104.7	94.2	99.4	95.7	99.45	97.55	0.980894922	0.98601
Q9BZ95	WHSC1L1	Histone-lysine N-methyltransferase NSD3 OS	5	102.7	95.8	100.2	94.5	99.25	97.35	0.980856423	0.985499
B2RD09		cDNA, FLJ96406, highly similar to Homo sap	18	102.8	100.8	99.5	100.2	101.8	99.85	0.980844794	0.985345
Q6UX04	CWC27	Peptidyl-prolyl cis-trans isomerase CWC27 h	5	107.7	95.5	98.1	101.2	101.6	99.65	0.980807087	0.984845
Q9NSB8	HOMER2	Homer protein homolog 2 OS=Homo sapiens	1	107.6	100.2	99.1	104.7	103.9	101.9	0.980750722	0.984099
Q96EX3	WDR34	WD repeat-containing protein 34 OS=Homo s	1	100	97.2	98.4	95	98.6	96.7	0.980730223	0.983827
B2R802		cDNA, FLJ93681, highly similar to Homo sap	8	100.8	101.5	98.1	100.3	101.15	99.2	0.9807217	0.983714
Q53H12	AGK	Acylglycerol kinase, mitochondrial OS=Hom	15	101.1	100.9	100.5	97.6	101	99.05	0.980693069	0.983334
A0A0S2Z3L2	ATP2A2	ATPase Ca++ transporting cardiac muscle slo	44	103.4	98.5	100.7	97.3	100.95	99	0.980683507	0.983208
Q6NVY1	HIBCH	3-hydroxyisobutyryl-CoA hydrolase, mitocho	12	99.7	97	97	95.9	98.35	96.45	0.98068124	0.983178
Q96SB8	SMC6	Structural maintenance of chromosomes prote	7	104.7	102.2	102.5	100.4	103.45	101.45	0.980666989	0.982989
Q9H0U4	RAB1B	Ras-related protein Rab-1B OS=Homo sapien	5	102.2	99.1	99.4	98	100.65	98.7	0.980625931	0.982445
Q5SWX8	ODR4	Protein odr-4 homolog OS=Homo sapiens GN	10	103.9	101.2	100.9	100.2	102.55	100.55	0.980497318	0.98074
A0A024R7X0	ARFGEF1	ADP-ribosylation factor guanine nucleotide-e	4	109.9	94.8	97.3	103.4	102.35	100.35	0.980459209	0.980235
A8K4Z6		cDNA FLJ76180 OS=Homo sapiens PE=2 SV	6	104.5	100.2	97.9	102.8	102.35	100.35	0.980459209	0.980235
V9HWD6	HEL-S-1	Epididymis secretory protein Li 1 OS=Homo	6	99.4	100	96.5	99	99.7	97.75	0.980441324	0.979998
O15347	HMGB3	High mobility group protein B3 OS=Homo sa	4	99.4	100	98.1	97.4	99.7	97.75	0.980441324	0.979998
Q9BTI9	NPM1	NPM1 protein (Fragment) OS=Homo sapiens	1	92.7	106.6	94.7	100.7	99.65	97.7	0.98043151	0.979868
Q15542	TAF5	Transcription initiation factor TFIID subunit 5	3	101.5	102.7	94.8	105.4	102.1	100.1	0.980411361	0.979601
A0A024R248	C9orf89	Chromosome 9 open reading frame 89, isofor	4	102.2	102	99.9	100.3	102.1	100.1	0.980411361	0.979601
Q96NN9	AIFM3	Apoptosis-inducing factor 3 OS=Homo sapier	1	101.5	92.4	99.3	90.8	96.95	95.05	0.980402269	0.979481
E7EVA0	MAP4	Microtubule-associated protein OS=Homo sap	29	103.3	100	100	99.3	101.65	99.65	0.980324643	0.978452
O15228	GNPAT	Dihydroxyacetone phosphate acyltransferase	8	105.5	97.7	101	98.2	101.6	99.6	0.980314961	0.978324
Q59HH3		Trifunctional purine biosynthetic protein aden	25	101	102	98.1	100.9	101.5	99.5	0.980295567	0.978066
P21675	TAF1	Transcription initiation factor TFIID subunit 1	2	105.1	97.3	100	98.4	101.2	99.2	0.980237154	0.977292
B0QY89	EIF3L	Eukaryotic translation initiation factor 3 subu	17	103.4	104	103.7	99.6	103.7	101.65	0.980231437	0.977217
B3KXY9		cDNA FLJ46359 fis, clone TESTI4049786, h	29	104	98.3	100	98.3	101.15	99.15	0.980227385	0.977163
Q5T0F3	GNL2	Guanine nucleotide binding protein-like 2 (Nu	18	101.8	100.4	99.9	98.3	101.1	99.1	0.980217606	0.977033
P17568	NDUFB7	NADH dehydrogenase [ubiquinone] 1 beta su	5	102.2	99.9	99.5	98.6	101.05	99.05	0.980207818	0.976903
Q9HCC0	MCCC2	Methylcrotonoyl-CoA carboxylase beta chain	16	99.5	102.5	98.9	99.1	101	99	0.98019802	0.976773
B2RB23		cDNA, FLJ95265, highly similar to Homo sap	19	100.6	101.1	98.3	99.4	100.85	98.85	0.980168567	0.976383
A0A024R2Q4	RPL15	Ribosomal protein L15 OS=Homo sapiens GN	13	101	105.7	103.7	98.9	103.35	101.3	0.98016449	0.976328
A0A024R250	NOL8	Nucleolar protein 8, isoform CRA_a OS=Hon	4	100.1	101.5	96.5	101.1	100.8	98.8	0.98015873	0.976253
B2RDG1		Fatty acyl-CoA reductase OS=Homo sapiens	11	97.2	104.3	104.3	93.2	100.75	98.75	0.980148883	0.976122
P10109	FDX1	Adrenodoxin, mitochondrial OS=Homo sapie	5	99.5	102	94.6	102.9	100.75	98.75	0.980148883	0.976122
P62244	RPS15A	40S ribosomal protein S15a OS=Homo sapier	4	100.4	106.1	99.4	103	103.25	101.2	0.980145278	0.976074
Q5J8M3	EMC4	ER membrane protein complex subunit 4 OS=	5	105.8	95.5	100.9	96.4	100.65	98.65	0.98012916	0.975861
Q9Y230	RUVBL2	RuvB-like 2 OS=Homo sapiens GN=RUVBL	27	100	101.2	98.7	98.5	100.6	98.6	0.980119284	0.97573
A8K690		cDNA FLJ76863, highly similar to Homo sap	42	100.4	100.7	99	98.1	100.55	98.55	0.980109398	0.975598
P49841	GSK3B	Glycogen synthase kinase-3 beta OS=Homo s	4	103.2	102.3	97	104.4	102.75	100.7	0.980048662	0.974793
A0A024R652	MTHFD1	Methylenetetrahydrofolate dehydrogenase (N.	38	99.8	100.4	98.4	97.8	100.1	98.1	0.98001998	0.974413
O60337	MARCH6	E3 ubiquitin-protein ligase MARCH6 OS=Ho	1	99.1	100.9	101.2	94.8	100	98	0.98	0.974148
Q9Y3F4	STRAP	Serine-threonine kinase receptor-associated pr	16	103.6	101.4	101.5	99.4	102.5	100.45	0.98	0.974148

Q9GZZ9	UBA5	Ubiquitin-like modifier-activating enzyme 5 (	4	103.4	101.4	100.1	100.6	102.4	100.35	0.979980469	0.973889
P61966	AP1S1	AP-1 complex subunit sigma-1A OS=Homo s	3	106.5	98	99.5	100.9	102.25	100.2	0.9799511	0.9735
Q6FHY4	NAPG	N-ethylmaleimide-sensitive factor attachment	5	101.5	98	97.3	98.2	99.75	97.75	0.979949875	0.973484
A0A024R7I0	GIPC1	GIPC PDZ domain containing family, membe	6	91.9	92.5	86.9	93.8	92.2	90.35	0.979934924	0.973285
A0A140VJX3		Sulfurtransferase OS=Homo sapiens PE=2 SV	8	99.3	99.7	98.1	96.9	99.5	97.5	0.979899497	0.972816
Q6FGU2	DTYMK	DTYMK protein (Fragment) OS=Homo sapie	11	100	103.8	100.6	99.1	101.9	99.85	0.979882237	0.972587
Q8NC56	LEMD2	LEM domain-containing protein 2 OS=Homo	9	104.3	99.1	99.9	99.4	101.7	99.65	0.979842675	0.972062
P67809	YBX1	Nuclease-sensitive element-binding protein 1	5	101.9	101.4	99	100.2	101.65	99.6	0.979832759	0.971931
Q13454	TUSC3	Tumor suppressor candidate 3 OS=Homo sapi	6	100.9	102.3	100.9	98.2	101.6	99.55	0.979822835	0.971799
Q8IWR0	ZC3H7A	Zinc finger CCCH domain-containing protein	7	101.6	101.5	97.6	101.4	101.55	99.5	0.9798129	0.971668
B4DDK9		cDNA FLJ55952, highly similar to Alpha-1,6	6	101.7	96.4	96	98.1	99.05	97.05	0.979808178	0.971605
B0YIW5	ARCN1	Archain 1, isoform CRA_b OS=Homo sapien	17	99.7	103.3	102.5	96.4	101.5	99.45	0.979802956	0.971536
B3KM97		cDNA FLJ10554 fis, clone NT2RP2002385, l	6	101	101.9	101.4	97.4	101.45	99.4	0.979793001	0.971404
Q549C5	MST065	HCG2010808, isoform CRA_a OS=Homo sap	6	101.3	100.5	98.2	99.5	100.9	98.85	0.979682854	0.969943
B2R5Y4		cDNA, FLJ92684, highly similar to Homo sap	13	101.7	99.8	100.2	97.2	100.75	98.7	0.979652605	0.969542
A0A024R5V1	TRPM7	Transient receptor potential cation channel, su	8	100.4	101	99	98.3	100.7	98.65	0.979642502	0.969408
Q13618	CUL3	Cullin-3 OS=Homo sapiens GN=CUL3 PE=1	20	101.1	100.2	96.9	100.3	100.65	98.6	0.979632389	0.969274
A0A024QZF1	hCG_19665	HCG19665, isoform CRA_a OS=Homo sapie	11	103	97.8	98.4	98.3	100.4	98.35	0.979581673	0.968601
Q5JWT2	MED23	Mediator of RNA polymerase II transcription	8	100.2	100.5	95.7	100.9	100.35	98.3	0.9795715	0.968467
P55884	EIF3B	Eukaryotic translation initiation factor 3 subu	27	105.2	100.3	101.3	100	102.75	100.65	0.979562044	0.968341
Q9H7L9	SUDS3	Sin3 histone deacetylase corepressor complex	3	97.3	98.3	94.8	96.8	97.8	95.8	0.979550102	0.968183
Q71N00		C75F OS=Homo sapiens PE=2 SV=1	1	107.5	102.1	100.3	105	104.8	102.65	0.979484733	0.967316
Q13620	CUL4B	Cullin-4B OS=Homo sapiens GN=CUL4B PE	6	101.1	98.6	96.7	98.9	99.85	97.8	0.979469204	0.96711
Q460N5	PARP14	Poly [ADP-ribose] polymerase 14 OS=Homo	8	105.5	94.1	97.6	97.9	99.8	97.75	0.979458918	0.966974
Q9NX20	MRPL16	39S ribosomal protein L16, mitochondrial OS	7	98	101.5	97.9	97.5	99.75	97.7	0.979448622	0.966837
Q96HY6	DDRGK1	DDRGK domain-containing protein 1 OS=Ho	4	101.4	97.8	96.9	98.2	99.6	97.55	0.979417671	0.966427
Q96HS1	PGAM5	Serine/threonine-protein phosphatase PGAM5	15	101.2	102.6	102.9	96.7	101.9	99.8	0.97939156	0.966081
Q9HCE5	METTL14	N6-adenosine-methyltransferase subunit MET	2	102.9	100.7	100.9	98.5	101.8	99.7	0.979371316	0.965812
Q5QJE6	DNTTIP2	Deoxynucleotidyltransferase terminal-interact	18	105.2	98.1	99.9	99.2	101.65	99.55	0.979340876	0.965408
Q9BZE1	MRPL37	39S ribosomal protein L37, mitochondrial OS	17	101.6	101.6	100.8	98.2	101.6	99.5	0.979330709	0.965274
Q53HB3		Proteasome 26S ATPase subunit 1 variant (Fr	19	100.2	102.6	99.1	99.5	101.4	99.3	0.979289941	0.964733
Q15084	PDIA6	Protein disulfide-isomerase A6 OS=Homo sap	22	101.1	101.5	99	99.4	101.3	99.2	0.979269497	0.964462
Q9BYD2	MRPL9	39S ribosomal protein L9, mitochondrial OS=	7	99.3	103.1	100.8	97.4	101.2	99.1	0.979249012	0.96419
P46734	MAP2K3	Dual specificity mitogen-activated protein kin	5	100.9	101.3	99	99	101.1	99	0.979228487	0.963918
Q9BR76	CORO1B	Coronin-1B OS=Homo sapiens GN=CORO1B	7	100.5	101.5	97.1	100.7	101	98.9	0.979207921	0.963646
Q9BTT0	ANP32E	Acidic leucine-rich nuclear phosphoprotein 32	6	103.7	98	98.8	98.7	100.85	98.75	0.979176996	0.963235
Q6AWA4	DKFZp686O08	Putative uncharacterized protein DKFZp686C	23	101.6	100.1	97.5	100	100.85	98.75	0.979176996	0.963235
O95059	RPP14	Ribonuclease P protein subunit p14 OS=Hom	3	101.2	105.3	104	98.2	103.25	101.1	0.979176755	0.963232
G1UI16	PDS5A	SCC-112 protein, isoform CRA_b OS=Homo	24	104.7	101.6	102.4	99.6	103.15	101	0.979156568	0.962964
Q9BY89	KIAA1671	Uncharacterized protein KIAA1671 OS=Hom	3	110.6	95.7	100.7	101.3	103.15	101	0.979156568	0.962964
O15020	SPTBN2	Spectrin beta chain, non-erythrocytic 2 OS=H	28	100.2	101.3	98	99.3	100.75	98.65	0.979156328	0.962961
A0A024R9N6	EHD4	EH-domain containing 4, isoform CRA_a OS	6	104.9	101.2	100.6	101.2	103.05	100.9	0.979136342	0.962696
A0A024R9J0	RAD21	RAD21 homolog (S. pombe), isoform CRA_a	16	101.1	100.1	97.8	99.2	100.6	98.5	0.979125249	0.962549
O00429	DNM1L	Dynamin-1-like protein OS=Homo sapiens G	22	97.9	98.4	97	95.2	98.15	96.1	0.979113602	0.962394
Q8N2K0	ABHD12	Monoacylglycerol lipase ABHD12 OS=Homo	6	101.8	104	103.1	98.4	102.9	100.75	0.979105928	0.962293
Q9NV66	TYW1	S-adenosyl-L-methionine-dependent tRNA 4-	2	99	97.2	92.2	99.9	98.1	96.05	0.979102956	0.962254
P0CG08	GPR89B	Golgi pH regulator B OS=Homo sapiens GN=	6	105.5	95.2	100.3	96.2	100.35	98.25	0.979073244	0.961859
A0A024R0L5	GSK3A	Glycogen synthase kinase 3 alpha, isoform C	6	99.3	101.2	96	100.3	100.25	98.15	0.979052369	0.961583
A0A024RB89	OBFC2B	Oligonucleotide/oligosaccharide-binding fold	2	103.8	101.4	97.7	103.2	102.6	100.45	0.979044834	0.961483
Q16706	MAN2A1	Alpha-mannosidase 2 OS=Homo sapiens GN=	15	100.9	99.5	99.1	97.1	100.2	98.1	0.979041916	0.961444
Q9Y3D2	MSRB2	Methionine-R-sulfoxide reductase B2, mitoch	1	100.5	94.8	96.6	94.6	97.65	95.6	0.979006656	0.960976
Q92945	KHSRP	Far upstream element-binding protein 2 OS=H	25	103	101.6	100.6	99.7	102.3	100.15	0.978983382	0.960668
A8K6K9		Hepatocyte growth factor OS=Homo sapiens	1	86.2	108.6	96.3	94.4	97.4	95.35	0.978952772	0.960262
Q96G03	PGM2	Phosphoglucomutase-2 OS=Homo sapiens G	7	98.6	100.8	98.4	96.8	99.7	97.6	0.97893681	0.96005
Q59HH7		X-ray repair cross complementing protein 1 v	2	103.7	100.4	96	103.8	102.05	99.9	0.978931896	0.959985
P56134	ATP5J2	ATP synthase subunit f, mitochondrial OS=H	1	111.3	92.4	101.4	98	101.85	99.7	0.978890525	0.959436
P46939	UTRN	Utrophin OS=Homo sapiens GN=UTRN PE=	40	104.6	99.1	99.6	99.8	101.85	99.7	0.978890525	0.959436
O00165	HAX1	HCLS1-associated protein X-1 OS=Homo sap	5	98.3	100.5	97.6	97	99.4	97.3	0.978873239	0.959207
Q15437	SEC23B	Protein transport protein Sec23B OS=Homo s	10	103	100.3	99.2	99.8	101.65	99.5	0.978848992	0.958885
Q8NBN7	RDH13	Retinol dehydrogenase 13 OS=Homo sapiens	7	101.1	102	100.2	98.6	101.55	99.4	0.978828163	0.958609
Q709C8	VPS13C	Vacuolar protein sorting-associated protein 13	11	102.3	96	97	97.1	99.15	97.05	0.97881997	0.9585
Q9GZS3	WDR61	WD repeat-containing protein 61 OS=Homo s	13	101.4	101.6	99.2	99.5	101.5	99.35	0.978817734	0.958471
Q14676	MDC1	Mediator of DNA damage checkpoint protein	18	102.8	100.1	96.4	102.2	101.45	99.3	0.978807294	0.958332
Q8TE02	ELP5	Elongator complex protein 5 OS=Homo sapie	1	100.6	102.3	85.7	112.9	101.45	99.3	0.978807294	0.958332
Q5VXV3	SET	SET OS=Homo sapiens GN=SET PE=2 SV=	7	102.7	100.2	98.4	100.2	101.45	99.3	0.978807294	0.958332
Q5SQH4	DBP2	DBP2 protein OS=Homo sapiens GN=DBP2	23	101.7	101.1	98.4	100.1	101.4	99.25	0.978796844	0.958194
O15287	FANCG	Fanconi anemia group G protein OS=Homo s	1	101.3	96.7	88.6	105.2	99	96.9	0.978787879	0.958075
A0A024R886	TRUB2	TruB pseudouridine (Psi) synthase homolog 2	3	100.2	102.5	102.2	96.2	101.35	99.2	0.978786384	0.958055
Q96HA1	POM121	Nuclear envelope pore membrane protein PO	6	103	99.5	98.6	99.6	101.25	99.1	0.978765432	0.957777
P52735	VAV2	Guanine nucleotide exchange factor VAV2 O	1	106.9	99.8	95.7	106.6	103.35	101.15	0.978713111	0.957083
Q05DF2	SF3A2	SF3A2 protein (Fragment) OS=Homo sapiens	7	104.3	97.6	99.8	97.8	100.95	98.8	0.978702328	0.95694
O43709	WBSCR22	Probable 18S rRNA (guanine-N(7))-methyltra	2	98.4	107.6	105.9	95.7	103	100.8	0.978640777	0.956124
Q9UJY4	GGA2	ADP-ribosylation factor-binding protein GGA	1	108.6	106.7	108.6	102.1	107.65	105.35	0.978634464	0.95604
O95235	KIF20A	Kinesin-like protein KIF20A OS=Homo sapie	7	100	101.2	99.6	97.3	100.6	98.45	0.978628231	0.955957
A0A024QZ70	TAOK1	TAO kinase 1, isoform CRA_a OS=Homo sap	5	99.6	101.5	101.7	95.1	100.55	98.4	0.978617603	0.955817
Q15118	PDK1	[Pyruvate dehydrogenase (acetyl-transferring)	3	103.7	97.4	94.2	102.6	100.55	98.4	0.978617603	0.955817
Q9NV56	MRGBP	MRG/MORF4L-binding protein OS=Homo s	1	106.8	98.9	95.4	105.9	102.85	100.65	0.978609626	0.955711
A0A087WV05		Uncharacterized protein OS=Homo sapiens P	5	99.4	101.5	99.3	97.3	100.45	98.3	0.978596317	0.955534
B5BUD2	RFC2	Replication factor C 2 isoform 1 (Fragment) (	10	99.6	101.2	96.9	99.6	100.4	98.25	0.978585657	0.955393
A0A1B0GW4	ING5	Inhibitor of growth protein 5 OS=Homo sapie	1	103.8	96.6	96.4	99.7	100.2	98.05	0.978542914	0.954826
P05423	POLR3D	DNA-directed RNA polymerase III subunit R	2	105.3	99.5	99.2	101.2	102.4	100.2	0.978515625	0.954464
Q08945	SSRP1	FACT complex subunit SSRP1 OS=Homo sap	25	101.4	103.2	100.2	100	102.3	100.1	0.978494624	0.954186
B4DR61	SEC61A1	Protein transport protein Sec61 subunit alpha	5	103.2	101.3	100.7	99.4	102.25	100.05	0.978484108	0.954046
A0A0A0MQR	MAP4K5	Mitogen-activated protein kinase kinase kinas	1	110.6	103.1	110	99.1	106.85	104.55	0.978474497	0.953919
A0A024RDM	MRP63	Mitochondrial ribosomal protein 63, isoform (	2	105.2	99.1	101.1	98.8	102.15	99.95	0.978463045	0.953767
A0A024RAD7	STEAP3	STEAP family member 3, isoform CRA_b OS	3	105.9	98.3	100.4	99.4	102.1	99.9	0.978452498	0.953627
A0A024R816	TTYH3	Protein tweety homolog OS=Homo sapiens G	1	109.2	76.3	76.4	105.1	92.75	90.75	0.978436658	0.953417
P04899	GNAI2	Guanine nucleotide-binding protein G(i) subu	7	101.7	102.2	101.4	98.1	101.95	99.75	0.978420795	0.953206
B7Z8E7		cDNA FLJ55400, highly similar to Protein OS	9	102.5	101.3	99.2	100.2	101.9	99.7	0.978410206	0.953066
P41252	IARS	Isoleucine--tRNA ligase, cytoplasmic OS=Ho	37	102.9	100.8	100.3	99	101.85	99.65	0.978399607	0.952926
P61289	PSME3	Proteasome activator complex subunit 3 OS=I	11	102.5	101.1	100.8	98.4	101.8	99.6	0.978388998	0.952785
Q5JR94	RPS8	40S ribosomal protein S8 OS=Homo sapiens (	9	102.7	100.7	98.3	100.7	101.7	99.5	0.978367748	0.952503
B4DWX3		Importin subunit alpha OS=Homo sapiens PE	5	102.6	100.2	100.9	97.5	101.4	99.2	0.978303748	0.951654
B2RD96		cDNA, FLJ96514 OS=Homo sapiens PE=2 S	5	103.4	99.4	101.1	97.3	101.4	99.2	0.978303748	0.951654
C9JJ19	MRPS34	28S ribosomal protein S34, mitochondrial OS	8	100.9	101.5	100.1	97.9	101.2	99	0.97826087	0.951085
P26447	S100A4	Protein S100-A4 OS=Homo sapiens GN=S10	2	96.7	91.9	90.4	94.1	94.3	92.25	0.97826087	0.951085
Q9NQT4	EXOSC5	Exosome complex component RRP46 OS=Ho	6	96	101.8	94.9	98.6	98.9	96.75	0.97826087	0.951085
A0A0A0MSC	ADCY3	Adenylate cyclase type 3 OS=Homo sapiens (	1	93.9	94.3	82.3	101.8	94.1	92.05	0.978214665	0.950473
Q9Y5B9	SUPT16H	FACT complex subunit SPT16 OS=Homo sap	42	100.4	101.5	98.5	99	100.95	98.75	0.978207033	0.950371
Q8N144	GJD3	Gap junction delta-3 protein OS=Homo sapie	1	118.6	101.6	118.2	97.2	110.1	107.7	0.978201635	0.950299
O15397	IPO8	Importin-8 OS=Homo sapiens GN=IPO8 PE=	5	102.2	99.6	95.4	102	100.9	98.7	0.978196234	0.950228
Q9UBQ7	GRHPR	Glyoxylate reductase/hydroxypyruvate reduct	6	100.3	101.5	97.7	99.7	100.9	98.7	0.978196234	0.950228
A0A024RCJ8	hCG_1821276	HCG1821276, isoform CRA_a OS=Homo sap	7	103.9	102.3	99.7	102	103.1	100.85	0.978176528	0.949967
B3KM74		cDNA FLJ10425 fis, clone NT2RP1000326, l	8	98.8	102.5	99.8	97.1	100.65	98.45	0.978142077	0.94951
H9NL19	IFITM3	IFITM3 OS=Homo sapiens GN=IFITM3 PE=	4	98.4	98.2	93.3	99	98.3	96.15	0.978128179	0.949325
Q9Y3C0	CCDC53	WASH complex subunit CCDC53 OS=Homo	3	99.1	97.2	93	99	98.15	96	0.978094753	0.948882
Q8NBP0	TTC13	Tetratricopeptide repeat protein 13 OS=Homo	3	99.6	96.2	97	94.5	97.9	95.75	0.978038815	0.94814
Q14683	SMC1A	Structural maintenance of chromosomes prote	39	102.9	101.9	100.6	99.7	102.4	100.15	0.978027344	0.947988
D6W625	CHAF1A	Chromatin assembly factor 1, subunit A (P150	8	99.3	100.4	97.7	97.6	99.85	97.65	0.97796695	0.947187
P53367	ARFIP1	Arfaptin-1 OS=Homo sapiens GN=ARFIP1 P	2	104.4	99.7	98.5	101.1	102.05	99.8	0.977951984	0.946989
Q9BX68	HINT2	Histidine triad nucleotide-binding protein 2, m	5	100.7	98.4	98.8	95.9	99.55	97.35	0.977900552	0.946306
A0A0C4DGQ	CAPNS1	Calpain small subunit 1 OS=Homo sapiens G	6	99.5	99.3	97.2	97.2	99.4	97.2	0.977867203	0.945864
P12004	PCNA	Proliferating cell nuclear antigen OS=Homo s	12	100.3	102.8	99.9	98.7	101.55	99.3	0.977843427	0.945548
A0A024R056	GNB1	Guanine nucleotide binding protein (G protein	6	101.3	101.7	98.6	99.9	101.5	99.25	0.977832512	0.945404
P08238	HSP90AB1	Heat shock protein HSP 90-beta OS=Homo s	30	96.7	101.2	96.1	97.4	98.95	96.75	0.977766549	0.944529
A0A024R075	UBE2J2	Ubiquitin-conjugating enzyme E2, J2 (UBC6	1	112	94.7	91.4	110.7	103.35	101.05	0.977745525	0.94425
Q96TA2	YME1L1	ATP-dependent zinc metalloprotease YME1L	17	101.9	100.2	98.5	99.1	101.05	98.8	0.977733795	0.944095
A0A024R125	PRKAG1	Protein kinase, AMP-activated, gamma 1 non-	4	104.2	97.5	97	100.2	100.85	98.6	0.977689638	0.943509
E5RG17	TATDN1	Putative deoxyribonuclease TATDN1 (Fragm	7	103.9	97.8	94.6	102.6	100.85	98.6	0.977689638	0.943509
Q8WVM8	SCFD1	Sec1 family domain-containing protein 1 OS=	12	103.6	102.5	100	101.5	103.05	100.75	0.977680738	0.943391
A0A0A6YZ17		Ubiquitin specific protease 13 OS=Homo sapi	2	92.7	108.8	91.7	105.3	100.75	98.5	0.977667494	0.943216
Q5T9B7	AK1	Adenylate kinase isoenzyme 1 OS=Homo sap	5	96.7	104.7	94	102.9	100.7	98.45	0.977656405	0.943068

Q8IYQ7	THNSL1	Threonine synthase-like 1 OS=Homo sapiens	4	104.7	96.5	99.2	97.5	100.6	98.35	0.977634195	0.942774
H3BQK9	MACF1	Microtubule-actin cross-linking factor 1, isofo	30	101.7	99.1	97.4	98.9	100.4	98.15	0.977589641	0.942183
P30622	CLIP1	CAP-Gly domain-containing linker protein 1 (	7	100.5	100.1	96.2	99.9	100.3	98.05	0.977567298	0.941887
A0A024R6Z0	DYNC1LI2	Dynein, cytoplasmic 1, light intermediate chai	6	105.8	99.1	98	102.3	102.45	100.15	0.977550024	0.941657
Q96RL7	VPS13A	Vacuolar protein sorting-associated protein 13	8	105.4	99.4	99.6	100.6	102.4	100.1	0.977539063	0.941512
A0A075B6F9	NOSIP	Nitric oxide synthase-interacting protein OS=	7	101	103.6	100.3	99.7	102.3	100	0.977517107	0.941221
Q9UBC2	EPS15L1	Epidermal growth factor receptor substrate 15	16	101.2	103.4	100.9	99.1	102.3	100	0.977517107	0.941221
B2R960		cDNA, FLJ94230, highly similar to Homo sap	11	100.3	99.8	96	99.6	100.05	97.8	0.977511244	0.941143
P62380	TBPL1	TATA box-binding protein-like protein 1 OS=	1	102.7	106.2	96.7	107.5	104.45	102.1	0.977501197	0.94101
Q5U4P2	ASPHD1	Aspartate beta-hydroxylase domain-containing	2	103.7	100.5	93.7	105.9	102.1	99.8	0.977473066	0.940637
P52747	ZNF143	Zinc finger protein 143 OS=Homo sapiens GN	1	98.5	101.2	98.3	96.9	99.85	97.6	0.977466199	0.940546
Q86U70	LDB1	LIM domain-binding protein 1 OS=Homo sap	1	109.1	90.3	97	97.9	99.7	97.45	0.977432297	0.940096
O43676	NDUFB3	NADH dehydrogenase [ubiquinone] 1 beta su	2	104.4	95	102.6	92.3	99.7	97.45	0.977432297	0.940096
B7Z6C9		Transmembrane 9 superfamily member OS=H	8	102.3	96.9	98.1	96.6	99.6	97.35	0.977409639	0.939796
A0A0A0MTC	RNF213	E3 ubiquitin-protein ligase RNF213 OS=Hom	4	108.6	95	96.9	102.1	101.8	99.5	0.97740668	0.939756
P10155	TROVE2	60 kDa SS-A/Ro ribonucleoprotein OS=Homo	18	101.6	101.7	99.1	99.6	101.65	99.35	0.97737334	0.939315
O95243	MBD4	Methyl-CpG-binding domain protein 4 OS=H	2	110.3	97.3	97.6	105.3	103.8	101.45	0.977360308	0.939141
O60341	KDM1A	Lysine-specific histone demethylase 1A OS=I	24	101	101.3	99.2	98.5	101.15	98.85	0.977261493	0.937831
Q14696	MESDC2	LDLR chaperone MESD OS=Homo sapiens (	7	103.1	103.4	101.4	100.4	103.25	100.9	0.977239709	0.937542
Q2KHR3	QSER1	Glutamine and serine-rich protein 1 OS=Hom	5	99.6	98.1	93.7	99.5	98.85	96.6	0.97723824	0.937523
A0A087X2H1	HECTD1	E3 ubiquitin-protein ligase HECTD1 OS=Hor	5	99.4	98.2	93.9	99.2	98.8	96.55	0.977226721	0.93737
A0A087WYL!	SEZ6L2	Seizure 6-like protein 2 OS=Homo sapiens Gl	9	102.5	99.4	96.4	100.9	100.95	98.65	0.977216444	0.937233
O00267	SUPT5H	Transcription elongation factor SPT5 OS=Hor	25	101.5	100.3	97.8	99.4	100.9	98.6	0.977205154	0.937084
Q5TF85	FAM46A	Family with sequence similarity 46, member A	1	118.4	91.8	102.8	102.6	105.1	102.7	0.977164605	0.936546
Q9C0B5	ZDHHC5	Palmitoyltransferase ZDHHC5 OS=Homo sap	3	105.1	100.4	97.9	102.9	102.75	100.4	0.977128954	0.936074
A8K0B9		rRNA adenine N(6)-methyltransferase OS=Ho	7	104.3	101.1	100.1	100.6	102.7	100.35	0.977117819	0.935926
A0A024QZH6	SR-A1	Serine arginine-rich pre-mRNA splicing facto	3	98.1	102.9	94.7	101.7	100.5	98.2	0.977114428	0.935881
Q9H967	WDR76	WD repeat-containing protein 76 OS=Homo s	7	102.4	98.5	98.1	98.2	100.45	98.15	0.977103036	0.93573
Q4LE43	PLCG1	Phosphoinositide phospholipase C (Fragment)	4	104	101	97.5	102.8	102.5	100.15	0.977073171	0.935334
Q8IVS2	MCAT	Malonyl-CoA-acyl carrier protein transacylase	9	104.4	100.4	101.1	99	102.4	100.05	0.977050781	0.935037
Q15020	SART3	Squamous cell carcinoma antigen recognized	19	101.6	102.5	99.8	99.6	102.05	99.7	0.976972073	0.933993
Q12904	AIMP1	Aminoacyl tRNA synthase complex-interactin	11	102.9	100.8	98.8	100.2	101.85	99.5	0.976926853	0.933394
J3KNQ4	PARVA	Alpha-parvin OS=Homo sapiens GN=PARVA	3	103.1	100.5	98.8	100.1	101.8	99.45	0.976915521	0.933243
Q58EY4	SMARCC1	SWI/SNF related, matrix associated, actin dep	8	103.9	98.9	97.7	100.4	101.4	99.05	0.976824458	0.932036
Q15645	TRIP13	Pachytene checkpoint protein 2 homolog OS=	3	99.7	94.4	92.9	96.7	97.05	94.8	0.976816074	0.931924
A0A024RAZ7	HNRPA1	Heterogeneous nuclear ribonucleoprotein A1,	15	97.5	100.6	94.3	99.2	99.05	96.75	0.976779404	0.931438
Q9Y375	NDUFAF1	Complex I intermediate-associated protein 30	6	100.6	101.2	97.6	99.5	100.9	98.55	0.976709613	0.930513
Q9H089	LSG1	Large subunit GTPase 1 homolog OS=Homo	14	104.7	100.7	99.7	100.9	102.7	100.3	0.976630964	0.929471
A8K8X0		cDNA FLJ75187, highly similar to Homo sap	4	99.6	101.4	93.6	102.7	100.5	98.15	0.976616915	0.929284
P62258	YWHAE	14-3-3 protein epsilon OS=Homo sapiens GN	17	100.9	100	97.7	98.5	100.45	98.1	0.976605276	0.92913
A0A024R5Z8	RAB11A	RAB11A, member RAS oncogene family, iso	1	99.7	105.2	94.5	105.6	102.45	100.05	0.976573939	0.928714
Q96Q45	TMEM237	Transmembrane protein 237 OS=Homo sapien	5	100.3	95.9	96.8	94.8	98.1	95.8	0.976554536	0.928457
A0A024R7E3	DNMT1	DNA (cytosine-5)-methyltransferase OS=Hon	37	100.4	100	97.8	97.9	100.2	97.85	0.976546906	0.928356
A0A140VK70		Testis secretory sperm-binding protein Li 197	26	100.8	103.7	100.7	99	102.25	99.85	0.976528117	0.928107
Q53FP3		NFS1 nitrogen fixation 1 isoform a variant (Fr	13	101.7	98.1	97.6	97.5	99.9	97.55	0.976476476	0.927422
A0A024R5X7	CLPX	ClpX caseinolytic peptidase X homolog (E. co	18	101.1	98.7	98	97.1	99.9	97.55	0.976476476	0.927422
B2RAH7		cDNA, FLJ94921, highly similar to Homo sap	14	98.4	101.3	96.7	98.3	99.85	97.5	0.976464697	0.927266
A8K669		cDNA FLJ78452, highly similar to Homo sap	4	91.1	99.8	90.6	95.8	95.45	93.2	0.976427449	0.926772
Q6PID6	TTC33	Tetratricopeptide repeat protein 33 OS=Homo	1	111.4	87.8	99.4	95.1	99.6	97.25	0.976405622	0.926483
Q9BT25	HAUS8	HAUS augmin-like complex subunit 8 OS=Ho	1	89.2	93	89.9	88	91.1	88.95	0.976399561	0.926402
B3KX14		cDNA FLJ44463 fis, clone UTERU2024969,	1	101.7	101.4	93.6	104.7	101.55	99.15	0.976366322	0.925962
H0YAV5	CHTF8	Chromosome transmission fidelity protein 8 h	1	100.7	102.3	103.2	95	101.5	99.1	0.97635468	0.925807
P14373	TRIM27	Zinc finger protein RFP OS=Homo sapiens G	3	103.4	99.2	93.4	104.4	101.3	98.9	0.976307996	0.925189
A0A024R688	MNAT1	Menage a trois homolog 1, cyclin H assembly	2	103.8	94.5	96.3	97.3	99.15	96.8	0.976298538	0.925063
A8KAC4		cDNA FLJ78754, highly similar to Homo sap	5	110.1	96.6	97.9	103.9	103.35	100.9	0.976294146	0.925005
A8K6H6		Tyrosine-protein phosphatase non-receptor typ	5	97	109.7	103.8	98	103.35	100.9	0.976294146	0.925005
P60891	PRPS1	Ribose-phosphate pyrophosphokinase 1 OS=F	4	101.4	101	98.4	99.2	101.2	98.8	0.976284585	0.924878
V9HW12	HEL-S-2a	Epididymis secretory sperm binding protein L	9	98.4	99.7	96.5	96.9	99.05	96.7	0.976274609	0.924746
A0A0S2PZM4	JMJD6	Bifunctional arginine demethylase and lysyl-h	4	104.2	98	96.3	101.1	101.1	98.7	0.976261128	0.924567
Q5U5J2	CSNK2A1	CSNK2A1 protein OS=Homo sapiens GN=CS	16	99.5	102.6	98.4	98.9	101.05	98.65	0.976249381	0.924412
Q8NFH4	NUP37	Nucleoporin Nup37 OS=Homo sapiens GN=N	11	97.4	100.3	96	97	98.85	96.5	0.976226606	0.92411
C0LQF2		Deoxyribonuclease II (Fragment) OS=Homo s	6	107.4	94.4	97.1	99.9	100.9	98.5	0.976214073	0.923943
Q9H5K3	POMK	Protein O-mannose kinase OS=Homo sapiens	2	106.3	99.7	101.3	99.8	103	100.55	0.976213592	0.923937
A8K8A4		cDNA FLJ77640, highly similar to Homo sap	3	97.6	99.9	95.6	97.2	98.75	96.4	0.976202532	0.923791
A0A024QYR8	TM9SF2	Transmembrane 9 superfamily member OS=H	11	105.2	99.8	102.7	97.4	102.5	100.05	0.976097561	0.9224
A0A0B4J2A0		Uncharacterized protein OS=Homo sapiens PI	1	99.2	88.3	87.4	95.6	93.75	91.5	0.976	0.921106
P30260	CDC27	Cell division cycle protein 27 homolog OS=H	13	101.3	98.3	96.8	98	99.8	97.4	0.975951904	0.920469
A0A024R571	EHD1	EH domain-containing protein 1 OS=Homo sa	7	102.1	101.5	97.7	101	101.8	99.35	0.975933202	0.920221
Q96C19	EFHD2	EF-hand domain-containing protein D2 OS=H	6	99.1	95.8	91.6	98.6	97.45	95.1	0.975885069	0.919583
A8K335		cDNA FLJ76254, highly similar to Homo sap	9	100	98.4	98.4	95.2	99.2	96.8	0.975806452	0.918541
Q0MQR4	PARG	Poly (ADP-ribose) glycohydrolase OS=Homo	1	103.2	94.7	92.4	100.7	98.95	96.55	0.975745326	0.917731
M0QYS1	RPL13A	60S ribosomal protein L13a (Fragment) OS=F	11	105	101.1	102.5	98.6	103.05	100.55	0.975739932	0.91766
P55786	NPEPPS	Puromycin-sensitive aminopeptidase OS=Hor	22	101.9	100	98.2	98.8	100.95	98.5	0.97573056	0.917535
O60826	CCDC22	Coiled-coil domain-containing protein 22 OS=	5	102.5	103.4	98.3	102.6	102.95	100.45	0.975716367	0.917347
B2RBP3		cDNA, FLJ95615, highly similar to Homo sap	10	101.6	100	93.4	103.3	100.8	98.35	0.975694444	0.917057
Q659C4	LARP1B	La-related protein 1B OS=Homo sapiens GN=	1	105.9	107.7	114.4	94	106.8	104.2	0.975655431	0.91654
Q53GF9		Full-length cDNA 5-PRIME end of clone CS(	8	104.6	100.7	101.3	99	102.65	100.15	0.975645397	0.916407
Q96AE7	TTC17	Tetratricopeptide repeat protein 17 OS=Homo	2	97.9	98.7	97	94.8	98.3	95.9	0.975584944	0.915606
E1NZA1	PRIC295	Peroxisome proliferator activated receptor inte	66	100.4	100.1	98.4	97.2	100.25	97.8	0.975561097	0.91529
B4E2I9		cDNA FLJ55486, highly similar to Semaphor	8	104.9	103.7	102.3	101.2	104.3	101.75	0.975551294	0.91516
A0A024R0Q6	ERCC1	Excision repair cross-complementing rodent r	2	99.1	97.2	89.4	102.1	98.15	95.75	0.975547631	0.915111
Q96Q42	ALS2	Alsin OS=Homo sapiens GN=ALS2 PE=1 SV	2	95.8	108.6	99.4	100	102.2	99.7	0.97553816	0.914986
Q13868	EXOSC2	Exosome complex component RRP4 OS=Hor	8	101.8	102.5	99	100.3	102.15	99.65	0.975526187	0.914828
Q9Y4X5	ARIH1	E3 ubiquitin-protein ligase ARIH1 OS=Homo	3	103.9	96.3	95	100.3	100.1	97.65	0.975524476	0.914805
Q7KZF4	SND1	Staphylococcal nuclease domain-containing p	45	102.8	101.1	100.6	98.3	101.95	99.45	0.975478176	0.914191
Q10570	CPSF1	Cleavage and polyadenylation specificity facto	27	103.8	99.8	100.9	97.7	101.8	99.3	0.975442043	0.913713
Q9NX62	IMPAD1	Inositol monophosphatase 3 OS=Homo sapien	9	100	99.5	96.7	97.9	99.75	97.3	0.975438596	0.913667
Q9UQ90	SPG7	Paraplegin OS=Homo sapiens GN=SPG7 PE=	5	103.9	95.6	96.4	98.2	99.75	97.3	0.975438596	0.913667
Q5T6F2	UBAP2	Ubiquitin-associated protein 2 OS=Homo sap	5	101.2	94.2	96.9	93.7	97.7	95.3	0.975435005	0.91362
Q2NL82	TSR1	Pre-rRNA-processing protein TSR1 homolog	16	100.6	102.9	99.3	99.2	101.75	99.25	0.975429975	0.913552
B4E2V3		cDNA FLJ56606, highly similar to Serine/thre	1	99.8	103.5	99	99.3	101.65	99.15	0.975405804	0.913233
E5KNL5		Mitochondrial endonuclease G OS=Homo sap	7	102.9	100.4	97.3	101	101.65	99.15	0.975405804	0.913233
A8K564		cDNA FLJ76716, highly similar to Homo sap	3	107.3	95.8	96.9	101.2	101.55	99.05	0.975381585	0.912912
A8K3W7		cDNA FLJ76271, highly similar to Homo sap	6	101.6	100.9	101.8	95.7	101.25	98.75	0.975308642	0.911946
A8K0C2		cDNA FLJ75950 OS=Homo sapiens PE=2 SV	3	103.2	98.9	94.7	102.4	101.05	98.55	0.975259772	0.911298
P23528	CFL1	Cofilin-1 OS=Homo sapiens GN=CFL1 PE=1	11	96.7	97.3	94.3	94.9	97	94.6	0.975257732	0.911271
H6QX63		Hepatocellular carcinoma related protein 2 OS	8	99.3	102.4	99.3	97.4	100.85	98.35	0.975210709	0.910648
F8W6G1	NRBP1	Nuclear receptor-binding protein OS=Homo s	2	101.8	103.9	103	97.6	102.85	100.3	0.975206612	0.910594
Q8WY22	BRI3BP	BRI3-binding protein OS=Homo sapiens GN=	2	103.6	97.9	94.3	102.2	100.75	98.25	0.975186104	0.910322
P56381	ATP5E	ATP synthase subunit epsilon, mitochondrial (	2	100	97.2	98	94.3	98.6	96.15	0.97515213	0.909872
B3KMS0		Condensin complex subunit 1 OS=Homo sapi	19	102	103	99.9	100	102.5	99.95	0.975121951	0.909473
Q86Y07	VRK2	Serine/threonine-protein kinase VRK2 OS=Ho	7	100	104.9	93.4	106.4	102.45	99.9	0.97510981	0.909312
Q96GD0	PDXP	Pyridoxal phosphate phosphatase OS=Homo s	4	103.5	97	95.3	100.2	100.25	97.75	0.975062344	0.908683
P25490	YY1	Transcriptional repressor protein YY1 OS=Ho	7	98.8	105.2	100.1	98.8	102	99.45	0.975	0.907858
Q86XK2	FBXO11	F-box only protein 11 OS=Homo sapiens GN=	1	97.6	106.2	100.4	98.3	101.9	99.35	0.974975466	0.907532
Q5T5H1	ENSA	Alpha-endosulfine OS=Homo sapiens GN=EN	2	97.7	102.1	91.9	102.9	99.9	97.4	0.974974975	0.907526
A0A087X0R6	SNX12	Sorting nexin-12 OS=Homo sapiens GN=SNX	4	102.4	101.2	104.1	94.4	101.8	99.25	0.974950884	0.907207
Q9NX40	OCIAD1	OCIA domain-containing protein 1 OS=Homo	13	103.3	100.2	98.8	99.6	101.75	99.2	0.974938575	0.907044
Q5VIR6	VPS53	Vacuolar protein sorting-associated protein 53	3	99.5	99.7	95.9	98.3	99.6	97.1	0.974899598	0.906528
A0A024R370	TMF1	TATA element modulatory factor 1, isoform (	19	105.7	97.3	97.5	100.4	101.5	98.95	0.974876847	0.906226
P43686	PSMC4	26S protease regulatory subunit 6B OS=Homo	17	101	101.9	98.4	99.4	101.45	98.9	0.974864465	0.906063
Q9UNL4	ING4	Inhibitor of growth protein 4 OS=Homo sapie	1	101	101.7	97.7	99.9	101.35	98.8	0.974839665	0.905734
Q7Z7H8	MRPL10	39S ribosomal protein L10, mitochondrial OS	3	102.7	100	95.3	102.3	101.35	98.8	0.974839665	0.905734
B2RE59		cDNA, FLJ93148, highly similar to Homo sap	5	102.4	104.2	101.2	100.2	103.3	100.7	0.974830591	0.905614
Q99755	PIP5K1A	Phosphatidylinositol 4-phosphate 5-kinase typ	8	104.5	101.6	101.8	99.1	103.05	100.45	0.974769529	0.904805
A0A0S2Z489	PSMD12	Proteasome (Prosome, macropain) 26S subuni	20	103.4	102.4	100.4	100.2	102.9	100.3	0.97473275	0.904318
A0A024R845	RAB14	RAB14, member RAS oncogene family, isofo	13	106.2	99.6	102	98.6	102.9	100.3	0.97473275	0.904318
D3DSW1	hCG_1640171	HCG1640171, isoform CRA_a OS=Homo sap	1	102.7	83.2	92.7	88.5	92.95	90.6	0.97471759	0.904118
Q9UNE7	STUB1	E3 ubiquitin-protein ligase CHIP OS=Homo s	10	101.5	99.9	99.8	96.5	100.7	98.15	0.974677259	0.903584
P50454	SERPINH1	Serpin H1 OS=Homo sapiens GN=SERPINH	27	99.4	101.9	96.7	99.5	100.65	98.1	0.97466468	0.903417
O75298	RTN2	Reticulon-2 OS=Homo sapiens GN=RTN2 PI	3	97	96.2	93.3	95	96.6	94.15	0.974637681	0.90306
Q6YN16	HSDL2	Hydroxysteroid dehydrogenase-like protein 2	15	100.4	100.6	99.6	96.3	100.5	97.95	0.974626866	0.902916

A8K3U6		cDNA FLJ76133, highly similar to Homo sap	2	103.2	101.6	93.8	105.8	102.4	99.8	0.974609375	0.902685
B3KM68		cDNA FLJ10398 fis, clone NT2RM4000349,	9	102.3	98.4	97.4	98.2	100.35	97.8	0.974588939	0.902414
Q9UK59	DBR1	Lariat debranching enzyme OS=Homo sapien	5	91.1	89.9	87	89.4	90.5	88.2	0.974585635	0.902371
Q96G25	MED8	Mediator of RNA polymerase II transcription	4	95.7	100.9	94.7	96.9	98.3	95.8	0.97456765	0.902133
A8K818		cDNA FLJ75784, highly similar to Homo sap	10	105.3	98.8	96.9	102	102.05	99.45	0.974522293	0.901532
P46108	CRK	Adapter molecule crk OS=Homo sapiens GN=	6	102.9	101.2	91.7	107.2	102.05	99.45	0.974522293	0.901532
A8KAQ3		cDNA FLJ76489, highly similar to Homo sap	1	101.4	98.6	92.8	102.1	100	97.45	0.9745	0.901237
O43609	SPRY1	Protein sprouty homolog 1 OS=Homo sapiens	5	102.7	101	98.9	99.6	101.85	99.25	0.974472263	0.90087
Q5VWN6	FAM208B	Protein FAM208B OS=Homo sapiens GN=FA	8	98.4	101.3	96.4	98.2	99.85	97.3	0.974461693	0.90073
B0QY95	MIEF1	Mitochondrial dynamics protein MID51 OS=I	3	100.1	103.3	99.9	98.3	101.7	99.1	0.974434612	0.900372
O95983	MBD3	Methyl-CpG-binding domain protein 3 OS=H	8	101.5	101.5	97.6	100.2	101.5	98.9	0.974384236	0.899705
Q3B726	TWISTNB	DNA-directed RNA polymerase I subunit RPA	6	99	92.1	91.3	94.9	95.55	93.1	0.974358974	0.899371
A7UJ17		DnaJ OS=Homo sapiens PE=2 SV=1	1	103.4	99.3	99.4	98.1	101.35	98.75	0.974346325	0.899203
P29992	GNA11	Guanine nucleotide-binding protein subunit al	4	110.5	99.9	104.5	100.5	105.2	102.5	0.974334601	0.899048
Q5U0F4	EIF3S2	Eukaryotic translation initiation factor 3 subun	17	102.5	103.7	102.9	98	103.1	100.45	0.974296799	0.898548
P07954	FH	Fumarate hydratase, mitochondrial OS=Homo	17	101.7	100.4	98.2	98.7	101.05	98.45	0.974270163	0.898195
Q7Z7N9	TMEM179B	Transmembrane protein 179B OS=Homo sapi	1	95.9	98.4	97.3	92	97.15	94.65	0.974266598	0.898148
Q96T58	SPEN	Msx2-interacting protein OS=Homo sapiens C	8	105.1	96.9	97.9	98.9	101	98.4	0.974257426	0.898026
A0A0B4J1W0	MED24	Mediator of RNA polymerase II transcription	13	103.1	98.8	97.1	99.6	100.95	98.35	0.974244676	0.897858
P48509	CD151	CD151 antigen OS=Homo sapiens GN=CD15	5	105.3	100.3	100	100.3	102.8	100.15	0.97422179	0.897555
A0A024R5S9	NEDD4	Neural cell expressed, developmentally down-	2	104.1	97.6	97.3	99.2	100.85	98.25	0.974219137	0.89752
P53609	PGGT1B	Geranylgeranyl transferase type-1 subunit bet	2	100.4	101.2	93.7	102.7	100.8	98.2	0.974206349	0.89735
A0A024QZR3	hCG_2002731	Protein pelota homolog OS=Homo sapiens GI	5	99.7	101.9	99.8	96.6	100.8	98.2	0.974206349	0.89735
O60888	CUTA	Protein CutA OS=Homo sapiens GN=CUTA	3	105.6	95.9	97.2	99.1	100.75	98.15	0.974193548	0.897182
Q86SF2	GALNT7	N-acetylgalactosaminyltransferase 7 OS=Hon	13	100.8	104.4	98.6	101.3	102.6	99.95	0.97417154	0.89689
A4D213	DKFZP586J06	DKFZP586J0619 protein OS=Homo sapiens C	11	104.2	100.9	99.7	100.1	102.55	99.9	0.974158947	0.896723
Q9BVG3	TRIM62	E3 ubiquitin-protein ligase TRIM62 OS=Hom	1	102.3	98.7	100.7	95.1	100.5	97.9	0.974129353	0.896332
P41743	PRKCI	Protein kinase C iota type OS=Homo sapiens	8	102.9	101.8	98.4	101	102.35	99.7	0.974108451	0.896055
P30154	PPP2R1B	Serine/threonine-protein phosphatase 2A 65 k	7	101.1	99.7	98.8	96.8	100.4	97.8	0.974103586	0.895991
Q9NPI1	BRD7	Bromodomain-containing protein 7 OS=Homo	6	105.6	99	96.4	102.9	102.3	99.65	0.974095797	0.895888
A0A024R098	AMPD2	Adenosine monophosphate deaminase 2 (Isofo	8	100.6	104	101.2	98.1	102.3	99.65	0.974095797	0.895888
Q13595	TRA2A	Transformer-2 protein homolog alpha OS=Ho	4	101.2	103.1	99.4	99.6	102.15	99.5	0.974057758	0.895384
A4LAA3	ATRX	Alpha thalassemia/mental retardation syndron	9	104	99.9	97.4	101.2	101.95	99.3	0.974006866	0.894712
P62913	RPL11	60S ribosomal protein L11 OS=Homo sapiens	10	101.4	102.5	100	98.6	101.95	99.3	0.974006866	0.894712
Q96BR5	COA7	Cytochrome c oxidase assembly factor 7 OS=	8	100	103.4	101.8	96.3	101.7	99.05	0.97394297	0.893866
Q9NYM9	BET1L	BET1-like protein OS=Homo sapiens GN=BE	4	99.5	107.2	101.1	100.2	103.35	100.65	0.973875181	0.892969
B9EG70	NPAT	Nuclear protein, ataxia-telangiectasia locus O	1	92.9	105.9	93.2	100.4	99.4	96.8	0.973843058	0.892544
Q52LJ0	FAM98B	Protein FAM98B OS=Homo sapiens GN=FA	8	106.1	100.3	101.7	99.3	103.2	100.5	0.973837209	0.892467
Q9H446	RWDD1	RWD domain-containing protein 1 OS=Homo	1	98.9	103.4	100	97	101.15	98.5	0.973801285	0.891991
Q6P1J9	CDC73	Parafibromin OS=Homo sapiens GN=CDC73	22	101.7	100.5	97.9	99	101.1	98.45	0.973788328	0.89182
A8K4K9		cDNA FLJ76169 OS=Homo sapiens PE=2 SV	9	102.4	99.8	99.1	97.8	101.1	98.45	0.973788328	0.89182
P19387	POLR2C	DNA-directed RNA polymerase II subunit RF	7	108.9	96.8	98.2	102.1	102.85	100.15	0.973748177	0.891289
Q15528	MED22	Mediator of RNA polymerase II transcription	2	103.1	98.7	95.6	100.9	100.9	98.25	0.973736373	0.891133
Q8N4V1	MMGT1	Membrane magnesium transporter 1 OS=Hom	2	106.8	98.5	96.2	103.7	102.65	99.95	0.973697029	0.890613
Q7L5D6	GET4	Golgi to ER traffic protein 4 homolog OS=Ho	5	102	95	93	98.8	98.5	95.9	0.973604061	0.889384
B3KTQ4		Hexosyltransferase OS=Homo sapiens PE=2 S	1	101.1	99.6	99.3	96.1	100.35	97.7	0.973592427	0.889229
O00159	MYO1C	Unconventional myosin-Ic OS=Homo sapiens	7	102.9	101.3	97.5	101.3	102.1	99.4	0.973555338	0.888739
L7RRS0	PIK3C2A	Phosphatidylinositol-4-phosphate 3-kinase, ca	14	102.2	98.1	96.6	98.4	100.15	97.5	0.97353969	0.888533
B3KRW4		cDNA FLJ34980 fis, clone OCBBF2000522,	6	102	98.3	94	101	100.15	97.5	0.97353969	0.888533
Q8WXQ4	ATP5S	ATP synthase coupling factor B-like 1 OS=H	1	105.2	95.1	99.3	95.7	100.15	97.5	0.97353969	0.888533
O95168	NDUFB4	NADH dehydrogenase [ubiquinone] 1 beta su	4	106.5	97.2	98.1	100.2	101.85	99.15	0.973490427	0.887881
Q8IUH5	ZDHHC17	Palmitoyltransferase ZDHHC17 OS=Homo sa	3	106	101.4	94.4	107.5	103.7	100.95	0.973481196	0.887759
Q6P5V6	SNX5	SNX5 protein (Fragment) OS=Homo sapiens	7	100.8	102.4	98.6	99.2	101.6	98.9	0.973425197	0.887019
B2R6H7		cDNA, FLJ92955, highly similar to Homo sap	16	101.8	101.3	99	98.7	101.55	98.85	0.973412112	0.886846
H7BXP1	NKIRAS2	NF-kappa-B inhibitor-interacting Ras-like pro	1	100.1	106.7	95.5	105.8	103.4	100.65	0.973404255	0.886742
Q9NYV4	CDK12	Cyclin-dependent kinase 12 OS=Homo sapien	4	100.5	102.2	98.3	99	101.35	98.65	0.973359645	0.886152
P62273	RPS29	40S ribosomal protein S29 OS=Homo sapiens	4	102.4	104	100.5	100.4	103.2	100.45	0.973352713	0.886061
Q9NTI5	PDS5B	Sister chromatid cohesion protein PDS5 homo	17	100.2	102.3	99.4	97.7	101.25	98.55	0.973333333	0.885805
Q71RC2	LARP4	La-related protein 4 OS=Homo sapiens GN=I	7	108	98.2	100.2	100.5	103.1	100.35	0.973326867	0.885719
Q13136	PPFIA1	Liprin-alpha-1 OS=Homo sapiens GN=PPFIA	7	99.5	102.6	96.4	100.3	101.05	98.35	0.973280554	0.885107
A2A274	ACO2	Aconitate hydratase, mitochondrial OS=Homo	8	104.7	97.4	99.2	97.5	101.05	98.35	0.973280554	0.885107
Q9P0M9	MRPL27	39S ribosomal protein L27, mitochondrial OS	5	99.8	102.1	100	96.5	100.95	98.25	0.973254086	0.884757
O43719	HTATSF1	HIV Tat-specific factor 1 OS=Homo sapiens C	11	105.3	100.1	100.5	99.4	102.7	99.95	0.97322298	0.884346
A0A024R2A7	LMAN1	Lectin, mannose-binding, 1, isoform CRA_b C	12	103.9	101.4	101	98.8	102.65	99.9	0.973209937	0.884173
F8WCF6	ARPC4-TTLL3	Actin-related protein 2/3 complex subunit 4 C	7	103	102.1	99.9	99.7	102.55	99.8	0.973183813	0.883828
O14929	HAT1	Histone acetyltransferase type B catalytic sub	8	99.8	101.3	100.4	95.3	100.55	97.85	0.973147688	0.883351
O75376	NCOR1	Nuclear receptor corepressor 1 OS=Homo sap	8	102.9	101.9	94.5	104.8	102.4	99.65	0.973144531	0.883309
Q8WUP2	FBLIM1	Filamin-binding LIM protein 1 OS=Homo sap	2	100.8	100.1	95.5	100	100.45	97.75	0.973120956	0.882997
Q8N4Q0	ZADH2	Prostaglandin reductase 3 OS=Homo sapiens	2	105.1	95.6	97.1	98.2	100.35	97.65	0.97309417	0.882644
O43504	LAMTOR5	Regulator complex protein LAMTOR5 OS=H	5	112	96	102.1	100.3	104	101.2	0.973076923	0.882416
Q8N5M1	ATPAF2	ATP synthase mitochondrial F1 complex asse	7	103.7	96.8	96	99.1	100.25	97.55	0.973067332	0.882289
Q4G0J3	LARP7	La-related protein 7 OS=Homo sapiens GN=I	5	101.3	105.5	100.8	100.4	103.4	100.6	0.972920696	0.880352
O94874	UFL1	E3 UFM1-protein ligase 1 OS=Homo sapiens	22	102.8	100.3	97.7	99.9	101.55	98.8	0.972919744	0.880339
J3QR44	CDK11B	Cyclin-dependent kinase 11B OS=Homo sapi	13	104.7	98.4	98.2	99.4	101.55	98.8	0.972919744	0.880339
A0A024RDE5	G3BP2	Ras-GTPase activating protein SH3 domain-b	14	102.6	104.1	102.5	98.6	103.35	100.55	0.972907596	0.880179
A0A140VKC9		Testis tissue sperm-binding protein Li 46a OS	14	100.7	102.2	98.4	99	101.45	98.7	0.972893051	0.879987
Q5T3F8	TMEM63B	CSC1-like protein 2 OS=Homo sapiens GN=T	3	102.9	99.5	95.4	101.5	101.2	98.45	0.972826087	0.879102
P24390	KDELR1	ER lumen protein-retaining receptor 1 OS=Ho	1	113.5	88.7	103.9	92.8	101.1	98.35	0.972799209	0.878747
E9PAU2	RAVER1	Ribonucleoprotein PTB-binding 1 OS=Homo	11	104.8	100.7	100.2	99.7	102.75	99.95	0.972749392	0.878089
Q15334	LLGL1	Lethal(2) giant larvae protein homolog 1 OS=	4	97.5	100.6	96.3	96.4	99.05	96.35	0.97274104	0.877979
A0A087WTW	UHRF1	E3 ubiquitin-protein ligase UHRF1 OS=Hom	13	100.6	97.5	94.9	97.8	99.05	96.35	0.97274104	0.877979
Q9BY44	EIF2A	Eukaryotic translation initiation factor 2A OS	20	102.9	102.5	98.7	101.1	102.7	99.9	0.972736125	0.877914
Q13200	PSMD2	26S proteasome non-ATPase regulatory subun	35	101.9	103.2	100.4	99.1	102.55	99.75	0.972696246	0.877387
Q86TB9	PATL1	Protein PAT1 homolog 1 OS=Homo sapiens C	4	101.9	92.2	94.7	94.1	97.05	94.4	0.972694487	0.877364
Q9P2W9	STX18	Syntaxin-18 OS=Homo sapiens GN=STX18 I	9	101.3	99.9	96.4	99.3	100.6	97.85	0.972664016	0.876961
B3KN49		cDNA FLJ13562 fis, clone PLACE1008080, I	6	98.7	95.1	93.7	94.8	96.9	94.25	0.972652219	0.876805
Q9GZS1	POLR1E	DNA-directed RNA polymerase I subunit RPA	9	103.2	94.2	97.8	94.2	98.7	96	0.972644377	0.876702
B4DPD5		Ubiquitin thioesterase OS=Homo sapiens PE=	10	99.3	101.6	97.3	98.1	100.45	97.7	0.972623196	0.876422
A0A087WZT2	METTL7B	Methyltransferase-like protein 7B OS=Homo	2	102.2	102.2	102.7	96.1	102.2	99.4	0.97260274	0.876152
P13639	EEF2	Elongation factor 2 OS=Homo sapiens GN=E	49	97.8	102.6	97.2	97.7	100.2	97.45	0.97255489	0.87552
A0A0S2Z500	OPTN	Optineurin isoform 1 (Fragment) OS=Homo s	3	105.2	95.1	94.9	99.9	100.15	97.4	0.972541188	0.87534
P80217	IFI35	Interferon-induced 35 kDa protein OS=Homo	7	108.1	99.3	98.8	102.9	103.7	100.85	0.972516876	0.875019
Q49AE6	PAIP2	PAIP2 protein OS=Homo sapiens GN=PAIP2	1	97.9	102.1	87	107.5	100	97.25	0.9725	0.874796
A0A0A0MRR	SNRPC	U1 small nuclear ribonucleoprotein C OS=Ho	4	102.4	101.2	102.8	95.2	101.8	99	0.972495088	0.874731
Q9Y3D8	AK6	Adenylate kinase isoenzyme 6 OS=Homo sap	3	103.2	100.1	97.7	100	101.65	98.85	0.972454501	0.874195
A6NFM0	POLR2J2	DNA-directed RNA polymerase II subunit RF	3	97.4	105.8	96.8	100.8	101.6	98.8	0.972440945	0.874016
Q9NWQ9	C14orf119	Uncharacterized protein C14orf119 OS=Hom	1	107.1	99.7	100.1	101	103.4	100.55	0.972437137	0.873966
Q99640	PKMYT1	Membrane-associated tyrosine- and threonine	3	98.5	104.6	99.6	97.9	101.55	98.75	0.972427376	0.873837
Q96K76	USP47	Ubiquitin carboxyl-terminal hydrolase 47 OS	7	107.6	102.7	104.5	100	105.15	102.25	0.972420352	0.873744
A0A024QZY5	PRPF4B	PRP4 pre-mRNA processing factor 4 homolo	21	99.3	103.7	100.3	97.1	101.5	98.7	0.972413793	0.873658
Q6FI81	CIAPIN1	Anamorsin OS=Homo sapiens GN=CIAPIN1	5	101.5	101.1	97	100	101.3	98.5	0.972359329	0.872939
Q9Y4A5	TRRAP	Transformation/transcription domain-associat	41	103.1	99.4	98	98.9	101.25	98.45	0.972345679	0.872758
P30876	POLR2B	DNA-directed RNA polymerase II subunit RF	30	102	100.5	99.6	97.3	101.25	98.45	0.972345679	0.872758
Q10567	AP1B1	AP-1 complex subunit beta-1 OS=Homo sapi	5	106.5	103.2	102.1	101.8	104.85	101.95	0.97234144	0.872702
Q9Y6M5	SLC30A1	Zinc transporter 1 OS=Homo sapiens GN=SL	4	102.8	103.1	101.2	99	102.95	100.1	0.972316659	0.872376
B2R5R5		cDNA, FLJ92583, highly similar to Homo sap	8	99.5	102.6	98.6	97.9	101.05	98.25	0.972290945	0.872036
Q15172	PPP2R5A	Serine/threonine-protein phosphatase 2A 56 k	3	101.4	100.7	99.4	97.1	101.05	98.25	0.972290945	0.872036
P55039	DRG2	Developmentally-regulated GTP-binding prot	2	103.8	98.2	92	104.4	101	98.2	0.972277228	0.871855
A0A0A0MTS	TTN	Titin OS=Homo sapiens GN=TTN PE=1 SV=	2	108.1	100.9	103.2	100	104.5	101.6	0.972248804	0.87148
Q7Z4X0		MO25-like protein OS=Homo sapiens PE=2 S	4	106.4	98.6	99	100.3	102.5	99.65	0.972195122	0.870772
O15269	SPTLC1	Serine palmitoyltransferase 1 OS=Homo sapi	11	105.2	99.8	99	100.3	102.5	99.65	0.972195122	0.870772
Q6PJT7	ZC3H14	Zinc finger CCCH domain-containing protein	15	103.4	101.6	101.1	98.2	102.5	99.65	0.972195122	0.870772
A0A0A0MT8	IVD	Isovaleryl-CoA dehydrogenase, mitochondria	11	100.1	101.3	96.2	99.6	100.7	97.9	0.972194638	0.870765
P78356	PIP4K2B	Phosphatidylinositol 5-phosphate 4-kinase typ	3	98.7	102.7	97.4	98.4	100.7	97.9	0.972194638	0.870765
A0A0U1RQM	MTHFS	5-formyltetrahydrofolate cyclo-ligase OS=Ho	3	101.7	99.6	96	99.7	100.65	97.85	0.972180825	0.870583
P62495	ETF1	Eukaryotic peptide chain release factor subuni	15	103.4	104.7	103.7	98.6	104.05	101.15	0.972128784	0.869897
B2RBM8		cDNA, FLJ95596, highly similar to Homo sap	17	101.1	99.7	98.4	96.8	100.4	97.6	0.972111554	0.86967
P54136	RARS	Arginine--tRNA ligase, cytoplasmic OS=Hom	32	102	102.3	100.9	97.7	102.15	99.3	0.972099853	0.869515
V9HW37	HEL-S-69	Epididymis secretory protein Li 69 OS=Homo	37	102.9	104.8	102.1	99.8	103.85	100.95	0.972075108	0.869188
Q9Y6A9	SPCS1	Signal peptidase complex subunit 1 OS=Hom	2	102.5	101.6	99.6	98.8	102.05	99.2	0.972072513	0.869155
P22570	FDXR	NADPH:adrenodoxin oxidoreductase, mitoch	13	97.2	99.6	94.4	96.9	98.4	95.65	0.972052846	0.868895
P62979	RPS27A	Ubiquitin-40S ribosomal protein S27a OS=Ho	3	103.1	100.7	99.2	98.9	101.9	99.05	0.972031403	0.868612

B3KUY2	PTGES3	Prostaglandin E synthase 3 (Cytosolic), isofor	6	99.5	104.3	98	100.1	101.9	99.05	0.972031403	0.868612
Q9NYK5	MRPL39	39S ribosomal protein L39, mitochondrial OS	15	104.8	98.9	100.1	97.9	101.85	99	0.972017673	0.86843
Q9NVA1	UQCC1	Ubiquinol-cytochrome-c reductase complex a	2	104.2	95.9	98.8	95.7	100.05	97.25	0.972013993	0.868382
Q9BZK7	TBL1XR1	F-box-like/WD repeat-containing protein TBI	13	100.9	102.6	99.2	98.6	101.75	98.9	0.971990172	0.868068
Q96KA5	CLPTM1L	Cleft lip and palate transmembrane protein 1-l	5	107.4	99.6	101.9	99.3	103.5	100.6	0.971980676	0.867943
Q13643	FHL3	Four and a half LIM domains protein 3 OS=H	3	106	100.4	101.2	99.4	103.2	100.3	0.971899225	0.866868
A0A0D9SF54	SPTAN1	Spectrin alpha chain, non-erythrocytic 1 OS=l	1	108.4	97.9	102.6	97.9	103.15	100.25	0.971885603	0.866689
Q9NNW5	WDR6	WD repeat-containing protein 6 OS=Homo sa	7	102.4	100	95.3	101.4	101.2	98.35	0.971837945	0.86606
A0A024RDR0	HMGB1	High-mobility group box 1, isoform CRA_a C	6	98.3	103.8	98.8	97.6	101.05	98.2	0.971796141	0.865509
A0A024R1X8	JUP	Junction plakoglobin, isoform CRA_a OS=Ho	12	104.5	104.6	104.2	99	104.55	101.6	0.971783835	0.865347
Q9NP79	VTA1	Vacuolar protein sorting-associated protein V	10	101.3	100.4	97.4	98.6	100.85	98	0.971740208	0.864771
O43765	SGTA	Small glutamine-rich tetratricopeptide repeat-	7	98.8	102.6	98.1	97.6	100.7	97.85	0.971698113	0.864216
A4D0P7	ORC5L	Origin recognition complex, subunit 5-like (Y	11	98.8	102.6	96.3	99.4	100.7	97.85	0.971698113	0.864216
Q5JSH3	WDR44	WD repeat-containing protein 44 OS=Homo s	2	98.6	106.3	94.7	104.4	102.45	99.55	0.971693509	0.864156
G8JLB6	HNRNPH1	Heterogeneous nuclear ribonucleoprotein H C	11	101.5	99.8	97.2	98.4	100.65	97.8	0.971684054	0.864031
Q5T0F9	CC2D1B	Coiled-coil and C2 domain-containing protein	3	101.5	99.8	97	98.6	100.65	97.8	0.971684054	0.864031
Q9HCU5	PREB	Prolactin regulatory element-binding protein C	9	102	99.1	97.7	97.7	100.55	97.7	0.971655893	0.86366
Q9UBS4	DNAJB11	DnaJ homolog subfamily B member 11 OS=H	10	103.6	101	100.3	98.5	102.3	99.4	0.971652004	0.863609
P62310	LSM3	U6 snRNA-associated Sm-like protein LSm3	2	87.6	95.7	81	97.1	91.65	89.05	0.971631206	0.863335
B2RC06		cDNA, FLJ95791, highly similar to Homo sap	4	108.8	95.6	98.9	99.7	102.2	99.3	0.971624266	0.863243
Q99460	PSMD1	26S proteasome non-ATPase regulatory subu	31	102.8	101.3	99.3	99	102.05	99.15	0.971582558	0.862693
Q15067	ACOX1	Peroxisomal acyl-coenzyme A oxidase 1 OS=	21	100.8	103.2	99.2	99	102	99.1	0.971568627	0.86251
P13929	ENO3	Beta-enolase OS=Homo sapiens GN=ENO3 F	5	98.7	94.2	89.4	98	96.45	93.7	0.971487818	0.861445
P16403	HIST1H1C	Histone H1.2 OS=Homo sapiens GN=HIST1I	4	93.3	106.5	96	98.1	99.9	97.05	0.971471471	0.861229
O60885	BRD4	Bromodomain-containing protein 4 OS=Homo	6	104.3	98.8	96.4	100.9	101.55	98.65	0.971442639	0.860849
B7Z7F0		cDNA FLJ56420, highly similar to Aspartyl a	8	102.3	100.6	101.5	95.6	101.45	98.55	0.97141449	0.860479
Q92685	ALG3	Dol-P-Man:Man(5)GlcNAc(2)-PP-Dol alpha-	2	107	92.3	96.1	97.5	99.65	96.8	0.9713999	0.860286
A0A0U1RRH	PHF14	PHD finger protein 14 OS=Homo sapiens GN	7	96	96.2	92	94.7	96.1	93.35	0.971383975	0.860076
A8K6K7		Glycogen [starch] synthase OS=Homo sapien	8	102	100.5	98	98.7	101.25	98.35	0.971358025	0.859734
Q8NFV4	ABHD11	Protein ABHD11 OS=Homo sapiens GN=AB	6	99.3	99.7	96.6	96.7	99.5	96.65	0.971356784	0.859718
A8K8K1		cDNA FLJ76936, highly similar to Homo sap	7	101.7	100.7	97.6	99	101.2	98.3	0.971343874	0.859548
Q8NFF5	FLAD1	FAD synthase OS=Homo sapiens GN=FLAD	11	99.9	99	95.4	97.8	99.45	96.6	0.971342383	0.859528
Q9Y266	NUDC	Nuclear migration protein nudC OS=Homo sa	14	102.4	103.1	100.3	99.3	102.75	99.8	0.971289538	0.858832
Q7L3S4	ZNF771	Zinc finger protein 771 OS=Homo sapiens GN	2	99	96	89.3	100.1	97.5	94.7	0.971282051	0.858734
Q9HC36	MRM3	rRNA methyltransferase 3, mitochondrial OS	5	103.9	101.4	102.8	96.6	102.65	99.7	0.971261568	0.858464
Q5T1V6	DDX59	Probable ATP-dependent RNA helicase DDX	1	105.9	95.9	95.4	100.6	100.9	98	0.971258672	0.858426
B5ME97	SEPT10	Septin 10, isoform CRA_c OS=Homo sapiens	8	105	100.2	99.6	99.7	102.6	99.65	0.971247563	0.858279
P42695	NCAPD3	Condensin-2 complex subunit D3 OS=Homo	4	105.2	100	97.1	102.2	102.6	99.65	0.971247563	0.858279
A0A024R8R4	NPLOC4	Nuclear protein localization 4 homolog (S. cer	18	101	100.7	98.2	97.7	100.85	97.95	0.971244422	0.858237
B3KM88		cDNA FLJ10527 fis, clone NT2RP2000932, l	2	103.3	101.8	102	97.2	102.55	99.6	0.971233545	0.858095
Q8TEM1	NUP210	Nuclear pore membrane glycoprotein 210 OS	31	101.1	100.3	99.2	96.4	100.7	97.8	0.971201589	0.857674
Q15907	RAB11B	Ras-related protein Rab-11B OS=Homo sapie	2	102.1	99.2	99.7	95.8	100.65	97.75	0.971187283	0.857485
Q9NRX2	MRPL17	39S ribosomal protein L17, mitochondrial OS	5	101.2	99.9	98	97.3	100.55	97.65	0.971158628	0.857108
P56937	HSD17B7	3-keto-steroid reductase OS=Homo sapiens G	3	102.2	98.9	98.2	97.1	100.55	97.65	0.971158628	0.857108
Q06203	PPAT	Amidophosphoribosyltransferase OS=Homo s	2	98.7	105.6	97.7	100.7	102.15	99.2	0.971120901	0.856611
Q7Z3R8	DKFZp686D1	DNA-directed RNA polymerase subunit beta	8	100.4	100.1	97.4	97.3	100.25	97.35	0.971072319	0.855971
H0YJ75	PPP2R5C	Serine/threonine-protein phosphatase 2A 56 k	3	98.4	101.9	99.3	95.2	100.15	97.25	0.971043435	0.855591
Q9Y3B1	PRELID3B	PRELI domain containing protein 3B OS=Ho	2	104.7	99	94.8	103	101.85	98.9	0.971035837	0.855491
P07814	EPRS	Bifunctional glutamate/proline--tRNA ligase C	55	103.5	100.1	99.2	98.5	101.8	98.85	0.971021611	0.855303
Q9Y2A7	NCKAP1	Nck-associated protein 1 OS=Homo sapiens C	17	101.6	101.6	98.4	98.9	101.6	98.65	0.970964567	0.854552
Q9UN37	VPS4A	Vacuolar protein sorting-associated protein 4A	9	99.4	96.6	92.8	97.5	98	95.15	0.970918367	0.853944
Q6PI78	TMEM65	Transmembrane protein 65 OS=Homo sapiens	1	109.8	103.3	105.5	101.4	106.55	103.45	0.970905678	0.853777
B2RBB2		cDNA, FLJ95416, highly similar to Homo sap	9	102.4	100.3	98.1	98.7	101.35	98.4	0.970892945	0.85361
Q8IZQ5	SELH	Selenoprotein H OS=Homo sapiens GN=SEL	2	106.6	99.2	100.8	99	102.9	99.9	0.970845481	0.852985
Q53H82	LACTB2	Endoribonuclease LACTB2 OS=Homo sapien	7	100	105.8	97.7	102.1	102.9	99.9	0.970845481	0.852985
B2R8W8		cDNA, FLJ94098, highly similar to Homo sap	5	102.4	99.8	100.8	95.5	101.1	98.15	0.970820969	0.852662
Q6ZRS2	SRCAP	Helicase SRCAP OS=Homo sapiens GN=SRC	5	106.5	99	98.7	100.8	102.75	99.75	0.97080292	0.852425
A0A0A0MQS	CDH23	Cadherin-23 OS=Homo sapiens GN=CDH23	2	95.4	106.5	98.2	97.8	100.95	98	0.970777613	0.852092
P46063	RECQL	ATP-dependent DNA helicase Q1 OS=Homo	5	103.6	101.5	99.9	99.2	102.55	99.55	0.970745978	0.851676
A0A0G2JQM	AP2A2	AP-2 complex subunit alpha-2 (Fragment) OS	1	95.5	92.4	96.7	85.7	93.95	91.2	0.970729111	0.851454
Q5JWF2	GNAS	Guanine nucleotide-binding protein G(s) subu	1	107.9	103.9	101	104.6	105.9	102.8	0.970727101	0.851427
Q15814	TBCC	Tubulin-specific chaperone C OS=Homo sapi	3	99.1	102.2	96.9	98.5	100.65	97.7	0.970690512	0.850945
Q9GZP9	DERL2	Derlin-2 OS=Homo sapiens GN=DERL2 PE=	2	104.2	90.2	99.4	89.3	97.2	94.35	0.970679012	0.850794
O14933	UBE2L6	Ubiquitin/ISG15-conjugating enzyme E2 L6 C	1	104.4	100.2	102.1	96.5	102.3	99.3	0.970674487	0.850735
Q00535	CDK5	Cyclin-dependent-like kinase 5 OS=Homo sap	7	96.9	104.2	97.9	97.3	100.55	97.6	0.970661363	0.850562
Q53EW4		Myotubularin-related protein 9 variant (Fragm	2	104.4	103.5	96.3	105.5	103.95	100.9	0.970658971	0.850531
A4D1E9	GTPBP10	GTP-binding protein 10 OS=Homo sapiens G	7	101.7	99.3	97	98.1	100.5	97.55	0.970646766	0.85037
L0R4W3	CHTF8	Alternative protein CHTF8 OS=Homo sapien	1	98.1	102.8	99.4	95.6	100.45	97.5	0.970632155	0.850178
P35221	CTNNA1	Catenin alpha-1 OS=Homo sapiens GN=CTN	12	105.7	98.5	97.1	101.1	102.1	99.1	0.970617042	0.849979
K4ENJ5		Beta actin (Fragment) OS=Homo sapiens PE=	1	109.4	94.4	94.5	103.3	101.9	98.9	0.970559372	0.84922
B2R8U8		cDNA, FLJ94073, highly similar to Homo sap	3	102.1	101.6	101	96.7	101.85	98.85	0.970544919	0.849031
A8K794		cDNA FLJ76308, highly similar to Homo sap	4	100	96.7	95.2	95.7	98.35	95.45	0.970513472	0.848617
Q69YN4	KIAA1429	Protein virilizer homolog OS=Homo sapiens C	13	103.6	99.8	98.1	99.3	101.7	98.7	0.970501475	0.848459
A0A024R1C1	PPIL2	Peptidylprolyl isomerase (Cyclophilin)-like 2,	4	106.4	96.9	101.1	96.2	101.65	98.65	0.970486965	0.848268
O95071	UBR5	E3 ubiquitin-protein ligase UBR5 OS=Homo	13	106.5	100.1	99.2	101.3	103.3	100.25	0.970474347	0.848102
A0A140VJJ2	ESD	S-formylglutathione hydrolase OS=Homo sap	4	97.2	102.5	96.6	97.2	99.85	96.9	0.970455684	0.847857
Q7Z5K2	WAPL	Wings apart-like protein homolog OS=Homo	13	102.9	100	97.7	99.2	101.45	98.45	0.970428783	0.847503
E9PAV3	NACA	Nascent polypeptide-associated complex subu	6	99.1	100.3	97.9	95.6	99.7	96.75	0.970411234	0.847272
Q16513	PKN2	Serine/threonine-protein kinase N2 OS=Homo	12	105.5	97.2	98.7	98	101.35	98.35	0.970399605	0.847119
Q9BTE3	MCMBP	Mini-chromosome maintenance complex-binc	10	101.1	101.5	100.4	96.2	101.3	98.3	0.970384995	0.846927
A0MNP0	WDR51B	CDW9/WDR51B OS=Homo sapiens GN=WI	1	105.8	96.6	102.2	94.2	101.2	98.2	0.970355731	0.846543
Q8NBU5	ATAD1	ATPase family AAA domain-containing prote	9	106.8	101.8	101.3	101.1	104.3	101.2	0.970278044	0.845521
Q9NZL4	HSPBP1	Hsp70-binding protein 1 OS=Homo sapiens C	8	96.5	101.9	94.2	98.3	99.2	96.25	0.970262097	0.845312
A8K4A8		cDNA FLJ76156, highly similar to Homo sap	14	103.8	97.9	96.8	98.9	100.85	97.85	0.970252851	0.84519
A8K0P8		cDNA FLJ78223 OS=Homo sapiens PE=2 SV	8	100.5	101.1	98.2	97.4	100.8	97.8	0.970238095	0.844996
P48426	PIP4K2A	Phosphatidylinositol 5-phosphate 4-kinase typ	2	107.5	100.5	100.2	101.6	104	100.9	0.970192308	0.844394
B4DIS3	LOC84661	Dpy-30-like protein, isoform CRA_b OS=Hor	2	112.9	98.2	105	99.8	105.55	102.4	0.970156324	0.843921
Q9NVH6	TMLHE	Trimethyllysine dioxygenase, mitochondrial C	2	108.3	99.4	102.5	99	103.85	100.75	0.970149254	0.843829
Q6FHV6	ENO2	ENO2 protein OS=Homo sapiens GN=ENO2	12	101.7	102.4	98.4	99.6	102.05	99	0.97011269	0.843348
Q9NP58	ABCB6	ATP-binding cassette sub-family B member 6	9	106.3	94.4	96.3	98.4	100.35	97.35	0.970104634	0.843242
B4DJ38		cDNA FLJ56092, highly similar to Pentatrico	9	101.4	99.3	99	95.7	100.35	97.35	0.970104634	0.843242
A0A024RAF1	BIN1	Bridging integrator 1, isoform CRA_k OS=Ho	5	103.5	100.3	98.8	98.9	101.9	98.85	0.970068695	0.84277
Q4LE60	TNPO2	TNPO2 variant protein (Fragment) OS=Homo	1	84.8	91.8	88.5	82.8	88.3	85.65	0.969988675	0.841719
B2RD36		cDNA, FLJ96437 OS=Homo sapiens PE=2 S7	2	100.1	99.8	95.1	98.8	99.95	96.95	0.969984992	0.84167
B2RCM2		cDNA, FLJ96156, highly similar to Homo sap	31	104.4	102	100.8	99.4	103.2	100.1	0.96996124	0.841358
Q59FZ8		Nebulette non-muscle isoform variant (Fragm	1	99.4	100.2	98.4	95.2	99.8	96.8	0.96993988	0.841078
B3KTV8		cDNA FLJ38830 fis, clone MESAN1000080,	7	100.5	102.1	98	98.5	101.3	98.25	0.969891412	0.840441
B7ZKR9	POLR1A	DNA-directed RNA polymerase subunit OS=l	31	102.3	100.2	98.3	98.1	101.25	98.2	0.969876543	0.840245
Q96D71	REPS1	RalBP1-associated Eps domain-containing pro	3	101.7	104.1	98.4	101.2	102.9	99.8	0.969873664	0.840208
A0A087X266	TMEM120A	Transmembrane protein 120A OS=Homo sapi	1	104.2	101.3	105.3	94	102.75	99.65	0.969829684	0.83963
Q68CQ7	GLT8D1	Glycosyltransferase 8 domain-containing prot	2	103	99.1	97.4	98.6	101.05	98	0.969816922	0.839463
Q16342	PDCD2	Programmed cell death protein 2 OS=Homo s	1	103.1	102.3	101.2	98	102.7	99.6	0.969814995	0.839438
Q969U7	PSMG2	Proteasome assembly chaperone 2 OS=Homo	4	104.2	104.4	99.6	102.7	104.3	101.15	0.969798658	0.839223
Q5SQP8	CTBP2	C-terminal-binding protein 2 OS=Homo sapie	6	105.7	96.2	95.4	100.4	100.95	97.9	0.969787023	0.83907
Q9NRP4	SDHAF3	Succinate dehydrogenase assembly factor 3, n	1	98.7	106	99.1	99.4	102.35	99.25	0.969711773	0.838082
D3DQS4	FNBP4	Formin binding protein 4, isoform CRA_d OS	6	106.6	97.8	97.5	100.7	102.2	99.1	0.969667319	0.837498
P61221	ABCE1	ATP-binding cassette sub-family E member 1	20	103.2	101	99.3	98.7	102.1	99	0.96963761	0.837109
Q13563	PKD2	Polycystin-2 OS=Homo sapiens GN=PKD2 P	4	96.3	97.9	93.4	94.9	97.1	94.15	0.96961895	0.836864
P52306	RAP1GDS1	Rap1 GTPase-GDP dissociation stimulator 1 C	10	98.3	99.1	93.3	98.1	98.7	95.7	0.969604863	0.836679
Q6P2C8	MED27	Mediator of RNA polymerase II transcription	3	105.1	98.8	97.9	99.8	101.95	98.85	0.969592938	0.836522
Q9BXW7	CECR5	Cat eye syndrome critical region protein 5 OS	10	103.7	100.1	99.4	98.2	101.9	98.8	0.969578018	0.836327
Q8IYL3	C1orf174	UPF0688 protein C1orf174 OS=Homo sapien	2	101.4	102.4	98.8	98.8	101.9	98.8	0.969578018	0.836327
D3YTI2	ACP1	Low molecular weight phosphotyrosine protei	1	99.9	100.4	90.6	103.6	100.15	97.1	0.969545681	0.835902
A0A024R6X1	CES2	Carboxylic ester hydrolase OS=Homo sapiens	1	99.5	104	98.9	98.4	101.75	98.65	0.96953317	0.835738
A0A024R6U3	hCG_2025883	HCG2025883, isoform CRA_c OS=Homo sap	4	101.5	98.7	96.4	97.7	100.1	97.05	0.96953047	0.835702
Q15052	ARHGEF6	Rho guanine nucleotide exchange factor 6 OS	1	98.8	91.5	81.1	103.4	95.15	92.25	0.969521808	0.835589
P07737	PFN1	Profilin-1 OS=Homo sapiens GN=PFN1 PE=	12	98.2	101.9	96	98	100.05	97	0.969515242	0.835503
Q53RG0	tmp_locus_9	Eukaryotic translation initiation factor 4E mer	3	97.9	98.9	95.4	95.4	98.4	95.4	0.969512195	0.835463
E9PR47	AAMDC	Mth938 domain-containing protein OS=Homo	1	100.5	96	91.2	99.3	98.25	95.25	0.969465649	0.834852
Q08J23	NSUN2	tRNA (cytosine(34)-C(5))-methyltransferase C	27	103.9	102.2	99.9	99.9	103.05	99.9	0.969432314	0.834414
Q14137	BOP1	Ribosome biogenesis protein BOP1 OS=Hom	20	103.8	99	99.2	97.4	101.4	98.3	0.969428008	0.834358
Q8TB72	PUM2	Pumilio homolog 2 OS=Homo sapiens GN=P	3	102.5	93.6	92.8	97.3	98.05	95.05	0.969403366	0.834035

A0A024R6H0	CPSF2	Cleavage and polyadenylation specific factor	12	100.5	98.8	94.7	98.5	99.65	96.6	0.969392875	0.833897
Q02750	MAP2K1	Dual specificity mitogen-activated protein kin	2	100.7	105.1	94.4	105.1	102.9	99.75	0.969387755	0.83383
Q8WU76	SCFD2	Sec1 family domain-containing protein 2 OS=	1	97.9	111	97.8	104.7	104.45	101.25	0.969363332	0.83351
J3KR97	TBCD	Tubulin-specific chaperone D OS=Homo sapi	2	102	97	99.8	93.1	99.5	96.45	0.969346734	0.833292
P41214	EIF2D	Eukaryotic translation initiation factor 2D OS	6	101.1	101.1	94.6	101.4	101.1	98	0.96933729	0.833168
P26038	MSN	Moesin OS=Homo sapiens GN=MSN PE=1 S	22	100.4	101.5	98.5	97.2	100.95	97.85	0.969291729	0.832571
Q9BX95	SGPP1	Sphingosine-1-phosphate phosphatase 1 OS=I	1	107	91.5	96.2	96.2	99.25	96.2	0.969269521	0.832279
A6H8Y5	NBN	Nibrin OS=Homo sapiens GN=NBN PE=2 SV	8	107.6	97.3	96.1	102.5	102.45	99.3	0.969253294	0.832067
B3KN83	C14orf46	Chromosome 14 open reading frame 46 OS=I	2	107.8	90.5	90.1	102.1	99.15	96.1	0.969238527	0.831873
Q6NZI2	PTRF	Polymerase I and transcript release factor OS=	3	98.2	100.1	94.7	97.5	99.15	96.1	0.969238527	0.831873
O95139	NDUFB6	NADH dehydrogenase [ubiquinone] 1 beta su	3	97.6	103.8	99.6	95.6	100.7	97.6	0.969215492	0.831571
Q8WUA2	PPIL4	Peptidyl-prolyl cis-trans isomerase-like 4 OS=	8	103.3	101.2	98	100.2	102.25	99.1	0.969193154	0.831278
Q3V6T2	CCDC88A	Girdin OS=Homo sapiens GN=CCDC88A PE	1	102.2	115.2	125.2	85.5	108.7	105.35	0.969181233	0.831122
Q15046	KARS	Lysine--tRNA ligase OS=Homo sapiens GN=	21	102.2	102.2	98.7	99.4	102.2	99.05	0.969178082	0.83108
O95486	SEC24A	Protein transport protein Sec24A OS=Homo s	2	103.6	100.6	101.8	96.1	102.1	98.95	0.969147894	0.830685
Q15058	KIF14	Kinesin-like protein KIF14 OS=Homo sapien	10	101.8	102.4	101.1	96.8	102.1	98.95	0.969147894	0.830685
P62312	LSM6	U6 snRNA-associated Sm-like protein LSm6	4	97.6	106.3	96	101.6	101.95	98.8	0.969102501	0.830089
A0A140VJG6	CYLN2	Cytoplasmic linker 2, isoform CRA_b OS=Ho	1	99.6	107.4	102	98.6	103.5	100.3	0.969082126	0.829822
A0A024R994	CPNE3	Copine III, isoform CRA_a OS=Homo sapien	17	99.3	101.2	97	97.3	100.25	97.15	0.969077307	0.829759
Q99543	DNAJC2	DnaJ homolog subfamily C member 2 OS=Ho	6	106.8	100.1	100.9	99.6	103.45	100.25	0.969067182	0.829626
Q12959	DLG1	Disks large homolog 1 OS=Homo sapiens GN	13	106.4	100.5	101	99.5	103.45	100.25	0.969067182	0.829626
A0A075B710	TRAJ39	Protein TRAJ39 (Fragment) OS=Homo sapier	1	88.2	83.1	83.2	82.8	85.65	83	0.969060128	0.829534
M0QZR4	ARHGEF1	Rho guanine nucleotide exchange factor 1 OS	1	107.3	96.2	95.1	102.1	101.75	98.6	0.969041769	0.829293
Q53FN7		BZW1 protein variant (Fragment) OS=Homo	14	101.5	101.9	99.1	98	101.7	98.55	0.969026549	0.829094
Q9BPX3	NCAPG	Condensin complex subunit 3 OS=Homo sapi	15	106.2	100.4	95.6	104.6	103.3	100.1	0.969022265	0.829038
A8K596		cDNA FLJ75087, highly similar to Homo sap	1	96.2	103.9	89.5	104.4	100.05	96.95	0.969015492	0.828949
O60664	PLIN3	Perilipin-3 OS=Homo sapiens GN=PLIN3 PE	12	99.9	100.2	94.4	99.5	100.05	96.95	0.969015492	0.828949
Q8N9F7	GDPD1	Glycerophosphodiester phosphodiesterase dor	1	100.6	105.9	104.1	96	103.25	100.05	0.969007264	0.828841
Q12830	BPTF	Nucleosome-remodeling factor subunit BPTF	5	104.4	98.8	99.6	97.3	101.6	98.45	0.968996063	0.828694
P78318	IGBP1	Immunoglobulin-binding protein 1 OS=Homo	2	99	100.8	96.1	97.5	99.9	96.8	0.968968969	0.82834
A0A109NGN(		Proteasome subunit alpha type OS=Homo sap	12	101.9	101	98.8	97.8	101.45	98.3	0.968950222	0.828094
Q9BST9	RTKN	Rhotekin OS=Homo sapiens GN=RTKN PE=	8	98.4	98	94.4	95.9	98.2	95.15	0.968940937	0.827972
Q8IYI6	EXOC8	Exocyst complex component 8 OS=Homo sap	4	102.2	97.4	96.1	97.3	99.8	96.7	0.968937876	0.827932
Q15370	TCEB2	Transcription elongation factor B polypeptide	6	98.7	103.8	99.1	97.1	101.25	98.1	0.968888889	0.82729
P54802	NAGLU	Alpha-N-acetylglucosaminidase OS=Homo sa	1	86.1	87.4	75.6	92.5	86.75	84.05	0.968876081	0.827122
O75486	SUPT3H	Transcription initiation protein SPT3 homolog	2	107.2	95.1	96.8	99.2	101.15	98	0.968858131	0.826887
B5ME19	EIF3CL	Eukaryotic translation initiation factor 3 subu	22	104.6	103.9	102.5	99.5	104.25	101	0.96882494	0.826452
Q53F47		Down-regulator of transcription 1 variant (Fra	4	102.5	96	94.9	97.4	99.25	96.15	0.968765743	0.825677
Q15269	PWP2	Periodic tryptophan protein 2 homolog OS=H	1	103.5	101.4	98.7	99.8	102.45	99.25	0.968765251	0.825671
Q59GM9		Alpha-1,4 glucan phosphorylase (Fragment) C	16	104.1	100.5	99.8	98.4	102.3	99.1	0.968719453	0.825071
A8K8N3		cDNA FLJ78740, highly similar to Homo sap	7	100.3	101.1	94.5	100.6	100.7	97.55	0.968718967	0.825064
P14174	MIF	Macrophage migration inhibitory factor OS=I	3	93.6	104.6	94.6	97.4	99.1	96	0.968718466	0.825058
Q92522	H1FX	Histone H1x OS=Homo sapiens GN=H1FX P	7	97.9	100.3	94.7	97.3	99.1	96	0.968718466	0.825058
Q8TF05	PPP4R1	Serine/threonine-protein phosphatase 4 regula	5	100.5	100.8	97.6	97.4	100.65	97.5	0.968703428	0.824861
Q9UEW8	STK39	STE20/SPS1-related proline-alanine-rich prot	4	101.5	102.7	97.2	100.6	102.1	98.9	0.968658178	0.824268
Q9NP66	HMG20A	High mobility group protein 20A OS=Homo s	4	105.8	95.2	97.6	97.1	100.5	97.35	0.968656716	0.824249
Q2NKX8	ERCC6L	DNA excision repair protein ERCC-6-like OS	4	102.8	95	94.5	97.1	98.9	95.8	0.968655207	0.82423
Q92570	NR4A3	Nuclear receptor subfamily 4 group A membe	1	97.6	96.7	92.8	95.4	97.15	94.1	0.96860525	0.823575
Q68D10	SPTY2D1	Protein SPT2 homolog OS=Homo sapiens GN	2	96.4	107.4	96.5	100.9	101.9	98.7	0.968596663	0.823463
H3BS01	C15orf41	Uncharacterized protein C15orf41 OS=Homo	1	94.5	106.1	98.3	96	100.3	97.15	0.968594217	0.823431
P50897	PPT1	Palmitoyl-protein thioesterase 1 OS=Homo sa	5	103.4	97.2	93.5	100.8	100.3	97.15	0.968594217	0.823431
Q14966	ZNF638	Zinc finger protein 638 OS=Homo sapiens GN	21	104.2	99.4	99.7	97.5	101.8	98.6	0.968565815	0.823059
B7Z6Q5		Beta-galactosidase OS=Homo sapiens PE=2 S	9	103.2	100.4	100.4	96.8	101.8	98.6	0.968565815	0.823059
Q13442	PDAP1	28 kDa heat- and acid-stable phosphoprotein (	5	100.5	106.2	101.1	99.1	103.35	100.1	0.968553459	0.822898
Q53LP3	SOWAHC	Ankyrin repeat domain-containing protein SO	1	122.8	99.7	96.7	118.8	111.25	107.75	0.968539326	0.822712
A0A140VJP2		Testicular tissue protein Li 118 OS=Homo sap	10	97.8	102.4	95.4	98.5	100.1	96.95	0.968531469	0.82261
P49821	NDUFV1	NADH dehydrogenase [ubiquinone] flavoprot	11	104.8	98.2	99.2	97.4	101.5	98.3	0.968472906	0.821843
A5D8W8	GGT7	Gamma-glutamyltransferase 7 OS=Homo sap	1	99.7	90.5	86	98.2	95.1	92.1	0.968454259	0.821599
Q14692	BMS1	Ribosome biogenesis protein BMS1 homolog	20	103.7	99	100	96.3	101.35	98.15	0.968426246	0.821233
F8VXU5	VPS29	Vacuolar protein sorting-associated protein 29	7	101.9	100.4	100.4	95.5	101.15	97.95	0.968363816	0.820416
Q5LJA5	UCHL5	Ubiquitin carboxyl-terminal hydrolase OS=Ho	8	102.3	102.9	100.8	97.9	102.6	99.35	0.968323587	0.81989
V9HWK0	HEL103	Signal recognition particle subunit SRP72 OS	2	103.2	98.8	97.6	98	101	97.8	0.968316832	0.819802
P45985	MAP2K4	Dual specificity mitogen-activated protein kin	2	95.5	100.1	90	99.4	97.8	94.7	0.968302658	0.819616
Q5U043		S-(hydroxymethyl)glutathione dehydrogenase	12	99.8	102.1	97.5	98	100.95	97.75	0.968301139	0.819596
Q96CE8	TM4SF18	Transmembrane 4 L6 family member 18 OS=	1	115.6	95.4	105	99.3	105.5	102.15	0.968246445	0.818881
Q5HYI7	MTX3	Metaxin-3 OS=Homo sapiens GN=MTX3 PE	2	103.3	101.4	101.2	97	102.35	99.1	0.968246214	0.818878
A0A024R2H2	TSEN2	tRNA-splicing endonuclease subunit Sen2 OS	1	102.9	101.8	90.9	107.3	102.35	99.1	0.968246214	0.818878
Q5JRA6	MIA3	Melanoma inhibitory activity protein 3 OS=H	25	101.9	99	97.5	97	100.45	97.25	0.968143355	0.817533
P51511	MMP15	Matrix metalloproteinase-15 OS=Homo sapie	1	102.3	101.4	100	97.2	101.85	98.6	0.968090329	0.81684
P13612	ITGA4	Integrin alpha-4 OS=Homo sapiens GN=ITGA	1	107.6	102.3	103.7	99.5	104.95	101.6	0.968080038	0.816705
P33316	DUT	Deoxyuridine 5'-triphosphate nucleotidohydro	8	97.3	103.2	99.7	94.4	100.25	97.05	0.9680798	0.816702
A0A087WXU	ESYT2	Extended synaptotagmin-2 OS=Homo sapiens	13	103.3	100.3	97.1	100	101.8	98.55	0.968074656	0.816635
Q96HR3	MED30	Mediator of RNA polymerase II transcription	2	107.1	99.6	99.9	100.2	103.35	100.05	0.968069666	0.816569
Q8TEY7	USP33	Ubiquitin carboxyl-terminal hydrolase 33 OS=	1	109	97.5	98.9	101	103.25	99.95	0.968038741	0.816165
P61599	NAA20	N-alpha-acetyltransferase 20 OS=Homo sapie	3	104.9	98.4	95	101.8	101.65	98.4	0.968027545	0.816019
P53370	NUDT6	Nucleoside diphosphate-linked moiety X moti	1	101.2	105.2	94.4	105.4	103.2	99.9	0.968023256	0.815963
P28288	ABCD3	ATP-binding cassette sub-family D member 3	14	102.1	101.1	99.4	97.3	101.6	98.35	0.968011811	0.815813
Q4G0N4	NADK2	NAD kinase 2, mitochondrial OS=Homo sapi	11	104.3	98.7	98.4	98.1	101.5	98.25	0.967980296	0.815402
Q8NC51	SERBP1	Plasminogen activator inhibitor 1 RNA-bindin	17	104	105.1	102.4	100	104.55	101.2	0.967957915	0.815109
Q96EZ8	MCRS1	Microspherule protein 1 OS=Homo sapiens G	2	105	91.6	90.7	99.6	98.3	95.15	0.967955239	0.815074
A0A024R0M6	TIMM50	Translocase of inner mitochondrial membrane	8	103.9	98.8	97.9	98.3	101.35	98.1	0.967932906	0.814782
Q9BU23	LMF2	Lipase maturation factor 2 OS=Homo sapiens	2	100.5	95.9	96	94.1	98.2	95.05	0.967922607	0.814648
A0A087WU53	MAGT1	Magnesium transporter protein 1 OS=Homo s	6	103	99.2	98.6	97.1	101.1	97.85	0.96785361	0.813746
Q6IBR2	FARSLA	FARSLA protein OS=Homo sapiens GN=FAI	12	104.3	100.9	99.8	98.8	102.6	99.3	0.967836257	0.813519
O96019	ACTL6A	Actin-like protein 6A OS=Homo sapiens GN=	13	101.4	100.6	97.1	98.4	101	97.75	0.967821782	0.81333
Q567U6	CCDC93	Coiled-coil domain-containing protein 93 OS=	3	101.5	106.7	100.2	101.3	104.1	100.75	0.967819404	0.813299
Q4LE64	NUMA1	NUMA1 variant protein (Fragment) OS=Hom	81	102.9	98.9	98.5	96.8	100.9	97.65	0.967789891	0.812914
Q15007	WTAP	Pre-mRNA-splicing regulator WTAP OS=Ho	7	105.9	99	99.9	98.4	102.45	99.15	0.967789165	0.812904
O94915	FRYL	Protein furry homolog-like OS=Homo sapiens	7	102	102.9	100	98.3	102.45	99.15	0.967789165	0.812904
Q9Y4W2	LAS1L	Ribosomal biogenesis protein LAS1L OS=Ho	13	104.8	96.9	99.1	96.1	100.85	97.6	0.967773922	0.812705
Q86XP3	DDX42	ATP-dependent RNA helicase DDX42 OS=H	28	102.7	101.6	99.5	98.2	102.15	98.85	0.967694567	0.811669
Q9Y2P8	RCL1	RNA 3'-terminal phosphate cyclase-like prote	9	104.4	99.8	98.7	98.9	102.1	98.8	0.967678746	0.811462
Q8WV19	SFT2D1	Vesicle transport protein SFT2A OS=Homo s	1	113.5	90.7	98.5	99.1	102.1	98.8	0.967678746	0.811462
Q8TDQ7	GNPDA2	Glucosamine-6-phosphate isomerase 2 OS=H	3	97.5	103.6	94.6	100	100.55	97.3	0.967677772	0.81145
A0A024RC37	P15RS	Uncharacterized protein OS=Homo sapiens G	4	99.4	101.3	96	98.2	100.35	97.1	0.967613353	0.810608
Q13330	MTA1	Metastasis-associated protein MTA1 OS=Hor	18	104.2	99.5	100.3	96.8	101.85	98.55	0.967599411	0.810426
A0A024R667	C14orf150	Chromosome 14 open reading frame 150, isof	4	102.7	100.6	97.5	99.2	101.65	98.35	0.967535662	0.809595
A0A024R4E1	NMNAT1	Nicotinamide-nucleotide adenylyltransferase (	2	103.8	102.3	105.4	94	103.05	99.7	0.967491509	0.809018
Q8IX18	DHX40	Probable ATP-dependent RNA helicase DHX	1	101.5	101.5	94.4	102	101.5	98.2	0.967487685	0.808969
Q3KQV9	UAP1L1	UDP-N-acetylhexosamine pyrophosphorylase	1	99.4	109.7	102.1	100.2	104.55	101.15	0.967479675	0.808864
Q15811	ITSN1	Intersectin-1 OS=Homo sapiens GN=ITSN1 I	1	114.6	94.4	83.6	118.6	104.5	101.1	0.967464115	0.808661
K7EK56	C19orf25	UPF0449 protein C19orf25 OS=Homo sapien	3	105.3	97.5	99.3	96.9	101.4	98.1	0.967455621	0.80855
Q99615	DNAJC7	DnaJ homolog subfamily C member 7 OS=Ho	15	100.7	102.1	98	98.2	101.4	98.1	0.967455621	0.80855
P49720	PSMB3	Proteasome subunit beta type-3 OS=Homo sa	9	105.2	100.4	99.8	99.1	102.8	99.45	0.967412451	0.807987
Q13625	TP53BP2	Apoptosis-stimulating of p53 protein 2 OS=H	1	108.5	97	97.8	101	102.75	99.4	0.967396594	0.80778
B1AKN8	NFIA	Nuclear factor 1 OS=Homo sapiens GN=NFIA	1	104.1	101.3	93.5	105.2	102.7	99.35	0.967380721	0.807573
Q15392	DHCR24	Delta(24)-sterol reductase OS=Homo sapiens	8	105.6	99.5	102.6	95.8	102.55	99.2	0.967333008	0.806951
Q9H2U1	DHX36	ATP-dependent RNA helicase DHX36 OS=H	6	107.9	99.8	99	101.9	103.85	100.45	0.967260472	0.806005
Q96F63	CCDC97	Coiled-coil domain-containing protein 97 OS=	3	103.5	104.2	99.4	101.5	103.85	100.45	0.967260472	0.806005
A8K7A1		cDNA FLJ77346, highly similar to Homo sap	11	104.5	100.1	97	100.9	102.3	98.95	0.967253177	0.805909
A0A0S2Z652	COG7	Component of oligomeric golgi complex 7 isc	3	98.2	103.2	94.9	99.9	100.7	97.4	0.967229394	0.805599
O76046	SKI2W	Putative RNA helicase Ski2w OS=Homo sapi	3	106.3	101	96.8	103.7	103.65	100.25	0.967197299	0.805181
A4D0W4	AASS	Aminoadipate-semialdehyde synthase OS=Ho	4	107.3	100	101.9	98.6	103.65	100.25	0.967197299	0.805181
A0A0S2Z5M8	ELAC2	ElaC homolog 2 (E. coli), isoform CRA_b (Fr	16	104.1	100.1	98.6	98.9	102.1	98.75	0.96718903	0.805073
V9HWE9	HEL-S-22	Epididymis secretory protein Li 22 OS=Homo	10	98.5	99.6	94.2	97.4	99.05	95.8	0.967188289	0.805064
A0A0A0MTH	ILK	Integrin-linked protein kinase OS=Homo sapi	11	101.9	102.2	98.7	98.7	102.05	98.7	0.967172954	0.804864
A0A024R1K7	YWHAH	Tyrosine 3-monooxygenase/tryptophan 5-mon	10	102.7	101.3	98	99.3	102	98.65	0.967156863	0.804654
A0A024R539	LOC51035	Uncharacterized protein OS=Homo sapiens G	5	97.7	100.2	93.6	97.8	98.95	95.7	0.967155129	0.804632
Q8IZK6	MCOLN2	Mucolipin-2 OS=Homo sapiens GN=MCOLN	2	113.3	93.6	109.1	91	103.45	100.05	0.967133881	0.804354
Q5VZU9	TPP2	Tripeptidyl-peptidase 2 OS=Homo sapiens GN	7	98.2	99.4	94	97.1	98.8	95.55	0.967105263	0.803981
A0A024R329	GMPPB	GDP-mannose pyrophosphorylase B, isoform	6	99.7	100.9	93.8	100.2	100.3	97	0.967098704	0.803896
P25789	PSMA4	Proteasome subunit alpha type-4 OS=Homo s	12	104.2	102.1	101.1	98.4	103.15	99.75	0.967038294	0.803108

Q9C0C9	UBE2O	(E3-independent) E2 ubiquitin-conjugating en	8	97.9	105.2	97.4	99	101.55	98.2	0.967011324	0.802757
Q13596	SNX1	Sorting nexin-1 OS=Homo sapiens GN=SNX	4	103.5	99.5	97.3	99	101.5	98.15	0.966995074	0.802545
Q86UY8	NT5DC3	5'-nucleotidase domain-containing protein 3 C	1	95	98.7	86.5	100.8	96.85	93.65	0.966959215	0.802078
A0A024R2T0	USP4	Ubiquitin specific peptidase 4 (Proto-oncogen	1	103.5	93.2	101.3	88.9	98.35	95.1	0.966954753	0.80202
Q9NYD6	HOXC10	Homeobox protein Hox-C10 OS=Homo sapie	2	99.1	100.4	96.2	96.7	99.75	96.45	0.966917293	0.801532
P62195	PSMC5	26S protease regulatory subunit 8 OS=Homo	23	104.7	100.8	100.3	98.4	102.75	99.35	0.966909976	0.801437
Q6FGH5	RPS21	RPS21 protein (Fragment) OS=Homo sapiens	6	101.1	104.2	97.9	100.6	102.65	99.25	0.96687774	0.801017
B2RCT6		cDNA, FLJ96276, highly similar to Homo sap	8	102.7	102.4	101	97.3	102.55	99.15	0.966845441	0.800596
P55771	PAX9	Paired box protein Pax-9 OS=Homo sapiens C	7	100.5	101.5	96.5	98.8	101	97.65	0.966831683	0.800417
E5KMT5		tRNA pseudouridine synthase OS=Homo sapi	8	104.4	97.5	98.6	96.6	100.95	97.6	0.966815255	0.800203
A8K037		cDNA FLJ77641 OS=Homo sapiens PE=2 SV	1	108.8	96	93.4	104.6	102.4	99	0.966796875	0.799964
Q9NQT5	EXOSC3	Exosome complex component RRP40 OS=Ho	9	102.4	102.2	97.9	99.9	102.3	98.9	0.966764418	0.799541
Q68D91	MBLAC2	Metallo-beta-lactamase domain-containing pr	6	98.2	103.3	97.3	97.5	100.75	97.4	0.96674938	0.799345
A6NFX8	NUDT5	ADP-sugar pyrophosphatase OS=Homo sapie	10	100.8	103.6	97.4	100.2	102.2	98.8	0.966731898	0.799118
Q6P4E1	CASC4	Protein CASC4 OS=Homo sapiens GN=CASC	6	102.9	98.4	96.2	98.4	100.65	97.3	0.966716344	0.798915
Q4LE40	C14orf159	C14orf159 variant protein (Fragment) OS=Ho	2	99.2	104.9	100.7	96.6	102.05	98.65	0.966682999	0.798481
A8K5R1	RAB30	RAB30, member RAS oncogene family, isofo	1	99.8	101.2	103.8	90.5	100.5	97.15	0.966666667	0.798268
Q9H061	TMEM126A	Transmembrane protein 126A OS=Homo sapi	6	102.7	101.2	99.8	97.3	101.95	98.55	0.966650319	0.798056
P07384	CAPN1	Calpain-1 catalytic subunit OS=Homo sapiens	16	98.9	102	97.8	96.4	100.45	97.1	0.966650075	0.798053
H7C4X9	ZMYND8	Protein kinase C-binding protein 1 (Fragment)	5	101	102.8	99.7	97.3	101.9	98.5	0.966633955	0.797843
A0A024R216	HDGFRP3	Hepatoma-derived growth factor, related prote	1	105.3	89.1	90.2	97.7	97.2	93.95	0.966563786	0.79693
P49736	MCM2	DNA replication licensing factor MCM2 OS=	39	102.1	101.2	98	98.5	101.65	98.25	0.966551894	0.796775
Q9Y3Y2	CHTOP	Chromatin target of PRMT1 protein OS=Hom	5	105.8	100.4	100.2	99.1	103.1	99.65	0.966537342	0.796586
B2RDN3	NUBP2	Cytosolic Fe-S cluster assembly factor NUBP	3	101	99.2	92.3	101.2	100.1	96.75	0.966533467	0.796536
B3KTT0		cDNA FLJ38675 fis, clone IMR322000243, h	7	104	102.1	102.4	96.8	103.05	99.6	0.966521106	0.796374
Q8NFI3	ENGASE	Cytosolic endo-beta-N-acetylglucosaminidase	1	108.6	93.8	88.7	106.9	101.2	97.8	0.966403162	0.794841
B3KN09		cDNA FLJ13173 fis, clone NT2RP3003831, l	5	108.3	94.1	97.6	98	101.2	97.8	0.966403162	0.794841
O00273	DFFA	DNA fragmentation factor subunit alpha OS=	7	100.9	101	94.9	100.2	100.95	97.55	0.96631996	0.793759
E9PMV1	PLEC	Plectin (Fragment) OS=Homo sapiens GN=PI	1	94.2	101.7	94.6	94.7	97.95	94.65	0.966309342	0.793621
A6XND5		Ribonuclease T2 OS=Homo sapiens PE=2 SV	1	104	97.8	98.3	96.7	100.9	97.5	0.966303271	0.793542
A0A024R9W5	HUWE1	HECT, UBA and WWE domain containing 1,	21	101.9	102.6	98.7	98.9	102.25	98.8	0.966259169	0.792969
Q9H6E4	CCDC134	Coiled-coil domain-containing protein 134 OS	6	104.4	99.9	98.1	99.3	102.15	98.7	0.966226138	0.79254
Q86WV7	CCDC43	CCDC43 protein OS=Homo sapiens GN=CCI	2	108	99	100.2	99.8	103.5	100	0.966183575	0.791986
A2RUF3	SRGAP2	SLIT-ROBO Rho GTPase activating protein 2	6	97.5	100.6	93.4	98	99.05	95.7	0.966178698	0.791923
A8K6A2		cDNA FLJ77317, highly similar to Homo sap	1	89.6	114.4	92.1	105	102	98.55	0.966176471	0.791894
A0A024R904	CACYBP	Calcyclin binding protein, isoform CRA_a OS	15	98.6	102.3	97.1	97	100.45	97.05	0.966152315	0.79158
Q53H77		Mitochondrial ribosomal protein S30 variant (	10	103.4	100.3	99.7	97.1	101.85	98.4	0.966126657	0.791247
Q9NZJ6	COQ3	Ubiquinone biosynthesis O-methyltransferase	2	103.1	97.6	97	96.9	100.35	96.95	0.966118585	0.791142
P14550	AKR1A1	Alcohol dehydrogenase [NADP(+)] OS=Hom	6	96.5	101.1	98.1	92.8	98.8	95.45	0.966093117	0.790811
Q9H981	ACTR8	Actin-related protein 8 OS=Homo sapiens GN	4	109.9	99.2	103	99	104.55	101	0.966044955	0.790185
Q8NEM7	SUPT20H	Transcription factor SPT20 homolog OS=Hor	2	101	102.1	98.7	97.5	101.55	98.1	0.966026588	0.789947
Q7Z6I8	C5orf24	UPF0461 protein C5orf24 OS=Homo sapiens	1	96.7	103.2	93.2	99.9	99.95	96.55	0.965982991	0.78938
Q8IWB7	WDFY1	WD repeat and FYVE domain-containing pro	12	102.9	99.9	98.4	97.5	101.4	97.95	0.965976331	0.789294
A0A024R2P4	C3orf39	Chromosome 3 open reading frame 39, isofor	3	103.9	101.7	97.7	100.9	102.8	99.3	0.965953307	0.788995
O95365	ZBTB7A	Zinc finger and BTB domain-containing prote	2	100.1	99.6	94.9	98	99.85	96.45	0.965948923	0.788938
Q8NHQ9	DDX55	ATP-dependent RNA helicase DDX55 OS=H	6	104.4	100.8	98.5	99.7	102.6	99.1	0.96588694	0.788134
Q5TGZ0	MINOS1	MICOS complex subunit MIC10 OS=Homo s	1	107.9	99.9	104.3	96.4	103.9	100.35	0.965832531	0.787427
Q13526	PIN1	Peptidyl-prolyl cis-trans isomerase NIMA-inte	5	101.5	100.3	91.4	103.5	100.9	97.45	0.96580773	0.787105
O15294	OGT	UDP-N-acetylglucosamine--peptide N-acetylg	17	104.4	100.2	97.6	100	102.3	98.8	0.965786901	0.786835
O95260	ATE1	Arginyl-tRNA--protein transferase 1 OS=Hon	1	105.2	96.1	93.9	100.5	100.65	97.2	0.965722802	0.786003
B3KMP2		cDNA FLJ11747 fis, clone HEMBA1005530,	12	102.5	98.6	95.6	98.6	100.55	97.1	0.965688712	0.785561
A0A024R1S7	SMARCE1	SWI/SNF related, matrix associated, actin dep	2	100.4	103.6	95.3	101.7	102	98.5	0.965686275	0.785529
P50851	LRBA	Lipopolysaccharide-responsive and beige-like	9	101	99.9	97.7	96.3	100.45	97	0.965654555	0.785118
O00268	TAF4	Transcription initiation factor TFIID subunit 4	3	105.2	104.2	100.2	102	104.7	101.1	0.965616046	0.784619
Q9UJX6	ANAPC2	Anaphase-promoting complex subunit 2 OS=I	3	100.9	99.7	97.5	96.2	100.3	96.85	0.96560319	0.784452
O95372	LYPLA2	Acyl-protein thioesterase 2 OS=Homo sapiens	5	103	100.3	98.4	97.9	101.65	98.15	0.965568126	0.783997
Q8NAV1	PRPF38A	Pre-mRNA-splicing factor 38A OS=Homo sa	4	102.5	103.6	100.9	98.1	103.05	99.5	0.965550704	0.783772
P49773	HINT1	Histidine triad nucleotide-binding protein 1 O	7	95.3	104.8	92.8	100.4	100.05	96.6	0.965517241	0.783337
P63261	ACTG1	Actin, cytoplasmic 2 OS=Homo sapiens GN=	3	110	92.9	99.3	96.6	101.45	97.95	0.965500246	0.783117
Q92769	HDAC2	Histone deacetylase 2 OS=Homo sapiens GN	10	100.9	102	98.3	97.6	101.45	97.95	0.965500246	0.783117
Q13951	CBFB	Core-binding factor subunit beta OS=Homo sa	5	99.5	103.4	96.8	99.1	101.45	97.95	0.965500246	0.783117
Q8TD16	BICD2	Protein bicaudal D homolog 2 OS=Homo sapi	6	104.4	101.3	97.7	100.9	102.85	99.3	0.965483714	0.782903
Q15654	TRIP6	Thyroid receptor-interacting protein 6 OS=Ho	11	100.1	99.8	95.8	97.2	99.95	96.5	0.965482741	0.78289
Q92917	GPKOW	G patch domain and KOW motifs-containing	16	102.5	100.2	98.2	97.5	101.35	97.85	0.965466206	0.782676
P02545	LMNA	Prelamin-A/C OS=Homo sapiens GN=LMNA	4	100.9	98.7	97.2	95.5	99.8	96.35	0.965430862	0.782218
Q9UIF9	BAZ2A	Bromodomain adjacent to zinc finger domain	17	100	96.3	95.5	94	98.15	94.75	0.965359144	0.781289
Q9NZ43	USE1	Vesicle transport protein USE1 OS=Homo sa	2	104.9	100	99	98.8	102.45	98.9	0.965348951	0.781157
Q96PU8	QKI	Protein quaking OS=Homo sapiens GN=QKI	8	107.8	97	97.6	100.1	102.4	98.85	0.965332031	0.780937
Q8N523	TFIP11	Tuftelin-interacting protein 11 OS=Homo sap	16	106.2	98.4	98.9	98.6	102.3	98.75	0.965298143	0.780498
Q9P2N5	RBM27	RNA-binding protein 27 OS=Homo sapiens C	10	104.9	102.5	102.2	98	103.7	100.1	0.965284474	0.780321
A0A024QZF8	EMP3	Epithelial membrane protein 3 OS=Homo sap	1	112.8	94.4	100.6	99.4	103.6	100	0.965250965	0.779887
A0A024RC00	CTPS2	CTP synthase OS=Homo sapiens GN=CTPS2	8	101.9	99.2	96.5	97.6	100.55	97.05	0.965191447	0.779116
A0A024R6A3	PSEN1	Presenilin OS=Homo sapiens GN=PSEN1 PE	2	103.1	103.7	98.4	101.2	103.4	99.8	0.965183752	0.779017
A0A024RAC5	RCC2	Regulator of chromosome condensation 2, iso	15	103.8	103	97.3	102.3	103.4	99.8	0.965183752	0.779017
A0A024R850	MRRF	Mitochondrial ribosome recycling factor, isofo	5	98.7	99.3	95.7	95.4	99	95.55	0.965151515	0.778599
X6R8Y7	SPIN4	Spindlin-4 OS=Homo sapiens GN=SPIN4 PE	1	109.6	97	88.3	111.1	103.3	99.7	0.965150048	0.778581
P06454	PTMA	Prothymosin alpha OS=Homo sapiens GN=P'	2	99.5	98.4	93	98	98.95	95.5	0.965133906	0.778372
O75179	ANKRD17	Ankyrin repeat domain-containing protein 17	8	103.9	102.6	99.7	99.6	103.25	99.65	0.965133172	0.778362
B3KN29		PRA1 family protein OS=Homo sapiens PE=:	2	99.3	98.5	92.5	98.4	98.9	95.45	0.965116279	0.778143
P04040	CAT	Catalase OS=Homo sapiens GN=CAT PE=1 S	16	101.3	99.3	96.3	97.3	100.3	96.8	0.965104686	0.777993
Q9NXS2	QPCTL	Glutaminyl-peptide cyclotransferase-like prote	4	96.5	106.9	100.5	95.8	101.7	98.15	0.965093412	0.777848
J7IHS8	MICA	MHC class I chain-related protein A OS=Hom	6	105.2	101	100.5	98.5	103.1	99.5	0.965082444	0.777706
Q5T0G8	ANXA11	Annexin OS=Homo sapiens GN=ANXA11 PI	12	103.4	99.9	99.7	96.5	101.65	98.1	0.965076242	0.777625
B2R9T9		cDNA, FLJ94551 OS=Homo sapiens PE=2 S'	3	106.2	99.9	100.9	98	103.05	99.45	0.965065502	0.777486
Q9Y2S0	POLR1D	DNA-directed RNA polymerases I and III sub	2	105.3	103.4	100	101.4	104.35	100.7	0.965021562	0.776917
Q53EP0	FNDC3B	Fibronectin type III domain-containing protei	12	102.5	103.3	102.7	95.9	102.9	99.3	0.965014577	0.776827
Q96H20	SNF8	Vacuolar-sorting protein SNF8 OS=Homo sap	3	107.8	94.8	98.2	97.3	101.3	97.75	0.964955577	0.776064
Q15008	PSMD6	26S proteasome non-ATPase regulatory subu	19	103.9	101.5	99.9	98.3	102.7	99.1	0.964946446	0.775946
P57721	PCBP3	Poly(rC)-binding protein 3 OS=Homo sapiens	1	102.8	102.3	101	96.9	102.55	98.95	0.964895173	0.775283
Q9H9S3	SEC61A2	Protein transport protein Sec61 subunit alpha	2	98.6	100.5	97	95.1	99.55	96.05	0.964841788	0.774592
Q9H2J4	PDCL3	Phosducin-like protein 3 OS=Homo sapiens C	7	103.8	100.9	99.3	98.2	102.35	98.75	0.964826575	0.774395
Q99497	PARK7	Protein deglycase DJ-1 OS=Homo sapiens GN	13	98.1	100.6	96.2	95.5	99.35	95.85	0.964771012	0.773677
P68371	TUBB4B	Tubulin beta-4B chain OS=Homo sapiens GN	2	96.9	104.4	96.7	97.5	100.65	97.1	0.96472926	0.773137
Q7KZ85	SUPT6H	Transcription elongation factor SPT6 OS=Hor	30	104.9	99.2	96.8	100.1	102.05	98.45	0.964723175	0.773058
Q9C0C2	TNKS1BP1	182 kDa tankyrase-1-binding protein OS=Hor	23	105.8	103.8	101.2	101	104.8	101.1	0.964694656	0.77269
Q5VWW1	C1QL3	Complement C1q-like protein 3 OS=Homo sa	1	94	107.1	97.8	96.2	100.55	97	0.964694182	0.772684
O00193	SMAP	Small acidic protein OS=Homo sapiens GN=S	3	105.6	98.3	100.3	96.4	101.95	98.35	0.964688573	0.772612
A0A024RC06	MTM1	Myotubularin 1, isoform CRA_a OS=Homo s	1	101.5	96.4	95.2	95.7	98.95	95.45	0.9646286	0.771836
Q9HAB8	PPCS	Phosphopantothenate--cysteine ligase OS=Ho	4	100.3	100.4	100.9	92.7	100.35	96.8	0.964623817	0.771774
Q96P70	IPO9	Importin-9 OS=Homo sapiens GN=IPO9 PE=	17	99.8	100.6	94.3	99	100.2	96.65	0.964570858	0.77109
P47224	RABIF	Guanine nucleotide exchange factor MSS4 OS	2	103.6	102	96.6	101.7	102.8	99.15	0.964494163	0.7701
O76081	RGS20	Regulator of G-protein signaling 20 OS=Hom	1	104.4	89.9	87.9	99.5	97.15	93.7	0.964487905	0.770019
Q92520	FAM3C	Protein FAM3C OS=Homo sapiens GN=FAM	9	105.9	107.5	104	101.8	106.7	102.9	0.964386129	0.768704
Q16629	SRSF7	Serine/arginine-rich splicing factor 7 OS=Hor	9	101.4	100.5	95.5	99.2	100.95	97.35	0.964338782	0.768093
P61160	ACTR2	Actin-related protein 2 OS=Homo sapiens GN	11	101.3	103.2	99.4	97.8	102.25	98.6	0.964303178	0.767634
A0A068F7M9	FHOD1	FH1/FH2 domain-containing protein 1 variant	7	102.8	104.5	100.4	99.5	103.65	99.95	0.964302943	0.767631
Q6FGG2	VAMP3	VAMP3 protein OS=Homo sapiens GN=VAM	2	104.9	99.5	100.9	96.2	102.2	98.55	0.964285714	0.767409
O95251	KAT7	Histone acetyltransferase KAT7 OS=Homo sa	2	99.7	98.8	96.5	94.9	99.25	95.7	0.964231738	0.766712
A0A087WYN	DHX29	ATP-dependent RNA helicase DHX29 OS=H	13	96.8	104.3	96.1	97.8	100.55	96.95	0.964196917	0.766263
P13056	NR2C1	Nuclear receptor subfamily 2 group C membe	4	98.2	97.2	93.1	95.3	97.7	94.2	0.964176049	0.765994
A8K5C5		cDNA FLJ77534, highly similar to Homo sap	5	103.6	100.1	98.5	97.9	101.85	98.2	0.964162985	0.765826
Q6IAA8	LAMTOR1	Ragulator complex protein LAMTOR1 OS=H	5	110.9	98.3	101.5	100.2	104.6	100.85	0.96414914	0.765647
M0QYZ2	AP2S1	AP-2 complex subunit sigma OS=Homo sapie	7	104.3	99.3	99.2	97.1	101.8	98.15	0.964145383	0.765598
Q8NBX0	SCCPDH	Saccharopine dehydrogenase-like oxidoreduct	8	102.9	100.6	98.4	97.8	101.75	98.1	0.964127764	0.765371
P63151	PPP2R2A	Serine/threonine-protein phosphatase 2A 55 k	8	102.7	100.8	97.2	99	101.75	98.1	0.964127764	0.765371
Q9BVL4	SELO	Selenoprotein O OS=Homo sapiens GN=SEL	1	113.3	92.8	105.3	93.4	103.05	99.35	0.964095099	0.76495
P38935	IGHMBP2	DNA-binding protein SMUBP-2 OS=Homo s	2	103.5	91.3	93.1	94.7	97.4	93.9	0.964065708	0.764571
D3DW56	UBL7	Ubiquitin-like 7 (Bone marrow stromal cell-de	2	105.4	100.4	94.5	103.9	102.9	99.2	0.96404276	0.764275
E5RFJ8	MCM4	DNA replication licensing factor MCM4 (Frag	1	109.3	93.7	103.4	92.3	101.5	97.85	0.964039409	0.764232
Q9NVC3	SLC38A7	Putative sodium-coupled neutral amino acid t	1	117.6	79.7	105.2	85	98.65	95.1	0.964014192	0.763907
Q6P2E9	EDC4	Enhancer of mRNA-decapping protein 4 OS=	21	104.7	100.9	99.9	98.3	102.8	99.1	0.964007782	0.763824
Q92538	GBF1	Golgi-specific brefeldin A-resistance guanine	9	102.8	102.7	99.3	98.8	102.75	99.05	0.963990268	0.763599

O14640	DVL1	Segment polarity protein dishevelled homolog	1	97.4	108	96.3	101.7	102.7	99	0.963972736	0.763373
Q9Y6X3	MAU2	MAU2 chromatid cohesion factor homolog O	3	100.4	104.5	100.3	97.2	102.45	98.75	0.963884822	0.76224
A0A024R4H0	PLOD1	Procollagen-lysine 1, 2-oxoglutarate 5-dioxyg	33	103.1	101.5	99.2	98	102.3	98.6	0.963831867	0.761557
Q01085	TIAL1	Nucleolysin TIAR OS=Homo sapiens GN=TI	7	98.5	106	99.6	97.5	102.25	98.55	0.963814181	0.76133
Q9UPR3	SMG5	Protein SMG5 OS=Homo sapiens GN=SMG5	1	95.3	109	99.1	97.8	102.15	98.45	0.963778757	0.760873
A0A0K0K1K4	HEL-S-276	Proteasome subunit alpha type OS=Homo sap	13	104.2	102.8	101.3	98.2	103.5	99.75	0.963768116	0.760737
Q05DB4	HEBP2	HEBP2 protein (Fragment) OS=Homo sapien	2	93.4	91.2	91.1	86.8	92.3	88.95	0.963705309	0.759928
Q53FE5		Putative uncharacterized protein (Fragment) C	3	104.6	99.2	93.9	102.5	101.9	98.2	0.963689892	0.759729
O75607	NPM3	Nucleoplasmin-3 OS=Homo sapiens GN=NPI	5	101.3	102.4	100.4	95.9	101.85	98.15	0.963672067	0.7595
Q14203	DCTN1	Dynactin subunit 1 OS=Homo sapiens GN=D	18	103.8	102.5	99	99.8	103.15	99.4	0.963645177	0.759154
Q9Y2X9	ZNF281	Zinc finger protein 281 OS=Homo sapiens GN	3	104.7	98.7	94.4	101.6	101.7	98	0.963618486	0.75881
A0A024RDJ1	DC2	DC2 protein, isoform CRA_a OS=Homo sapi	1	106.6	99.5	99.6	99	103.05	99.3	0.963609898	0.7587
D3DUK1	BCAR1	Breast cancer anti-estrogen resistance 1, isofo	1	116.8	94.7	100	103.8	105.75	101.9	0.963593381	0.758487
Q9BV44	THUMPD3	THUMP domain-containing protein 3 OS=Ho	2	99.2	104	99.6	96.2	101.6	97.9	0.963582677	0.75835
A0A024R4Q8	RPS5	Ribosomal protein S5, isoform CRA_a OS=H	11	101.2	101.9	98.6	97.1	101.55	97.85	0.963564746	0.758119
D6REX3	SEC31A	Protein transport protein Sec31A OS=Homo s	14	103.9	99	97.1	98.4	101.45	97.75	0.963528832	0.757657
O96028	WHSC1	Histone-lysine N-methyltransferase NSD2 OS	9	102.7	100.2	97.1	98.4	101.45	97.75	0.963528832	0.757657
J3QR07	YTHDC1	YTH domain-containing protein 1 OS=Homo	8	103.9	101.7	100.4	97.7	102.8	99.05	0.963521401	0.757562
P29084	GTF2E2	Transcription initiation factor IIE subunit beta	5	102.8	102.5	98.9	98.9	102.65	98.9	0.963468095	0.756876
B7Z4T6		Nuclear factor 1 OS=Homo sapiens PE=2 SV	3	107.3	98	99.6	98.2	102.65	98.9	0.963468095	0.756876
Q9Y483	MTF2	Metal-response element-binding transcription	5	101.5	101	93.7	101.4	101.25	97.55	0.96345679	0.756731
Q96RT1	ERBIN	Erbin OS=Homo sapiens GN=ERBIN PE=1 S	7	102.2	100.3	98	97.1	101.25	97.55	0.96345679	0.756731
A0A024QYY5	SPECC1	Spectrin domain with coiled-coils 1, isoform C	6	109.1	101.6	102.7	100.3	105.35	101.5	0.96345515	0.756709
P54819	AK2	Adenylate kinase 2, mitochondrial OS=Homo	12	101.1	98.6	96	96.4	99.85	96.2	0.963445168	0.756581
P42345	MTOR	Serine/threonine-protein kinase mTOR OS=H	7	103.7	103.9	101.5	98.5	103.8	100	0.963391137	0.755886
Q15631	TSN	Translin OS=Homo sapiens GN=TSN PE=1 S	3	100.8	101.2	96.3	98.3	101	97.3	0.963366337	0.755567
Q9BTX1	NDC1	Nucleoporin NDC1 OS=Homo sapiens GN=N	9	109.1	98.3	101.1	98.7	103.7	99.9	0.963355834	0.755432
Q8IVL6	P3H3	Prolyl 3-hydroxylase 3 OS=Homo sapiens GN	9	104.2	100.2	93.8	103.1	102.2	98.45	0.963307241	0.754808
D3DQR0	PACSIN3	Protein kinase C and casein kinase substrate in	5	106.8	102.5	99.5	102.1	104.65	100.8	0.963210702	0.753567
E9PCY5	TOP2B	DNA topoisomerase 2 (Fragment) OS=Homo	1	103.1	103.4	86.9	112	103.25	99.45	0.963196126	0.75338
Q8NFA0	USP32	Ubiquitin carboxyl-terminal hydrolase 32 OS=	2	112.7	80.2	91.8	94	96.45	92.9	0.963193364	0.753344
Q9NU22	MDN1	Midasin OS=Homo sapiens GN=MDN1 PE=1	20	105.3	101.1	97.7	101.1	103.2	99.4	0.963178295	0.753151
P61513	RPL37A	60S ribosomal protein L37a OS=Homo sapien	6	103.9	105.2	101.9	99.5	104.55	100.7	0.963175514	0.753116
O94906	PRPF6	Pre-mRNA-processing factor 6 OS=Homo sap	34	103	100.5	97.9	98.1	101.75	98	0.963144963	0.752723
E7EQR4	EZR	Ezrin OS=Homo sapiens GN=EZR PE=1 SV=	21	101.7	101.8	98.7	97.3	101.75	98	0.963144963	0.752723
Q7Z4H8	KDELC2	KDEL motif-containing protein 2 OS=Homo	15	104.1	101.7	99	99.2	102.9	99.1	0.963070943	0.751772
Q9NYP9	MIS18A	Protein Mis18-alpha OS=Homo sapiens GN=	1	108.3	97.5	94.9	103.3	102.9	99.1	0.963070943	0.751772
Q13325	IFIT5	Interferon-induced protein with tetratricopepti	3	99.9	95	92.4	95.3	97.45	93.85	0.963057978	0.751606
Q8N1F8	STK11IP	Serine/threonine-protein kinase 11-interacting	5	101.4	96	95	95.1	98.7	95.05	0.96301925	0.751109
A0A024R7R0	RAP80	Receptor associated protein 80, isoform CRA_	3	108	97.5	100.2	97.7	102.75	98.95	0.963017032	0.75108
Q9Y6X9	MORC2	MORC family CW-type zinc finger protein 2	6	103.2	102.1	96.9	100.8	102.65	98.85	0.962981003	0.750618
Q5H9R7	PPP6R3	Serine/threonine-protein phosphatase 6 regula	8	101.6	103.7	96.5	101.2	102.65	98.85	0.962981003	0.750618
A4D2D2	PCOLCE	Procollagen C-endopeptidase enhancer OS=H	1	94.3	100	88.4	98.7	97.15	93.55	0.962943901	0.750142
Q8TD43	TRPM4	Transient receptor potential cation channel sul	2	107.3	95	98	96.8	101.15	97.4	0.962926347	0.749916
Q13444	ADAM15	Disintegrin and metalloproteinase domain-con	6	105.1	99.7	97.2	100	102.4	98.6	0.962890625	0.749458
P12036	NEFH	Neurofilament heavy polypeptide OS=Homo	12	103	101.8	96.6	100.6	102.4	98.6	0.962890625	0.749458
A0A024R912	UCK2	Uridine kinase OS=Homo sapiens GN=UCK2	6	101.2	100.5	95.7	98.5	100.85	97.1	0.962816063	0.748502
O15226	NKRF	NF-kappa-B-repressing factor OS=Homo sapi	17	103.2	98.4	99.3	94.8	100.8	97.05	0.962797619	0.748266
A0A0F7KYT8	FXR1	Fragile X mental retardation autosomal homol	21	101.4	105.4	100.5	98.6	103.4	99.55	0.962765957	0.747859
A0A024RAN2	CAST	Calpastatin, isoform CRA_a OS=Homo sapier	8	101.9	102.2	99.6	96.9	102.05	98.25	0.962763351	0.747826
P23634	ATP2B4	Plasma membrane calcium-transporting ATPa	8	102.9	101.1	95.6	100.8	102	98.2	0.962745098	0.747591
E5KMK5		Mitochondrial DNA topoisomerase I OS=Hom	1	112.3	91.5	85.8	110.4	101.9	98.1	0.962708538	0.747123
O95433	AHSA1	Activator of 90 kDa heat shock protein ATPas	13	99.3	99.1	98.5	92.5	99.2	95.5	0.962701613	0.747034
Q59FM4		Scavenger receptor class B member 1 variant	4	99.9	106	99.5	98.7	102.95	99.1	0.962603205	0.745773
A0A024R7V7	CHD7	Chromodomain helicase DNA binding proteir	4	99.4	106	99.2	98.5	102.7	98.85	0.962512171	0.744607
Q9Y3E7	CHMP3	Charged multivesicular body protein 3 OS=H	3	103.5	99.2	98.9	96.2	101.35	97.55	0.962506167	0.74453
Q9NXX6	NSMCE4A	Non-structural maintenance of chromosomes	6	103.3	96.5	96.3	96	99.9	96.15	0.962462462	0.743971
Q5QPA5	MRPS18A	28S ribosomal protein S18a, mitochondrial (F	3	96.9	105.2	96.7	97.8	101.05	97.25	0.962394854	0.743105
Q53HS1		Achalasia, adrenocortical insufficiency, alacri	13	100.3	101.7	95.5	98.9	101	97.2	0.962376238	0.742867
Q9GZT8	NIF3L1	NIF3-like protein 1 OS=Homo sapiens GN=N	5	101.6	105.7	97.6	101.9	103.65	99.75	0.962373372	0.74283
O15164	TRIM24	Transcription intermediary factor 1-alpha OS=	8	106.2	101	101.3	98.1	103.6	99.7	0.962355212	0.742597
P27708	CAD	CAD protein OS=Homo sapiens GN=CAD PI	39	101.9	99.9	95.9	98.3	100.9	97.1	0.962338949	0.742389
Q9P2J9	PDP2	[Pyruvate dehydrogenase [acetyl-transferring]	1	108.4	93.3	94.5	99.6	100.85	97.05	0.962320278	0.74215
Q9NVH2	INTS7	Integrator complex subunit 7 OS=Homo sapie	9	102.9	98.8	96.3	97.8	100.85	97.05	0.962320278	0.74215
Q75N03	CBLL1	E3 ubiquitin-protein ligase Hakai OS=Homo s	5	103.4	98.2	96	98	100.8	97	0.962301587	0.741912
Q96RR1	PEO1	Twinkle protein, mitochondrial OS=Homo sap	2	102.2	101.9	97.9	98.5	102.05	98.2	0.962273395	0.741551
B2R665		cDNA, FLJ92810, highly similar to Homo sap	8	100	101.4	97	96.8	100.7	96.9	0.962264151	0.741433
G3V5N8	ZFYVE1	Zinc finger FYVE domain-containing protein	1	110.2	91	95.4	98.2	100.6	96.8	0.96222664	0.740952
Q9NWV4	C1orf123	UPF0587 protein C1orf123 OS=Homo sapien	2	97.8	103.4	93.3	100.3	100.6	96.8	0.96222664	0.740952
Q9BYC9	MRPL20	39S ribosomal protein L20, mitochondrial OS	5	107.7	101.3	103.8	97.3	104.5	100.55	0.962200957	0.740624
A2VCK8	TMSB4X	Thymosin beta 4, X-linked OS=Homo sapiens	6	86.8	103.5	84.5	98.6	95.15	91.55	0.962165003	0.740164
O15357	INPPL1	Phosphatidylinositol 3,4,5-trisphosphate 5-ph	5	104.7	98.6	94	101.6	101.65	97.8	0.962124939	0.739652
A8K3H8		cDNA FLJ77680, highly similar to Homo sap	15	102.3	100.8	97	98.4	101.55	97.7	0.962087642	0.739175
Q9P0U1	TOMM7	Mitochondrial import receptor subunit TOM7	2	108.1	95	100.7	94.7	101.55	97.7	0.962087642	0.739175
Q549N0	CFL2	Cofilin 2 (Muscle), isoform CRA_a OS=Hom	8	98.9	104.2	98.9	96.5	101.55	97.7	0.962087642	0.739175
Q9UIA9	XPO7	Exportin-7 OS=Homo sapiens GN=XPO7 PE	13	98.6	99.2	95.1	95.2	98.9	95.15	0.962082912	0.739115
A8KA19		cDNA FLJ75831, highly similar to Homo sap	9	99.7	98.1	91.8	98.5	98.9	95.15	0.962082912	0.739115
B3KNC3		cDNA FLJ14222 fis, clone NT2RP3003992, l	15	102.8	100.2	97.9	97.4	101.5	97.65	0.962068966	0.738936
Q96CT7	CCDC124	Coiled-coil domain-containing protein 124 OS	8	102	103.6	101	96.8	102.8	98.9	0.962062257	0.73885
Q5HYK3	COQ5	2-methoxy-6-polyprenyl-1,4-benzoquinol met	3	100.2	105.4	98.4	99.4	102.8	98.9	0.962062257	0.73885
U3KQK5		Uncharacterized protein OS=Homo sapiens PI	1	98.3	102	97.4	95.3	100.15	96.35	0.962056915	0.738783
Q6ICB0	DESI1	Desumoylating isopeptidase 1 OS=Homo sapi	1	103	97.1	89.1	103.4	100.05	96.25	0.962018991	0.738297
Q8WXX5	DNAJC9	DnaJ homolog subfamily C member 9 OS=H	5	105.3	100	93.6	103.9	102.65	98.75	0.962006819	0.738142
P54727	RAD23B	UV excision repair protein RAD23 homolog I	11	104.9	100.4	98.7	98.8	102.65	98.75	0.962006819	0.738142
A0A024R8Y0	CDCA1	Cell division cycle associated 1, isoform CRA	1	102.6	105.3	95.2	104.8	103.95	100	0.962000962	0.738067
Q8WUX2	CHAC2	Putative glutathione-specific gamma-glutamy	1	100	83.8	88.1	88.7	91.9	88.4	0.961915125	0.736971
Q9UK61	FAM208A	Protein TASOR OS=Homo sapiens GN=FAM	10	104.1	100.7	98	99	102.4	98.5	0.961914063	0.736957
E9PQY2	PFDN4	Prefoldin subunit 4 OS=Homo sapiens GN=P	5	101.8	102.9	97.2	99.7	102.35	98.45	0.961895457	0.73672
P56556	NDUFA6	NADH dehydrogenase [ubiquinone] 1 alpha s	5	101.6	102.6	99.7	96.7	102.1	98.2	0.961802155	0.735529
A6NHB5	ZMYM3	Zinc finger MYM-type protein 3 OS=Homo s	17	101.6	99.9	95.5	98.3	100.75	96.9	0.9617866	0.73533
A0A087WWF	POLD2	DNA polymerase delta subunit 2 OS=Homo s	4	103.2	98.3	89.2	104.6	100.75	96.9	0.9617866	0.73533
C9JEJ2	PCYT1A	Choline-phosphate cytidylyltransferase A OS=	6	105.7	103.5	101.3	99.9	104.6	100.6	0.961759082	0.734978
A0A087WYL	MED6	Mediator of RNA polymerase II transcription	1	102.1	96.5	95.1	95.9	99.3	95.5	0.961732125	0.734635
P50570	DNM2	Dynamin-2 OS=Homo sapiens GN=DNM2 PI	14	103.1	100.7	98.6	97.4	101.9	98	0.961727184	0.734571
A5PLM5	EXOSC9	Exosome component 9 OS=Homo sapiens GN	9	103.3	100.4	99.4	96.5	101.85	97.95	0.961708395	0.734331
A0A024R6R2	GPT2	Glutamic pyruvate transaminase (Alanine ami	11	102.4	101.1	96	99.7	101.75	97.85	0.961670762	0.733851
A0A024RCB9	TSSC4	Tumor suppressing subtransferable candidate	1	89.7	103.3	93	92.6	96.5	92.8	0.961658031	0.733689
V9HW29	HEL-S-61	Kinesin-like protein OS=Homo sapiens GN=I	41	102.5	100.6	97.8	97.5	101.55	97.65	0.961595273	0.732889
Q9NSI2	FAM207A	Protein FAM207A OS=Homo sapiens GN=F/	4	104.7	103.6	104.2	96.1	104.15	100.15	0.961593855	0.73287
Q9BZ23	PANK2	Pantothenate kinase 2, mitochondrial OS=Hor	3	103.2	97.2	91.5	101.2	100.2	96.35	0.961576846	0.732653
P62888	RPL30	60S ribosomal protein L30 OS=Homo sapiens	7	103.5	102	100.1	97.5	102.75	98.8	0.961557178	0.732402
Q658Y4	FAM91A1	Protein FAM91A1 OS=Homo sapiens GN=F/	5	110.5	94.7	98.7	98.6	102.6	98.65	0.961500975	0.731686
E9PES4	KIF3A	Kinesin-like protein OS=Homo sapiens GN=I	2	106.7	95.9	94.4	100.4	101.3	97.4	0.961500494	0.731679
Q9NXR1	NDE1	Nuclear distribution protein nudE homolog 1	5	103.1	96.8	95.1	97.1	99.95	96.1	0.96148074	0.731428
B4DM85		Kinesin-like protein OS=Homo sapiens PE=2	7	104.5	100.5	100.7	96.4	102.5	98.55	0.961463415	0.731207
A8K5M4		cDNA FLJ75088, highly similar to Homo sap	10	102	100.2	95.5	98.9	101.1	97.2	0.961424332	0.730709
Q16891	IMMT	MICOS complex subunit MIC60 OS=Homo s	25	103.1	99.1	97.7	96.7	101.1	97.2	0.961424332	0.730709
A5JGZ4	AIP	Non-functional aryl hydrocarbon receptor inte	5	103.3	98.9	98.5	95.9	101.1	97.2	0.961424332	0.730709
B0S7P4		cDNA, FLJ92156, Homo sapiens mitochondri	5	101.5	100.6	97.5	96.8	101.05	97.15	0.961405245	0.730466
A0A024R3X1	LGALS8	Galectin OS=Homo sapiens GN=LGALS8 PE	4	107.1	100	101	98.1	103.55	99.55	0.961371318	0.730033
Q96FS4	SIPA1	Signal-induced proliferation-associated protei	3	107.3	97.2	94.4	102.2	102.25	98.3	0.961369193	0.730007
Q9NTJ3	SMC4	Structural maintenance of chromosomes prote	30	102.4	102	98.4	98.1	102.2	98.25	0.961350294	0.729766
Q9NS93	TM7SF3	Transmembrane 7 superfamily member 3 OS=	1	103.6	100.7	98.3	98.1	102.15	98.2	0.961331375	0.729525
O43414	ERI3	ERI1 exoribonuclease 3 OS=Homo sapiens G	1	93.3	115.9	103.8	97.3	104.6	100.55	0.961281071	0.728884
A0A024QZG0	RNF40	Ring finger protein 40, isoform CRA_a OS=H	16	104.7	101.9	100.3	98.3	103.3	99.3	0.961277832	0.728843
B3KNI0		cDNA FLJ14646 fis, clone NT2RP2001883, l	5	104.5	99.4	99.1	96.9	101.95	98	0.961255517	0.728558
Q7Z2W4	ZC3HAV1	Zinc finger CCCH-type antiviral protein 1 OS	24	103.2	103.2	99.7	98.7	103.2	99.2	0.96124031	0.728365
P18077	RPL35A	60S ribosomal protein L35a OS=Homo sapier	7	102.5	103.9	101.1	97.3	103.2	99.2	0.96124031	0.728365
A0A0G2JMW	RASSF7	Ras association domain-containing protein 7 C	1	100	103.8	96.8	99.1	101.9	97.95	0.961236506	0.728316
Q9P253	VPS18	Vacuolar protein sorting-associated protein 18	7	101	100.2	96.9	96.5	100.6	96.7	0.961232604	0.728267
Q15648	MED1	Mediator of RNA polymerase II transcription	5	109.1	94.5	94.7	101	101.8	97.85	0.961198428	0.727832
B2R7G6		cDNA, FLJ93437, highly similar to Homo sap	8	100.2	103.4	99.7	96	101.8	97.85	0.961198428	0.727832

A0A024RBY9	HCCS	Holocytochrome c synthase (Cytochrome c he	7	103.1	103	100.8	97.3	103.05	99.05	0.961183891	0.727647
Q13158	FADD	FAS-associated death domain protein OS=Ho	2	96.8	93.8	85.6	97.6	95.3	91.6	0.961175236	0.727537
Q49AR2	C5orf22	UPF0489 protein C5orf22 OS=Homo sapiens	1	103.1	97.8	90.9	102.2	100.45	96.55	0.961174714	0.72753
H0YIC4	CS	Citrate synthase (Fragment) OS=Homo sapien	1	111.1	81.8	102.6	82.8	96.45	92.7	0.961119751	0.726831
A0A024QZC1	CD2BP2	CD2 antigen (Cytoplasmic tail) binding protei	8	100.6	97.3	95.3	94.9	98.95	95.1	0.96109146	0.726471
A0A0S2Z319	BAK1	BCL2-antagonist/killer 1 isoform 2 OS=Hom	3	110	95.6	95.8	101.8	102.8	98.8	0.961089494	0.726446
Q8WXF1	PSPC1	Paraspeckle component 1 OS=Homo sapiens	17	102.4	102.9	98.9	98.4	102.65	98.65	0.961032635	0.725722
Q6P4A7	SFXN4	Sideroflexin-4 OS=Homo sapiens GN=SFXN	1	101.2	101.3	95.9	98.7	101.25	97.3	0.960987654	0.72515
Q9ULH0	KIDINS220	Kinase D-interacting substrate of 220 kDa OS	12	104.8	102.8	99.5	100	103.8	99.75	0.960982659	0.725087
A4D0V4	CAPZA2	Capping protein (Actin filament) muscle Z-lin	5	103.5	98.9	97.2	97.3	101.2	97.25	0.960968379	0.724905
Q7Z4V5	HDGFRP2	Hepatoma-derived growth factor-related prote	4	101.2	100.9	98.1	96.1	101.05	97.1	0.96091044	0.724169
Q9BWF3	RBM4	RNA-binding protein 4 OS=Homo sapiens GN	4	100.2	101.8	97.7	96.4	101	97.05	0.960891089	0.723922
O75110	ATP9A	Probable phospholipid-transporting ATPase II	5	110.6	104	103.5	102.7	107.3	103.1	0.960857409	0.723494
K7EJ20	GPX4	Glutathione peroxidase OS=Homo sapiens GN	2	99.1	105	95.2	100.9	102.05	98.05	0.960803528	0.72281
Q03111	MLLT1	Protein ENL OS=Homo sapiens GN=MLLT1	3	100.1	98.8	93.5	97.6	99.45	95.55	0.960784314	0.722566
R4SBI6	EPHX1	EPHX1 OS=Homo sapiens GN=EPHX1 PE=2	16	107	99.4	97.6	100.7	103.2	99.15	0.960755814	0.722204
E5KT65		DNA-directed RNA polymerase subunit RPA	8	103.9	102.4	100	98.2	103.15	99.1	0.960736791	0.721963
Q96BN8	OTULIN	Ubiquitin thioesterase otulin OS=Homo sapie	3	105	98.6	93.8	101.8	101.8	97.8	0.960707269	0.721587
Q9H6A9	PCNX3	Pecanex-like protein 3 OS=Homo sapiens GN	2	88.1	87.4	80.7	87.9	87.75	84.3	0.960683761	0.721289
Q14573	ITPR3	Inositol 1,4,5-trisphosphate receptor type 3 OS	5	104.3	106.8	97.1	105.7	105.55	101.4	0.960682141	0.721268
Q9H4A4	RNPEP	Aminopeptidase B OS=Homo sapiens GN=RN	18	99.4	101.4	96.8	96.1	100.4	96.45	0.960657371	0.720954
A0A140VKG4		Testis tissue sperm-binding protein Li 81P OS	2	107.2	100.8	102.5	97.3	104	99.9	0.960576923	0.719933
Q9Y263	PLAA	Phospholipase A-2-activating protein OS=Ho	16	104.5	100.9	98.2	99.1	102.7	98.65	0.960564752	0.719778
A0A024R324	RHOA	Ras homolog gene family, member A, isoform	3	104.6	103.3	100.8	98.9	103.95	99.85	0.960557961	0.719692
Q5T653	MRPL2	39S ribosomal protein L2, mitochondrial OS=	8	99.4	103.3	99.1	95.6	101.35	97.35	0.960532807	0.719373
Q9H4B0	OSGEPL1	Probable tRNA N6-adenosine threonylcarbam	3	100.7	104.4	99.8	97.2	102.55	98.5	0.96050707	0.719047
O43488	AKR7A2	Aflatoxin B1 aldehyde reductase member 2 O	6	102.1	97.9	95.5	96.6	100	96.05	0.9605	0.718957
Q9Y4K0	LOXL2	Lysyl oxidase homolog 2 OS=Homo sapiens	1	112.3	97.8	100.1	101.7	105.05	100.9	0.960495002	0.718893
V9HW91	HEL-S-8a	Epididymis secretory sperm binding protein L	11	102.5	100	95.8	98.7	101.25	97.25	0.960493827	0.718878
Q9NX76	CMTM6	CKLF-like MARVEL transmembrane domain	1	113.7	98.8	96.5	107.6	106.25	102.05	0.960470588	0.718584
O43795	MYO1B	Unconventional myosin-Ib OS=Homo sapiens	14	104.6	102.8	99.4	99.8	103.7	99.6	0.960462874	0.718486
Q5VTR2	RNF20	E3 ubiquitin-protein ligase BRE1A OS=Hom	14	105.1	99.5	97.6	98.9	102.3	98.25	0.960410557	0.717822
Q15954	HL14	HL14 protein (Fragment) OS=Homo sapiens	1	98.8	105.8	93.6	102.9	102.3	98.25	0.960410557	0.717822
Q9H000	MKRN2	Probable E3 ubiquitin-protein ligase makorin-	4	102.8	99.2	99.7	94.3	101	97	0.96039604	0.717639
Q9UEY8	ADD3	Gamma-adducin OS=Homo sapiens GN=ADD	2	106.8	90.1	98.8	90.3	98.45	94.55	0.960385983	0.717511
Q6NW34	NEPRO	Nucleolus and neural progenitor protein OS=H	8	99.5	102.4	96.6	97.3	100.95	96.95	0.960376424	0.71739
Q6DD87	ZNF787	Zinc finger protein 787 OS=Homo sapiens GN	2	100.2	106.7	97.9	100.8	103.45	99.35	0.960367327	0.717274
Q8WXG6	MADD	MAP kinase-activating death domain protein	1	106.2	95.2	97.6	95.8	100.7	96.7	0.960278054	0.716143
Q14789	GOLGB1	Golgin subfamily B member 1 OS=Homo sap	40	102.2	99.2	96.6	96.8	100.7	96.7	0.960278054	0.716143
A0A024R1X3	VPS25	Vacuolar protein sorting 25 (Yeast), isoform	3	104.8	99.1	96	99.8	101.95	97.9	0.960274644	0.7161
K7EM18	EIF1	Eukaryotic translation initiation factor 1 OS=H	7	103.3	100.6	97.9	97.9	101.95	97.9	0.960274644	0.7161
Q13045	FLII	Protein flightless-1 homolog OS=Homo sapie	11	103.6	102.7	100.8	97.3	103.15	99.05	0.96025206	0.715813
P34896	SHMT1	Serine hydroxymethyltransferase, cytosolic O	4	96.6	97.1	90.4	95.6	96.85	93	0.960247806	0.71576
H7C5E2	BCAP29	B-cell receptor-associated protein 29 (Fragme	3	103.2	100.3	98.5	96.9	101.75	97.7	0.96019656	0.71511
Q5U602	RAB33B	RAB33B, member RAS oncogene family OS=	2	100	98.4	94.3	96.2	99.2	95.25	0.960181452	0.714919
O75970	MPDZ	Multiple PDZ domain protein OS=Homo sapi	24	105	100.7	97.8	99.7	102.85	98.75	0.960136121	0.714345
A8K8N5		cDNA FLJ76940, highly similar to Homo sap	3	100.5	115.2	95.9	111.2	107.85	103.55	0.96012981	0.714265
B2R4A9	FXC1	Fracture callus 1 homolog (Rat) OS=Homo sa	5	101.2	101.9	98.7	96.3	101.55	97.5	0.960118168	0.714118
Q9NPH0	ACP6	Lysophosphatidic acid phosphatase type 6 OS	3	100.3	105.3	96.7	100.7	102.8	98.7	0.960116732	0.7141
O94822	LTN1	E3 ubiquitin-protein ligase listerin OS=Homo	1	104.7	95.8	85.6	106.9	100.25	96.25	0.960099751	0.713885
Q15257	PPP2R4	Serine/threonine-protein phosphatase 2A activ	4	102.7	102.7	97.5	99.7	102.7	98.6	0.960077897	0.713608
Q9Y333	LSM2	U6 snRNA-associated Sm-like protein LSm2	3	102.5	97.8	95	97.3	100.15	96.15	0.96005991	0.71338
Q9UJQ4	SALL4	Sal-like protein 4 OS=Homo sapiens GN=SA	2	100	107.8	98.7	100.8	103.9	99.75	0.960057748	0.713353
D6RER5	SEPT11	Septin-11 OS=Homo sapiens GN=SEPT11 PE	5	108.3	99.5	100.6	98.9	103.9	99.75	0.960057748	0.713353
Q5THK1	PRR14L	Protein PRR14L OS=Homo sapiens GN=PRR	1	110.3	97.2	95.2	104	103.75	99.6	0.96	0.712622
Q7KZI7	MARK2	Serine/threonine-protein kinase MARK2 OS=	2	103.4	91.5	95.2	91.9	97.45	93.55	0.959979477	0.712363
Q9Y3B3	TMED7	Transmembrane emp24 domain-containing pr	8	107.1	100	101.5	97.3	103.55	99.4	0.959922743	0.711645
Q6I9Y2	THOC7	THO complex subunit 7 homolog OS=Homo	4	107.7	99.1	99.7	98.8	103.4	99.25	0.959864603	0.71091
Q969M1	TOMM40L	Mitochondrial import receptor subunit TOM4	3	98.9	102.6	96.6	96.8	100.75	96.7	0.959801489	0.710112
Q6FHU2	PGAM1	Phosphoglycerate mutase (Fragment) OS=Ho	13	98.9	102.3	96	97.1	100.6	96.55	0.959741551	0.709354
P10586	PTPRF	Receptor-type tyrosine-protein phosphatase F	9	106.8	99.3	98.4	99.4	103.05	98.9	0.959728287	0.709187
K7EIG1	CLUH	Clustered mitochondria protein homolog (Fra	26	103.2	102.8	99.5	98.2	103	98.85	0.959708738	0.70894
Q96JC1	VPS39	Vam6/Vps39-like protein OS=Homo sapiens	5	106.5	101.9	103.3	96.7	104.2	100	0.959692898	0.70874
Q9NWU2	GID8	Glucose-induced degradation protein 8 homol	4	107.4	98.5	99.5	98.1	102.95	98.8	0.959689169	0.708692
Q5T4U5	ACADM	Acyl-Coenzyme A dehydrogenase, C-4 to C-1	14	103.7	99.7	99.1	96.1	101.7	97.6	0.959685349	0.708644
Q9HD45	TM9SF3	Transmembrane 9 superfamily member 3 OS=	8	104.1	104.2	100.8	99.1	104.15	99.95	0.959673548	0.708495
U6FSN9	Mprip-Ntrk1	Tyrosine-protein kinase receptor OS=Homo s	10	102.6	103	97.8	99.5	102.8	98.65	0.95963035	0.70795
Q9UBD5	ORC3	Origin recognition complex subunit 3 OS=Ho	12	103.4	99.6	98.4	96.4	101.5	97.4	0.959605911	0.707641
A0A024QYZ9	CEP72	Centrosomal protein 72kDa, isoform CRA_b	1	98.5	94.5	98.8	86.4	96.5	92.6	0.959585492	0.707383
F5H2X7	CIZ1	Cip1-interacting zinc finger protein OS=Hom	1	100	105.3	84.2	112.8	102.65	98.5	0.959571359	0.707205
A0A140VJQ6		Oxysterol-binding protein OS=Homo sapiens	2	107.2	100.3	99.7	99.4	103.75	99.55	0.959518072	0.706532
A8K4P8		cDNA FLJ75337 OS=Homo sapiens PE=2 SV	12	101.7	100.8	97.4	96.9	101.25	97.15	0.959506173	0.706382
Q13510	ASAH1	Acid ceramidase OS=Homo sapiens GN=ASA	9	104.9	105	101.1	100.3	104.95	100.7	0.959504526	0.706362
O75592	MYCBP2	E3 ubiquitin-protein ligase MYCBP2 OS=Ho	1	117.3	87.6	80.7	115.9	102.45	98.3	0.959492435	0.706209
Q99797	MIPEP	Mitochondrial intermediate peptidase OS=Ho	11	101.9	95.5	95.3	94.1	98.7	94.7	0.959473151	0.705965
Q8IWL3	HSCB	Iron-sulfur cluster co-chaperone protein HscB	1	105.9	103.7	101.4	99.7	104.8	100.55	0.959446565	0.70563
A0A0S2Z3G3	SLC25A10	Solute carrier family 25 (Mitochondrial carrie	12	100.1	102.1	97.4	96.6	101.1	97	0.959446093	0.705624
Q53HH3		General transcription factor IIH, polypeptide 4	3	102.9	99.1	95.5	98.3	101	96.9	0.959405941	0.705118
Q9BSK2	SLC25A33	Solute carrier family 25 member 33 OS=Hom	3	106.7	100.1	102.4	96	103.4	99.2	0.959381044	0.704804
Q02083	NAAA	N-acylethanolamine-hydrolyzing acid amidase	3	103.8	95.5	96.1	95.1	99.65	95.6	0.959357752	0.70451
A0A024R6M3	PAPOLA	Poly(A) polymerase alpha, isoform CRA_a O	11	104	102.6	98.1	100.1	103.3	99.1	0.959341723	0.704307
P47897	QARS	Glutamine--tRNA ligase OS=Homo sapiens G	25	101.1	102.9	97.3	98.4	102	97.85	0.959313725	0.703955
Q7Z7F7	MRPL55	39S ribosomal protein L55, mitochondrial OS	4	102.2	104.2	102.2	95.8	103.2	99	0.959302326	0.703811
Q9HD34	LYRM4	LYR motif-containing protein 4 OS=Homo sa	2	99.1	104.4	94	101.2	101.75	97.6	0.959213759	0.702694
P78371	CCT2	T-complex protein 1 subunit beta OS=Homo s	34	103.4	104.7	99.5	100.1	104.05	99.8	0.959154253	0.701945
Q9H6Z4	RANBP3	Ran-binding protein 3 OS=Homo sapiens GN	8	99	104.2	95.7	99.2	101.6	97.45	0.959153543	0.701936
Q9H147	DNTTIP1	Deoxynucleotidyltransferase terminal-interact	6	103.8	101.8	100.2	97	102.8	98.6	0.959143969	0.701816
Q9BW92	TARS2	Threonine--tRNA ligase, mitochondrial OS=H	18	101.9	98.5	96.5	95.7	100.2	96.1	0.959081836	0.701033
Q15223	NECTIN1	Nectin-1 OS=Homo sapiens GN=NECTIN1 P	2	89.9	90.9	83.3	90.1	90.4	86.7	0.959070796	0.700894
Q96EK6	GNPNAT1	Glucosamine 6-phosphate N-acetyltransferase	4	97.3	103	95.5	96.6	100.15	96.05	0.959061408	0.700776
P29692	EEF1D	Elongation factor 1-delta OS=Homo sapiens	5	101.5	106	101.1	97.9	103.75	99.5	0.959036145	0.700458
A0A0S2Z6V5	EFHA1	EF-hand domain family member A1 isoform 1	5	107.2	97.8	99	97.6	102.5	98.3	0.95902439	0.70031
A0A0S2Z4J1	HSD17B4	Hydroxysteroid (17-beta) dehydrogenase 4, is	9	102.9	102	99.6	96.9	102.45	98.25	0.959004392	0.700058
Q14914	PTGR1	Prostaglandin reductase 1 OS=Homo sapiens	10	102.7	99.5	95.1	98.8	101.1	96.95	0.958951533	0.699393
Q5W009	RBM17	RNA binding motif protein 17, isoform CRA_	16	105.2	99.4	98.5	97.7	102.3	98.1	0.958944282	0.699302
B3KMZ5		cDNA FLJ13047 fis, clone NT2RP3001383,	1	100	102.1	94.5	99.3	101.05	96.9	0.958931222	0.699137
A0A024RDB4	HNRPD	Heterogeneous nuclear ribonucleoprotein D (	1	94	105.3	94.1	97	99.65	95.55	0.958855996	0.698191
P33552	CKS2	Cyclin-dependent kinases regulatory subunit 2	2	98.9	97.7	90.2	98.3	98.3	94.25	0.958799593	0.697482
O60637	TSPAN3	Tetraspanin-3 OS=Homo sapiens GN=TSPAN	2	112.1	98.8	102.4	99.8	105.45	101.1	0.958748222	0.696837
Q5TA45	CPSF3L	Integrator complex subunit 11 OS=Homo sap	3	104.1	99.5	92.1	103.1	101.8	97.6	0.958742633	0.696766
H0YIV9		Uncharacterized protein (Fragment) OS=Hom	5	104.3	99	95.1	99.8	101.65	97.45	0.958681751	0.696001
Q92797	SYMPK	Symplekin OS=Homo sapiens GN=SYMPK	17	106.3	99.2	98.1	98.9	102.75	98.5	0.95863747	0.695445
Q9NR33	POLE4	DNA polymerase epsilon subunit 4 OS=Hom	2	104.2	98.8	97.4	97.2	101.5	97.3	0.95862069	0.695234
Q3KQU3	MAP7D1	MAP7 domain-containing protein 1 OS=Hom	4	106.6	103.6	104.5	97	105.1	100.75	0.958610847	0.695111
P49770	EIF2B2	Translation initiation factor eIF-2B subunit be	7	99.9	100.5	94.2	97.9	100.2	96.05	0.958582834	0.694759
Q9C037	TRIM4	E3 ubiquitin-protein ligase TRIM4 OS=Hom	1	97.2	112.6	108.7	92.4	104.9	100.55	0.958531935	0.69412
A0A087WXL6	VPS11	Vacuolar protein sorting 11 (Yeast), isoform	9	102.9	101.8	95.8	100.4	102.35	98.1	0.958475818	0.693416
P53041	PPP5C	Serine/threonine-protein phosphatase 5 OS=H	9	101.9	100.3	95.4	98.4	101.1	96.9	0.958456973	0.69318
A0A0S2Z5U7	DIABLO	Diablo-like protein isoform 1 (Fragment) OS=	8	104	103	98.5	99.9	103.5	99.2	0.958454106	0.693144
Q9UJY5	GGA1	ADP-ribosylation factor-binding protein GGA	2	104.8	102	100.9	97.3	103.4	99.1	0.958413926	0.692639
Q9ULW3	ABT1	Activator of basal transcription 1 OS=Homo s	3	101.8	97.6	96.1	95	99.7	95.55	0.958375125	0.692152
Q96AY3	FKBP10	Peptidyl-prolyl cis-trans isomerase FKBP10	19	103.5	100.7	98.6	97.1	102.1	97.85	0.958374143	0.69214
Q9NVX2	NLE1	Notchless protein homolog 1 OS=Homo sapie	9	101.1	100.6	96.4	96.9	100.85	96.65	0.958353991	0.691888
Q8WUH2	TGFBRAP1	Transforming growth factor-beta receptor-ass	1	100.4	101.3	103.8	89.5	100.85	96.65	0.958353991	0.691888
Q9NYB0	TERF2IP	Telomeric repeat-binding factor 2-interacting	3	102.9	101.1	95.5	100	102	97.75	0.958333333	0.691628
Q9NRW1	RAB6B	Ras-related protein Rab-6B OS=Homo sapien	2	106	95.6	94.5	98.7	100.8	96.6	0.958333333	0.691628
Q9H3H3	C11orf68	UPF0696 protein C11orf68 OS=Homo sapien	1	101.4	109.7	94.9	107.4	105.55	101.15	0.958313595	0.691381
Q6PJG6	BRAT1	BRCA1-associated ATM activator 1 OS=Hom	4	104.8	98.7	98.8	96.2	101.75	97.5	0.958230958	0.690345
O95721	SNAP29	Synaptosomal-associated protein 29 OS=Hom	3	100.6	95.5	92.9	95	98.05	93.95	0.9581846	0.689764
Q96IV0	NGLY1	Peptide-N(4)-(N-acetyl-beta-glucosaminyl)asp	4	101.8	101.4	95.5	99.2	101.6	97.35	0.958169291	0.689573
Q8NE71	ABCF1	ATP-binding cassette sub-family F member 1	17	104.8	98.3	96.3	98.3	101.55	97.3	0.958148695	0.689315
Q15742	NAB2	NGFI-A-binding protein 2 OS=Homo sapiens	1	97.5	112.4	106.7	94.4	104.95	100.55	0.958075274	0.688395

P45973	CBX5	Chromobox protein homolog 5 OS=Homo sap	10	102.4	102.5	99.4	96.9	102.45	98.15	0.958028306	0.687807
Q96C86	DCPS	m7GpppX diphosphatase OS=Homo sapiens (	3	104	98.5	87.9	106.1	101.25	97	0.958024691	0.687762
Q96F46	IL17RA	Interleukin-17 receptor A OS=Homo sapiens (	1	105.6	99.1	96.7	99.4	102.35	98.05	0.957987298	0.687294
A8K6X9		cDNA FLJ76427, highly similar to Homo sap	21	103.6	103.2	101.2	96.9	103.4	99.05	0.957930368	0.686582
Q8WWH5	TRUB1	Probable tRNA pseudouridine synthase 1 OS=	3	103	101.3	93.8	101.9	102.15	97.85	0.957905042	0.686265
A0A024R5Q1	hCG_2003792	HCG2003792, isoform CRA_b OS=Homo sap	6	101.4	98	93.3	97.7	99.7	95.5	0.957873621	0.685872
A0A024R9G0	POLR2K	Polymerase (RNA) II (DNA directed) polypep	1	98	108.4	106.2	91.5	103.2	98.85	0.957848837	0.685562
A0A024R829	POLE3	Polymerase (DNA directed), epsilon 3 (P17 su	5	104	100	98.1	97.3	102	97.7	0.957843137	0.685491
Q9BQ39	DDX50	ATP-dependent RNA helicase DDX50 OS=H	16	102.6	101.3	98.3	97	101.95	97.65	0.957822462	0.685232
E5DZN4	COX2	Cytochrome c oxidase subunit 2 OS=Homo sa	1	122.4	86.2	97	102.8	104.3	99.9	0.957813998	0.685126
P52701	MSH6	DNA mismatch repair protein Msh6 OS=Hom	28	104.8	101.2	99	98.3	103	98.65	0.95776699	0.684539
Q00403	GTF2B	Transcription initiation factor IIB OS=Homo s	7	102.4	103.5	97.6	99.6	102.95	98.6	0.957746479	0.684283
Q5VV42	CDKAL1	Threonylcarbamoyladenosine tRNA methylth	6	108.9	101.1	101	100.1	105	100.55	0.957619048	0.682691
Q8WUK0	PTPMT1	Phosphatidylglycerophosphatase and protein-t	4	101	101.7	95.7	98.4	101.35	97.05	0.957572768	0.682113
Q9BPX5	ARPC5L	Actin-related protein 2/3 complex subunit 5-li	5	102.6	102.3	96.8	99.4	102.45	98.1	0.957540264	0.681708
Q8TEP9	FLJ00144	FLJ00144 protein (Fragment) OS=Homo sapi	1	111.8	95.4	96.1	102.3	103.6	99.2	0.957528958	0.681566
P51116	FXR2	Fragile X mental retardation syndrome-relatec	13	107.1	102.2	99.5	100.9	104.65	100.2	0.957477305	0.680922
V9HWJ1	HEL-S-64p	Glutathione synthetase OS=Homo sapiens GN	14	101.6	100.4	96.1	97.3	101	96.7	0.957425743	0.680278
Q9BTA9	WAC	WW domain-containing adapter protein with c	1	96	101.3	94.4	94.5	98.65	94.45	0.957425241	0.680272
Q9H0C8	ILKAP	Integrin-linked kinase-associated serine/threo	5	94.4	102.9	95.4	93.5	98.65	94.45	0.957425241	0.680272
A0A140VJZ1		Testicular tissue protein Li 218 OS=Homo saj	18	100.2	99.3	95.7	95.3	99.75	95.5	0.957393484	0.679876
A8MTY0	ZNF724	Zinc finger protein 724 OS=Homo sapiens GN	1	98	106.1	92.1	103.3	102.05	97.7	0.957373836	0.679632
A0A024R9Y7	MAGED2	Melanoma antigen family D, 2, isoform CRA_	7	103.9	97.8	95.7	97.4	100.85	96.55	0.957362419	0.679489
A0A0C4DFX9	NELFA	Negative elongation factor A OS=Homo sapie	6	110	98.4	100.5	99	104.2	99.75	0.957293666	0.678632
P17275	JUNB	Transcription factor jun-B OS=Homo sapiens	1	106.4	104.3	100.6	101.1	105.35	100.85	0.95728524	0.678527
Q86W50	METTL16	Methyltransferase-like protein 16 OS=Homo s	2	97	101.9	92.2	98.2	99.45	95.2	0.957264957	0.678274
P08047	SP1	Transcription factor Sp1 OS=Homo sapiens G	4	108.6	104.2	103.9	99.8	106.4	101.85	0.957236842	0.677924
Q9NT62	ATG3	Ubiquitin-like-conjugating enzyme ATG3 OS	3	96.8	101.9	94.3	95.9	99.35	95.1	0.957221943	0.677738
Q15738	NSDHL	Sterol-4-alpha-carboxylate 3-dehydrogenase,	13	104.9	100.7	99.4	97.4	102.8	98.4	0.957198444	0.677446
A0A140VJR2		Testicular tissue protein Li 138 OS=Homo saj	3	97.4	119.6	122.1	85.6	108.5	103.85	0.957142857	0.676753
Q92604	LPGAT1	Acyl-CoA:lysophosphatidylglycerol acyltrans	5	103.7	96.9	97.9	94.1	100.3	96	0.957128614	0.676576
P21399	ACO1	Cytoplasmic aconitate hydratase OS=Homo sa	6	100.8	102.1	98.6	95.6	101.45	97.1	0.957121735	0.676491
Q59EL2		COP9 constitutive photomorphogenic homolo	13	102.7	100.1	97.6	96.5	101.4	97.05	0.957100592	0.676227
Q02543	RPL18A	60S ribosomal protein L18a OS=Homo sapier	9	104.3	102.9	100.9	97.4	103.6	99.15	0.957046332	0.675552
Q9BPW8	NIPSNAP1	Protein NipSnap homolog 1 OS=Homo sapier	10	101.5	100.8	96.6	97	101.15	96.8	0.956994563	0.674908
Q96KC8	DNAJC1	DnaJ homolog subfamily C member 1 OS=Hc	8	102	104.8	100	97.9	103.4	98.95	0.95696325	0.674518
Q92541	RTF1	RNA polymerase-associated protein RTF1 ho	9	101.3	100.8	96.5	96.9	101.05	96.7	0.956952004	0.674378
Q541A5	UFD1L	Ubiquitin fusion degradation 1 like (Yeast), is	12	102.8	101.6	98	97.6	102.2	97.8	0.956947162	0.674318
Q9GZX9	TWSG1	Twisted gastrulation protein homolog 1 OS=F	1	103.8	102.3	95.5	101.7	103.05	98.6	0.956817079	0.672702
A0A024QZB9	ZNF553	Zinc finger protein 553, isoform CRA_a OS=	1	107.9	98.1	94.6	102.5	103	98.55	0.956796117	0.672441
O60830	TIMM17B	Mitochondrial import inner membrane translo	3	108.7	97.2	95.3	101.7	102.95	98.5	0.956775134	0.67218
Q8N3D4	EHBP1L1	EH domain-binding protein 1-like protein 1 O	11	101.3	104.5	96.8	100.1	102.9	98.45	0.95675413	0.67192
Q8TF68	ZNF384	Zinc finger protein 384 OS=Homo sapiens GN	5	100	101.1	94	98.4	100.55	96.2	0.956737941	0.671718
A0A0G2JNW8	MICB	MHC class I polypeptide-related sequence B (	5	104.8	103.2	89.3	109.7	104	99.5	0.956730769	0.671629
Q53EY9		F-box only protein 22 isoform a variant (Fragi	5	98.1	100.4	91.7	98.2	99.25	94.95	0.956675063	0.670937
Q9NSI6	BRWD1	Bromodomain and WD repeat-containing prot	1	89.1	90.9	88.1	84.1	90	86.1	0.956666667	0.670833
P49915	GMPS	GMP synthase [glutamine-hydrolyzing] OS=I	28	102.8	100.2	97	97.2	101.5	97.1	0.956650246	0.67063
Q8NB37	PDDC1	Parkinson disease 7 domain-containing protei	1	101.8	105.7	95.9	102.6	103.75	99.25	0.956626506	0.670335
Q5T1Z8	PUM1	Pumilio homolog 1 OS=Homo sapiens GN=P	5	104.6	107.5	112.8	90.1	106.05	101.45	0.956624234	0.670306
A0A0M3HER	CENPV	Centromere protein V OS=Homo sapiens GN	1	104.6	95.9	97.1	94.7	100.25	95.9	0.956608479	0.670111
Q9H814	PHAX	Phosphorylated adapter RNA export protein C	3	105.3	102.1	93.7	104.7	103.7	99.2	0.956605593	0.670075
D3DR24	EXT2	Exostoses (Multiple) 2, isoform CRA_a OS=I	3	102.1	102.2	95.3	100.1	102.15	97.7	0.956436613	0.66798
Q96NT0	CCDC115	Coiled-coil domain-containing protein 115 OS	2	105.1	103.6	100.7	98.9	104.35	99.8	0.956396742	0.667485
A0A024R374	CTSB	Cathepsin B, isoform CRA_a OS=Homo sapi	6	104.6	101.6	98.2	99	103.1	98.6	0.956353055	0.666944
A8K548		cDNA FLJ75008, highly similar to Homo sap	10	103.1	100.7	97.9	97	101.9	97.45	0.956329735	0.666655
A0A0B4J1V9	HELLS	Helicase, lymphoid-specific, isoform CRA_b	18	102.2	101.6	97.4	97.5	101.9	97.45	0.956329735	0.666655
Q13011	ECH1	Delta(3,5)-Delta(2,4)-dienoyl-CoA isomerase	14	101	100.5	96.5	96.2	100.75	96.35	0.956327543	0.666628
A6NEM2	HCFC1	Host cell factor 1 OS=Homo sapiens GN=HC	1	103.7	104.6	98.3	100.9	104.15	99.6	0.95631301	0.666448
Q9BVS4	RIOK2	Serine/threonine-protein kinase RIO2 OS=Ho	2	97.9	107.9	92.3	104.5	102.9	98.4	0.956268222	0.665893
H0Y2W2	ATAD3A	ATPase family AAA domain-containing prote	6	104.8	103.2	101	97.9	104	99.45	0.95625	0.665667
A0A140VJY2		Testicular tissue protein Li 209 OS=Homo saj	4	102	99.1	96.4	95.9	100.55	96.15	0.956240676	0.665551
Q9H4L7	SMARCAD1	SWI/SNF-related matrix-associated actin-depe	11	103.6	101.8	96.4	100	102.7	98.2	0.956183057	0.664839
P08574	CYC1	Cytochrome c1, heme protein, mitochondrial (	10	105.3	99.9	98.9	97.3	102.6	98.1	0.956140351	0.66431
Q9UQR0	SCML2	Sex comb on midleg-like protein 2 OS=Homo	9	101.8	100.9	100	93.8	101.35	96.9	0.956092748	0.663721
A8K3X3		cDNA FLJ77685 OS=Homo sapiens PE=2 SV	1	103	92.7	95	92.1	97.85	93.55	0.956055187	0.663256
A0A087WWE	POLR2A	DNA-directed RNA polymerase subunit OS=I	38	102.9	101.6	97.4	98.1	102.25	97.75	0.95599022	0.662453
B4E3G8		cDNA FLJ61576, highly similar to Mitochonc	2	106.4	95.8	94.9	98.4	101.1	96.65	0.955984174	0.662378
P35052	GPC1	Glypican-1 OS=Homo sapiens GN=GPC1 PE	8	103.6	100.6	97	98.2	102.1	97.6	0.955925563	0.661654
P04075	ALDOA	Fructose-bisphosphate aldolase A OS=Homo	23	99.7	102.2	96.7	96.3	100.95	96.5	0.955918772	0.66157
B2R9C5		cDNA, FLJ94330 OS=Homo sapiens PE=2 S	2	102.5	99.4	97.2	95.8	100.95	96.5	0.955918772	0.66157
Q7Z351	DKFZp686N02	Putative uncharacterized protein DKFZp686N	1	95.5	90.5	83.8	94	93	88.9	0.955913978	0.661511
P36551	CPOX	Oxygen-dependent coproporphyrinogen-III ox	14	103.9	100.2	98.2	96.9	102.05	97.55	0.955903969	0.661387
Q9NZB2	FAM120A	Constitutive coactivator of PPAR-gamma-like	15	105.2	101.1	99.3	97.9	103.15	98.6	0.955889481	0.661208
A0A024R1F4	hCG_2010666	HCG2010666, isoform CRA_a OS=Homo sap	1	106	98	94.9	100.1	102	97.5	0.955882353	0.66112
Q02952	AKAP12	A-kinase anchor protein 12 OS=Homo sapien	5	111.2	104.1	102.1	103.7	107.65	102.9	0.955875523	0.661036
P00558	PGK1	Phosphoglycerate kinase 1 OS=Homo sapiens	24	101.4	100.3	96.5	96.3	100.85	96.4	0.955875062	0.66103
A0A090N8P3	FKBP9	FK506 binding protein 9, 63 kDa OS=Homo s	13	102.9	100.9	98.3	96.5	101.9	97.4	0.955839058	0.660585
A0A087WSV8	NUCB2	Nucleobindin 2, isoform CRA_b OS=Homo s	16	105.1	100.9	99.7	97.2	103	98.45	0.955825243	0.660415
Q8N5W9	FAM101B	Filamin-interacting protein FAM101B OS=Ho	1	106.1	101.8	100.2	98.5	103.95	99.35	0.955747956	0.659461
Q7Z4Q2	HEATR3	HEAT repeat-containing protein 3 OS=Homo	1	97.2	110.6	98.5	100.1	103.9	99.3	0.95572666	0.659198
P35606	COPB2	Coatomer subunit beta' OS=Homo sapiens GN	32	102.8	102.5	98.2	98	102.65	98.1	0.955674623	0.658556
Q6NW29	RWDD4	RWD domain-containing protein 4 OS=Homo	3	106.5	102.9	104.8	95.3	104.7	100.05	0.955587393	0.65748
Q92610	ZNF592	Zinc finger protein 592 OS=Homo sapiens GN	3	99.8	102.8	97.2	96.4	101.3	96.8	0.955577493	0.657358
P07919	UQCRH	Cytochrome b-c1 complex subunit 6, mitocho	6	101.8	100.6	97.4	96	101.2	96.7	0.955533597	0.656817
J3QQ67	RPL18	60S ribosomal protein L18 (Fragment) OS=H	6	103.6	105.3	103.5	96.1	104.45	99.8	0.955481091	0.65617
Q96ER9	CCDC51	Coiled-coil domain-containing protein 51 OS:	8	104.9	101.7	99.9	97.5	103.3	98.7	0.955469506	0.656027
A0A024RDQ8	RFC3	Replication factor C (Activator 1) 3, 38kDa, is	9	103.2	101.1	97.5	97.7	102.15	97.6	0.95545766	0.655881
O15198	SMAD9	Mothers against decapentaplegic homolog 9 C	1	95.9	103.3	96.2	94.1	99.6	95.15	0.955321285	0.654202
Q9UHR4	BAIAP2L1	Brain-specific angiogenesis inhibitor 1-associ	5	105.6	97.8	97.1	97.2	101.7	97.15	0.95526057	0.653455
P48960	CD97	CD97 antigen OS=Homo sapiens GN=CD97	9	103.6	99.6	97.3	96.8	101.6	97.05	0.955216535	0.652913
H0Y322	LIN9	Protein lin-9 homolog (Fragment) OS=Homo	1	105.8	104	100.1	100.3	104.9	100.2	0.955195424	0.652653
A0A024QZT4	SERPINB9	Serpin peptidase inhibitor, clade B (Ovalbumi	9	105.4	99.7	99	96.9	102.55	97.95	0.955143832	0.652019
Q4LE48	STAG1	STAG1 variant protein (Fragment) OS=Homo	6	96.7	97.1	89.7	95.4	96.9	92.55	0.955108359	0.651583
Q08379	GOLGA2	Golgin subfamily A member 2 OS=Homo sap	21	103.6	101.3	95.7	100	102.45	97.85	0.955100049	0.651481
P12268	IMPDH2	Inosine-5'-monophosphate dehydrogenase 2 C	19	101	103.9	97.9	97.8	102.45	97.85	0.955100049	0.651481
E7EQZ4	SMN1	Survival motor neuron protein OS=Homo sap	4	106	98.9	99.4	96.3	102.45	97.85	0.955100049	0.651481
Q9UKK6	NXT1	NTF2-related export protein 1 OS=Homo sap	2	102.5	104.6	101.5	96.3	103.55	98.9	0.955094157	0.651408
Q14997	PSME4	Proteasome activator complex subunit 4 OS=I	6	105.7	103.6	101.3	98.6	104.65	99.95	0.95508839	0.651337
Q9NUU7	DDX19A	ATP-dependent RNA helicase DDX19A OS=	15	99.6	105.2	97.9	97.7	102.4	97.8	0.955078125	0.651211
J9R021	eIF3a	Eukaryotic translation initiation factor 3 subu	57	104.5	104.1	100.9	98.3	104.3	99.6	0.95493768	0.649486
B2R5W6		cDNA, FLJ92661, highly similar to Homo saj	3	93.2	112.7	102.6	94	102.95	98.3	0.954832443	0.648195
Q7Z333	SETX	Probable helicase senataxin OS=Homo sapien	4	113	99.3	99.8	102.9	106.15	101.35	0.95478097	0.647564
O00233	PSMD9	26S proteasome non-ATPase regulatory subu	7	101.1	102.3	97.1	97.1	101.7	97.1	0.954768928	0.647416
Q6PKG0	LARP1	La-related protein 1 OS=Homo sapiens GN=L	20	106.7	103	103.4	96.8	104.85	100.1	0.954697186	0.646536
Q13885	TUBB2A	Tubulin beta-2A chain OS=Homo sapiens GN	3	110.8	98.8	102.1	98	104.8	100.05	0.954675573	0.646271
P49321	NASP	Nuclear autoantigenic sperm protein OS=Hom	23	101.9	103.2	96.4	99.4	102.55	97.9	0.954656265	0.646034
Q96A35	MRPL24	39S ribosomal protein L24, mitochondrial OS	10	102	100.7	96.7	96.8	101.35	96.75	0.954612728	0.645501
Q9H8Y8	GORASP2	Golgi reassembly-stacking protein 2 OS=Hom	4	96.7	92.7	92.7	88.1	94.7	90.4	0.954593453	0.645265
F1B6A5	ND5	NADH-ubiquinone oxidoreductase chain 5 OS	1	109.3	92.8	97.9	95	101.05	96.45	0.954477981	0.643851
Q96GM8	TOE1	Target of EGR1 protein 1 OS=Homo sapiens	8	99.4	107	97.9	99.1	103.2	98.5	0.954457364	0.643599
O43242	PSMD3	26S proteasome non-ATPase regulatory subu	21	104.3	104.2	101.1	97.9	104.25	99.5	0.954436451	0.643343
Q6ZXV5	TMTC3	Transmembrane and TPR repeat-containing p	2	107.7	96.4	103	91.8	102.05	97.4	0.954434101	0.643314
B4E290		cDNA FLJ50039, highly similar to Homo sap	11	102.1	97.6	95.7	94.9	99.85	95.3	0.954431647	0.643284
Q8WV93	LACE1	Lactation elevated protein 1 OS=Homo sapier	1	93.6	99.4	91.3	92.9	96.5	92.1	0.954404145	0.642948
Q9P086	MED11	Mediator of RNA polymerase II transcription	1	95.8	90.6	91.7	86.2	93.2	88.95	0.954399142	0.642887
P19623	SRM	Spermidine synthase OS=Homo sapiens GN=	8	100.7	101	95.5	97	100.85	96.25	0.954387705	0.642747
Q9Y6K1	DNMT3A	DNA (cytosine-5)-methyltransferase 3A OS=	2	105	101	93.3	103.3	103	98.3	0.954368932	0.642517
Q16352	INA	Alpha-internexin OS=Homo sapiens GN=INA	33	101.8	101.9	97.9	96.5	101.85	97.2	0.954344624	0.64222
Q9Y2U8	LEMD3	Inner nuclear membrane protein Man1 OS=Hc	21	101.2	102.5	97.8	96.6	101.85	97.2	0.954344624	0.64222
A0A087WXK	FAM83D	Chromosome 20 open reading frame 129 OS=	2	100.8	105	97	99.4	102.9	98.2	0.954324587	0.641975
Q96AT9	RPE	Ribulose-phosphate 3-epimerase OS=Homo s	2	100	101.3	97.1	95	100.65	96.05	0.954297069	0.641638
B4DEQ7		cDNA FLJ50003, highly similar to Homo sap	1	98.9	100.2	99	91	99.55	95	0.954294324	0.641605

Q7Z4S6	KIF21A	Kinesin-like protein KIF21A OS=Homo sapie	6	105.6	97.8	99.3	94.8	101.7	97.05	0.954277286	0.641397
A0A0D9SFG6	CYTH2	Cytohesin-2 OS=Homo sapiens GN=CYTH2	1	107.3	95.8	93.6	100.2	101.55	96.9	0.954209749	0.640571
Q9NSD9	FARSB	Phenylalanine--tRNA ligase beta subunit OS=	17	103.6	103.8	98.4	99.5	103.7	98.95	0.954194793	0.640389
Q9Y490	TLN1	Talin-1 OS=Homo sapiens GN=TLN1 PE=1 S	32	103.2	97.6	94.1	97.5	100.4	95.8	0.954183267	0.640248
Q9BV68	RNF126	E3 ubiquitin-protein ligase RNF126 OS=Hom	1	101	106.3	96.7	101.1	103.65	98.9	0.954172697	0.640119
Q86T24	ZBTB33	Transcriptional regulator Kaiso OS=Homo sap	2	100.6	104.3	93.3	102.2	102.45	97.75	0.954123963	0.639524
P08240	SRPRA	Signal recognition particle receptor subunit al	17	103.2	101.6	97.6	97.8	102.4	97.7	0.954101563	0.63925
P27695	APEX1	DNA-(apurinic or apyrimidinic site) lyase OS	11	98.6	99.6	94.5	94.6	99.1	94.55	0.954086781	0.63907
A5PL36	USP19	USP19 protein (Fragment) OS=Homo sapiens	3	111.8	101.6	102.9	100.7	106.7	101.8	0.954076851	0.638948
Q68EM7	ARHGAP17	Rho GTPase-activating protein 17 OS=Homo	4	105.1	96.6	96	96.4	100.85	96.2	0.953891919	0.636693
A0A0S2Z507	BTRC	Beta-transducin repeat containing isoform 3 (I	2	100.7	98.5	87.7	102.3	99.6	95	0.953815261	0.635759
Q9BVK8	TMEM147	Transmembrane protein 147 OS=Homo sapier	1	114.6	95.2	102.1	98	104.9	100.05	0.953765491	0.635153
O60869	EDF1	Endothelial differentiation-related factor 1 OS	6	104.8	102.6	99.1	98.7	103.7	98.9	0.953712633	0.634509
Q18PE1	DOK7	Protein Dok-7 OS=Homo sapiens GN=DOK7	1	106	101.1	103.1	94.4	103.55	98.75	0.953645582	0.633693
Q8NBK3	SUMF1	Sulfatase-modifying factor 1 OS=Homo sapie	2	106.5	100.5	96.3	101.1	103.5	98.7	0.953623188	0.63342
A0A140VK83	PPP1R7	Protein phosphatase 1, regulatory subunit 7, is	6	102.6	100	95	98.2	101.3	96.6	0.953603159	0.633177
Q96AB3	ISOC2	Isochorismatase domain-containing protein 2	3	100.9	97.3	94.1	94.9	99.1	94.5	0.95358224	0.632922
Q8N6T7	SIRT6	NAD-dependent protein deacetylase sirtuin-6	5	100.7	103.7	95.9	99	102.2	97.45	0.953522505	0.632196
Q96AA8	JAKMIP2	Janus kinase and microtubule-interacting prot	1	110	90	80	110.7	100	95.35	0.9535	0.631922
H0Y9Z5	CNOT6L	CCR4-NOT transcription complex subunit 6-l	2	98.9	101.1	92.5	98.2	100	95.35	0.9535	0.631922
Q14151	SAFB2	Scaffold attachment factor B2 OS=Homo sapi	17	107.7	98.7	96.2	100.6	103.2	98.4	0.953488372	0.631781
Q9HBI1	PARVB	Beta-parvin OS=Homo sapiens GN=PARVB	1	102.2	102	96.1	98.6	102.1	97.35	0.953476983	0.631643
Q9BV20	MRI1	Methylthioribose-1-phosphate isomerase OS=	4	108.7	97.2	97.1	99.2	102.95	98.15	0.953375425	0.630409
P28799	GRN	Granulins OS=Homo sapiens GN=GRN PE=1	3	103.4	100.3	91.8	102.4	101.85	97.1	0.953362788	0.630255
A0A0A0MSI8	EXOC5	Exocyst complex component 5 OS=Homo sap	6	105.5	100	94.5	101.4	102.75	97.95	0.953284672	0.629307
Q9HBD4	SMARCA4	SMARCA4 isoform 2 OS=Homo sapiens GN	18	103.8	101.6	98.2	97.6	102.7	97.9	0.953261928	0.629031
Q9Y6M7	SLC4A7	Sodium bicarbonate cotransporter 3 OS=Hom	1	108.1	105.3	89.2	114.2	106.7	101.7	0.953139644	0.627548
Q4V339	CBWD6	COBW domain-containing protein 6 OS=Hom	1	101.9	96.5	89.7	99.4	99.2	94.55	0.953125	0.62737
Q14807	KIF22	Kinesin-like protein KIF22 OS=Homo sapien	11	100.6	104.2	99.5	95.7	102.4	97.6	0.953125	0.62737
P35269	GTF2F1	General transcription factor IIF subunit 1 OS=	6	104.9	99.8	95.1	100	102.35	97.55	0.953102101	0.627093
K7EL20	EIF3G	Eukaryotic translation initiation factor 3 subu	1	82.9	85.5	91.9	68.6	84.2	80.25	0.953087886	0.62692
Q9ULR0	ISY1	Pre-mRNA-splicing factor ISY1 homolog OS	4	105.1	97.4	93.7	99.3	101.25	96.5	0.95308642	0.626903
P54105	CLNS1A	Methylosome subunit pICln OS=Homo sapier	5	99.8	102.6	97.3	95.6	101.2	96.45	0.953063241	0.626622
B2RNR6	ZFR	Zinc finger RNA binding protein OS=Homo s	26	104.1	102.3	98.1	98.6	103.2	98.35	0.953003876	0.625903
A8K6T5		cDNA FLJ75144, highly similar to Homo sap	10	103.3	103.1	98	98.7	103.2	98.35	0.953003876	0.625903
D3DR37	CEP55	Centrosomal protein 55kDa, isoform CRA_a O	4	108.4	100.1	101.4	97.3	104.25	99.35	0.952997602	0.625827
A0A1C7CYX9	DPYSL2	Dihydropyrimidinase-related protein 2 OS=H	4	100.3	99.6	96.8	93.7	99.95	95.25	0.952976488	0.625571
Q16643	DBN1	Drebrin OS=Homo sapiens GN=DBN1 PE=1	26	107.4	103.1	100.9	99.7	105.25	100.3	0.952969121	0.625482
H3BQJ5	NOL3	Nucleolar protein 3 (Fragment) OS=Homo sap	1	110.3	98	95.7	102.8	104.15	99.25	0.952952472	0.62528
J3KNR0	MARK3	Non-specific serine/threonine protein kinase (	2	99.7	104.2	100.3	94	101.95	97.15	0.952918097	0.624864
A8K333		cDNA FLJ75639, highly similar to Homo sap	2	102.3	97.2	94.9	95.2	99.75	95.05	0.952882206	0.62443
P42858	HTT	Huntingtin OS=Homo sapiens GN=HTT PE=	4	97.4	102.1	91.1	99	99.75	95.05	0.952882206	0.62443
Q9Y3X0	CCDC9	Coiled-coil domain-containing protein 9 OS=	1	106.4	105.8	102.1	100.1	106.1	101.1	0.952874647	0.624338
Q6P2H3	CEP85	Centrosomal protein of 85 kDa OS=Homo sap	1	109	101	97.1	103	105	100.05	0.952857143	0.624126
O75821	EIF3G	Eukaryotic translation initiation factor 3 subu	1	110.5	101.4	97.1	104.8	105.95	100.95	0.952807928	0.623531
B3KUZ7		cDNA FLJ40986 fis, clone UTERU2014898,	9	105.4	100.1	98.6	97.2	102.75	97.9	0.952798054	0.623412
P53621	COPA	Coatomer subunit alpha OS=Homo sapiens G	43	103.6	101.9	98.6	97.2	102.75	97.9	0.952798054	0.623412
Q8N4A0	GALNT4	Polypeptide N-acetylgalactosaminyltransferas	6	107.4	102.2	102	97.7	104.8	99.85	0.952767176	0.623039
P43246	MSH2	DNA mismatch repair protein Msh2 OS=Hom	29	104.2	101.1	98.3	97.3	102.65	97.8	0.95275207	0.622856
Q6STE5	SMARCD3	SWI/SNF-related matrix-associated actin-depe	3	106.1	99.1	101.4	94.1	102.6	97.75	0.952729045	0.622577
A0A140VK41		Testicular secretory protein Li 41 OS=Homo s	5	98.8	108.4	102.8	94.6	103.6	98.7	0.952702703	0.622259
Q9UDX5	MTFP1	Mitochondrial fission process protein 1 OS=H	3	104.3	98.6	96.6	96.7	101.45	96.65	0.952686052	0.622058
A0A087X211	KIAA1524	Protein CIP2A OS=Homo sapiens GN=KIAA	4	102.8	100	94.1	99.1	101.4	96.6	0.952662722	0.621776
Q6MZM3	DKFZp686C21	Putative uncharacterized protein DKFZp686C	14	102.9	102	97.5	97.7	102.45	97.6	0.952659834	0.621741
A0A024R2I7	RAD18	RAD18 homolog (S. cerevisiae), isoform CRA	3	101.4	101.2	94.1	98.9	101.3	96.5	0.952615992	0.621211
A0A024R7E0	BTBD14B	BTB (POZ) domain containing 14B, isoform (	4	104.5	97.9	93.7	99.1	101.2	96.4	0.95256917	0.620646
Q86VR2	FAM134C	Protein FAM134C OS=Homo sapiens GN=FA	2	104.5	100	97.7	97.1	102.25	97.4	0.952567237	0.620622
O95639	CPSF4	Cleavage and polyadenylation specificity facto	6	105.9	100.7	99.6	97.2	103.3	98.4	0.952565344	0.6206
P29401	TKT	Transketolase OS=Homo sapiens GN=TKT P	25	99.4	102.9	96.3	96.4	101.15	96.35	0.952545724	0.620363
Q9NQG5	RPRD1B	Regulation of nuclear pre-mRNA domain-con	8	102.2	100.1	95.7	97	101.15	96.35	0.952545724	0.620363
Q9Y3C8	UFC1	Ubiquitin-fold modifier-conjugating enzyme 1	5	105.2	99.1	97.2	97.4	102.15	97.3	0.952520803	0.620062
A0JP11	PIK3R4	Phosphoinositide-3-kinase, regulatory subunit	5	107	99.4	98.5	98.1	103.2	98.3	0.95251938	0.620045
O95425	SVIL	Supervillin OS=Homo sapiens GN=SVIL PE=	6	110.9	101.8	102.1	100.5	106.35	101.3	0.95251528	0.619996
V9HW48	HEL-S-115	SH3 domain-binding glutamic acid-rich-like p	5	96.7	96.6	88.4	95.7	96.65	92.05	0.952405587	0.618672
Q8WWK9	CKAP2	Cytoskeleton-associated protein 2 OS=Homo	1	105.3	98.3	94.9	99	101.8	96.95	0.952357564	0.618094
Q9BXV9	C14orf142	Uncharacterized protein C14orf142 OS=Hom	1	117.2	84.1	95.6	96.1	100.65	95.85	0.952309985	0.61752
Q9UNS1	TIMELESS	Protein timeless homolog OS=Homo sapiens (	5	108.2	101.2	98.2	101.2	104.7	99.7	0.952244508	0.616731
A0A024R6B7	CHURC1	Churchill domain containing 1, isoform CRA_	1	104.3	94.3	91.1	98	99.3	94.55	0.952165156	0.615775
A0A068BUJ5	ND2	NADH-ubiquinone oxidoreductase chain 2 OS	1	99	109.9	108.2	90.7	104.45	99.45	0.952130206	0.615355
P13674	P4HA1	Prolyl 4-hydroxylase subunit alpha-1 OS=Hor	1	99	111.7	94.3	106.3	105.35	100.3	0.952064547	0.614565
P49903	SEPHS1	Selenide, water dikinase 1 OS=Homo sapiens	7	107.3	99	100.2	96.2	103.15	98.2	0.952011634	0.613929
O60476	MAN1A2	Mannosyl-oligosaccharide 1,2-alpha-mannosi	1	99.1	98.6	90.8	97.4	98.85	94.1	0.951947395	0.613156
J3KQY1	MRPL22	39S ribosomal protein L22, mitochondrial OS	9	107.2	98.8	98.7	97.4	103	98.05	0.951941748	0.613088
A0A1B0GVF3	CPT2	Carnitine O-palmitoyltransferase 2, mitochon	15	104.4	101.4	96.8	99.1	102.9	97.95	0.951895044	0.612528
Q9NWY4	HPF1	Histone PARylation factor 1 OS=Homo sapie	2	107.8	95.7	101	92.7	101.75	96.85	0.951842752	0.611899
Q96N67	DOCK7	Dedicator of cytokinesis protein 7 OS=Homo	13	106.3	103.4	98.5	101.1	104.85	99.8	0.951835956	0.611818
Q96J42	TXNDC15	Thioredoxin domain-containing protein 15 OS	2	109.4	91.9	95.6	96	100.65	95.8	0.951813214	0.611545
P39880	CUX1	Homeobox protein cut-like 1 OS=Homo sapie	12	106.2	101.3	98.4	99.1	103.75	98.75	0.951807229	0.611473
P58557	YBEY	Putative ribonuclease OS=Homo sapiens GN=	2	110.4	103.1	107.4	95.8	106.75	101.6	0.95175644	0.610863
P55060	CSE1L	Exportin-2 OS=Homo sapiens GN=CSE1L PE	31	102	98.9	97.1	94.1	100.45	95.6	0.951717272	0.610393
P49454	CENPF	Centromere protein F OS=Homo sapiens GN=	17	106.8	98.1	95.7	99.3	102.45	97.5	0.951683748	0.609992
A0A024R161	DNAJC25-GNG	Guanine nucleotide-binding protein subunit ga	2	105.9	108.4	101.9	102	107.15	101.95	0.951469902	0.607429
Q14527	HLTF	Helicase-like transcription factor OS=Homo s	13	106	102	99.4	98.5	104	98.95	0.951442308	0.607098
A8K6K2		cDNA FLJ75877, highly similar to Homo sap	12	100.7	101.1	96	96	100.9	96	0.951437066	0.607036
A0A024R6F4	NUMB	Numb homolog (Drosophila), isoform CRA_c	5	106.3	103.7	101.5	98.3	105	99.9	0.951428571	0.606934
O94888	UBXN7	UBX domain-containing protein 7 OS=Homo	1	100	99.5	91.2	98.6	99.75	94.9	0.951378446	0.606335
B2R9D9		cDNA, FLJ94347 OS=Homo sapiens PE=2 SV	2	103.4	100.2	95.7	98	101.8	96.85	0.951375246	0.606296
Q96PC3	AP1S3	AP-1 complex subunit sigma-3 OS=Homo sap	1	114.5	101.4	98.4	107	107.95	102.7	0.951366373	0.60619
Q9P031	CCDC59	Thyroid transcription factor 1-associated prote	5	106.7	103	106	93.5	104.85	99.75	0.951359084	0.606103
P53992	SEC24C	Protein transport protein Sec24C OS=Homo s	11	101.6	103.9	98.3	97.2	102.75	97.75	0.9513382	0.605852
Q9UL15	BAG5	BAG family molecular chaperone regulator 5	3	104.4	98.5	94.5	98.5	101.45	96.5	0.951207491	0.604291
A0A024R625	PPP4C	Serine/threonine-protein phosphatase OS=Ho	6	103	103.7	98.5	98.1	103.35	98.3	0.951136913	0.603447
B4DZH7		cDNA FLJ56367 OS=Homo sapiens PE=2 SV	1	106.2	98.3	79.2	115.3	102.25	97.25	0.951100244	0.603009
I3L4A1	CHMP6	Charged multivesicular body protein 6 (Fragm	3	106.3	102.2	100.5	97.8	104.25	99.15	0.951079137	0.602757
A0A024RDS1	HSPH1	Heat shock 105kDa/110kDa protein 1, isoform	26	102.1	102.3	97.2	97.2	102.2	97.2	0.951076321	0.602724
Q9NVM6	DNAJC17	DnaJ homolog subfamily C member 17 OS=H	3	101.6	98.7	92.6	97.9	100.15	95.25	0.95107339	0.602689
Q13085	ACACA	Acetyl-CoA carboxylase 1 OS=Homo sapiens	29	103	101.1	96.6	97.5	102.05	97.05	0.95100441	0.601865
Q9UGP4	LIMD1	LIM domain-containing protein 1 OS=Homo	4	109.8	100.3	96.8	103	105.05	99.9	0.950975726	0.601523
Q06587	RING1	E3 ubiquitin-protein ligase RING1 OS=Homo	3	100.4	101.5	95.5	96.5	100.95	96	0.950965825	0.601405
P78345	RPP38	Ribonuclease P protein subunit p38 OS=Homo	5	107.7	100.2	102.5	95.2	103.95	98.85	0.950937951	0.601073
Q96ME7	ZNF512	Zinc finger protein 512 OS=Homo sapiens GN	1	120.9	101.1	110.3	100.8	111	105.55	0.950900901	0.600631
Q9UKV8	AGO2	Protein argonaute-2 OS=Homo sapiens GN=A	7	99.6	95.6	89.4	96.2	97.6	92.8	0.950819672	0.599663
Q9UNS2	COPS3	COP9 signalosome complex subunit 3 OS=Ho	9	101.7	103.6	97.3	97.9	102.65	97.6	0.950803702	0.599473
V9HW90	HEL-75	Epididymis luminal protein 75 OS=Homo sap	16	99.5	101.7	95.2	96.1	100.6	95.65	0.950795229	0.599372
Q7L592	NDUFAF7	Protein arginine methyltransferase NDUFAF7	2	106.5	94.7	91.1	100.2	100.6	95.65	0.950795229	0.599372
Q9ULM3	YEATS2	YEATS domain-containing protein 2 OS=Hor	2	104.7	100.5	92.8	102.3	102.6	97.55	0.950779727	0.599188
Q15056	EIF4H	Eukaryotic translation initiation factor 4H OS	5	101	100	96.4	94.7	100.5	95.55	0.950746269	0.59879
Q9UJA5	TRMT6	tRNA (adenine(58)-N(1))-methyltransferase n	1	104.4	112.8	104.5	102	108.6	103.25	0.950736648	0.598675
E5KNQ5	TK2	Mitochondrial thymidine kinase 2 OS=Homo	1	95.4	109.5	96.5	98.3	102.45	97.4	0.950707662	0.59833
Q13608	PEX6	Peroxisome assembly factor 2 OS=Homo sapi	2	98.5	92	87.3	93.8	95.25	90.55	0.950656168	0.597717
Q96ST3	SIN3A	Paired amphipathic helix protein Sin3a OS=H	16	103.6	102.9	97.7	98.6	103.25	98.15	0.950605327	0.597112
Q9HC35	EML4	Echinoderm microtubule-associated protein-li	8	99	101.4	93.4	97.1	100.2	95.25	0.950598802	0.597034
Q9NQW7	XPNPEP1	Xaa-Pro aminopeptidase 1 OS=Homo sapiens	6	101.3	101	97	95.3	101.15	96.15	0.950568463	0.596674
Q86Y56	DNAAF5	Dynein assembly factor 5, axonemal OS=Hon	16	99.1	103.2	94.2	98.1	101.15	96.15	0.950568463	0.596674
A8K4U4		cDNA FLJ77113 OS=Homo sapiens PE=2 SV	3	106.3	103.9	98.8	101	105.1	99.9	0.950523311	0.596137
M0R1T5	CHMP2A	Charged multivesicular body protein 2a (Frag	3	97	92.9	88	92.5	94.95	90.25	0.950500263	0.595863
Q9NRP2	CMC2	COX assembly mitochondrial protein 2 homo	3	105.2	102.6	101.7	95.8	103.9	98.75	0.950433109	0.595066
A0A024QZE6	C16orf58	Chromosome 16 open reading frame 58, isofo	6	102	101.6	95.8	97.7	101.8	96.75	0.950392927	0.594588
Q93008	USP9X	Probable ubiquitin carboxyl-terminal hydrolas	16	101.9	101.4	97.7	95.5	101.65	96.6	0.950319725	0.59372
O00154	ACOT7	Cytosolic acyl coenzyme A thioester hydrolas	8	102.8	100.4	94.7	98.4	101.6	96.55	0.950295276	0.59343
A0A0X1KG71	NELFB	Negative elongation factor B OS=Homo sapie	4	107.3	99.9	100.1	96.8	103.6	98.45	0.950289575	0.593362
A0A024RDY0	RANBP5	RAN binding protein 5, isoform CRA_d OS=	26	103.3	101.8	98.4	96.5	102.55	97.45	0.950268162	0.593108

Q9NP61	ARFGAP3	ADP-ribosylation factor GTPase-activating pr	6	104.4	102.6	100.2	96.5	103.5	98.35	0.950241546	0.592793
Q53HB4		M-phase phosphoprotein 6 variant (Fragment)	4	99.3	101.6	97	93.9	100.45	95.45	0.950223992	0.592584
Q9NX08	COMMD8	COMM domain-containing protein 8 OS=Hor	4	103.4	99.4	94.8	97.9	101.4	96.35	0.950197239	0.592267
Q9NY33	DPP3	Dipeptidyl peptidase 3 OS=Homo sapiens GN	12	101.3	101.4	94.5	98.1	101.35	96.3	0.950172669	0.591976
A0A0B4J1R7	BORCS7-ASM	Protein BORCS7-ASMT OS=Homo sapiens C	3	107.1	97.4	95.7	98.6	102.25	97.15	0.950122249	0.591379
P83111	LACTB	Serine beta-lactamase-like protein LACTB, m	2	109.9	100.6	103.8	96.2	105.25	100	0.950118765	0.591338
P62633	CNBP	Cellular nucleic acid-binding protein OS=Hor	6	107.5	102.9	100.7	99.2	105.2	99.95	0.950095057	0.591057
Q8WZ82	OVCA2	Esterase OVCA2 OS=Homo sapiens GN=OV	2	107.4	92.9	93.8	96.5	100.15	95.15	0.950074888	0.590818
B3KT21		cDNA FLJ37476 fis, clone BRAWH2012827	3	99.7	102.6	94	98.2	101.15	96.1	0.950074147	0.590809
Q969V5	MUL1	Mitochondrial ubiquitin ligase activator of NF	5	102	104.3	97.2	98.8	103.15	98	0.95007271	0.590792
B4DJV9		cDNA FLJ60607, highly similar to Acyl-prote	5	101.2	102.5	97.2	96.3	101.85	96.75	0.949926362	0.58906
Q8WUB8	PHF10	PHD finger protein 10 OS=Homo sapiens GN	5	104.7	99	97.3	96.2	101.85	96.75	0.949926362	0.58906
Q9H832	UBE2Z	Ubiquitin-conjugating enzyme E2 Z OS=Hom	4	97.9	103.8	95.5	96.1	100.85	95.8	0.949925632	0.589052
Q9H0R6	QRSL1	Glutamyl-tRNA(Gln) amidotransferase subun	11	104.5	97.1	95.1	96.4	100.8	95.75	0.949900794	0.588758
Q14139	UBE4A	Ubiquitin conjugation factor E4 A OS=Homo	7	104.6	98.9	98.1	95.2	101.75	96.65	0.94987715	0.588478
A0A024RD18	STK38	Serine/threonine kinase 38, isoform CRA_a C	1	114.4	99	100.2	102.5	106.7	101.35	0.949859419	0.588269
Q86TP1	PRUNE	Protein prune homolog OS=Homo sapiens GN	2	103.7	101.7	88.2	106.9	102.7	97.55	0.949853944	0.588204
Q9BVG4	PBDC1	Protein PBDC1 OS=Homo sapiens GN=PBD(	8	103.5	101.9	99.5	95.6	102.7	97.55	0.949853944	0.588204
Q9H5X1	FAM96A	MIP18 family protein FAM96A OS=Homo sa	2	104.1	103.2	96.3	100.6	103.65	98.45	0.949831163	0.587935
A0A024R7X1	TERF1	Telomeric repeat binding factor (NIMA-intera	1	101.3	104	90.6	104.4	102.65	97.5	0.949829518	0.587915
P00387	CYB5R3	NADH-cytochrome b5 reductase 3 OS=Homo	13	112.6	100.5	103.2	99.2	106.55	101.2	0.949788832	0.587434
Q8NAF0	ZNF579	Zinc finger protein 579 OS=Homo sapiens GN	2	100.5	104.6	99.6	95.2	102.55	97.4	0.949780595	0.587337
P14735	IDE	Insulin-degrading enzyme OS=Homo sapiens	22	102.8	99.9	96.1	96.4	101.35	96.25	0.949679329	0.586141
O14579	COPE	Coatomer subunit epsilon OS=Homo sapiens	11	104.2	104.4	98.3	99.8	104.3	99.05	0.94966443	0.585966
Q96AJ9	VTI1A	Vesicle transport through interaction with t-SI	1	106.9	95.7	94.3	98.1	101.3	96.2	0.949654492	0.585849
A0A024R7F9	GCDH	Glutaryl-Coenzyme A dehydrogenase, isoforr	7	100.4	103.9	95.6	98.4	102.15	97	0.949583945	0.585016
B7ZMD6	IRGQ	IRGQ protein OS=Homo sapiens GN=IRGQ	5	103.8	96.3	92.3	97.7	100.05	95	0.949525237	0.584324
Q9H0H0	INTS2	Integrator complex subunit 2 OS=Homo sapie	6	101.4	100.6	93.7	98.1	101	95.9	0.94950495	0.584085
Q86VX2	COMMD7	COMM domain-containing protein 7 OS=Hor	1	109.1	102.8	95.7	105.5	105.95	100.6	0.949504483	0.58408
K7ESQ2	SPC24	Kinetochore protein Spc24 (Fragment) OS=H	1	98.6	103.3	91.3	100.4	100.95	95.85	0.949479941	0.58379
Q86VS8	HOOK3	Protein Hook homolog 3 OS=Homo sapiens C	5	109.1	96.4	96.1	99	102.75	97.55	0.949391727	0.582751
A0A0S2Z2Z3	ABCB7	ATP-binding cassette sub-family B member 7	10	104.9	100.5	98.4	96.6	102.7	97.5	0.949367089	0.582461
Q9UPM8	AP4E1	AP-4 complex subunit epsilon-1 OS=Homo sa	5	101.7	99.7	92.8	98.4	100.7	95.6	0.949354518	0.582313
O14562	UBFD1	Ubiquitin domain-containing protein UBFD1	1	111.2	96.1	105.9	90.9	103.65	98.4	0.94934877	0.582245
Q9BTC8	MTA3	Metastasis-associated protein MTA3 OS=Hor	4	106.3	99	97.3	97.6	102.65	97.45	0.949342426	0.582171
Q53FR9	COMMD9	COMM domain containing 9 variant (Fragme	2	104.1	105.1	97.3	101.3	104.6	99.3	0.949330784	0.582034
Q9UHD8	SEPT9	Septin-9 OS=Homo sapiens GN=SEPT9 PE=	22	101	104.2	97	97.8	102.6	97.4	0.949317739	0.58188
A0A087WU03	HNRNPDL	Heterogeneous nuclear ribonucleoprotein D-li	1	104.4	96.7	95.6	95.3	100.55	95.45	0.949278966	0.581424
B3KR33		cDNA FLJ33589 fis, clone BRAMY2012536,	1	110.5	96.5	93.1	103.4	103.5	98.25	0.949275362	0.581382
A0MZ66	SHTN1	Shootin-1 OS=Homo sapiens GN=SHTN1 PE	5	109	96	97	97.6	102.5	97.3	0.949268293	0.581298
Q15126	PMVK	Phosphomevalonate kinase OS=Homo sapiens	5	101.9	102.8	97.8	96.5	102.35	97.15	0.949193942	0.580425
B1ANH3	GUK1	Guanylate kinase (Fragment) OS=Homo sapie	1	106.9	97.6	100.7	93.4	102.25	97.05	0.949144254	0.57984
Q92990	GLMN	Glomulin OS=Homo sapiens GN=GLMN PE	7	101.5	100.9	95.7	96.4	101.2	96.05	0.949110672	0.579446
Q9NR50	EIF2B3	Translation initiation factor eIF-2B subunit ga	4	106	103.7	97.2	101.8	104.85	99.5	0.948974726	0.57785
B5M450	SLC4A7	Anion exchange protein OS=Homo sapiens G	2	98.4	99.4	96.6	91.1	98.9	93.85	0.948938322	0.577423
Q6MZT3	DKFZp686C10	Putative uncharacterized protein DKFZp686C	3	105.9	99.7	97.3	97.8	102.8	97.55	0.948929961	0.577325
E9PN70	TRAPPC4	Trafficking protein particle complex subunit 4	3	107.2	98.4	96.8	98.3	102.8	97.55	0.948929961	0.577325
A0A024R411	ORC2L	Origin recognition complex, subunit 2-like (Y	9	105.9	99.6	96.7	98.3	102.75	97.5	0.948905109	0.577033
B5BUI8	DUSP3	Dual specificity phosphatase 3 (Fragment) OS	6	105.8	103.6	98.9	99.8	104.7	99.35	0.948901624	0.576993
A0A024R4E9	RERE	Arginine-glutamic acid dipeptide (RE) repeats	3	108.9	102.4	99.4	101.1	105.65	100.25	0.948887837	0.576831
Q7RTP0	NIPA1	Magnesium transporter NIPA1 OS=Homo sap	3	102	95.5	95.1	92.3	98.75	93.7	0.948860759	0.576513
P20618	PSMB1	Proteasome subunit beta type-1 OS=Homo sa	9	102.4	104.6	99.7	96.7	103.5	98.2	0.948792271	0.575711
A0A024RBR3	DENR	Density-regulated protein OS=Homo sapiens	2	104	106.4	97.2	102.4	105.2	99.8	0.948669202	0.57427
P78332	RBM6	RNA-binding protein 6 OS=Homo sapiens GN	11	104.9	99	97.4	96	101.95	96.7	0.948504169	0.57234
Q9Y3D0	FAM96B	Mitotic spindle-associated MMXD complex s	2	104.7	101	94.8	100.3	102.85	97.55	0.948468644	0.571925
Q8TAM9	ICAM5	Intercellular adhesion molecule 5, telencephal	1	96.4	101.5	96.3	91.4	98.95	93.85	0.948458818	0.57181
Q7Z2Z1	TICRR	Treslin OS=Homo sapiens GN=TICRR PE=1	1	98.8	106.8	96	99	102.8	97.5	0.94844358	0.571632
Q562T7	ACT	Actin-like protein (Fragment) OS=Homo sapi	1	100	105.5	96.9	98	102.75	97.45	0.948418491	0.571339
A0A0S2Z392	ADRBK1	Protein-serine/threonine kinase (Fragment) OS	3	98.6	104.9	94.5	98.5	101.75	96.5	0.948402948	0.571158
A0A024QZU8	RREB1	Ras responsive element binding protein 1, iso	4	99	102.4	97.9	93.1	100.7	95.5	0.94836147	0.570673
A0A024RD07	TNRC5	Trinucleotide repeat containing 5, isoform CR	5	106.3	98.6	96.9	97.4	102.45	97.15	0.948267448	0.569577
B4E2Q0	ATP2C1	Calcium-transporting ATPase OS=Homo sapi	1	106.6	96.3	100.1	92.3	101.45	96.2	0.94825037	0.569377
P01031	C5	Complement C5 OS=Homo sapiens GN=C5 I	1	106.7	101.9	98.6	99.2	104.3	98.9	0.94822627	0.569096
Q8NFZ8	CADM4	Cell adhesion molecule 4 OS=Homo sapiens (	3	102.4	102.2	97	97	102.3	97	0.948191593	0.568692
A0A024RCN9	EHMT2	Euchromatic histone-lysine N-methyltransfera	12	101.5	99.2	93.6	96.7	100.35	95.15	0.948181365	0.568573
D3DS14	FLJ10357	Uncharacterized protein OS=Homo sapiens G	1	109.7	100.6	95.5	103.9	105.15	99.7	0.948169282	0.568432
A2RRC9	IQGAP3	IQ motif containing GTPase activating protei	4	104.4	100.1	94.6	99.3	102.25	96.95	0.948166259	0.568397
O14618	CCS	Copper chaperone for superoxide dismutase C	2	101.7	98.9	93.2	97	100.3	95.1	0.948155533	0.568272
B2R6X8		cDNA, FLJ93169, highly similar to Homo sap	6	104.5	103.1	97.5	99.3	103.8	98.4	0.947976879	0.566192
Q9Y2K3	MYH15	Myosin-15 OS=Homo sapiens GN=MYH15 F	1	108.2	99.4	90.1	106.7	103.8	98.4	0.947976879	0.566192
P06132	UROD	Uroporphyrinogen decarboxylase OS=Homo s	6	100.4	99.5	95.3	94.2	99.95	94.75	0.947973987	0.566159
A0A0S2Z3F2	ARAF	V-raf murine sarcoma 3611 viral oncogene-lil	1	112	97.3	98.2	100.2	104.65	99.2	0.947921644	0.56555
Q9H857	NT5DC2	5'-nucleotidase domain-containing protein 2 C	17	102	101.5	95	97.9	101.75	96.45	0.947911548	0.565433
A0A024R397	PANX1	Pannexin OS=Homo sapiens GN=PANX1 PE	3	113.1	93.7	99.2	96.8	103.4	98	0.947775629	0.563853
P02686	MBP	Myelin basic protein OS=Homo sapiens GN=	1	106.3	94.7	93.8	96.7	100.5	95.25	0.947761194	0.563686
Q5GLZ8	HERC4	Probable E3 ubiquitin-protein ligase HERC4 (	4	101.8	104.9	94.7	101.2	103.35	97.95	0.947750363	0.56356
A0A024R2L0	UBP1	Upstream binding protein 1 (LBP-1a), isoforn	1	98.5	96.6	88.2	96.7	97.55	92.45	0.947719118	0.563197
Q9UK39	NOCT	Nocturnin OS=Homo sapiens GN=NOCT PE	5	101	97.9	93.3	95.2	99.45	94.25	0.947712418	0.56312
Q96BI3	APH1A	Gamma-secretase subunit APH-1A OS=Homo	1	103.7	95.2	92.6	95.9	99.45	94.25	0.947712418	0.56312
A8MU27	SUMO3	Small ubiquitin-related modifier 3 OS=Homo	1	84.6	85.6	81.4	79.9	85.1	80.65	0.947708578	0.563076
Q9H3K6	BOLA2	BolA-like protein 2 OS=Homo sapiens GN=E	3	103.2	99.5	93.7	98.4	101.35	96.05	0.947705969	0.563045
O75528	TADA3	Transcriptional adapter 3 OS=Homo sapiens (	3	102.6	107.7	101.9	97.4	105.15	99.65	0.947693771	0.562903
Q8N128	FAM177A1	Protein FAM177A1 OS=Homo sapiens GN=I	4	111	99.2	96.6	102.6	105.1	99.6	0.947668887	0.562615
F8W031		Uncharacterized protein (Fragment) OS=Hom	10	107.8	98.5	98	97.5	103.15	97.75	0.947649055	0.562385
E7EQY1	FAM136A	Protein FAM136A OS=Homo sapiens GN=F/	3	102.8	101.4	100.7	92.8	102.1	96.75	0.947600392	0.56182
Q14558	PRPSAP1	Phosphoribosyl pyrophosphate synthase-assoc	6	104.4	101.7	94.9	100.4	103.05	97.65	0.947598253	0.561796
Q06124	PTPN11	Tyrosine-protein phosphatase non-receptor ty	11	104.2	101.7	95.2	99.9	102.95	97.55	0.947547353	0.561206
A0A1B0GW7	ALDH7A1	Alpha-aminoadipic semialdehyde dehydrogen	21	101.7	102.2	97.4	95.8	101.95	96.6	0.947523296	0.560927
O15050	TRANK1	TPR and ankyrin repeat-containing protein 1 (	1	111.4	103.8	101.8	102.1	107.6	101.95	0.947490706	0.560549
B4DLV7		cDNA FLJ60299, highly similar to Rab GDP	21	99.8	102	96.8	94.4	100.9	95.6	0.947472745	0.560341
Q9BZG1	RAB34	Ras-related protein Rab-34 OS=Homo sapiens	12	103	102.5	97.4	97.3	102.75	97.35	0.947445255	0.560023
A0A090N8E9	EZH2	Enhancer of zeste homolog 2 (Drosophila) OS	13	103	100.5	95.8	97	101.75	96.4	0.947420147	0.559732
Q12792	TWF1	Twinfilin-1 OS=Homo sapiens GN=TWF1 PE	8	103.4	102	97.9	96.7	102.7	97.3	0.947419669	0.559727
B3KQ33		cDNA FLJ32715 fis, clone TESTI2000784, h	10	105.4	100	97.3	97.3	102.7	97.3	0.947419669	0.559727
A0A0K0K1K7	HEL-S-304	6-phosphogluconolactonase, isoform CRA_b	8	101.6	99.9	93.7	97.2	100.75	95.45	0.947394541	0.559436
Q9Y232	CDYL	Chromodomain Y-like protein OS=Homo sap	3	102.3	103	95	99.5	102.65	97.25	0.947394057	0.559431
Q8N6X1	LOC541472	LOC541472 protein OS=Homo sapiens GN=I	1	103.7	101.3	98.4	95.8	102.5	97.1	0.947317073	0.55854
O75391	SPAG7	Sperm-associated antigen 7 OS=Homo sapien	6	102.1	99.1	92.7	97.9	100.6	95.3	0.947316103	0.558529
A7E2E5	PDE3B	Phosphodiesterase OS=Homo sapiens GN=PI	2	107.3	101.4	102.6	95.1	104.35	98.85	0.947292765	0.558259
M0QXB5	ETHE1	Persulfide dioxygenase ETHE1, mitochondria	6	101.7	101.3	96.7	95.6	101.5	96.15	0.94729064	0.558234
Q4LE38	IKBKAP	IKBKAP variant protein (Fragment) OS=Hom	8	101.5	97.7	93.9	94.8	99.6	94.35	0.947289157	0.558217
Q7L576	CYFIP1	Cytoplasmic FMR1-interacting protein 1 OS=	7	102.3	102.4	99.8	94.1	102.35	96.95	0.947239863	0.557648
F5H5P2		Uncharacterized protein OS=Homo sapiens Pl	3	105.2	101.1	93.5	101.9	103.15	97.7	0.947164324	0.556774
A0A024R9G7	ATAD2	ATPase family, AAA domain containing 2, is	12	103.7	100.5	95.9	97.5	102.1	96.7	0.947110676	0.556155
Q9BRA2	TXNDC17	Thioredoxin domain-containing protein 17 OS	4	101.3	101	94.3	97.3	101.15	95.8	0.947108255	0.556128
P04066	FUCA1	Tissue alpha-L-fucosidase OS=Homo sapiens	4	101.9	96.5	91.8	96.1	99.2	93.95	0.947076613	0.555762
Q9BSJ2	TUBGCP2	Gamma-tubulin complex component 2 OS=H	1	107.9	99.8	98.3	98.4	103.85	98.35	0.947038999	0.555328
Q96S55	WRNIP1	ATPase WRNIP1 OS=Homo sapiens GN=WI	7	103	104.6	98	98.6	103.8	98.3	0.947013487	0.555034
A0A024R5U9	C1orf71	Chromosome 1 open reading frame 71, isofor	1	94.3	107.6	100.5	90.7	100.95	95.6	0.947003467	0.554919
Q53F18		Homer 1 variant (Fragment) OS=Homo sapie	2	106.4	101.1	98.7	97.8	103.75	98.25	0.946987952	0.554739
Q9H0L4	CSTF2T	Cleavage stimulation factor subunit 2 tau vari	3	103.7	101.9	93.7	101	102.8	97.35	0.946984436	0.554699
Q96BP2	CHCHD1	Coiled-coil-helix-coiled-coil-helix domain-co	3	106.1	103.2	100.8	97.4	104.65	99.1	0.946966077	0.554487
L0R588	C11orf48	Alternative protein C11orf48 OS=Homo sapie	4	98.9	95.3	93.6	90.3	97.1	91.95	0.946961895	0.554439
A0A1B0GTL5	RAB11FIP5	Rab11 family-interacting protein 5 OS=Homo	3	103.8	97.9	87.8	103.2	100.85	95.5	0.946950917	0.554313
P61457	PCBD1	Pterin-4-alpha-carbinolamine dehydratase OS	3	101.9	99.7	98.1	92.8	100.8	95.45	0.946924603	0.55401
P49588	AARS	Alanine--tRNA ligase, cytoplasmic OS=Homo	31	101.8	99.7	95.7	95.1	100.75	95.4	0.946898263	0.553706
B4E0E0		cDNA FLJ54898, highly similar to Homo sap	4	100.1	99.4	96.5	92.4	99.75	94.45	0.946867168	0.553348
F2Z2W7	TRMT2A	tRNA (uracil-5-)-methyltransferase homolog .	8	101.9	103.2	96.6	97.6	102.55	97.1	0.946855193	0.55321
J3QLM1	STARD3	StAR-related lipid transfer protein 3 OS=Hom	2	104.5	102.3	96.6	99.2	103.4	97.9	0.946808511	0.552673
O95295	SNAPIN	SNARE-associated protein Snapin OS=Homo	2	108.6	99.9	97.5	99.9	104.25	98.7	0.94676259	0.552144
L0R8F8	SMCR7L	Alternative protein SMCR7L OS=Homo sapie	1	105.3	99.4	92.3	101.5	102.35	96.9	0.946751343	0.552015
O95219	SNX4	Sorting nexin-4 OS=Homo sapiens GN=SNX	4	109.7	104.3	104.1	98.5	107	101.3	0.946728972	0.551757

A0A140VKC8		Testis tissue sperm-binding protein Li 45a OS	3	100.8	96.3	93.4	93.2	98.55	93.3	0.946727549	0.551741
B2R7C5		DNA helicase OS=Homo sapiens PE=2 SV=1	40	102.7	103.5	98.1	97.1	103.1	97.6	0.946653734	0.550892
B2R5M8		Isocitrate dehydrogenase [NADP] OS=Homo	15	97.8	100.9	94.5	93.6	99.35	94.05	0.946653246	0.550887
Q5NDL2	EOGT	EGF domain-specific O-linked N-acetylgluco	8	106.8	100.8	99.3	97.2	103.8	98.25	0.946531792	0.549491
P61803	DAD1	Dolichyl-diphosphooligosaccharide--protein g	4	107.9	97.8	100.5	94.2	102.85	97.35	0.946524064	0.549403
B4DZE5		cDNA FLJ58806, highly similar to DNA-dire	1	105	100.7	101.9	92.8	102.85	97.35	0.946524064	0.549403
A0A024R9W0	LIAS	Lipoyl synthase, mitochondrial OS=Homo sap	1	104.7	99.1	98.1	94.8	101.9	96.45	0.946516192	0.549312
Q9HD23	MRS2	Magnesium transporter MRS2 homolog, mito	6	105.1	98.2	94.3	98.1	101.65	96.2	0.946384653	0.547803
A0A087X0M4	SLC4A1AP	Kanadaptin OS=Homo sapiens GN=SLC4A1.	8	105.3	101.6	95.7	100.1	103.45	97.9	0.946350894	0.547416
O00258	WRB	Tail-anchored protein insertion receptor WRB	1	114.3	88.8	94.2	98	101.55	96.1	0.946331856	0.547198
P14618	PKM	Pyruvate kinase PKM OS=Homo sapiens GN	5	104.4	100.4	96.5	97.3	102.4	96.9	0.946289063	0.546708
A0A024R4I7	LZIC	Leucine zipper and CTNNBIP1 domain conta	2	94.2	100.6	91.6	92.7	97.4	92.15	0.946098563	0.544527
Q99584	S100A13	Protein S100-A13 OS=Homo sapiens GN=S1	5	96.1	102.3	93.1	94.6	99.2	93.85	0.946068548	0.544184
Q9HCS7	XAB2	Pre-mRNA-splicing factor SYF1 OS=Homo s	22	102.9	100.9	96.4	96.4	101.9	96.4	0.946025515	0.543692
Q53EV6		Basic leucine zipper nuclear factor 1 variant (	1	109.2	96.4	96.1	98.4	102.8	97.25	0.946011673	0.543534
Q13308	PTK7	Inactive tyrosine-protein kinase 7 OS=Homo s	4	113	98.1	98	101.7	105.55	99.85	0.945997158	0.543368
C9JA08	NMD3	60S ribosomal export protein NMD3 OS=Hor	6	103.5	103.8	98.1	98	103.65	98.05	0.945972021	0.543081
E9PGK7	TRPM2	Transient receptor potential cation channel sul	1	103.3	96.5	97.1	91.9	99.9	94.5	0.945945946	0.542783
Q53G08		DNA replication complex GINS protein PSF2	1	106.9	94.5	93.7	96.8	100.7	95.25	0.945878848	0.542018
Q6UW78	UQCC3	Ubiquinol-cytochrome-c reductase complex a	1	105.9	88.1	88.5	95	97	91.75	0.945876289	0.541988
I3L504	EIF5A	Eukaryotic translation initiation factor 5A-1 C	11	104	102.9	98.9	96.8	103.45	97.85	0.945867569	0.541889
Q9NQH7	XPNPEP3	Probable Xaa-Pro aminopeptidase 3 OS=Hom	5	102.8	98.3	96.8	93.4	100.55	95.1	0.94579811	0.541096
A2A2Q9	AAR2	Protein AAR2 homolog OS=Homo sapiens G	2	103.5	95.7	92.5	95.9	99.6	94.2	0.945783133	0.540926
A0A0G2JR96	CYFIP1	Cytoplasmic FMR1-interacting protein 1 (Fra	1	105.8	100.5	99.5	95.6	103.15	97.55	0.945710131	0.540094
B4DS05		cDNA FLJ59403, highly similar to Nucleosor	6	104.7	103.2	97.7	98.9	103.95	98.3	0.945646946	0.539374
Q15418	RPS6KA1	Ribosomal protein S6 kinase alpha-1 OS=Hor	5	101.2	103	94.8	98.3	102.1	96.55	0.945641528	0.539312
Q96P47	AGAP3	Arf-GAP with GTPase, ANK repeat and PH d	3	103.6	104.2	94.9	101.6	103.9	98.25	0.945620789	0.539076
Q96PZ2	FAM111A	Protein FAM111A OS=Homo sapiens GN=F.	1	97.4	112.1	108.6	89.5	104.75	99.05	0.945584726	0.538666
Q15059	BRD3	Bromodomain-containing protein 3 OS=Hom	2	110.3	93.6	97.1	95.7	101.95	96.4	0.94556155	0.538402
Q06787	FMR1	Synaptic functional regulator FMR1 OS=Hor	2	103.3	102.4	96.4	98.1	102.85	97.25	0.945551774	0.538291
A0A024R637	TBC1D4	TBC1 domain family, member 4, isoform CR.	1	102	105.1	100.8	95	103.55	97.9	0.945436987	0.536986
Q9H269	VPS16	Vacuolar protein sorting-associated protein 16	10	105.5	97.9	97.7	94.6	101.7	96.15	0.945427729	0.53688
A0A024RD83	TTC19	Tetratricopeptide repeat domain 19, isoform C	5	101.9	97.8	92.4	96.4	99.85	94.4	0.945418127	0.536771
Q9BRS2	RIOK1	Serine/threonine-protein kinase RIO1 OS=Ho	1	94.9	104.8	101	87.8	99.85	94.4	0.945418127	0.536771
A0A024R7C5	MRPL4	Mitochondrial ribosomal protein L4, isoform	9	103.2	103.8	98.2	97.5	103.5	97.85	0.945410628	0.536686
Q9UJW0	DCTN4	Dynactin subunit 4 OS=Homo sapiens GN=D	6	103	107.4	102.7	96.2	105.2	99.45	0.945342205	0.535909
Q712K3	UBE2R2	Ubiquitin-conjugating enzyme E2 R2 OS=Ho	3	105.4	97.6	94.9	97	101.5	95.95	0.945320197	0.535659
Q6P1N0	CC2D1A	Coiled-coil and C2 domain-containing protein	7	105.6	99.2	94.7	98.9	102.4	96.8	0.9453125	0.535572
G4U4J3	PAPD5	PAP associated domain containing 5 OS=Hor	5	97.2	98.4	92.2	92.7	97.8	92.45	0.945296524	0.535391
V9HWA6	HEL32	Epididymis luminal protein 32 OS=Homo sap	9	101.7	103	97.9	95.6	102.35	96.75	0.945285784	0.535269
B2R921		cDNA, FLJ94171, highly similar to Homo sap	3	106.3	105.7	97.7	102.7	106	100.2	0.945283019	0.535237
J3QTA6	CHCHD6	MICOS complex subunit OS=Homo sapiens C	3	102.4	102.2	98.1	95.3	102.3	96.7	0.945259042	0.534965
Q4ZIN3	TMEM259	Membralin OS=Homo sapiens GN=TMEM25	1	98.6	98.6	93.5	92.9	98.6	93.2	0.945233266	0.534673
D3DNI2	PFN2	Profilin (Fragment) OS=Homo sapiens GN=P	4	98.5	100.5	94.4	93.7	99.5	94.05	0.945226131	0.534592
Q504R6	RAB13	RAB13 protein (Fragment) OS=Homo sapien	5	107.9	98.2	95.7	99.1	103.05	97.4	0.945172246	0.533981
B7Z3J6		Non-lysosomal glucosylceramidase OS=Hom	1	104.6	105	94.9	103.2	104.8	99.05	0.945133588	0.533543
Q15436	SEC23A	Protein transport protein Sec23A OS=Homo s	13	102.9	103	96.5	98.1	102.95	97.3	0.94511899	0.533377
Q01650	SLC7A5	Large neutral amino acids transporter small su	2	105.2	107.9	101.7	99.7	106.55	100.7	0.945096199	0.533119
Q7Z4W1	DCXR	L-xylulose reductase OS=Homo sapiens GN=	3	101.3	99	93	96.3	100.15	94.65	0.945082376	0.532963
B2R6S5	CMPK	UMP-CMP kinase OS=Homo sapiens GN=CI	5	101.7	102.2	99.5	93.2	101.95	96.35	0.945071113	0.532835
O43159	RRP8	Ribosomal RNA-processing protein 8 OS=Ho	11	103.6	102.1	98.2	96.2	102.85	97.2	0.94506563	0.532773
O75312	ZPR1	Zinc finger protein ZPR1 OS=Homo sapiens	7	103	100.5	94.9	97.4	101.75	96.15	0.944963145	0.531613
Q13188	STK3	Serine/threonine-protein kinase 3 OS=Homo s	8	104	101.1	97.7	96.1	102.55	96.9	0.944904924	0.530955
Q969R2	OSBP2	Oxysterol-binding protein 2 OS=Homo sapier	1	109.2	95.9	96.5	97.3	102.55	96.9	0.944904924	0.530955
H0Y4Z8	RABL6	Chromosome 9 open reading frame 86, isofor	3	101	101.7	97.6	93.9	101.35	95.75	0.94474593	0.529159
Q13232	NME3	Nucleoside diphosphate kinase 3 OS=Homo s	8	93.9	110.6	95.6	97.6	102.25	96.6	0.944743276	0.529129
A0A087WVB(	EIF2D	Eukaryotic translation initiation factor 2D (Fr	1	105.1	103	95.5	101.1	104.05	98.3	0.944738107	0.52907
Q15833	STXBP2	Syntaxin-binding protein 2 OS=Homo sapiens	6	106.2	101.9	99	97.6	104.05	98.3	0.944738107	0.52907
V9HW53	HEL-S-277	Dimethylarginine dimethylaminohydrolase 2,	3	101.5	101	95.8	95.5	101.25	95.65	0.944691358	0.528543
Q96GX2	ATXN7L3B	Putative ataxin-7-like protein 3B OS=Homo s	1	104.3	76.1	80	90.4	90.2	85.2	0.944567627	0.527148
B3KX82		cDNA FLJ44964 fis, clone BRAWH2016209	4	101.3	100.5	91.7	98.9	100.9	95.3	0.944499504	0.526381
B3KUH0		cDNA FLJ39883 fis, clone SPLEN2016268, l	2	103	98.7	92.1	98.4	100.85	95.25	0.944471988	0.526071
B2R4N3		cDNA, FLJ92155, highly similar to Homo sap	2	102.8	100.6	97.7	94.4	101.7	96.05	0.944444444	0.525761
Q8ND82	ZNF280C	Zinc finger protein 280C OS=Homo sapiens C	3	104.3	100.9	96.7	97.1	102.6	96.9	0.944444444	0.525761
O00231	PSMD11	26S proteasome non-ATPase regulatory subu	23	106.6	102.1	100	97.1	104.35	98.55	0.944417825	0.525461
B2R823		cDNA, FLJ93703, highly similar to Homo sap	3	111.9	98.4	101.2	97.4	105.15	99.3	0.944365193	0.524869
Q6IAX6	PAPSS1	3'-phosphoadenosine 5'-phosphosulfate syntha	9	101.8	99.5	94	96.1	100.65	95.05	0.944361649	0.524829
Q9UBF2	COPG2	Coatomer subunit gamma-2 OS=Homo sapier	12	105.9	100.7	95.5	99.6	103.3	97.55	0.944336883	0.524551
Q59GW7		Replication factor C 5 isoform 1 variant (Frag	9	106.2	102.1	98.5	98.2	104.15	98.35	0.94431109	0.524261
P46736	BRCC3	Lys-63-specific deubiquitinase BRCC36 OS=	2	109.7	96.7	103.2	91.7	103.2	97.45	0.944282946	0.523945
O96007	MOCS2	Molybdopterin synthase catalytic subunit OS=	2	101.9	93.7	90.5	94.2	97.8	92.35	0.944274029	0.523844
Q12899	TRIM26	Tripartite motif-containing protein 26 OS=Ho	3	108.2	101.7	103.4	94.8	104.95	99.1	0.944259171	0.523678
Q9Y6I4	USP3	Ubiquitin carboxyl-terminal hydrolase 3 OS=	2	102.4	100.3	95.7	95.7	101.35	95.7	0.94425259	0.523604
Q9NTX5	ECHDC1	Ethylmalonyl-CoA decarboxylase OS=Homo	11	102.2	98.7	94.4	95.3	100.45	94.85	0.944250871	0.523584
O75817	POP7	Ribonuclease P protein subunit p20 OS=Hom	3	102.9	101.3	96.9	95.9	102.1	96.4	0.94417238	0.522703
P28072	PSMB6	Proteasome subunit beta type-6 OS=Homo sa	9	103.7	104	98	98.1	103.85	98.05	0.944150217	0.522454
Q9BUR4	WRAP53	Telomerase Cajal body protein 1 OS=Homo s	5	102.6	103.3	99.8	94.6	102.95	97.2	0.944147644	0.522425
Q8IYA2	CCDC144CP	Putative coiled-coil domain-containing protein	1	123.9	90.9	102.8	100	107.4	101.4	0.944134078	0.522273
Q96HJ9	C7orf55	UPF0562 protein C7orf55 OS=Homo sapiens	3	101.9	103.6	98.9	95.1	102.75	97	0.944038929	0.521206
A8K7W3		cDNA FLJ75780, highly similar to Homo sap	8	106.1	100.8	96.2	99.1	103.45	97.65	0.943934268	0.520034
Q8TE01	derp12	DERP12 (Dermal papilla derived protein 12)	13	112.4	105	105.6	99.6	108.7	102.6	0.943882245	0.519451
Q9Y678	COPG1	Coatomer subunit gamma-1 OS=Homo sapier	23	102.8	101.9	95.3	97.9	102.35	96.6	0.943820225	0.518758
O95801	TTC4	Tetratricopeptide repeat protein 4 OS=Homo s	3	101.7	103	92.2	101	102.35	96.6	0.943820225	0.518758
Q13867	BLMH	Bleomycin hydrolase OS=Homo sapiens GN=	6	105.9	102.3	98	98.5	104.1	98.25	0.943804035	0.518576
Q9BXP2	SLC12A9	Solute carrier family 12 member 9 OS=Homo	5	107.5	98.8	94.5	100.2	103.15	97.35	0.943771207	0.518209
A0A024R8U4	WDR45L	WDR45-like, isoform CRA_c OS=Homo sapi	2	105	101.3	97	97.7	103.15	97.35	0.943771207	0.518209
Q9UPU5	USP24	Ubiquitin carboxyl-terminal hydrolase 24 OS=	7	101.1	103.4	95.5	97.5	102.25	96.5	0.943765281	0.518143
Q8NHS3	MFSD8	Major facilitator superfamily domain-containi	1	104.3	94.7	100.9	86.9	99.5	93.9	0.943718593	0.517622
Q9H4A6	GOLPH3	Golgi phosphoprotein 3 OS=Homo sapiens G	4	103.1	102.8	99.2	95.1	102.95	97.15	0.943661972	0.516989
Q14764	MVP	Major vault protein OS=Homo sapiens GN=M	17	100.8	96.2	92.6	93.3	98.5	92.95	0.943654822	0.516909
Q8NDZ4	C3orf58	Deleted in autism protein 1 OS=Homo sapien	6	109.9	99.4	96.9	100.6	104.65	98.75	0.943621596	0.516538
Q8NEW0	SLC30A7	Zinc transporter 7 OS=Homo sapiens GN=SL	3	109	102	98.3	100.8	105.5	99.55	0.943601896	0.516318
Q6UXT9	ABHD15	Protein ABHD15 OS=Homo sapiens GN=AB	2	97.4	109.9	103.9	91.7	103.65	97.8	0.943560058	0.515851
A0A024R4R9	LENG8	Leukocyte receptor cluster (LRC) member 8,	8	100.7	101.2	95.2	95.3	100.95	95.25	0.943536404	0.515588
P62263	RPS14	40S ribosomal protein S14 OS=Homo sapiens	8	105.2	101.8	97.4	97.9	103.5	97.65	0.943478261	0.51494
A3R0T8	HIST1H1E	Histone 1, H1e OS=Homo sapiens GN=HIST	4	97.4	102.5	96	92.6	99.95	94.3	0.943471736	0.514867
Q5PSV4	BRMS1L	Breast cancer metastasis-suppressor 1-like pro	1	115.7	98.3	97.9	104	107	100.95	0.943457944	0.514713
Q13546	RIPK1	Receptor-interacting serine/threonine-protein	3	104.9	98.4	91	100.8	101.65	95.9	0.94343335	0.514439
A0A024R5Z9	PKM2	Pyruvate kinase OS=Homo sapiens GN=PKM	1	97.8	110.7	99.1	97.6	104.25	98.35	0.943405276	0.514126
B4DU42		cDNA FLJ56153, highly similar to Homo sap	21	104.3	102.3	98.6	96.3	103.3	97.45	0.943368829	0.513721
Q5T3J3	LRIF1	Ligand-dependent nuclear receptor-interacting	2	99.1	96.7	94.2	90.5	97.9	92.35	0.943309499	0.51306
Q01844	EWSR1	RNA-binding protein EWS OS=Homo sapien	1	108.4	96.2	95.2	97.8	102.3	96.5	0.943304008	0.512999
B2R7X3		cDNA, FLJ93645, highly similar to Homo sap	6	102	102.4	97	95.8	102.2	96.4	0.943248532	0.512382
Q5SR56	MFSD14B	Hippocampus abundant transcript-like protein	1	109.9	95.9	95.8	98.3	102.9	97.05	0.943148688	0.511272
K7EJ96	ROGDI	Protein rogdi homolog (Fragment) OS=Homo	1	103.2	96.8	90.1	98.5	100	94.3	0.943	0.509622
P62854	RPS26	40S ribosomal protein S26 OS=Homo sapiens	5	103.7	106.8	100.9	97.6	105.25	99.25	0.942992874	0.509543
Q5U077	LDHB	L-lactate dehydrogenase OS=Homo sapiens C	15	98	101.9	94.4	94.1	99.95	94.25	0.942971486	0.509305
Q8WXH0	SYNE2	Nesprin-2 OS=Homo sapiens GN=SYNE2 PE	16	104.7	102.2	98.1	97	103.45	97.55	0.942967617	0.509262
F8VXC8	SMARCC2	SWI/SNF complex subunit SMARCC2 OS=H	10	103.3	101.3	96.2	96.7	102.3	96.45	0.942815249	0.507575
E7EXA6	CHTF18	Chromosome transmission fidelity protein 18	3	103.3	104.7	91.5	104.6	104	98.05	0.942788462	0.507278
A3KMH1	VWA8	von Willebrand factor A domain-containing p	12	106.9	101.1	99.8	96.3	104	98.05	0.942788462	0.507278
Q9NX61	TMEM161A	Transmembrane protein 161A OS=Homo sapi	3	99.4	101.6	96.6	92.9	100.5	94.75	0.94278607	0.507252
A8K4A1		cDNA FLJ76790 OS=Homo sapiens PE=2 SV	8	106.1	101.8	96.6	99.4	103.95	98	0.942760943	0.506974
F5GWE5	PITPNA	Phosphatidylinositol transfer protein alpha iso	4	105.8	96.8	96.2	94.8	101.3	95.5	0.942744324	0.50679
Q8N3S0	DKFZp451C20	GrpE protein homolog (Fragment) OS=Homo	2	99.7	104.6	99.3	93.3	102.15	96.3	0.942731278	0.506645
P61081	UBE2M	NEDD8-conjugating enzyme Ubc12 OS=Hon	5	103.8	102	102.1	91.9	102.9	97	0.942662779	0.505888
Q8TE73	DNAH5	Dynein heavy chain 5, axonemal OS=Homo s	1	101.8	98.7	97.1	91.9	100.25	94.5	0.942643392	0.505674
P17987	TCP1	T-complex protein 1 subunit alpha OS=Homo	29	105.3	103.7	99.1	97.9	104.5	98.5	0.942583732	0.505015
Q8N3Y0	ITPK1	ITPK1 protein (Fragment) OS=Homo sapiens	1	97.9	98.9	93.2	92.3	98.4	92.75	0.942581301	0.504988
Q8N6M0	OTUD6B	OTU domain-containing protein 6B OS=Hom	3	106.7	104	99.8	98.8	105.35	99.3	0.942572378	0.504889
Q9UBB5	MBD2	Methyl-CpG-binding domain protein 2 OS=H	3	109.9	98.7	99.3	97.3	104.3	98.3	0.942473634	0.5038
Q53FX5		Lin-7 homolog C variant (Fragment) OS=Hor	7	108.3	102	97.4	100.8	105.15	99.1	0.942463148	0.503684

Q9NT99	LRRC4B	Leucine-rich repeat-containing protein 4B OS	1	108.1	96.5	98.5	94.3	102.3	96.4	0.942326491	0.502178
A8K168		Malic enzyme OS=Homo sapiens PE=2 SV=1	5	99.9	104.6	94	98.7	102.25	96.35	0.942298289	0.501867
A0A0C4DGB(	SCOC	Short coiled-coil protein OS=Homo sapiens G	1	91.5	104.1	87.4	96.9	97.8	92.15	0.942229039	0.501105
H7C1M2	SON	Protein SON (Fragment) OS=Homo sapiens G	1	109.4	98.2	93.4	102.2	103.8	97.8	0.942196532	0.500748
Q9UJF2	RASAL2	Ras GTPase-activating protein nGAP OS=Ho	1	105.5	98.6	94.2	98.1	102.05	96.15	0.942185203	0.500623
P31939	ATIC	Bifunctional purine biosynthesis protein PUR	28	103.5	100.4	96.9	95.2	101.95	96.05	0.942128494	0.499999
Q9HC03		Dolichyl-phosphate beta-glucosyltransferase (	5	112.7	94.6	95.5	99.8	103.65	97.65	0.94211288	0.499828
Q9Y303	AMDHD2	N-acetylglucosamine-6-phosphate deacetylase	1	106.6	95.4	99.6	90.7	101	95.15	0.942079208	0.499458
Q9H330	TMEM245	Transmembrane protein 245 OS=Homo sapien	1	110.8	98	99.7	97	104.4	98.35	0.942049808	0.499135
P29374	ARID4A	AT-rich interactive domain-containing protein	3	108	97.2	95.8	97.5	102.6	96.65	0.942007797	0.498674
P30047	GCHFR	GTP cyclohydrolase 1 feedback regulatory pro	1	101.5	101.9	94.3	97.3	101.7	95.8	0.941986234	0.498437
A0A024R2W7	MAPKAPK3	Mitogen-activated protein kinase-activated pro	3	103.9	101.2	96.8	96.4	102.55	96.6	0.941979522	0.498364
A8K7I0	CLK2	CDC-like kinase 2, isoform CRA_a OS=Hom	1	99.3	97.1	87.6	97.4	98.2	92.5	0.941955193	0.498097
Q9UJU6	DBNL	Drebrin-like protein OS=Homo sapiens GN=I	9	104	102.7	96.6	98.1	103.35	97.35	0.941944848	0.497983
Q9BRT9	GINS4	DNA replication complex GINS protein SLD5	2	97	107.9	95.1	97.9	102.45	96.5	0.941922889	0.497742
B3KM35		cDNA FLJ10144 fis, clone HEMBA1003286,	1	104.2	100.6	105.4	87.5	102.4	96.45	0.941894531	0.497431
Q15047	SETDB1	Histone-lysine N-methyltransferase SETDB1	2	107.1	97.7	95.1	97.8	102.4	96.45	0.941894531	0.497431
F8WAL6	ABI2	Abl interactor 2 OS=Homo sapiens GN=ABI2	1	107.6	105.7	114.1	86.8	106.65	100.45	0.941865917	0.497118
Q8IUC4	RHPN2	Rhophilin-2 OS=Homo sapiens GN=RHPN2	1	108.2	99.8	96.5	99.4	104	97.95	0.941826923	0.496691
Q9UIU6	SIX4	Homeobox protein SIX4 OS=Homo sapiens G	2	104	98.6	94.1	96.7	101.3	95.4	0.941757157	0.495926
Q96KP4	CNDP2	Cytosolic non-specific dipeptidase OS=Homo	8	101	101.5	93.7	97	101.25	95.35	0.941728395	0.495612
P50748	KNTC1	Kinetochore-associated protein 1 OS=Homo s	3	105.2	97.2	90.7	99.9	101.2	95.3	0.941699605	0.495297
Q9HBM0	VEZT	Vezatin OS=Homo sapiens GN=VEZT PE=1	4	104.4	104.7	99.1	97.8	104.55	98.45	0.941654711	0.494805
A5YM53	ITGAV	ITGAV protein OS=Homo sapiens GN=ITGA	46	105.5	103.3	98.2	98.4	104.4	98.3	0.941570881	0.493889
Q969T9	WBP2	WW domain-binding protein 2 OS=Homo sap	2	104.8	97.1	100	90.1	100.95	95.05	0.941555225	0.493718
P62253	UBE2G1	Ubiquitin-conjugating enzyme E2 G1 OS=Ho	2	103.4	100.2	90.3	101.4	101.8	95.85	0.941552063	0.493683
O75717	WDHD1	WD repeat and HMG-box DNA-binding prote	15	101.9	103.2	95.2	97.9	102.55	96.55	0.941491955	0.493027
P10644	PRKAR1A	cAMP-dependent protein kinase type I-alpha r	13	104.4	100.5	96.9	96	102.45	96.45	0.941434846	0.492404
Q96MG7	NSMCE3	Non-structural maintenance of chromosomes e	3	106	92	93.4	93	99	93.2	0.941414141	0.492178
Q7LBC6	KDM3B	Lysine-specific demethylase 3B OS=Homo sa	6	109.9	101.7	97.7	101.5	105.8	99.6	0.941398866	0.492012
A8K4T8		Condensin complex subunit 2 OS=Homo sapi	7	102.1	102.5	96.2	96.4	102.3	96.3	0.941348974	0.491468
A0A087WW4	SH3GLB1	Endophilin-B1 OS=Homo sapiens GN=SH3G	6	101.1	101.7	97.2	93.7	101.4	95.45	0.941321499	0.491168
P50991	CCT4	T-complex protein 1 subunit delta OS=Homo	34	104.2	105.3	99.2	98	104.75	98.6	0.941288783	0.490812
Q8TBK6	ZCCHC10	Zinc finger CCHC domain-containing protein	1	107.7	100	91.3	104.2	103.85	97.75	0.941261435	0.490514
D3DSM6	SLC19A1	Solute carrier family 19 (Folate transporter), r	4	107.4	98.4	95.8	97.9	102.9	96.85	0.941205053	0.4899
A0A024R1A4	UBE2L3	Ubiquitin-conjugating enzyme E2L 3, isoform	7	101.9	103.9	96.3	97.4	102.9	96.85	0.941205053	0.4899
Q9BW72	HIGD2A	HIG1 domain family member 2A, mitochondr	1	110.4	95	97.6	95.7	102.7	96.65	0.941090555	0.488655
A0A0G2JH68	DIAPH1	Protein diaphanous homolog 1 OS=Homo sap	15	105.5	99.9	95.3	98	102.7	96.65	0.941090555	0.488655
A1L390	PLEKHG3	Pleckstrin homology domain-containing famil	1	102.1	104.9	98.1	96.7	103.5	97.4	0.941062802	0.488354
P61024	CKS1B	Cyclin-dependent kinases regulatory subunit 1	2	101.7	103.6	99.7	93.5	102.65	96.6	0.941061861	0.488343
B2R7E8		cDNA, FLJ93412, highly similar to Homo sap	5	102.4	102.5	95.8	97	102.45	96.4	0.940946803	0.487094
A8K4T6		cDNA FLJ76282, highly similar to Homo sap	12	100.6	102.6	94.2	97	101.6	95.6	0.940944882	0.487074
A0A024R4V5	BRD1	Bromodomain containing 1, isoform CRA_a (	3	102.6	97.2	96.4	91.6	99.9	94	0.940940941	0.48703
O75832	PSMD10	26S proteasome non-ATPase regulatory subu	5	104.3	102.2	99.8	94.5	103.25	97.15	0.940920097	0.486805
D3DSY9	FNTA	Farnesyltransferase, CAAX box, alpha, isofor	6	103.1	98.3	95.8	93.7	100.7	94.75	0.940913605	0.486734
M0QY77	KXD1	KxDL motif-containing protein 1 OS=Homo s	1	109	93.9	94.5	96.4	101.45	95.45	0.940857565	0.486126
P35580	MYH10	Myosin-10 OS=Homo sapiens GN=MYH10 F	39	104.3	101.8	96.5	97.4	103.05	96.95	0.940805434	0.485561
Q96CP2	FLYWCH2	FLYWCH family member 2 OS=Homo sapien	3	97.9	108.1	95.9	97.9	103	96.9	0.940776699	0.48525
Q9UNZ2	NSFL1C	NSFL1 cofactor p47 OS=Homo sapiens GN=	1	93.1	90.8	83.1	89.9	91.95	86.5	0.940728657	0.48473
A6NDU8	C5orf51	UPF0600 protein C5orf51 OS=Homo sapiens	1	101.4	98.9	85.8	102.6	100.15	94.2	0.940589116	0.48322
A0A140VKAS		Testis secretory sperm-binding protein Li 236	3	102.7	105.9	94.1	102.1	104.3	98.1	0.940556088	0.482864
P07199	CENPB	Major centromere autoantigen B OS=Homo sa	3	104.3	99.2	92.5	98.9	101.75	95.7	0.940540541	0.482696
Q9UFF9	CNOT8	CCR4-NOT transcription complex subunit 8 (	2	101.8	96.6	92.5	94.1	99.2	93.3	0.940524194	0.482519
Q9NZJ4	SACS	Sacsin OS=Homo sapiens GN=SACS PE=1 S	5	105.1	99.8	96.9	95.8	102.45	96.35	0.94045876	0.481813
O60783	MRPS14	28S ribosomal protein S14, mitochondrial OS	2	108.7	102.9	102.5	96.5	105.8	99.5	0.940453686	0.481758
Q969J3	BORCS5	BLOC-1-related complex subunit 5 OS=Hom	1	103.5	101.2	92.9	99.6	102.35	96.25	0.940400586	0.481185
A0A024R4Z1	hCG_2042749	HCG2042749, isoform CRA_b OS=Homo sap	1	110.6	94	93.9	98.5	102.3	96.2	0.940371457	0.480871
V9HWK2	HEL114	Epididymis luminal protein 114 OS=Homo sa	62	100.8	102.1	95.5	95.3	101.45	95.4	0.940364712	0.480798
P20073	ANXA7	Annexin A7 OS=Homo sapiens GN=ANXA7	11	101.5	99.6	95	94.1	100.55	94.55	0.940328195	0.480404
Q96AA3	RFT1	Protein RFT1 homolog OS=Homo sapiens GN	1	104	102.1	95.9	97.9	103.05	96.9	0.940320233	0.480319
P63172	DYNLT1	Dynein light chain Tctex-type 1 OS=Homo sa	2	104.4	98.3	94.9	95.7	101.35	95.3	0.940305871	0.480164
Q08AG7	MZT1	Mitotic-spindle organizing protein 1 OS=Hom	3	103.9	105.5	94.5	102.4	104.7	98.45	0.940305635	0.480161
H0YNJ6	GMPR2	GMP reductase OS=Homo sapiens GN=GMP	2	101.9	104.1	97.8	95.9	103	96.85	0.940291262	0.480007
Q14149	MORC3	MORC family CW-type zinc finger protein 3	2	98.4	78.9	79.7	87	88.65	83.35	0.940214326	0.479178
Q92930	RAB8B	Ras-related protein Rab-8B OS=Homo sapien	1	111.2	107.6	102.6	103.1	109.4	102.85	0.940127971	0.478249
A0A0S2Z5E9	CWF19L1	CWF19-like 1 cell cycle control isoform 1 (Fr	3	104.3	99.4	94	97.5	101.85	95.75	0.940108002	0.478034
O76095	JTB	Protein JTB OS=Homo sapiens GN=JTB PE=	1	102.3	101.4	97.9	93.6	101.85	95.75	0.940108002	0.478034
A0A024R3L9	VPS26B	Vacuolar protein sorting 26 homolog B (Yeas	3	102.3	104.6	97.7	96.8	103.45	97.25	0.940067666	0.4776
Q53Y51	DDT	D-dopachrome tautomerase OS=Homo sapien	6	94.4	105.7	90.5	97.6	100.05	94.05	0.940029985	0.477196
A0A0A1HAV		H.sapiens ras-related Hrab3B protein OS=Hor	3	107.7	95.7	97.6	93.6	101.7	95.6	0.940019666	0.477085
Q8NEY8	PPHLN1	Periphilin-1 OS=Homo sapiens GN=PPHLN1	1	103.3	108.2	98	100.8	105.75	99.4	0.939952719	0.476366
Q9H3U1	UNC45A	Protein unc-45 homolog A OS=Homo sapiens	19	106.4	100.1	95.7	98.4	103.25	97.05	0.939951574	0.476354
P54725	RAD23A	UV excision repair protein RAD23 homolog A	6	105.7	102.3	96.9	98.6	104	97.75	0.939903846	0.475842
P15408	FOSL2	Fos-related antigen 2 OS=Homo sapiens GN=	2	104.7	101.6	91.8	102.1	103.15	96.95	0.939893359	0.475729
A0A024R9K2	BAHD1	Bromo adjacent homology domain containing	1	103.7	97.5	96.9	92.2	100.6	94.55	0.939860835	0.47538
A0A024R5Q8	CTDSPL2	CTD (Carboxy-terminal domain, RNA polym	7	104.5	100	98	94.2	102.25	96.1	0.939853301	0.4753
O15116	LSM1	U6 snRNA-associated Sm-like protein LSm1	1	104	90.5	89.5	93.3	97.25	91.4	0.939845758	0.475219
P23378	GLDC	Glycine dehydrogenase (decarboxylating), mi	17	102.3	102.1	95.4	96.7	102.2	96.05	0.939823875	0.474984
A0A024R2G1	CRELD1	Cysteine-rich with EGF-like domains 1, isofor	1	103.2	101.2	96.8	95.3	102.2	96.05	0.939823875	0.474984
Q59HE8		Putative uncharacterized protein (Fragment) C	1	108.5	97.5	100.3	93.3	103	96.8	0.939805825	0.474791
G5E9G6	TRIP12	E3 ubiquitin-protein ligase TRIP12 OS=Hom	1	107.2	101.5	107.6	88.5	104.35	98.05	0.939626258	0.472869
Q9NQ48	LZTFL1	Leucine zipper transcription factor-like protei	1	104.8	100.5	92.3	100.6	102.65	96.45	0.939600585	0.472595
Q71RS6	SLC24A5	Sodium/potassium/calcium exchanger 5 OS=I	1	110.9	99.1	100.2	97.1	105	98.65	0.93952381	0.471774
B2RAX1		cDNA, FLJ95169 OS=Homo sapiens PE=2 S	1	98.5	115.7	104.7	96.5	107.1	100.6	0.939309057	0.469484
O14874	BCKDK	[3-methyl-2-oxobutanoate dehydrogenase [lip	5	101.9	100.7	94.1	96.2	101.3	95.15	0.93928924	0.469272
O43251	RBFOX2	RNA binding protein fox-1 homolog 2 OS=H	3	109	105.1	103.7	97.4	107.05	100.55	0.93928071	0.469181
A0A024R7I5	TRMT1	TRM1 tRNA methyltransferase 1 homolog (S	9	102.6	104.6	98.5	96.1	103.6	97.3	0.939189189	0.468207
A0A024R5J6	CHCHD8	Coiled-coil-helix-coiled-coil-helix domain co	5	100.2	101.9	95.2	94.6	101.05	94.9	0.93913904	0.467674
B1AKJ5	NRDC	Nardilysin OS=Homo sapiens GN=NRDC PE	12	106.2	100.6	96.9	97.3	103.4	97.1	0.939071567	0.466957
Q6UX07	DHRS13	Dehydrogenase/reductase SDR family membe	1	105.7	105.9	95.6	103.1	105.8	99.35	0.939035917	0.466578
A8K2H6		Lipase OS=Homo sapiens PE=2 SV=1	4	103.3	101.7	98.7	93.8	102.5	96.25	0.93902439	0.466456
Q9BQA1	WDR77	Methylosome protein 50 OS=Homo sapiens G	5	105.7	100.9	98.1	95.9	103.3	97	0.939012585	0.46633
Q8WUW2	AGPAT2	1-acyl-sn-glycerol-3-phosphate acyltransferas	1	99.9	104.9	94.3	98	102.4	96.15	0.938964844	0.465824
Q9H9V9	JMJD4	JmjC domain-containing protein 4 OS=Homo	1	107.7	103.6	99.4	99	105.65	99.2	0.938949361	0.465659
Q7Z783		SPRY2 protein OS=Homo sapiens PE=1 SV=	2	101.2	106.7	96.7	98.5	103.95	97.6	0.938912939	0.465273
A0A0S2Z428	KRT6A	HCG2039812, isoform CRA_b (Fragment) O	1	105.1	122.3	111.7	101.8	113.7	106.75	0.93887423	0.464863
Q9H993	ARMT1	Protein-glutamate O-methyltransferase OS=H	4	99.7	96.6	93	91.3	98.15	92.15	0.938869078	0.464808
P07437	TUBB	Tubulin beta chain OS=Homo sapiens GN=TU	4	96.6	99.5	90.5	93.6	98.05	92.05	0.938806731	0.464147
Q13573	SNW1	SNW domain-containing protein 1 OS=Homo	15	104.6	102.9	99.1	95.7	103.75	97.4	0.938795181	0.464025
A6NDG6	PGP	Glycerol-3-phosphate phosphatase OS=Homo	5	99.7	99.4	94.9	92	99.55	93.45	0.938724259	0.463274
A0A0S2Z4A5	MCM7	DNA helicase (Fragment) OS=Homo sapiens	27	103.5	103.7	97.2	97.3	103.6	97.25	0.938706564	0.463087
Q9BZW4	TM6SF2	Transmembrane 6 superfamily member 2 OS=	1	103.6	105.2	101	95	104.4	98	0.938697318	0.462989
O60503	ADCY9	Adenylate cyclase type 9 OS=Homo sapiens (	2	108.5	98.6	92.5	101.9	103.55	97.2	0.938676968	0.462774
Q9Y5B6	PAXBP1	PAX3- and PAX7-binding protein 1 OS=Hom	11	105.7	104.5	101.6	95.7	105.1	98.65	0.938629876	0.462276
A8K724		cDNA FLJ76899, highly similar to Homo sap	4	102.8	102.3	96.6	95.9	102.55	96.25	0.938566553	0.461607
Q9UNX3	RPL26L1	60S ribosomal protein L26-like 1 OS=Homo s	2	109.7	100.1	99.9	97	104.9	98.45	0.938512869	0.46104
Q6IA86	ELP2	Elongator complex protein 2 OS=Homo sapie	1	109.9	101.5	107.9	90.5	105.7	99.2	0.938505203	0.46096
Q15276	RABEP1	Rab GTPase-binding effector protein 1 OS=H	6	101.4	101.8	91.8	98.9	101.6	95.35	0.938484252	0.460738
Q8IXB1	DNAJC10	DnaJ homolog subfamily C member 10 OS=H	11	108.3	103	100.9	97.4	105.65	99.15	0.9384761	0.460652
A0A087WT23	UBXN8	UBX domain-containing protein 8 OS=Homo	1	108.2	90	86.9	99.1	99.1	93	0.938446014	0.460334
O60524	NEMF	Nuclear export mediator factor NEMF OS=Ho	4	95.6	97.1	88.8	92	96.35	90.4	0.938245978	0.458227
Q9UNI6	DUSP12	Dual specificity protein phosphatase 12 OS=H	5	104.7	102.4	97	97.3	103.55	97.15	0.938194109	0.457682
A0A140VK94	RANBP1	RAN binding protein 1, isoform CRA_g OS=	7	100.3	103.5	96.3	94.9	101.9	95.6	0.938174681	0.457477
A0A024R3M1	THYN1	Thymocyte nuclear protein 1, isoform CRA_a	2	103.5	110	100.8	99.5	106.75	100.15	0.938173302	0.457463
O15084	ANKRD28	Serine/threonine-protein phosphatase 6 regula	3	108.3	92.2	87.3	100.8	100.25	94.05	0.938154613	0.457267
O14523	C2CD2L	C2 domain-containing protein 2-like OS=Hon	1	115.4	102.8	100.1	104.6	109.1	102.35	0.938130156	0.457009
Q96AG3	SLC25A46	Solute carrier family 25 member 46 OS=Hom	2	116.1	90.6	94.2	99.7	103.35	96.95	0.938074504	0.456425
A0A0A0MTN	CUL2	Cullin-2 OS=Homo sapiens GN=CUL2 PE=1	14	108.2	101.7	98.4	98.5	104.95	98.45	0.938065746	0.456333
O75436	VPS26A	Vacuolar protein sorting-associated protein 26	10	102.9	107	100.2	96.7	104.95	98.45	0.938065746	0.456333
P09110	ACAA1	3-ketoacyl-CoA thiolase, peroxisomal OS=Ho	14	104.1	100.8	93.6	98.6	102.45	96.1	0.938018546	0.455838
Q92791	P3H4	Synaptonemal complex protein SC65 OS=Ho	5	104.1	99.1	94.2	96.4	101.6	95.3	0.937992126	0.455561
O14561	NDUFAB1	Acyl carrier protein, mitochondrial OS=Homo	4	103.3	101.4	96.2	95.8	102.35	96	0.937957987	0.455202

Q9Y520	PRRC2C	Protein PRRC2C OS=Homo sapiens GN=PRI	23	106.1	101.7	96	98.9	103.9	97.45	0.937921078	0.454815
Q9UFN0	NIPSNAP3A	Protein NipSnap homolog 3A OS=Homo sapi	4	104.5	98.4	93.3	97	101.45	95.15	0.937900444	0.454599
Q643R3	LPCAT4	Lysophospholipid acyltransferase LPCAT4 O	3	111.3	99.6	99.6	98.2	105.45	98.9	0.937885254	0.454439
A0A024R830	IMPA1	Inositol(Myo)-1(Or 4)-monophosphatase 1, is	6	103.5	99	95.1	94.8	101.25	94.95	0.937777778	0.453314
B7Z2E3		cDNA FLJ55917, highly similar to Cytochron	1	113.6	98.5	98.2	100.7	106.05	99.45	0.937765205	0.453183
Q9UGN5	PARP2	Poly [ADP-ribose] polymerase 2 OS=Homo s	4	102.7	99.5	97	92.6	101.1	94.8	0.93768546	0.452349
B2RCM6		cDNA, FLJ96161 OS=Homo sapiens PE=2 S'	1	97.2	111.4	97.4	98.2	104.3	97.8	0.93767977	0.452289
A0A024RCN6	VARS	Valyl-tRNA synthetase, isoform CRA_a OS=	20	106.9	102.9	98.2	98.5	104.9	98.35	0.937559581	0.451034
O14957	UQCR11	Cytochrome b-c1 complex subunit 10 OS=Ho	1	105.9	94.2	95.2	92.4	100.05	93.8	0.937531234	0.450738
B2R6J3		cDNA, FLJ92974, highly similar to Homo sap	5	104	102.4	96.8	96.7	103.2	96.75	0.9375	0.450412
A8K4V6		cDNA FLJ76394, highly similar to Homo sap	1	100.7	99.2	89.2	98.2	99.95	93.7	0.937468734	0.450086
B1AHB0	MCM5	DNA helicase OS=Homo sapiens GN=MCM5	32	104.4	101.8	98.2	95.1	103.1	96.65	0.937439379	0.449781
Q96IJ6	GMPPA	Mannose-1-phosphate guanyltransferase alpha	4	103.3	101	95.6	95.9	102.15	95.75	0.937347039	0.448819
Q8TDB6	DTX3L	E3 ubiquitin-protein ligase DTX3L OS=Hom	4	109.2	96.6	95.2	97.7	102.9	96.45	0.937317784	0.448514
O95758	PTBP3	Polypyrimidine tract-binding protein 3 OS=H	3	105.2	103.7	100.7	95.1	104.45	97.9	0.93729057	0.448231
Q15031	LARS2	Probable leucine--tRNA ligase, mitochondrial	12	107	100.3	97.7	96.6	103.65	97.15	0.937288953	0.448214
Q9UPT8	ZC3H4	Zinc finger CCCH domain-containing protein	5	107.5	99.7	95.7	98.5	103.6	97.1	0.937258687	0.447899
Q96HY7	DHTKD1	Probable 2-oxoglutarate dehydrogenase E1 co	1	103.4	100.6	92.5	98.7	102	95.6	0.937254902	0.44786
Q86US8	SMG6	Telomerase-binding protein EST1A OS=Hom	2	101.2	101.2	92.8	96.9	101.2	94.85	0.937252964	0.44784
Q3T8J9	GON4L	GON-4-like protein OS=Homo sapiens GN=C	1	102.2	95.4	89.9	95.3	98.8	92.6	0.937246964	0.447778
Q9NYF3	FAM53C	Protein FAM53C OS=Homo sapiens GN=FA	1	109.9	92.4	93.4	96.2	101.15	94.8	0.937221948	0.447517
Q8IVM0	CCDC50	Coiled-coil domain-containing protein 50 OS=	3	105.4	103.1	95.3	100.1	104.25	97.7	0.937170264	0.44698
Q5EBL4	RILPL1	RILP-like protein 1 OS=Homo sapiens GN=R	1	116.3	93.6	98.6	98.1	104.95	98.35	0.937112911	0.446385
F8W0Q9	PPHLN1	Periphilin-1 OS=Homo sapiens GN=PPHLN1	2	101.5	100.1	90.2	98.7	100.8	94.45	0.937003968	0.445255
Q01581	HMGCS1	Hydroxymethylglutaryl-CoA synthase, cytopl	6	103.9	97.6	88.9	99.9	100.75	94.4	0.936972705	0.44493
Q9NZZ3	CHMP5	Charged multivesicular body protein 5 OS=H	5	97.6	100.7	91.9	93.9	99.15	92.9	0.936964196	0.444843
Q8WUQ7	CACTIN	Cactin OS=Homo sapiens GN=CACTIN PE=	3	106.8	96.1	96.2	93.9	101.45	95.05	0.936914736	0.44433
Q92576	PHF3	PHD finger protein 3 OS=Homo sapiens GN=	11	106.7	102.5	98.6	97.4	104.6	98	0.936902486	0.444203
B4DP48		cDNA FLJ61147 OS=Homo sapiens PE=2 SV	2	103.8	103.8	99.1	95.4	103.8	97.25	0.936897881	0.444156
A0A024R0J7	hCG_1995540	HCG1995540, isoform CRA_a OS=Homo sap	1	101.8	105.7	107.6	86.8	103.75	97.2	0.93686747	0.443841
A0A024QZY1	JTV1	JTV1 gene, isoform CRA_a OS=Homo sapien	5	102.5	104.7	97.7	96.4	103.6	97.05	0.936776062	0.442895
Q68D85	NCR3LG1	Natural cytotoxicity triggering receptor 3 ligan	3	110.7	102.6	99.3	100.5	106.65	99.9	0.936708861	0.442201
Q9NYP7	ELOVL5	Elongation of very long chain fatty acids prote	2	108	98.9	94.7	99.1	103.45	96.9	0.936684389	0.441948
A0A024R9G4	FAM49B	Family with sequence similarity 49, member I	9	107.1	99.7	96	97.7	103.4	96.85	0.936653772	0.441632
Q8NDD1	C1orf131	Uncharacterized protein C1orf131 OS=Homo	3	107.6	102.3	97.7	98.9	104.95	98.3	0.936636494	0.441454
B2R749		cDNA, FLJ93285, highly similar to Homo sap	2	111.8	102.6	97.4	103.4	107.2	100.4	0.936567164	0.440739
Q6NTG0	SLC9A3R2	SLC9A3R2 protein (Fragment) OS=Homo sa	5	104.9	101.6	95.8	97.6	103.25	96.7	0.936561743	0.440683
B3KMQ6		cDNA FLJ12172 fis, clone MAMMA100068	5	104.7	100	96.4	95.3	102.35	95.85	0.936492428	0.439968
E5RHJ2	ASPH	Aspartyl/asparaginyl beta-hydroxylase OS=H	1	112.5	99.8	99	99.8	106.15	99.4	0.93641074	0.439127
Q07820	MCL1	Induced myeloid leukemia cell differentiation	1	104.1	101.8	89.2	103.6	102.95	96.4	0.936376882	0.438779
Q96S94	CCNL2	Cyclin-L2 OS=Homo sapiens GN=CCNL2 PI	4	103.6	99.1	93	96.8	101.35	94.9	0.936359151	0.438597
A0A024R497	ACSL3	Acyl-CoA synthetase long-chain family meml	24	105.2	105.1	98.8	98.1	105.15	98.45	0.936281503	0.437798
A0A024R323	ABHD6	Abhydrolase domain containing 6, isoform CI	1	108.7	103	101.8	96.4	105.85	99.1	0.936230515	0.437275
P51610	HCFC1	Host cell factor 1 OS=Homo sapiens GN=HC	1	109	107.3	107.3	95.2	108.15	101.25	0.936199723	0.436958
Q14642	INPP5A	Type I inositol 1,4,5-trisphosphate 5-phosphat	1	108.5	96.7	92.3	99.8	102.6	96.05	0.936159844	0.436549
Q16222	UAP1	UDP-N-acetylhexosamine pyrophosphorylase	7	108.3	99.9	97.7	97.2	104.1	97.45	0.936119116	0.436132
P41227	NAA10	N-alpha-acetyltransferase 10 OS=Homo sapie	11	108.6	101.1	100	96.3	104.85	98.15	0.936099189	0.435927
A0A024R529	DAK	Dihydroxyacetone kinase 2 homolog (Yeast),	4	99.3	99.4	93.8	92.2	99.35	93	0.93608455	0.435777
P49368	CCT3	T-complex protein 1 subunit gamma OS=Hon	3	106.2	104.8	97.5	100	105.5	98.75	0.936018957	0.435105
Q8IZ21	PHACTR4	Phosphatase and actin regulator 4 OS=Homo	4	105.7	101.9	96.2	98.1	103.8	97.15	0.935934489	0.43424
A0A096LNH6	DOCK1	Dedicator of cytokinesis protein 1 OS=Homo	1	105.4	98.9	97.6	93.6	102.15	95.6	0.93587861	0.433669
P23229	ITGA6	Integrin alpha-6 OS=Homo sapiens GN=ITGA	10	106.2	101.2	96.7	97.4	103.7	97.05	0.93587271	0.433609
A0A024RE02	PHGDHL1	Phosphoglycerate dehydrogenase like 1, isofo	2	106.4	100.9	94.1	99.9	103.65	97	0.935841775	0.433293
Q5JPH6	EARS2	Probable glutamate--tRNA ligase, mitochondr	5	106.8	98.8	94.6	97.8	102.8	96.2	0.935797665	0.432842
A0A024R3K2	RPUSD4	RNA pseudouridylate synthase domain contai	3	102.4	106.1	102.3	92.8	104.25	97.55	0.935731415	0.432165
D6W551	C2orf28	Chromosome 2 open reading frame 28, isofor	2	107	106	106	93.3	106.5	99.65	0.935680751	0.431649
C9JA93	TBC1D15	TBC1 domain family member 15 (Fragment)	1	102.8	97.7	90.3	97.3	100.25	93.8	0.935660848	0.431446
Q96JN8	NEURL4	Neuralized-like protein 4 OS=Homo sapiens (	3	109.4	100.4	99.1	97.2	104.9	98.15	0.935653003	0.431366
Q5T5U3	ARHGAP21	Rho GTPase-activating protein 21 OS=Homo	1	101.8	104.6	100.2	92.9	103.2	96.55	0.935562016	0.430439
P14324	FDPS	Farnesyl pyrophosphate synthase OS=Homo s	2	97.2	95	88	91.8	96.1	89.9	0.935483871	0.429643
B2RBD5		Tubulin beta chain OS=Homo sapiens PE=2 S	1	111.2	98	108.4	87.3	104.6	97.85	0.935468451	0.429486
Q9Y2D8	SSX2IP	Afadin- and alpha-actinin-binding protein OS	5	106.6	99.5	98.1	94.7	103.05	96.4	0.935468219	0.429484
Q9Y4R8	TELO2	Telomere length regulation protein TEL2 hom	5	101	102	96.9	93	101.5	94.95	0.93546798	0.429482
P23497	SP100	Nuclear autoantigen Sp-100 OS=Homo sapien	2	108.3	99.1	94.3	99.7	103.7	97	0.93539055	0.428695
P49281	SLC11A2	Natural resistance-associated macrophage pro	1	108.4	103.6	98.7	99.6	106	99.15	0.935377358	0.428561
A7E244	TBC1D10A	TBC1 domain family, member 10A OS=Hom	2	104.7	101.1	97.4	95.1	102.9	96.25	0.93537415	0.428528
Q16763	UBE2S	Ubiquitin-conjugating enzyme E2 S OS=Hom	5	106.8	103.5	96.9	99.8	105.15	98.35	0.93533048	0.428084
Q9NWX6	THG1L	Probable tRNA(His) guanylyltransferase OS=	3	106.3	102.2	96.1	98.9	104.25	97.5	0.935251799	0.427286
A0A024R781	CNOT4	CCR4-NOT transcription complex, subunit 4,	1	119.5	91.9	98.2	99.5	105.7	98.85	0.935193945	0.4267
P42166	TMPO	Lamina-associated polypeptide 2, isoform alp	16	103.1	102	96.5	95.3	102.55	95.9	0.935153584	0.426291
Q68CP9	ARID2	AT-rich interactive domain-containing protein	13	106.1	103.4	99.1	96.8	104.75	97.95	0.935083532	0.425581
Q9BUH6	C9orf142	Protein PAXX OS=Homo sapiens GN=C9orf	2	105.1	102.8	102	92.4	103.95	97.2	0.935064935	0.425393
P26639	TARS	Threonine--tRNA ligase, cytoplasmic OS=Ho	28	104.5	103.2	97.6	96.6	103.85	97.1	0.935002407	0.424761
Q8NCW6	GALNT11	Polypeptide N-acetylgalactosaminyltransferas	2	108	102.6	98.5	98.4	105.3	98.45	0.934947768	0.424209
H3BM91	COMMD4	COMM domain-containing protein 4 (Fragme	5	107.3	103.3	103.7	93.2	105.3	98.45	0.934947768	0.424209
Q9BTY7	HGH1	Protein HGH1 homolog OS=Homo sapiens G	6	103.4	102.5	92.5	100	102.95	96.25	0.934919864	0.423927
Q5TDH0	DDI2	Protein DDI1 homolog 2 OS=Homo sapiens (	8	99.9	106	96.6	95.9	102.95	96.25	0.934919864	0.423927
A8K486		Peptidyl-prolyl cis-trans isomerase OS=Homo	14	98.6	102.6	93.1	95	100.6	94.05	0.934890656	0.423631
Q6P9B9	INTS5	Integrator complex subunit 5 OS=Homo sapie	4	100.5	109.7	103.6	92.9	105.1	98.25	0.934823977	0.422959
P15924	DSP	Desmoplakin OS=Homo sapiens GN=DSP PE	3	109.6	105.2	104.4	96.4	107.4	100.4	0.934823091	0.42295
A0A0S2Z4R1	YARS	Tyrosine--tRNA ligase (Fragment) OS=Homo	23	103.5	100.5	96.4	94.3	102	95.35	0.934803922	0.422756
Q9H1A4	ANAPC1	Anaphase-promoting complex subunit 1 OS=I	10	104.5	101	96.8	95.3	102.75	96.05	0.934793187	0.422648
Q8NHP8	PLBD2	Putative phospholipase B-like 2 OS=Homo sa	7	104.6	103.3	99.8	94.5	103.95	97.15	0.934583935	0.420542
Q96L58	B3GALT6	Beta-1,3-galactosyltransferase 6 OS=Homo sa	1	104.9	98.3	95.6	94.3	101.6	94.95	0.934547244	0.420172
Q9NUI1	DECR2	Peroxisomal 2,4-dienoyl-CoA reductase OS=I	10	105.1	101.1	96.6	96.1	103.1	96.35	0.934529583	0.419995
Q8N122	RPTOR	Regulatory-associated protein of mTOR OS=I	1	108.8	105	101	98.8	106.9	99.9	0.934518241	0.419881
Q15813	TBCE	Tubulin-specific chaperone E OS=Homo sapi	5	103.6	102.5	98.8	93.8	103.05	96.3	0.934497817	0.419676
Q9NVP2	ASF1B	Histone chaperone ASF1B OS=Homo sapiens	2	102.8	86.4	84.6	92.2	94.6	88.4	0.934460888	0.419305
P63096	GNAI1	Guanine nucleotide-binding protein G(i) subu	3	108.9	100.1	97.5	97.8	104.5	97.65	0.934449761	0.419193
Q5VW38	GPR107	Protein GPR107 OS=Homo sapiens GN=GPR	2	107	97.4	91.8	99.2	102.2	95.5	0.93444227	0.419118
Q13363	CTBP1	C-terminal-binding protein 1 OS=Homo sapie	6	102.2	102.2	96.1	94.9	102.2	95.5	0.93444227	0.419118
P02461	COL3A1	Collagen alpha-1(III) chain OS=Homo sapien	7	106.6	100.5	93.8	99.7	103.55	96.75	0.934331241	0.418004
B7ZMF2	FANCI	Fanconi anemia, complementation group I OS	16	105.3	103.2	95.8	99	104.25	97.4	0.934292566	0.417617
P55263	ADK	Adenosine kinase OS=Homo sapiens GN=AD	4	101.5	100.9	93.7	95.4	101.2	94.55	0.934288538	0.417577
Q7L9B9	EEPD1	Endonuclease/exonuclease/phosphatase family	3	108.5	101.5	97.7	98.5	105	98.1	0.934285714	0.417548
Q9UNZ5	C19orf53	Leydig cell tumor 10 kDa protein homolog OS	3	98.8	106.2	98.9	92.6	102.5	95.75	0.934146341	0.416154
V9GYT7	PSIP1	PC4 and SFRS1-interacting protein (Fragmen	1	112.6	101.5	97.9	102.1	107.05	100	0.934142924	0.41612
B2RD51		Proteasome assembly chaperone 1 OS=Homo	5	105.6	100.9	92.8	100.1	103.25	96.45	0.934140436	0.416095
Q8TBM8	DNAJB14	DnaJ homolog subfamily B member 14 OS=H	4	114.9	93.1	98.3	96	104	97.15	0.934134615	0.416036
Q9H9T3	ELP3	Elongator complex protein 3 OS=Homo sapie	2	104.5	101.6	98.8	93.7	103.05	96.25	0.934012615	0.414817
Q9BU76	MMTAG2	Multiple myeloma tumor-associated protein 2	5	97.6	111.5	98.2	97.1	104.55	97.65	0.934002869	0.41472
Q8N543	OGFOD1	Prolyl 3-hydroxylase OGFOD1 OS=Homo sa	3	106.9	102.1	96.3	98.9	104.5	97.6	0.933971292	0.414405
Q14566	MCM6	DNA replication licensing factor MCM6 OS=	35	105	102.4	96.4	97.3	103.7	96.85	0.933944069	0.414134
O75907	DGAT1	Diacylglycerol O-acyltransferase 1 OS=Homo	2	107.3	93.9	95.5	92.4	100.6	93.95	0.93389662	0.413661
A4D105	RPA3	Replication protein A3, 14kDa OS=Homo sap	5	104.2	100	95.3	95.4	102.1	95.35	0.933888345	0.413578
E5KRP5	SPAST	Spastin OS=Homo sapiens GN=SPAST PE=3	1	110.4	99.8	96.6	99.7	105.1	98.15	0.933872502	0.413421
Q9NWZ8	GEMIN8	Gem-associated protein 8 OS=Homo sapiens (	2	112.7	102	92.9	107.6	107.35	100.25	0.933861202	0.413308
Q15554	TERF2	Telomeric repeat-binding factor 2 OS=Homo	1	113.5	90.3	90.7	99.6	101.9	95.15	0.933758587	0.412287
Q495P6	TLR8	Toll-like receptor 8 OS=Homo sapiens GN=T	1	112.2	99.1	102.5	94.8	105.65	98.65	0.933743493	0.412136
Q59G98		TIA1 protein variant (Fragment) OS=Homo sa	4	108.1	100.1	95.4	99	104.1	97.2	0.933717579	0.411879
O75600	GCAT	2-amino-3-ketobutyrate coenzyme A ligase, m	1	110.4	91.6	93.8	94.8	101	94.3	0.933663366	0.41134
Q9NWH9	SLTM	SAFB-like transcription modulator OS=Homo	14	111.6	99.4	98.1	98.9	105.5	98.5	0.933649289	0.4112
Q14155	ARHGEF7	Rho guanine nucleotide exchange factor 7 OS	1	110.5	106.4	97	105.5	108.45	101.25	0.933609959	0.410809
Q9UNL2	SSR3	Translocon-associated protein subunit gamma	1	106.6	98	99.4	91.6	102.3	95.5	0.933528837	0.410005
B3KRB2		cDNA FLJ33964 fis, clone CTONG2019029,	8	103.4	105.7	100.8	94.4	104.55	97.6	0.933524629	0.409963
D3DTX6	PPP1R9B	Neurabin-2 OS=Homo sapiens GN=PPP1R9E	13	104.6	99.7	95.3	95.4	102.15	95.35	0.933431229	0.409037
A8K6I5		Signal transducer and activator of transcriptio	2	105.6	97.1	97.9	91.3	101.35	94.6	0.933399112	0.408719
Q9NQ88	TIGAR	Fructose-2,6-bisphosphatase TIGAR OS=Hor	7	102.7	103	98.2	93.8	102.85	96	0.933398153	0.408709
P23526	AHCY	Adenosylhomocysteinase OS=Homo sapiens (	20	100.9	103.2	95	95.5	102.05	95.25	0.933365997	0.408391
Q9BSF4	C19orf52	Uncharacterized protein C19orf52 OS=Homo	4	106.8	98.8	94.7	97.2	102.8	95.95	0.933365759	0.408389
P52732	KIF11	Kinesin-like protein KIF11 OS=Homo sapien	13	106.9	102.4	95.7	99.6	104.65	97.65	0.933110368	0.405866
O75521	ECI2	Enoyl-CoA delta isomerase 2, mitochondrial (	15	102	102.7	95.6	95.4	102.35	95.5	0.933072789	0.405496

Q4LE36	ACLY	ACLY variant protein (Fragment) OS=Homo	32	100.3	101.4	93.6	94.6	100.85	94.1	0.933068914	0.405458
A0A024R5U5	ADAM10	ADAM metallopeptidase domain 10, isoform	9	110.1	102	99.7	98.2	106.05	98.95	0.933050448	0.405275
Q92966	SNAPC3	snRNA-activating protein complex subunit 3 (	1	103.2	101.4	96.5	94.4	102.3	95.45	0.933040078	0.405173
F1T0J8	RCOR2	REST corepressor 2 OS=Homo sapiens GN=I	6	102.9	97.2	94.1	92.6	100.05	93.35	0.933033483	0.405108
Q9HB66	MKKS	Alternative protein MKKS OS=Homo sapiens	2	85.1	134.1	107.4	97.1	109.6	102.25	0.932937956	0.404168
Q8NBZ0	INO80E	INO80 complex subunit E OS=Homo sapiens	1	106.2	102.1	99	95.3	104.15	97.15	0.932789246	0.402706
O14578	CIT	Citron Rho-interacting kinase OS=Homo sapi	12	103.3	99	94.6	94.1	101.15	94.35	0.932773109	0.402548
P17535	JUND	Transcription factor jun-D OS=Homo sapiens	5	106	100.6	90.6	102.1	103.3	96.35	0.932720232	0.402029
P36956	SREBF1	Sterol regulatory element-binding protein 1 O	1	108.4	101.1	101.1	94.3	104.75	97.7	0.932696897	0.4018
B2R8Y4		cDNA, FLJ94117, highly similar to Homo sap	4	108.7	105.2	106	93.5	106.95	99.75	0.932678822	0.401623
Q7Z5J4	RAI1	Retinoic acid-induced protein 1 OS=Homo sa	7	104.5	100.4	95.1	96	102.45	95.55	0.932650073	0.401341
Q59GJ6		Methylenetetrahydrofolate reductase (Fragme	2	97.9	103.9	95.3	92.9	100.9	94.1	0.932606541	0.400914
A0A024R3W2	TOMM20	Translocase of outer mitochondrial membrane	2	108.8	97.4	96.4	95.9	103.1	96.15	0.932589719	0.400749
Q7L5N1	COPS6	COP9 signalosome complex subunit 6 OS=Ho	8	110.1	100.5	98.2	98.2	105.3	98.2	0.932573599	0.400592
O00299	CLIC1	Chloride intracellular channel protein 1 OS=H	12	99.3	102.4	91.6	96.5	100.85	94.05	0.932573128	0.400587
Q9BQ04	RBM4B	RNA-binding protein 4B OS=Homo sapiens G	2	110.2	97.2	92.4	101	103.7	96.7	0.932497589	0.399848
B7Z518		cDNA FLJ55237, highly similar to Homo sap	2	110.2	89.7	89.7	96.7	99.95	93.2	0.932466233	0.399541
A0A024QYX(	EBP	Emopamil binding protein OS=Homo sapiens	3	103.6	100.7	95.5	95	102.15	95.25	0.932452276	0.399405
A0A024R4Q5	TFPT	TCF3 (E2A) fusion partner (In childhood Leu	2	103.8	97.4	92.8	94.8	100.6	93.8	0.932405567	0.398949
Q8WVY7	UBLCP1	Ubiquitin-like domain-containing CTD phosp	3	109.8	98.3	94.5	99.5	104.05	97	0.932244113	0.397373
Q5Y190	RESDA1	Anchor protein OS=Homo sapiens GN=RESI	3	102.8	102.2	99.2	91.9	102.5	95.55	0.932195122	0.396896
B9EK46	CGN	Cingulin OS=Homo sapiens GN=CGN PE=2	3	108.1	101.3	91.7	103.5	104.7	97.6	0.932187202	0.396818
A8K4K1		cDNA FLJ78216, highly similar to Homo sap	5	104.6	97.4	92.7	95.6	101	94.15	0.932178218	0.396731
A4D2Q0	UNC84A	Unc-84 homolog A (C. elegans) OS=Homo sa	12	101.5	99	92.3	94.6	100.25	93.45	0.932169576	0.396647
Q9Y219	JAG2	Protein jagged-2 OS=Homo sapiens GN=JAG	1	93.7	108.2	87.9	100.3	100.95	94.1	0.932144626	0.396404
Q06330	RBPJ	Recombining binding protein suppressor of ha	3	101.1	97.8	93	92.4	99.45	92.7	0.932126697	0.39623
P40925	MDH1	Malate dehydrogenase, cytoplasmic OS=Hom	15	102.2	101.1	95.6	93.9	101.65	94.75	0.93212002	0.396165
Q9BQT8	SLC25A21	Mitochondrial 2-oxodicarboxylate carrier OS=	5	100.1	97.3	93.9	90.1	98.7	92	0.932117528	0.39614
C9JBG5	SIDT2	SID1 transmembrane family member 2 OS=H	2	116	96.1	92.3	105.4	106.05	98.85	0.932107496	0.396043
A0A024R9B5	RBBP5	Retinoblastoma binding protein 5, isoform CF	11	104.9	102.4	98.4	94.8	103.65	96.6	0.931982634	0.394829
Q13952	NFYC	Nuclear transcription factor Y subunit gamma	2	97	94.1	85.9	92.2	95.55	89.05	0.931972789	0.394734
B3KRM2		Serine/threonine-protein phosphatase OS=Ho	1	108.3	101.8	98.5	97.3	105.05	97.9	0.931937173	0.394388
A0A024R0Q5	PPP1R13L	Protein phosphatase 1, regulatory (Inhibitor) s	1	103.3	109.7	97.3	101.2	106.5	99.25	0.931924883	0.394268
Q8IWI9	MGA	MAX gene-associated protein OS=Homo sapi	2	104.6	96.5	89.9	97.5	100.55	93.7	0.931874689	0.393782
Q8N6H7	ARFGAP2	ADP-ribosylation factor GTPase-activating pr	3	104.5	100.9	93.4	98	102.7	95.7	0.931840312	0.393448
Q9H0P0	NT5C3A	Cytosolic 5'-nucleotidase 3A OS=Homo sapie	7	102.7	101.1	96	93.9	101.9	94.95	0.931795878	0.393018
Q96T23	RSF1	Remodeling and spacing factor 1 OS=Homo s	11	104.1	102.3	95.1	97.2	103.2	96.15	0.931686047	0.391955
P09382	LGALS1	Galectin-1 OS=Homo sapiens GN=LGALS1 I	10	99.8	102.2	93.5	94.7	101	94.1	0.931683168	0.391927
A0A024QYZ0	SEC61G	Sec61 gamma subunit, isoform CRA_a OS=H	2	108.7	93.3	90.8	97.4	101	94.1	0.931683168	0.391927
Q59EK0		Epsilon isoform of regulatory subunit B56, pr	3	110.8	101.4	100.5	97.2	106.1	98.85	0.931668238	0.391782
Q9UK76	HN1	Hematological and neurological expressed 1 p	6	100.1	101.8	93.4	94.7	100.95	94.05	0.931649331	0.3916
Q9UKM7	MAN1B1	Endoplasmic reticulum mannosyl-oligosaccha	4	108.1	99.4	97.6	95.7	103.75	96.65	0.931566265	0.390797
Q53FA7	TP53I3	Quinone oxidoreductase PIG3 OS=Homo sap	3	101.7	101.4	95.3	93.9	101.55	94.6	0.931560807	0.390744
P27694	RPA1	Replication protein A 70 kDa DNA-binding s	24	103.8	100.7	95.8	94.7	102.25	95.25	0.931540342	0.390547
B7Z8T0		cDNA FLJ56198, highly similar to Serine/thre	2	109	105.4	107	92.7	107.2	99.85	0.931436567	0.389546
Q05086	UBE3A	Ubiquitin-protein ligase E3A OS=Homo sapie	5	109.7	94.3	91.1	98.9	102	95	0.931372549	0.388929
E7EV01	CAPN5	Calpain-5 OS=Homo sapiens GN=CAPN5 PE	2	104.5	102.4	103.1	89.6	103.45	96.35	0.931367811	0.388884
A0A024R7U6	MCM4	DNA helicase OS=Homo sapiens GN=MCM4	26	104	104.3	96.7	97.3	104.15	97	0.931349016	0.388703
Q9UEG4	ZNF629	Zinc finger protein 629 OS=Homo sapiens GI	1	95.8	103.6	88.7	97	99.7	92.85	0.931293882	0.388172
P60983	GMFB	Glia maturation factor beta OS=Homo sapiens	4	103.1	99.2	94.7	93.7	101.15	94.2	0.931290163	0.388136
Q9BYI3	FAM126A	Hyccin OS=Homo sapiens GN=FAM126A PI	2	113.4	91.8	88.8	102.3	102.6	95.55	0.93128655	0.388102
Q6UW63	KDELC1	KDEL motif-containing protein 1 OS=Homo	2	112.6	94	97.9	94.5	103.3	96.2	0.931268151	0.387925
P22059	OSBP	Oxysterol-binding protein 1 OS=Homo sapier	9	103.2	104.8	96.1	97.6	104	96.85	0.93125	0.38775
E5KS55		Succinate-CoA ligase subunit beta OS=Homo	15	108.1	99.8	98.2	95.4	103.95	96.8	0.931216931	0.387432
A0A0A0MQS	PYCRL	Pyrroline-5-carboxylate reductase OS=Homo	4	105.2	98.3	93.5	96	101.75	94.75	0.931203931	0.387307
D6W4Z6	hCG_23833	HCG23833, isoform CRA_b OS=Homo sapie	8	103.1	103.2	95.1	97	103.15	96.05	0.931168202	0.386964
A8K0F7		cDNA FLJ76587, highly similar to Homo sap	3	115.6	96.4	101	96.4	106	98.7	0.931132075	0.386617
A0A024RBQ5	OAS3	2'-5'-oligoadenylate synthetase 3, 100kDa, iso	6	113.1	98.9	93.3	104.1	106	98.7	0.931132075	0.386617
Q9BZQ6	EDEM3	ER degradation-enhancing alpha-mannosidase	7	102.5	100.7	93.7	95.5	101.6	94.6	0.931102362	0.386332
P11117	ACP2	Lysosomal acid phosphatase OS=Homo sapie	7	108.9	101.5	97.5	98.4	105.2	97.95	0.93108365	0.386153
Q9GZT9	EGLN1	Egl nine homolog 1 OS=Homo sapiens GN=E	1	104.6	102.6	95.3	97.6	103.6	96.45	0.930984556	0.385203
Q06547	GABPB1	GA-binding protein subunit beta-1 OS=Homo	2	102	106.6	99.9	94.3	104.3	97.1	0.93096836	0.385048
P51571	SSR4	Translocon-associated protein subunit delta O	5	109.6	100.4	99.4	96.1	105	97.75	0.930952381	0.384895
P11802	CDK4	Cyclin-dependent kinase 4 OS=Homo sapiens	2	108.1	99	91.6	101.2	103.55	96.4	0.930951231	0.384884
P57081	WDR4	tRNA (guanine-N(7)-)-methyltransferase non-	2	103	105.4	101.1	92.9	104.2	97	0.930902111	0.384414
Q5SVZ6	ZMYM1	Zinc finger MYM-type protein 1 OS=Homo s	6	102.1	103.4	95.7	95.6	102.75	95.65	0.930900243	0.384396
Q9H1I8	ASCC2	Activating signal cointegrator 1 complex subu	1	110.6	99.2	102.2	93.1	104.9	97.65	0.930886559	0.384265
B3KVH4		cDNA FLJ16549 fis, clone PLACE7003657, I	2	107.4	106.6	101.4	97.8	107	99.6	0.930841121	0.383831
Q9Y657	SPIN1	Spindlin-1 OS=Homo sapiens GN=SPIN1 PE	3	100.9	107.3	98.6	95.2	104.1	96.9	0.930835735	0.383779
B3KTL8		cDNA FLJ38476 fis, clone FEBRA2022504,	2	108.6	95	98.8	90.7	101.8	94.75	0.930746562	0.382928
Q9UID3	VPS51	Vacuolar protein sorting-associated protein 51	3	106.5	97	93	96.4	101.75	94.7	0.930712531	0.382603
P20290	BTF3	Transcription factor BTF3 OS=Homo sapiens	7	103.7	108.3	98.3	99	106	98.65	0.930660377	0.382105
Q6ZWH1		cDNA FLJ41081 fis, clone ADRGL2009755,	1	110.9	96.7	99.4	93.8	103.8	96.6	0.930635838	0.381871
Q59EH3		Acid phosphatase 1 isoform c variant (Fragme	7	104.4	97.3	94.9	92.8	100.85	93.85	0.930589985	0.381435
A0A024R6K3	SETD3	SET domain containing 3, isoform CRA_a OS	6	104.2	108.7	98.8	99.3	106.45	99.05	0.930483795	0.380424
V9HW62	HEL-S-74	Lactoylglutathione lyase OS=Homo sapiens G	11	101.8	103.8	97.2	94.1	102.8	95.65	0.930447471	0.380079
Q5BIX2	ARKL1	Chromosome 18 open reading frame 25, isofo	1	93.5	106.1	91.2	94.5	99.8	92.85	0.930360721	0.379255
Q9Y388	RBMX2	RNA-binding motif protein, X-linked 2 OS=F	3	106.1	99.2	93.1	97.9	102.65	95.5	0.930345835	0.379114
G5E975	SMARCB1	SWI/SNF related, matrix associated, actin dep	7	108.2	99.5	97.8	95.4	103.85	96.6	0.930187771	0.377615
Q9BUK6	MSTO1	Protein misato homolog 1 OS=Homo sapiens	3	100.9	96.6	91.8	91.9	98.75	91.85	0.930126582	0.377036
Q92558	WASF1	Wiskott-Aldrich syndrome protein family mei	2	105.5	99	89	101.2	102.25	95.1	0.93007335	0.376532
Q9BRP8	PYM1	Partner of Y14 and mago OS=Homo sapiens (	7	100.7	103.8	96.2	94	102.25	95.1	0.93007335	0.376532
Q8N9F0	NAT8L	N-acetylaspartate synthetase OS=Homo sapie	2	106.9	100.4	95.3	97.5	103.65	96.4	0.930053063	0.376341
Q8ND04	SMG8	Protein SMG8 OS=Homo sapiens GN=SMG8	2	101.3	103.1	99	91.1	102.2	95.05	0.930039139	0.37621
H3BP13	TRAPPC2L	Trafficking protein particle complex subunit 2	2	98.9	104	92.5	96.2	101.45	94.35	0.930014786	0.375979
Q27J81	INF2	Inverted formin-2 OS=Homo sapiens GN=INI	4	105.4	102.9	99	94.7	104.15	96.85	0.929908785	0.374979
P41229	KDM5C	Lysine-specific demethylase 5C OS=Homo sa	8	102.2	101.6	92.7	96.8	101.9	94.75	0.92983317	0.374266
B3KS69		cDNA FLJ35627 fis, clone SPLEN2011139, I	1	91.3	99.5	85.9	91.5	95.4	88.7	0.929769392	0.373665
Q9H8S9	MOB1A	MOB kinase activator 1A OS=Homo sapiens	5	109	100.3	99.1	95.5	104.65	97.3	0.929765886	0.373632
Q00013	MPP1	55 kDa erythrocyte membrane protein OS=Ho	5	105.3	106.8	96.7	100.5	106.05	98.6	0.929750118	0.373484
E5RJD8	TBCA	Tubulin-specific chaperone A OS=Homo sapi	8	99.8	103.6	92.2	96.9	101.7	94.55	0.929695182	0.372967
A8K646		cDNA FLJ75699, highly similar to Homo sap	1	103.5	102.7	97.3	94.4	103.1	95.85	0.929679922	0.372824
Q58WW2	DCAF6	DDB1- and CUL4-associated factor 6 OS=Ho	2	112.6	99.1	99.9	96.9	105.85	98.4	0.929617383	0.372237
P30626	SRI	Sorcin OS=Homo sapiens GN=SRI PE=1 SV=	3	98.6	100	92.4	92.2	99.3	92.3	0.929506546	0.371196
Q96EE3	SEH1L	Nucleoporin SEH1 OS=Homo sapiens GN=SI	14	106.2	102.3	97.2	96.6	104.25	96.9	0.929496403	0.371101
Q96T76	MMS19	MMS19 nucleotide excision repair protein hor	3	105.6	102.8	96.7	97	104.2	96.85	0.929462572	0.370784
Q9UPY3	DICER1	Endoribonuclease Dicer OS=Homo sapiens G	6	105.3	100.2	93.3	97.7	102.75	95.5	0.929440389	0.370576
A0A024R301	CHDH	Choline dehydrogenase, isoform CRA_a OS=	3	104.8	97.5	93.2	94.8	101.15	94	0.929312902	0.369383
Q7KYR7	BTN2A1	Butyrophilin subfamily 2 member A1 OS=Ho	2	110.8	101.1	94.8	102.1	105.95	98.45	0.929211892	0.36844
O95057	DIRAS1	GTP-binding protein Di-Ras1 OS=Homo sapi	1	104.8	92.9	83.9	99.8	98.85	91.85	0.929185635	0.368194
A8K4W5		cDNA FLJ76813, highly similar to Homo sap	12	101.1	100.8	92.5	95.1	100.95	93.8	0.929172858	0.368075
A8K5H3		cDNA FLJ78647, highly similar to Homo sap	2	106.4	105.3	95.4	101.3	105.85	98.35	0.929145017	0.367816
H7BY58	PCMT1	Protein-L-isoaspartate O-methyltransferase O	7	108.4	103.3	100	96.7	105.85	98.35	0.929145017	0.367816
R4GN35	DENND4C	DENN domain-containing protein 4C OS=Ho	3	109.7	99.1	96.1	97.9	104.4	97	0.929118774	0.367571
Q12834	CDC20	Cell division cycle protein 20 homolog OS=H	7	103.6	100.8	93	96.9	102.2	94.95	0.929060665	0.367029
P52739	ZNF131	Zinc finger protein 131 OS=Homo sapiens GI	3	107.5	99.7	93.6	98.9	103.6	96.25	0.929054054	0.366968
P62955	CACNG7	Voltage-dependent calcium channel gamma-7	1	113	84.3	52.5	130.8	98.65	91.65	0.929042068	0.366856
E9PR30	FAU	40S ribosomal protein S30 OS=Homo sapiens	1	102.4	107.5	91.9	103.1	104.95	97.5	0.929013816	0.366593
Q8N4J0	CARNMT1	Carnosine N-methyltransferase OS=Homo sap	1	112.1	99.1	101	95.2	105.6	98.1	0.928977273	0.366253
P05026	ATP1B1	Sodium/potassium-transporting ATPase subu	9	106	105.2	99.9	96.3	105.6	98.1	0.928977273	0.366253
Q92896	GLG1	Golgi apparatus protein 1 OS=Homo sapiens (	26	103.8	101.7	96.1	94.8	102.75	95.45	0.928953771	0.366034
Q13535	ATR	Serine/threonine-protein kinase ATR OS=Hor	5	104.9	100.5	95.3	95.5	102.7	95.4	0.928919182	0.365713
A0A024R6N2	CDC42BPB	CDC42 binding protein kinase beta (DMPK-li	5	115	101.3	102.7	98.2	108.15	100.45	0.928802589	0.36463
D3DPK5	SH3BGRL3	SH3 domain binding glutamic acid-rich protei	3	104.5	99	94	95	101.75	94.5	0.928746929	0.364114
B4DGC6		cDNA FLJ58177, highly similar to Transmem	2	109.8	96.4	95.5	96	103.1	95.75	0.92870999	0.363771
Q9HAF1	MEAF6	Chromatin modification-related protein MEA	3	107.7	105.4	99.1	98.8	106.55	98.95	0.928671985	0.363419
Q9H6Y2	WDR55	WD repeat-containing protein 55 OS=Homo s	8	102.8	103.2	93.5	97.8	103	95.65	0.928640777	0.36313
Q96H79	ZC3HAV1L	Zinc finger CCCH-type antiviral protein 1-lik	5	105.5	101.9	95.3	97.3	103.7	96.3	0.928640309	0.363126
P48201	ATP5G3	ATP synthase F(0) complex subunit C3, mitoc	1	95.9	108.5	100	89.8	102.2	94.9	0.928571429	0.362488
Q6FIC5	CLIC4	Chloride intracellular channel protein OS=Ho	5	105.9	99.9	91.2	99.9	102.9	95.55	0.928571429	0.362488
B7Z3K9		Fructose-bisphosphate aldolase OS=Homo sap	7	96	102.8	90.1	94.5	99.4	92.3	0.928571429	0.362488
Q8WV22	NSMCE1	Non-structural maintenance of chromosomes (	3	108	97.5	93.7	97.1	102.75	95.4	0.928467153	0.361525

P27635	RPL10	60S ribosomal protein L10 OS=Homo sapiens	2	108.1	105.5	95.1	103.2	106.8	99.15	0.928370787	0.360636
Q14011	CIRBP	Cold-inducible RNA-binding protein OS=Hor	4	106.4	101.6	96.4	96.7	104	96.55	0.928365385	0.360586
Q92834	RPGR	X-linked retinitis pigmentosa GTPase regulat	1	100.1	114.8	92.9	106.6	107.45	99.75	0.928338762	0.360341
Q9Y2Z9	COQ6	Ubiquinone biosynthesis monooxygenase CO	1	95.2	108.5	95.6	93.5	101.85	94.55	0.92832597	0.360223
A8K0D4		cDNA FLJ78693, highly similar to Homo sap	3	103	99.3	91.4	96.4	101.15	93.9	0.928324271	0.360207
Q1ED39	KNOP1	Lysine-rich nucleolar protein 1 OS=Homo sap	6	105.7	99.3	93.5	96.8	102.5	95.15	0.928292683	0.359916
E7EX59	PCCB	Propionyl-CoA carboxylase beta chain, mitoc	4	106.9	98.1	94.8	95.5	102.5	95.15	0.928292683	0.359916
A0A0A0MTS:	GPI	Glucose-6-phosphate isomerase (Fragment) O	20	98.6	104.8	96	92.8	101.7	94.4	0.928220256	0.35925
Q8IWC1	MAP7D3	MAP7 domain-containing protein 3 OS=Hom	1	102.7	99.3	87.6	99.9	101	93.75	0.928217822	0.359227
Q9HAU5	UPF2	Regulator of nonsense transcripts 2 OS=Hom	6	111.4	101.5	98.3	99.3	106.45	98.8	0.928135275	0.358468
L0R6F6	CSRP2BP	Alternative protein CSRP2BP OS=Homo sapi	1	114.5	98.4	96.6	101	106.45	98.8	0.928135275	0.358468
Q96HQ2	CDKN2AIPNI	CDKN2AIP N-terminal-like protein OS=Hom	2	116.1	102.3	99.7	103	109.2	101.35	0.928113553	0.358269
A4D1W8	UCC1	Ependymin related protein 1 (Zebrafish), isof	2	106.6	101.8	97.4	96	104.2	96.7	0.928023033	0.357438
Q9UKG9	CROT	Peroxisomal carnitine O-octanoyltransferase	3	106	102.4	93.2	100.2	104.2	96.7	0.928023033	0.357438
P07741	APRT	Adenine phosphoribosyltransferase OS=Hom	8	103.1	101	95.6	93.8	102.05	94.7	0.927976482	0.357011
Q96BW9	TAMM41	Phosphatidate cytidylyltransferase, mitochon	4	108.6	99.2	92.9	99.9	103.9	96.4	0.927815207	0.355535
Q6UVK1	CSPG4	Chondroitin sulfate proteoglycan 4 OS=Homo	39	105	101.3	95.1	96.3	103.15	95.7	0.927775085	0.355168
A0A024RB12	RASSF8	Ras association (RalGDS/AF-6) domain famil	2	108.1	103.4	98.3	97.9	105.75	98.1	0.927659574	0.354113
Q8NCN4	RNF169	E3 ubiquitin-protein ligase RNF169 OS=Hom	2	103.4	102.2	96	94.7	102.8	95.35	0.927529183	0.352925
O14745	SLC9A3R1	Na(+)/H(+) exchange regulatory cofactor NH	7	102.8	102.7	92.4	98.2	102.75	95.3	0.927493917	0.352604
Q9Y5B0	CTDP1	RNA polymerase II subunit A C-terminal don	1	108.5	99.6	96.7	96.3	104.05	96.5	0.927438731	0.352102
Q9Y217	MTMR6	Myotubularin-related protein 6 OS=Homo sap	2	107.3	104.9	97.9	98.9	106.1	98.4	0.927426956	0.351995
U3KQ54	PMF1-BGLAP	HCG2044777 OS=Homo sapiens GN=PMF1-	1	112.5	98.3	90.2	105.3	105.4	97.75	0.927419355	0.351926
P0DN79	CBSL	Cystathionine beta-synthase-like protein OS=	10	104.5	100.6	95.4	94.8	102.55	95.1	0.927352511	0.351319
Q7KZN9	COX15	Cytochrome c oxidase assembly protein COX	6	104.9	100.2	96.6	93.6	102.55	95.1	0.927352511	0.351319
B7Z2A4		Receptor-type tyrosine-protein phosphatase O	1	96.5	103	89.9	95.1	99.75	92.5	0.927318296	0.351008
Q05932	FPGS	Folylpolyglutamate synthase, mitochondrial C	2	97.5	94.7	88.2	90	96.1	89.1	0.927159209	0.349567
Q96DB5	RMDN1	Regulator of microtubule dynamics protein 1	8	107.1	101.5	97.3	96.1	104.3	96.7	0.927133269	0.349332
P42566	EPS15	Epidermal growth factor receptor substrate 15	6	110.7	96.3	95	96.9	103.5	95.95	0.92705314	0.348607
A0A024R9V7	LOC92689	Uncharacterized protein OS=Homo sapiens G	2	103.9	104.4	93	100.1	104.15	96.55	0.927028325	0.348383
Q9Y5P4	COL4A3BP	Collagen type IV alpha-3-binding protein OS	3	101.4	107.9	96.2	97.8	104.65	97	0.926899188	0.347218
Q6IBW4	NCAPH2	Condensin-2 complex subunit H2 OS=Homo	4	110.7	102.7	99.4	98.4	106.7	98.9	0.926897844	0.347205
Q6ZMG9	CERS6	Ceramide synthase 6 OS=Homo sapiens GN=	2	112.8	94.9	103.6	88.9	103.85	96.25	0.926817525	0.346482
Q96D53	COQ8B	Atypical kinase COQ8B, mitochondrial OS=H	4	103.5	98.4	93.2	93.9	100.95	93.55	0.926696384	0.345392
Q6R327	RICTOR	Rapamycin-insensitive companion of mTOR	1	120.1	91.2	101.3	94.5	105.65	97.9	0.926644581	0.344926
Q24JP5	TMEM132A	Transmembrane protein 132A OS=Homo sapi	1	108.5	91.8	90.6	95	100.15	92.8	0.926610085	0.344617
Q6IAX1	FDFT1	FDFT1 protein OS=Homo sapiens GN=FDFT	15	103.3	99.5	95.8	92.1	101.4	93.95	0.9265286	0.343886
E9PEJ6	ATP11A	Phospholipid-transporting ATPase OS=Homo	1	109.7	102.6	103.4	93.3	106.15	98.35	0.926519077	0.343801
Q8WV44	TRIM41	E3 ubiquitin-protein ligase TRIM41 OS=Hom	2	106.9	99.9	91.5	100.1	103.4	95.8	0.926499033	0.343621
Q9BRK5	SDF4	45 kDa calcium-binding protein OS=Homo sa	6	102.7	103.9	94.9	96.5	103.3	95.7	0.92642788	0.342984
Q9H7B4	SMYD3	Histone-lysine N-methyltransferase SMYD3	1	102.9	99.6	90.8	96.8	101.25	93.8	0.926419753	0.342911
H0UI80	TH1L	Negative elongation factor C/D OS=Homo sa	2	114	95.2	96.2	97.6	104.6	96.9	0.926386233	0.342611
G9BZK0		Glyceraldehyde-3-phosphate dehydrogenase	1	108.4	97.9	101.6	89.5	103.15	95.55	0.926320892	0.342027
Q14686	NCOA6	Nuclear receptor coactivator 6 OS=Homo sap	2	100.3	99.1	91	93.7	99.7	92.35	0.926278837	0.341651
P27348	YWHAQ	14-3-3 protein theta OS=Homo sapiens GN=Y	9	103.8	100.8	95.3	94.2	102.3	94.75	0.926197458	0.340925
Q9Y5K6	CD2AP	CD2-associated protein OS=Homo sapiens G	7	106.3	102.2	96	97.1	104.25	96.55	0.926139089	0.340405
A0A024RB16	FAM62A	Family with sequence similarity 62 (C2 doma	1	111.3	109.2	99.7	104.5	110.25	102.1	0.926077098	0.339853
P83110	HTRA3	Serine protease HTRA3 OS=Homo sapiens G	1	101.9	110.4	100.5	96.1	106.15	98.3	0.926048045	0.339594
A0A024R0N7	SAMD4B	Sterile alpha motif domain containing 4B, iso	2	106.4	104.3	96.8	98.3	105.35	97.55	0.925961082	0.338821
A8K984		Structural maintenance of chromosomes prote	22	107.4	104.5	98.3	97.9	105.95	98.1	0.925908447	0.338354
Q96GS4	BORCS6	BLOC-1-related complex subunit 6 OS=Hom	3	111.6	101.3	97.8	99.3	106.45	98.55	0.925786754	0.337274
Q99720	SIGMAR1	Sigma non-opioid intracellular receptor 1 OS	3	108.6	98.9	97.5	94.6	103.75	96.05	0.925783133	0.337242
Q8TDJ6	DMXL2	DmX-like protein 2 OS=Homo sapiens GN=D	1	105.5	98.8	84.6	104.5	102.15	94.55	0.925599608	0.335619
P15531	NME1	Nucleoside diphosphate kinase A OS=Homo s	1	104.9	99.3	93.6	95.4	102.1	94.5	0.925563173	0.335297
Q9NUY8	TBC1D23	TBC1 domain family member 23 OS=Homo s	2	111.1	99.8	94.2	101	105.45	97.6	0.925557136	0.335243
A0A0D9SF60	PKP4	Plakophilin-4 OS=Homo sapiens GN=PKP4 I	1	108.2	107.9	95.7	104.3	108.05	100	0.925497455	0.334717
Q13451	FKBP5	Peptidyl-prolyl cis-trans isomerase FKBP5 O	16	105.2	103.9	96.2	97.3	104.55	96.75	0.925394548	0.33381
Q7L3T8	PARS2	Probable proline--tRNA ligase, mitochondrial	6	103.8	103.8	93.6	98.5	103.8	96.05	0.925337187	0.333305
O95487	SEC24B	Protein transport protein Sec24B OS=Homo s	4	104.9	100	89.9	99.7	102.45	94.8	0.925329429	0.333237
O60502	MGEA5	Protein O-GlcNAcase OS=Homo sapiens GN	3	105.9	92.1	89.4	93.8	99	91.6	0.925252525	0.33256
O43567	RNF13	E3 ubiquitin-protein ligase RNF13 OS=Homo	1	106.9	100.4	93.3	98.5	103.65	95.9	0.925229137	0.332355
H0YBH7	SARAF	Store-operated calcium entry-associated regul	3	106.7	100.5	93.3	98.4	103.6	95.85	0.92519305	0.332039
Q05CW7	NAT10	NAT10 protein (Fragment) OS=Homo sapien	1	109.6	101.6	100.5	94.9	105.6	97.7	0.925189394	0.332006
Q96GX9	APIP	Methylthioribulose-1-phosphate dehydratase	5	104.9	103.6	95.6	97.3	104.25	96.45	0.925179856	0.331922
Q9HCM4	EPB41L5	Band 4.1-like protein 5 OS=Homo sapiens G	2	103.5	103.6	93.1	98.5	103.55	95.8	0.925156929	0.331722
P29083	GTF2E1	General transcription factor IIE subunit 1 OS	5	108.1	104.3	98.9	97.6	106.2	98.25	0.925141243	0.331584
Q9H9B4	SFXN1	Sideroflexin-1 OS=Homo sapiens GN=SFXN	11	106.2	100.7	98.1	93.3	103.45	95.7	0.925084582	0.331087
Q8IYS1	PM20D2	Peptidase M20 domain-containing protein 2 C	4	110.5	99	99.2	94.6	104.75	96.9	0.925059666	0.330869
Q13257	MAD2L1	Mitotic spindle assembly checkpoint protein	7	105	104.5	98	95.8	104.75	96.9	0.925059666	0.330869
Q9BSY4	CHCHD5	Coiled-coil-helix-coiled-coil-helix domain-co	5	102.8	94.6	87.8	94.8	98.7	91.3	0.925025329	0.330568
A0A024R8K0	AMZ2	Archaemetzincins-2, isoform CRA_d OS=Ho	1	96	102.7	91.9	91.9	99.35	91.9	0.925012582	0.330457
O43427	FIBP	Acidic fibroblast growth factor intracellular-b	8	106.3	104.4	96.9	98	105.35	97.45	0.925011865	0.33045
B7Z5W1		cDNA FLJ54854, highly similar to Junctional	6	108.6	104.7	99.6	97.7	106.65	98.65	0.924988279	0.330244
A8K1M4		cDNA FLJ76754, highly similar to Homo sap	1	110.8	94.5	92.7	97.2	102.65	94.95	0.924987823	0.33024
Q96M27	PRRC1	Protein PRRC1 OS=Homo sapiens GN=PRR	3	108.1	106.4	102.5	95.9	107.25	99.2	0.924941725	0.329837
Q08378	GOLGA3	Golgin subfamily A member 3 OS=Homo sap	15	106.7	103.8	97.6	97.1	105.25	97.35	0.924940618	0.329827
Q6P158	DHX57	Putative ATP-dependent RNA helicase DHX5	8	107.3	100.4	95.1	97	103.85	96.05	0.924891671	0.329399
P11474	ESRRA	Steroid hormone receptor ERR1 OS=Homo sa	2	103.7	102.3	96.7	93.8	103	95.25	0.924757282	0.328227
F8WBA3	PRKD1	Serine/threonine-protein kinase OS=Homo sap	1	133.7	82.8	98.8	101.4	108.25	100.1	0.924711316	0.327826
Q6P3X3	TTC27	Tetratricopeptide repeat protein 27 OS=Hom	5	105	101.9	92.7	98.6	103.45	95.65	0.924601257	0.326869
B3KQU6		cDNA PSEC0207 fis, clone HEMBA1002981	2	101.9	104.9	94.4	96.8	103.4	95.6	0.924564797	0.326552
Q9NUT2	ABCB8	ATP-binding cassette sub-family B member 8	4	104.9	103.2	97	95.4	104.05	96.2	0.924555502	0.326471
Q8N8R3	SLC25A29	Mitochondrial basic amino acids transporter C	1	117.6	101	107	95.1	109.3	101.05	0.924519671	0.32616
A0A024QZC8	FLJ23436	Uncharacterized protein OS=Homo sapiens G	2	105.8	104.8	93.1	101.6	105.3	97.35	0.924501425	0.326001
O75879	GATB	Glutamyl-tRNA(Gln) amidotransferase subun	2	103.6	105.6	98.7	94.7	104.6	96.7	0.924474187	0.325765
A8MQ07	RAPGEF5	Rap guanine nucleotide exchange factor 5 OS	1	96.4	135.1	111.6	102.4	115.75	107	0.924406048	0.325174
J3KNF8	CYB5B	Cytochrome b5 type B OS=Homo sapiens G	4	105.9	97.8	93.3	95	101.85	94.15	0.924398625	0.32511
Q6IBS0	TWF2	Twinfilin-2 OS=Homo sapiens GN=TWF2 P	6	105.6	98	95.9	92.3	101.8	94.1	0.924361493	0.324788
Q6NZY4	ZCCHC8	Zinc finger CCHC domain-containing protein	17	108	99.5	95.2	96.6	103.75	95.9	0.924337349	0.324579
Q4G148	GXYLT1	Glucoside xylosyltransferase 1 OS=Homo sap	2	103.3	106.5	97.2	96.7	104.9	96.95	0.924213537	0.323508
P61086	UBE2K	Ubiquitin-conjugating enzyme E2 K OS=Hom	7	105.1	104.6	99.6	94.2	104.85	96.9	0.924177396	0.323196
P50990	CCT8	T-complex protein 1 subunit theta OS=Homo	36	102.3	103.4	95.9	94.2	102.85	95.05	0.9241614	0.323058
P18827	SDC1	Syndecan-1 OS=Homo sapiens GN=SDC1 P	4	107.8	105.8	97.6	99.8	106.8	98.7	0.924157303	0.323023
A0A024R7L2	HSPC142	HSPC142 protein, isoform CRA_a OS=Homo	4	100.8	103.3	92.5	96.1	102.05	94.3	0.924056835	0.322156
O43731	KDELR3	ER lumen protein-retaining receptor 3 OS=H	3	112.5	92.9	96.9	92.9	102.7	94.9	0.924050633	0.322103
Q16539	MAPK14	Mitogen-activated protein kinase 14 OS=Hom	6	100.5	98	94.5	88.9	99.25	91.7	0.923929471	0.32106
R4GMX8	RANBP10	Ran-binding protein 10 OS=Homo sapiens G	3	103.7	111.6	104.2	94.7	107.65	99.45	0.923827218	0.320181
Q05GC8	BCAR2	Breast cancer anti-estrogen resistance 2 OS=	1	113.1	99.5	102.6	93.8	106.3	98.2	0.923800564	0.319953
Q8N9N7	LRRC57	Leucine-rich repeat-containing protein 57 OS	2	116.8	91.6	96.7	95.8	104.2	96.25	0.923704415	0.319128
Q9BWW4	SSBP3	Single-stranded DNA-binding protein 3 OS=	1	109	103.3	98.4	97.7	106.15	98.05	0.923692887	0.319029
A0A023QT24	COX1	Cytochrome c oxidase subunit 1 OS=Homo sa	3	103.6	99.5	96.5	91.1	101.55	93.8	0.923682915	0.318944
Q8N1G2	CMTR1	Cap-specific mRNA (nucleoside-2'-O-)-methy	4	100.6	106.4	96.2	95	103.5	95.6	0.923671498	0.318846
P49815	TSC2	Tuberin OS=Homo sapiens GN=TSC2 PE=1	1	98.9	108	98	93.1	103.45	95.55	0.923634606	0.31853
Q9H300	PARL	Presenilins-associated rhomboid-like protein,	2	110.6	100.1	91.7	102.9	105.35	97.3	0.92358804	0.318132
Q9NR77	PXMP2	Peroxisomal membrane protein 2 OS=Homo s	1	105.4	102.6	95.6	96.5	104	96.05	0.923557692	0.317873
A0A0F7G8J1	PLG	Plasminogen OS=Homo sapiens GN=PLG PE	1	99.7	100.4	94.4	90.4	100.05	92.4	0.923538231	0.317706
A0A087WVC	PTPRJ	Receptor-type tyrosine-protein phosphatase et	2	103.2	99.4	89.4	97.7	101.3	93.55	0.923494571	0.317333
Q969Q0	RPL36AL	60S ribosomal protein L36a-like OS=Homo s	1	103.8	100.1	94.8	93.5	101.95	94.15	0.923491908	0.317311
A0A140VKE		Testis tissue sperm-binding protein Li 66n OS	3	111.8	97.3	100.3	92.8	104.55	96.55	0.923481588	0.317222
Q8IY92	SLX4	Structure-specific endonuclease subunit SLX4	1	108.2	103.4	90.3	105.1	105.8	97.7	0.923440454	0.316872
Q14691	GINS1	DNA replication complex GINS protein PSF1	1	96.4	98.1	91.3	88.3	97.25	89.8	0.923393316	0.31647
G5E9D4	ELP4	Elongation protein 4 homolog (S. cerevisiae),	1	121	89	99.6	94.3	105	96.95	0.923333333	0.315959
A0A0C4DH7	MPP2	MAGUK p55 subfamily member 2 OS=Homo	2	105.2	106.1	97.4	97.7	105.65	97.55	0.923331756	0.315945
E9PL71	EEF1D	Elongation factor 1-delta (Fragment) OS=Hor	1	104.6	114.2	98.1	103.9	109.4	101	0.92321755	0.314973
Q9BZF3	OSBPL6	Oxysterol-binding protein-related protein 6 O	5	107.8	99.2	96.9	94.2	103.5	95.55	0.923188406	0.314726
A0A024R774	NRF1	Nuclear respiratory factor 1, isoform CRA_b	1	104.7	108.7	98.4	98.6	106.7	98.5	0.923149016	0.314391
P53677	AP3M2	AP-3 complex subunit mu-2 OS=Homo sapie	1	107.8	106.6	108.5	89.4	107.2	98.95	0.923041045	0.313476
B3KY60		cDNA FLJ16777 fis, clone BRHIP2029567,	22	113.1	98.7	99.6	95.9	105.9	97.75	0.923040604	0.313472
Q6P1S2	C3orf33	Protein C3orf33 OS=Homo sapiens GN=C3o	1	115.6	94.6	96.2	97.8	105.1	97	0.922930542	0.31254
Q9Y6D9	MAD1L1	Mitotic spindle assembly checkpoint protein	10	106.4	99.6	94.2	95.9	103	95.05	0.922815534	0.311569
P11182	DBT	Lipoamide acyltransferase component of bran	5	106.4	98.1	94.9	93.8	102.25	94.35	0.922738386	0.310917
P62314	SNRPD1	Small nuclear ribonucleoprotein Sm D1 OS=I	5	104.6	97.3	94.3	92	100.95	93.15	0.922734027	0.310881

A0A024R0L6	PAFAH1B3	Platelet-activating factor acetylhydrolase, isof	8	99.1	100.9	90.7	93.8	100	92.25	0.9225	0.308912
Q92581	SLC9A6	Sodium/hydrogen exchanger 6 OS=Homo sap	1	107.1	104.5	98.8	96.4	105.8	97.6	0.922495274	0.308872
Q8WVJ2	NUDCD2	NudC domain-containing protein 2 OS=Homo	4	97.4	98.7	89	91.9	98.05	90.45	0.922488526	0.308815
Q53HL1		Myosin regulatory light chain MRCL3 variant	4	110.7	94.4	91.8	97.4	102.55	94.6	0.922476841	0.308717
A0A024RAD8	ALDH4A1	Aldehyde dehydrogenase 4 family, member A	7	106.5	98.4	97	92	102.45	94.5	0.922401171	0.308083
Q9UN36	NDRG2	Protein NDRG2 OS=Homo sapiens GN=NDR	1	102.4	112.8	95.8	102.7	107.6	99.25	0.92239777	0.308054
A0A1B0GVH	FTO	Alpha-ketoglutarate-dependent dioxygenase F	1	105.6	101.8	88.1	103.2	103.7	95.65	0.922372228	0.30784
Q59GP6		Polyhomeotic-like protein 1 variant (Fragmen	3	106.9	97.9	97.1	91.8	102.4	94.45	0.922363281	0.307765
B2R9K8		cDNA, FLJ94440, highly similar to Homo sap	10	107.4	105	99.1	96.8	106.2	97.95	0.922316384	0.307372
B2RAN0		cDNA, FLJ95010, highly similar to Homo sap	1	120.4	99.6	106.5	96.4	110	101.45	0.922272727	0.307007
Q14790	CASP8	Caspase-8 OS=Homo sapiens GN=CASP8 PE	2	109.8	102.4	96.2	99.5	106.1	97.85	0.922243167	0.30676
Q9H7P6	MVB12B	Multivesicular body subunit 12B OS=Homo s	1	118.5	98.6	98	102.2	108.55	100.1	0.922155689	0.306029
Q86UL3	GPAT4	Glycerol-3-phosphate acyltransferase 4 OS=H	2	103.9	104.2	101.9	90	104.05	95.95	0.922152811	0.306005
P51965	UBE2E1	Ubiquitin-conjugating enzyme E2 E1 OS=Ho	1	101.2	106.8	96.3	95.5	104	95.9	0.922115385	0.305693
H7C426	RNF181	E3 ubiquitin-protein ligase RNF181 (Fragmen	1	105.1	105	98.5	95.2	105.05	96.85	0.921941932	0.304248
Q9NX00	TMEM160	Transmembrane protein 160 OS=Homo sapien	2	108	99.5	97.5	93.8	103.75	95.65	0.921927711	0.304129
R4GNH3	PSMC3	26S protease regulatory subunit 6A OS=Hom	1	106.7	104.6	96.9	97.9	105.65	97.4	0.921911973	0.303998
A8K9E1		cDNA FLJ77957, highly similar to Homo sap	11	106.5	103.4	98.1	95.4	104.95	96.75	0.921867556	0.303629
Q08AM6	VAC14	Protein VAC14 homolog OS=Homo sapiens G	3	111.7	85.2	90.3	91.2	98.45	90.75	0.921787709	0.302967
Q9NX74	DUS2	tRNA-dihydrouridine(20) synthase [NAD(P)+	1	107.5	100.9	93.4	98.7	104.2	96.05	0.921785029	0.302945
Q92547	TOPBP1	DNA topoisomerase 2-binding protein 1 OS=	3	110.5	96.4	89.5	101.2	103.45	95.35	0.921701305	0.30225
Q8WV41	SNX33	Sorting nexin-33 OS=Homo sapiens GN=SNX	1	103.7	107	90.2	104	105.35	97.1	0.921689606	0.302154
A0A024RDS3	ZNF198	Zinc finger protein 198, isoform CRA_b OS=	6	108.8	104.4	93.7	102.8	106.6	98.25	0.921669794	0.301989
Q8WUV3	PRMT3	PRMT3 protein (Fragment) OS=Homo sapien	2	102.5	109	97.9	97	105.75	97.45	0.921513002	0.300693
B2RBY4		DNA primase OS=Homo sapiens PE=2 SV=1	3	106.1	100.2	89.9	100.2	103.15	95.05	0.921473582	0.300367
A8K032		Translocating chain-associated membrane pro	3	117.7	92.4	96.8	96.8	105.05	96.8	0.921465969	0.300305
Q14296	FASTK	Fas-activated serine/threonine kinase OS=Ho	1	104.4	104.1	95.9	96.2	104.25	96.05	0.921342926	0.29929
Q9Y3A6	TMED5	Transmembrane emp24 domain-containing pr	5	105.5	103	100.5	91.6	104.25	96.05	0.921342926	0.29929
Q8TAE8	GADD45GIP1	Growth arrest and DNA damage-inducible pro	10	111.4	97	97.4	94.6	104.2	96	0.921305182	0.29898
Q9H9B1	EHMT1	Histone-lysine N-methyltransferase EHMT1 O	7	107	100.1	93.5	97.3	103.55	95.4	0.921294061	0.298888
A0A024QZN5	ZWINT	ZW10 interactor, isoform CRA_b OS=Homo	1	106.8	98.7	80.3	109	102.75	94.65	0.921167883	0.297852
Q8NI62	OK/KNS-cl.6	Ribosomal protein S2 OS=Homo sapiens GN=	1	115.7	91	102.2	88.2	103.35	95.2	0.921141751	0.297637
A0A024RC16	CD99L2	CD99 antigen-like 2, isoform CRA_d OS=Ho	1	105.6	107.3	96.4	99.7	106.45	98.05	0.921089713	0.29721
B3KQF4		cDNA FLJ90373 fis, clone NT2RP2004606, h	5	103.6	101.5	94.7	94.2	102.55	94.45	0.921014139	0.296591
A0A140VJT8		Testicular tissue protein Li 164 OS=Homo sap	18	102.7	101	92.6	95	101.85	93.8	0.920962199	0.296167
A0A0S2Z5D6	ABHD5	Abhydrolase domain containing 5 isoform 1 (	1	110.6	104.4	105.9	92.1	107.5	99	0.920930233	0.295905
B1AKK2	DDAH1	Dimethylarginine dimethylaminohydrolase 1,	9	105.7	101.6	92	98.9	103.65	95.45	0.920887603	0.295557
Q96JB2	COG3	Conserved oligomeric Golgi complex subunit	2	114.7	97.5	96.2	99.2	106.1	97.7	0.920829406	0.295082
Q9Y2V2	CARHSP1	Calcium-regulated heat-stable protein 1 OS=H	2	97.8	100.5	82.3	100.3	99.15	91.3	0.92082703	0.295062
P51688	SGSH	N-sulphoglucosamine sulphohydrolase OS=H	1	106.1	102.3	96.2	95.7	104.2	95.95	0.920825336	0.295049
H0Y7R8	AFDN	Afadin (Fragment) OS=Homo sapiens GN=A	1	108.5	115.7	103.5	102.9	112.1	103.2	0.920606601	0.293267
Q86WJ1	CHD1L	Chromodomain-helicase-DNA-binding protei	3	106.9	103.3	96.6	96.9	105.1	96.75	0.920551855	0.292822
V9HWC7	HEL-S-128m	Epididymis secretory sperm binding protein L	16	102.9	103.5	95.9	94.1	103.2	95	0.920542636	0.292748
Q8N129	CNPY4	Protein canopy homolog 4 OS=Homo sapiens	3	104.3	105.8	96.4	97	105.05	96.7	0.920514041	0.292515
Q5U5X0	LYRM7	Complex III assembly factor LYRM7 OS=Ho	1	105.6	95.6	90.9	94.3	100.6	92.6	0.920477137	0.292216
Q6NXT4	SLC30A6	Zinc transporter 6 OS=Homo sapiens GN=SL	2	107.5	96.2	96.3	91.2	101.85	93.75	0.920471281	0.292169
Q96CU9	FOXRED1	FAD-dependent oxidoreductase domain-conta	4	101	103.7	91.3	97.1	102.35	94.2	0.920371275	0.291358
Q8N5A5	ZGPAT	Zinc finger CCCH-type with G patch domain-	9	108.2	98.9	96	94.6	103.55	95.3	0.920328344	0.291011
A0A024R4R3	CNOT3	CCR4-NOT transcription complex, subunit 3,	3	108.9	99.4	92.7	99	104.15	95.85	0.920307249	0.290841
B4E0T2	PEX5	Peroxisomal targeting signal 1 receptor OS=H	2	110.2	99.2	94	98.7	104.7	96.35	0.920248329	0.290364
P11169	SLC2A3	Solute carrier family 2, facilitated glucose tra	3	98.4	103.2	98.5	87	100.8	92.75	0.920138889	0.289481
B0QZ18	CPNE1	Copine-1 OS=Homo sapiens GN=CPNE1 PE	4	100.4	103.7	91.2	96.6	102.05	93.9	0.920137188	0.289467
O00458	IFRD1	Interferon-related developmental regulator 1 O	1	103.3	104.5	94	97.2	103.9	95.6	0.920115496	0.289292
A0A0K2GN21	BCKDHB	BCKDHB protein OS=Homo sapiens GN=BC	2	105	101.3	96.7	93.1	103.15	94.9	0.920019389	0.288519
Q9NWL6	ASNSD1	Asparagine synthetase domain-containing pro	1	104.8	104	95.2	96.9	104.4	96.05	0.920019157	0.288517
A7MBN3	COL4A5	Collagen, type IV, alpha 5 OS=Homo sapiens	1	101	91.4	84.9	92.1	96.2	88.5	0.91995842	0.288028
B7Z5U5		cDNA FLJ61655, highly similar to Phosphory	1	96.3	100.9	88.5	92.9	98.6	90.7	0.919878296	0.287385
A0A0C4DFL7	CYP51A1	Lanosterol 14-alpha demethylase OS=Homo s	12	103.2	106.4	99.5	93.3	104.8	96.4	0.919847328	0.287137
Q9Y6Y0	IVNS1ABP	Influenza virus NS1A-binding protein OS=Ho	2	98.2	108.9	99.1	91.4	103.55	95.25	0.919845485	0.287122
B4DGG2		cDNA FLJ56070, highly similar to Sorting ne	2	106.7	104.1	92	101.9	105.4	96.95	0.919829222	0.286992
Q9BWL3	C1orf43	Uncharacterized protein C1orf43 OS=Homo s	1	109	101.7	94	99.8	105.35	96.9	0.919791172	0.286686
A0A0R4J2E2	R3HCC1	R3H and coiled-coil domain-containing protei	1	105.8	99.9	97.9	91.3	102.85	94.6	0.919786096	0.286646
H3BLV9	SRPK1	SRSF protein kinase 1 (Fragment) OS=Homo	6	108.4	101	96.5	96.1	104.7	96.3	0.919770774	0.286523
J3KQ12	BSCL2	HCG2020143, isoform CRA_b OS=Homo sap	1	101	105.8	100.3	89.9	103.4	95.1	0.919729207	0.286191
H0Y8I7	TOPBP1	DNA topoisomerase 2-binding protein 1 (Frag	1	113.8	104.1	104.7	95.7	108.95	100.2	0.91968793	0.28586
O94923	GLCE	D-glucuronyl C5-epimerase OS=Homo sapier	1	103.6	100.5	88.1	99.6	102.05	93.85	0.919647232	0.285535
Q8IW50	FAM219A	Protein FAM219A OS=Homo sapiens GN=FA	1	110	90.2	92.1	92	100.1	92.05	0.91958042	0.285001
A0A024R0D2	hCG_38636	AP complex subunit beta OS=Homo sapiens G	1	128.7	76.4	99.4	89.2	102.55	94.3	0.919551438	0.28477
A0A024R5C4	RTN3	Reticulon OS=Homo sapiens GN=RTN3 PE=	5	109.4	101.8	97.7	96.5	105.6	97.1	0.919507576	0.28442
Q9H773	DCTPP1	dCTP pyrophosphatase 1 OS=Homo sapiens G	4	108.3	100.4	98	93.9	104.35	95.95	0.919501677	0.284373
O94804	STK10	Serine/threonine-protein kinase 10 OS=Homo	2	111	96.4	96.6	94.1	103.7	95.35	0.919479267	0.284194
A0A140TA35	VARS2	Valine--tRNA ligase, mitochondrial OS=Hom	3	99.4	97.6	89.3	91.8	98.5	90.55	0.91928934	0.282684
P56181	NDUFV3	NADH dehydrogenase [ubiquinone] flavoprot	1	97.4	90.9	90.2	82.9	94.15	86.55	0.919277748	0.282592
Q86X55	CARM1	Histone-arginine methyltransferase CARM1 O	1	106.3	105.5	100.5	94.2	105.9	97.35	0.919263456	0.282479
P61956	SUMO2	Small ubiquitin-related modifier 2 OS=Homo	2	100.7	102.4	93	93.7	101.55	93.35	0.9192516	0.282384
P00338	LDHA	L-lactate dehydrogenase A chain OS=Homo s	19	103.4	100.9	95.7	92.1	102.15	93.9	0.919236417	0.282264
Q8N680	ZBTB2	Zinc finger and BTB domain-containing prote	1	108.7	107.8	89.6	109.4	108.25	99.5	0.919168591	0.281726
Q14689	DIP2A	Disco-interacting protein 2 homolog A OS=H	1	99.5	111.9	93.9	100.4	105.7	97.15	0.919110691	0.281268
Q9UI30	TRMT112	Multifunctional methyltransferase subunit TR	7	102.6	100.1	92.8	93.5	101.35	93.15	0.919092255	0.281122
A0A140VK72		Testis secretory sperm-binding protein Li 199	3	113	96.9	98.2	94.7	104.95	96.45	0.919009052	0.280464
Q6PJI9	WDR59	WD repeat-containing protein 59 OS=Homo s	1	91	105.3	90.1	90.3	98.15	90.2	0.919001528	0.280404
O95714	HERC2	E3 ubiquitin-protein ligase HERC2 OS=Hom	6	106.4	102.1	96.9	94.7	104.25	95.8	0.918944844	0.279957
B7Z5V6		cDNA FLJ57046, highly similar to Lysosoma	1	108.5	106.9	107.8	90.1	107.7	98.95	0.918755803	0.278468
C9JIF9	APEH	Acylamino-acid-releasing enzyme OS=Homo	13	102.6	102.9	94	94.8	102.75	94.4	0.918734793	0.278302
O95155	UBE4B	Ubiquitin conjugation factor E4 B OS=Homo	8	108.1	102.3	98.3	95	105.2	96.65	0.918726236	0.278235
Q6P1X6	C8orf82	UPF0598 protein C8orf82 OS=Homo sapiens	6	102.4	104.3	94.4	95.5	103.35	94.95	0.918722787	0.278208
Q59EF6		Calpain 2, large [catalytic] subunit variant (Fr	12	107.9	100	94.2	96.8	103.95	95.5	0.918710919	0.278115
A0A024RD01	TBN	Taube nuss homolog (Mouse), isoform CRA_	1	99.6	109.5	107.8	84.3	104.55	96.05	0.918699187	0.278023
Q5VV89	MGST3	Microsomal glutathione S-transferase 3 OS=H	3	111.7	98.3	100.1	92.8	105	96.45	0.918571429	0.27702
B4DX01		cDNA FLJ59092 OS=Homo sapiens PE=2 SV	8	110.9	96.6	94.5	96.1	103.75	95.3	0.918554217	0.276885
Q9Y6D5	ARFGEF2	Brefeldin A-inhibited guanine nucleotide-exch	3	111.1	98.8	93.7	99.1	104.95	96.4	0.918532635	0.276716
A8KAK5		cDNA FLJ77399, highly similar to Homo sap	12	108.8	102.3	96.3	97.6	105.55	96.95	0.918522027	0.276633
Q9Y5S5	POLE1	DNA polymerase epsilon catalytic subunit pro	11	104.5	101.6	91.8	97.5	103.05	94.65	0.918486172	0.276352
B7ZW15		Uncharacterized protein OS=Homo sapiens PE	1	99.4	72.2	73.1	84.5	85.8	78.8	0.918414918	0.275795
A0A024RCR2	GNL1	Guanine nucleotide binding protein-like 1, iso	9	103.6	103.2	92.4	97.5	103.4	94.95	0.91827853	0.27473
Q9P0U4	CXXC1	CXXC-type zinc finger protein 1 OS=Homo s	3	111.1	96.9	96.8	94.2	104	95.5	0.918269231	0.274658
P29372	MPG	DNA-3-methyladenine glycosylase OS=Hom	6	103.7	106.5	98.5	94.5	105.1	96.5	0.918173168	0.27391
Q9P0U3	SENP1	Sentrin-specific protease 1 OS=Homo sapiens	1	105.2	101.3	104.6	85	103.25	94.8	0.918159806	0.273806
Q9UMP7	G4	G4 protein OS=Homo sapiens GN=G4 PE=2	1	109.2	103.1	92.7	102.2	106.15	97.45	0.918040509	0.272879
Q13315	ATM	Serine-protein kinase ATM OS=Homo sapien	1	102.3	112.2	92.9	104	107.25	98.45	0.917948718	0.272167
Q9UNW9	NOVA2	RNA-binding protein Nova-2 OS=Homo sapi	1	103.1	101.5	99	88.8	102.3	93.9	0.917888563	0.271701
P82979	SARNP	SAP domain-containing ribonucleoprotein OS	1	101.7	104	96.3	92.5	102.85	94.4	0.917841517	0.271337
Q9Y3L3	SH3BP1	SH3 domain-binding protein 1 OS=Homo sap	3	102.1	99.7	87.5	97.7	100.9	92.6	0.917740337	0.270555
Q13907	IDI1	Isopentenyl-diphosphate Delta-isomerase 1 O	5	104.9	99.3	93.5	93.9	102.1	93.7	0.917727718	0.270458
A8K8N7	PFAS	Phosphoribosylformylglycinamidine synthase	19	104	101.3	94.3	94.1	102.65	94.2	0.917681442	0.270101
P26583	HMGB2	High mobility group protein B2 OS=Homo sa	6	103.1	104.5	96.9	93.6	103.8	95.25	0.917630058	0.269705
A0A024QZE9	FLJ20643	Uncharacterized protein OS=Homo sapiens G	7	107.8	101	95.9	95.7	104.4	95.8	0.917624521	0.269662
B0YIW9	ARL5B	ADP-ribosylation factor-like 5B OS=Homo sa	1	102	106.8	89.2	102.4	104.4	95.8	0.917624521	0.269662
P48507	GCLM	Glutamate--cysteine ligase regulatory subunit	2	104.5	98	89.9	95.9	101.25	92.9	0.917530864	0.268941
Q9BWC9	CCDC106	Coiled-coil domain-containing protein 106 OS	2	100	101	92.8	91.6	100.5	92.2	0.917412935	0.268035
S4R347	FNBP1L	Formin-binding protein 1-like OS=Homo sapi	1	108	105.1	97.8	97.7	106.55	97.75	0.917409667	0.26801
H3BMV3	HN1L	Hematological and neurological-expressed 1-l	5	101	102.3	93	93.5	101.65	93.25	0.917363502	0.267656
A8K710		cDNA FLJ77428, highly similar to Homo sap	3	105.8	104.6	95.2	97.8	105.2	96.5	0.91730038	0.267173
Q5T5X7	BEND3	BEN domain-containing protein 3 OS=Homo	4	111.3	100.3	95.7	98.4	105.8	97.05	0.917296786	0.267145
F8VVA7	COPZ1	Coatomer subunit zeta-1 OS=Homo sapiens C	5	104.2	102.4	92.7	96.8	103.3	94.75	0.917231365	0.266645
Q8TDM6	DLG5	Disks large homolog 5 OS=Homo sapiens GN	1	100.4	103.6	86.6	100.5	102	93.55	0.917156863	0.266075
B2R9R2		cDNA, FLJ94517, highly similar to Homo sap	1	96.7	108.5	99.3	88.9	102.6	94.1	0.917153996	0.266053
Q6DKJ4	NXN	Nucleoredoxin OS=Homo sapiens GN=NXN	13	103.1	102	93.3	94.8	102.55	94.05	0.917113603	0.265745
Q96KB5	PBK	Lymphokine-activated killer T-cell-originated	9	100.7	101.9	93.1	92.7	101.3	92.9	0.917077986	0.265473
Q9H900	ZWILCH	Protein zwilch homolog OS=Homo sapiens G	1	105.9	101.5	93.6	96.6	103.7	95.1	0.917068467	0.265401
Q14653	IRF3	Interferon regulatory factor 3 OS=Homo sapie	1	101	99.1	90.8	92.7	100.05	91.75	0.917041479	0.265195
Q96GW9	MARS2	Methionine--tRNA ligase, mitochondrial OS=	3	109.6	100.1	95.5	96.8	104.85	96.15	0.91702432	0.265064

Q8TB96	ITFG1	T-cell immunomodulatory protein OS=Homo	1	103.1	114.7	93.6	106.1	108.9	99.85	0.916896235	0.26409
A0A0A0MSE:	HADH	Hydroxyacyl-coenzyme A dehydrogenase, mi	8	103.1	101.4	92.3	95.2	102.25	93.75	0.916870416	0.263894
A0A0A0MR5:	FADS1	Fatty acid desaturase 1 OS=Homo sapiens GN	5	106.8	101.3	97.7	93.1	104.05	95.4	0.916866891	0.263867
Q53F11		tRNA isopentenyltransferase 1 variant (Fragm	4	105.6	103.7	98	93.9	104.65	95.95	0.916865743	0.263858
Q9Y385	UBE2J1	Ubiquitin-conjugating enzyme E2 J1 OS=Hor	1	103.6	100.8	90.4	97	102.2	93.7	0.916829746	0.263585
Q9Y315	DERA	Deoxyribose-phosphate aldolase OS=Homo s:	3	100.7	106.1	97.2	92.4	103.4	94.8	0.916827853	0.26357
D3DU01	TMEM49	Transmembrane protein 49, isoform CRA_b (	2	113.5	99.2	102.7	92.3	106.35	97.5	0.916784203	0.26324
Q5VW36	FOCAD	Focadhesin OS=Homo sapiens GN=FOCAD I	2	102.7	102.4	91.6	96.4	102.55	94	0.916626036	0.262042
P00492	HPRT1	Hypoxanthine-guanine phosphoribosyltransfe	7	100.9	100.6	90.9	93.8	100.75	92.35	0.91662531	0.262036
G0XQ39	STIM1	STIM1L OS=Homo sapiens GN=STIM1 PE=	8	108.3	102.6	98.7	94.6	105.45	96.65	0.916548127	0.261453
Q14376	GALE	UDP-glucose 4-epimerase OS=Homo sapiens	7	101.7	104.3	93.9	94.9	103	94.4	0.916504854	0.261127
P23193	TCEA1	Transcription elongation factor A protein 1 O	13	103.5	103.6	94.4	95.4	103.55	94.9	0.916465476	0.260829
B2R8X4		cDNA, FLJ94105, highly similar to Homo sap	3	100	104.5	97.2	90.2	102.25	93.7	0.916381418	0.260196
Q2TB10	ZNF800	Zinc finger protein 800 OS=Homo sapiens GN	4	102.8	103.8	95.1	94.2	103.3	94.65	0.916263311	0.259308
B2RCD8		cDNA, FLJ96012, highly similar to Homo sap	6	106.4	101.9	92	98.8	104.15	95.4	0.915986558	0.257236
A0A024R807	SGK3	Serum/glucocorticoid regulated kinase family	1	100.8	99.1	93.3	89.8	99.95	91.55	0.915957979	0.257023
Q96BD5	PHF21A	PHD finger protein 21A OS=Homo sapiens G	2	104.2	98	93.7	91.5	101.1	92.6	0.915924827	0.256775
A8K940		cDNA FLJ77630, highly similar to Homo sap	1	102.1	100.1	85	100.2	101.1	92.6	0.915924827	0.256775
Q9H6H4	REEP4	Receptor expression-enhancing protein 4 OS=	3	111.3	99.2	96.5	96.3	105.25	96.4	0.915914488	0.256698
Q13905	RAPGEF1	Rap guanine nucleotide exchange factor 1 OS	1	107.1	81.9	82.4	90.7	94.5	86.55	0.915873016	0.256388
Q3ZCW2	LGALSL	Galectin-related protein OS=Homo sapiens G	1	109	107.3	102	96.1	108.15	99.05	0.915857605	0.256274
P32189	GK	Glycerol kinase OS=Homo sapiens GN=GK F	2	112.7	101.2	99	96.9	106.95	97.95	0.915848527	0.256206
E7ERK9	EIF2B4	Translation initiation factor eIF-2B subunit de	7	105.4	104.8	94.6	97.9	105.1	96.25	0.915794481	0.255804
B1ALW7	COG2	Component of oligomeric golgi complex 2, is	1	109.2	99.7	98.9	92.4	104.45	95.65	0.915749162	0.255466
O14777	NDC80	Kinetochore protein NDC80 homolog OS=Hc	1	101.8	103.4	95.9	92	102.6	93.95	0.915692008	0.255042
P52655	GTF2A1	Transcription initiation factor IIA subunit 1 O	3	106.1	97.4	93.5	92.8	101.75	93.15	0.915479115	0.253464
J3KR72	TAF6	Transcription initiation factor TFIID subunit 6	7	111.8	101.1	95.7	99.2	106.45	97.45	0.915453264	0.253273
Q5T7Y6	S100A1	Protein S100 OS=Homo sapiens GN=S100A1	1	105.1	94.6	85.6	97.2	99.85	91.4	0.91537306	0.25268
A0A024R5F7	DHCR7	7-dehydrocholesterol reductase isoform 1 OS=	6	102.5	99.5	93.2	91.7	101	92.45	0.915346535	0.252484
P49327	FASN	Fatty acid synthase OS=Homo sapiens GN=F.	87	101.9	103.6	94.1	94	102.75	94.05	0.915328467	0.252351
Q969F9	HPS3	Hermansky-Pudlak syndrome 3 protein OS=H	1	111	96.8	94.5	95.7	103.9	95.1	0.915303176	0.252164
A0A0K0K1J9	HEL-S-98	Epididymis secretory protein Li 98 OS=Homo	1	102.8	97.9	93.6	90.1	100.35	91.85	0.915296462	0.252115
O60504	SORBS3	Vinexin OS=Homo sapiens GN=SORBS3 PE	3	109	103.1	95.3	98.8	106.05	97.05	0.915134371	0.250922
Q5H9Q6	DKFZp781N0(	Inosine-5'-monophosphate dehydrogenase OS	4	112.1	98.8	92.1	100.9	105.45	96.5	0.915125652	0.250858
E7EW49	CLASP2	CLIP-associating protein 2 OS=Homo sapiens	7	103.5	103.8	97.5	92.2	103.65	94.85	0.91509889	0.250661
A0A024R1Z1	CDC34	Cell division cycle 34, isoform CRA_a OS=H	2	111.9	101.2	98.7	96.3	106.55	97.5	0.915063351	0.2504
Q8WUN7	UBTD2	Ubiquitin domain-containing protein 2 OS=H	1	110.1	97.1	90.9	98.7	103.6	94.8	0.915057915	0.25036
Q6NT16	SLC18B1	MFS-type transporter SLC18B1 OS=Homo sa	1	103.9	105.4	91.9	99.6	104.65	95.75	0.914954611	0.249603
Q9P2K8	EIF2AK4	eIF-2-alpha kinase GCN2 OS=Homo sapiens	1	109	103.7	100.3	94.3	106.35	97.3	0.91490362	0.24923
Q5TFE4	NT5DC1	5'-nucleotidase domain-containing protein 1 C	1	103.7	107.8	97.8	95.7	105.75	96.75	0.914893617	0.249157
V9GYM8	ARHGEF2	Rho guanine nucleotide exchange factor 2 OS	2	102.8	108.6	95.5	97.9	105.7	96.7	0.914853359	0.248862
A0A024RD93	PAICS	Phosphoribosylaminoimidazole carboxylase, p	19	108.6	99.2	95.7	94.4	103.9	95.05	0.914821944	0.248633
Q92830	KAT2A	Histone acetyltransferase KAT2A OS=Homo	5	107.7	103.5	98.7	94.5	105.6	96.6	0.914772727	0.248274
A0A087X1N8	SERPINB6	Serpin B6 OS=Homo sapiens GN=SERPINB(	15	102.7	102.6	94.2	93.6	102.65	93.9	0.914758889	0.248173
O60870	KIN	DNA/RNA-binding protein KIN17 OS=Homo	3	112.4	96.2	96.5	94.3	104.3	95.4	0.914669223	0.247519
O75427	LRCH4	Leucine-rich repeat and calponin homology d	2	112.7	93.5	93.6	95	103.1	94.3	0.914645975	0.24735
B2RAR2		cDNA, FLJ95064, highly similar to Homo sap	7	106.7	103	93.7	98.1	104.85	95.9	0.914639962	0.247306
X6REB3	TCF3	Transcription factor E2-alpha OS=Homo sapi	1	98.3	109	98.4	91.2	103.65	94.8	0.914616498	0.247136
A8K8D9		Glucose-6-phosphate 1-dehydrogenase OS=H	15	102	102.8	91.6	95.7	102.4	93.65	0.914550781	0.246658
F8WA39	MTMR1	Myotubularin-related protein 1 OS=Homo sap	1	111.6	102.1	94.5	100.9	106.85	97.7	0.914365934	0.245317
Q9BVQ7	SPATA5L1	Spermatogenesis-associated protein 5-like pro	2	107.3	96.8	90	96.6	102.05	93.3	0.914257717	0.244534
A0A0A0MTQ	CCDC175	Coiled-coil domain-containing protein 175 OS	2	114	107.5	104.9	97.6	110.75	101.25	0.914221219	0.244271
B2R6D8	CDC42EP4	CDC42 effector protein (Rho GTPase binding	1	103.7	103.8	90.3	99.4	103.75	94.85	0.914216867	0.24424
A8K4U6		cDNA FLJ77255, highly similar to Homo sap	1	112	83.7	93.5	85.4	97.85	89.45	0.914154318	0.243788
B3KPM8		cDNA FLJ31974 fis, clone NT2RP7008167, v	7	104.1	103	93.1	96.2	103.55	94.65	0.914051183	0.243046
O43708	GSTZ1	Maleylacetoacetate isomerase OS=Homo sapi	3	112.1	101.8	99.9	95.6	106.95	97.75	0.913978495	0.242523
B7Z532		cDNA FLJ51028, highly similar to 60 kDa he	1	128.7	86.3	99.3	97.2	107.5	98.25	0.913953488	0.242343
Q9UBI1	COMMD3	COMM domain-containing protein 3 OS=Hor	1	105.9	102.1	94.8	95.3	104	95.05	0.913942308	0.242263
L0R6P0	RPP14	Alternative protein RPP14 OS=Homo sapiens	1	106.7	102.3	87.7	103.3	104.5	95.5	0.913875598	0.241784
Q8N4C8	MINK1	Misshapen-like kinase 1 OS=Homo sapiens G	3	113.7	99.8	100.7	94.4	106.75	97.55	0.91381733	0.241367
A0A024R9R7	INOC1	INO80 complex homolog 1 (S. cerevisiae), is	1	100.3	99.1	90.4	91.8	99.7	91.1	0.913741224	0.240822
Q15911	ZFHX3	Zinc finger homeobox protein 3 OS=Homo sa	4	105.2	104.6	99.8	91.9	104.9	95.85	0.913727359	0.240723
Q86UK7	ZNF598	Zinc finger protein 598 OS=Homo sapiens GN	8	109.2	103.9	99.7	95	106.55	97.35	0.913655561	0.24021
Q9H8P0	SRD5A3	Polyprenol reductase OS=Homo sapiens GN=	2	105.1	95.2	95.9	87.1	100.15	91.5	0.913629556	0.240025
O00625	PIR	Pirin OS=Homo sapiens GN=PIR PE=1 SV=1	4	105.3	104.1	96.3	95	104.7	95.65	0.91356256	0.239547
Q8WXE0	CASKIN2	Caskin-2 OS=Homo sapiens GN=CASKIN2 I	1	107.3	104.2	92.3	100.9	105.75	96.6	0.913475177	0.238925
B4DQJ7		cDNA FLJ54836 OS=Homo sapiens PE=2 SV	1	100.3	106.5	87.8	101.1	103.4	94.45	0.91344294	0.238696
Q9NZM3	ITSN2	Intersectin-2 OS=Homo sapiens GN=ITSN2 I	11	112.9	99.4	95.3	98.6	106.15	96.95	0.913330193	0.237895
Q96RF0	SNX18	Sorting nexin-18 OS=Homo sapiens GN=SNX	1	96.2	104.3	99.7	83.4	100.25	91.55	0.913216958	0.237093
A8K5Q1		cDNA FLJ77548, highly similar to Homo sap	5	109.4	101.4	95.9	96.6	105.4	96.25	0.913187856	0.236887
O75911	DHRS3	Short-chain dehydrogenase/reductase 3 OS=H	1	103.7	101.3	90.9	96.3	102.5	93.6	0.913170732	0.236766
O60930	RNASEH1	Ribonuclease H1 OS=Homo sapiens GN=RN.	1	106.4	110.1	102.2	95.5	108.25	98.85	0.913163972	0.236718
Q8TCG5	CPT1C	Carnitine O-palmitoyltransferase 1, brain isof	3	110.5	102.5	95.7	98.8	106.5	97.25	0.91314554	0.236588
C7DJS2	GSTP1	Glutathione S-transferase pi (Fragment) OS=I	2	102.1	102.5	93.9	92.9	102.3	93.4	0.913000978	0.235568
Q5JTZ9	AARS2	Alanine--tRNA ligase, mitochondrial OS=Hor	9	107.6	101.3	94.7	96	104.45	95.35	0.912876975	0.234695
Q3LIB4	Nbla00360	Putative uncharacterized protein Nbla00360 C	4	104.5	101.9	94.1	94.3	103.2	94.2	0.912790698	0.23409
Q96F86	EDC3	Enhancer of mRNA-decapping protein 3 OS=	2	102.9	102.3	89	98.3	102.6	93.65	0.912768031	0.23393
P23511	NFYA	Nuclear transcription factor Y subunit alpha C	2	112.3	97.4	97.1	94.3	104.85	95.7	0.912732475	0.233681
D6RFG8	DCK	Deoxycytidine kinase OS=Homo sapiens GN=	3	105.7	106.1	97.1	96.2	105.9	96.65	0.912653447	0.233128
Q16512	PKN1	Serine/threonine-protein kinase N1 OS=Homo	2	103.4	104.9	89.4	100.7	104.15	95.05	0.91262602	0.232936
P51570	GALK1	Galactokinase OS=Homo sapiens GN=GALK	11	103.8	99.9	91.4	94.5	101.85	92.95	0.912616593	0.23287
Q9UIF8	BAZ2B	Bromodomain adjacent to zinc finger domain	1	107.8	103.8	93.2	99.9	105.8	96.55	0.912570888	0.232551
Q86UA3	C12orf10	Chromosome 12 open reading frame 10 OS=H	8	101.1	104.7	92.9	94.9	102.9	93.9	0.912536443	0.23231
P18858	LIG1	DNA ligase 1 OS=Homo sapiens GN=LIG1 F	4	105.7	101.2	92	96.8	103.45	94.4	0.912518125	0.232182
Q9H974	QTRT2	Queuine tRNA-ribosyltransferase accessory su	6	107.2	100.7	95.5	94.2	103.95	94.85	0.912457912	0.231762
P31153	MAT2A	S-adenosylmethionine synthase isoform type-	13	101.9	103.7	94.7	92.9	102.8	93.8	0.912451362	0.231717
Q9GZP4	PITHD1	PITH domain-containing protein 1 OS=Homo	5	102.9	104.8	93.5	96	103.85	94.75	0.912373616	0.231175
P67775	PPP2CA	Serine/threonine-protein phosphatase 2A catal	1	106.2	100.2	96.4	91.9	103.2	94.15	0.912306202	0.230707
Q2T9J0	TYSND1	Peroxisomal leader peptide-processing proteas	3	105.2	101	92.3	95.8	103.1	94.05	0.912221145	0.230116
B4DL97		cDNA FLJ56458, highly similar to Homo sap	2	107.1	102.3	92.4	98.6	104.7	95.5	0.912129895	0.229484
Q8N3Z6	ZCCHC7	Zinc finger CCHC domain-containing protein	2	109.2	92.1	88.2	95.4	100.65	91.8	0.912071535	0.22908
Q53YD8	hCG_23373	ADP-ribosylation factor-like 2 OS=Homo sap	7	102.1	104.8	95	93.7	103.45	94.35	0.912034799	0.228826
Q03393	PTS	6-pyruvoyl tetrahydrobiopterin synthase OS=I	1	97	107.5	96.4	90.1	102.25	93.25	0.91198044	0.228451
B2RBJ7		cDNA, FLJ95547, highly similar to Homo sap	1	103.4	106.2	96.3	94.8	104.8	95.55	0.911736641	0.226773
H0Y2P9	PIGX	Phosphatidylinositol-glycan biosynthesis class	2	103.3	100.6	93.3	92.6	101.95	92.95	0.911721432	0.226668
Q8N8Z6	DCBLD1	Discoidin, CUB and LCCL domain-containin	1	106.7	102.4	92.3	98.3	104.55	95.3	0.911525586	0.225326
Q96FL9	GALNT14	Polypeptide N-acetylgalactosaminyltransferas	1	115.4	95.9	93.8	98.8	105.65	96.3	0.911500237	0.225153
A0A0M4G3H:	AGL	Amylo-alpha-1, 6-glucosidase, 4-alpha-glucar	5	105.2	101.5	89.6	98.8	103.35	94.2	0.911465893	0.224918
A0A1B0GUM	PLEKHA7	Pleckstrin homology domain-containing famil	1	121.3	105.7	101.2	105.7	113.5	103.45	0.911453744	0.224835
E9PL57	NEDD8-MDP1	Protein NEDD8-MDP1 (Fragment) OS=Hom	3	107.5	100.3	94.7	94.7	103.9	94.7	0.911453321	0.224832
Q6QNY5	GNE	UDP-N-acetylglucosamine-2-epimerase / N-a	6	106.1	99.4	93.1	94.2	102.75	93.65	0.911435523	0.224711
A8K005		cDNA FLJ77896, highly similar to Homo sap	5	115.7	99.9	98.5	98	107.8	98.25	0.911410019	0.224537
A0A024R102	SMARCD1	SWI/SNF related, matrix associated, actin dep	4	100	111	97.2	95.1	105.5	96.15	0.911374408	0.224294
O43156	TTI1	TELO2-interacting protein 1 homolog OS=Hc	1	106.4	110	101.7	95.5	108.2	98.6	0.911275416	0.22362
Q9UL63	MKLN1	Muskelin OS=Homo sapiens GN=MKLN1 PI	7	114.1	99.9	96.1	98.9	107	97.5	0.911214953	0.223209
Q86U75		Dihydropyrimidinase-like 2 OS=Homo sapien	6	106	104.1	97.2	94.2	105.05	95.7	0.910994764	0.221716
B2R717		cDNA, FLJ93235, highly similar to Homo sap	2	98.6	103.2	90.9	92.9	100.9	91.9	0.910802775	0.22042
Q96GA3	LTV1	Protein LTV1 homolog OS=Homo sapiens GI	2	85	99.6	85.3	82.8	92.3	84.05	0.910617551	0.219175
E9PD50	SMG7	Protein SMG7 OS=Homo sapiens GN=SMG7	1	103.1	92.6	90.1	88.1	97.85	89.1	0.910577414	0.218906
Q9NZN5	ARHGEF12	Rho guanine nucleotide exchange factor 12 O	1	108.2	96.2	89.2	96.9	102.2	93.05	0.910469667	0.218184
A0A140VK27		Leukotriene A(4) hydrolase OS=Homo sapien	16	101.4	105.1	94.4	93.6	103.25	94	0.910411622	0.217796
A0A024R7X7	STAU2	Staufen, RNA binding protein, homolog 2 (Dr	3	106.7	102.6	95.8	94.7	104.65	95.25	0.91017678	0.216231
A0A169TED2	PRKCA	Protein kinase C OS=Homo sapiens GN=PRK	3	112.1	100.4	99.1	94.3	106.25	96.7	0.910117647	0.215838
Q53EN0		Zinc finger protein 330 variant (Fragment) OS	1	124.3	88.1	97.1	96.2	106.2	96.65	0.91007533	0.215557
Q14542	SLC29A2	Equilibrative nucleoside transporter 2 OS=Ho	1	111.1	88.9	93.3	88.7	100	91	0.91	0.215058
P10244	MYBL2	Myb-related protein B OS=Homo sapiens GN	1	108.6	106.5	95.5	100.2	107.55	97.85	0.909809391	0.213798
Q05DJ2	MTAP	Purine nucleoside phosphorylase OS=Homo s	10	105.3	104.1	94.5	96	104.7	95.25	0.90974212	0.213354
Q9BXW9	FANCD2	Fanconi anemia group D2 protein OS=Homo	2	109.7	100.8	94.5	97	105.25	95.75	0.909738717	0.213332
P23921	RRM1	Ribonucleoside-diphosphate reductase large s	26	103.3	102.6	93.6	93.7	102.95	93.65	0.909664886	0.212846
Q8TBX8	PIP4K2C	Phosphatidylinositol 5-phosphate 4-kinase typ	8	110.9	104.8	98.7	97.5	107.85	98.1	0.909596662	0.212398
Q9H7E9	C8orf33	UPF0488 protein C8orf33 OS=Homo sapiens	3	108.6	100.9	92.9	97.6	104.75	95.25	0.909307876	0.210507
Q9NZQ3	NCKIPSD	NCK-interacting protein with SH3 domain OS	2	107.8	92.7	92.5	89.8	100.25	91.15	0.909226933	0.209979

A0A0G2JK44	BRD2	Bromodomain-containing protein 2 OS=Homo	3	98.2	104.5	91.7	92.6	101.35	92.15	0.909225456	0.209969
B3KTN5		cDNA FLJ38538 fis, clone HCHON2001407,	3	104.1	105.2	98.7	91.6	104.65	95.15	0.909221214	0.209942
Q16514	TAF12	Transcription initiation factor TFIID subunit 1	1	121.5	99.5	94.3	106.6	110.5	100.45	0.909049774	0.208827
A7LFP5	MANBA	Beta-mannosidase OS=Homo sapiens GN=M.	6	107.2	103.8	96.2	95.6	105.5	95.9	0.909004739	0.208535
Q14739	LBR	Lamin-B receptor OS=Homo sapiens GN=LB	1	114.7	103.8	103.5	95.1	109.25	99.3	0.908924485	0.208014
B4DYZ7		cDNA FLJ54316, highly similar to DNA poly	2	98.3	114.6	97.5	96	106.45	96.75	0.908877407	0.20771
A0A024R2M7	OXSR1	Oxidative-stress responsive 1, isoform CRA_a	7	109.2	103.6	96.1	97.3	106.4	96.7	0.908834586	0.207433
B2RDT8		cDNA, FLJ96764, highly similar to Homo sap	5	107	101.4	94.4	95	104.2	94.7	0.908829175	0.207398
B4E218		cDNA FLJ61052, highly similar to Homo sap	2	106.7	89.4	84.5	93.7	98.05	89.1	0.908720041	0.206694
B4DN80		Peptidyl-prolyl cis-trans isomerase OS=Homo	4	101.2	99	90.5	91.4	100.1	90.95	0.908591409	0.205866
G8IFA7		p90 OS=Homo sapiens PE=2 SV=1	2	101.8	109.3	96.8	95	105.55	95.9	0.908574135	0.205755
Q8TBP6	SLC25A40	Solute carrier family 25 member 40 OS=Hom	1	115.3	97.3	97.1	96	106.3	96.55	0.908278457	0.203861
Q3KRA6	C2orf76	UPF0538 protein C2orf76 OS=Homo sapiens	1	106.7	98.2	88.8	97.3	102.45	93.05	0.908247926	0.203666
Q562Q6	ACT	Actin-like protein (Fragment) OS=Homo sapi	1	101.5	107.7	100.8	89.2	104.6	95	0.908221797	0.2035
Q9Y448	KNSTRN	Small kinetochore-associated protein OS=Ho	5	108.1	106.4	98.9	95.9	107.25	97.4	0.908158508	0.203096
A7MD06	ZNF483	Zinc finger protein 483 OS=Homo sapiens GN	1	104.9	108.5	90.9	102.9	106.7	96.9	0.908153702	0.203066
B3KTR4		cDNA FLJ38643 fis, clone HHDPC2005513,	1	104.2	107.8	97.8	94.7	106	96.25	0.908018868	0.202209
F8WEC0	MMADHC	Methylmalonic aciduria and homocystinuria t	2	115.3	107.2	101.6	100.4	111.25	101	0.907865169	0.201234
B7ZKS3	USP48	Ubiquitin specific peptidase 48 OS=Homo sap	5	109.8	101.6	97.9	94	105.7	95.95	0.907757085	0.200556
Q9HCK8	CHD8	Chromodomain-helicase-DNA-binding protei	9	106.1	106.1	97.3	95.3	106.1	96.3	0.907634307	0.199777
A0A024R3V8	TSNAX	Translin-associated factor X, isoform CRA_c	7	112.5	100.7	95.7	97.8	106.6	96.75	0.907598499	0.199552
A2RRD8	ZNF320	Zinc finger protein 320 OS=Homo sapiens GN	2	116.5	96.2	94.2	98.8	106.35	96.5	0.907381288	0.198189
Q7Z4G4	TRMT11	tRNA (guanine(10)-N2)-methyltransferase ho	2	102.7	102.1	92.2	93.6	102.4	92.9	0.907226563	0.197222
A0A024R577	TM7SF2	Transmembrane 7 superfamily member 2, iso	2	102.2	100.3	92	91.7	101.25	91.85	0.907160494	0.19681
B3KX63	MUS81	MUS81 endonuclease homolog (Yeast), isofo	1	113.4	96.2	80.7	109.4	104.8	95.05	0.906965649	0.195599
A8K9U1		cDNA FLJ76468, highly similar to Homo sap	2	96.9	96.4	86.8	88.5	96.65	87.65	0.906880497	0.195071
A8K9K0		cDNA FLJ78032, highly similar to Homo sap	1	106.6	102.7	90.1	99.7	104.65	94.9	0.906832298	0.194773
Q53HR4		Chromosome 15 open reading frame 22 variar	4	111.6	102.7	95.4	98.9	107.15	97.15	0.906672888	0.193789
E9PL17	CLP1	Polyribonucleotide 5'-hydroxyl-kinase Clp1 C	3	110.5	103.7	99.1	95.1	107.1	97.1	0.906629318	0.19352
Q9BYG5	PARD6B	Partitioning defective 6 homolog beta OS=Ho	3	104.2	108.9	95.6	97.6	106.55	96.6	0.906616612	0.193442
Q9BTY2	FUCA2	Plasma alpha-L-fucosidase OS=Homo sapiens	5	109.2	102.7	96.8	95.3	105.95	96.05	0.906559698	0.193092
O75386	TULP3	Tubby-related protein 3 OS=Homo sapiens G	1	107.3	94.9	88.8	94.5	101.1	91.65	0.90652819	0.192898
Q15545	TAF7	Transcription initiation factor TFIID subunit 7	2	110.6	108.2	100	98.3	109.4	99.15	0.90630713	0.191543
A0A024RD28	C6orf139	Chromosome 6 open reading frame 139, isofo	2	117.8	98.6	99.1	97	108.2	98.05	0.906192237	0.190842
P52434	POLR2H	DNA-directed RNA polymerases I, II, and III	5	108	102.9	96.2	94.9	105.45	95.55	0.906116643	0.190382
O75506	HSBP1	Heat shock factor-binding protein 1 OS=Hom	2	108.4	101	93.9	95.8	104.7	94.85	0.905921681	0.189198
Q9BW60	ELOVL1	Elongation of very long chain fatty acids prot	3	97.5	111.7	97.1	92.4	104.6	94.75	0.90583174	0.188653
Q6ZMY0		cDNA FLJ16598 fis, clone TESTI4006473, w	1	117.8	96.7	99.7	94.6	107.25	97.15	0.905827506	0.188628
B3KSZ4		cDNA FLJ37346 fis, clone BRAMY2021310,	10	110	103.2	98.2	94.9	106.6	96.55	0.905722326	0.187992
Q01968	OCRL	Inositol polyphosphate 5-phosphatase OCRL-	2	104.1	112	100.8	94.8	108.05	97.8	0.905136511	0.184481
P15121	AKR1B1	Aldose reductase OS=Homo sapiens GN=AK	11	102.3	102.2	93.6	91.5	102.25	92.55	0.905134474	0.184469
Q96GE9	TMEM261	Transmembrane protein 261 OS=Homo sapie	1	115.9	110.7	103.3	101.8	113.3	102.55	0.905119153	0.184378
Q7L3B6	CDC37L1	Hsp90 co-chaperone Cdc37-like 1 OS=Homo	2	111.1	106	99.2	97.3	108.55	98.25	0.905112851	0.18434
B3KSP2		cDNA FLJ36730 fis, clone UTERU2012610,	1	120.8	98.3	103.2	95.1	109.55	99.15	0.90506618	0.184063
A0A024R6D1	NEK9	NIMA (Never in mitosis gene a)-related kinas	2	107	98.4	91.8	94.1	102.7	92.95	0.905063291	0.184045
Q9UJ83	HACL1	2-hydroxyacyl-CoA lyase 1 OS=Homo sapier	3	113.2	101.3	97.9	96.2	107.25	97.05	0.904895105	0.183048
Q8WUR7	C15orf40	UPF0235 protein C15orf40 OS=Homo sapien	1	116.6	93.3	96.1	93.8	104.95	94.95	0.904716532	0.181993
A0A024R101	SPATS2	Spermatogenesis associated, serine-rich 2, iso	3	118.8	98.4	98.9	97.6	108.6	98.25	0.904696133	0.181872
Q6P1M3	LLGL2	Lethal(2) giant larvae protein homolog 2 OS=	5	110.3	102.7	97.2	95.5	106.5	96.35	0.904694836	0.181865
Q9BY42	RTFDC1	Protein RTF2 homolog OS=Homo sapiens GN	4	107.7	109.3	100.8	95.5	108.5	98.15	0.904608295	0.181355
B5BU41	CAMK1	Calcium/calmodulin-dependent protein kinase	5	105.2	99.2	91.7	93.2	102.2	92.45	0.904598826	0.1813
A0A024RB03	KIAA0528	KIAA0528, isoform CRA_b OS=Homo sapie	2	104	106.6	95.6	94.9	105.3	95.25	0.904558405	0.181062
A0A0H4IV28		CK20 OS=Homo sapiens PE=2 SV=1	1	107.6	100.9	95.8	92.8	104.25	94.3	0.904556355	0.18105
Q96JH7	VCPIP1	Deubiquitinating protein VCIP135 OS=Homo	1	97.3	107	98.9	85.9	102.15	92.4	0.904552129	0.181025
B7ZKJ3	EPHA5	EPH receptor A5 OS=Homo sapiens GN=EPI	1	111.3	100.2	90.2	101.1	105.75	95.65	0.904491726	0.180671
P51812	RPS6KA3	Ribosomal protein S6 kinase alpha-3 OS=Hor	3	99.3	110	94.5	94.8	104.65	94.65	0.904443383	0.180387
Q9UJX4	ANAPC5	Anaphase-promoting complex subunit 5 OS=I	8	109.3	103	100	92	106.15	96	0.904380593	0.18002
Q86UB9	TMEM135	Transmembrane protein 135 OS=Homo sapie	5	105.8	104.4	93.7	96.4	105.1	95.05	0.904376784	0.179997
Q9NYG5	ANAPC11	Anaphase-promoting complex subunit 11 OS=	1	112.1	105.3	97.1	99.5	108.7	98.3	0.904323827	0.179687
Q9NRG7	SDR39U1	Epimerase family protein SDR39U1 OS=Hon	1	115.6	95.5	97.7	93.2	105.55	95.45	0.904310753	0.179611
F5H872	MED21	Mediator of RNA polymerase II transcription	2	110.2	101.8	95.6	96.1	106	95.85	0.904245283	0.179229
P31150	GDI1	Rab GDP dissociation inhibitor alpha OS=Ho	8	108.3	101.5	94.4	95.3	104.9	94.85	0.904194471	0.178933
Q59FC3		G protein-coupled receptor kinase interactor 1	2	96	103.3	85.5	94.7	99.65	90.1	0.904164576	0.178758
Q6F5E8	CARMIL2	Capping protein, Arp2/3 and myosin-I linker	1	100.7	112.1	97.2	95.2	106.4	96.2	0.904135338	0.178589
Q6AWC2	WWC2	Protein WWC2 OS=Homo sapiens GN=WWC	1	106.9	99.6	82.4	104.3	103.25	93.35	0.904116223	0.178477
Q4LE69	PIK4CA	PIK4CA variant protein (Fragment) OS=Hom	2	106.6	103	91.8	97.7	104.8	94.75	0.904103053	0.178401
Q5T2E6	C10orf76	UPF0668 protein C10orf76 OS=Homo sapien	1	107.8	104.6	92.6	99.4	106.2	96	0.903954802	0.17754
Q9NVA4	TMEM184C	Transmembrane protein 184C OS=Homo sapi	1	106.1	96.8	93.7	89.7	101.45	91.7	0.903893544	0.177186
A0A0C4DFM.	ZNF574	HCG1643764, isoform CRA_b OS=Homo sa	1	98.9	99.8	88.7	90.9	99.35	89.8	0.903875189	0.177079
B2RAR3	QTRT1	Queuine tRNA-ribosyltransferase catalytic su	5	105.5	103.5	92.8	96.1	104.5	94.45	0.903827751	0.176805
B4DDQ8		cDNA FLJ50439, highly similar to Phosphogl	1	101.6	109.2	92.3	98.2	105.4	95.25	0.90370019	0.176069
A0A024R9T3	GUF1	Translation factor GUF1, mitochondrial OS=I	1	108.6	101.1	96.6	92.9	104.85	94.75	0.903671912	0.175907
A0A0D2X7Z3	CRY2	Cryptochrome-2 OS=Homo sapiens GN=CRY	1	112.3	100	91.1	100.7	106.15	95.9	0.90343853	0.174567
Q8WTW3	COG1	Conserved oligomeric Golgi complex subunit	4	106.7	103.4	93.8	96	105.05	94.9	0.903379343	0.174228
Q9UJ68	MSRA	Mitochondrial peptide methionine sulfoxide re	1	109.1	106.1	102.2	92.2	107.6	97.2	0.903345725	0.174036
A0A024RA36	KIAA0241	KIAA0241, isoform CRA_b OS=Homo sapie	1	115.7	102.6	99.3	97.9	109.15	98.6	0.903344022	0.174027
Q8IUH3	RBM45	RNA-binding protein 45 OS=Homo sapiens C	2	105.9	105	92.4	98.1	105.45	95.25	0.903271693	0.173614
Q8WV60	PTCD2	Pentatricopeptide repeat-containing protein 2,	3	108.5	103.4	92.9	98.5	105.95	95.7	0.903256253	0.173526
J3KTI2	MIEN1	Migration and invasion enhancer 1 OS=Homo	1	104.8	103.9	92.1	96.4	104.35	94.25	0.90321035	0.173264
O14832	PHYH	Phytanoyl-CoA dioxygenase, peroxisomal OS	4	107	103.7	96.1	94.2	105.35	95.15	0.903179877	0.173091
Q8WU79	SMAP2	Stromal membrane-associated protein 2 OS=I	1	122.1	94.6	90.6	105.1	108.35	97.85	0.903091832	0.17259
Q9UJ70	NAGK	N-acetyl-D-glucosamine kinase OS=Homo sa	4	106.1	102.2	92.1	96	104.15	94.05	0.903024484	0.172208
P16402	HIST1H1D	Histone H1.3 OS=Homo sapiens GN=HIST1I	8	100.3	103.8	92	92.3	102.05	92.15	0.902988731	0.172005
Q9H1B7	IRF2BPL	Interferon regulatory factor 2-binding protein-	6	109.6	106.3	98.1	96.8	107.95	97.45	0.902732747	0.17056
Q6YHU6	THADA	Thyroid adenoma-associated protein OS=Hon	1	109.1	112.8	101.9	98.4	110.95	100.15	0.902658855	0.170144
Q9BRQ0	PYGO2	Pygopus homolog 2 OS=Homo sapiens GN=I	1	110.6	101	97.5	93.5	105.8	95.5	0.902646503	0.170075
Q86Y91	KIF18B	Kinesin-like protein KIF18B OS=Homo sapie	1	105.6	100.6	93.5	92.6	103.1	93.05	0.902521823	0.169375
Q9H2M9	RAB3GAP2	Rab3 GTPase-activating protein non-catalytic	14	103.7	107.5	96	94.6	105.6	95.3	0.902462121	0.169041
O60658	PDE8A	High affinity cAMP-specific and IBMX-inser	2	103.3	108.9	98.2	93.3	106.1	95.75	0.902450518	0.168976
Q3KQZ1	SLC25A35	Solute carrier family 25 member 35 OS=Hom	4	101.8	104.1	93	92.8	102.95	92.9	0.902379796	0.168581
A0A024R096	C1orf119	Protein kish OS=Homo sapiens GN=C1orf119	1	112.3	102.7	101.6	92.4	107.5	97	0.902325581	0.168278
Q8TCE6	FAM45A	Protein FAM45A OS=Homo sapiens GN=FA	2	104.1	103.5	90.5	96.8	103.8	93.65	0.9022158	0.167667
Q9H0B6	KLC2	Kinesin light chain 2 OS=Homo sapiens GN=	11	112.1	99.5	94.6	96.3	105.8	95.45	0.902173913	0.167434
Q92793	CREBBP	CREB-binding protein OS=Homo sapiens GN	3	106.4	105.1	94.2	96.6	105.75	95.4	0.90212766	0.167177
A5PLM9	CTSL1	Cathepsin L1 OS=Homo sapiens GN=CTSL1	3	110.1	103.2	98.6	93.8	106.65	96.2	0.90201594	0.166558
A8K228		cDNA FLJ77874, highly similar to Homo sap	1	99	81.4	83.5	79.2	90.2	81.35	0.901884701	0.165832
D9IAI1	PEBP1	Epididymis secretory protein Li 34 OS=Homo	8	104.3	101.3	93.1	92.3	102.8	92.7	0.901750973	0.165096
Q8IUI8	CRLF3	Cytokine receptor-like factor 3 OS=Homo sap	2	102.7	103.9	93.4	92.9	103.3	93.15	0.901742498	0.165049
P27482	CALML3	Calmodulin-like protein 3 OS=Homo sapiens	1	113.3	103.3	84.9	110.4	108.3	97.65	0.90166205	0.164607
Q9BWH6	RPAP1	RNA polymerase II-associated protein 1 OS=	4	108.7	98.6	89.4	97.5	103.65	93.45	0.901591896	0.164222
Q9UBB4	ATXN10	Ataxin-10 OS=Homo sapiens GN=ATXN10 I	11	105.6	104.6	95.6	93.9	105.1	94.75	0.90152236	0.163842
A4ZI32		Fanconi anemia core complex 100 kDa subun	1	108.6	100.5	91.1	97.4	104.55	94.25	0.901482544	0.163624
Q9BU89	DOHH	Deoxyhypusine hydroxylase OS=Homo sapie	5	111	100.1	96.2	94.1	105.55	95.15	0.901468498	0.163547
A8K1K8	C18orf55	Chromosome 18 open reading frame 55, isofo	1	114.7	102.2	92.2	103.3	108.45	97.75	0.901337022	0.16283
A8K9X5		cDNA FLJ76472, highly similar to Homo sap	4	106.8	102.9	90.5	98.5	104.85	94.5	0.901287554	0.162561
A0A090N7T9	SCRN1	Secernin 1 OS=Homo sapiens GN=SCRN1 PI	8	111.9	98.7	95.5	94.3	105.3	94.9	0.901234568	0.162273
Q96KG9	SCYL1	N-terminal kinase-like protein OS=Homo sap	5	112	101.6	95.2	97.3	106.8	96.25	0.901217228	0.162179
B2RCN8		cDNA, FLJ96179, highly similar to Homo sap	1	116.2	94.2	89.4	100.2	105.2	94.8	0.901140684	0.161763
Q9Y6K9	IKBKG	NF-kappa-B essential modulator OS=Homo s	3	110.3	100.1	96.8	92.8	105.2	94.8	0.901140684	0.161763
Q9HCM3	KIAA1549	UPF0606 protein KIAA1549 OS=Homo sapie	2	109.2	94	89.2	93.9	101.6	91.55	0.901082677	0.161449
A8K8W4		cDNA FLJ77609, highly similar to Homo sap	4	122.9	95.4	96.6	100.1	109.15	98.35	0.901053596	0.161292
E9PK54	HSPA8	Heat shock cognate 71 kDa protein (Fragment	1	111.9	100.1	92.9	98.1	106	95.5	0.900943396	0.160697
B7ZA12		cDNA, FLJ79026, highly similar to Dual spec	1	106.7	104.1	101	88.9	105.4	94.95	0.90085389	0.160214
Q9H0V1	TMEM168	Transmembrane protein 168 OS=Homo sapie	1	102.5	107.1	93.1	95.7	104.8	94.4	0.900763359	0.159727
B4DRL5		cDNA FLJ60834, highly similar to Engulfme	2	111.4	99.8	96.5	93.7	105.6	95.1	0.900568182	0.158682
Q15018	FAM175B	BRISC complex subunit Abro1 OS=Homo sa	1	103.9	97.2	91.1	90	100.55	90.55	0.900546992	0.158568
P23677	ITPKA	Inositol-trisphosphate 3-kinase A OS=Homo s	4	110.7	99.4	95.2	94	105.05	94.6	0.90052356	0.158443
A0A024R6R1	SHCBP1	SHC SH2-domain binding protein 1, isoform	9	119.4	94.5	95.5	97.1	106.95	96.3	0.900420757	0.157895
Q9NY64	SLC2A8	Solute carrier family 2, facilitated glucose tra	1	108.4	105.4	96.1	96.4	106.9	96.25	0.900374181	0.157647
Q7Z2K6	ERMP1	Endoplasmic reticulum metallopeptidase 1 OS	17	106.3	102.1	94	93.6	104.2	93.8	0.900191939	0.15668
Q2TU64		T-complex protein 1 subunit gamma OS=Hon	1	114.3	98	98.4	92.7	106.15	95.55	0.900141309	0.156412
J3QQX2	ARHGDIA	Rho GDP-dissociation inhibitor 1 OS=Homo	8	101.1	107.1	95.6	91.8	104.1	93.7	0.900096061	0.156172

B3KS36		cDNA FLJ35376 fis, clone SKMUS2004044,	1	114.4	97.8	105.2	85.8	106.1	95.5	0.900094251	0.156163
Q96TA1	FAM129B	Niban-like protein 1 OS=Homo sapiens GN=I	3	101.8	99.2	90.6	90.3	100.5	90.45	0.9	0.155665
A0A024R1Q5	NAGA	N-acetylgalactosaminidase, alpha-, isoform C	5	102	102.9	89.9	94.5	102.45	92.2	0.899951196	0.155408
O95864	FADS2	Fatty acid desaturase 2 OS=Homo sapiens GN	9	102.4	105.4	92.5	94.5	103.9	93.5	0.899903754	0.155159
P49641	MAN2A2	Alpha-mannosidase 2x OS=Homo sapiens GN	3	107	97.8	89.6	94.7	102.4	92.15	0.899902344	0.155151
O75330	HMMR	Hyaluronan mediated motility receptor OS=H	1	115.4	98.6	100.6	91.9	107	96.25	0.89953271	0.153216
O95503	CBX6	Chromobox protein homolog 6 OS=Homo sap	1	119.7	96.2	88.2	106	107.95	97.1	0.899490505	0.152996
Q9P1F3	ABRACL	Costars family protein ABRACL OS=Homo s	1	102.8	110.1	100.4	91.1	106.45	95.75	0.899483326	0.152959
Q5T280	C9orf114	Putative methyltransferase C9orf114 OS=Hor	5	106.2	103.7	94.2	94.6	104.95	94.4	0.899475941	0.152921
P16930	FAH	Fumarylacetoacetase OS=Homo sapiens GN=	2	101.7	100	92.6	88.8	100.85	90.7	0.899355478	0.152295
Q5T440	IBA57	Putative transferase CAF17, mitochondrial O	3	107.1	107.3	99.9	92.9	107.2	96.4	0.899253731	0.151767
B3KSP9		cDNA FLJ36765 fis, clone 3NB691000191 O	2	107.1	106.1	96.2	95.5	106.6	95.85	0.899155722	0.151261
P50583	NUDT2	Bis(5'-nucleosyl)-tetraphosphatase [asymmetr	2	101.5	102.7	89.7	93.9	102.1	91.8	0.899118511	0.151069
E5KTI5	NTHL1	Endonuclease III-like protein 1 OS=Homo sap	1	106.6	108	93.4	99.5	107.3	96.45	0.89888164	0.149851
A0A024QZ86	TBX2	T-box 2, isoform CRA_a OS=Homo sapiens C	3	104.7	100	94.4	89.6	102.35	92	0.898876404	0.149824
O00767	SCD	Acyl-CoA desaturase OS=Homo sapiens GN=	3	106.3	100.3	90.6	95.1	103.3	92.85	0.898838335	0.149629
P36405	ARL3	ADP-ribosylation factor-like protein 3 OS=Ho	10	102.6	103	92.1	92.7	102.8	92.4	0.898832685	0.1496
O95630	STAMBP	STAM-binding protein OS=Homo sapiens GN	2	109.2	107	105	89.3	108.1	97.15	0.898704903	0.148946
B4E2A6		cDNA FLJ55508, highly similar to Sad1/unc-	10	110	98.2	90.6	96.5	104.1	93.55	0.898655139	0.148693
A8K4M5		cDNA FLJ76043, highly similar to Homo sap	4	105.3	112.7	98	97.9	109	97.95	0.898623853	0.148533
J3KR35	CCDC12	Coiled-coil domain containing 12, isoform CF	6	114.1	100.8	98.5	94.6	107.45	96.55	0.898557469	0.148195
Q96QZ7	MAGI1	Membrane-associated guanylate kinase, WW	1	109.9	103.9	97.5	94.6	106.9	96.05	0.898503274	0.14792
P0CG22	DHRS4L1	Putative dehydrogenase/reductase SDR family	2	112.3	102.3	91.5	101.3	107.3	96.4	0.898415657	0.147475
A0A024RAJ4	POLR2D	Polymerase (RNA) II (DNA directed) polypep	6	110.3	100.9	94.6	95.1	105.6	94.85	0.898200758	0.146389
O75446	SAP30	Histone deacetylase complex subunit SAP30 (	2	104.5	102.9	91.4	94.8	103.7	93.1	0.897782064	0.14429
O75354	ENTPD6	Ectonucleoside triphosphate diphosphohydrol	1	109.5	103.6	92.7	98.6	106.55	95.65	0.89770061	0.143884
P58546	MTPN	Myotrophin OS=Homo sapiens GN=MTPN P	5	110.9	101.1	92.1	98.2	106	95.15	0.897641509	0.14359
B7ZC38	SH3GLB2	Endophilin-B2 OS=Homo sapiens GN=SH3G	5	107.5	100.5	94.6	92.1	104	93.35	0.897596154	0.143365
F8W7C6	RPL10	60S ribosomal protein L10 OS=Homo sapiens	1	113.9	96.7	95.6	93.4	105.3	94.5	0.897435897	0.142571
P30520	ADSS	Adenylosuccinate synthetase isozyme 2 OS=H	11	105.3	100.3	92.4	92.1	102.8	92.25	0.897373541	0.142263
Q53FP0		Pyridoxine 5'-phosphate oxidase variant (Frag	2	111.9	100.4	92.8	97.7	106.15	95.25	0.89731512	0.141975
Q86X76	NIT1	Nitrilase homolog 1 OS=Homo sapiens GN=N	4	104.3	101.9	92.2	92.8	103.1	92.5	0.897187197	0.141345
G9LHJ5	COX2	Cytochrome c oxidase subunit 2 OS=Homo sa	1	110.6	91.5	95.2	86.1	101.05	90.65	0.897080653	0.140823
Q8NI22	MCFD2	Multiple coagulation factor deficiency protein	1	129.6	84.1	100.7	91	106.85	95.85	0.897051942	0.140682
Q8NCR1	VPS53	VPS53 protein OS=Homo sapiens GN=VPS53	2	109	106	95.1	97.7	107.5	96.4	0.896744186	0.139182
A0A024R869	GOLGA1	Golgi autoantigen, golgin subfamily a, 1, isof	6	111.3	98.8	92.9	95.5	105.05	94.2	0.89671585	0.139044
Q9BRJ6	C7orf50	Uncharacterized protein C7orf50 OS=Homo s	3	102.9	104.2	93.8	91.9	103.55	92.85	0.896668276	0.138813
Q2TAA5	ALG11	GDP-Man:Man(3)GlcNAc(2)-PP-Dol alpha-1	1	100.6	93.5	89.8	84.2	97.05	87	0.896445131	0.137735
E5RIM7	ATOX1	Copper transport protein ATOX1 OS=Homo s	3	107.3	101.2	90.1	96.8	104.25	93.45	0.896402878	0.137531
Q5R3I4	TTC38	Tetratricopeptide repeat protein 38 OS=Homo	3	110.8	100.5	91.9	97.5	105.65	94.7	0.896355892	0.137305
A1L0T0	ILVBL	Acetolactate synthase-like protein OS=Homo	15	112.9	101.2	94	97.9	107.05	95.95	0.896310135	0.137085
Q86X29	LSR	Lipolysis-stimulated lipoprotein receptor OS=	4	109.8	107.1	95.7	98.7	108.45	97.2	0.89626556	0.136871
Q5JU69	TOR2A	Torsin-2A OS=Homo sapiens GN=TOR2A P	1	101.7	107.3	94.6	92.7	104.5	93.65	0.896172249	0.136424
P20226	TBP	TATA-box-binding protein OS=Homo sapien	1	111.1	108.9	108.2	88.9	110	98.55	0.895909091	0.135169
A0A090N8Q3	C7orf21	Chromosome 7 open reading frame 21 OS=Ho	3	107.8	107	95.3	97.1	107.4	96.2	0.895716946	0.134258
Q9H2P9	DPH5	Diphthine methyl ester synthase OS=Homo sa	2	100.7	105.3	91	93.5	103	92.25	0.895631068	0.133852
Q6UWP7	LCLAT1	Lysocardiolipin acyltransferase 1 OS=Homo s	8	111.1	99.5	95.9	92.7	105.3	94.3	0.895536562	0.133407
A0A087X1W2	PRMT1	Protein arginine N-methyltransferase 1 OS=H	1	109.7	100.8	96.6	91.9	105.25	94.25	0.895486936	0.133174
B8ZZN6	SUMO1	Small ubiquitin-related modifier 1 OS=Homo	4	110.2	102.9	98	92.8	106.55	95.4	0.895354294	0.132551
O14530	TXNDC9	Thioredoxin domain-containing protein 9 OS=	1	111	101.1	86.7	103.2	106.05	94.95	0.89533239	0.132449
V9HWH1	HEL57	Epididymis luminal protein 57 OS=Homo sap	8	105.9	102.2	93.7	92.6	104.05	93.15	0.895242672	0.132029
P23443	RPS6KB1	Ribosomal protein S6 kinase beta-1 OS=Hom	2	114.5	97.7	93.7	96.2	106.1	94.95	0.894910462	0.130484
V9HWC4	HEL-S-132P	Epididymis secretory sperm binding protein L	2	113.1	98	90.1	98.8	105.55	94.45	0.89483657	0.130142
O00468	AGRN	Agrin OS=Homo sapiens GN=AGRN PE=1 S	5	107	103	91.1	96.8	105	93.95	0.894761905	0.129798
Q9Y6M4	CSNK1G3	Casein kinase I isoform gamma-3 OS=Homo	1	111.9	98	98.8	89	104.95	93.9	0.894711768	0.129567
P48059	LIMS1	LIM and senescent cell antigen-like-containin	1	108.5	102.8	96.7	92.3	105.65	94.5	0.894462849	0.128424
Q9UKJ3	GPATCH8	G patch domain-containing protein 8 OS=Hor	1	105.5	101	101	83.7	103.25	92.35	0.894430993	0.128278
A0A0A0MT33	SCAF8	Protein SCAF8 OS=Homo sapiens GN=SCAI	1	103.1	102.4	84.9	98.9	102.75	91.9	0.894403893	0.128154
P11171	EPB41	Protein 4.1 OS=Homo sapiens GN=EPB41 PI	2	112.6	99.4	90.2	99.4	106	94.8	0.894339623	0.127861
O43422	THAP12	52 kDa repressor of the inhibitor of the protei	1	104.9	105.1	81.2	106.6	105	93.9	0.894285714	0.127616
Q14061	COX17	Cytochrome c oxidase copper chaperone OS=	2	110.2	98.8	95.9	91	104.5	93.45	0.894258373	0.127491
A8K6M4		cDNA FLJ75725, highly similar to Homo sap	4	110.5	107.8	100.5	94.7	109.15	97.6	0.894182318	0.127145
B2RBA0		cDNA, FLJ95388, highly similar to Homo sap	3	110.5	92.8	87.9	93.8	101.65	90.85	0.893753074	0.125207
B5BU83	STMN1	Stathmin OS=Homo sapiens GN=STMN1 PE	11	100	103.9	90.1	92.1	101.95	91.1	0.893575282	0.124411
Q13057	COASY	Bifunctional coenzyme A synthase OS=Homo	1	133.2	89.4	103.1	95.8	111.3	99.45	0.893530997	0.124213
A0A024QZW0	TPMT	Thiopurine S-methyltransferase, isoform CRA	1	106.8	97.6	85.8	96.8	102.2	91.3	0.89334638	0.123391
A0A024RDT9	FNDC3A	Fibronectin type III domain containing 3A, is	3	113.9	100.7	95	96.7	107.3	95.85	0.893289842	0.12314
Q5VSQ6	P4HA1	Procollagen-proline, 2-oxoglutarate 4-dioxyge	1	112	103.5	98.2	94.3	107.75	96.25	0.893271462	0.123059
Q14554	PDIA5	Protein disulfide-isomerase A5 OS=Homo sap	3	102.6	106.2	93.7	92.8	104.4	93.25	0.893199234	0.122739
Q8NHM5	KDM2B	Lysine-specific demethylase 2B OS=Homo sa	1	121.8	87.9	94	93.3	104.85	93.65	0.893180734	0.122657
B3KN52		cDNA FLJ13606 fis, clone PLACE1010579, I	3	103.1	104.7	95	90.6	103.9	92.8	0.893166506	0.122594
Q86WQ0	NR2C2AP	Nuclear receptor 2C2-associated protein OS=I	3	105.8	100.5	91.3	92.9	103.15	92.1	0.892874455	0.121309
P04424	ASL	Argininosuccinate lyase OS=Homo sapiens G	1	100.3	104.8	93	90.1	102.55	91.55	0.892735251	0.1207
O95363	FARS2	Phenylalanine--tRNA ligase, mitochondrial O	2	110.3	101.2	97	91.8	105.75	94.4	0.892671395	0.120421
Q9BQ70	TCF25	Transcription factor 25 OS=Homo sapiens GN	7	106.7	102.8	92.5	94.5	104.75	93.5	0.892601432	0.120116
Q8IWY9	CDAN1	Codanin-1 OS=Homo sapiens GN=CDAN1 P	2	122.3	88	89.3	98.4	105.15	93.85	0.892534475	0.119825
A3KN83	SBNO1	Protein strawberry notch homolog 1 OS=Hom	1	105.9	111	93.6	99.9	108.45	96.75	0.892116183	0.118018
A0A096LP25	AAK1	AP2-associated protein kinase 1 (Fragment) C	3	108.5	103.6	94.3	94.9	106.05	94.6	0.89203206	0.117658
A0A024R7D0	DHPS	Deoxyhypusine synthase, isoform CRA_b OS	5	103.1	103.4	94.7	89.5	103.25	92.1	0.892009685	0.117562
Q9Y294	ASF1A	Histone chaperone ASF1A OS=Homo sapiens	2	114.2	99.1	96.2	94	106.65	95.1	0.891701828	0.116249
Q8WUY8	NAT14	N-acetyltransferase 14 OS=Homo sapiens GN	4	107.7	104.6	95.9	93.4	106.15	94.65	0.891662741	0.116083
A0A024R7C0	KEAP1	Kelch-like ECH-associated protein 1, isoform	3	104.1	97.1	91.3	88.1	100.6	89.7	0.891650099	0.11603
O00764	PDXK	Pyridoxal kinase OS=Homo sapiens GN=PD2	4	104.6	104.8	94.1	92.6	104.7	93.35	0.891595033	0.115796
Q9Y2G0	EFR3B	Protein EFR3 homolog B OS=Homo sapiens (	1	110.9	101.5	101	88.3	106.2	94.65	0.891242938	0.114313
Q5T7M9	FAM69A	Protein FAM69A OS=Homo sapiens GN=FA	1	115.7	95.7	92.2	96.2	105.7	94.2	0.891201514	0.114139
P16401	HIST1H1B	Histone H1.5 OS=Homo sapiens GN=HIST1I	8	102.5	104.3	92	92.3	103.4	92.15	0.891199226	0.11413
A0A0S2Z3S5	GNAS	GNAS complex locus isoform 2 (Fragment) C	1	122.3	95.5	98.7	95.4	108.9	97.05	0.891184573	0.114069
Q8IXI1	RHOT2	Mitochondrial Rho GTPase 2 OS=Homo sapi	7	119.9	93.8	95	95.4	106.85	95.2	0.890968648	0.113167
P49247	RPIA	Ribose-5-phosphate isomerase OS=Homo sap	6	107.5	105.2	95.1	94.4	106.35	94.75	0.890926187	0.112991
B0FTY2		NudC-like protein OS=Homo sapiens PE=1 S	1	124.7	88.2	94.2	95.4	106.45	94.8	0.890558948	0.111473
Q9Y3P4	RHBDD3	Rhomboid domain-containing protein 3 OS=H	1	109.4	100.6	98.2	88.8	105	93.5	0.89047619	0.111133
Q96GG9	DCUN1D1	DCN1-like protein 1 OS=Homo sapiens GN=	2	110.8	97.2	94.3	90.9	104	92.6	0.890384615	0.110757
A0A024R3Z5	LANCL1	LanC lantibiotic synthetase component C-like	4	112.6	100.5	91	98.7	106.55	94.85	0.890192398	0.109973
G3V2U7	ACYP1	Acylphosphatase OS=Homo sapiens GN=AC	2	101.7	103.2	95.2	87.2	102.45	91.2	0.890190337	0.109964
Q8NB90	SPATA5	Spermatogenesis-associated protein 5 OS=Ho	1	105	103.3	89.4	96	104.15	92.7	0.89006241	0.109444
Q8WYQ5	DGCR8	Microprocessor complex subunit DGCR8 OS	1	91.9	98.1	92.8	76.3	95	84.55	0.89	0.109191
P49746	THBS3	Thrombospondin-3 OS=Homo sapiens GN=T	2	98.2	93.6	86.1	84.6	95.9	85.35	0.889989572	0.109149
Q9GZU8	FAM192A	Protein FAM192A OS=Homo sapiens GN=F.	4	110	99	92.2	93.8	104.5	93	0.889952153	0.108998
A0A024R1Y7	LGP1	Homolog of mouse LGP1, isoform CRA_a O	1	113.9	98	92.8	95.7	105.95	94.25	0.889570552	0.107463
A8K654		cDNA FLJ75178, highly similar to Homo sap	2	108.5	96.8	96.4	86.2	102.65	91.3	0.889430102	0.106903
P46977	STT3A	Dolichyl-diphosphooligosaccharide--protein g	9	115.1	97.4	96	93	106.25	94.5	0.889411765	0.106829
Q5U0J5	CREB1	cAMP responsive element binding protein 1 (	1	114.2	92.7	95.6	88.4	103.45	92	0.889318511	0.106459
O43896	KIF1C	Kinesin-like protein KIF1C OS=Homo sapien	2	107.1	104.7	91.2	97.1	105.9	94.15	0.88904627	0.105381
A0A024R4Z9	SBF1	SET binding factor 1, isoform CRA_a OS=H	6	116	103	94.1	100.6	109.5	97.35	0.889041096	0.105361
F4MHI3	UTY	Ubiquitously transcribed tetratricopeptide rep	1	105.9	101.2	93.3	90.8	103.55	92.05	0.88894254	0.104973
Q96F88	POP1	Processing of 1, ribonuclease P/MRP subunit	16	110.5	105.4	97.2	94.7	107.95	95.95	0.888837425	0.104561
A8K291		cDNA FLJ77924, highly similar to Homo sap	1	101.7	102.9	91.9	89.9	102.3	90.9	0.88856305	0.10349
A8K4M4		cDNA FLJ75329, highly similar to Homo sap	5	119.3	95.9	97.4	93.8	107.6	95.6	0.888475836	0.103152
O00762	UBE2C	Ubiquitin-conjugating enzyme E2 C OS=Hom	1	111.9	100.4	89.3	99.3	106.15	94.3	0.88836552	0.102725
Q14181	POLA2	DNA polymerase alpha subunit B OS=Homo	1	107.5	103	98.2	88.8	105.25	93.5	0.888361045	0.102707
Q92614	MYO18A	Unconventional myosin-XVIIIa OS=Homo sa	5	112.1	104.6	97	95.5	108.35	96.25	0.888324873	0.102568
B2R623		mRNA-capping enzyme OS=Homo sapiens P	2	120.8	102.4	100.8	97.4	111.6	99.1	0.887992832	0.101292
Q9H8J5	MANSC1	MANSC domain-containing protein 1 OS=Ho	1	118.4	94.9	94	95.4	106.65	94.7	0.887951242	0.101133
D3DPG9	CALCRL	Calcitonin receptor-like, isoform CRA_a OS=	1	107.4	105.9	97.3	92.1	106.65	94.7	0.887951242	0.101133
A8K4S9		Signal transducer and activator of transcriptio	2	113.8	98.6	93.8	94.8	106.2	94.3	0.887947269	0.101118
Q86TI2	DPP9	Dipeptidyl peptidase 9 OS=Homo sapiens GN	2	100.7	108.9	92.9	93.2	104.8	93.05	0.887881679	0.100868
P46934	NEDD4	E3 ubiquitin-protein ligase NEDD4 OS=Hom	1	113.6	101.2	94.9	95.8	107.4	95.35	0.887802607	0.100567
Q15369	TCEB1	Transcription elongation factor B polypeptide	5	105.3	106.8	96.9	91.4	106.05	94.15	0.887788779	0.100514
P52888	THOP1	Thimet oligopeptidase OS=Homo sapiens GN	13	106.1	102.1	92.3	92.5	104.1	92.4	0.887608069	0.0998291
B3KN15		cDNA FLJ13198 fis, clone NT2RP3004454, I	7	104.2	107.5	93.9	94	105.85	93.95	0.88757676	0.0997109
A0A0S2Z5C9	FTSJ1	Putative tRNA (cytidine(32)/guanosine(34)-2'	1	111	106	92.8	99.8	108.5	96.3	0.887557604	0.0996386
B3KRQ2		cDNA FLJ34689 fis, clone MESAN2000815,	5	114.9	103.8	97.9	96.2	109.35	97.05	0.887517147	0.0994859

B4E2U0		6-phosphogluconate dehydrogenase, decarbox	8	105.9	102.1	94.7	89.9	104	92.3	0.8875	0.0994212
A0A024R8U5	SFRS2	Splicing factor, arginine/serine-rich 2, isoform	7	113.1	101.1	94.5	95.6	107.1	95.05	0.887488329	0.0993772
Q8WZ19	KCTD13	BTB/POZ domain-containing adapter for CUI	1	102.8	110.5	96.5	92.8	106.65	94.65	0.887482419	0.0993549
A0A024RBE7	TMPO	Thymopoietin, isoform CRA_c OS=Homo sap	11	105	104.2	91.8	93.8	104.6	92.8	0.887189293	0.0982554
Q9UBU7	DBF4	Protein DBF4 homolog A OS=Homo sapiens	1	105.4	99	88.4	92.9	102.2	90.65	0.886986301	0.0974995
A0A024R319	LAMB2	Laminin, beta 2 (Laminin S), isoform CRA_a	19	106.7	102.1	90.5	94.7	104.4	92.6	0.88697318	0.0974509
B2R9W9		Craniofacial development protein 1 OS=Hom	1	112.2	97.4	93.2	92.7	104.8	92.95	0.886927481	0.0972813
Q09472	EP300	Histone acetyltransferase p300 OS=Homo sap	1	109.9	110.7	94.7	100.9	110.3	97.8	0.886672711	0.0963406
A0A140T8X7	PPT2	Lysosomal thioesterase PPT2 OS=Homo sapi	1	102.1	113	97.4	93.3	107.55	95.35	0.886564389	0.0959426
O43423	ANP32C	Acidic leucine-rich nuclear phosphoprotein 32	1	102.7	103.5	84.6	98.2	103.1	91.4	0.886517944	0.0957724
Q9HCN3	TMEM8A	Transmembrane protein 8A OS=Homo sapien	1	136.1	97.1	87.2	119.4	116.6	103.3	0.88593482	0.0936553
Q9GZN8	C20orf27	UPF0687 protein C20orf27 OS=Homo sapien	3	106.9	106.8	94.7	94.6	106.85	94.65	0.885821245	0.093247
Q9UBY8	CLN8	Protein CLN8 OS=Homo sapiens GN=CLN8	2	113.8	102.5	93	98.6	108.15	95.8	0.88580675	0.0931951
Q8IUR7	ARMC8	Armadillo repeat-containing protein 8 OS=Ho	3	111.7	106.3	93.9	99.2	109	96.55	0.885779817	0.0930985
Q9Y4E6	WDR7	WD repeat-containing protein 7 OS=Homo sa	2	117.8	102.4	96.2	98.8	110.1	97.5	0.885558583	0.0923086
A0A0S2Z462	AGFG1	ArfGAP with FG repeats 1 isoform 2 (Fragme	3	115.5	102.9	97.7	95.7	109.2	96.7	0.885531136	0.0922108
Q6UJX3		CGI-112 protein OS=Homo sapiens PE=2 SV	1	106.2	112.2	97.2	96.2	109.2	96.7	0.885531136	0.0922108
Q9H9A7	RMI1	RecQ-mediated genome instability protein 1 (	1	115.3	106.3	97.1	99.1	110.8	98.1	0.885379061	0.0916713
D9ZGF8	ROCK1	Rho-associated protein kinase OS=Homo sapi	9	105.1	111.2	98.4	93.1	108.15	95.75	0.885344429	0.0915487
B3KVB3		cDNA FLJ16332 fis, clone SYNOV2016837,	1	108	113	104.5	91.1	110.5	97.8	0.885067873	0.0905746
A0A087WSV(	BCL2L12	Bcl-2-like protein 12 OS=Homo sapiens GN=	4	111	100.1	90.2	96.6	105.55	93.4	0.884888678	0.0899476
Q9NZJ5	EIF2AK3	Eukaryotic translation initiation factor 2-alpha	4	107.5	101.7	96.4	88.7	104.6	92.55	0.884799235	0.0896362
Q9HBM1	SPC25	Kinetochore protein Spc25 OS=Homo sapiens	2	111.6	101.8	92	96.8	106.7	94.4	0.884723524	0.0893731
A0A024R8U1	SLC16A3	Solute carrier family 16 (Monocarboxylic acid	2	108.2	103.4	94.9	92.3	105.8	93.6	0.884688091	0.08925
A0A0A1TTQ(	LU	Lutheran blood group glycoprotein (Fragment	6	105.7	99.8	88.6	93.2	102.75	90.9	0.884671533	0.0891926
A0A1B0GTF3	HSPA12A	Heat shock 70 kDa protein 12A OS=Homo sa	1	125.8	97.4	93.7	103.7	111.6	98.7	0.884408602	0.088285
O15085	ARHGEF11	Rho guanine nucleotide exchange factor 11 O	4	112.6	106.2	95.8	97.7	109.4	96.75	0.884369287	0.08815
E7EQB3	TSEN34	tRNA-splicing endonuclease subunit Sen34 O	3	107.1	102.9	95.4	90.3	105	92.85	0.884285714	0.0878632
Q86UA1	PRPF39	Pre-mRNA-processing factor 39 OS=Homo sa	1	97.2	115.3	92.4	95.5	106.25	93.95	0.884235294	0.0876907
Q9NRY4	ARHGAP35	Rho GTPase-activating protein 35 OS=Homo	3	106.5	101.5	94.5	89.3	104	91.9	0.883653846	0.0857192
O75884	RBBP9	Putative hydrolase RBBP9 OS=Homo sapiens	1	107.2	100.7	84	99.7	103.95	91.85	0.883597884	0.0855315
H7BYY1	TPM1	Tropomyosin 1 (Alpha), isoform CRA_m OS=	1	111.1	107.9	101.9	91.6	109.5	96.75	0.883561644	0.08541
Q9HAV0	GNB4	Guanine nucleotide-binding protein subunit be	3	106.1	107.2	93.4	95	106.65	94.2	0.88326301	0.0844136
B2R7N9		cDNA, FLJ93532, highly similar to Homo sap	1	122.7	113.5	110.7	97.9	118.1	104.3	0.883149873	0.0840387
Q9UBW8	COPS7A	COP9 signalosome complex subunit 7a OS=H	4	104.2	104.1	91.1	92.8	104.15	91.95	0.882861258	0.0830878
A0A140VK84	FN3KRP	Fructosamine-3-kinase-related protein, isoforr	1	110.6	106.9	87.4	104.6	108.75	96	0.882758621	0.0827518
Q9NSY1	BMP2K	BMP-2-inducible protein kinase OS=Homo sa	6	109.3	98.7	93.3	90.3	104	91.8	0.882692308	0.0825353
O43347	MSI1	RNA-binding protein Musashi homolog 1 OS	1	121.9	95.3	97.5	94.2	108.6	95.85	0.882596685	0.0822236
A0A024RBL2	SDSL	Serine dehydratase-like, isoform CRA_a OS=	1	106.7	110.2	93.4	98	108.45	95.7	0.882434302	0.0816968
A0A024R509	ARSA	Arylsulfatase A, isoform CRA_a OS=Homo s	2	117.3	103.8	97	98.1	110.55	97.55	0.882406151	0.0816058
P49069	CAMLG	Calcium signal-modulating cyclophilin ligand	2	108.5	109.1	91.1	100.9	108.8	96	0.882352941	0.0814338
Q9Y5W7	SNX14	Sorting nexin-14 OS=Homo sapiens GN=SN>	5	109.8	102.5	95.7	91.6	106.15	93.65	0.88224211	0.0810765
P42677	RPS27	40S ribosomal protein S27 OS=Homo sapiens	2	112.9	105.2	99	93.4	109.05	96.2	0.882164145	0.0808259
O94763	URI1	Unconventional prefoldin RPB5 interactor 1 (	2	114.3	97	92	94.4	105.65	93.2	0.882158069	0.0808064
H0YHG0		Uncharacterized protein (Fragment) OS=Hom	1	102	115.2	94.4	97.2	108.6	95.8	0.88213628	0.0807366
M0QXR3	MDN1	Midasin (Fragment) OS=Homo sapiens GN=M	1	92.3	130.8	103.1	93.7	111.55	98.4	0.882115643	0.0806705
Q01664	TFAP4	Transcription factor AP-4 OS=Homo sapiens	1	124.5	86.5	97.3	88.8	105.5	93.05	0.881990521	0.0802701
A0A1B0GUA'	KIF1BP	KIF1-binding protein OS=Homo sapiens GN=	5	104.2	105.4	93.1	91.7	104.8	92.4	0.881679389	0.0792819
D3DTP7	hCG_1987383	HCG1987383, isoform CRA_a OS=Homo sap	1	107.9	107.8	88.8	101.3	107.85	95.05	0.881316643	0.0781416
B4DZK0		cDNA FLJ56391, highly similar to Cysteine p	3	109.1	105.4	92.4	96.6	107.25	94.5	0.881118881	0.0775253
M0QXA7	WIZ	Protein Wiz OS=Homo sapiens GN=WIZ PE=	2	94.3	92.4	86	78.5	93.35	82.25	0.881092662	0.0774439
C9K0J5	RAPH1	Ras association (RalGDS/AF-6) and pleckstri	1	115.1	104.2	87.7	105.5	109.65	96.6	0.880984952	0.0771101
E9PK91	BCLAF1	Bcl-2-associated transcription factor 1 OS=Ho	14	120.1	98.3	97.7	94.7	109.2	96.2	0.880952381	0.0770093
B4DEH1		cDNA FLJ60436, highly similar to Homo sap	2	104	101.8	94	87.3	102.9	90.65	0.880952381	0.0770093
V9HWC9	HEL-S-44	Superoxide dismutase [Cu-Zn] OS=Homo sap	6	108.4	105.6	94.6	93.9	107	94.25	0.880841121	0.0766663
D3DRP5	C9orf19	Chromosome 9 open reading frame 19, isofor	4	128.7	94.3	99.9	96.5	111.5	98.2	0.880717489	0.0762864
Q12873	CHD3	Chromodomain-helicase-DNA-binding protei	5	105.3	102.4	91.5	91.4	103.85	91.45	0.880597015	0.0759174
Q9UHD9	UBQLN2	Ubiquilin-2 OS=Homo sapiens GN=UBQLN2	4	111.1	101.5	90.2	97	106.3	93.6	0.880526811	0.0757033
B7ZLW0	LPP	LPP protein OS=Homo sapiens GN=LPP PE=	6	107	102.9	91.2	93.6	104.95	92.4	0.880419247	0.0753759
Q9BVL2	NUP58	Nucleoporin p58/p45 OS=Homo sapiens GN=	7	121.5	96.7	96.5	95.6	109.1	96.05	0.880384968	0.0752718
O14569	CYB561D2	Cytochrome b561 domain-containing protein	1	108.5	103.3	88.5	97.9	105.9	93.2	0.880075543	0.0743372
A0A024R8B2	FREQ	Frequenin homolog (Drosophila), isoform CR	2	113.6	102.8	99.5	90.9	108.2	95.2	0.879852126	0.0736682
Q96FX7	TRMT61A	tRNA (adenine(58)-N(1))-methyltransferase c	3	110.2	101.6	85.7	100.6	105.9	93.15	0.879603399	0.072929
P08397	HMBS	Porphobilinogen deaminase OS=Homo sapier	5	101.9	107.4	92.8	91.3	104.65	92.05	0.879598662	0.072915
A0A024QYX3	RBM3	RNA binding motif (RNP1, RRM) protein 3,	2	100.3	115.6	92.6	97.3	107.95	94.95	0.879573877	0.0728417
Q9P206	KIAA1522	Uncharacterized protein KIAA1522 OS=Hom	1	111.7	107.4	94.6	98.1	109.55	96.35	0.879507074	0.0726443
Q9H3S5	PIGM	GPI mannosyltransferase 1 OS=Homo sapiens	1	123	98.5	103.4	91.4	110.75	97.4	0.879458239	0.0725003
Q53H76	PLA1A	Phospholipase A1 member A OS=Homo sapie	1	123.6	78.2	93.8	83.6	100.9	88.7	0.879088206	0.0714166
Q9ULP9	TBC1D24	TBC1 domain family member 24 OS=Homo s	2	123.2	95.1	95.2	96.7	109.15	95.95	0.879065506	0.0713505
Q86UT6	NLRX1	NLR family member X1 OS=Homo sapiens C	1	125.4	102.8	87.8	112.8	114.1	100.3	0.879053462	0.0713154
O95551	TDP2	Tyrosyl-DNA phosphodiesterase 2 OS=Homo	2	105.8	109.7	96.5	92.9	107.75	94.7	0.878886311	0.0708305
A8MST6	CEP78	Centrosomal protein of 78 kDa OS=Homo sap	1	112.6	96.7	92.8	91.1	104.65	91.95	0.878643096	0.0701298
Q7Z736	PLEKHH3	Pleckstrin homology domain-containing famil	1	112.1	105.4	96.9	94.2	108.75	95.55	0.87862069	0.0700655
O14521	SDHD	Succinate dehydrogenase [ubiquinone] cytoch	2	118.5	101.6	100.4	92.9	110.05	96.65	0.878237165	0.0689726
Q00796	SORD	Sorbitol dehydrogenase OS=Homo sapiens GI	7	106.1	100	90.2	90.7	103.05	90.45	0.877729258	0.0675458
P04406	GAPDH	Glyceraldehyde-3-phosphate dehydrogenase (	10	98	103.9	88	89.2	100.95	88.6	0.877662209	0.0673592
O75147	OBSL1	Obscurin-like protein 1 OS=Homo sapiens GI	3	115.4	100.2	97.6	91.6	107.8	94.6	0.87755102	0.0670508
A0A024RBG7	NFYB	Nuclear transcription factor Y, beta, isoform (	1	110.7	105.2	94.1	95.3	107.95	94.7	0.87725799	0.0662432
H0Y3T6	SDF4	45 kDa calcium-binding protein (Fragment) C	1	105.1	102.7	86.3	95.9	103.9	91.1	0.87680462	0.0650089
D3DVC4	NES	Nestin, isoform CRA_c OS=Homo sapiens GI	65	106.1	104.6	92.5	92.2	105.35	92.35	0.876601804	0.0644628
Q6UWZ7	FAM175A	BRCA1-A complex subunit Abraxas OS=Hor	1	106.2	99.3	89.6	90.5	102.75	90.05	0.876399027	0.0639204
Q8N0V3	RBFA	Putative ribosome-binding factor A, mitochon	2	98.3	101.5	85.5	89.6	99.9	87.55	0.876376376	0.0638601
Q14526	HIC1	Hypermethylated in cancer 1 protein OS=Hon	1	123.9	83.9	91.4	90.7	103.9	91.05	0.876323388	0.0637191
P36639	NUDT1	7,8-dihydro-8-oxoguanine triphosphatase OS=	1	99.7	108.1	92.5	89.6	103.9	91.05	0.876323388	0.0637191
Q9UJK0	TSR3	Ribosome biogenesis protein TSR3 homolog (	1	100	108.3	87.1	95.4	104.15	91.25	0.876140182	0.0632333
A0A024R2H0	TBC1D5	TBC1 domain family, member 5, isoform CR	1	108.6	103.6	86.7	99.2	106.1	92.95	0.87606032	0.0630225
Q92508	PIEZO1	Piezo-type mechanosensitive ion channel com	2	119.8	97.9	95.1	95.6	108.85	95.35	0.875976114	0.0628008
A0A0J9YXC7	LIMS4	LIM and senescent cell antigen-like-containin	3	109.9	105.4	93.4	95.1	107.65	94.25	0.875522527	0.0616178
Q8IXL7	MSRB3	Methionine-R-sulfoxide reductase B3 OS=Ho	1	110.5	110.2	94.9	98.3	110.35	96.6	0.875396466	0.0612921
Q96PY5	FMNL2	Formin-like protein 2 OS=Homo sapiens GN=	2	122.8	104.9	103.9	95.4	113.85	99.65	0.875274484	0.0609783
P42025	ACTR1B	Beta-centractin OS=Homo sapiens GN=ACTI	3	112.2	98.6	93.8	90.7	105.4	92.25	0.875237192	0.0608825
Q9BXK1	KLF16	Krueppel-like factor 16 OS=Homo sapiens GI	1	116.6	98.2	91.3	96.7	107.4	94	0.875232775	0.0608712
Q9UBG0	MRC2	C-type mannose receptor 2 OS=Homo sapiens	2	108.7	111.3	94.9	97.6	110	96.25	0.875	0.0602769
A0A140VJV0		Testicular tissue protein Li 177 OS=Homo sap	1	114.7	101.6	98.4	90.8	108.15	94.6	0.874711049	0.0595455
A0A024R7N7	IFI30	Interferon, gamma-inducible protein 30, isofo	1	97.2	115.1	92.9	92.8	106.15	92.85	0.874705605	0.0595318
A0A024RBB7	NAP1L1	Nucleosome assembly protein 1-like 1, isofor	1	117.4	101	90.1	100.8	109.2	95.45	0.874084249	0.0579835
Q16658	FSCN1	Fascin OS=Homo sapiens GN=FSCN1 PE=1	5	112	101.8	92	94.8	106.9	93.4	0.873713751	0.0570758
Q8N4T8	CBR4	Carbonyl reductase family member 4 OS=Hoi	1	111.5	101.4	95.1	90.9	106.45	93	0.873649601	0.0569198
P52797	EFNA3	Ephrin-A3 OS=Homo sapiens GN=EFNA3 P	1	117.5	100.1	96.4	93.7	108.8	95.05	0.873621324	0.0568512
O95273	CCNDBP1	Cyclin-D1-binding protein 1 OS=Homo sapie	2	104.4	109.6	92.1	94.8	107	93.45	0.873364486	0.0562307
Q9Y4G6	TLN2	Talin-2 OS=Homo sapiens GN=TLN2 PE=1 S	2	117.5	111.4	104.6	95.3	114.45	99.95	0.873307121	0.0560928
Q9H9Y2	RPF1	Ribosome production factor 1 OS=Homo sapi	8	114.7	98.3	92.3	93.7	106.5	93	0.873239437	0.0559306
B4DSS4		cDNA FLJ56631 OS=Homo sapiens PE=2 SV	1	111.3	88.8	83.8	90.9	100.05	87.35	0.873063468	0.0555103
Q8IW35	CEP97	Centrosomal protein of 97 kDa OS=Homo sap	3	109.8	102.8	88	97.6	106.3	92.8	0.873000941	0.0553616
Q96A54	ADIPOR1	Adiponectin receptor protein 1 OS=Homo sap	1	109.3	104.6	84.4	102.3	106.95	93.35	0.872837775	0.0549752
Q5U000		Cathepsin Z OS=Homo sapiens PE=2 SV=1	5	107.1	99.3	93	87.1	103.2	90.05	0.872577519	0.0543633
M0QXC9	FBL	rRNA 2'-O-methyltransferase fibrillarin OS=I	1	104.5	111.5	97.4	91	108	94.2	0.872222222	0.0535368
Q6ZS86	GK5	Putative glycerol kinase 5 OS=Homo sapiens	1	112.3	98.9	78	106.2	105.6	92.1	0.872159091	0.0533909
A0A1B0GUI1	RALGAPA1	Ral GTPase-activating protein subunit alpha-1	3	118.2	102.8	101.8	90.9	110.5	96.35	0.871945701	0.0529005
Q92859	NEO1	Neogenin OS=Homo sapiens GN=NEO1 PE=	1	120.8	95.4	94	94.5	108.1	94.25	0.871877891	0.0527455
Q9NPH2	ISYNA1	Inositol-3-phosphate synthase 1 OS=Homo sa	9	100.8	105.9	89	91.2	103.35	90.1	0.871794872	0.0525561
P15559	NQO1	NAD(P)H dehydrogenase [quinone] 1 OS=Ho	12	100.9	105.7	91.4	88.7	103.3	90.05	0.871732817	0.052415
A0A024R3L1	SLC37A4	Solute carrier family 37 (Glycerol-6-phosphat	1	108.7	103.9	96.2	89.1	106.3	92.65	0.87158984	0.0520908
Q9UHY7	ENOPH1	Enolase-phosphatase E1 OS=Homo sapiens G	5	113.3	104.5	97.3	92.5	108.9	94.9	0.87144169	0.0517566
Q99571	P2RX4	P2X purinoceptor 4 OS=Homo sapiens GN=F	1	136	87.2	102.4	92.1	111.6	97.25	0.871415771	0.0516985
Q96EP0	RNF31	E3 ubiquitin-protein ligase RNF31 OS=Homo	1	117.8	94.5	83.8	101.1	106.15	92.45	0.870937353	0.0506322
Q9NRK6	ABCB10	ATP-binding cassette sub-family B member 1	8	116.7	98.6	90.8	96.7	107.65	93.75	0.870877845	0.0505009
O95394	PGM3	Phosphoacetylglucosamine mutase OS=Homo	3	104.4	94.6	84.4	88.9	99.5	86.65	0.870854271	0.0504489
P52943	CRIP2	Cysteine-rich protein 2 OS=Homo sapiens GN	5	105.6	105.7	93.6	90.4	105.65	92	0.870799811	0.0503291

Table S4. Correlation between mir-150 and 1851 protein-coding genes in 265 OC tissues from TCGA.

MicroRNA	Gene Symbol	Gene ID	r (Pearson)	*P* value
hsa-mir-150	UNC119B	84747	-0.413702	2.22E-12
hsa-mir-150	USP21	27005	-0.383968	9.75E-11
hsa-mir-150	ALLC	55821	-0.380503	1.48E-10
hsa-mir-150	KIAA1549	57670	-0.374316	3.08E-10
hsa-mir-150	FAM117B	150864	-0.365063	8.93E-10
hsa-mir-150	FGFR3	2261	-0.363122	1.11E-09
hsa-mir-150	RCOR2	283248	-0.361058	1.40E-09
hsa-mir-150	ASB5	140458	-0.359626	1.64E-09
hsa-mir-150	ALPI	248	-0.359159	1.73E-09
hsa-mir-150	CACNA2D2	9254	-0.358098	1.95E-09
hsa-mir-150	LANCL2	55915	-0.357550	2.07E-09
hsa-mir-150	ZCCHC3	85364	-0.350700	4.37E-09
hsa-mir-150	CBX2	84733	-0.349719	4.86E-09
hsa-mir-150	KCNG1	3755	-0.348560	5.50E-09
hsa-mir-150	PREPL	9581	-0.347507	6.16E-09
hsa-mir-150	ACVR2B	93	-0.344031	8.90E-09
hsa-mir-150	IRS1	3667	-0.343878	9.05E-09
hsa-mir-150	C14orf135	64430	-0.341861	1.12E-08
hsa-mir-150	COLEC11	78989	-0.341310	1.18E-08
hsa-mir-150	ZNF512	84450	-0.340366	1.31E-08
hsa-mir-150	DUSP19	142679	-0.340192	1.33E-08
hsa-mir-150	C14orf104	55172	-0.337131	1.83E-08
hsa-mir-150	SMO	6608	-0.335082	2.25E-08
hsa-mir-150	IFT81	28981	-0.334713	2.34E-08
hsa-mir-150	TET1	80312	-0.334621	2.36E-08
hsa-mir-150	EFR3B	22979	-0.332602	2.90E-08
hsa-mir-150	PEX19	5824	-0.329650	3.90E-08
hsa-mir-150	CNPY1	285888	-0.328419	4.41E-08
hsa-mir-150	SLC5A6	8884	-0.328110	4.55E-08
hsa-mir-150	PFKM	5213	-0.327593	4.79E-08
hsa-mir-150	DHX57	90957	-0.325407	5.95E-08
hsa-mir-150	KIAA0895	23366	-0.325377	5.96E-08
hsa-mir-150	CYTL1	54360	-0.324525	6.48E-08
hsa-mir-150	ING5	84289	-0.322433	7.96E-08
hsa-mir-150	PLEKHH1	57475	-0.321793	8.47E-08
hsa-mir-150	EFNA3	1944	-0.321132	9.03E-08
hsa-mir-150	ZBED3	84327	-0.320794	9.33E-08
hsa-mir-150	KCP	375616	-0.320033	1.00E-07
hsa-mir-150	FGFRL1	53834	-0.319754	1.03E-07
hsa-mir-150	ZSCAN18	65982	-0.319288	1.08E-07
hsa-mir-150	ZNF10	7556	-0.319255	1.08E-07
hsa-mir-150	WDR35	57539	-0.318077	1.21E-07
hsa-mir-150	CFC1B	653275	-0.317623	1.27E-07
hsa-mir-150	PTCH1	5727	-0.315848	1.50E-07
hsa-mir-150	SDK2	54549	-0.314999	1.63E-07
hsa-mir-150	GPC1	2817	-0.314045	1.78E-07
hsa-mir-150	MAP4K3	8491	-0.313736	1.83E-07
hsa-mir-150	GTF3C2	2976	-0.313481	1.88E-07
hsa-mir-150	PIGM	93183	-0.312680	2.03E-07
hsa-mir-150	LRP4	4038	-0.312666	2.03E-07
hsa-mir-150	SBK1	388228	-0.310126	2.57E-07
hsa-mir-150	WDR12	55759	-0.309642	2.69E-07

hsa-mir-150	SEC14L5	9717	-0.308504	2.99E-07
hsa-mir-150	COL4A5	1287	-0.308270	3.06E-07
hsa-mir-150	KDM5B	10765	-0.307339	3.33E-07
hsa-mir-150	LRP6	4040	-0.306643	3.55E-07
hsa-mir-150	RSPH4A	345895	-0.306636	3.56E-07
hsa-mir-150	RMND5A	64795	-0.306542	3.59E-07
hsa-mir-150	QSOX2	169714	-0.305841	3.83E-07
hsa-mir-150	SAMD13	148418	-0.305382	3.99E-07
hsa-mir-150	BTBD3	22903	-0.304678	4.26E-07
hsa-mir-150	B3GNT1	11041	-0.304527	4.32E-07
hsa-mir-150	PANK1	53354	-0.304253	4.43E-07
hsa-mir-150	ADNP	23394	-0.304239	4.43E-07
hsa-mir-150	TTLL4	9654	-0.304107	4.48E-07
hsa-mir-150	SMARCD1	6602	-0.303163	4.89E-07
hsa-mir-150	FBXO11	80204	-0.302033	5.41E-07
hsa-mir-150	RBM8A	9939	-0.301726	5.57E-07
hsa-mir-150	ATXN7L3B	552889	-0.301392	5.74E-07
hsa-mir-150	HES6	55502	-0.301020	5.93E-07
hsa-mir-150	SALL2	6297	-0.300968	5.96E-07
hsa-mir-150	STK36	27148	-0.300577	6.17E-07
hsa-mir-150	DGKD	8527	-0.300493	6.22E-07
hsa-mir-150	NUP133	55746	-0.300174	6.40E-07
hsa-mir-150	ZNF84	7637	-0.299711	6.67E-07
hsa-mir-150	USP30	84749	-0.299538	6.78E-07
hsa-mir-150	WNK2	65268	-0.299523	6.79E-07
hsa-mir-150	LOC728723	728723	-0.299320	6.91E-07
hsa-mir-150	DNAI2	64446	-0.298078	7.72E-07
hsa-mir-150	DACH1	1602	-0.297566	8.08E-07
hsa-mir-150	ZNF605	100289635	-0.297265	8.30E-07
hsa-mir-150	ZNF212	7988	-0.297140	8.40E-07
hsa-mir-150	IGF1R	3480	-0.296771	8.68E-07
hsa-mir-150	LRRTM3	347731	-0.296741	8.70E-07
hsa-mir-150	FAM171A2	284069	-0.296027	9.27E-07
hsa-mir-150	ZNF219	51222	-0.295976	9.31E-07
hsa-mir-150	B4GALNT3	283358	-0.295494	9.71E-07
hsa-mir-150	LZTFL1	54585	-0.295065	1.01E-06
hsa-mir-150	LOC642852	642852	-0.294774	1.04E-06
hsa-mir-150	B4GALNT4	338707	-0.294375	1.07E-06
hsa-mir-150	TRAF3IP1	26146	-0.294248	1.08E-06
hsa-mir-150	ZNF253	56242	-0.294088	1.10E-06
hsa-mir-150	STK33	65975	-0.293314	1.18E-06
hsa-mir-150	DHX9	1660	-0.292933	1.22E-06
hsa-mir-150	KBTBD6	89890	-0.292922	1.22E-06
hsa-mir-150	DNAH6	1768	-0.292769	1.23E-06
hsa-mir-150	CHST10	9486	-0.292587	1.25E-06
hsa-mir-150	MAPK8IP1	9479	-0.292518	1.26E-06
hsa-mir-150	CEL	1056	-0.292127	1.31E-06
hsa-mir-150	IGSF9	57549	-0.291277	1.41E-06
hsa-mir-150	CA14	23632	-0.291074	1.43E-06
hsa-mir-150	ZNF423	23090	-0.290791	1.47E-06
hsa-mir-150	ZNF516	9658	-0.290603	1.49E-06
hsa-mir-150	C2orf77	129881	-0.290375	1.52E-06
hsa-mir-150	ZNF3	7551	-0.290174	1.55E-06
hsa-mir-150	PLEKHG4B	153478	-0.289936	1.58E-06

hsa-mir-150	DDX20	11218	-0.289932	1.58E-06
hsa-mir-150	TMEM198	130612	-0.289566	1.63E-06
hsa-mir-150	C10orf71	118461	-0.289335	1.66E-06
hsa-mir-150	EFCAB10	100130771	-0.289317	1.67E-06
hsa-mir-150	PCMTD2	55251	-0.289012	1.71E-06
hsa-mir-150	ZNF167	55888	-0.287840	1.89E-06
hsa-mir-150	PIAS3	10401	-0.287537	1.94E-06
hsa-mir-150	CCDC121	79635	-0.286962	2.04E-06
hsa-mir-150	TSPAN6	7105	-0.286506	2.12E-06
hsa-mir-150	PUM2	23369	-0.286400	2.14E-06
hsa-mir-150	BSN	8927	-0.286374	2.14E-06
hsa-mir-150	ALS2	57679	-0.286163	2.18E-06
hsa-mir-150	COL9A3	1299	-0.285778	2.25E-06
hsa-mir-150	TRMT5	57570	-0.285768	2.26E-06
hsa-mir-150	C20orf96	140680	-0.285707	2.27E-06
hsa-mir-150	SLC19A2	10560	-0.285626	2.28E-06
hsa-mir-150	TTLL2	83887	-0.285507	2.31E-06
hsa-mir-150	VANGL2	57216	-0.285504	2.31E-06
hsa-mir-150	PSPH	5723	-0.285290	2.35E-06
hsa-mir-150	EDN3	1908	-0.285115	2.39E-06
hsa-mir-150	DLK1	8788	-0.284618	2.49E-06
hsa-mir-150	CHD4	1108	-0.284599	2.49E-06
hsa-mir-150	BBS5	129880	-0.284546	2.50E-06
hsa-mir-150	MSH6	2956	-0.284374	2.54E-06
hsa-mir-150	MTERFD2	130916	-0.284287	2.56E-06
hsa-mir-150	SSTR4	6754	-0.284265	2.56E-06
hsa-mir-150	POMT2	29954	-0.283706	2.69E-06
hsa-mir-150	LOC144438	144438	-0.283506	2.73E-06
hsa-mir-150	ANKZF1	55139	-0.283449	2.75E-06
hsa-mir-150	LRRC10B	390205	-0.283371	2.76E-06
hsa-mir-150	FASTKD2	22868	-0.283327	2.77E-06
hsa-mir-150	FGF8	2253	-0.283095	2.83E-06
hsa-mir-150	LCN10	414332	-0.282588	2.95E-06
hsa-mir-150	DCAF8	50717	-0.282464	2.98E-06
hsa-mir-150	LOC100130238	100130238	-0.281887	3.13E-06
hsa-mir-150	ZNF664	144348	-0.281764	3.16E-06
hsa-mir-150	GXYLT1	283464	-0.281670	3.19E-06
hsa-mir-150	TIGD1	200765	-0.281668	3.19E-06
hsa-mir-150	SPAST	6683	-0.281668	3.19E-06
hsa-mir-150	URB2	9816	-0.281635	3.20E-06
hsa-mir-150	DYNC2LI1	51626	-0.280943	3.39E-06
hsa-mir-150	ORC2L	4999	-0.280856	3.41E-06
hsa-mir-150	HNF1A	6927	-0.280669	3.47E-06
hsa-mir-150	PWRN1	791114	-0.280661	3.47E-06
hsa-mir-150	GPR125	166647	-0.280588	3.49E-06
hsa-mir-150	PMS2CL	441194	-0.280514	3.51E-06
hsa-mir-150	ZNF286A	57335	-0.280478	3.52E-06
hsa-mir-150	SEMA6A	57556	-0.280216	3.60E-06
hsa-mir-150	ACTR3B	57180	-0.280206	3.60E-06
hsa-mir-150	LSM14B	149986	-0.279635	3.78E-06
hsa-mir-150	PRRT1	80863	-0.279627	3.78E-06
hsa-mir-150	WNT2B	7482	-0.279384	3.86E-06
hsa-mir-150	FOXN4	121643	-0.278785	4.06E-06
hsa-mir-150	CAD	790	-0.278738	4.07E-06

hsa-mir-150	PNMAL1	55228	-0.278696	4.09E-06
hsa-mir-150	PAQR5	54852	-0.278661	4.10E-06
hsa-mir-150	NKAIN4	128414	-0.278656	4.10E-06
hsa-mir-150	ARMC9	80210	-0.278616	4.11E-06
hsa-mir-150	STAR	6770	-0.278262	4.23E-06
hsa-mir-150	ARNT2	9915	-0.278119	4.29E-06
hsa-mir-150	LAMC3	10319	-0.278018	4.32E-06
hsa-mir-150	EFCAB12	90288	-0.277879	4.37E-06
hsa-mir-150	CCDC40	55036	-0.277822	4.39E-06
hsa-mir-150	KLHL23	151230	-0.277617	4.47E-06
hsa-mir-150	ZNF713	349075	-0.277597	4.47E-06
hsa-mir-150	C7orf41	222166	-0.277551	4.49E-06
hsa-mir-150	ZBTB39	9880	-0.277022	4.69E-06
hsa-mir-150	DNAH7	56171	-0.277000	4.70E-06
hsa-mir-150	VWA5B1	127731	-0.276929	4.73E-06
hsa-mir-150	MARS2	92935	-0.276806	4.77E-06
hsa-mir-150	COL2A1	1280	-0.276703	4.81E-06
hsa-mir-150	NBPF3	84224	-0.276184	5.02E-06
hsa-mir-150	WFDC1	58189	-0.276158	5.03E-06
hsa-mir-150	CRY1	1407	-0.276129	5.05E-06
hsa-mir-150	ITM2C	81618	-0.276056	5.08E-06
hsa-mir-150	ZNF432	9668	-0.275885	5.15E-06
hsa-mir-150	AQP12A	375318	-0.275734	5.21E-06
hsa-mir-150	ZMYM3	9203	-0.275514	5.31E-06
hsa-mir-150	ALS2CR4	65062	-0.275255	5.42E-06
hsa-mir-150	CCDC39	339829	-0.275053	5.51E-06
hsa-mir-150	LYPD6	130574	-0.274987	5.54E-06
hsa-mir-150	CPSF6	11052	-0.274562	5.74E-06
hsa-mir-150	ZNF273	10793	-0.274429	5.80E-06
hsa-mir-150	SF3B1	23451	-0.274307	5.86E-06
hsa-mir-150	PTCH2	8643	-0.274008	6.00E-06
hsa-mir-150	ATP6V1E2	90423	-0.273956	6.03E-06
hsa-mir-150	FGF19	9965	-0.273867	6.07E-06
hsa-mir-150	WASF1	8936	-0.273781	6.11E-06
hsa-mir-150	CUL3	8452	-0.273719	6.14E-06
hsa-mir-150	ACP1	52	-0.273654	6.18E-06
hsa-mir-150	SLC22A5	6584	-0.273396	6.31E-06
hsa-mir-150	SUPT3H	8464	-0.273239	6.39E-06
hsa-mir-150	NDST4	64579	-0.273211	6.40E-06
hsa-mir-150	STOML3	161003	-0.273010	6.51E-06
hsa-mir-150	PRPF40B	25766	-0.272836	6.60E-06
hsa-mir-150	GTF2IP1	2970	-0.272571	6.74E-06
hsa-mir-150	ZNF786	136051	-0.272528	6.77E-06
hsa-mir-150	LOC728819	728819	-0.272214	6.94E-06
hsa-mir-150	TTC21B	79809	-0.272150	6.97E-06
hsa-mir-150	ZNF426	79088	-0.272142	6.98E-06
hsa-mir-150	USP2	9099	-0.271881	7.13E-06
hsa-mir-150	TPPP2	122664	-0.271637	7.27E-06
hsa-mir-150	LANCL1	10314	-0.271536	7.33E-06
hsa-mir-150	SPAG16	79582	-0.271505	7.35E-06
hsa-mir-150	COPS8	10920	-0.271323	7.45E-06
hsa-mir-150	RNF157	114804	-0.271233	7.51E-06
hsa-mir-150	ADAM11	4185	-0.271163	7.55E-06
hsa-mir-150	AGBL5	60509	-0.271088	7.60E-06

hsa-mir-150	IL17RB	55540	-0.271051	7.62E-06
hsa-mir-150	C2orf63	130162	-0.270916	7.70E-06
hsa-mir-150	TUB	7275	-0.270816	7.76E-06
hsa-mir-150	GPLD1	2822	-0.270704	7.84E-06
hsa-mir-150	TCF7L1	83439	-0.270682	7.85E-06
hsa-mir-150	ST7L	54879	-0.270668	7.86E-06
hsa-mir-150	ANO2	57101	-0.270588	7.91E-06
hsa-mir-150	TNFRSF19	55504	-0.270511	7.96E-06
hsa-mir-150	HSPC159	29094	-0.270381	8.04E-06
hsa-mir-150	GNRHR2	114814	-0.270349	8.06E-06
hsa-mir-150	SLC4A8	9498	-0.270348	8.06E-06
hsa-mir-150	SMEK2	57223	-0.270120	8.21E-06
hsa-mir-150	TRIM45	80263	-0.269733	8.47E-06
hsa-mir-150	EIF2B4	8890	-0.269582	8.57E-06
hsa-mir-150	C10orf53	282966	-0.269570	8.58E-06
hsa-mir-150	JPH4	84502	-0.269450	8.66E-06
hsa-mir-150	UGGT2	55757	-0.269427	8.68E-06
hsa-mir-150	IGDCC4	57722	-0.269146	8.87E-06
hsa-mir-150	ZKSCAN2	342357	-0.269137	8.88E-06
hsa-mir-150	ALG10B	144245	-0.268862	9.08E-06
hsa-mir-150	C5orf13	9315	-0.268697	9.20E-06
hsa-mir-150	STK25	10494	-0.268447	9.38E-06
hsa-mir-150	E2F6	1876	-0.268373	9.44E-06
hsa-mir-150	ODC1	4953	-0.268371	9.44E-06
hsa-mir-150	CAPN7	23473	-0.268252	9.53E-06
hsa-mir-150	ULBP3	79465	-0.268069	9.67E-06
hsa-mir-150	RP1-177G6.2	286411	-0.267478	1.01E-05
hsa-mir-150	MEST	4232	-0.267361	1.02E-05
hsa-mir-150	FKBP9	11328	-0.266917	1.06E-05
hsa-mir-150	LGI3	203190	-0.266889	1.06E-05
hsa-mir-150	B3GALNT2	148789	-0.266814	1.07E-05
hsa-mir-150	GPR156	165829	-0.266756	1.07E-05
hsa-mir-150	TSPYL4	23270	-0.266621	1.08E-05
hsa-mir-150	TMED4	222068	-0.266541	1.09E-05
hsa-mir-150	DLEC1	9940	-0.266485	1.10E-05
hsa-mir-150	FAM66C	440078	-0.266451	1.10E-05
hsa-mir-150	HDAC4	9759	-0.265795	1.16E-05
hsa-mir-150	ZNF660	285349	-0.265679	1.17E-05
hsa-mir-150	GLS2	27165	-0.265396	1.19E-05
hsa-mir-150	LOC154449	154449	-0.265364	1.20E-05
hsa-mir-150	LOC388428	388428	-0.264812	1.25E-05
hsa-mir-150	EXTL2	2135	-0.264482	1.28E-05
hsa-mir-150	CAP2	10486	-0.264436	1.29E-05
hsa-mir-150	REPIN1	29803	-0.264393	1.29E-05
hsa-mir-150	C12orf76	400073	-0.264346	1.30E-05
hsa-mir-150	XRCC5	7520	-0.264205	1.31E-05
hsa-mir-150	PRAP1	118471	-0.264161	1.32E-05
hsa-mir-150	RHOBTB1	9886	-0.264051	1.33E-05
hsa-mir-150	KLHL13	90293	-0.263970	1.33E-05
hsa-mir-150	PKP2	5318	-0.263907	1.34E-05
hsa-mir-150	PCSK6	5046	-0.263425	1.39E-05
hsa-mir-150	PAIP2B	400961	-0.263401	1.40E-05
hsa-mir-150	C2orf42	54980	-0.263299	1.41E-05
hsa-mir-150	C2orf44	80304	-0.263201	1.42E-05

hsa-mir-150	HYDIN	54768	-0.263065	1.43E-05
hsa-mir-150	PFAS	5198	-0.262952	1.45E-05
hsa-mir-150	DACT2	168002	-0.262742	1.47E-05
hsa-mir-150	PNMA3	29944	-0.262636	1.48E-05
hsa-mir-150	CLK2P	1197	-0.262616	1.48E-05
hsa-mir-150	LOC654342	654342	-0.262554	1.49E-05
hsa-mir-150	BCL7A	605	-0.262517	1.49E-05
hsa-mir-150	TSC22D2	9819	-0.262301	1.52E-05
hsa-mir-150	TADA1	117143	-0.262151	1.54E-05
hsa-mir-150	HMGA2	8091	-0.262059	1.55E-05
hsa-mir-150	SLC16A1	6566	-0.261945	1.56E-05
hsa-mir-150	PPIL6	285755	-0.261473	1.62E-05
hsa-mir-150	COPS7B	64708	-0.261270	1.65E-05
hsa-mir-150	RFX6	222546	-0.261118	1.67E-05
hsa-mir-150	IPO5	3843	-0.260957	1.69E-05
hsa-mir-150	GLI2	2736	-0.260453	1.75E-05
hsa-mir-150	FBRSL1	57666	-0.260272	1.78E-05
hsa-mir-150	PRMT8	56341	-0.260123	1.80E-05
hsa-mir-150	ZNF763	284390	-0.260058	1.81E-05
hsa-mir-150	GSTM3	2947	-0.259853	1.84E-05
hsa-mir-150	LOC149134	149134	-0.259647	1.87E-05
hsa-mir-150	COL18A1	80781	-0.259622	1.87E-05
hsa-mir-150	FLRT1	23769	-0.259445	1.89E-05
hsa-mir-150	LOC388152	388152	-0.259081	1.95E-05
hsa-mir-150	SKP2	6502	-0.258941	1.97E-05
hsa-mir-150	TMEM67	91147	-0.258812	1.99E-05
hsa-mir-150	LIG3	3980	-0.258795	1.99E-05
hsa-mir-150	NKPD1	284353	-0.258596	2.02E-05
hsa-mir-150	WDR43	23160	-0.258486	2.04E-05
hsa-mir-150	KCNRG	283518	-0.258477	2.04E-05
hsa-mir-150	C2orf86	51057	-0.258348	2.06E-05
hsa-mir-150	KBTBD7	84078	-0.258313	2.07E-05
hsa-mir-150	SLIT1	6585	-0.258174	2.09E-05
hsa-mir-150	KDM1A	23028	-0.258120	2.10E-05
hsa-mir-150	CCNT2	905	-0.257647	2.17E-05
hsa-mir-150	IL17RD	54756	-0.257604	2.18E-05
hsa-mir-150	UNC93A	54346	-0.257496	2.20E-05
hsa-mir-150	PPHLN1	51535	-0.257445	2.21E-05
hsa-mir-150	CRLF1	9244	-0.257406	2.21E-05
hsa-mir-150	FLJ13197	79667	-0.256915	2.30E-05
hsa-mir-150	ATG4B	23192	-0.256867	2.31E-05
hsa-mir-150	ZSCAN2	54993	-0.256750	2.33E-05
hsa-mir-150	GUCA1C	9626	-0.256589	2.36E-05
hsa-mir-150	TYRO3	7301	-0.256393	2.39E-05
hsa-mir-150	RND2	8153	-0.256223	2.42E-05
hsa-mir-150	TET3	200424	-0.256109	2.44E-05
hsa-mir-150	SPRR2F	6705	-0.256007	2.46E-05
hsa-mir-150	FGF23	8074	-0.256004	2.46E-05
hsa-mir-150	ZFYVE9	9372	-0.255865	2.49E-05
hsa-mir-150	PPOX	5498	-0.255834	2.49E-05
hsa-mir-150	C14orf138	79609	-0.255802	2.50E-05
hsa-mir-150	C9orf163	158055	-0.255770	2.51E-05
hsa-mir-150	MSI1	4440	-0.255757	2.51E-05
hsa-mir-150	CAPN10	11132	-0.255721	2.52E-05

hsa-mir-150	FAM161A	84140	-0.255586	2.54E-05
hsa-mir-150	TTC30B	150737	-0.255381	2.58E-05
hsa-mir-150	NKAIN1	79570	-0.255277	2.60E-05
hsa-mir-150	CDHR3	222256	-0.255256	2.61E-05
hsa-mir-150	ZNF692	55657	-0.255248	2.61E-05
hsa-mir-150	C3orf39	84892	-0.255212	2.61E-05
hsa-mir-150	APOC3	345	-0.255155	2.62E-05
hsa-mir-150	CCDC48	79825	-0.255038	2.65E-05
hsa-mir-150	ZNF133	7692	-0.254426	2.77E-05
hsa-mir-150	LOC646762	646762	-0.254393	2.78E-05
hsa-mir-150	PCSK2	5126	-0.254313	2.80E-05
hsa-mir-150	ZNF20	7568	-0.254293	2.80E-05
hsa-mir-150	CCDC151	115948	-0.253973	2.87E-05
hsa-mir-150	ZNF709	163051	-0.253732	2.92E-05
hsa-mir-150	KCNK16	83795	-0.253617	2.95E-05
hsa-mir-150	PLK1S1	55857	-0.253531	2.97E-05
hsa-mir-150	C3orf15	89876	-0.253504	2.97E-05
hsa-mir-150	POGK	57645	-0.253456	2.98E-05
hsa-mir-150	DDN	23109	-0.253165	3.05E-05
hsa-mir-150	GREB1	9687	-0.253088	3.07E-05
hsa-mir-150	CHRND	1144	-0.253059	3.07E-05
hsa-mir-150	LRIG2	9860	-0.252999	3.09E-05
hsa-mir-150	ENKUR	219670	-0.252704	3.15E-05
hsa-mir-150	JUB	84962	-0.252535	3.19E-05
hsa-mir-150	ADCY1	107	-0.252478	3.21E-05
hsa-mir-150	PRDM11	56981	-0.252173	3.28E-05
hsa-mir-150	SHF	90525	-0.252050	3.31E-05
hsa-mir-150	ICA1L	130026	-0.251686	3.40E-05
hsa-mir-150	DNMT3A	1788	-0.251657	3.41E-05
hsa-mir-150	GLP1R	2740	-0.251582	3.43E-05
hsa-mir-150	TIA1	7072	-0.251578	3.43E-05
hsa-mir-150	CHERP	10523	-0.251571	3.43E-05
hsa-mir-150	ZNF354C	30832	-0.251519	3.45E-05
hsa-mir-150	KLHL15	80311	-0.251491	3.45E-05
hsa-mir-150	NRTN	4902	-0.251411	3.47E-05
hsa-mir-150	NCOA5	57727	-0.251375	3.48E-05
hsa-mir-150	LOC644669	644669	-0.251270	3.51E-05
hsa-mir-150	DCX	1641	-0.251238	3.52E-05
hsa-mir-150	CCNB1IP1	57820	-0.251131	3.55E-05
hsa-mir-150	MAGED2	10916	-0.251086	3.56E-05
hsa-mir-150	FKBP4	2288	-0.251051	3.57E-05
hsa-mir-150	SPRED2	200734	-0.250893	3.61E-05
hsa-mir-150	TGDS	23483	-0.250822	3.63E-05
hsa-mir-150	TMEM72	643236	-0.250796	3.63E-05
hsa-mir-150	UBXN10	127733	-0.250624	3.68E-05
hsa-mir-150	SPTB	6710	-0.250591	3.69E-05
hsa-mir-150	LRRC43	254050	-0.250572	3.70E-05
hsa-mir-150	C20orf177	63939	-0.250553	3.70E-05
hsa-mir-150	C9orf45	81571	-0.250297	3.77E-05
hsa-mir-150	GSTA4	2941	-0.250225	3.79E-05
hsa-mir-150	FLJ43390	646113	-0.250218	3.79E-05
hsa-mir-150	RLTPR	146206	0.250205	3.80E-05
hsa-mir-150	AREG	374	0.250220	3.79E-05
hsa-mir-150	GAS1	2619	0.250553	3.70E-05

hsa-mir-150	RAB3IL1	5866	0.250648	3.67E-05
hsa-mir-150	CXCL14	9547	0.250649	3.67E-05
hsa-mir-150	SNX11	29916	0.250691	3.66E-05
hsa-mir-150	RPS6KA1	6195	0.250714	3.66E-05
hsa-mir-150	CTSG	1511	0.250736	3.65E-05
hsa-mir-150	TSHZ3	57616	0.250841	3.62E-05
hsa-mir-150	JAZF1	221895	0.250915	3.60E-05
hsa-mir-150	COL1A1	1277	0.251032	3.57E-05
hsa-mir-150	TMEM26	219623	0.251347	3.49E-05
hsa-mir-150	TRPS1	7227	0.251478	3.46E-05
hsa-mir-150	ALDH2	217	0.251491	3.45E-05
hsa-mir-150	C19orf22	91300	0.251539	3.44E-05
hsa-mir-150	AMPD3	272	0.251583	3.43E-05
hsa-mir-150	SLC16A7	9194	0.251847	3.36E-05
hsa-mir-150	GALNTL2	117248	0.251868	3.36E-05
hsa-mir-150	ACSM5	54988	0.252207	3.27E-05
hsa-mir-150	CHI3L1	1116	0.252218	3.27E-05
hsa-mir-150	ST18	9705	0.252280	3.26E-05
hsa-mir-150	CDKN2A	1029	0.252350	3.24E-05
hsa-mir-150	KRT39	390792	0.252507	3.20E-05
hsa-mir-150	SLC19A3	80704	0.252562	3.19E-05
hsa-mir-150	GFPT2	9945	0.252597	3.18E-05
hsa-mir-150	TIFA	92610	0.252697	3.16E-05
hsa-mir-150	PODNL1	79883	0.253015	3.08E-05
hsa-mir-150	TGFBR2	7048	0.253050	3.07E-05
hsa-mir-150	TMEM51	55092	0.253083	3.07E-05
hsa-mir-150	IFITM1	8519	0.253204	3.04E-05
hsa-mir-150	ME2	4200	0.253219	3.04E-05
hsa-mir-150	ETFDH	2110	0.253427	2.99E-05
hsa-mir-150	C22orf34	348645	0.253542	2.96E-05
hsa-mir-150	LILRA1	11024	0.253607	2.95E-05
hsa-mir-150	OLFML1	283298	0.253778	2.91E-05
hsa-mir-150	HP	3240	0.253804	2.91E-05
hsa-mir-150	PIGR	5284	0.254054	2.85E-05
hsa-mir-150	ADCY7	113	0.254562	2.74E-05
hsa-mir-150	SREBF1	6720	0.254777	2.70E-05
hsa-mir-150	C9orf21	195827	0.254780	2.70E-05
hsa-mir-150	CSTB	1476	0.254814	2.69E-05
hsa-mir-150	COL5A3	50509	0.254831	2.69E-05
hsa-mir-150	ZPLD1	131368	0.255178	2.62E-05
hsa-mir-150	CMPK2	129607	0.255422	2.57E-05
hsa-mir-150	C22orf25	128989	0.255718	2.52E-05
hsa-mir-150	RPLP0P2	113157	0.255747	2.51E-05
hsa-mir-150	PFN1	5216	0.255762	2.51E-05
hsa-mir-150	SCN1B	6324	0.255826	2.50E-05
hsa-mir-150	ABHD3	171586	0.255880	2.49E-05
hsa-mir-150	FAM23A	653567	0.255976	2.47E-05
hsa-mir-150	CP	1356	0.255981	2.47E-05
hsa-mir-150	C9orf25	203259	0.256007	2.46E-05
hsa-mir-150	TLN1	7094	0.256033	2.46E-05
hsa-mir-150	EIF5A2	56648	0.256191	2.43E-05
hsa-mir-150	ERG	2078	0.256207	2.42E-05
hsa-mir-150	SMAD1	4086	0.256382	2.39E-05
hsa-mir-150	C22orf26	55267	0.256489	2.37E-05

hsa-mir-150	LOC285830	285830	0.256665	2.34E-05
hsa-mir-150	MX1	4599	0.256795	2.32E-05
hsa-mir-150	BFSP2	8419	0.256939	2.29E-05
hsa-mir-150	RASSF2	9770	0.257115	2.26E-05
hsa-mir-150	ADAMTS2	9509	0.257122	2.26E-05
hsa-mir-150	AQP9	366	0.257465	2.20E-05
hsa-mir-150	CHMP5	51510	0.257474	2.20E-05
hsa-mir-150	COX7A1	1346	0.257685	2.17E-05
hsa-mir-150	IGFBP6	3489	0.257743	2.16E-05
hsa-mir-150	PLA2G2A	5320	0.257935	2.13E-05
hsa-mir-150	COLEC12	81035	0.258156	2.09E-05
hsa-mir-150	DNASE1L1	1774	0.258273	2.07E-05
hsa-mir-150	VGLL3	389136	0.258291	2.07E-05
hsa-mir-150	PTGDS	5730	0.258409	2.05E-05
hsa-mir-150	MMP19	4327	0.258420	2.05E-05
hsa-mir-150	CKLF	51192	0.258520	2.03E-05
hsa-mir-150	PAMR1	25891	0.258586	2.02E-05
hsa-mir-150	S100A6	6277	0.258796	1.99E-05
hsa-mir-150	PLVAP	83483	0.258963	1.97E-05
hsa-mir-150	C7orf58	79974	0.259044	1.95E-05
hsa-mir-150	SLC25A43	203427	0.259079	1.95E-05
hsa-mir-150	SGIP1	84251	0.259171	1.93E-05
hsa-mir-150	VAMP3	9341	0.259277	1.92E-05
hsa-mir-150	CPNE8	144402	0.259562	1.88E-05
hsa-mir-150	ART4	420	0.259575	1.88E-05
hsa-mir-150	PCGF5	84333	0.259579	1.88E-05
hsa-mir-150	PAPSS2	9060	0.260403	1.76E-05
hsa-mir-150	BTN2A2	10385	0.260512	1.75E-05
hsa-mir-150	TREM1	54210	0.260791	1.71E-05
hsa-mir-150	ITPRIP	85450	0.260876	1.70E-05
hsa-mir-150	PARP3	10039	0.260892	1.70E-05
hsa-mir-150	IRF5	3663	0.260940	1.69E-05
hsa-mir-150	LTBP2	4053	0.261410	1.63E-05
hsa-mir-150	SOCS1	8651	0.261418	1.63E-05
hsa-mir-150	IRF7	3665	0.261422	1.63E-05
hsa-mir-150	TNFRSF1A	7132	0.261460	1.62E-05
hsa-mir-150	TLL1	7092	0.261640	1.60E-05
hsa-mir-150	LOC654433	654433	0.261696	1.59E-05
hsa-mir-150	SLC18A1	6570	0.262006	1.56E-05
hsa-mir-150	VPREB3	29802	0.262126	1.54E-05
hsa-mir-150	FMO3	2328	0.262204	1.53E-05
hsa-mir-150	AP1B1	162	0.262286	1.52E-05
hsa-mir-150	KIAA1618	57714	0.262424	1.51E-05
hsa-mir-150	STARD5	80765	0.262608	1.48E-05
hsa-mir-150	LPPR4	9890	0.262611	1.48E-05
hsa-mir-150	KIAA0247	9766	0.262793	1.46E-05
hsa-mir-150	FAM49A	81553	0.262852	1.46E-05
hsa-mir-150	CCL4L2	388372	0.262889	1.45E-05
hsa-mir-150	OTOR	56914	0.262893	1.45E-05
hsa-mir-150	GJA5	2702	0.263089	1.43E-05
hsa-mir-150	NECAP2	55707	0.263169	1.42E-05
hsa-mir-150	IRAK2	3656	0.263315	1.40E-05
hsa-mir-150	P4HA3	283208	0.263656	1.37E-05
hsa-mir-150	C1orf38	9473	0.263723	1.36E-05

hsa-mir-150	PIK3IP1	113791	0.263805	1.35E-05
hsa-mir-150	TNFSF9	8744	0.263958	1.34E-05
hsa-mir-150	SIGLEC14	100049587	0.264071	1.32E-05
hsa-mir-150	SLC25A45	283130	0.264271	1.30E-05
hsa-mir-150	LOC400696	400696	0.264464	1.28E-05
hsa-mir-150	CFLAR	8837	0.264499	1.28E-05
hsa-mir-150	SWAP70	23075	0.264703	1.26E-05
hsa-mir-150	APOL4	80832	0.264722	1.26E-05
hsa-mir-150	EPAS1	2034	0.265056	1.23E-05
hsa-mir-150	HERC5	51191	0.265086	1.22E-05
hsa-mir-150	TGM2	7052	0.265098	1.22E-05
hsa-mir-150	CARD9	64170	0.265282	1.20E-05
hsa-mir-150	AEBP1	165	0.265307	1.20E-05
hsa-mir-150	PDE1B	5153	0.265331	1.20E-05
hsa-mir-150	ABCA1	19	0.265336	1.20E-05
hsa-mir-150	EBF2	64641	0.265350	1.20E-05
hsa-mir-150	DYSF	8291	0.265351	1.20E-05
hsa-mir-150	SNX8	29886	0.265511	1.18E-05
hsa-mir-150	PDLIM2	64236	0.265808	1.16E-05
hsa-mir-150	IDI1	3422	0.265971	1.14E-05
hsa-mir-150	RNF217	154214	0.266029	1.14E-05
hsa-mir-150	JOSD2	126119	0.266178	1.12E-05
hsa-mir-150	PLEK2	26499	0.266425	1.10E-05
hsa-mir-150	C12orf70	341346	0.267082	1.05E-05
hsa-mir-150	RPL23AP82	284942	0.267084	1.05E-05
hsa-mir-150	SYNE1	23345	0.267186	1.04E-05
hsa-mir-150	KLF6	1316	0.267262	1.03E-05
hsa-mir-150	CAPZB	832	0.267287	1.03E-05
hsa-mir-150	PAPPA	5069	0.267386	1.02E-05
hsa-mir-150	RIMBP3	85376	0.267551	1.01E-05
hsa-mir-150	KLF9	687	0.267603	1.00E-05
hsa-mir-150	APOBEC3C	27350	0.267647	1.00E-05
hsa-mir-150	IL3RA	3563	0.267986	9.73E-06
hsa-mir-150	FAM167B	84734	0.267988	9.73E-06
hsa-mir-150	ADAM12	8038	0.268156	9.60E-06
hsa-mir-150	FXYD5	53827	0.268297	9.49E-06
hsa-mir-150	STX4	6810	0.268467	9.37E-06
hsa-mir-150	RGS2	5997	0.268565	9.29E-06
hsa-mir-150	HBEGF	1839	0.268635	9.24E-06
hsa-mir-150	CHCHD10	400916	0.268669	9.22E-06
hsa-mir-150	IQGAP1	8826	0.268694	9.20E-06
hsa-mir-150	C19orf66	55337	0.268792	9.13E-06
hsa-mir-150	CHSY3	337876	0.268972	9.00E-06
hsa-mir-150	CTSH	1512	0.269106	8.90E-06
hsa-mir-150	CALCRL	10203	0.269161	8.86E-06
hsa-mir-150	TBC1D22A	25771	0.269313	8.76E-06
hsa-mir-150	FILIP1L	11259	0.269569	8.58E-06
hsa-mir-150	HPGDS	27306	0.270061	8.25E-06
hsa-mir-150	ZCCHC6	79670	0.270337	8.07E-06
hsa-mir-150	GADD45B	4616	0.270389	8.04E-06
hsa-mir-150	AIM1	202	0.270398	8.03E-06
hsa-mir-150	CYSLTR2	57105	0.270416	8.02E-06
hsa-mir-150	MAPK11	5600	0.270606	7.90E-06
hsa-mir-150	PLA2G15	23659	0.270608	7.90E-06

hsa-mir-150	FKBP5	2289	0.270827	7.76E-06
hsa-mir-150	C11orf80	79703	0.271022	7.64E-06
hsa-mir-150	CBX7	23492	0.271035	7.63E-06
hsa-mir-150	PTPLAD2	401494	0.271048	7.62E-06
hsa-mir-150	EDEM1	9695	0.271273	7.49E-06
hsa-mir-150	DENND1C	79958	0.271305	7.47E-06
hsa-mir-150	MMRN1	22915	0.271587	7.30E-06
hsa-mir-150	UBAP1	51271	0.271610	7.28E-06
hsa-mir-150	TMEM50A	23585	0.271680	7.24E-06
hsa-mir-150	FPR2	2358	0.271685	7.24E-06
hsa-mir-150	ICAM2	3384	0.271686	7.24E-06
hsa-mir-150	SLC28A3	64078	0.271888	7.12E-06
hsa-mir-150	PTPRO	5800	0.271939	7.09E-06
hsa-mir-150	CXCL1	2919	0.272083	7.01E-06
hsa-mir-150	TTC39B	158219	0.272086	7.01E-06
hsa-mir-150	ARHGAP21	57584	0.272087	7.01E-06
hsa-mir-150	FAM70A	55026	0.272264	6.91E-06
hsa-mir-150	CEBPA	1050	0.272364	6.86E-06
hsa-mir-150	RIN3	79890	0.272488	6.79E-06
hsa-mir-150	APOBEC3A	200315	0.272817	6.61E-06
hsa-mir-150	PTGIS	5740	0.273309	6.35E-06
hsa-mir-150	ALDH1L2	160428	0.273311	6.35E-06
hsa-mir-150	CIDEC	63924	0.273370	6.32E-06
hsa-mir-150	ZEB1	6935	0.273413	6.30E-06
hsa-mir-150	ITPRIPL2	162073	0.273486	6.26E-06
hsa-mir-150	B4GALT1	2683	0.273501	6.25E-06
hsa-mir-150	HEG1	57493	0.273709	6.15E-06
hsa-mir-150	PAX5	5079	0.273814	6.10E-06
hsa-mir-150	IFNAR2	3455	0.273951	6.03E-06
hsa-mir-150	GYG1	2992	0.274066	5.97E-06
hsa-mir-150	TXNDC3	51314	0.274102	5.96E-06
hsa-mir-150	S1PR1	1901	0.274173	5.92E-06
hsa-mir-150	MAFF	23764	0.274260	5.88E-06
hsa-mir-150	MICAL2	9645	0.274294	5.86E-06
hsa-mir-150	RHBDL2	54933	0.274339	5.84E-06
hsa-mir-150	SIDT1	54847	0.274405	5.81E-06
hsa-mir-150	GJD3	125111	0.274441	5.79E-06
hsa-mir-150	PLRG1	5356	0.274468	5.78E-06
hsa-mir-150	SP110	3431	0.274574	5.73E-06
hsa-mir-150	CCL26	10344	0.274598	5.72E-06
hsa-mir-150	MMP2	4313	0.274640	5.70E-06
hsa-mir-150	CNRIP1	25927	0.274886	5.59E-06
hsa-mir-150	RIPK3	11035	0.274932	5.57E-06
hsa-mir-150	RASGRP1	10125	0.275150	5.47E-06
hsa-mir-150	SAP30	8819	0.275175	5.46E-06
hsa-mir-150	MARCKS	4082	0.275426	5.35E-06
hsa-mir-150	ASGR2	433	0.275567	5.28E-06
hsa-mir-150	CNN2	1265	0.275683	5.23E-06
hsa-mir-150	ABCC9	10060	0.275785	5.19E-06
hsa-mir-150	LAMP3	27074	0.276226	5.01E-06
hsa-mir-150	TAGLN	6876	0.276347	4.96E-06
hsa-mir-150	TNFRSF8	943	0.276403	4.93E-06
hsa-mir-150	MITF	4286	0.276412	4.93E-06
hsa-mir-150	CD58	965	0.276576	4.87E-06

hsa-mir-150	PLB1	151056	0.276720	4.81E-06
hsa-mir-150	CD93	22918	0.276734	4.80E-06
hsa-mir-150	TRPM2	7226	0.276958	4.72E-06
hsa-mir-150	C3orf64	285203	0.277419	4.54E-06
hsa-mir-150	ATP10D	57205	0.277680	4.44E-06
hsa-mir-150	TDRD7	23424	0.277766	4.41E-06
hsa-mir-150	ITGAD	3681	0.277875	4.37E-06
hsa-mir-150	ANKRD44	91526	0.277878	4.37E-06
hsa-mir-150	PDE1A	5136	0.278388	4.19E-06
hsa-mir-150	PARP12	64761	0.278597	4.12E-06
hsa-mir-150	CARD8	22900	0.278704	4.08E-06
hsa-mir-150	MR1	3140	0.278717	4.08E-06
hsa-mir-150	MT1L	4500	0.278857	4.03E-06
hsa-mir-150	AMDHD2	51005	0.279050	3.97E-06
hsa-mir-150	LHFP	10186	0.279071	3.96E-06
hsa-mir-150	OAS3	4940	0.279302	3.89E-06
hsa-mir-150	TREML1	340205	0.279478	3.83E-06
hsa-mir-150	ALDH1A3	220	0.279719	3.75E-06
hsa-mir-150	TAOK3	51347	0.279738	3.75E-06
hsa-mir-150	CMTM3	123920	0.279908	3.69E-06
hsa-mir-150	PARM1	25849	0.280075	3.64E-06
hsa-mir-150	RAP1A	5906	0.280147	3.62E-06
hsa-mir-150	PCDHGA12	26025	0.280310	3.57E-06
hsa-mir-150	CXCL16	58191	0.280349	3.56E-06
hsa-mir-150	KAT2B	8850	0.280385	3.55E-06
hsa-mir-150	GBGT1	26301	0.280390	3.55E-06
hsa-mir-150	CTSD	1509	0.280599	3.49E-06
hsa-mir-150	TNF	7124	0.281131	3.34E-06
hsa-mir-150	C22orf9	23313	0.281158	3.33E-06
hsa-mir-150	FKBP15	23307	0.281467	3.24E-06
hsa-mir-150	TWF2	11344	0.282273	3.03E-06
hsa-mir-150	IDO2	169355	0.282644	2.94E-06
hsa-mir-150	XCL1	6375	0.282674	2.93E-06
hsa-mir-150	EGR2	1959	0.282741	2.92E-06
hsa-mir-150	RALB	5899	0.282854	2.89E-06
hsa-mir-150	IFI44L	10964	0.283137	2.82E-06
hsa-mir-150	ADAMTSL1	92949	0.283281	2.79E-06
hsa-mir-150	ABLIM3	22885	0.283287	2.78E-06
hsa-mir-150	GPR150	285601	0.283413	2.75E-06
hsa-mir-150	MEFV	4210	0.283603	2.71E-06
hsa-mir-150	DENND3	22898	0.283633	2.70E-06
hsa-mir-150	CRISPLD2	83716	0.283669	2.70E-06
hsa-mir-150	SQSTM1	8878	0.283692	2.69E-06
hsa-mir-150	SH3BP1	23616	0.283762	2.67E-06
hsa-mir-150	MYL12A	10627	0.283960	2.63E-06
hsa-mir-150	IL2	3558	0.284097	2.60E-06
hsa-mir-150	PLXDC2	84898	0.284102	2.60E-06
hsa-mir-150	PDE3B	5140	0.284134	2.59E-06
hsa-mir-150	S100B	6285	0.284135	2.59E-06
hsa-mir-150	LMF2	91289	0.284139	2.59E-06
hsa-mir-150	SFRP2	6423	0.284265	2.56E-06
hsa-mir-150	DSE	29940	0.284377	2.54E-06
hsa-mir-150	MAP3K5	4217	0.284389	2.54E-06
hsa-mir-150	PLEKHF1	79156	0.284521	2.51E-06

hsa-mir-150	PRR16	51334	0.284521	2.51E-06
hsa-mir-150	EPYC	1833	0.284542	2.50E-06
hsa-mir-150	ELTD1	64123	0.284552	2.50E-06
hsa-mir-150	DHRS9	10170	0.284598	2.49E-06
hsa-mir-150	PDLIM5	10611	0.285051	2.40E-06
hsa-mir-150	ANXA5	308	0.285089	2.39E-06
hsa-mir-150	OBFC2A	64859	0.285119	2.38E-06
hsa-mir-150	KBTBD8	84541	0.285149	2.38E-06
hsa-mir-150	UTS2	10911	0.285166	2.38E-06
hsa-mir-150	SC4MOL	6307	0.285181	2.37E-06
hsa-mir-150	HERC3	8916	0.285229	2.36E-06
hsa-mir-150	GCH1	2643	0.285341	2.34E-06
hsa-mir-150	WDR44	54521	0.285472	2.31E-06
hsa-mir-150	CHIT1	1118	0.285483	2.31E-06
hsa-mir-150	CATSPER1	117144	0.285567	2.30E-06
hsa-mir-150	COL3A1	1281	0.285735	2.26E-06
hsa-mir-150	CLEC12B	387837	0.285766	2.26E-06
hsa-mir-150	CTSC	1075	0.286151	2.18E-06
hsa-mir-150	HAPLN3	145864	0.286199	2.18E-06
hsa-mir-150	FBN1	2200	0.286225	2.17E-06
hsa-mir-150	CD101	9398	0.286227	2.17E-06
hsa-mir-150	SGMS2	166929	0.286384	2.14E-06
hsa-mir-150	VNN3	55350	0.286566	2.11E-06
hsa-mir-150	TNFSF4	7292	0.286695	2.09E-06
hsa-mir-150	SLC2A3	6515	0.286771	2.07E-06
hsa-mir-150	PLCG2	5336	0.286850	2.06E-06
hsa-mir-150	MYH9	4627	0.286877	2.05E-06
hsa-mir-150	ADH1B	125	0.286992	2.03E-06
hsa-mir-150	SCARF1	8578	0.287107	2.01E-06
hsa-mir-150	CLEC14A	161198	0.287312	1.98E-06
hsa-mir-150	COQ2	27235	0.287372	1.97E-06
hsa-mir-150	ECSCR	641700	0.287683	1.92E-06
hsa-mir-150	ASPA	443	0.287751	1.91E-06
hsa-mir-150	ALPK1	80216	0.287838	1.89E-06
hsa-mir-150	ZNF524	147807	0.288070	1.85E-06
hsa-mir-150	DDO	8528	0.288093	1.85E-06
hsa-mir-150	SAMHD1	25939	0.288502	1.79E-06
hsa-mir-150	GRK5	2869	0.288602	1.77E-06
hsa-mir-150	GABRE	2564	0.288688	1.76E-06
hsa-mir-150	THBD	7056	0.289072	1.70E-06
hsa-mir-150	CCL7	6354	0.289312	1.67E-06
hsa-mir-150	NRP1	8829	0.289322	1.66E-06
hsa-mir-150	IFIT2	3433	0.289493	1.64E-06
hsa-mir-150	SCT	6343	0.289952	1.58E-06
hsa-mir-150	RUNX1	861	0.289988	1.57E-06
hsa-mir-150	GPR155	151556	0.290062	1.56E-06
hsa-mir-150	PLK3	1263	0.290072	1.56E-06
hsa-mir-150	CYP1B1	1545	0.290206	1.54E-06
hsa-mir-150	TMSL3	7117	0.290472	1.51E-06
hsa-mir-150	C19orf28	126321	0.290793	1.47E-06
hsa-mir-150	CAMK4	814	0.290866	1.46E-06
hsa-mir-150	RHBDF2	79651	0.290911	1.45E-06
hsa-mir-150	IL1RN	3557	0.291024	1.44E-06
hsa-mir-150	AKAP5	9495	0.291053	1.43E-06

hsa-mir-150	C9orf95	54981	0.291063	1.43E-06
hsa-mir-150	TBXA2R	6915	0.291085	1.43E-06
hsa-mir-150	PRKCH	5583	0.291320	1.40E-06
hsa-mir-150	RASGRF2	5924	0.291439	1.39E-06
hsa-mir-150	NBL1	4681	0.291627	1.36E-06
hsa-mir-150	HTATIP2	10553	0.291921	1.33E-06
hsa-mir-150	CARD17	440068	0.291926	1.33E-06
hsa-mir-150	LRRC15	131578	0.292030	1.32E-06
hsa-mir-150	SERPINB1	1992	0.292149	1.30E-06
hsa-mir-150	SLC43A3	29015	0.292311	1.28E-06
hsa-mir-150	CCR8	1237	0.292501	1.26E-06
hsa-mir-150	KIAA0125	9834	0.292550	1.26E-06
hsa-mir-150	KIR3DL2	3812	0.292583	1.25E-06
hsa-mir-150	PPAP2A	8611	0.293330	1.17E-06
hsa-mir-150	CBR3	874	0.293458	1.16E-06
hsa-mir-150	DNAJC1	64215	0.293588	1.15E-06
hsa-mir-150	POLD4	57804	0.293751	1.13E-06
hsa-mir-150	DOCK11	139818	0.293884	1.12E-06
hsa-mir-150	TMOD2	29767	0.293884	1.12E-06
hsa-mir-150	TFPI2	7980	0.293940	1.11E-06
hsa-mir-150	GTSF1	121355	0.293968	1.11E-06
hsa-mir-150	UBA7	7318	0.294059	1.10E-06
hsa-mir-150	PPPDE2	27351	0.294134	1.09E-06
hsa-mir-150	OAS1	4938	0.294156	1.09E-06
hsa-mir-150	CEACAM1	634	0.294536	1.06E-06
hsa-mir-150	SAT1	6303	0.295004	1.01E-06
hsa-mir-150	NCK1	4690	0.295083	1.01E-06
hsa-mir-150	NFKBIA	4792	0.295268	9.91E-07
hsa-mir-150	SEC24D	9871	0.295605	9.62E-07
hsa-mir-150	LCN2	3934	0.295648	9.58E-07
hsa-mir-150	DIO2	1734	0.296026	9.27E-07
hsa-mir-150	RNF166	115992	0.296027	9.27E-07
hsa-mir-150	BANK1	55024	0.296425	8.95E-07
hsa-mir-150	SP100	6672	0.296431	8.94E-07
hsa-mir-150	F13A1	2162	0.296802	8.65E-07
hsa-mir-150	CYBA	1535	0.296893	8.58E-07
hsa-mir-150	DEF6	50619	0.297282	8.29E-07
hsa-mir-150	LILRB3	11025	0.297318	8.26E-07
hsa-mir-150	STEAP4	79689	0.297334	8.25E-07
hsa-mir-150	RAB33A	9363	0.297669	8.01E-07
hsa-mir-150	CORO7	79585	0.297907	7.84E-07
hsa-mir-150	ITGBL1	9358	0.298246	7.61E-07
hsa-mir-150	MYD88	4615	0.298352	7.54E-07
hsa-mir-150	CSF2	1437	0.298378	7.52E-07
hsa-mir-150	RAB39	54734	0.299073	7.07E-07
hsa-mir-150	FCRL6	343413	0.299386	6.87E-07
hsa-mir-150	LOC401463	401463	0.299588	6.75E-07
hsa-mir-150	GRN	2896	0.299619	6.73E-07
hsa-mir-150	C5orf46	389336	0.299730	6.66E-07
hsa-mir-150	PPARG	5468	0.300087	6.45E-07
hsa-mir-150	TMEM158	25907	0.300301	6.33E-07
hsa-mir-150	IL6	3569	0.300356	6.30E-07
hsa-mir-150	STAT3	6774	0.300430	6.26E-07
hsa-mir-150	ZMYND15	84225	0.300443	6.25E-07

hsa-mir-150	NFATC1	4772	0.300502	6.22E-07
hsa-mir-150	C6orf114	85411	0.300624	6.15E-07
hsa-mir-150	IRF9	10379	0.300710	6.10E-07
hsa-mir-150	RAB37	326624	0.300875	6.01E-07
hsa-mir-150	SLC25A28	81894	0.301042	5.92E-07
hsa-mir-150	FAM26E	254228	0.301228	5.82E-07
hsa-mir-150	TICAM1	148022	0.301295	5.79E-07
hsa-mir-150	S100A9	6280	0.301444	5.71E-07
hsa-mir-150	SERPINB9	5272	0.301945	5.46E-07
hsa-mir-150	ABI1	10006	0.302084	5.39E-07
hsa-mir-150	IKBKE	9641	0.302811	5.05E-07
hsa-mir-150	C13orf33	84935	0.302843	5.03E-07
hsa-mir-150	IL27	246778	0.302911	5.00E-07
hsa-mir-150	EHBP1L1	254102	0.303050	4.94E-07
hsa-mir-150	ARSB	411	0.303241	4.85E-07
hsa-mir-150	KAL1	3730	0.303243	4.85E-07
hsa-mir-150	TUSC5	286753	0.303303	4.83E-07
hsa-mir-150	GBP6	163351	0.303474	4.75E-07
hsa-mir-150	LMO2	4005	0.303496	4.74E-07
hsa-mir-150	ARRB1	408	0.303580	4.71E-07
hsa-mir-150	MFRP	83552	0.303826	4.60E-07
hsa-mir-150	CFH	3075	0.303957	4.55E-07
hsa-mir-150	GPX3	2878	0.304030	4.52E-07
hsa-mir-150	TPM2	7169	0.304167	4.46E-07
hsa-mir-150	CCR3	1232	0.304318	4.40E-07
hsa-mir-150	LILRP2	79166	0.304350	4.39E-07
hsa-mir-150	NTAN1	123803	0.304368	4.38E-07
hsa-mir-150	RGS4	5999	0.304373	4.38E-07
hsa-mir-150	CCDC88B	283234	0.304530	4.31E-07
hsa-mir-150	AQP1	358	0.304560	4.30E-07
hsa-mir-150	SLC38A5	92745	0.304728	4.24E-07
hsa-mir-150	ARSA	410	0.304905	4.17E-07
hsa-mir-150	CD36	948	0.305000	4.13E-07
hsa-mir-150	DUSP10	11221	0.305025	4.12E-07
hsa-mir-150	CALHM2	51063	0.305419	3.98E-07
hsa-mir-150	ST8SIA1	6489	0.305611	3.91E-07
hsa-mir-150	FAM180A	389558	0.305699	3.88E-07
hsa-mir-150	JAKMIP1	152789	0.306174	3.71E-07
hsa-mir-150	ABCC3	8714	0.306877	3.48E-07
hsa-mir-150	JOSD1	9929	0.306947	3.46E-07
hsa-mir-150	LRRC33	375387	0.307004	3.44E-07
hsa-mir-150	ICAM3	3385	0.307274	3.35E-07
hsa-mir-150	CTHRC1	115908	0.307312	3.34E-07
hsa-mir-150	TRIM47	91107	0.307381	3.32E-07
hsa-mir-150	PTCRA	171558	0.307826	3.19E-07
hsa-mir-150	TACC1	6867	0.308004	3.14E-07
hsa-mir-150	ADAMTS4	9507	0.308407	3.02E-07
hsa-mir-150	TPST2	8459	0.308519	2.99E-07
hsa-mir-150	C10orf11	83938	0.308809	2.91E-07
hsa-mir-150	SLC12A8	84561	0.308940	2.87E-07
hsa-mir-150	FTH1	2495	0.309196	2.81E-07
hsa-mir-150	GUCY1B3	2983	0.309218	2.80E-07
hsa-mir-150	CASS4	57091	0.309817	2.65E-07
hsa-mir-150	MARCO	8685	0.309979	2.61E-07

hsa-mir-150	ARHGAP18	93663	0.310145	2.57E-07
hsa-mir-150	LYN	4067	0.310385	2.51E-07
hsa-mir-150	STAT5A	6776	0.310419	2.50E-07
hsa-mir-150	CBFA2T3	863	0.310818	2.41E-07
hsa-mir-150	SDCBP	6386	0.310916	2.39E-07
hsa-mir-150	SERPING1	710	0.311032	2.37E-07
hsa-mir-150	ANTXR2	118429	0.311077	2.36E-07
hsa-mir-150	ENTPD1	953	0.311159	2.34E-07
hsa-mir-150	TCIRG1	10312	0.311509	2.26E-07
hsa-mir-150	RSAD2	91543	0.312245	2.11E-07
hsa-mir-150	TRADD	8717	0.312257	2.11E-07
hsa-mir-150	HAMP	57817	0.312300	2.10E-07
hsa-mir-150	LOC100129066	100129066	0.312331	2.09E-07
hsa-mir-150	TRANK1	9881	0.312589	2.04E-07
hsa-mir-150	FAM70B	348013	0.312916	1.98E-07
hsa-mir-150	RRAS	6237	0.313205	1.93E-07
hsa-mir-150	KCNE4	23704	0.313414	1.89E-07
hsa-mir-150	COL5A2	1290	0.313715	1.84E-07
hsa-mir-150	GEM	2669	0.313798	1.82E-07
hsa-mir-150	CYTSB	92521	0.314485	1.71E-07
hsa-mir-150	TGFB1I1	7041	0.314521	1.70E-07
hsa-mir-150	BTN3A1	11119	0.314523	1.70E-07
hsa-mir-150	EDNRA	1909	0.314536	1.70E-07
hsa-mir-150	BTN3A3	10384	0.314543	1.70E-07
hsa-mir-150	IGSF21	84966	0.314611	1.69E-07
hsa-mir-150	SUN2	25777	0.314657	1.68E-07
hsa-mir-150	SELP	6403	0.314746	1.67E-07
hsa-mir-150	BCL3	602	0.314845	1.65E-07
hsa-mir-150	JAK3	3718	0.314881	1.65E-07
hsa-mir-150	FAM115C	285966	0.315243	1.59E-07
hsa-mir-150	GFOD1	54438	0.315347	1.57E-07
hsa-mir-150	CASP4	837	0.315428	1.56E-07
hsa-mir-150	PSME1	5720	0.315477	1.56E-07
hsa-mir-150	ANKRD55	79722	0.315576	1.54E-07
hsa-mir-150	CCR6	1235	0.315596	1.54E-07
hsa-mir-150	TAPBPL	55080	0.315913	1.49E-07
hsa-mir-150	ELMO1	9844	0.315955	1.49E-07
hsa-mir-150	CTSA	5476	0.316330	1.43E-07
hsa-mir-150	SPSB1	80176	0.316647	1.39E-07
hsa-mir-150	C10orf128	170371	0.316708	1.38E-07
hsa-mir-150	P2RY14	9934	0.317209	1.32E-07
hsa-mir-150	C2orf85	285093	0.317255	1.31E-07
hsa-mir-150	PVRIG	79037	0.317353	1.30E-07
hsa-mir-150	ARRB2	409	0.317468	1.29E-07
hsa-mir-150	SAA1	6288	0.317624	1.27E-07
hsa-mir-150	PSAP	5660	0.317649	1.26E-07
hsa-mir-150	MEIS3	56917	0.317697	1.26E-07
hsa-mir-150	EMR4P	326342	0.317790	1.25E-07
hsa-mir-150	FBXO32	114907	0.318033	1.22E-07
hsa-mir-150	PROCR	10544	0.318192	1.20E-07
hsa-mir-150	TMEM86A	144110	0.318642	1.15E-07
hsa-mir-150	WISP1	8840	0.318732	1.14E-07
hsa-mir-150	SLFN12L	342615	0.318879	1.12E-07
hsa-mir-150	ACSL5	51703	0.319124	1.10E-07

hsa-mir-150	BLNK	29760	0.319363	1.07E-07
hsa-mir-150	ECM1	1893	0.319397	1.07E-07
hsa-mir-150	ABCB1	5243	0.319767	1.03E-07
hsa-mir-150	C17orf88	23591	0.320140	9.94E-08
hsa-mir-150	CNR2	1269	0.321038	9.12E-08
hsa-mir-150	FOXF1	2294	0.321168	9.00E-08
hsa-mir-150	TFEB	7942	0.321207	8.97E-08
hsa-mir-150	ASB2	51676	0.321631	8.61E-08
hsa-mir-150	OAS2	4939	0.321705	8.54E-08
hsa-mir-150	C1orf200	644997	0.321939	8.35E-08
hsa-mir-150	OSMR	9180	0.322237	8.11E-08
hsa-mir-150	SGPP1	81537	0.322432	7.96E-08
hsa-mir-150	RILP	83547	0.322934	7.58E-08
hsa-mir-150	HRH2	3274	0.323378	7.26E-08
hsa-mir-150	HSPA6	3310	0.323476	7.19E-08
hsa-mir-150	LXN	56925	0.323521	7.16E-08
hsa-mir-150	LRMP	4033	0.323600	7.10E-08
hsa-mir-150	AKNA	80709	0.323703	7.03E-08
hsa-mir-150	KLRC4	8302	0.323785	6.97E-08
hsa-mir-150	SLC2A6	11182	0.324061	6.79E-08
hsa-mir-150	KCNQ5	56479	0.324419	6.55E-08
hsa-mir-150	TMEM71	137835	0.324512	6.49E-08
hsa-mir-150	MOBKL2C	148932	0.324566	6.46E-08
hsa-mir-150	CD209	30835	0.325271	6.03E-08
hsa-mir-150	HHEX	3087	0.325424	5.94E-08
hsa-mir-150	GPR120	338557	0.325934	5.64E-08
hsa-mir-150	IL33	90865	0.326010	5.60E-08
hsa-mir-150	PARP9	83666	0.326319	5.43E-08
hsa-mir-150	IFI16	3428	0.326401	5.39E-08
hsa-mir-150	CLNK	116449	0.326850	5.15E-08
hsa-mir-150	CXCR5	643	0.326921	5.12E-08
hsa-mir-150	OAZ1	4946	0.327238	4.96E-08
hsa-mir-150	EAF2	55840	0.327394	4.88E-08
hsa-mir-150	HTR2B	3357	0.327446	4.86E-08
hsa-mir-150	FAM69A	388650	0.327560	4.80E-08
hsa-mir-150	GREM1	26585	0.327641	4.77E-08
hsa-mir-150	TPSB2	64499	0.327831	4.68E-08
hsa-mir-150	CCDC69	26112	0.327972	4.61E-08
hsa-mir-150	SMAP2	64744	0.328001	4.60E-08
hsa-mir-150	SLC16A3	9123	0.328042	4.58E-08
hsa-mir-150	ALPK2	115701	0.328067	4.57E-08
hsa-mir-150	ACTR3	10096	0.328070	4.57E-08
hsa-mir-150	GCET2	257144	0.328226	4.50E-08
hsa-mir-150	RNF19B	127544	0.328309	4.46E-08
hsa-mir-150	KIR3DX1	90011	0.328483	4.38E-08
hsa-mir-150	SAA2	6289	0.328564	4.35E-08
hsa-mir-150	TRIM34	53840	0.328585	4.34E-08
hsa-mir-150	SEP4	5414	0.328640	4.31E-08
hsa-mir-150	SKAP2	8935	0.328920	4.20E-08
hsa-mir-150	DDX60	55601	0.329018	4.15E-08
hsa-mir-150	SNAI3	333929	0.329095	4.12E-08
hsa-mir-150	DNAJC5B	85479	0.329127	4.11E-08
hsa-mir-150	RAB8B	51762	0.329540	3.94E-08
hsa-mir-150	CTSL1	1514	0.329577	3.93E-08

hsa-mir-150	TSPO	706	0.329791	3.85E-08
hsa-mir-150	FGD3	89846	0.329843	3.83E-08
hsa-mir-150	TDO2	6999	0.329991	3.77E-08
hsa-mir-150	HNMT	3176	0.330031	3.75E-08
hsa-mir-150	HTR7	3363	0.330193	3.69E-08
hsa-mir-150	KMO	8564	0.330371	3.63E-08
hsa-mir-150	ACP2	53	0.330536	3.57E-08
hsa-mir-150	MMP12	4321	0.330569	3.56E-08
hsa-mir-150	PLAC8	51316	0.330714	3.51E-08
hsa-mir-150	TFPI	7035	0.330964	3.42E-08
hsa-mir-150	CALCOCO2	10241	0.331010	3.40E-08
hsa-mir-150	CCDC80	151887	0.331263	3.32E-08
hsa-mir-150	SECTM1	6398	0.331278	3.31E-08
hsa-mir-150	LPAR5	57121	0.331319	3.30E-08
hsa-mir-150	ASPN	54829	0.331342	3.29E-08
hsa-mir-150	KIAA1539	80256	0.331370	3.28E-08
hsa-mir-150	HERPUD1	9709	0.331530	3.23E-08
hsa-mir-150	IGF1	3479	0.331605	3.20E-08
hsa-mir-150	FCER1A	2205	0.331695	3.18E-08
hsa-mir-150	GPR132	29933	0.331812	3.14E-08
hsa-mir-150	AOC3	8639	0.331844	3.13E-08
hsa-mir-150	GPR4	2828	0.331850	3.13E-08
hsa-mir-150	MIR155HG	114614	0.331945	3.10E-08
hsa-mir-150	MYLK	4638	0.331970	3.09E-08
hsa-mir-150	BLK	640	0.332108	3.05E-08
hsa-mir-150	THBS1	7057	0.332661	2.88E-08
hsa-mir-150	TLR3	7098	0.332680	2.87E-08
hsa-mir-150	ACSL1	2180	0.333057	2.77E-08
hsa-mir-150	CTSB	1508	0.333182	2.73E-08
hsa-mir-150	CLEC6A	93978	0.333362	2.68E-08
hsa-mir-150	IFIH1	64135	0.333535	2.64E-08
hsa-mir-150	KCNMB1	3779	0.333599	2.62E-08
hsa-mir-150	IFNGR1	3459	0.333719	2.59E-08
hsa-mir-150	CCL13	6357	0.333818	2.56E-08
hsa-mir-150	MFNG	4242	0.334355	2.43E-08
hsa-mir-150	CRYBB1	1414	0.334677	2.35E-08
hsa-mir-150	ZNFX1	57169	0.335050	2.26E-08
hsa-mir-150	LRRC55	219527	0.335103	2.25E-08
hsa-mir-150	NT5E	4907	0.335334	2.19E-08
hsa-mir-150	IL12B	3593	0.335558	2.15E-08
hsa-mir-150	ACP5	54	0.335668	2.12E-08
hsa-mir-150	ARHGAP25	9938	0.336238	2.00E-08
hsa-mir-150	GJB2	2706	0.336344	1.98E-08
hsa-mir-150	LGALS12	85329	0.336749	1.90E-08
hsa-mir-150	UPP1	7378	0.337787	1.71E-08
hsa-mir-150	RAB27A	5873	0.337818	1.70E-08
hsa-mir-150	PRDM8	56978	0.337888	1.69E-08
hsa-mir-150	C5orf56	441108	0.338256	1.63E-08
hsa-mir-150	C21orf63	59271	0.339203	1.47E-08
hsa-mir-150	NAAA	27163	0.339719	1.40E-08
hsa-mir-150	SLC46A3	283537	0.339818	1.38E-08
hsa-mir-150	FLJ36031	168455	0.340506	1.29E-08
hsa-mir-150	LYSMD2	256586	0.340653	1.27E-08
hsa-mir-150	TNFSF8	944	0.340891	1.24E-08

hsa-mir-150	OGFRL1	79627	0.341405	1.17E-08
hsa-mir-150	C10orf55	414236	0.342496	1.05E-08
hsa-mir-150	KCNK13	56659	0.342713	1.02E-08
hsa-mir-150	FTL	2512	0.342777	1.02E-08
hsa-mir-150	S100A8	6279	0.342913	1.00E-08
hsa-mir-150	GPR141	353345	0.343091	9.83E-09
hsa-mir-150	RAB32	10981	0.343235	9.68E-09
hsa-mir-150	POSTN	10631	0.343346	9.57E-09
hsa-mir-150	SSC5D	284297	0.343450	9.46E-09
hsa-mir-150	STK17B	9262	0.343535	9.38E-09
hsa-mir-150	PAG1	55824	0.343859	9.06E-09
hsa-mir-150	C21orf96	80215	0.343887	9.04E-09
hsa-mir-150	RNF213	57674	0.343916	9.01E-09
hsa-mir-150	C15orf48	84419	0.344008	8.92E-09
hsa-mir-150	LPAR6	10161	0.344116	8.82E-09
hsa-mir-150	CD300E	342510	0.344427	8.54E-09
hsa-mir-150	CECR1	51816	0.344860	8.16E-09
hsa-mir-150	EMB	133418	0.345052	7.99E-09
hsa-mir-150	VDR	7421	0.345190	7.88E-09
hsa-mir-150	CSTA	1475	0.345352	7.74E-09
hsa-mir-150	DBI	1622	0.345559	7.57E-09
hsa-mir-150	PTGFR	5737	0.345596	7.54E-09
hsa-mir-150	CHI3L2	1117	0.345643	7.51E-09
hsa-mir-150	HLX	3142	0.346301	7.00E-09
hsa-mir-150	HAR1A	768096	0.346397	6.93E-09
hsa-mir-150	C10orf10	11067	0.346781	6.65E-09
hsa-mir-150	IL22RA2	116379	0.347283	6.31E-09
hsa-mir-150	DCN	1634	0.347300	6.30E-09
hsa-mir-150	CFB	629	0.347654	6.06E-09
hsa-mir-150	SLC11A1	6556	0.347728	6.01E-09
hsa-mir-150	GM2A	2760	0.347911	5.90E-09
hsa-mir-150	GGTA1	2681	0.347931	5.89E-09
hsa-mir-150	RTP4	64108	0.348004	5.84E-09
hsa-mir-150	C19orf38	255809	0.348222	5.70E-09
hsa-mir-150	CRLF3	51379	0.348275	5.67E-09
hsa-mir-150	ATP6V1B2	526	0.348563	5.50E-09
hsa-mir-150	OSTM1	28962	0.349096	5.20E-09
hsa-mir-150	SPIB	6689	0.349494	4.98E-09
hsa-mir-150	PSME2	5721	0.349608	4.92E-09
hsa-mir-150	CSGALNACT2	55454	0.349786	4.82E-09
hsa-mir-150	OLFML2B	25903	0.349915	4.76E-09
hsa-mir-150	SCO2	9997	0.349931	4.75E-09
hsa-mir-150	FBXO6	26270	0.349951	4.74E-09
hsa-mir-150	SIRPD	128646	0.350124	4.65E-09
hsa-mir-150	MYO5A	4644	0.350170	4.63E-09
hsa-mir-150	HTRA4	203100	0.350441	4.50E-09
hsa-mir-150	CEBPB	1051	0.351007	4.23E-09
hsa-mir-150	SIPA1	6494	0.351336	4.08E-09
hsa-mir-150	RFTN1	23180	0.351836	3.87E-09
hsa-mir-150	TNFSF10	8743	0.351949	3.82E-09
hsa-mir-150	CACNA2D4	93589	0.351960	3.82E-09
hsa-mir-150	CCL18	6362	0.352169	3.73E-09
hsa-mir-150	PLN	5350	0.352299	3.68E-09
hsa-mir-150	DDX60L	91351	0.352426	3.63E-09

hsa-mir-150	ARRDC5	645432	0.352675	3.53E-09
hsa-mir-150	TLR10	81793	0.353352	3.28E-09
hsa-mir-150	RASSF5	83593	0.353363	3.28E-09
hsa-mir-150	C5orf39	389289	0.354385	2.93E-09
hsa-mir-150	NAGK	55577	0.354910	2.77E-09
hsa-mir-150	IFIT3	3437	0.355075	2.72E-09
hsa-mir-150	ACTA2	59	0.355455	2.61E-09
hsa-mir-150	TM6SF1	53346	0.355461	2.60E-09
hsa-mir-150	CREB3L3	84699	0.355705	2.54E-09
hsa-mir-150	DTX3L	151636	0.355739	2.53E-09
hsa-mir-150	CARD6	84674	0.355752	2.52E-09
hsa-mir-150	PDLIM3	27295	0.355804	2.51E-09
hsa-mir-150	SFMBT2	57713	0.355820	2.50E-09
hsa-mir-150	DAB2	1601	0.355894	2.48E-09
hsa-mir-150	FBP1	2203	0.356264	2.38E-09
hsa-mir-150	MOBKL2A	126308	0.357174	2.16E-09
hsa-mir-150	TNFRSF14	8764	0.357188	2.15E-09
hsa-mir-150	AXL	558	0.357489	2.08E-09
hsa-mir-150	PLD4	122618	0.357509	2.08E-09
hsa-mir-150	FAM105A	54491	0.357592	2.06E-09
hsa-mir-150	SELM	140606	0.357604	2.06E-09
hsa-mir-150	CELF2	10659	0.357830	2.01E-09
hsa-mir-150	BST1	683	0.357892	1.99E-09
hsa-mir-150	IFI27	3429	0.358163	1.93E-09
hsa-mir-150	TOM1	10043	0.358266	1.91E-09
hsa-mir-150	GNAI2	2771	0.358370	1.89E-09
hsa-mir-150	NTM	50863	0.358506	1.86E-09
hsa-mir-150	RAB42	115273	0.358518	1.86E-09
hsa-mir-150	LUM	4060	0.358599	1.84E-09
hsa-mir-150	PTPRE	5791	0.358756	1.81E-09
hsa-mir-150	OASL	8638	0.358772	1.81E-09
hsa-mir-150	DHX58	79132	0.359071	1.75E-09
hsa-mir-150	SERPINE1	5054	0.360032	1.57E-09
hsa-mir-150	FCRL4	83417	0.360045	1.57E-09
hsa-mir-150	MBNL1	4154	0.360095	1.56E-09
hsa-mir-150	TNFRSF18	8784	0.360223	1.54E-09
hsa-mir-150	LAP3	51056	0.360270	1.53E-09
hsa-mir-150	PLEKHO1	51177	0.360475	1.50E-09
hsa-mir-150	RELB	5971	0.360520	1.49E-09
hsa-mir-150	PLXNC1	10154	0.360625	1.47E-09
hsa-mir-150	GYPC	2995	0.360638	1.47E-09
hsa-mir-150	NR3C1	2908	0.360891	1.43E-09
hsa-mir-150	SPOCD1	90853	0.361016	1.41E-09
hsa-mir-150	SVEP1	79987	0.361077	1.40E-09
hsa-mir-150	MAFB	9935	0.362540	1.19E-09
hsa-mir-150	PSTPIP2	9050	0.362673	1.17E-09
hsa-mir-150	GPR15	2838	0.362753	1.16E-09
hsa-mir-150	G0S2	50486	0.362974	1.13E-09
hsa-mir-150	ISG20	3669	0.363170	1.11E-09
hsa-mir-150	SFRP4	6424	0.363569	1.06E-09
hsa-mir-150	FOLR2	2350	0.364025	1.00E-09
hsa-mir-150	ACVRL1	94	0.364184	9.86E-10
hsa-mir-150	GPR82	27197	0.364298	9.74E-10
hsa-mir-150	CMAH	8418	0.364368	9.66E-10

hsa-mir-150	NMI	9111	0.364519	9.50E-10
hsa-mir-150	LOX	4015	0.364670	9.34E-10
hsa-mir-150	HIVEP3	59269	0.364714	9.29E-10
hsa-mir-150	IL9R	3581	0.364979	9.01E-10
hsa-mir-150	PARP14	54625	0.365062	8.93E-10
hsa-mir-150	NCR1	9437	0.365329	8.66E-10
hsa-mir-150	RGS13	6003	0.365448	8.55E-10
hsa-mir-150	FAM20A	54757	0.365449	8.55E-10
hsa-mir-150	INMT	11185	0.365673	8.33E-10
hsa-mir-150	NUPR1	26471	0.365756	8.25E-10
hsa-mir-150	COTL1	23406	0.365795	8.22E-10
hsa-mir-150	MARCH3	115123	0.366352	7.71E-10
hsa-mir-150	SBNO2	22904	0.366401	7.67E-10
hsa-mir-150	CCL17	6361	0.366485	7.60E-10
hsa-mir-150	PLSCR1	5359	0.366741	7.38E-10
hsa-mir-150	ABCD2	225	0.366955	7.20E-10
hsa-mir-150	IL1B	3553	0.367486	6.78E-10
hsa-mir-150	ANKRD58	347454	0.367549	6.73E-10
hsa-mir-150	HPSE	10855	0.367838	6.51E-10
hsa-mir-150	CD300LB	124599	0.367881	6.48E-10
hsa-mir-150	EFEMP1	2202	0.367941	6.43E-10
hsa-mir-150	C11orf75	56935	0.368137	6.29E-10
hsa-mir-150	SERPINA1	5265	0.368311	6.17E-10
hsa-mir-150	SCML4	256380	0.369989	5.08E-10
hsa-mir-150	NFKBIE	4794	0.370765	4.65E-10
hsa-mir-150	ACTB	60	0.370775	4.64E-10
hsa-mir-150	ZNF804A	91752	0.370981	4.53E-10
hsa-mir-150	FGF1	2246	0.371566	4.24E-10
hsa-mir-150	KIR3DL1	3811	0.372296	3.89E-10
hsa-mir-150	MBD2	8932	0.372407	3.84E-10
hsa-mir-150	RTN1	6252	0.372543	3.78E-10
hsa-mir-150	MT2A	4502	0.372628	3.74E-10
hsa-mir-150	PODN	127435	0.373239	3.49E-10
hsa-mir-150	C8orf80	389643	0.373650	3.32E-10
hsa-mir-150	FAS	355	0.374143	3.14E-10
hsa-mir-150	ITGA11	22801	0.374252	3.10E-10
hsa-mir-150	FCGR1C	100132417	0.374264	3.09E-10
hsa-mir-150	STAT1	6772	0.374327	3.07E-10
hsa-mir-150	MATK	4145	0.374373	3.05E-10
hsa-mir-150	ZNF80	7634	0.374460	3.02E-10
hsa-mir-150	MOXD1	26002	0.375013	2.83E-10
hsa-mir-150	COPZ2	51226	0.375120	2.80E-10
hsa-mir-150	VNN1	8876	0.375383	2.71E-10
hsa-mir-150	SH2D3C	10044	0.375420	2.70E-10
hsa-mir-150	SH3BGRL3	83442	0.375898	2.55E-10
hsa-mir-150	GATA3	2625	0.375932	2.54E-10
hsa-mir-150	CHST11	50515	0.376090	2.50E-10
hsa-mir-150	CAPG	822	0.376135	2.48E-10
hsa-mir-150	C15orf53	400359	0.376147	2.48E-10
hsa-mir-150	LIPA	3988	0.376367	2.42E-10
hsa-mir-150	TMEM140	55281	0.377056	2.23E-10
hsa-mir-150	CLEC1A	51267	0.377162	2.20E-10
hsa-mir-150	VMO1	284013	0.379159	1.74E-10
hsa-mir-150	TNFAIP8	25816	0.379544	1.66E-10

hsa-mir-150	FLVCR2	55640	0.379870	1.60E-10
hsa-mir-150	PDZK1IP1	10158	0.380197	1.53E-10
hsa-mir-150	APOBEC3H	164668	0.380367	1.50E-10
hsa-mir-150	ENTHD1	150350	0.380615	1.46E-10
hsa-mir-150	GK	2710	0.380938	1.40E-10
hsa-mir-150	RAMP3	10268	0.381011	1.39E-10
hsa-mir-150	XAF1	54739	0.381247	1.35E-10
hsa-mir-150	SIRPA	140885	0.381291	1.35E-10
hsa-mir-150	MICB	4277	0.381685	1.28E-10
hsa-mir-150	CD274	29126	0.381716	1.28E-10
hsa-mir-150	VENTX	27287	0.382327	1.19E-10
hsa-mir-150	TMEM176A	55365	0.382419	1.18E-10
hsa-mir-150	PMP22	5376	0.382723	1.13E-10
hsa-mir-150	SPP1	6696	0.382926	1.11E-10
hsa-mir-150	KLRD1	3824	0.383521	1.03E-10
hsa-mir-150	TNFSF14	8740	0.383617	1.02E-10
hsa-mir-150	CH25H	9023	0.383625	1.02E-10
hsa-mir-150	PPP3CC	5533	0.384022	9.69E-11
hsa-mir-150	ATP10A	57194	0.384565	9.07E-11
hsa-mir-150	F5	2153	0.384703	8.92E-11
hsa-mir-150	CD1E	913	0.385176	8.42E-11
hsa-mir-150	ARID5A	10865	0.385942	7.68E-11
hsa-mir-150	CASP5	838	0.386149	7.48E-11
hsa-mir-150	CXCL12	6387	0.386401	7.26E-11
hsa-mir-150	CD40	958	0.386818	6.90E-11
hsa-mir-150	STARD8	9754	0.386861	6.86E-11
hsa-mir-150	TNFRSF11A	8792	0.386868	6.86E-11
hsa-mir-150	SH2D1B	117157	0.386982	6.76E-11
hsa-mir-150	OTOA	146183	0.388066	5.92E-11
hsa-mir-150	NFKB2	4791	0.388213	5.82E-11
hsa-mir-150	ITGA5	3678	0.388248	5.79E-11
hsa-mir-150	PLA2G7	7941	0.388489	5.62E-11
hsa-mir-150	ZCCHC5	203430	0.388570	5.57E-11
hsa-mir-150	RASGRP2	10235	0.388625	5.53E-11
hsa-mir-150	P2RY6	5031	0.388696	5.48E-11
hsa-mir-150	STAP1	26228	0.388874	5.36E-11
hsa-mir-150	CCL11	6356	0.389002	5.28E-11
hsa-mir-150	FMO2	2327	0.389418	5.02E-11
hsa-mir-150	PTGER3	5733	0.390188	4.56E-11
hsa-mir-150	CYLD	1540	0.390737	4.26E-11
hsa-mir-150	NR1H3	10062	0.390769	4.24E-11
hsa-mir-150	ARPC1B	10095	0.390916	4.17E-11
hsa-mir-150	PPM1M	132160	0.391083	4.08E-11
hsa-mir-150	MVP	9961	0.391211	4.02E-11
hsa-mir-150	CEACAM4	1089	0.391285	3.98E-11
hsa-mir-150	CTSZ	1522	0.391548	3.85E-11
hsa-mir-150	VNN2	8875	0.391700	3.78E-11
hsa-mir-150	MRC1	4360	0.392663	3.36E-11
hsa-mir-150	BTN3A2	11118	0.392987	3.22E-11
hsa-mir-150	NLRP1	22861	0.393107	3.17E-11
hsa-mir-150	CTSO	1519	0.393212	3.13E-11
hsa-mir-150	CDKN2B	1030	0.393341	3.08E-11
hsa-mir-150	VCAN	1462	0.393463	3.04E-11
hsa-mir-150	GPR25	2848	0.393783	2.92E-11

hsa-mir-150	YWHAH	7533	0.393962	2.85E-11
hsa-mir-150	ITM2A	9452	0.394252	2.75E-11
hsa-mir-150	STAB1	23166	0.394576	2.64E-11
hsa-mir-150	GUCY1A3	2982	0.394719	2.59E-11
hsa-mir-150	PIK3CD	5293	0.395420	2.38E-11
hsa-mir-150	CPA3	1359	0.395653	2.31E-11
hsa-mir-150	P2RY12	64805	0.396501	2.07E-11
hsa-mir-150	CD28	940	0.396748	2.01E-11
hsa-mir-150	PIP4K2A	5305	0.396757	2.01E-11
hsa-mir-150	CTSK	1513	0.397044	1.94E-11
hsa-mir-150	NEXN	91624	0.397345	1.86E-11
hsa-mir-150	GRAP	10750	0.397434	1.84E-11
hsa-mir-150	ODF3B	440836	0.397747	1.77E-11
hsa-mir-150	PRRX1	5396	0.397764	1.77E-11
hsa-mir-150	IRAK3	11213	0.397967	1.72E-11
hsa-mir-150	SERPINB8	5271	0.398732	1.56E-11
hsa-mir-150	NFKB1	4790	0.398826	1.55E-11
hsa-mir-150	COL8A1	1295	0.398949	1.52E-11
hsa-mir-150	FLT3	2322	0.399162	1.48E-11
hsa-mir-150	ST8SIA4	7903	0.399309	1.45E-11
hsa-mir-150	KLF2	10365	0.399451	1.43E-11
hsa-mir-150	CYB5R4	51167	0.399544	1.41E-11
hsa-mir-150	HLA-DQA2	3118	0.399547	1.41E-11
hsa-mir-150	CLECL1	160365	0.399695	1.38E-11
hsa-mir-150	CYSLTR1	10800	0.400189	1.30E-11
hsa-mir-150	STOM	2040	0.400388	1.27E-11
hsa-mir-150	CD1C	911	0.401028	1.17E-11
hsa-mir-150	TMEM106A	113277	0.401375	1.12E-11
hsa-mir-150	NCR3	259197	0.401679	1.07E-11
hsa-mir-150	CSF1	1435	0.401717	1.07E-11
hsa-mir-150	PTK2B	2185	0.401984	1.03E-11
hsa-mir-150	OSTF1	26578	0.402751	9.35E-12
hsa-mir-150	LGALS2	3957	0.402853	9.23E-12
hsa-mir-150	IL18	3606	0.402978	9.08E-12
hsa-mir-150	TIMD4	91937	0.403384	8.62E-12
hsa-mir-150	FCRLA	84824	0.403399	8.60E-12
hsa-mir-150	TPSAB1	7177	0.403471	8.52E-12
hsa-mir-150	RNASE2	6036	0.403809	8.16E-12
hsa-mir-150	ARHGAP31	57514	0.403947	8.01E-12
hsa-mir-150	CCL23	6368	0.404323	7.63E-12
hsa-mir-150	TNFRSF9	3604	0.404509	7.45E-12
hsa-mir-150	RASGRP4	115727	0.404964	7.02E-12
hsa-mir-150	CYP4F22	126410	0.406200	5.98E-12
hsa-mir-150	AIM2	9447	0.406354	5.86E-12
hsa-mir-150	CEACAM21	90273	0.406452	5.79E-12
hsa-mir-150	TMEM229B	161145	0.406507	5.75E-12
hsa-mir-150	C2	717	0.406516	5.74E-12
hsa-mir-150	BHLHE22	27319	0.406534	5.73E-12
hsa-mir-150	TGFBI	7045	0.407035	5.36E-12
hsa-mir-150	PIM2	11040	0.407246	5.22E-12
hsa-mir-150	MEI1	150365	0.407489	5.05E-12
hsa-mir-150	MAF	4094	0.407968	4.75E-12
hsa-mir-150	PARVG	64098	0.407988	4.73E-12
hsa-mir-150	RARRES1	5918	0.408089	4.67E-12

hsa-mir-150	NAGA	4668	0.408919	4.19E-12
hsa-mir-150	EMR1	2015	0.409072	4.11E-12
hsa-mir-150	PHF11	51131	0.409083	4.10E-12
hsa-mir-150	FAM129A	116496	0.409416	3.92E-12
hsa-mir-150	HLA-DQB2	3120	0.409613	3.82E-12
hsa-mir-150	GPBAR1	151306	0.409976	3.65E-12
hsa-mir-150	HOPX	84525	0.410612	3.35E-12
hsa-mir-150	SIGLEC8	27181	0.411410	3.02E-12
hsa-mir-150	TAPBP	6892	0.411902	2.82E-12
hsa-mir-150	HLA-DRB5	3127	0.412252	2.70E-12
hsa-mir-150	PTGDR	5729	0.412885	2.48E-12
hsa-mir-150	MRVI1	10335	0.413516	2.28E-12
hsa-mir-150	C10orf54	64115	0.413557	2.27E-12
hsa-mir-150	TMEM200A	114801	0.413829	2.19E-12
hsa-mir-150	INHBA	3624	0.413897	2.17E-12
hsa-mir-150	DERL3	91319	0.414032	2.13E-12
hsa-mir-150	MMP25	64386	0.414414	2.02E-12
hsa-mir-150	PDCD1	5133	0.414637	1.96E-12
hsa-mir-150	TBC1D10C	374403	0.415056	1.85E-12
hsa-mir-150	IL4I1	259307	0.415427	1.76E-12
hsa-mir-150	MEF2C	4208	0.415929	1.65E-12
hsa-mir-150	HGF	3082	0.416062	1.62E-12
hsa-mir-150	CD1A	909	0.416078	1.62E-12
hsa-mir-150	CD44	960	0.416328	1.56E-12
hsa-mir-150	APOL2	23780	0.416732	1.48E-12
hsa-mir-150	GRIN3A	116443	0.417406	1.35E-12
hsa-mir-150	POU2AF1	5450	0.418324	1.19E-12
hsa-mir-150	HLA-DMB	3109	0.418408	1.18E-12
hsa-mir-150	POU2F2	5452	0.418670	1.14E-12
hsa-mir-150	IGFL2	147920	0.418815	1.12E-12
hsa-mir-150	ADAM8	101	0.418918	1.10E-12
hsa-mir-150	CLEC12A	160364	0.418953	1.10E-12
hsa-mir-150	TARP	445347	0.419029	1.08E-12
hsa-mir-150	TNFRSF4	7293	0.419159	1.07E-12
hsa-mir-150	APOBEC3D	140564	0.419416	1.03E-12
hsa-mir-150	KLRC1	3821	0.419789	9.78E-13
hsa-mir-150	LILRA2	11027	0.419835	9.71E-13
hsa-mir-150	KIR2DL4	3805	0.420025	9.47E-13
hsa-mir-150	PYCARD	29108	0.420106	9.36E-13
hsa-mir-150	BATF3	55509	0.420693	8.64E-13
hsa-mir-150	MDFIC	29969	0.421254	8.00E-13
hsa-mir-150	KCNAB2	8514	0.421737	7.49E-13
hsa-mir-150	CMKLR1	1240	0.422191	7.04E-13
hsa-mir-150	COL10A1	1300	0.422339	6.90E-13
hsa-mir-150	GPR114	221188	0.423464	5.91E-13
hsa-mir-150	OSM	5008	0.424230	5.31E-13
hsa-mir-150	PRAM1	84106	0.424289	5.27E-13
hsa-mir-150	RAP2B	5912	0.424291	5.27E-13
hsa-mir-150	IL4R	3566	0.424726	4.96E-13
hsa-mir-150	TMEM150B	284417	0.424903	4.84E-13
hsa-mir-150	CD226	10666	0.425140	4.68E-13
hsa-mir-150	KLRC3	3823	0.426079	4.11E-13
hsa-mir-150	HLA-DOA	3111	0.426136	4.08E-13
hsa-mir-150	LRRC8C	84230	0.426888	3.67E-13

hsa-mir-150	BIRC3	330	0.427088	3.57E-13
hsa-mir-150	NOD2	64127	0.427385	3.43E-13
hsa-mir-150	SAMD9L	219285	0.427969	3.16E-13
hsa-mir-150	THBS2	7058	0.428968	2.74E-13
hsa-mir-150	C1orf54	79630	0.429028	2.72E-13
hsa-mir-150	SIRPB1	10326	0.429156	2.67E-13
hsa-mir-150	CCL3	6348	0.429293	2.62E-13
hsa-mir-150	HVCN1	84329	0.429742	2.46E-13
hsa-mir-150	FN1	2335	0.429817	2.44E-13
hsa-mir-150	IL10	3586	0.430051	2.36E-13
hsa-mir-150	FUT7	2529	0.430616	2.18E-13
hsa-mir-150	CD80	941	0.431058	2.04E-13
hsa-mir-150	C3	718	0.431437	1.94E-13
hsa-mir-150	CD1B	910	0.431819	1.84E-13
hsa-mir-150	SERPINF1	5176	0.432846	1.59E-13
hsa-mir-150	PLEKHO2	80301	0.433333	1.48E-13
hsa-mir-150	MAN1A1	4121	0.433395	1.47E-13
hsa-mir-150	HLA-DOB	3112	0.433583	1.43E-13
hsa-mir-150	RENBP	5973	0.434166	1.31E-13
hsa-mir-150	SLC15A3	51296	0.434334	1.28E-13
hsa-mir-150	ZBP1	81030	0.434340	1.28E-13
hsa-mir-150	TLR6	10333	0.434910	1.18E-13
hsa-mir-150	NLRC3	197358	0.435590	1.07E-13
hsa-mir-150	IRF2	3660	0.435758	1.05E-13
hsa-mir-150	ADORA3	140	0.436481	9.42E-14
hsa-mir-150	MS4A2	2206	0.437405	8.25E-14
hsa-mir-150	LAIR2	3904	0.437628	7.99E-14
hsa-mir-150	SPIC	121599	0.438886	6.66E-14
hsa-mir-150	CD200R1	131450	0.438913	6.63E-14
hsa-mir-150	AMPD1	270	0.438951	6.59E-14
hsa-mir-150	ETS1	2113	0.439022	6.53E-14
hsa-mir-150	WARS	7453	0.439537	6.06E-14
hsa-mir-150	CLEC5A	23601	0.439708	5.91E-14
hsa-mir-150	TAP2	6891	0.439896	5.75E-14
hsa-mir-150	APOE	348	0.440241	5.47E-14
hsa-mir-150	MIAT	440823	0.440326	5.40E-14
hsa-mir-150	RARRES3	5920	0.440626	5.17E-14
hsa-mir-150	ADRB2	154	0.440686	5.12E-14
hsa-mir-150	RSU1	6251	0.440798	5.04E-14
hsa-mir-150	LILRA4	23547	0.442068	4.18E-14
hsa-mir-150	TMEM176B	28959	0.442295	4.05E-14
hsa-mir-150	SPHK1	8877	0.443731	3.28E-14
hsa-mir-150	CRTAM	56253	0.444355	2.99E-14
hsa-mir-150	TNFAIP3	7128	0.446083	2.31E-14
hsa-mir-150	JAK2	3717	0.446140	2.29E-14
hsa-mir-150	RASSF4	83937	0.446577	2.15E-14
hsa-mir-150	SMPDL3A	10924	0.446657	2.12E-14
hsa-mir-150	INPP4B	8821	0.447256	1.94E-14
hsa-mir-150	CSF3R	1441	0.447466	1.88E-14
hsa-mir-150	TRIM21	6737	0.447505	1.87E-14
hsa-mir-150	ITGB7	3695	0.447512	1.87E-14
hsa-mir-150	C4orf7	260436	0.447665	1.83E-14
hsa-mir-150	STK10	6793	0.448603	1.59E-14
hsa-mir-150	NNMT	4837	0.449321	1.43E-14

hsa-mir-150	TIGIT	201633	0.449694	1.35E-14
hsa-mir-150	PTGER4	5734	0.449968	1.29E-14
hsa-mir-150	MFSD1	64747	0.450408	1.21E-14
hsa-mir-150	MMP9	4318	0.450700	1.16E-14
hsa-mir-150	VAV1	7409	0.450776	1.15E-14
hsa-mir-150	C12orf59	120939	0.451089	1.09E-14
hsa-mir-150	UBE2L6	9246	0.451204	1.07E-14
hsa-mir-150	PLCB2	5330	0.451452	1.03E-14
hsa-mir-150	PLA2G4C	8605	0.451455	1.03E-14
hsa-mir-150	IFI35	3430	0.452425	8.92E-15
hsa-mir-150	LACTB	114294	0.452478	8.85E-15
hsa-mir-150	STAC3	246329	0.452785	8.45E-15
hsa-mir-150	MICAL1	64780	0.452824	8.40E-15
hsa-mir-150	PLAUR	5329	0.453692	7.36E-15
hsa-mir-150	CIITA	4261	0.453823	7.21E-15
hsa-mir-150	NAPSB	256236	0.454037	6.98E-15
hsa-mir-150	TLR1	7096	0.454872	6.15E-15
hsa-mir-150	ATP8B4	79895	0.455347	5.71E-15
hsa-mir-150	LGALS9	3965	0.455754	5.37E-15
hsa-mir-150	TMEM154	201799	0.456231	4.99E-15
hsa-mir-150	A2M	2	0.456569	4.74E-15
hsa-mir-150	FAM113B	91523	0.457053	4.40E-15
hsa-mir-150	NLRC4	58484	0.458563	3.48E-15
hsa-mir-150	LAT	27040	0.458574	3.48E-15
hsa-mir-150	IFNG	3458	0.459077	3.22E-15
hsa-mir-150	C5AR1	728	0.459781	2.88E-15
hsa-mir-150	BATF2	116071	0.459970	2.80E-15
hsa-mir-150	TPP1	1200	0.459972	2.80E-15
hsa-mir-150	FCGR2C	9103	0.460026	2.78E-15
hsa-mir-150	BCL11B	64919	0.460883	2.43E-15
hsa-mir-150	IL18RAP	8807	0.461602	2.17E-15
hsa-mir-150	CCDC109B	55013	0.461926	2.06E-15
hsa-mir-150	TREM2	54209	0.461965	2.05E-15
hsa-mir-150	SQRDL	58472	0.462098	2.01E-15
hsa-mir-150	ARL11	115761	0.462226	1.97E-15
hsa-mir-150	GPR34	2857	0.462756	1.81E-15
hsa-mir-150	TRAF1	7185	0.462848	1.79E-15
hsa-mir-150	PSMB8	5696	0.463534	1.60E-15
hsa-mir-150	LAX1	54900	0.463586	1.59E-15
hsa-mir-150	RASGRP3	25780	0.464455	1.39E-15
hsa-mir-150	PIK3R6	146850	0.464501	1.38E-15
hsa-mir-150	TIFAB	497189	0.465237	1.23E-15
hsa-mir-150	TNFRSF17	608	0.465426	1.19E-15
hsa-mir-150	ZNF831	128611	0.465883	1.11E-15
hsa-mir-150	TNFSF12	8742	0.466061	1.08E-15
hsa-mir-150	PDE4B	5142	0.467065	9.17E-16
hsa-mir-150	DAPP1	27071	0.467735	8.24E-16
hsa-mir-150	C13orf31	144811	0.467750	8.22E-16
hsa-mir-150	APOL1	8542	0.469649	6.07E-16
hsa-mir-150	ICAM1	3383	0.470424	5.35E-16
hsa-mir-150	IL32	9235	0.470667	5.15E-16
hsa-mir-150	RAB31	11031	0.471719	4.35E-16
hsa-mir-150	PARP15	165631	0.472267	3.98E-16
hsa-mir-150	GPNMB	10457	0.472864	3.61E-16

hsa-mir-150	HLA-C	3107	0.473405	3.31E-16
hsa-mir-150	HMOX1	3162	0.473658	3.17E-16
hsa-mir-150	FASLG	356	0.473710	3.15E-16
hsa-mir-150	CARD16	114769	0.474920	2.58E-16
hsa-mir-150	GNB4	59345	0.475085	2.51E-16
hsa-mir-150	SIGLEC1	6614	0.475883	2.21E-16
hsa-mir-150	GNG2	54331	0.476063	2.14E-16
hsa-mir-150	HLA-DQB1	3119	0.477029	1.83E-16
hsa-mir-150	TCL1A	8115	0.477494	1.69E-16
hsa-mir-150	SOD2	6648	0.477643	1.65E-16
hsa-mir-150	LGALS1	3956	0.477956	1.57E-16
hsa-mir-150	MSC	9242	0.478065	1.54E-16
hsa-mir-150	FGF7	2252	0.478160	1.52E-16
hsa-mir-150	ADAM19	8728	0.478358	1.47E-16
hsa-mir-150	SDS	10993	0.478716	1.38E-16
hsa-mir-150	FCRL2	79368	0.479236	1.27E-16
hsa-mir-150	GAPT	202309	0.479536	1.21E-16
hsa-mir-150	CCL22	6367	0.480301	1.06E-16
hsa-mir-150	ADORA2A	135	0.480428	1.04E-16
hsa-mir-150	MS4A14	84689	0.481037	9.42E-17
hsa-mir-150	LRRK2	120892	0.482899	6.90E-17
hsa-mir-150	EPSTI1	94240	0.483472	6.27E-17
hsa-mir-150	APOBEC3G	60489	0.483609	6.12E-17
hsa-mir-150	HCP5	10866	0.483698	6.03E-17
hsa-mir-150	SH2B3	10019	0.484337	5.42E-17
hsa-mir-150	GAL3ST4	79690	0.484501	5.27E-17
hsa-mir-150	XCR1	2829	0.484960	4.88E-17
hsa-mir-150	C1R	715	0.485558	4.41E-17
hsa-mir-150	SNX10	29887	0.486045	4.06E-17
hsa-mir-150	RHOG	391	0.486235	3.93E-17
hsa-mir-150	SLC31A2	1318	0.486278	3.90E-17
hsa-mir-150	FPR1	2357	0.486304	3.89E-17
hsa-mir-150	CD8B	926	0.486606	3.69E-17
hsa-mir-150	APOC2	344	0.486753	3.60E-17
hsa-mir-150	HCG26	352961	0.486777	3.59E-17
hsa-mir-150	IDO1	3620	0.487075	3.41E-17
hsa-mir-150	TNFAIP6	7130	0.487089	3.40E-17
hsa-mir-150	C5orf20	140947	0.487632	3.10E-17
hsa-mir-150	CTLA4	1493	0.487812	3.01E-17
hsa-mir-150	SLCO2B1	11309	0.488347	2.75E-17
hsa-mir-150	LOC100188949	100188949	0.488440	2.70E-17
hsa-mir-150	NCF1B	654816	0.488655	2.60E-17
hsa-mir-150	KYNU	8942	0.489163	2.39E-17
hsa-mir-150	MSN	4478	0.489628	2.21E-17
hsa-mir-150	SLC37A2	219855	0.489793	2.14E-17
hsa-mir-150	CD1D	912	0.490445	1.92E-17
hsa-mir-150	TMEM156	80008	0.490982	1.75E-17
hsa-mir-150	SEPTIN1	1731	0.491318	1.65E-17
hsa-mir-150	CCL2	6347	0.491416	1.62E-17
hsa-mir-150	NLRP3	114548	0.491923	1.49E-17
hsa-mir-150	IKZF3	22806	0.491939	1.48E-17
hsa-mir-150	ZC3H12D	340152	0.492272	1.40E-17
hsa-mir-150	SIGLEC5	8778	0.492366	1.38E-17
hsa-mir-150	MPP1	4354	0.492665	1.31E-17

hsa-mir-150	IL12RB1	3594	0.492772	1.28E-17
hsa-mir-150	TBX21	30009	0.493312	1.17E-17
hsa-mir-150	ATP2A3	489	0.493550	1.12E-17
hsa-mir-150	GPSM3	63940	0.493961	1.04E-17
hsa-mir-150	GZMM	3004	0.494029	1.03E-17
hsa-mir-150	ELL2	22936	0.494585	9.37E-18
hsa-mir-150	FAP	2191	0.494622	9.31E-18
hsa-mir-150	VSIG4	11326	0.494787	9.04E-18
hsa-mir-150	CD70	970	0.494874	8.91E-18
hsa-mir-150	CCR4	1233	0.495295	8.28E-18
hsa-mir-150	GGT5	2687	0.495940	7.39E-18
hsa-mir-150	CLEC9A	283420	0.496421	6.80E-18
hsa-mir-150	CCL8	6355	0.498570	4.66E-18
hsa-mir-150	HLA-H	3136	0.498975	4.34E-18
hsa-mir-150	ZNF683	257101	0.499392	4.03E-18
hsa-mir-150	TNIP3	79931	0.499703	3.81E-18
hsa-mir-150	IL7	3574	0.499974	3.63E-18
hsa-mir-150	HLA-DMA	3108	0.500857	3.11E-18
hsa-mir-150	PILRA	29992	0.501318	2.86E-18
hsa-mir-150	FCRL1	115350	0.501352	2.84E-18
hsa-mir-150	MARCHF1	55016	0.501425	2.81E-18
hsa-mir-150	SAMD3	154075	0.501882	2.59E-18
hsa-mir-150	LCP1	3936	0.503122	2.07E-18
hsa-mir-150	IRF1	3659	0.503374	1.98E-18
hsa-mir-150	LHFPL2	10184	0.503943	1.79E-18
hsa-mir-150	PSMB10	5699	0.504673	1.57E-18
hsa-mir-150	PIK3AP1	118788	0.504803	1.53E-18
hsa-mir-150	EOMES	8320	0.505217	1.42E-18
hsa-mir-150	DARC	2532	0.505937	1.25E-18
hsa-mir-150	HCLS1	3059	0.506042	1.23E-18
hsa-mir-150	TGFB1	7040	0.506083	1.22E-18
hsa-mir-150	PTAFR	5724	0.506435	1.14E-18
hsa-mir-150	TMIGD2	126259	0.507655	9.15E-19
hsa-mir-150	OPTN	10133	0.507761	8.98E-19
hsa-mir-150	CXCL13	10563	0.508179	8.32E-19
hsa-mir-150	PRKCB	5579	0.508449	7.92E-19
hsa-mir-150	C19orf35	374872	0.509089	7.05E-19
hsa-mir-150	HSPA7	3311	0.509823	6.16E-19
hsa-mir-150	GLRX	2745	0.510254	5.70E-19
hsa-mir-150	TMEM149	79713	0.510314	5.63E-19
hsa-mir-150	FGD2	221472	0.510732	5.22E-19
hsa-mir-150	CD79A	973	0.511175	4.81E-19
hsa-mir-150	GZMB	3002	0.511564	4.48E-19
hsa-mir-150	XCL2	6846	0.511604	4.44E-19
hsa-mir-150	IL15	3600	0.512910	3.49E-19
hsa-mir-150	UBASH3A	53347	0.513507	3.13E-19
hsa-mir-150	TLR8	51311	0.513564	3.09E-19
hsa-mir-150	PLA2G2D	26279	0.514891	2.42E-19
hsa-mir-150	PLAU	5328	0.515277	2.25E-19
hsa-mir-150	P2RX7	5027	0.515633	2.11E-19
hsa-mir-150	GNLY	10578	0.515840	2.03E-19
hsa-mir-150	CLEC4E	26253	0.516539	1.78E-19
hsa-mir-150	GPRIN3	285513	0.516576	1.77E-19
hsa-mir-150	CLEC10A	10462	0.517333	1.53E-19

hsa-mir-150	GPR18	2841	0.517352	1.53E-19
hsa-mir-150	RGS1	5996	0.517680	1.44E-19
hsa-mir-150	PSTPIP1	9051	0.518848	1.15E-19
hsa-mir-150	MS4A7	58475	0.519470	1.02E-19
hsa-mir-150	IL18BP	10068	0.520244	8.85E-20
hsa-mir-150	CD3G	917	0.520278	8.79E-20
hsa-mir-150	LTB	4050	0.521222	7.35E-20
hsa-mir-150	HLA-DRB1	3123	0.522034	6.30E-20
hsa-mir-150	VCAM1	7412	0.522770	5.47E-20
hsa-mir-150	DOCK8	81704	0.524742	3.75E-20
hsa-mir-150	NLRC5	84166	0.526073	2.90E-20
hsa-mir-150	P2RY10	27334	0.527431	2.23E-20
hsa-mir-150	IL15RA	3601	0.527681	2.12E-20
hsa-mir-150	CD40LG	959	0.528166	1.93E-20
hsa-mir-150	SLC2A5	6518	0.528472	1.82E-20
hsa-mir-150	KIAA0748	9840	0.529557	1.47E-20
hsa-mir-150	DRAM1	55332	0.529672	1.44E-20
hsa-mir-150	PRDM1	639	0.530330	1.27E-20
hsa-mir-150	GNA15	2769	0.530344	1.26E-20
hsa-mir-150	CD79B	974	0.530483	1.23E-20
hsa-mir-150	CSF1R	1436	0.531261	1.06E-20
hsa-mir-150	LOC606724	606724	0.531769	9.55E-21
hsa-mir-150	ITGA4	3676	0.532598	8.11E-21
hsa-mir-150	CD163	9332	0.533022	7.46E-21
hsa-mir-150	ADAMDEC1	27299	0.533793	6.41E-21
hsa-mir-150	ETV7	51513	0.534559	5.50E-21
hsa-mir-150	RHOH	399	0.534642	5.41E-21
hsa-mir-150	FAM26F	441168	0.535065	4.98E-21
hsa-mir-150	IL21R	50615	0.535482	4.58E-21
hsa-mir-150	LILRA5	353514	0.536765	3.54E-21
hsa-mir-150	CXCR2P1	3580	0.536902	3.45E-21
hsa-mir-150	DOCK10	55619	0.536914	3.44E-21
hsa-mir-150	SULF1	23213	0.537383	3.13E-21
hsa-mir-150	ADAP2	55803	0.537742	2.91E-21
hsa-mir-150	DPEP2	64174	0.537843	2.86E-21
hsa-mir-150	SLC7A7	9056	0.537958	2.79E-21
hsa-mir-150	FCRL3	115352	0.538393	2.56E-21
hsa-mir-150	ALOX5AP	241	0.538804	2.35E-21
hsa-mir-150	KIAA1949	170954	0.538888	2.31E-21
hsa-mir-150	KLHL6	89857	0.539771	1.94E-21
hsa-mir-150	HLA-A	3105	0.540318	1.73E-21
hsa-mir-150	GZMH	2999	0.540459	1.68E-21
hsa-mir-150	NPL	80896	0.540461	1.68E-21
hsa-mir-150	LILRA6	79168	0.540610	1.63E-21
hsa-mir-150	TAP1	6890	0.541883	1.26E-21
hsa-mir-150	ITK	3702	0.541911	1.25E-21
hsa-mir-150	SIGLEC7	27036	0.542126	1.20E-21
hsa-mir-150	EMR2	30817	0.542204	1.18E-21
hsa-mir-150	CD74	972	0.542432	1.13E-21
hsa-mir-150	CCR2	729230	0.542607	1.09E-21
hsa-mir-150	STAT4	6775	0.543106	9.83E-22
hsa-mir-150	INPP5D	3635	0.543204	9.64E-22
hsa-mir-150	ZAP70	7535	0.544768	6.99E-22
hsa-mir-150	MS4A1	931	0.545156	6.46E-22

hsa-mir-150	KLRB1	3820	0.546986	4.43E-22
hsa-mir-150	C17orf60	284021	0.548135	3.49E-22
hsa-mir-150	CFD	1675	0.548316	3.36E-22
hsa-mir-150	CD6	923	0.548516	3.22E-22
hsa-mir-150	GBP1	2633	0.548999	2.91E-22
hsa-mir-150	IFFO1	25900	0.549032	2.89E-22
hsa-mir-150	SIGLEC9	27180	0.550229	2.25E-22
hsa-mir-150	SIRPG	55423	0.551127	1.87E-22
hsa-mir-150	LYL1	4066	0.552029	1.54E-22
hsa-mir-150	APOC1	341	0.552086	1.53E-22
hsa-mir-150	FCRL5	83416	0.552225	1.48E-22
hsa-mir-150	GIMAP2	26157	0.552393	1.43E-22
hsa-mir-150	MSR1	4481	0.553017	1.25E-22
hsa-mir-150	TNFRSF13B	23495	0.553392	1.16E-22
hsa-mir-150	LOC400759	400759	0.554011	1.02E-22
hsa-mir-150	VAMP5	10791	0.554495	9.17E-23
hsa-mir-150	TBXAS1	6916	0.554559	9.04E-23
hsa-mir-150	PYHIN1	149628	0.555536	7.35E-23
hsa-mir-150	CD300A	11314	0.556250	6.31E-23
hsa-mir-150	CD69	969	0.556522	5.95E-23
hsa-mir-150	CCL19	6363	0.556648	5.79E-23
hsa-mir-150	CD14	929	0.557381	4.95E-23
hsa-mir-150	OSCAR	126014	0.558410	3.97E-23
hsa-mir-150	SLAMF1	6504	0.558917	3.56E-23
hsa-mir-150	MCTP1	79772	0.559197	3.35E-23
hsa-mir-150	TLR2	7097	0.559656	3.03E-23
hsa-mir-150	ALOX5	240	0.559867	2.90E-23
hsa-mir-150	ZEB2	9839	0.560008	2.81E-23
hsa-mir-150	CLEC2B	9976	0.560009	2.81E-23
hsa-mir-150	CD300C	10871	0.560276	2.65E-23
hsa-mir-150	CXCL11	6373	0.560551	2.50E-23
hsa-mir-150	TLR4	7099	0.561335	2.11E-23
hsa-mir-150	PIK3R5	23533	0.561398	2.08E-23
hsa-mir-150	FCN1	2219	0.561441	2.06E-23
hsa-mir-150	ITGAM	3684	0.562021	1.82E-23
hsa-mir-150	SLC9A9	285195	0.562278	1.72E-23
hsa-mir-150	MGC29506	51237	0.562437	1.66E-23
hsa-mir-150	GPR84	53831	0.562588	1.61E-23
hsa-mir-150	EPB41L3	23136	0.563192	1.41E-23
hsa-mir-150	B2M	567	0.563284	1.38E-23
hsa-mir-150	CD244	51744	0.563426	1.34E-23
hsa-mir-150	TNFRSF1B	7133	0.563765	1.24E-23
hsa-mir-150	CD72	971	0.565103	9.25E-24
hsa-mir-150	IFI30	10437	0.565341	8.78E-24
hsa-mir-150	P2RY13	53829	0.566520	6.77E-24
hsa-mir-150	HLA-DRA	3122	0.567221	5.79E-24
hsa-mir-150	TRIM22	10346	0.567325	5.66E-24
hsa-mir-150	PIK3CG	5294	0.567489	5.46E-24
hsa-mir-150	PTGIR	5739	0.568278	4.58E-24
hsa-mir-150	FLI1	2313	0.568443	4.42E-24
hsa-mir-150	LY9	4063	0.569376	3.59E-24
hsa-mir-150	FAM78A	286336	0.569787	3.27E-24
hsa-mir-150	C4orf32	132720	0.570371	2.87E-24
hsa-mir-150	IRF8	3394	0.570686	2.68E-24

hsa-mir-150	FMNL1	752	0.571460	2.25E-24
hsa-mir-150	LRRC25	126364	0.571709	2.13E-24
hsa-mir-150	ARHGEF6	9459	0.571906	2.03E-24
hsa-mir-150	GRAP2	9402	0.572810	1.66E-24
hsa-mir-150	HLA-DQA1	3117	0.572826	1.65E-24
hsa-mir-150	CORO1A	11151	0.572929	1.62E-24
hsa-mir-150	TLR7	51284	0.573333	1.47E-24
hsa-mir-150	HLA-E	3133	0.574578	1.11E-24
hsa-mir-150	GBP4	115361	0.574829	1.05E-24
hsa-mir-150	APOB48R	55911	0.575436	9.14E-25
hsa-mir-150	CASP1	834	0.576169	7.73E-25
hsa-mir-150	KLRK1	22914	0.576856	6.61E-25
hsa-mir-150	BCL2A1	597	0.577563	5.62E-25
hsa-mir-150	HLA-B	3106	0.578411	4.62E-25
hsa-mir-150	LTA	4049	0.578433	4.60E-25
hsa-mir-150	CD84	8832	0.579034	4.00E-25
hsa-mir-150	TRAT1	50852	0.579315	3.75E-25
hsa-mir-150	UBD	10537	0.579572	3.54E-25
hsa-mir-150	ICOS	29851	0.581985	2.02E-25
hsa-mir-150	CSF2RA	1438	0.582171	1.93E-25
hsa-mir-150	MS4A4A	51338	0.582410	1.83E-25
hsa-mir-150	HK3	3101	0.582506	1.79E-25
hsa-mir-150	GPR174	84636	0.582922	1.62E-25
hsa-mir-150	LAG3	3902	0.583966	1.27E-25
hsa-mir-150	KCNA3	3738	0.585421	9.02E-26
hsa-mir-150	FCGR2B	2213	0.585546	8.76E-26
hsa-mir-150	C17orf87	388325	0.585600	8.65E-26
hsa-mir-150	APOL6	80830	0.586465	7.05E-26
hsa-mir-150	CLIC2	1193	0.586808	6.50E-26
hsa-mir-150	KLHDC7B	113730	0.586870	6.41E-26
hsa-mir-150	TMC8	147138	0.587237	5.87E-26
hsa-mir-150	DOK3	79930	0.587309	5.77E-26
hsa-mir-150	CD33	945	0.587598	5.39E-26
hsa-mir-150	THEMIS	387357	0.587654	5.32E-26
hsa-mir-150	CXCL10	3627	0.587762	5.18E-26
hsa-mir-150	AGAP2	116986	0.588800	4.05E-26
hsa-mir-150	C1S	716	0.589035	3.83E-26
hsa-mir-150	RNASE6	6039	0.589439	3.47E-26
hsa-mir-150	WDFY4	57705	0.589780	3.20E-26
hsa-mir-150	EMP3	2014	0.590326	2.81E-26
hsa-mir-150	MPEG1	219972	0.590469	2.71E-26
hsa-mir-150	IL7R	3575	0.591691	2.02E-26
hsa-mir-150	NFAM1	150372	0.592729	1.57E-26
hsa-mir-150	RGS18	64407	0.592895	1.51E-26
hsa-mir-150	SIRPB2	284759	0.593389	1.34E-26
hsa-mir-150	OLR1	4973	0.593415	1.33E-26
hsa-mir-150	PSMB9	5698	0.593963	1.17E-26
hsa-mir-150	EBI3	10148	0.594600	1.00E-26
hsa-mir-150	FAIM3	9214	0.594722	9.70E-27
hsa-mir-150	FCGR2A	2212	0.595371	8.28E-27
hsa-mir-150	C3AR1	719	0.597326	5.13E-27
hsa-mir-150	GPR171	29909	0.597939	4.41E-27
hsa-mir-150	HLA-F	3134	0.598155	4.18E-27
hsa-mir-150	SH2D1A	4068	0.598482	3.86E-27

hsa-mir-150	GLIPR1	11010	0.599019	3.38E-27
hsa-mir-150	GPR68	8111	0.599481	3.01E-27
hsa-mir-150	HMHA1	23526	0.601124	2.00E-27
hsa-mir-150	ARHGAP30	257106	0.601986	1.62E-27
hsa-mir-150	LAT2	7462	0.602519	1.41E-27
hsa-mir-150	CCL4	6351	0.604054	9.62E-28
hsa-mir-150	APOL3	80833	0.605062	7.46E-28
hsa-mir-150	FLT3LG	2323	0.606655	4.98E-28
hsa-mir-150	LY86	9450	0.606707	4.92E-28
hsa-mir-150	GIMAP8	155038	0.608270	3.30E-28
hsa-mir-150	HLA-DPA1	3113	0.608304	3.27E-28
hsa-mir-150	DPYD	1806	0.609055	2.70E-28
hsa-mir-150	ITGB2	3689	0.609738	2.27E-28
hsa-mir-150	CCR1	1230	0.610673	1.78E-28
hsa-mir-150	BATF	10538	0.611878	1.31E-28
hsa-mir-150	FCGR1A	2209	0.612117	1.23E-28
hsa-mir-150	CYTH4	27128	0.612135	1.22E-28
hsa-mir-150	FCGR1B	2210	0.612879	1.01E-28
hsa-mir-150	MLKL	197259	0.612993	9.78E-29
hsa-mir-150	HLA-DPB1	3115	0.613233	9.18E-29
hsa-mir-150	FCGR3A	2214	0.613277	9.08E-29
hsa-mir-150	TRPV2	51393	0.614344	6.87E-29
hsa-mir-150	ITGAX	3687	0.614800	6.10E-29
hsa-mir-150	AOAH	313	0.614824	6.06E-29
hsa-mir-150	ANKRD22	118932	0.616188	4.24E-29
hsa-mir-150	CLEC4A	50856	0.616716	3.69E-29
hsa-mir-150	PTPN7	5778	0.616886	3.53E-29
hsa-mir-150	SIGLEC10	89790	0.617103	3.33E-29
hsa-mir-150	C1QC	714	0.618513	2.30E-29
hsa-mir-150	NCF2	4688	0.618548	2.27E-29
hsa-mir-150	IRF4	3662	0.618814	2.12E-29
hsa-mir-150	FGR	2268	0.620181	1.47E-29
hsa-mir-150	CD180	4064	0.621688	9.85E-30
hsa-mir-150	CLEC7A	64581	0.622033	8.98E-30
hsa-mir-150	GPR183	1880	0.623099	6.74E-30
hsa-mir-150	CD38	952	0.623321	6.35E-30
hsa-mir-150	FPR3	2359	0.623526	6.01E-30
hsa-mir-150	WIPF1	7456	0.623579	5.92E-30
hsa-mir-150	GZMK	3003	0.623667	5.78E-30
hsa-mir-150	GBP2	2634	0.623871	5.47E-30
hsa-mir-150	CD68	968	0.624224	4.98E-30
hsa-mir-150	CXorf21	80231	0.624539	4.57E-30
hsa-mir-150	NCF1C	654817	0.624956	4.08E-30
hsa-mir-150	GPR65	8477	0.626017	3.06E-30
hsa-mir-150	GAB3	139716	0.627800	1.88E-30
hsa-mir-150	CTSS	1520	0.628212	1.68E-30
hsa-mir-150	GNGT2	2793	0.628275	1.65E-30
hsa-mir-150	CD3D	915	0.628277	1.65E-30
hsa-mir-150	CD4	920	0.628386	1.60E-30
hsa-mir-150	CYBB	1536	0.628462	1.57E-30
hsa-mir-150	TNFAIP8L2	79626	0.629337	1.23E-30
hsa-mir-150	C1QB	713	0.632129	5.68E-31
hsa-mir-150	RCSD1	92241	0.633420	3.96E-31
hsa-mir-150	LPXN	9404	0.633704	3.66E-31

hsa-mir-150	ARHGDIB	397	0.635003	2.54E-31
hsa-mir-150	SLA	6503	0.635444	2.24E-31
hsa-mir-150	NCKAP1L	3071	0.637259	1.34E-31
hsa-mir-150	SRGN	5552	0.637539	1.24E-31
hsa-mir-150	MS4A6A	64231	0.637658	1.20E-31
hsa-mir-150	LAPTM5	7805	0.638181	1.03E-31
hsa-mir-150	HCK	3055	0.638438	9.61E-32
hsa-mir-150	NCF4	4689	0.639690	6.72E-32
hsa-mir-150	SLAMF7	57823	0.641962	3.50E-32
hsa-mir-150	TYROBP	7305	0.643355	2.34E-32
hsa-mir-150	IL2RA	3559	0.644274	1.79E-32
hsa-mir-150	CCRL2	9034	0.644788	1.54E-32
hsa-mir-150	IL16	3603	0.645453	1.27E-32
hsa-mir-150	CD52	1043	0.645484	1.26E-32
hsa-mir-150	LOC96610	96610	0.645831	1.14E-32
hsa-mir-150	SELPLG	6404	0.646470	9.41E-33
hsa-mir-150	C1orf162	128346	0.647657	6.64E-33
hsa-mir-150	C1QA	712	0.647910	6.16E-33
hsa-mir-150	MYO1F	4542	0.648592	5.03E-33
hsa-mir-150	PECAM1	5175	0.651601	2.06E-33
hsa-mir-150	GIMAP6	474344	0.652496	1.57E-33
hsa-mir-150	HCST	10870	0.653064	1.33E-33
hsa-mir-150	LY96	23643	0.655227	6.89E-34
hsa-mir-150	DOCK2	1794	0.655446	6.45E-34
hsa-mir-150	LILRB1	10859	0.655657	6.05E-34
hsa-mir-150	MNDA	4332	0.656052	5.36E-34
hsa-mir-150	BTK	695	0.656515	4.66E-34
hsa-mir-150	AIF1	199	0.656863	4.19E-34
hsa-mir-150	LSP1	4046	0.657494	3.45E-34
hsa-mir-150	IGSF6	10261	0.657719	3.22E-34
hsa-mir-150	SP140	11262	0.659778	1.71E-34
hsa-mir-150	APBB1IP	54518	0.660306	1.45E-34
hsa-mir-150	FERMT3	83706	0.660778	1.26E-34
hsa-mir-150	IKZF1	10320	0.661102	1.14E-34
hsa-mir-150	GBP5	115362	0.661273	1.08E-34
hsa-mir-150	CD300LF	146722	0.661697	9.44E-35
hsa-mir-150	LILRB4	11006	0.661960	8.70E-35
hsa-mir-150	NCF1	653361	0.662458	7.45E-35
hsa-mir-150	HAVCR2	84868	0.663759	4.96E-35
hsa-mir-150	LAIR1	3903	0.664039	4.54E-35
hsa-mir-150	MYO1G	64005	0.664860	3.51E-35
hsa-mir-150	TFEC	22797	0.666105	2.37E-35
hsa-mir-150	FCER1G	2207	0.666122	2.36E-35
hsa-mir-150	SPI1	6688	0.666663	1.98E-35
hsa-mir-150	CD86	942	0.666845	1.87E-35
hsa-mir-150	LYZ	4069	0.667182	1.68E-35
hsa-mir-150	CD7	924	0.667900	1.34E-35
hsa-mir-150	RAC2	5880	0.668634	1.06E-35
hsa-mir-150	LCK	3932	0.668982	9.48E-36
hsa-mir-150	GIMAP1	170575	0.670635	5.58E-36
hsa-mir-150	FOXP3	50943	0.670898	5.13E-36
hsa-mir-150	EVI2A	2123	0.670937	5.06E-36
hsa-mir-150	ABI3	51225	0.672950	2.64E-36
hsa-mir-150	TYMP	1890	0.674877	1.41E-36

hsa-mir-150	LST1	7940	0.675257	1.24E-36
hsa-mir-150	IL10RA	3587	0.675399	1.19E-36
hsa-mir-150	GFI1	2672	0.676111	9.39E-37
hsa-mir-150	PLEK	5341	0.676719	7.68E-37
hsa-mir-150	SAMSN1	64092	0.677479	5.98E-37
hsa-mir-150	LILRB2	10288	0.679870	2.70E-37
hsa-mir-150	CSF2RB	1439	0.680476	2.21E-37
hsa-mir-150	GMFG	9535	0.681100	1.79E-37
hsa-mir-150	FGL2	10875	0.682755	1.02E-37
hsa-mir-150	SELL	6402	0.684282	6.11E-38
hsa-mir-150	GZMA	3001	0.686564	2.80E-38
hsa-mir-150	CD53	963	0.687310	2.17E-38
hsa-mir-150	CTSW	1521	0.688603	1.39E-38
hsa-mir-150	DOK2	9046	0.688919	1.24E-38
hsa-mir-150	LOC100233209	100233209	0.690661	6.79E-39
hsa-mir-150	SLAMF6	114836	0.691170	5.69E-39
hsa-mir-150	WAS	7454	0.691444	5.17E-39
hsa-mir-150	ARHGAP9	64333	0.691680	4.76E-39
hsa-mir-150	SPN	6693	0.693839	2.23E-39
hsa-mir-150	ADAM6	8755	0.694293	1.90E-39
hsa-mir-150	PDCD1LG2	80380	0.697165	6.82E-40
hsa-mir-150	P2RY8	286530	0.697297	6.51E-40
hsa-mir-150	TNFSF13B	10673	0.697591	5.85E-40
hsa-mir-150	TAGAP	117289	0.701257	1.56E-40
hsa-mir-150	IGJ	3512	0.708471	1.08E-41
hsa-mir-150	SASH3	54440	0.711384	3.59E-42
hsa-mir-150	AMICA1	120425	0.712138	2.69E-42
hsa-mir-150	SNX20	124460	0.713342	1.70E-42
hsa-mir-150	RASAL3	64926	0.713815	1.42E-42
hsa-mir-150	LCP2	3937	0.717208	3.81E-43
hsa-mir-150	SLAMF8	56833	0.717765	3.07E-43
hsa-mir-150	BIN2	51411	0.719933	1.31E-43
hsa-mir-150	C16orf54	283897	0.720815	9.25E-44
hsa-mir-150	EVI2B	2124	0.722632	4.49E-44
hsa-mir-150	GIMAP4	55303	0.723925	2.68E-44
hsa-mir-150	ARHGAP15	55843	0.724095	2.50E-44
hsa-mir-150	CXCL9	4283	0.724864	1.84E-44
hsa-mir-150	CD2	914	0.726965	7.84E-45
hsa-mir-150	GVIN1	387751	0.727118	7.36E-45
hsa-mir-150	PTPN22	26191	0.728368	4.42E-45
hsa-mir-150	GIMAP5	55340	0.731365	1.29E-45
hsa-mir-150	CD37	951	0.732441	8.21E-46
hsa-mir-150	CYTIP	9595	0.734625	3.29E-46
hsa-mir-150	CD8A	925	0.735451	2.32E-46
hsa-mir-150	CST7	8530	0.736782	1.32E-46
hsa-mir-150	PRF1	5551	0.738247	7.07E-47
hsa-mir-150	CD27	939	0.746595	1.85E-48
hsa-mir-150	PTPRC	5788	0.747144	1.45E-48
hsa-mir-150	CCR7	1236	0.747343	1.33E-48
hsa-mir-150	ITGAL	3683	0.747963	1.00E-48
hsa-mir-150	TRAF3IP3	80342	0.749374	5.33E-49
hsa-mir-150	STX11	8676	0.749887	4.23E-49
hsa-mir-150	FYB	2533	0.752367	1.37E-49
hsa-mir-150	CD5	921	0.755378	3.42E-50

hsa-mir-150	IL2RB	3560	0.755453	3.30E-50
hsa-mir-150	CD48	962	0.756529	2.00E-50
hsa-mir-150	PTPRCAP	5790	0.759611	4.70E-51
hsa-mir-150	GIMAP7	168537	0.768821	5.42E-53
hsa-mir-150	NKG7	4818	0.769208	4.48E-53
hsa-mir-150	SLA2	84174	0.771694	1.29E-53
hsa-mir-150	CCR5	1234	0.772460	8.78E-54
hsa-mir-150	SIT1	27240	0.772769	7.51E-54
hsa-mir-150	S1PR4	8698	0.775157	2.23E-54
hsa-mir-150	CD247	919	0.775288	2.08E-54
hsa-mir-150	CXCR6	10663	0.781535	8.05E-56
hsa-mir-150	ACAP1	9744	0.786073	7.08E-57
hsa-mir-150	IL2RG	3561	0.786548	5.47E-57
hsa-mir-150	CCL5	6352	0.788929	1.49E-57
hsa-mir-150	CD96	10225	0.791490	3.59E-58
hsa-mir-150	CXCR3	2833	0.804969	1.44E-61
hsa-mir-150	CD3E	916	0.841490	2.77E-72

Table S5. The putative miR-150-5p and miR-150-3p binding sites in the CDS or 3'UTR region of five predicted targets by miRWalk.

Predicted pairing of target region (top) and miRNA (bottom)

```
Position 3868-3901 of IGF1R CDS      5'      TCGCTGTCCTGTTGATCGTGGAGGGTTGGTGA     3'
                                                                   ||||||||
miR-150-5p                           3'                  GUGACCAUGUUCCCAACCCUCU    5'

Position 4056-4078 of IGF1R CDS      5'      CTTGGGCAGGGGTCGTTTGGGAT              3'
                                                             ||||||
miR-150-5p                           3'        GUGACCAUGUUCCCAACCCUCU             5'

Position 8406-8428 of IGF1R 3'UTR    5'      AGTCACCCAGCCTGTGCGCCAGA              3'
                                              ||| ||||| |  ||  ||||
miR-150-3p                           3'     GACAGGGGGUCCG GACAUGGUC               5'

Position 9663-9892 of IGF1R 3'UTR    5'       TGCTCGGTGCACATTGGGGTGCTTTGGGAT          3'
                                                      |||           ||||||
miR-150-5p                           3'               GUGACCAUGUUCCCAACCCUCU      5'

Position 895-916 of IRS1 CDS         5'      CGTCCCCTGCGCCGGCACCAT                3'
                                              |||||||
miR-150-3p                           3'     GACAGGGGGUCCGGACAUGGUC                5'

Position 2142-2167 of IRS1 CDS       5'      AGCTGTGGACAAACGGGGTAGGGGGC           3'
                                                            |||| |||
miR-150-5p                           3'          GUGACCAUGUUCCCAACCCUCU           5'

Position 3501-3527 of IRS1 CDS       5'      GGCTGAGGCCTGGGGAGCTTGGGGGAG          3'
                                                               ||||| ||
miR-150-5p                           3'          GUGACCAUGUUCCCAACCCUCU           5'

Position 3546-3576 of IRS1 CDS       5'      AACTGTGTGGGGCTGCTGGGGGTTTGGAGAA      3'
                                                                ||||| |||||
miR-150-5p                           3'              GUGACCAUGUUCCCAACCCUCU       5'

Position 5499-5518 of IRS1 3'UTR     5'         GGGGCCAGGGTTGGAGGGC               3'
                                                      ||||||||
miR-150-5p                           3'     GUGACCAUGUUCCCAACCCUCU                5'

Position 1724-1746 of PSPH 3'UTR     5'      TCTGTTGCCCAGGCTGGAGTGCA              3'
                                                    |||||||
miR-150-3p                           3'       GACAGGGGGUCCGGACAUGGUC              5'

Position 1774-1796 of PSPH 3'UTR     5'      TCTGCCTCCCAGGTTCACGCCAT              3'
                                                    ||||||
miR-150-3p                           3'       GACAGGGGGUCCGGACAUGGUC              5'

Position 2253-2272 of PSPH 3'UTR     5'      GCCCCCAGGCAAGAAACAGG                 3'
                                              |||||||||
miR-150-3p                           3'   GACAGGGGGUCCGGACAUGGUC                  5'

Position 2480-2505 of PSPH 3'UTR     5'      TTTGGGCTCAGGCCTGTAATCTCAGC           3'
                                                     ||||||||||
miR-150-3p                           3'      GACAGGGGGUCCGGACAUGGUC               5'

Position 2893-2909 of LRIG2 CDS      5'      GCACTGGTACCCGGGTG                    3'
                                              |||||||||
miR-150-5p                           3'       GUGACCAUGUUCCCAACCCUCU              5'

Position 3900-3941 of LRIG2 CDS      5'      GGTCTTTGGCAAGAATCCCAGGCTTGACCAACTGGTACCAGG    3'
                                                             ||||||| ||
miR-150-3p                           3'                GACAGGGGGUCCGGACAUGGUC     5'

Position 690-715 of ARMC9 CDS        5'      CTCTTCCAGGATTCCTGGACTCCAGA           3'
                                                          |||| ||
miR-150-3p                           3'       GACAGGGGGUCCGGACAUGGUC              5'

Position 985-1026 of ARMC9 CDS       5'      AGTACCTCCAGAGCGTCTGTGTCCGCCTGTTCAGTAACCAGA    3'
                                                                     |||||| |
miR-150-3p                           3'                   GACAGGGGGUCCGGACAUGGUC  5'
```

Table S6. Comprehensive analysis of miR-150-5p/3p and five predicted targets.

Gene Symbol	Number of binding sites (miRWalk)				Protein expression level (fold change)		Pearson's correlation analysis	
	miR-150-5p		miR-150-3p		Lv-mir-150-A2780 /Lv-mir-NC-A2780	*P* value	r	*P* value
	CDS	3'UTR	CDS	3'UTR				
IRS1	3	1	1	1	0.809898	1.698800E-03	-0.343878	9.050000E-09
PSPH	*NA*	*NA*	*NA*	4	0.797771	7.139150E-04	-0.285290	2.350000E-06
LRIG2	1	*NA*	*NA*	1	0.476913	5.825560E-32	-0.252999	3.090000E-05
ARMC9	*NA*	*NA*	2	*NA*	0.806437	1.335840E-03	-0.278616	4.110000E-06
SKP2	*NA*	*NA*	*NA*	*NA*	0.731830	1.669770E-06	-0.258941	1.970000E-05
IGF1R	2	1	*NA*	1	*NA*	*NA*	-0.296771	8.680000E-07
